# The potential use of *Azolla pinnata* as an alternative bio-insecticide

**DOI:** 10.1038/s41598-020-75054-0

**Published:** 2020-11-06

**Authors:** Rajiv Ravi, Dinesh Rajendran, Wen-Da Oh, Mohd Sukhairi Mat Rasat, Zulhazman Hamzah, Intan H. Ishak, Mohamad Faiz Mohd Amin

**Affiliations:** 1 School of Biological Sciences, Faculty of Science and Technology, Quest International University, 30250 Ipoh, Perak Malaysia; 2 School of Biological Sciences, Universiti Sains Malaysia, 11800 Gelugor, Penang Malaysia; 3 Vector Control and Research Unit, School of Biological Sciences, Universiti Sains Malaysia, 11800 Gelugor, Penang Malaysia; 4 School of Chemisty, Universiti Sains Malaysia, 11800 Gelugor, Penang Malaysia; 5 Faculty of Earth Science, Universiti Malaysia Kelantan, Jeli Campus, 17600 Jeli, Kelantan Malaysia

**Keywords:** Biochemistry, Chemical biology

## Abstract

Four different tests showed the effectiveness of *Azolla pinnata* plant extracts against *Aedes aegypti* and *Aedes albopictus* mosquitoes*.* In the adulticidal test, there was a significant increase in mortality as test concentration increases and *A. pinnata* extracts showed LC_50_ and LC_95_ values of 2572.45 and 6100.74 ppm, respectively, against *Ae. aegypti* and LC_50_ and LC_95_ values of 2329.34 and 5315.86 ppm, respectively, against *Ae. albopictus.* The ovicidal test showed 100% eggs mortality for both species tested for all the concentrations tested at 1500 ppm, 1000 ppm, 500 ppm, 250 ppm and 125 ppm. Both tested samples of *Ae. aegypti* and *Ae. albopictus* did not lay any eggs in the plastic cups filled with the *A. pinnata* extract but instead opted to lay eggs in the plastic cups filled with water during the oviposition deterrence test. Similarly, the non-choice test of *Ae. aegypti* mosquitoes laid eggs on the sucrose solution meant for the nutrient source of the mosquitoes instead of in the plastic cup that was designed to facilitate oviposition filled with the extract. This clearly indicates the presence of bioactive compounds which are responsible in adulticidal and ovicidal activity in *Aedes* mosquitoes and at the same time inducing repellence towards the mosquitoes. The LC–MS results showed mainly three important chemical compounds from *A. pinnata* extracts such as 1-(O-alpha-D-glucopyranosyl)-(1,3R,25R)-hexacosanetriol, Pyridate and Nicotinamide N-oxide. All these chemicals have been used for various applications such as both emulsion and non-emulsion type of cosmetics, against mosquito vector such as *Culex pipens* and *Anopheles* spp. Finally, the overall view of these chemical components from *A. pinnata* extracts has shown the potential for developing natural product against dengue vectors.

## Introduction

Dengue is transmitted by viral infected female mosquitoes known as *Aedes albopictus* and *Aedes aegypti*, primary vectors for vector-borne diseases which affect humans^1^. Dengue is known as one of the most common infections for humans, which is transmitted through bites of infected *Aedes* mosquitoes. It is considered to be a major health problem in tropical and subtropical countries^2^. Currently, Malaysia has recorded 80,000 dengue cases, with 113 deaths between January and August 2019^3^.

Physical control method is always a daunting task as it provides temporary solutions and is currently used as equipment’s such as bed nets, clothes and mosquito rackets^4^. Meanwhile, the use of chemical method such as temephos and pyrethroids is more prominent but it has resistance challenges. Problems of resistance in mosquitoes towards the application of permethrin and temephos has been reported in two major cities in Malaysia, namely Kuala Lumpur and Penang^5,6^. The insecticide resistances in mosquitoes are caused by factors of alterations in its targeted sites and the increase in insecticide metabolisms rate^7^.

Current application of the synthetic chemical controls and its constant repetitive applications have resulted in resistant mosquitoes and environmental pollutions^8^. Thus, the limited success of biocontrol programs on *Aedes* has encouraged the necessity for new insecticide search. Plant products have produced positive outcomes as an alternative to chemicals in dengue vector control programs. With this concept in mind, the use of *Azolla pinnata* has been recently reported against *Aedes* vectors^4,6,8–10^. Most of *A. pinnata* studies were only conducted for *Aedes* larvicidal effects and for its chemical compositions analyzed with emitted compounds by gas chromatographic techniques. Our previous findings revealed that the most effective *A. pinnata* extraction technique used was soxhlet extraction using methanol solvent in showing*Aedes* larvicidal activity. Thus, as a progression, this current study used the same extraction techniques^4,6,8^. Furthermore, previous findings of *A. pinnata* plant reiterates the killing mechanisms of *Aedes* larvae by direct contact applications or poison effects without being toxic to other organisms^4^. Thus, it is vital to investigate the applications of direct contact from *A. pinnata* plant extracts against adult *Aedes* mosquitoes and to its entire life cycle satges. Moreover, it is necessary to investigate the chemical constituent of *A. pinnata* in its liquid compositions, which may be responsible against *Aedes* mosquitoes’ interactions. Meanwhile, unlike conventional insecticides, the advantages of plant-derived insecticides composed by botanical blends of multiple chemical compounds is that it may act concertedly on both physiological and behavioural processes^11^.

The alternative uses of bio-insecticides will provide a more suitable and sustainable solution against *Ae. aegypti* and *Ae. albopictus.* In line with United Nations (UN) global sustainable goals and following a safer and greener alternative conception from *A. pinnata* plant, the purpose of this study is to evaluate the effectiveness of *A. pinnata* liquid extracts on *Aedes* adulticidal, ovicidal and ovipositional deterring activity and its liquid chemical compounds.

## Results

### Adulticidal activity

**Figure 1:**
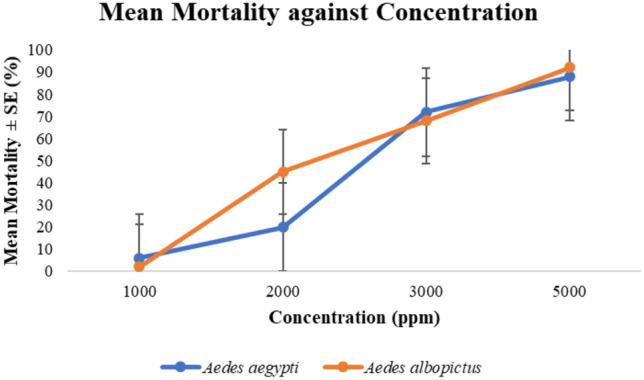
Dose response relationship of the different concentrations of *A. pinnata* extracts on the mortality on *Ae. aegypti* and *Ae. albopictus.*

As presented in Fig. 1, the adulticidal bioassay test showed a significant increase in mortality with increasing *A. pinnata* plant extract concentration. The lethal concentrations, LC_50_ and LC_95_, for *Ae. aegypti* were recorded at 2572.45 ppm and 6100.74 ppm, respectively, while testing on *Ae. albopictus* recorded the LC_50_ and LC_95_ values at 2329.34 ppm and 5315.86 ppm, respectively (Table 1). In the control assay, there was no significant mortality.

**Table 1:** Mean LC_50_ and LC_95_ (in ppm) adult efficacy of *A. pinnata* plant after 24 h of exposure on *Ae. aegypti* and *Ae. albopictus* (95% confidence limit).

Species	LC_50_	LC_95_	Regression equation
*Ae. aegypti*	2572.45	6100.74	Y = − 14.96 + 4.39X
*Ae. albopictus*	2329.34	5315.86	Y = − 17.55 + 1.55X

### Ovicidal activity

**Figure 2:**
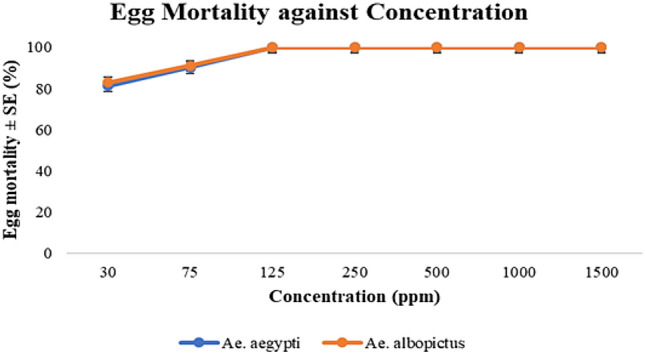
Graph of egg mortality (%) of *Ae. aegypti* and *Ae. albopictus* on different concentrations of *Azolla* extracts.

The ovicidal test showed that all the eggs of *Ae. aegypti* and *Ae. albopictus* possess mortality during the immersion into the trays containing *A. pinnata* plant extracts of varying concentrations as shown in Fig. 2 and Table 2. The concentration of 30 ppm caused 81% mortality for *Ae. aegypti* and 83% for *Ae. albopictus*. Furthermore, the concentration of 75 ppm caused 90% mortality for *Ae. aegypti* and 91% for *Ae. albopictus*. All the other concentrations recorded 100% mortality. Meanwhile, the full hatchability of *Aedes* eggs can be seen in control treatments.

**Table 2:** Egg mortality (%) of *Ae. aegypti* and *Ae. albopictus* on different concentrations of *Azolla* extracts.

Species	Control	Concentration (ppm)						
		30	75	125	250	500	1000	1500
*Ae. aegypti*	0	81	90	100	100	100	100	100
*Ae. albopictus*	0	83	91	100	100	100	100	100

### Oviposition deterrence assay

**Figure 3:**
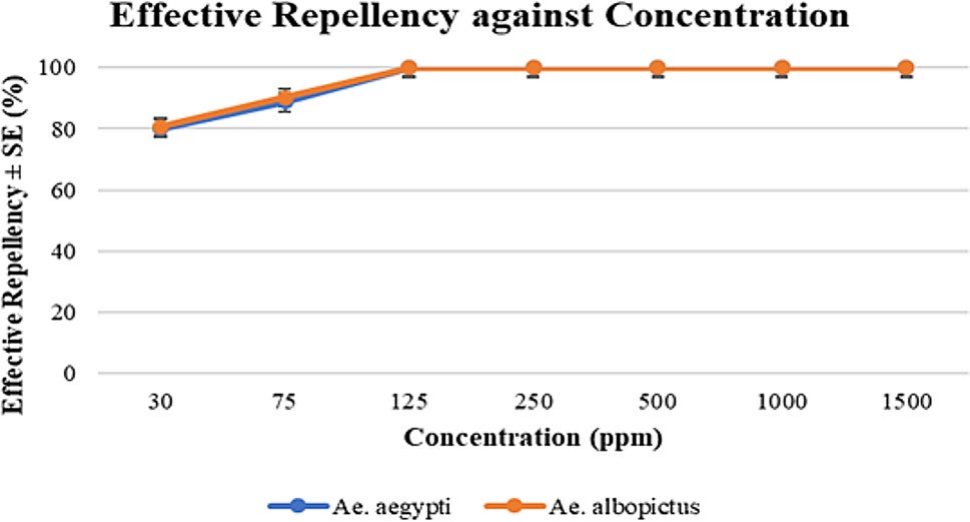
Graph of effective repellence of different concentrations of *Azolla* extracts on *Ae. aegypti* and *Ae. Albopictus.*

**Figure 4:**
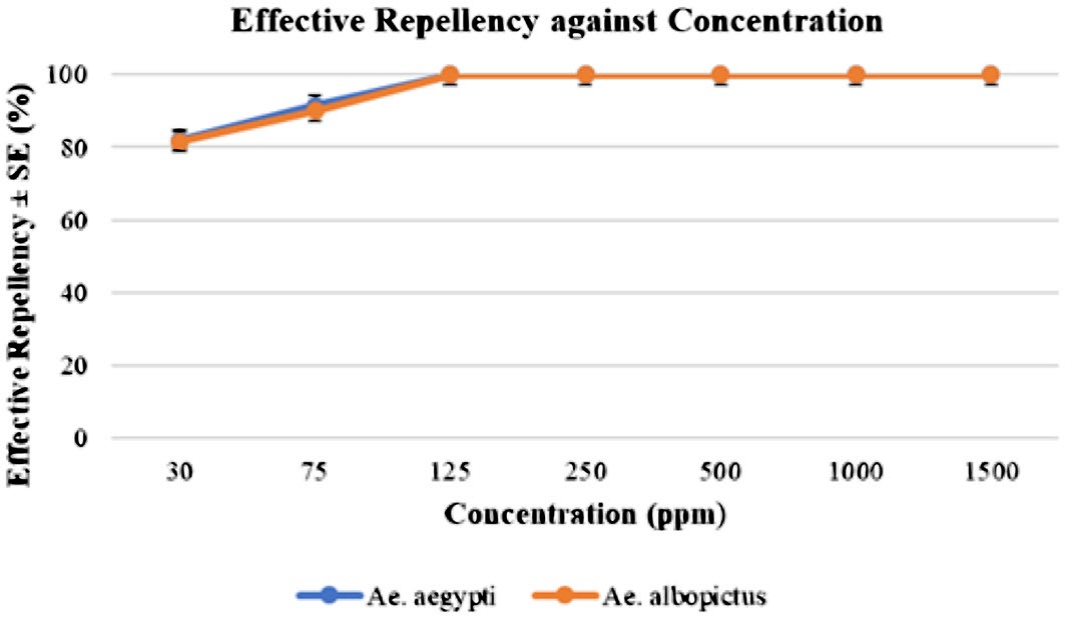
Graph of effective repellence of different concentrations of *Azolla* extracts on *Ae. aegypti* and *Ae. albopictus* (non-choice test).

**Figure 5:**
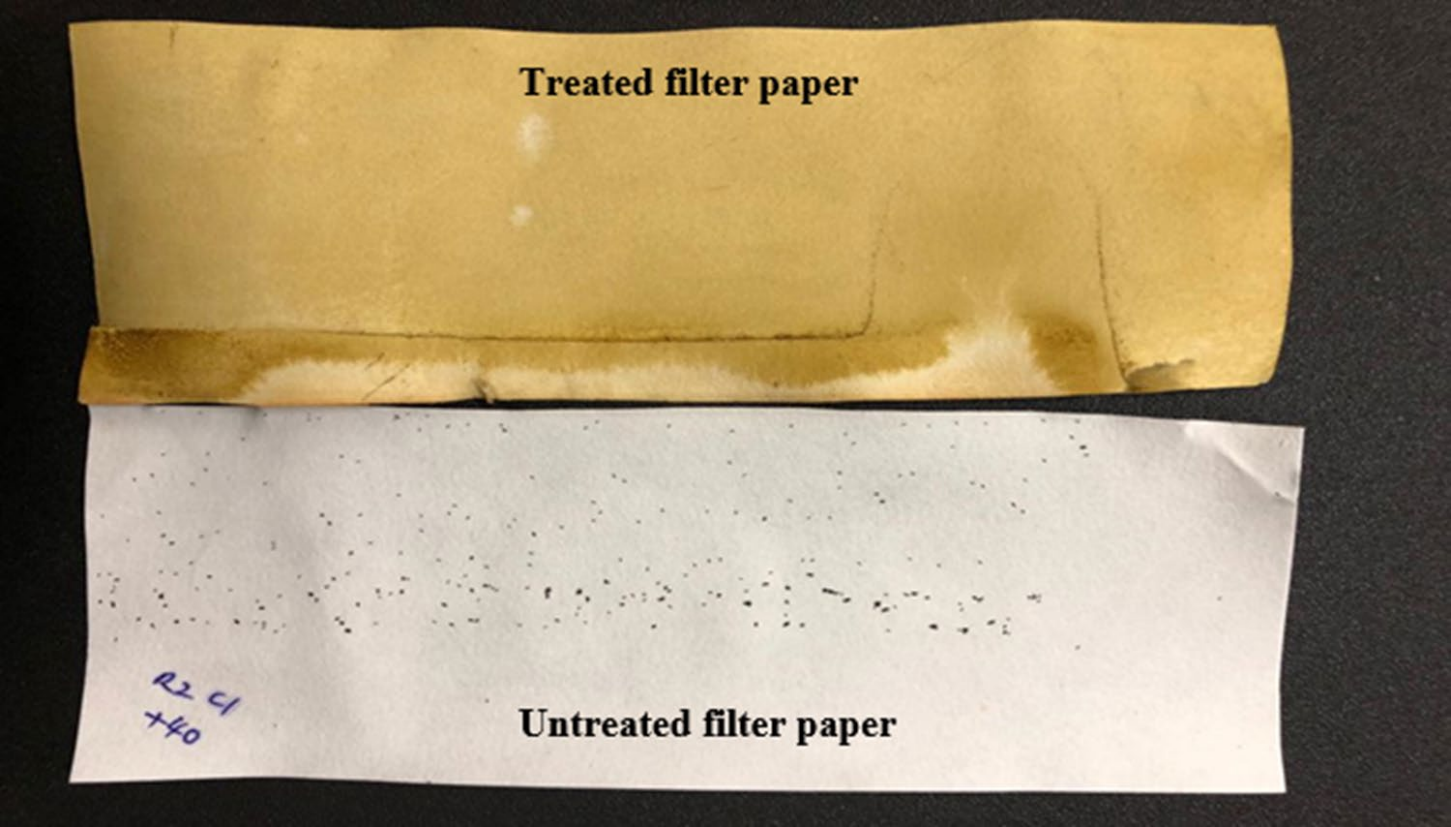
Eggs found on the untreated filter paper. No eggs found on the treated filter papers.

**Figure 6:**
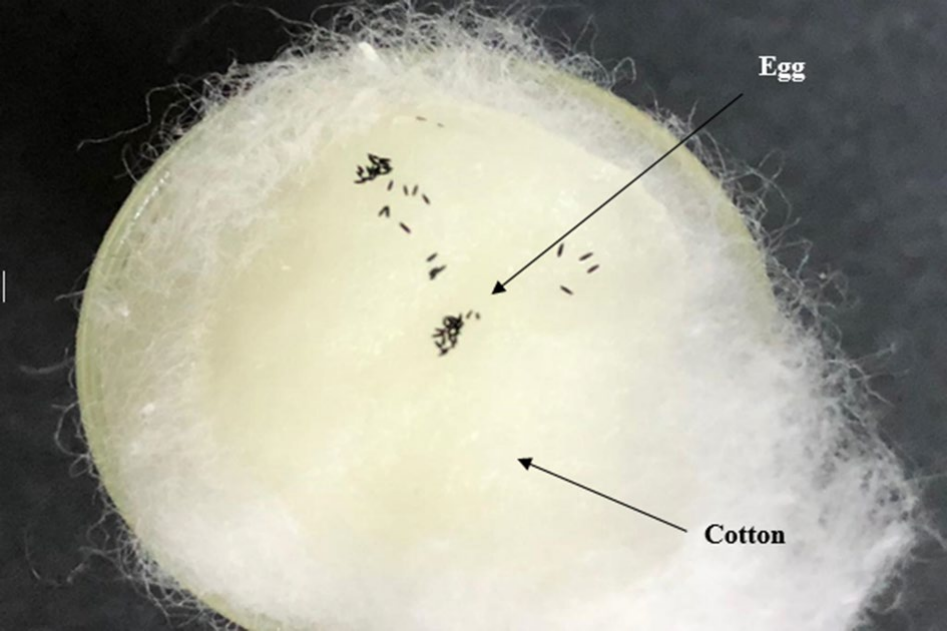
Eggs were found laid on the sucrose provided as a food sources in the non-choice oviposition test.

Both *Aedes* mosquitoes preferred to lay eggs only in the untreated cups. This can be clearly seen in Fig. 3, 4, 5, 6 and further proven through the data on Table 3 and 4 where by, the effective repellency is at 100% for all five concentrations. However, for choice test, *Ae. aegypti* and *Ae. albopictus* mosquitoes at a concentration of 30 ppm caused 80% repellence for *Ae. aegypti* and 81% for *Ae. albopictus*. The 75 ppm concentration caused 89% repellence for *Ae. aegypti* and 90% for *Ae. albopictus*. The non-choice test recorded 82% repellence for both *Ae. aegypti* and *Ae. albopictus* for 30 ppm concentration and at concentration 75 ppm caused 91% repellence for *Ae. aegypti* and 90% for *Ae. albopictus*. Interestingly, *Ae. aegypti* in the non-choice test laid eggs on sucrose solution which was placed as diet enrichment of mosquitoes as seen in Fig. 6.

**Table 3:** Effective repellence of different concentrations of *Azolla* extracts on *Ae. aegypti* and *Ae. albopictus.*

Species	Control	30	75	125	250	Concentration (ppm)		
						500	1000	1500
*Ae. aegypti*	0	80	89	100	100	100	100	100
*Ae. albopictus*	0	81	90	100	100	100	100	100

**Table 4:** Effective repellence of different concentrations of *Azolla* extracts on *Ae. aegypti* and *Ae. albopictus* (Non-choice test).

Species	Control	30	75	125	250	Concentration (ppm)		
						500	1000	1500
*Ae. aegypti*	0	82	91	100	100	100	100	100
*Ae. albopictus*	0	82	90	100	100	100	100	100

## Discussion

The experimental test showed that *A. pinnata* plant extracts can be used for *Aedes* adulticidal activities by impregnated paper method (method similarly used to test chemical insecticides). However, *A. pinnata* extract acted as relatively slow insecticidal chemical with moderate LC values when tested using *Ae. aegypti* and *Ae. albopictus.* During the adult bioassay tests, it can be observed that the tested mosquitoes tend to rest at the top of the bioassay kit instead of the impregnated papers. Thus, this suggests that the impregnated papers were repelling agent as well as killing agents for the mosquitoes. These findings can be confirmed by a study of Nsirim et al., (2013), which suggested that *A. pinnata* fresh plant possess repellent attributes which are commonly used in Southeast Nigeria for repelling mosquitoes. However, current study is the first study to test the crude extracts of *A. pinnata* plant instead of fresh plant applications.

Furthermore, findings of this current study have piqued our interest to further test on *A*. *pinnata* extract for ovicidal and oviposition deterrence. In current ovicidal test, there were no eggs hatched for higher than 125 ppm treated trays indicating, 100% egg mortality for all the concentrations of both *Ae. aegypti* and *Ae. albopictus.* Similarly results by Govindarajan et al., (2010), with the *Ervatamia coronaria* and *Caesalpinia pulcherrima* plant extracts caused 100% egg mortality on *Culex quinquefasciatus, Ae. aegypti* and *Anopheles stephensi* at higher than 300 ppm. Meanwhile, the eggs in the control treatment had 0% mortality when immersed in regular de-chlorinated water. Additionally, the eggs mortality was 80% till 90% for *A*. *pinnata* extract concentration of 30 ppm and 75 ppm. These proved that even at the lowest concentration,the efficacy was high in causing *Aedes* eggs mortality.

The oviposition deterrence test proved that the eggs can only be observed in the plain water medium and not in *A. pinnata* plant extracts. Additionally, the non-choice test shows that without any choice or options, the *Ae. aegypti* and *Ae. albopictus* laid their eggs on the cotton soaked with sucrose solution. As discussed earlier, it is obvious that the methanolic extract of *A. pinnata* had repellence properties. Thus, at 500 ppm concentration of plant extract, the *Aedes* mosquitoes will not lay their eggs in test cups.

In this current study, all the results of adulticidal, ovicidal, oviposition deterrence were between concentrations of 125 ppm till 6100 ppm. Meanwhile, previous larvicidal results by Ravi et al., (2018) on *A*. *pinnata* extract reported lethal concentration of 1000 ppm till 1500 ppm. In an overall view, the concentration of LC_50_ for adulticidal, 2572.45 ppm can be used for all the assays against *Aedes* mosquitoes. Thus, a single concentration of *A. pinnata* plant extracts will be effective for further field-based applications and it may serve as alternative for vector control strategies. In order to proceed with field-based applications, some contribution of knowledge is required on the chemical compositions from *A. pinnata* plant extracts.

The LC–MS results (Table 5) have shown the presence of various compounds in the *A. pinnata* extracts which can be difficult to determined. In this study, the three most likely active compounds with promising ovicidal and repellency activities, namely 1-(O-alpha-D-glucopyranosyl)-(1,3R,25R)-hexacosanetriol, pyridate and nicotinamide N-oxide were selectively identified. Other compounds were not likely to contribute to the ovicidal and repellency activities. According to Kawasaki et al., (2006), chemical compound of 1-(O-alpha-D-glucopyranosyl)-(1,3R,25R)-hexacosanetriol is used for antibacterial cosmetic composition which is easily applied to both emulsion and non-emulsion type of cosmetics. Thus,1-(O-alpha-D-glucopyranosyl)-(1,3R,25R)-hexacosanetriol compound has the potential to be used as a natural ingredient for formulations in developing mosquito repellence creams in near future. Furthermore, pyridate compound were also used in several studies against mosquito vector such as *Culex pipens* and has been proven effective^12^. Besides that, nicotinamide N-oxide is used as insecticides in commercialized products and tested to be effective against *Anopheles*^13^. Our previous studies on *A. Pinnata* have recorded several interesting chemical compounds with insecticidal properties such as *n*-hexadecanoic acid, diethyl phthalate, neophytadiene, 3,7,11,15-tetramethyl-2-hexadecen-1-ol^4,8^ . The chemical analysis conducted in previous studies of *A. Pinnata* were based on GC–MS analysis which differs compared to the current study due to the progressive experimental efforts in classifying all the volatile compounds, as LC–MS analysis is suitable for compounds of lower volatility. The overall view of these chemical components from *A. pinnata* extract has shown the potential for developing natural product against dengue vectors.

**Table 5:** Liquid Chromatography-Mass Spectrometry (LCMS), chemical compounds, retention time, molecular weight and properties *of A. pinnata* extract.

Chemical compounds	Retention time	Molecular weight	Properties
1-(O-alpha-D-glucopyranosyl)-(1,3R,25R)-hexacosanetriol	23.805	576.4623	Metabolite^16^, antibacterial^17^
Pyridate	3.771	378.1163	Pesticide^18^
Nicotinamide N-oxide	4.064	138.0427	Antagonist of the CXCR2 receptor, insecticides^19^

## Methodology

### Plant extraction

*Azolla pinnata* plant were collected at Kuala Krai, Kelantan (50 31′ N 1020 12′ E), dried and blended. The fresh plant sample was then extracted using Soxhlet extraction technique (Favorit, Malaysia). Briefly, 40 g dried plant powder sample was weighed, and then placed on a paper thimble. Cotton wools were placed on the upper part of the thimble to prevent the sample from evaporating. Exactly 1 L, of methanol solvent was placed in the round-bottom flask with the heating mantle underneath. The extraction in soxhlet apparatus were at boiling point 70 °C for about 3 h until the solvent in the siphon arm becomes clear, indicating complete extraction process. The extracts were then evaporated in the vacuum evaporator in order. The crude extracts were kept at − 20 °C until further used.

### *Aedes* rearing

Eggs of *Aedes aegypti* and *Aedes albopictus* were obtained from Vector Control Research Unit (VCRU) at Universiti Sains Malaysia (USM), Penang, Malaysia. We follow the method used by Ahbirami et al., (2014) in larvae rearing. The eggs were hatched in de-chlorinated water for 24 h and maintained at 25–30 °C, pH 6.95 to 7.03, and relative humidity of 80 ± 10% and dissolved oxygen of 5.5–6.1 mg/L in the laboratory. The larvae were then reared until they pupated. The pupae were then transferred into mosquito cages to allow the mosquitoes to emerge. Sucrose solution was provided in the mosquito cage to provide food source for the mosquitoes.

### Adulticidal activity

**Figure 7:**
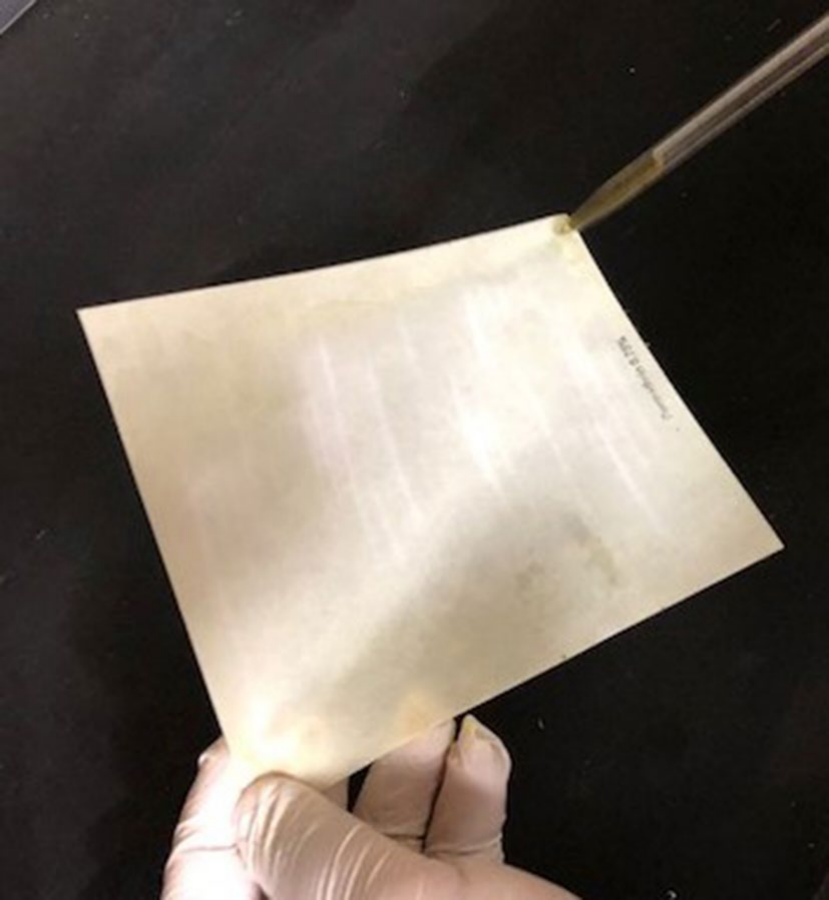
Plant extracts were impregnated on filter papers (140 × 120 mm).

The paper impregnation method was adopted from Kovendan et al., (2013). The *Azolla pinnata* plant extracts were diluted with methanol to make the different concentrations (30, 75, 125, 250, 500, 1000, 1500, 2000, 3000, 5000, 6000 ppm) prepared. Next, as shown in Fig. 7, the diluted plant extracts were impregnated on filter papers (140 × 120 mm)^14^. A blank paper consisting of only methanol was used as control^14^. All the papers were left to dry at room temperature to evaporate off the methanol overnight^14^. The methods for the adulticidal activity were adapted from WHO, 1997. Female, *Ae. aegypti* and *Ae. albopictus* mosquitoes aging 2–5 days old of each strain were used in the separate test as they are fully developed and have reached sexual maturity at this age. A total of 25 female mosquitoes were collected with an aspirator and were placed in each holding tubes of the bioassay kits. The holding tubes were left for an hour acclimation time to check on any weak or unhealthy mosquitoes that might die in the tube. Dead mosquitoes were replaced with new ones and the test was continued. Strips of impregnated papers with different concentrations of methanolic *A. pinnata* extracts were rolled into the exposure tubes of the bioassay kits. The mosquitoes in the holding tubes were then blown into the exposure tubes and left in the exposure tube for an hour (Fig. 2).

The numbers of the mosquitoes knocked-down were recorded at an interval of 5 min throughout the 60 min. The knockdown of mosquitoes was evaluated with the inability of the mosquitoes to stand up or fly (Jahangir et al., 2008). After 1 h, all 25 mosquitoes were then collected and placed in a paper cup with cotton bud soaked in sucrose solution containing 10% of vitamin B complex and was left for 24 h. The mortality of among the 25 post-treated mosquitoes was obtained. A negative control test was done with impregnated paper containing oil where no mortality was recorded. After the bioassay test, the remaining surviving mosquitoes after 24 h were taken out and were used for the subsequent test. A total of four replicates were done for each species and solvent used to obtain a better data set.

### Ovicidal activity

The methodology for the ovicidal activity was followed by Su and Mulla (1998) with slight modification. Total of 100 eggs of *Ae. aegypti* and *Ae. albopictus* were immersed in 6 trays. 5 trays filled with methanolic *A. pinnata* extracts at different concentrations; 1500 ppm, 1000 ppm, 500 ppm, 250 ppm and 125 ppm. Control cup was filled with distilled water. The test was conducted in 5 replicates for each concentration. After treatment, the eggs from each treatment were counted under microscope and transferred into distilled water cup for hatching assessments. The percentage of eggs mortality was calculated based on the non-hatchability of eggs with unopened opercula. The hatch rates were assessed by 48 h post-treatment using formula by Govindarajan (2011);

$$ {\text{Egg }}\;{\text{mortality }}\left( \% \right) \, = \, \left( {{\text{number}}\;{\text{ of }}\;{\text{unhatched }}\;{\text{larvae}}/{\text{total}}\;{\text{number}}\;{\text{of}}\;{\text{eggs}}} \right) \, \times { 1}00\% $$

### Oviposition deterrence activities

The oviposition deterrent activity was conducted in a laboratory using the method of Reegan et al., (2013)^15^ and a dual-choice oviposition bioassay was performed on gravid females of *Ae. aegypti.* Fifteen, gravid females (5 days old) of *Ae. aegypti* were introduced into an insect cage (30 cm × 30 cm × 30 cm) under room conditions. The adults were provided with 10% glucose solution which was available at all time. Two 50 mL plastic cups were filled with dechlorinated water and two 50 mL plastic cups were filled with methanolic *A. pinnata* extracts for oviposition. Different concentrations of 1500 ppm, 1000 ppm and 500 ppm of a plant extract solutions were used in different cages. A support for oviposition was provided by placing a piece of filter paper (Whatman No.1) on the inner surface of each plastic cup so that the lower half of it was submerged in the treated solution or untreated solution for the whole paper to get moistened while the upper half of it was above the solution where the mosquitoes would lay their eggs on. The untreated and treated cups were placed at alternate diagonally opposite locations for each replicate to nullify any effect of their locations on oviposition. After 3 days, the numbers of eggs laid in the treated and untreated cups were counted under a stereomicroscope. A non-choice test, was also conducted by placing one 50 mL plastic cup filled with *A. pinnata* and placed with filter paper into the mosquito cage with 15 gravid female *Aedes* mosquitoes. The test was conducted with different concentrations as well. Percent effective repellency (ER percentage) for each oviposition repellent concentration was calculated using the following formula from Xue et al., (2001):

$$ {\text{ER }} = \, \left( {\left( {{\text{NC}} - {\text{NT}}} \right) \, /{\text{ NC}}} \right) \, \times { 1}00\% $$

where ER = percent effective repellency; NC = number of eggs in control; and NT = number of eggs in treatment.

### Liquid chromatography–mass spectrometry (LCMS)

LC–MS was performed with Shimadzu LCMS systems using Agilent C18 column (I.D. 2.0 × 150 mm,), at a flow-rate of 0.25 ml min^−1^ , 250 °C. The eluent was MeCN/H2O/HCOOH (3 : 92 : 5). Expected Empirical Formula: Polar Compounds (40–1300amu) mass range.

## Conclusion

In conclusion, the *A. pinnata* plant extracts showed promising results in adulticidal and ovicidal test against *Ae. aegypti* and *Ae. albopictus.* The oviposition deterrence test showed that there are repellent properties present from the *A. pinnata* extract. With the identification of chemical compounds in the *A. pinnata* extract, it can be developed as bio-insecticides for *Aedes* mosquito vector control. This study also suggests that future research work can be conducted on the field applications of *A. pinnata* extracts for its long-term effects on other non-target organisms, including on human health.

## Supplementary information

Supplementary Information
