# Supplementary material for: The potential use of Azolla pinnata as an alternative bio-insecticide
Source: Sci Rep. 2020 Nov 6;10:19245. doi: 10.1038/s41598-020-75054-0 (PMC7648075; doi:10.1038/s41598-020-75054-0)
Supplement: Supplementary file 1 — Supplementary Information [file 41598_2020_75054_MOESM1_ESM.pdf]

The potential use of Azolla pinnata as an alternative bio-insecticide

Rajiv Ravi[1], Dinesh Rajendran[2,3], Wen-Da Oh[4], Mohd Sukhairi Mat Rasat[6], Zulhazman Hamzah[5], Intan H. Ishak[2,3*], Mohamad Faiz Mohd Amin[5]*

[1]School of Biological Sciences, Faculty of Science and Technology, Quest International University, Jalan Raja Permaisuri Bainun, 30250 Ipoh, Perak, Malaysia.
[2]School of Biological Sciences, Universiti Sains Malaysia, 11800 Penang, Malaysia.
[3]Vector Control Research Unit, School of Biological Sciences, Universiti Sains Malaysia, 11800 Penang, Malaysia.
[4]School of Chemical Sciences, Universiti Sains Malaysia, 11800 Penang, Malaysia.
[5]Faculty of Earth Science, Universiti Malaysia Kelantan, Jeli Campus, 17600 Jeli, Kelantan, Malaysia
[6]Faculty of Bioengineering and Technology, Universiti Malaysia Kelantan, Jeli Campus, 17600 Jeli, Kelantan, Malaysia

Correspondence and requests for materials should be addressed to M.F.M.A, I.H.I (email: mohamadfaiz@umk.edu.my, intanishak@usm.my)

Qualitative Compound Report

**Data File**	M1.d	**Sample Name**	M1
**Sample Type**	Sample	**Position**	P1-A1
**Instrument Name**	Instrument 1	**User Name**	
**Acq Method**	RAJIV MEOH POS.m	**Acquired Time**	21-Dec-18 10:48:09 AM
**IRM Calibration Status**	Success	**DA Method**	1.m
**Comment**			
**Sample Group**		**Info.**	
**Acquisition SW Version**	6200 series TOF/6500 series Q-TOF B.05.01 (B5125.1)		

Fragmentor Voltage 175 **Collision Energy** 0 **Ionization Mode** ESI

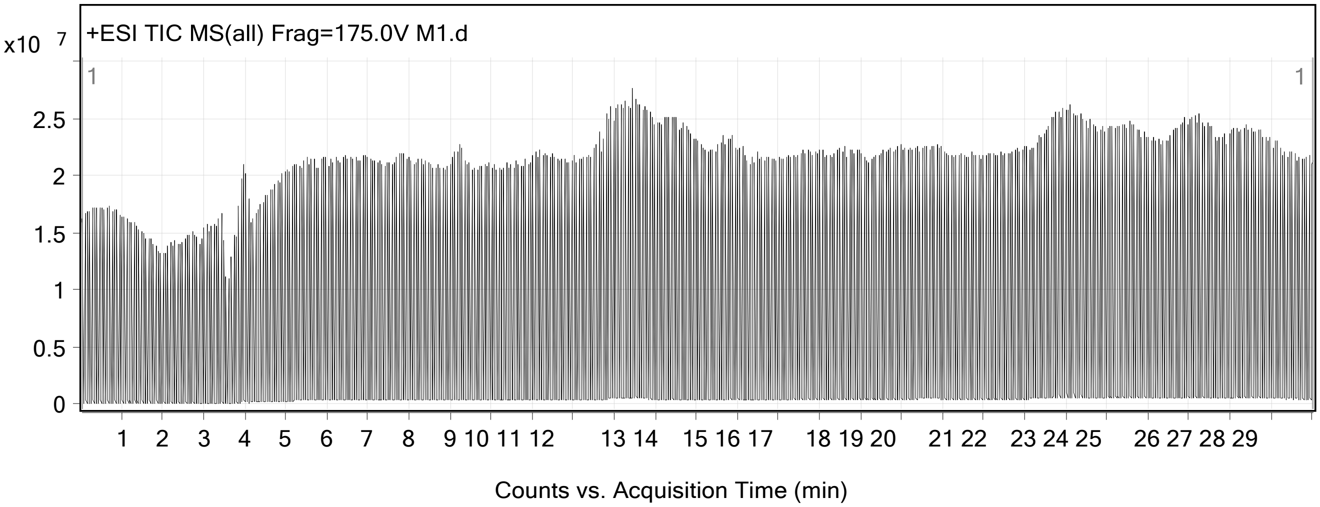

Fragmentor Voltage 175 **Collision Energy** 0 **Ionization Mode** ESI

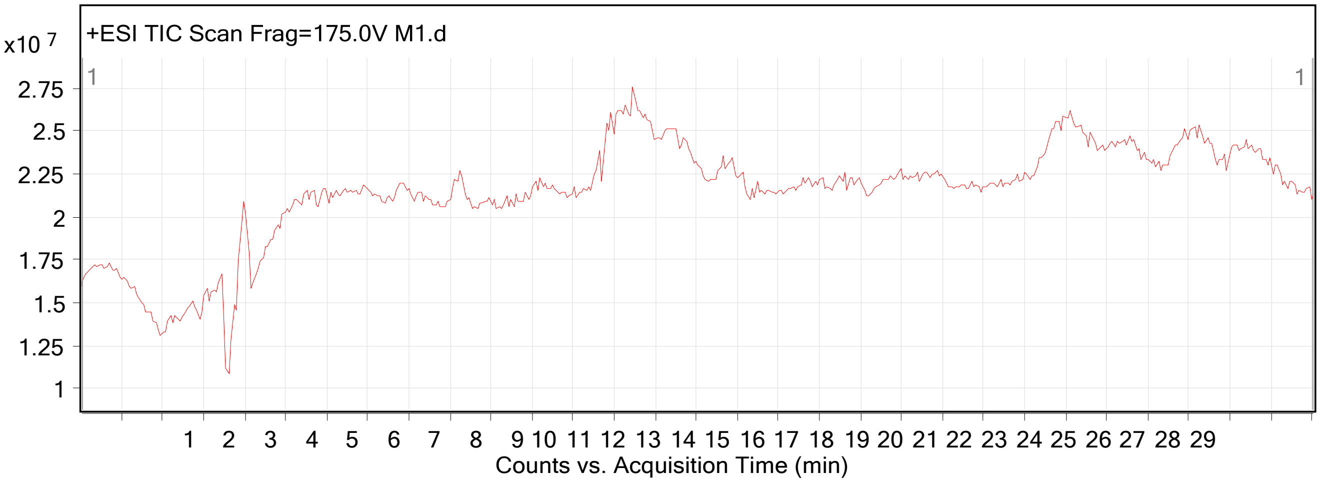

Compound Table

Compound Label	RT	Mass	Abund	Name	MFG Formula	MFG Diff (ppm)	DB Formula	DB Diff (ppm)
Cpd 1: C24 H46 N3 O12	6.78	568.3092	763846		C24 H46 N3 O12	-1.81		
Cpd 2: C26 H48 O13	7.076	568.3093			C26 H48 O13	0.38		

Cpd 3: C26 H48 O13	7.373	568.3093			C26 H48 O13	0.33		
Cpd 4: C28 H52 O14	7.389	612.3357			C28 H52 O14	0.01		
Cpd 5: C26 H48 O13	7.669	568.3092			C26 H48 O13	0.49		
Cpd 6: C28 H52 O14	7.686	612.3356			C28 H52 O14	0.23		
Cpd 7: C24 H46 N3 O12	7.966	568.3091			C24 H46 N3 O12	-1.65		
Cpd 8: C28 H52 O14	7.982	612.3354			C28 H52 O14	0.44		
Cpd 9: C24 H46 N3 O12	8.262	568.3091			C24 H46 N3 O12	-1.76		
Cpd 10: C28 H52 O14	8.279	612.3355			C28 H52 O14	0.36		
Cpd 11: C24 H46 N3 O12	8.559	568.309			C24 H46 N3 O12	-1.51		
Cpd 12: C26 H50 N3 O13	8.575	612.3354			C26 H50 N3 O13	-1.61		
Cpd 13: C24 H46 N3 O12	8.855	568.3088			C24 H46 N3 O12	-1.13		
Cpd 14: C26 H50 N3 O13	8.872	612.3351	780661		C26 H50 N3 O13	-1.28		
Cpd 15: C26 H48 O13	9.152	568.3092			C26 H48 O13	0.5		
Cpd 16: C24 H46 N3 O12	9.448	568.3087			C24 H46 N3 O12	-0.99		
Cpd 17: C24 H46 N3 O12	9.744	568.3092			C24 H46 N3 O12	-1.83		
Cpd 18: C28 H52 O14	9.761	612.3356	759748		C28 H52 O14	0.18		
Cpd 19: C24 H46 N3 O12	10.041	568.3089			C24 H46 N3 O12	-1.32		
Cpd 20: C26 H50 N3 O13	10.057	612.3353	761718		C26 H50 N3 O13	-1.59		
Cpd 21: C24 H46 N3 O12	10.337	568.309			C24 H46 N3 O12	-1.58		
Cpd 22: C28 H52 O14	10.354	612.3355			C28 H52 O14	0.37		
Cpd 23: C24 H46 N3 O12	10.634	568.3092			C24 H46 N3 O12	-1.85		
Cpd 24: C28 H52 O14	10.65	612.3356			C28 H52 O14	0.15		
Cpd 25: C26 H48 O13	10.93	568.3094			C26 H48 O13	0.08		
Cpd 26: C24 H46 N3 O12	11.227	568.3092			C24 H46 N3 O12	-1.88		
Cpd 27: C26 H48 O13	11.523	568.3096			C26 H48 O13	-0.12		
Cpd 28: C26 H48 O13	11.82	568.3093			C26 H48 O13	0.41		
Cpd 29: C26 H48 O13	12.116	568.3095			C26 H48 O13	-0.04		
Cpd 30: C26 H48 O13	12.413	568.3093			C26 H48 O13	0.3		
Cpd 31: C25 H48 N3 O6	12.777	486.355			C25 H48 N3 O6	-1.51		
Cpd 32: C23 H44 N3 O5	12.806	442.3289			C23 H44 N3 O5	-1.75		
Cpd 33: C25 H48 N3 O6	13.065	486.3551			C25 H48 N3 O6	-1.58		
Cpd 34: C23 H44 N3 O5	13.081	442.3288			C23 H44 N3 O5	-1.7		
Cpd 35: C25 H48 N3 O6	13.361	486.3551			C25 H48 N3 O6	-1.63		
Cpd 36: C23 H44 N3 O5	13.378	442.3288	1142531		C23 H44 N3 O5	-1.52		
Cpd 37: C25 H48 N3 O6	13.658	486.3552			C25 H48 N3 O6	-1.75		
Cpd 38: C23 H44 N3 O5	13.674	442.3288			C23 H44 N3 O5	-1.49		
Cpd 39: C25 H46 O6	13.941	442.3291			C25 H46 O6	0.67		
Cpd 40: C25 H48 N3 O6	13.954	486.3553			C25 H48 N3 O6	-2.1		
Cpd 41: C24 H46 N3 O12	14.149	568.3092			C24 H46 N3 O12	-1.84		
Cpd 42: C25 H48 N3 O6	14.251	486.3551			C25 H48 N3 O6	-1.59		
Cpd 43: C23 H44 N3 O5	14.386	442.3289			C23 H44 N3 O5	-1.75		
Cpd 44: C25 H48 N3 O6	14.547	486.3551			C25 H48 N3 O6	-1.66		
Cpd 45: C26 H48 O13	14.911	568.3094			C26 H48 O13	0.14		
Cpd 46: C26 H48 O13	15.199	568.3094			C26 H48 O13	0.18		
Cpd 47: C28 H52 O14	15.394	612.3355			C28 H52 O14	0.32		
Cpd 48: C26 H48 O13	15.496	568.3093			C26 H48 O13	0.35		
Cpd 49: C28 H52 O14	15.69	612.3356			C28 H52 O14	0.13		
Cpd 50: C24 H46 N3 O12	15.792	568.3091			C24 H46 N3 O12	-1.6		
Cpd 51: C28 H52 O14	15.987	612.3356			C28 H52 O14	0.22		
Cpd 52: C24 H46 N3 O12	16.089	568.3092			C24 H46 N3 O12	-1.77		
Cpd 53: C28 H52 O14	16.283	612.3357			C28 H52 O14	0.07		
Cpd 54: C24 H46 N3 O12	16.385	568.3093			C24 H46 N3 O12	-1.95		
Cpd 55: C26 H50 N3 O13	16.58	612.3354			C26 H50 N3 O13	-1.74		
Cpd 56: C24 H46 N3 O12	16.682	568.3092			C24 H46 N3 O12	-1.87		
Cpd 57: C28 H52 O14	16.876	612.3356			C28 H52 O14	0.12		
Cpd 58: C24 H46 N3 O12	16.978	568.3091			C24 H46 N3 O12	-1.63		
Cpd 59: C28 H52 O14	17.173	612.3354			C28 H52 O14	0.51		
Cpd 60: C24 H46 N3 O12	17.274	568.3089			C24 H46 N3 O12	-1.4		
Cpd 61: C28 H52 O14	17.469	612.3355			C28 H52 O14	0.35		
Cpd 62: C24 H46 N3 O12	17.571	568.309			C24 H46 N3 O12	-1.41		
Cpd 63: C28 H52 O14	17.766	612.3356			C28 H52 O14	0.21		
Cpd 64: C26 H48 O13	17.868	568.3093			C26 H48 O13	0.35		
Cpd 65: C28 H52 O14	18.062	612.3359			C28 H52 O14	-0.32		
Cpd 66: C26 H48 O13	18.164	568.3094			C26 H48 O13	0.25		
Cpd 67: C28 H52 O14	18.359	612.3356			C28 H52 O14	0.1		
Cpd 68: C24 H46 N3 O12	18.46	568.3091			C24 H46 N3 O12	-1.69		
Cpd 69: C28 H52 O14	18.655	612.3356			C28 H52 O14	0.19		
Cpd 70: C24 H46 N3 O12	18.757	568.3092			C24 H46 N3 O12	-1.94		
Cpd 71: C28 H52 O14	18.951	612.3357			C28 H52 O14	0.09		
Cpd 72: C24 H46 N3 O12	19.053	568.3092			C24 H46 N3 O12	-1.94		
Cpd 73: C28 H52 O14	19.248	612.3354			C28 H52 O14	0.48		
Cpd 74: C24 H46 N3 O12	19.35	568.3089			C24 H46 N3 O12	-1.35		
Cpd 75: C28 H52 O14	19.544	612.3355			C28 H52 O14	0.38		
Cpd 76: C24 H46 N3 O12	19.646	568.3092			C24 H46 N3 O12	-1.82		
Cpd 77: C28 H52 O14	19.841	612.3355			C28 H52 O14	0.4		
Cpd 78: C24 H46 N3 O12	19.943	568.3092			C24 H46 N3 O12	-1.88		
Cpd 79: C28 H52 O14	20.137	612.3358			C28 H52 O14	-0.14		
Cpd 80: C26 H48 O13	20.239	568.3092			C26 H48 O13	0.55		
Cpd 81: C28 H52 O14	20.434	612.3356			C28 H52 O14	0.19		
Cpd 82: C24 H46 N3 O12	20.536	568.3091			C24 H46 N3 O12	-1.66		
Cpd 83: C28 H52 O14	20.73	612.3356			C28 H52 O14	0.18		
Cpd 84: C26 H48 O13	20.832	568.3092			C26 H48 O13	0.44		
Cpd 85: C28 H52 O14	21.027	612.3357			C28 H52 O14	-0.02		
Cpd 86: C24 H46 N3 O12	21.129	568.3092			C24 H46 N3 O12	-1.82		
Cpd 87: C28 H52 O14	21.323	612.3358			C28 H52 O14	-0.21		
Cpd 88: C26 H48 O13	21.425	568.3095			C26 H48 O13	0.07		
Cpd 89: C28 H52 O14	21.62	612.3355			C28 H52 O14	0.38		
Cpd 90: C24 H46 N3 O12	21.722	568.3088			C24 H46 N3 O12	-1.18		
Cpd 91: C28 H52 O14	21.916	612.3358			C28 H52 O14	-0.11		
Cpd 92: C24 H46 N3 O12	22.018	568.3092			C24 H46 N3 O12	-1.89		
Cpd 93: C28 H52 O14	22.213	612.3356			C28 H52 O14	0.25		
Cpd 94: C26 H48 O13	22.315	568.3093			C26 H48 O13	0.25		
Cpd 95: C28 H52 O14	22.509	612.3359			C28 H52 O14	-0.24		

Cpd 96: C24 H46 N3 O12	22.611	568.3091			C24 H46 N3 O12	-1.66		
Cpd 97: C28 H52 O14	22.806	612.3354			C28 H52 O14	0.48		
Cpd 98: C24 H46 N3 O12	22.907	568.3091			C24 H46 N3 O12	-1.6		
Cpd 99: C28 H52 O14	23.102	612.3356			C28 H52 O14	0.1		
Cpd 100: C24 H46 N3 O12	23.204	568.3092			C24 H46 N3 O12	-1.77		
Cpd 101: C28 H52 O14	23.398	612.3357			C28 H52 O14	0.05		
Cpd 102: C26 H48 O13	23.5	568.3097			C26 H48 O13	-0.29		
Cpd 103: C28 H52 O14	23.606	612.3364			C28 H52 O14	-1.06		
Cpd 104: C26 H48 O13	23.776	568.3094			C26 H48 O13	0.16		
Cpd 105: 1-(O-alpha-D-glucopyranosyl)-(1,3R,25R)-hexacosanetriol	23.805	576.4623		1-(O-alpha-D-glucopyranosyl)-(1,3R,25R)-hexacosanetriol	C32 H64 O8	-3.84	C32 H64 O8	-3.84
Cpd 106: C26 H48 O13	24.051	568.3094			C26 H48 O13	0.22		
Cpd 107: 1-(O-alpha-D-glucopyranosyl)-(1,3R,25R)-hexacosanetriol	24.093	576.4627		1-(O-alpha-D-glucopyranosyl)-(1,3R,25R)-hexacosanetriol	C32 H64 O8	-4.5	C32 H64 O8	-4.5
Cpd 108: C26 H48 O13	24.347	568.3096			C26 H48 O13	-0.14		
Cpd 109: 1-(O-alpha-D-glucopyranosyl)-(1,3R,25R)-hexacosanetriol	24.39	576.4624		1-(O-alpha-D-glucopyranosyl)-(1,3R,25R)-hexacosanetriol	C32 H64 O8	-3.92	C32 H64 O8	-3.92
Cpd 110: 1-(O-alpha-D-glucopyranosyl)-(1,3R,25R)-hexacosanetriol	24.606	576.4626		1-(O-alpha-D-glucopyranosyl)-(1,3R,25R)-hexacosanetriol	C32 H64 O8	-4.27	C32 H64 O8	-4.27
Cpd 111: C26 H48 O13	24.635	568.3095			C26 H48 O13	0.04		
Cpd 112: C28 H52 O14	24.821	612.3358			C28 H52 O14	-0.14		
Cpd 113: C26 H48 O13	24.923	568.3095			C26 H48 O13	0.07		
Cpd 114: C28 H52 O14	25.118	612.3355			C28 H52 O14	0.39		
Cpd 115: C26 H48 O13	25.22	568.3095			C26 H48 O13	0.03		
Cpd 116: C28 H52 O14	25.414	612.3362			C28 H52 O14	-0.85		
Cpd 117: C26 H48 O13	25.516	568.3099			C26 H48 O13	-0.73		
Cpd 118: C28 H52 O14	25.711	612.3359			C28 H52 O14	-0.27		
Cpd 119: C26 H48 O13	25.813	568.3096			C26 H48 O13	-0.22		
Cpd 120: C28 H52 O14	26.007	612.3357	1077429		C28 H52 O14	0.02		
Cpd 121: C26 H48 O13	26.109	568.3095			C26 H48 O13	-0.04		
Cpd 122: C28 H52 O14	26.215	612.3362	1003813		C28 H52 O14	-0.88		
Cpd 123: C26 H48 O13	26.406	568.3095			C26 H48 O13	0.05		
Cpd 124: C24 H44 O12	26.422	524.2832			C24 H44 O12	0.15		
Cpd 125: C26 H48 O13	26.702	568.3096			C26 H48 O13	-0.13		
Cpd 126: C24 H44 O12	26.719	524.2832			C24 H44 O12	0.2		
Cpd 127: C24 H46 N3 O12	26.999	568.3091			C24 H46 N3 O12	-1.76		
Cpd 128: C22 H42 N3 O11	27.015	524.2827			C22 H42 N3 O11	-1.5		
Cpd 129: C26 H48 O13	27.295	568.3093			C26 H48 O13	0.29		
Cpd 130: C22 H42 N3 O11	27.312	524.2829			C22 H42 N3 O11	-1.93		
Cpd 131: C26 H48 O13	27.592	568.3094			C26 H48 O13	0.25		
Cpd 132: C22 H42 N3 O11	27.608	524.283			C22 H42 N3 O11	-2.03		
Cpd 133: C22 H42 N3 O11	27.845	524.2825			C22 H42 N3 O11	-1.08		
Cpd 134: C26 H48 O13	27.888	568.3094	1198499		C26 H48 O13	0.19		
Cpd 135: C28 H52 O14	27.905	612.3358	1050410		C28 H52 O14	-0.13		
Cpd 136: C26 H48 O13	28.185	568.3096	1252735		C26 H48 O13	-0.14		
Cpd 137: C28 H52 O14	28.201	612.3358			C28 H52 O14	-0.2		
Cpd 138: C26 H48 O13	28.481	568.3095	1294616		C26 H48 O13	0.04		
Cpd 139: C28 H52 O14	28.498	612.3357			C28 H52 O14	0.02		
Cpd 140: C26 H48 O13	28.756	568.3096	1257625		C26 H48 O13	-0.27		
Cpd 141: C28 H52 O14	28.786	612.3358			C28 H52 O14	-0.17		
Cpd 142: C26 H48 O13	28.972	568.3095	1199141		C26 H48 O13	-0.04		
Cpd 143: C28 H52 O14	29.074	612.3356			C28 H52 O14	0.21		
Cpd 144: C22 H42 N3 O11	29.18	524.2828			C22 H42 N3 O11	-1.6		
Cpd 145: C26 H48 O13	29.319	568.3094	1095542		C26 H48 O13	0.24		
Cpd 146: C28 H52 O14	29.349	612.3357			C28 H52 O14	0.08		
Cpd 147: C24 H46 N3 O12	29.608	568.3092	1063629		C24 H46 N3 O12	-1.88		
Cpd 148: C28 H52 O14	29.624	612.3356			C28 H52 O14	0.23		
Cpd 149: C26 H48 O13	29.874	568.3093	1046445		C26 H48 O13	0.35		
Cpd 150: C28 H52 O14	29.891	612.3357			C28 H52 O14	-0.02		

**Compound Label**	***m/z***	**RT**	**Algorithm**	**Mass**
Cpd 1: C24 H46 N3 O12	569.3166	6.78	Auto MS/MS	568.3092

Compound Chromatograms

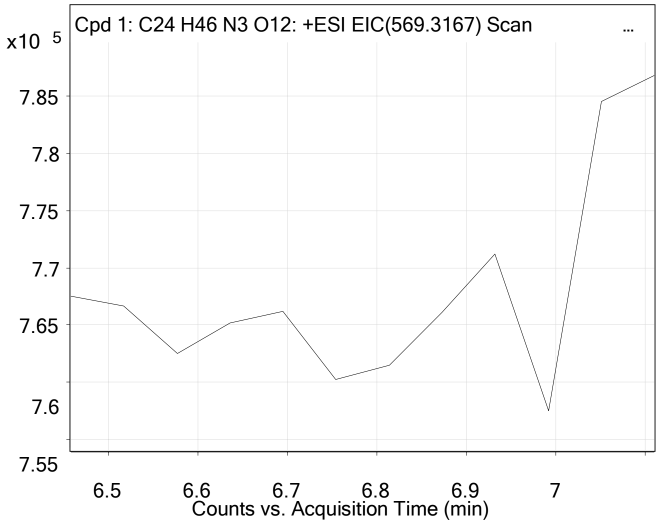

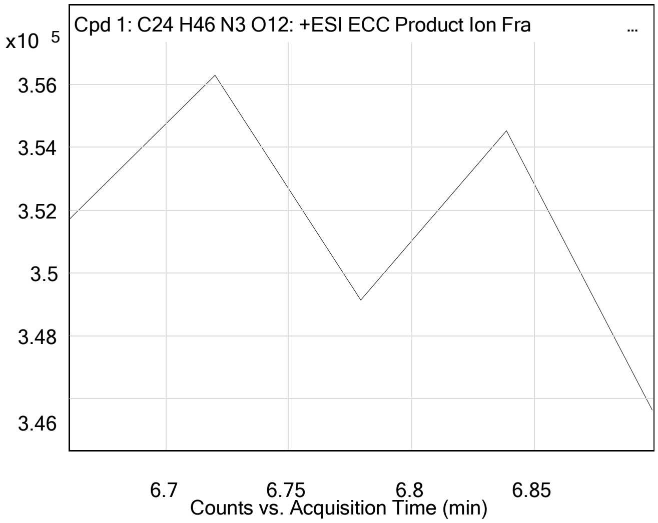

MS Spectrum

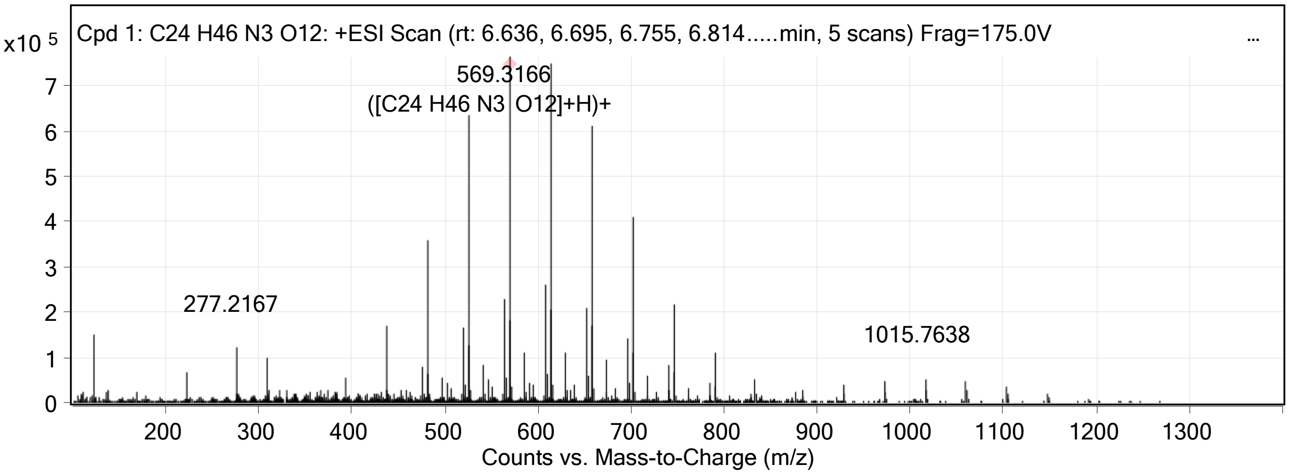

MS Zoomed Spectrum

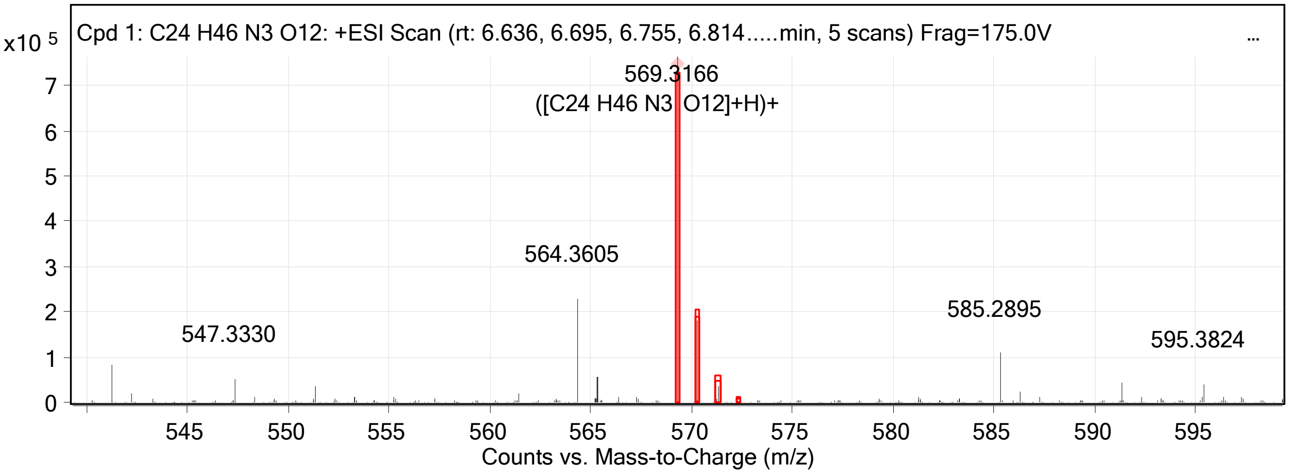

MS Spectrum Peak List

m/z	Calc *m/z*	Diff(ppm)	z	Abund	Formula	Ion
481.2634			1	360453.59		
525.2899			1	635024.13		
569.3166	569.3154	-2.11	1	763845.75	C24 H46 N3 O12	(M+H)+
570.3192	570.3186	-1.12	1	182956.59	C24 H46 N3 O12	(M+H)+
571.3204	571.3209	0.89	1	35173.77	C24 H46 N3 O12	(M+H)+
572.323	572.3235	0.98	1	5395.52	C24 H46 N3 O12	(M+H)+
608.3868			1	259448.13		
613.343			1	746702.31		
657.3691			1	611324.63		

MSMS Spectrum

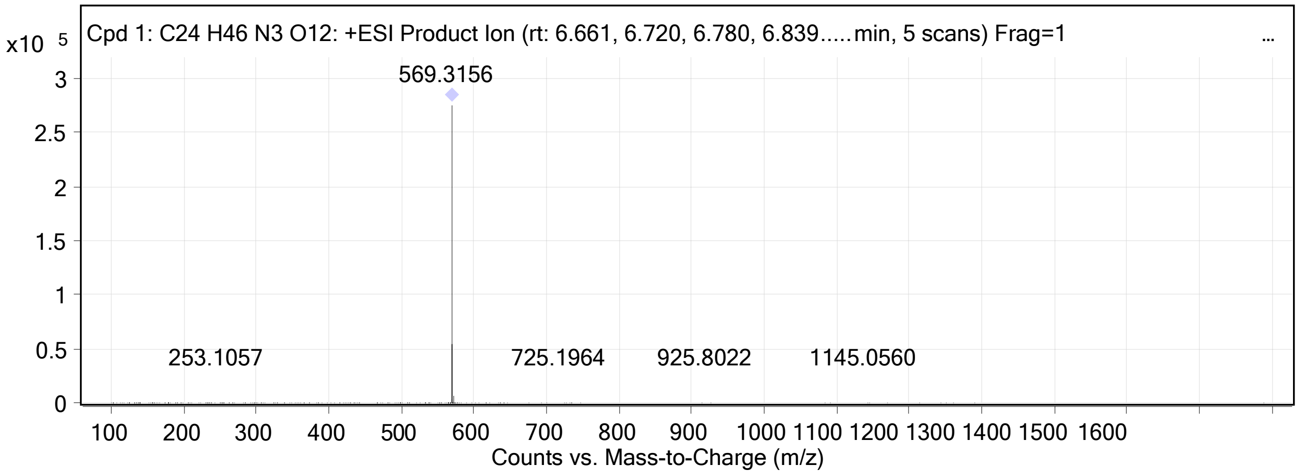

MS/MS Spectrum Peak List

m/z	*Calc m/z*	Diff(ppm)	z	Abund
123.0416	123.0427	9.05	1	62.5
137.0579	137.0584	3.32	1	52.39
181.084	181.0846	3.03		23.68
209.0807	209.0768	-18.37		31.15
221.1134	221.1132	-1.01		30.31
237.1122	237.1081	-17.25		20.51
253.1057	253.103	-10.5	1	46.32
309.2783	309.2788	1.74		24.28
566.2923	566.292	-0.62	1	41.6
569.3156	569.3154	-0.36	1	275670.47

Compound Label	*m/z*	RT	Algorithm	Mass
Cpd 2: C26 H48 O13	569.3168	7.076	Auto MS/MS	568.3093

Compound Chromatograms

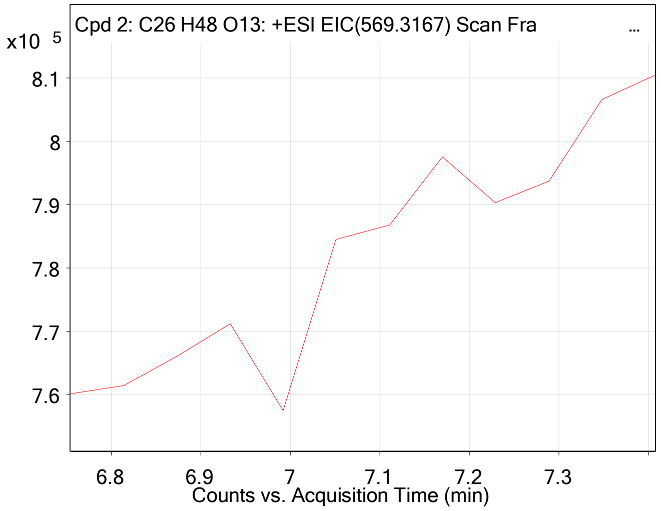

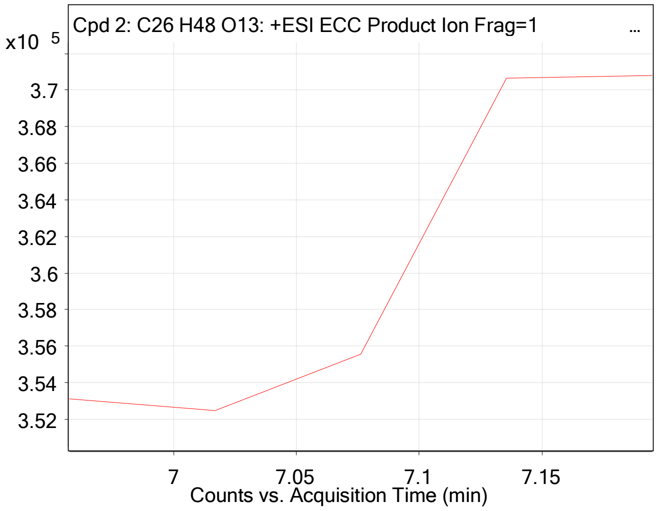

MS Spectrum

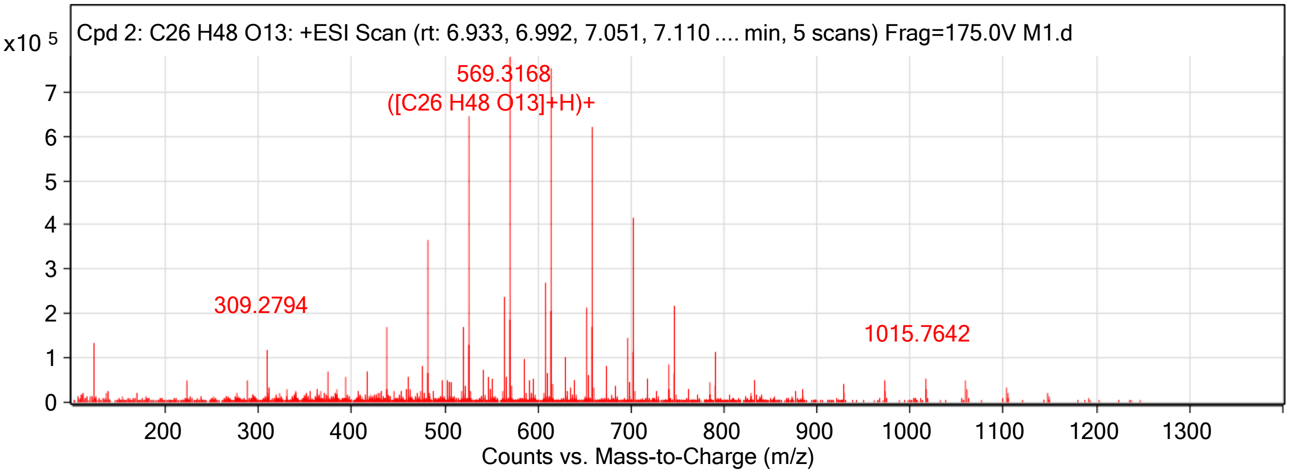

MS Zoomed Spectrum

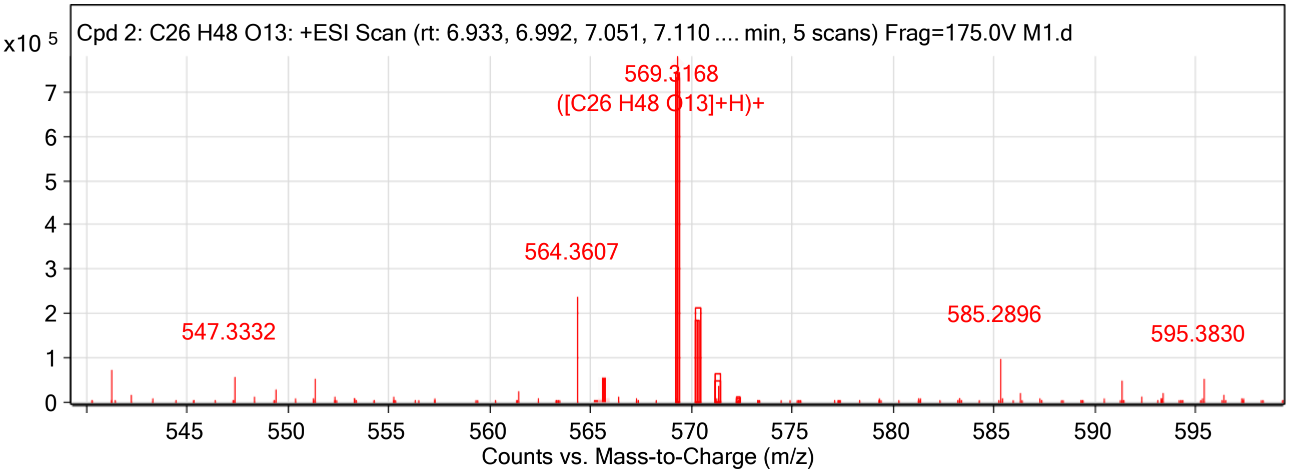

MS Spectrum Peak List

m/z	Calc *m/z*	Diff(ppm)	z	Abund	Formula	Ion
481.2635			1	365058.25		
525.2901			1	644685.44		
569.3168	569.3168	-0.03	1	779540.69	C26 H48 O13	(M+H)+
570.3193	570.3202	1.49	1	184214.08	C26 H48 O13	(M+H)+
571.3208	571.3226	3.17	1	35404.6	C26 H48 O13	(M+H)+
572.3233	572.3253	3.55	1	5395.84	C26 H48 O13	(M+H)+
608.387			1	267952.19		
613.3432			1	753142.5		
657.3692			1	620547.69		
701.3954			1	412605.53		

MSMS Spectrum

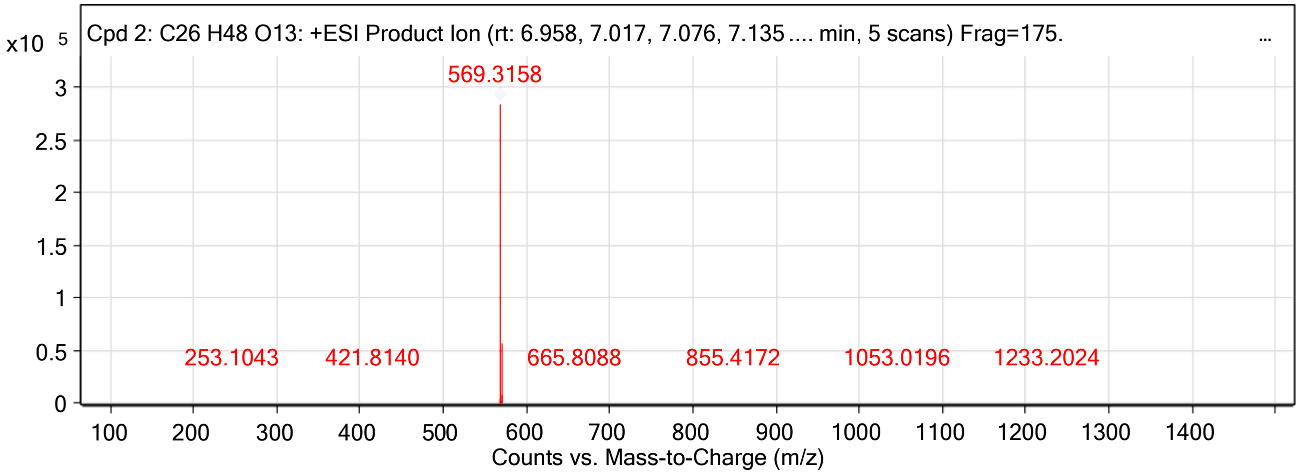

MS/MS Spectrum Peak List

m/z	*Calc m/z*	Diff(ppm)	z	Abund
113.0592	113.0597	4.69		14.42
123.0418	123.0441	18.06		72.87
137.0582	137.0597	10.63		21.84
139.0753	139.0754	0.05		14.65
175.1335	175.1329	-3.67		24.55
265.1693	265.1646	-17.78		19.75
309.2774	309.2788	4.65	1	17.28
566.285	566.2933	14.63	1	28.29
567.3067			1	16.31
569.3158	569.3168	1.75	1	283397.41

Compound Label	*m/z*	RT	Algorithm	Mass
Cpd 3: C26 H48 O13	569.3168	7.373	Auto MS/MS	568.3093

Compound Chromatograms

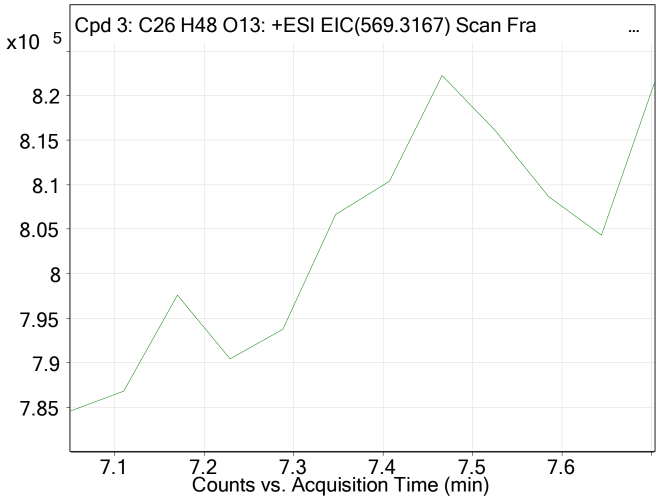

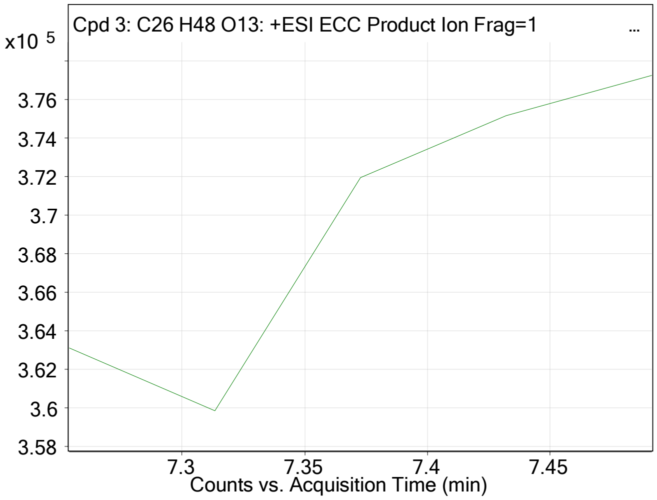

MS Spectrum

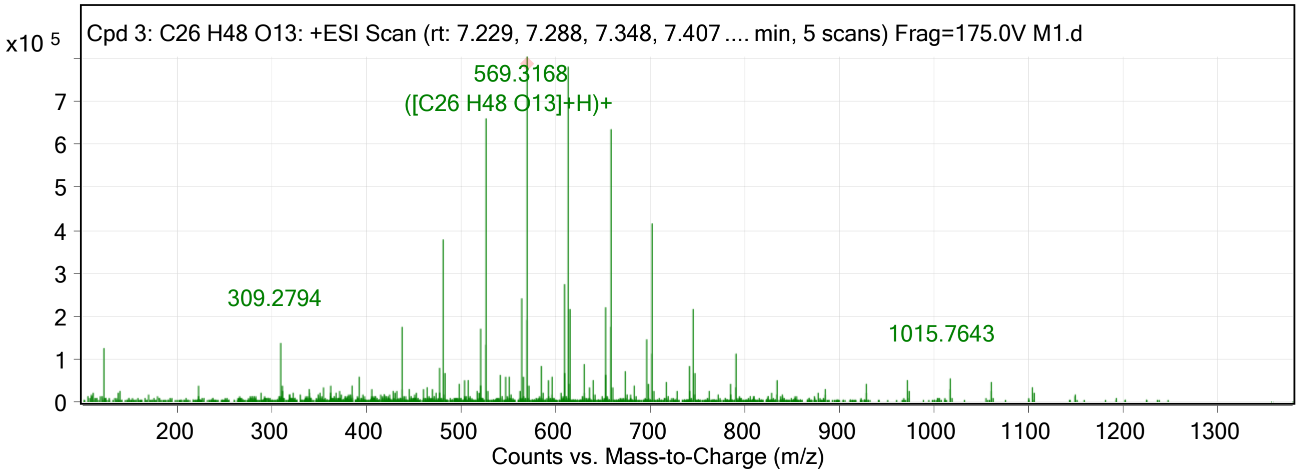

MS Zoomed Spectrum

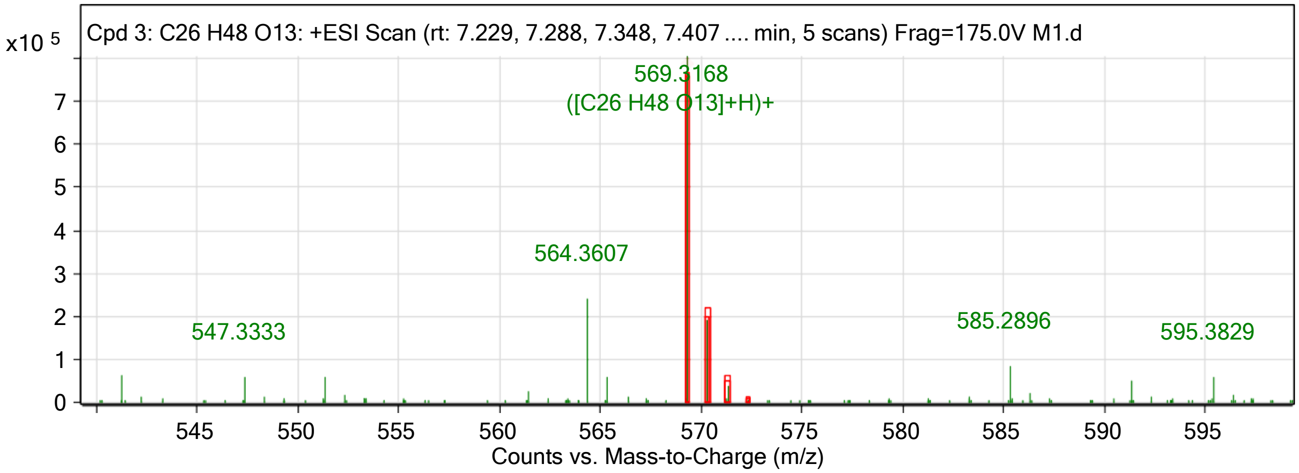

MS Spectrum Peak List

m/z	Calc *m/z*	Diff(ppm)	z	Abund	Formula	Ion
481.2636			1	375904.41		
525.2902			1	658268.69		
569.3168	569.3168	-0.09	1	804684.19	C26 H48 O13	(M+H)+
570.3194	570.3202	1.47	1	192350.2	C26 H48 O13	(M+H)+
571.3208	571.3226	3.06	1	35794.53	C26 H48 O13	(M+H)+
572.3232	572.3253	3.58	1	5368.08	C26 H48 O13	(M+H)+
608.3871			1	273130.44		
613.3432			1	778643.75		
657.3693			1	633454.88		
701.3954			1	417069.81		

MSMS Spectrum

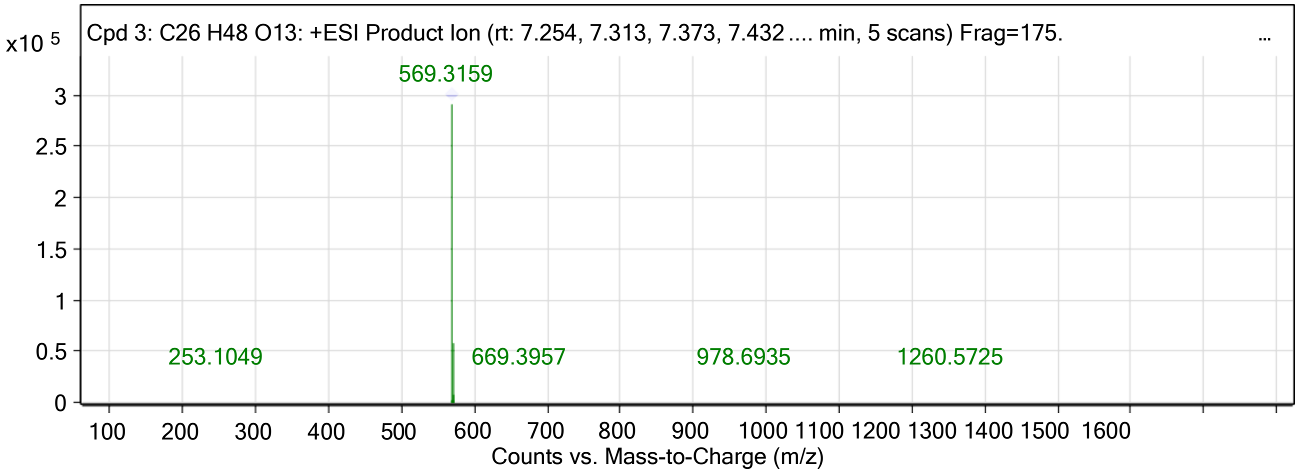

MS/MS Spectrum Peak List

m/z	*Calc m/z*	Diff(ppm)	z	Abund
123.0416	123.0441	19.75		32.08
123.0802	123.0804	2.23		22.43
137.058	137.0597	12.58		19.64
137.0951	137.0961	7.29		18.28
139.0754	139.0754	-0.56		18.33
175.1308	175.1329	11.82		13.59
195.0956	195.1016	30.61		15.54
283.2608	283.2632	8.47		16.79
553.2885	553.2855	-5.54		14.62
569.3159	569.3168	1.52	1	291079.69

Compound Label	*m/z*	RT	Algorithm	Mass
Cpd 4: C28 H52 O14	613.3432	7.389	Auto MS/MS	612.3357

Compound Chromatograms

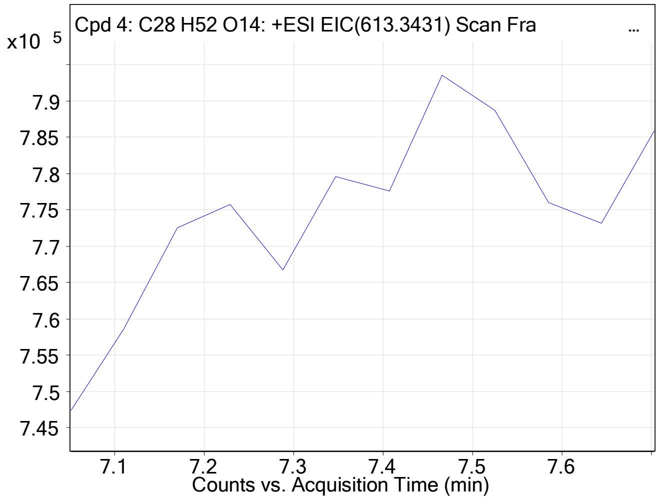

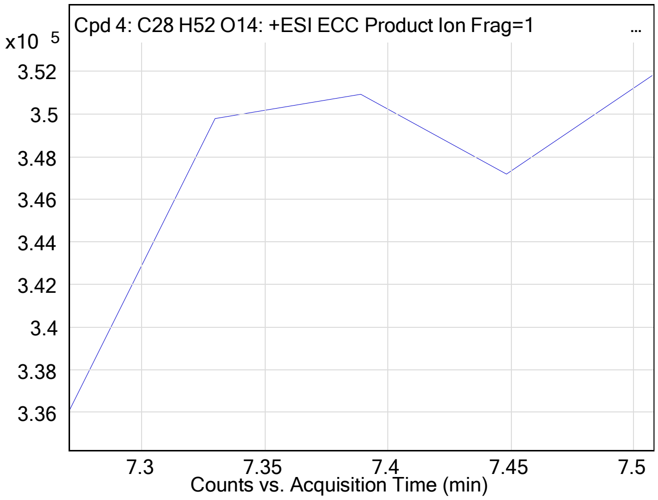

MS Spectrum

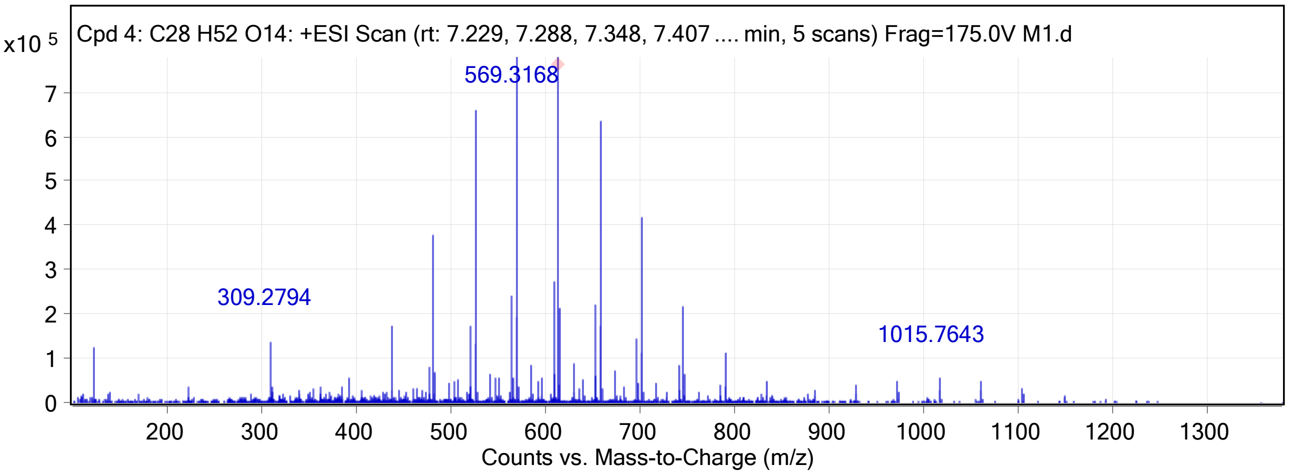

MS Zoomed Spectrum

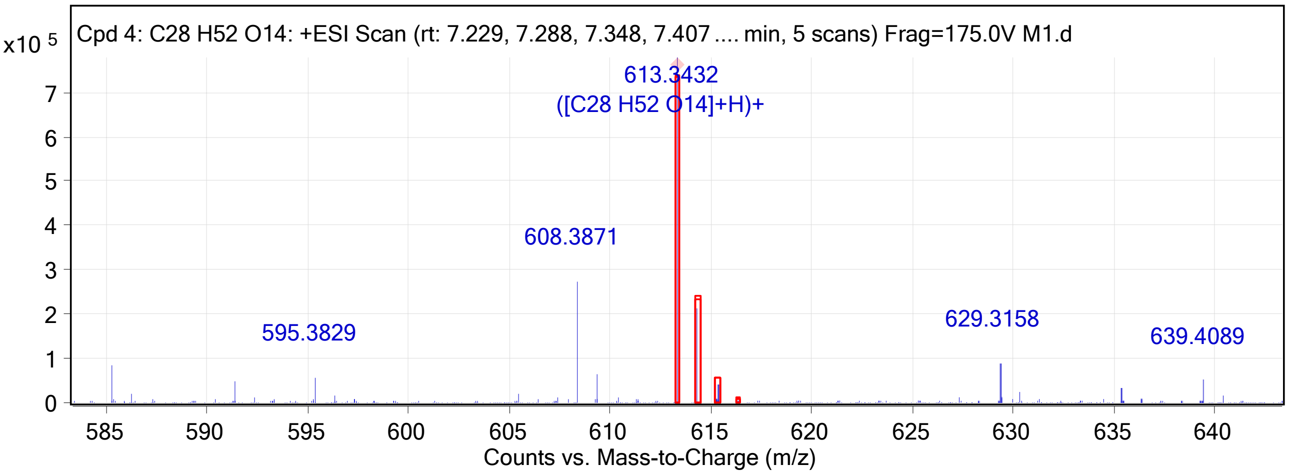

MS Spectrum Peak List

m/z	Calc *m/z*	Diff(ppm)	z	Abund	Formula	Ion
481.2636			1	375904.41		
525.2902			1	658268.69		
569.3168			1	804684.19		
608.3871			1	273130.44		
613.3432	613.343	-0.41	1	778643.75	C28 H52 O14	(M+H)+
614.3458	614.3464	1	1	214062.55	C28 H52 O14	(M+H)+
615.3474	615.3489	2.42	1	40915	C28 H52 O14	(M+H)+
616.3503	616.3515	2.02	1	5899.75	C28 H52 O14	(M+H)+

MSMS Spectrum

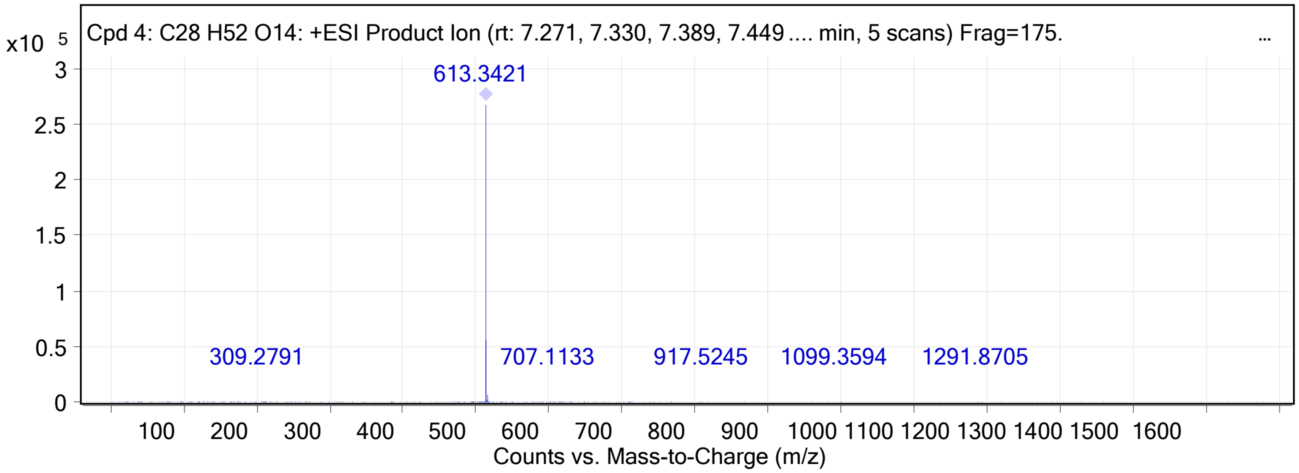

MS/MS Spectrum Peak List

m/z	*Calc m/z*	Diff(ppm)	z	Abund
111.116	111.1168	7.13		20.45
123.0416	123.0441	19.86		59.09
133.0865	133.0859	-4.06		42.84
137.0576	137.0597	15.3		49.66
181.0852	181.0859	3.98		22.86
221.1382	221.1384	0.67		37.26
305.1549	305.1595	14.95		20.98
309.2791	309.2788	-0.84	1	81
609.3179	609.3117	-10.14		16.11
613.3421	613.343	1.46	1	267731.75

Compound Label	*m/z*	RT	Algorithm	Mass
Cpd 5: C26 H48 O13	569.3167	7.669	Auto MS/MS	568.3092

Compound Chromatograms

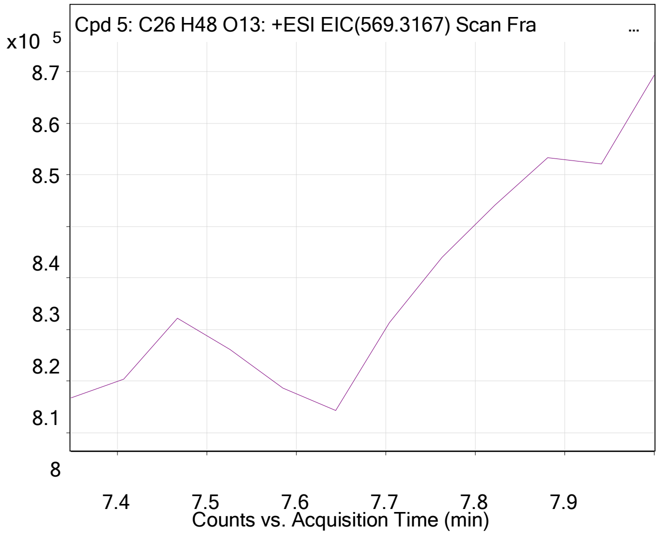

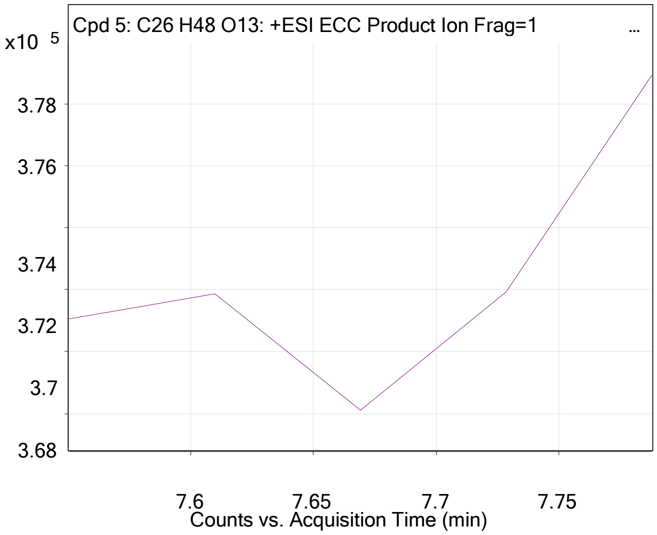

MS Spectrum

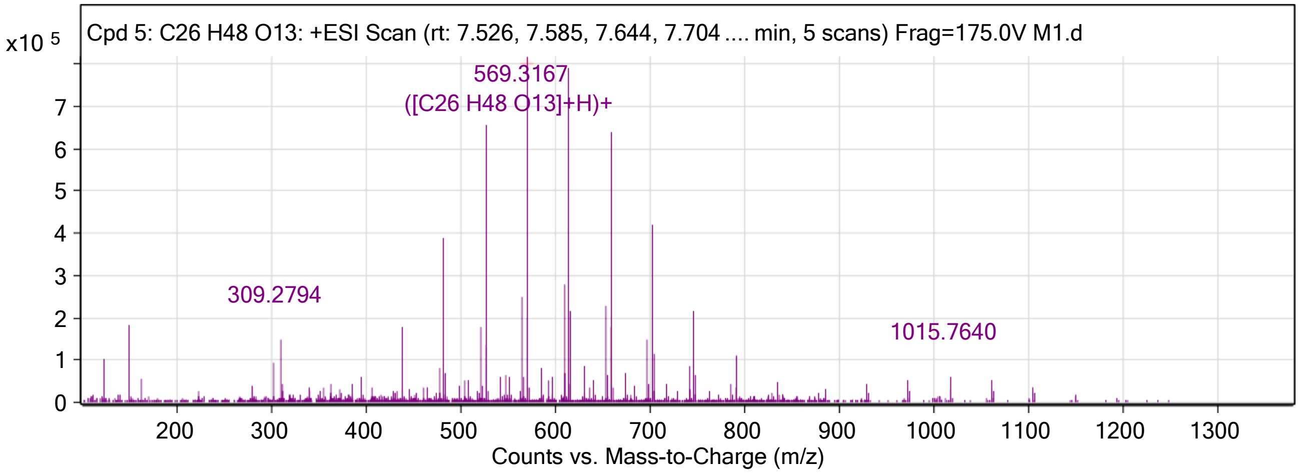

MS Zoomed Spectrum

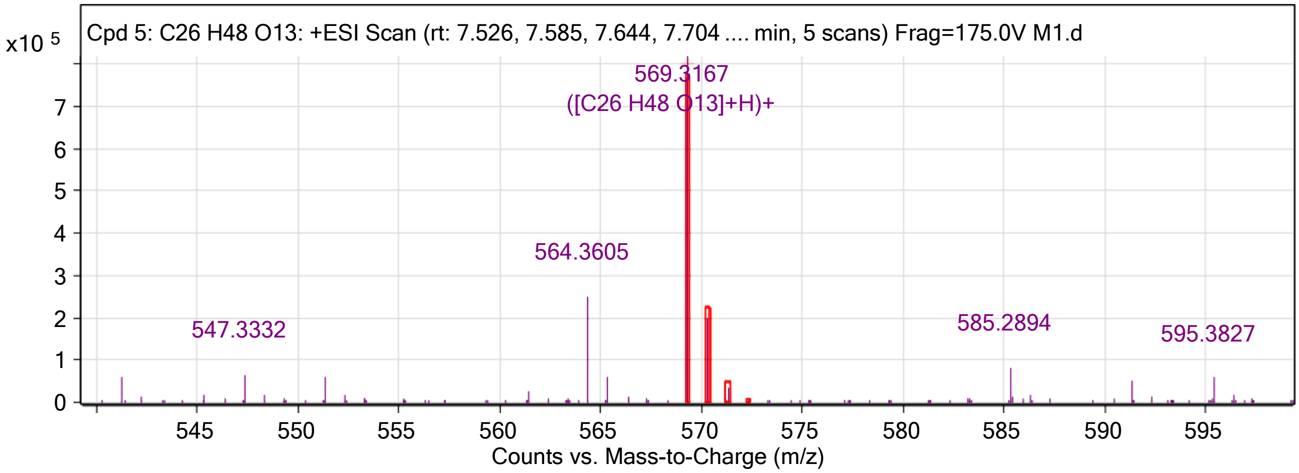

MS Spectrum Peak List

m/z	Calc *m/z*	Diff(ppm)	z	Abund	Formula	Ion
481.2634			1	385549.22		
525.2903			1	652988.13		
569.3167	569.3168	0.08	1	816876.81	C26 H48 O13	(M+H)+
570.3192	570.3202	1.66	1	196281.53	C26 H48 O13	(M+H)+
571.321	571.3226	2.8	1	35324.41	C26 H48 O13	(M+H)+
572.323	572.3253	4.04	1	5107.74	C26 H48 O13	(M+H)+
608.387			1	280152.34		
613.3431			1	786583.56		
657.3692			1	635701.56		
701.3953			1	417346.56		

MSMS Spectrum

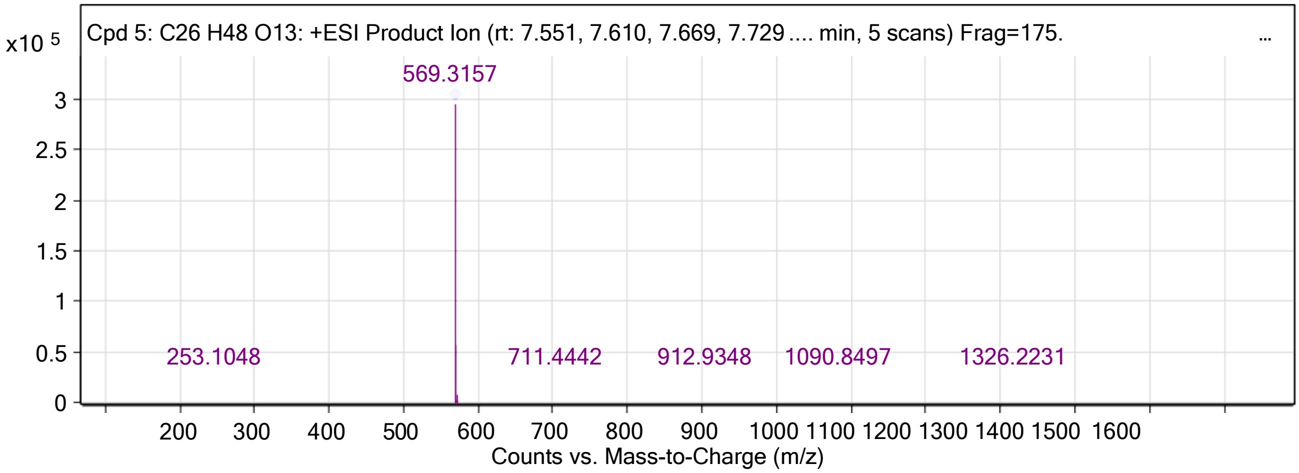

MS/MS Spectrum Peak List

m/z	Calc m/z	Diff(ppm)	z	Abund
123.0415	123.0441	20.59		27.43
124.0827	124.0883	45.17		10.63
170.0876	170.0937	36.09		10.83
171.1006	171.1016	5.64		11.2
233.1736	233.1747	4.84		11.88
255.1536	255.1591	21.57		11.26
309.2776	309.2788	3.91		40.03
318.1972	318.2037	20.44		12.42
350.2605	350.2663	16.49		12.08
569.3157	569.3168	1.89	1	295311.06

Compound Label	m/z	RT	Algorithm	Mass
Cpd 6: C28 H52 O14	613.3431	7.686	Auto MS/MS	612.3356

Compound Chromatograms

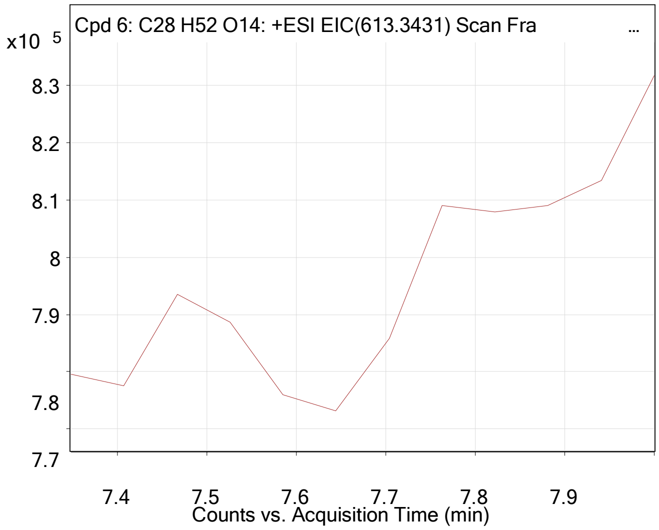

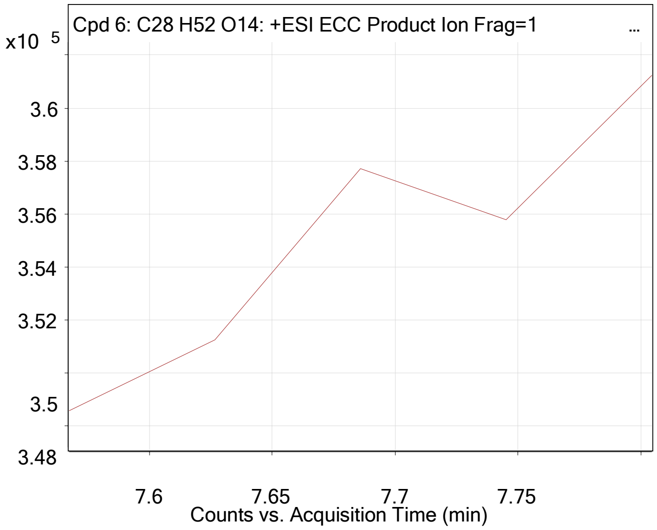

MS Spectrum

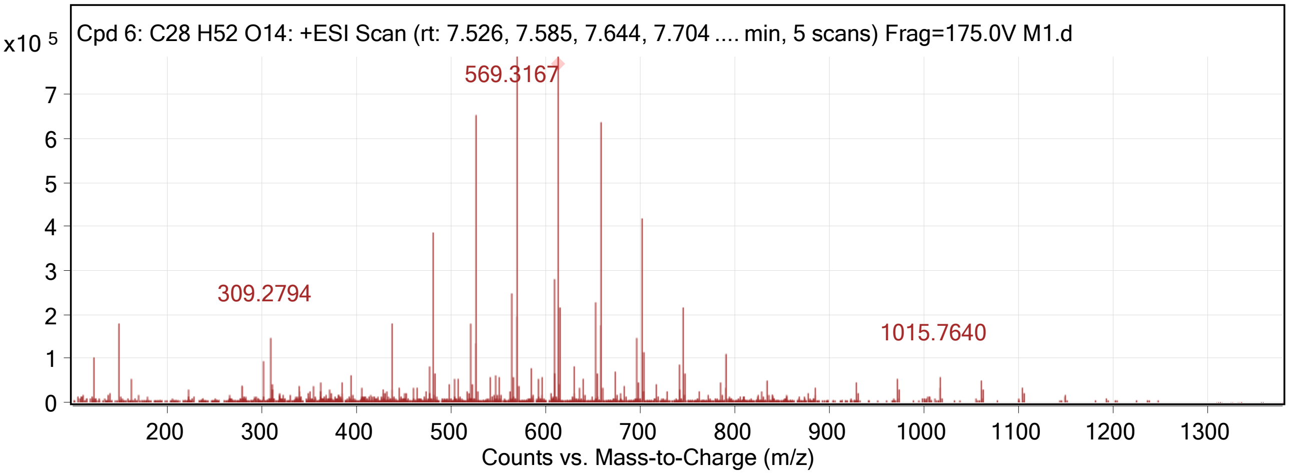

MS Zoomed Spectrum

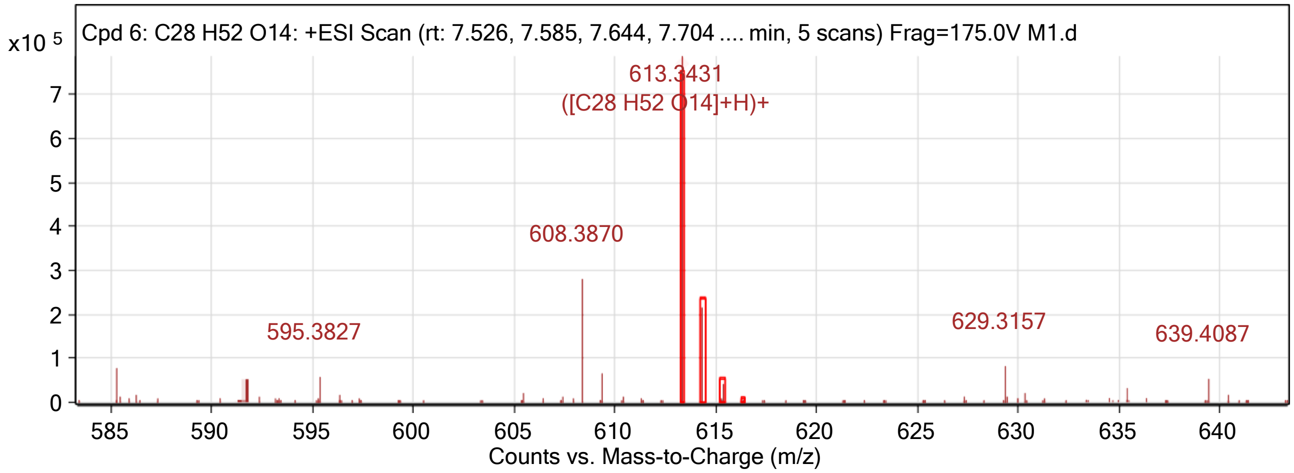

MS Spectrum Peak List

m/z	Calc *m/z*	Diff(ppm)	z	Abund	Formula	Ion
481.2634			1	385549.22		
525.2903			1	652988.13		
569.3167			1	816876.81		
608.387			1	280152.34		
613.3431	613.343	-0.17	1	786583.56	C28 H52 O14	(M+H)+
614.3457	614.3464	1.21	1	216046.84	C28 H52 O14	(M+H)+
615.3474	615.3489	2.31	1	41876.41	C28 H52 O14	(M+H)+
616.35	616.3515	2.55	1	5973.35	C28 H52 O14	(M+H)+
657.3692			1	635701.56		
701.3953			1	417346.56		

MSMS Spectrum

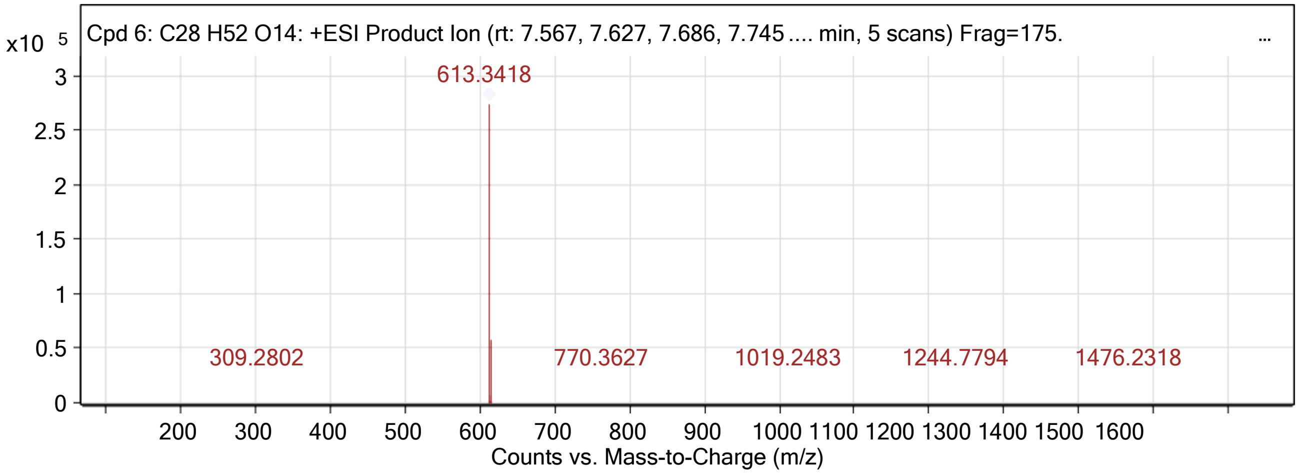

MS/MS Spectrum Peak List

m/z	*Calc m/z*	Diff(ppm)	z	Abund
123.0417	123.0441	19.22	1	42.43
137.0579	137.0597	13.45		22.99
185.1195	185.1172	-12.23		18.4
221.1375	221.1384	3.79		15.86
283.2636	283.2632	-1.49		20.1
298.1724	298.1775	17.17		19.89
309.2802	309.2788	-4.44	1	109.57
613.3418	613.343	1.91	1	273451.75
614.3446			1	57546
615.3465			1	7294.92

Compound Label	*m/z*	RT	Algorithm	Mass
Cpd 7: C24 H46 N3 O12	569.3165	7.966	Auto MS/MS	568.3091

Compound Chromatograms

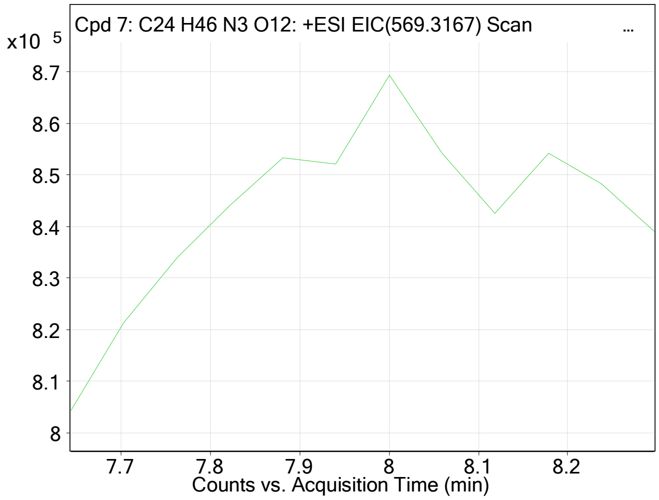

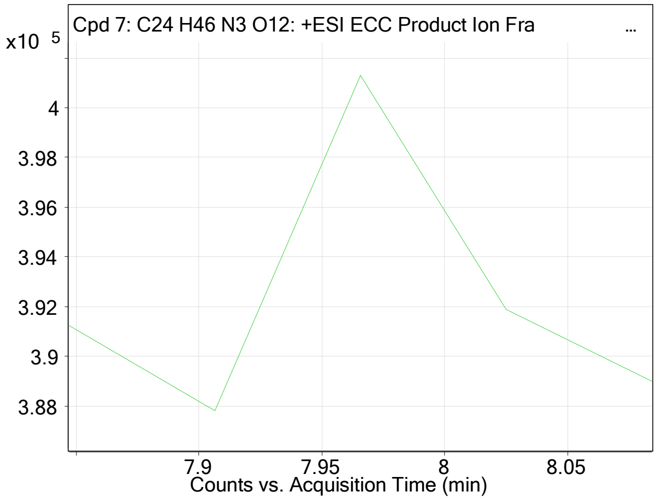

MS Spectrum

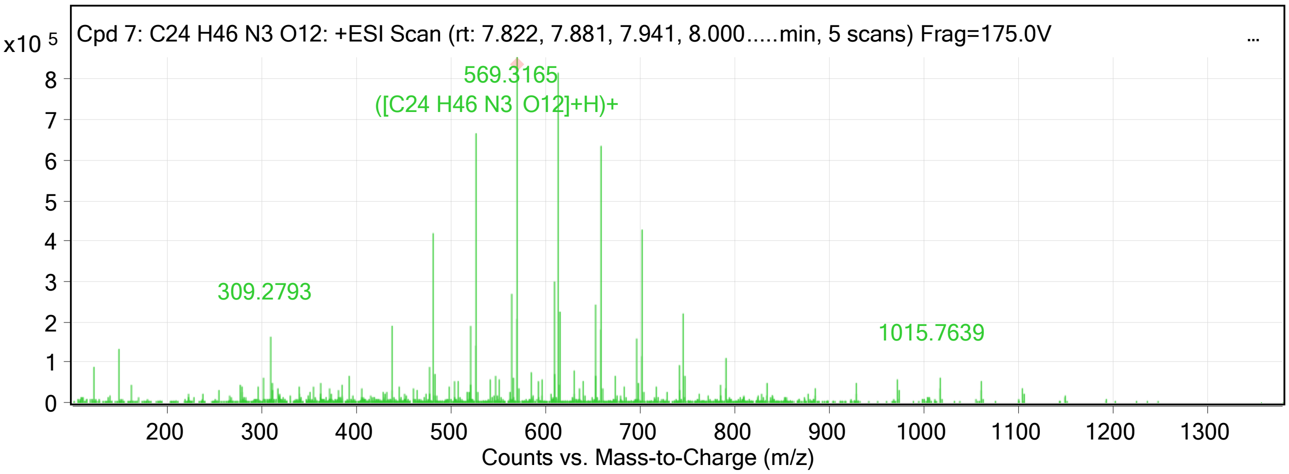

MS Zoomed Spectrum

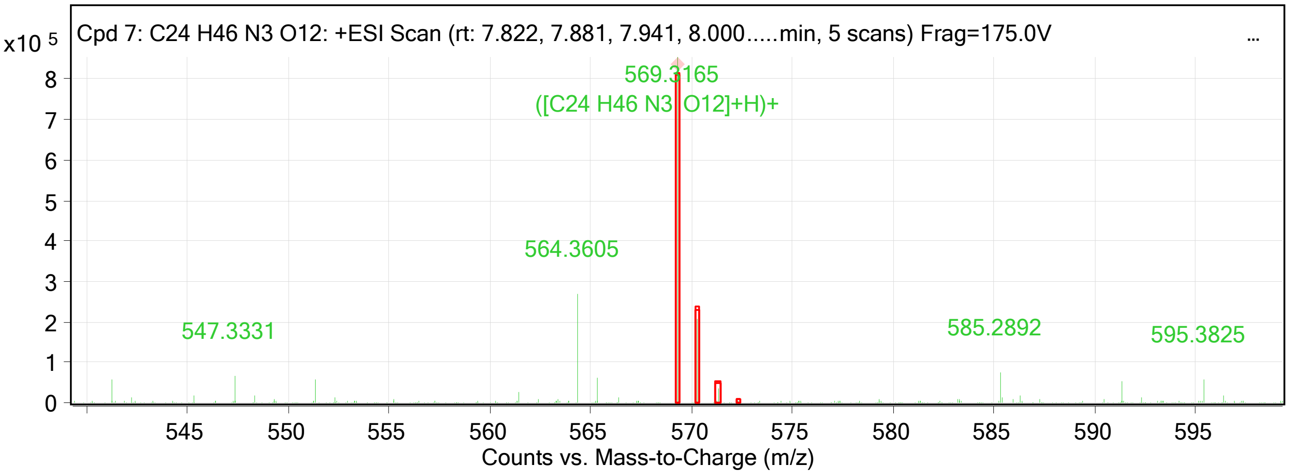

MS Spectrum Peak List

m/z	Calc *m/z*	Diff(ppm)	z	Abund	Formula	Ion
481.2633			1	418216.53		
525.2904			1	666824.44		
569.3165	569.3154	-1.94	1	854662.19	C24 H46 N3 O12	(M+H)+
570.3191	570.3186	-0.85	1	206067.25	C24 H46 N3 O12	(M+H)+
571.3208	571.3209	0.32	1	36367.93	C24 H46 N3 O12	(M+H)+
572.3236	572.3235	-0.21	1	5244.75	C24 H46 N3 O12	(M+H)+
608.3869			1	301534.28		
613.3429			1	815728.44		

MSMS Spectrum

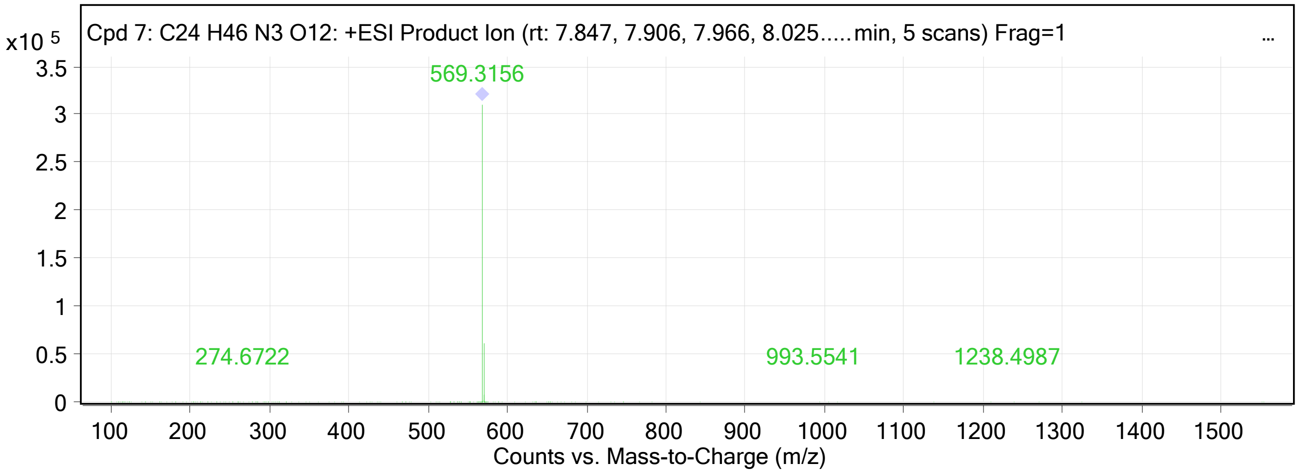

MS/MS Spectrum Peak List

m/z	*Calc m/z*	Diff(ppm)	z	Abund
115.9964	115.9978	12.72		15.36
123.0422	123.0427	4.19		17.8
164.0824	164.0832	4.48		12.47
181.081	181.0819	5.14		28.2
209.0792	209.0768	-11.43		17.38
223.0927	223.0925	-0.86		13.26
309.277	309.2775	1.49		27.67
510.2817	510.2783	-6.61		14.82
551.2882	551.2811	-12.98		29.41
569.3156	569.3154	-0.23	1	310403.88

Compound Label	*m/z*	RT	Algorithm	Mass
Cpd 8: C28 H52 O14	613.3429	7.982	Auto MS/MS	612.3354

Compound Chromatograms

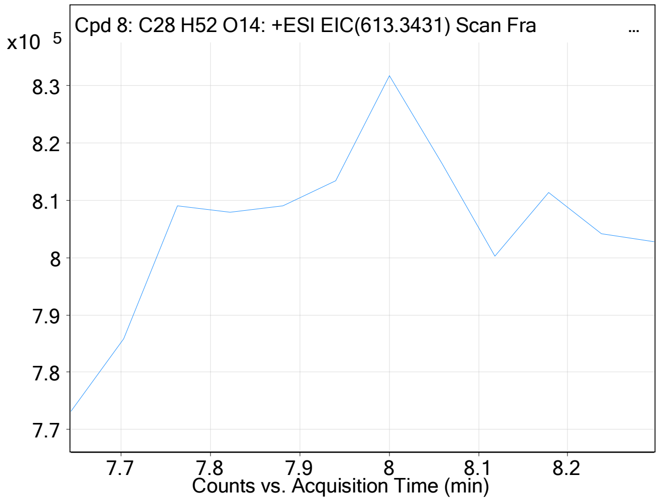

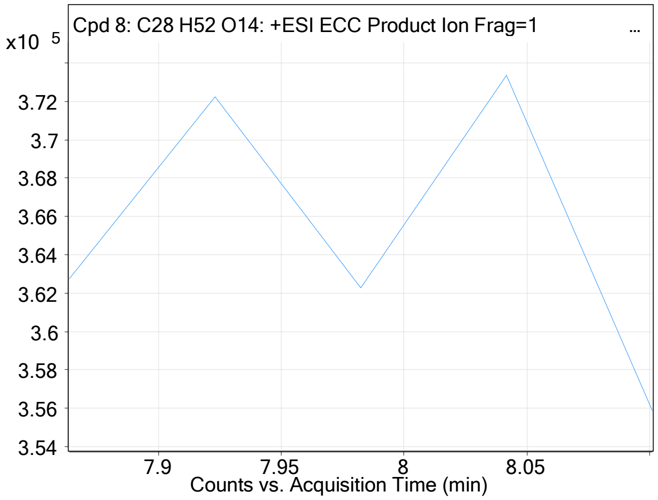

MS Spectrum

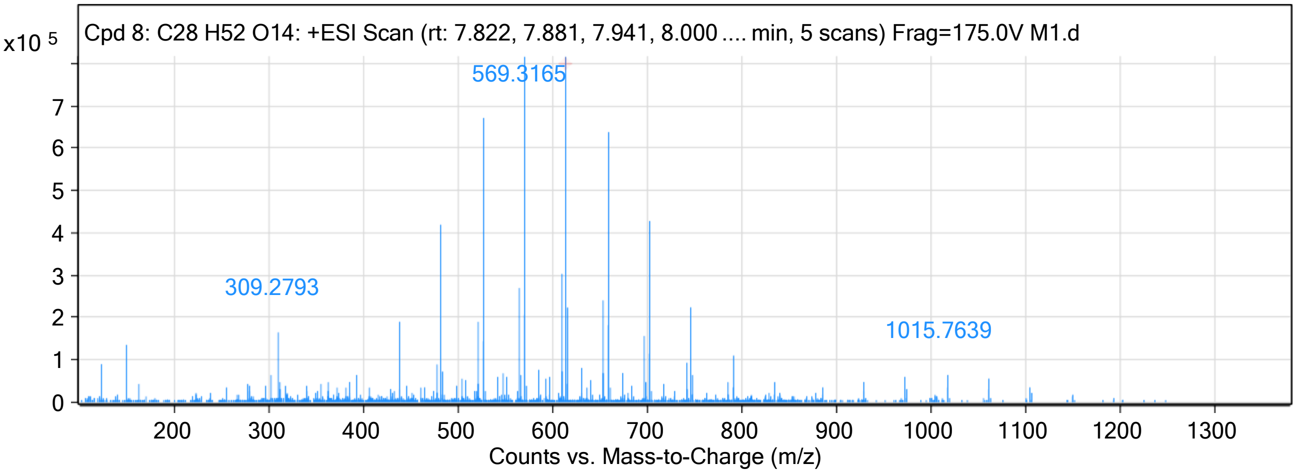

MS Zoomed Spectrum

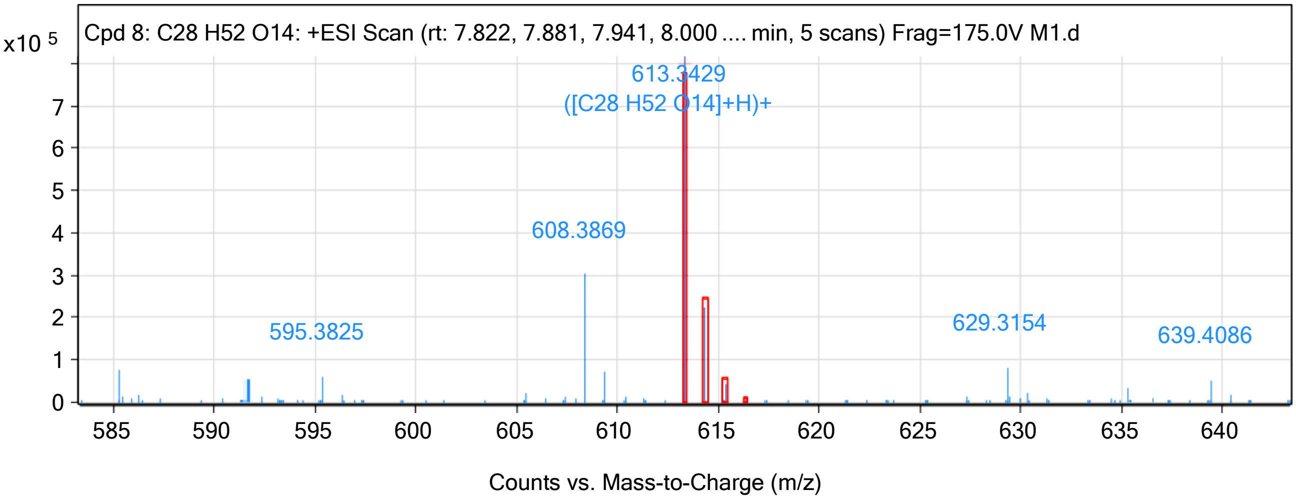

MS Spectrum Peak List

m/z	Calc *m/z*	Diff(ppm)	z	Abund	Formula	Ion
481.2633			1	418216.53		
525.2904			1	666824.44		
569.3165			1	854662.19		
608.3869			1	301534.28		
613.3429	613.343	0.07	1	815728.44	C28 H52 O14	(M+H)+
614.3455	614.3464	1.4	1	223819.8	C28 H52 O14	(M+H)+
615.3474	615.3489	2.35	1	42158.16	C28 H52 O14	(M+H)+
616.3505	616.3515	1.67	1	6091.69	C28 H52 O14	(M+H)+
657.3691			1	633670.44		
701.3951			1	426540.5		

MSMS Spectrum

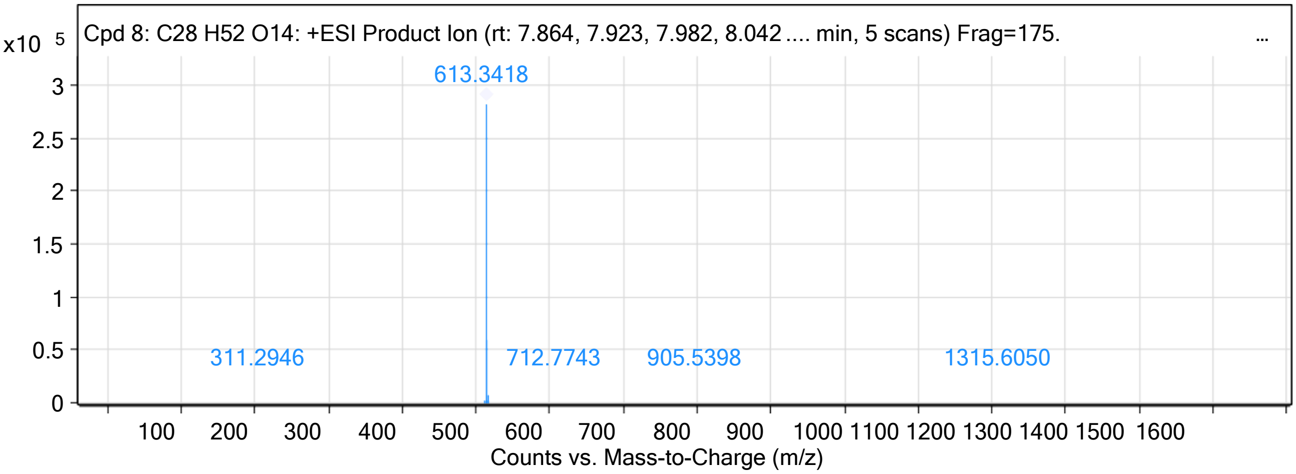

MS/MS Spectrum Peak List

m/z	*Calc m/z*	Diff(ppm)	z	Abund
101.0605	101.0597	-7.94		25.93
123.0417	123.0441	18.89		16.61
133.0861	133.0859	-1		41.7
137.0576	137.0597	15.2		36.92
137.1331	137.1325	-4.29		16.64
169.0869	169.0859	-5.9		16.36
177.1117	177.1121	2.7		32.11
221.1384	221.1384	-0.16		15.9
309.2789	309.2788	-0.27	1	91.47
613.3418	613.343	1.9	1	281919.84

Compound Label	*m/z*	RT	Algorithm	Mass
Cpd 9: C24 H46 N3 O12	569.3166	8.262	Auto MS/MS	568.3091

Compound Chromatograms

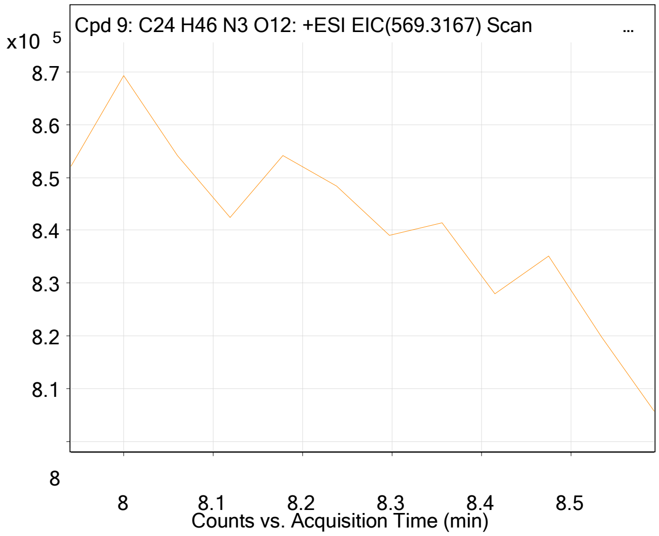

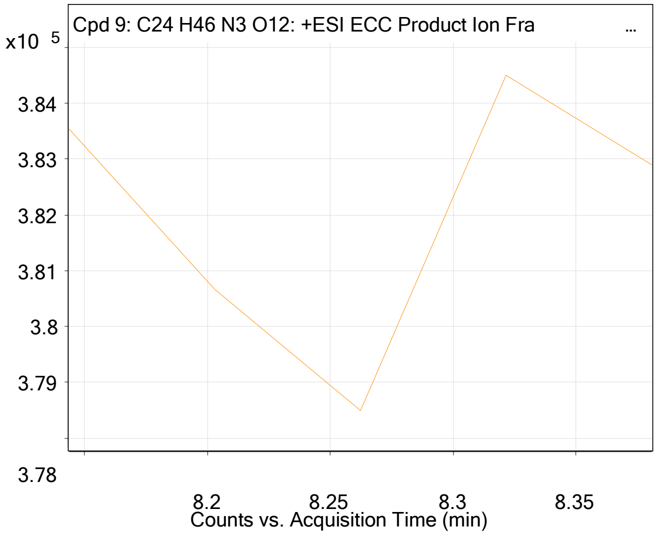

MS Spectrum

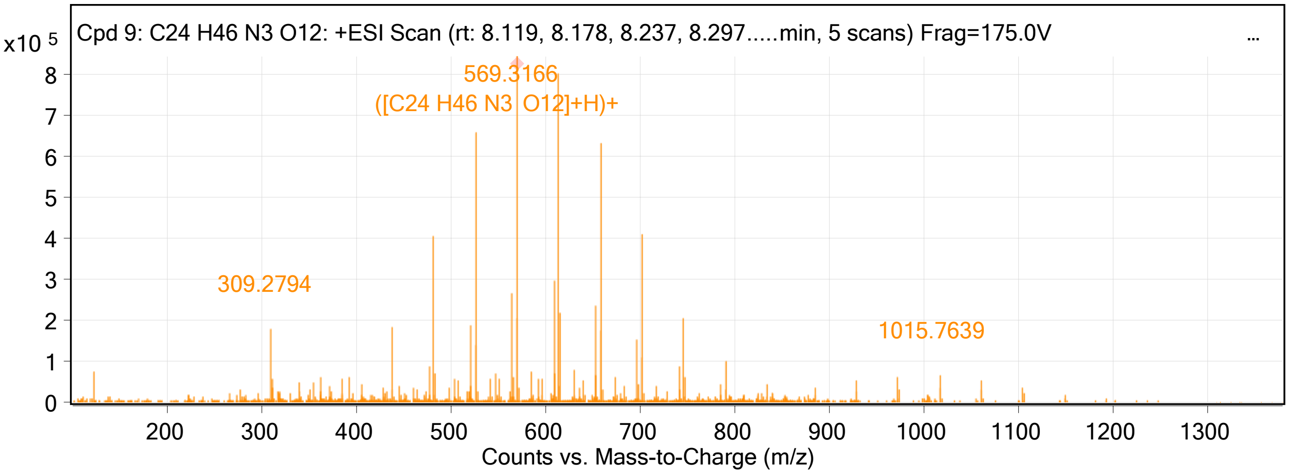

MS Zoomed Spectrum

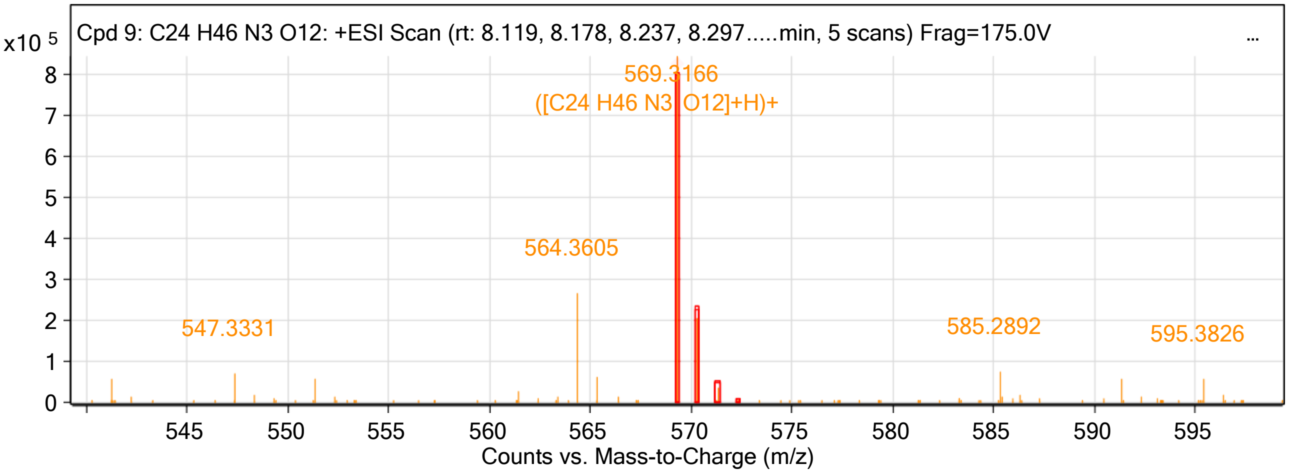

MS Spectrum Peak List

m/z	Calc *m/z*	Diff(ppm)	z	Abund	Formula	Ion
481.2633			1	406037.31		
525.2904			1	660089.81		
569.3166	569.3154	-2.04	1	845153.81	C24 H46 N3 O12	(M+H)+
570.3191	570.3186	-0.91	1	204008.38	C24 H46 N3 O12	(M+H)+
571.3209	571.3209	0.08	1	35704.37	C24 H46 N3 O12	(M+H)+
572.3234	572.3235	0.24	1	5251.58	C24 H46 N3 O12	(M+H)+
608.3869			1	294750.59		
613.343			1	802966.44		
657.3691			1	632231.88		
701.3951			1	410099.91		

MSMS Spectrum

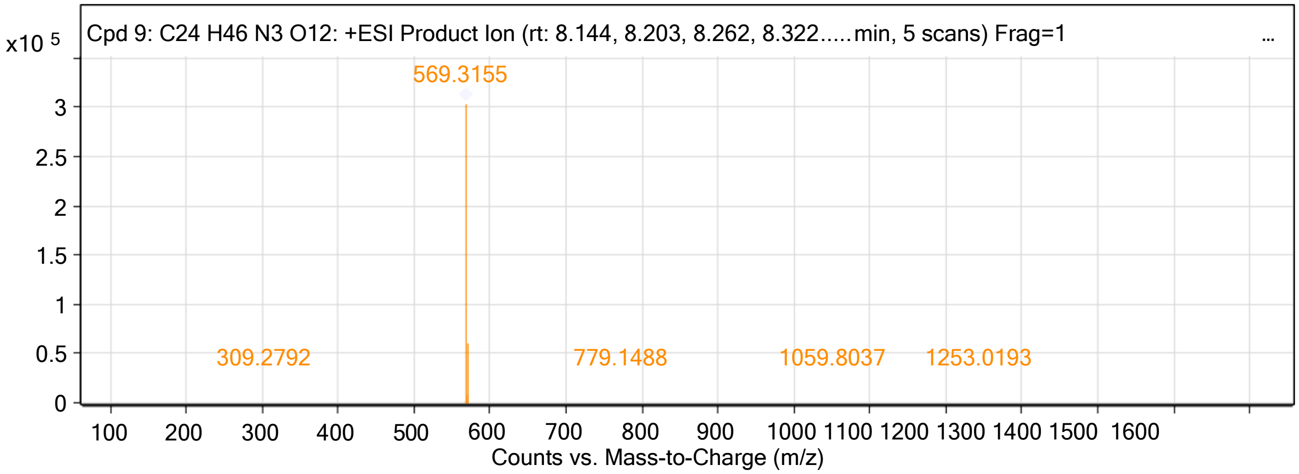

MS/MS Spectrum Peak List

m/z	Calc *m/z*	Diff(ppm)	z	Abund
115.0735	115.074	4.62		16.22
123.0412	123.04	-9.74		25.55
133.0849	133.0846	-2.36		32
195.0978	195.0975	-1.29		16.53
283.2634	283.2632	-0.9		16.22
309.2792	309.2788	-1.19		35.89
311.2934	311.2931	-1.05		17.91
412.2706	412.2694	-2.94		17.19
566.298	566.292	-10.65	1	40.56
569.3155	569.3154	-0.15	1	302640.94

Compound Label	*m/z*	RT	Algorithm	Mass
Cpd 10: C28 H52 O14	613.343	8.279	Auto MS/MS	612.3355

Compound Chromatograms

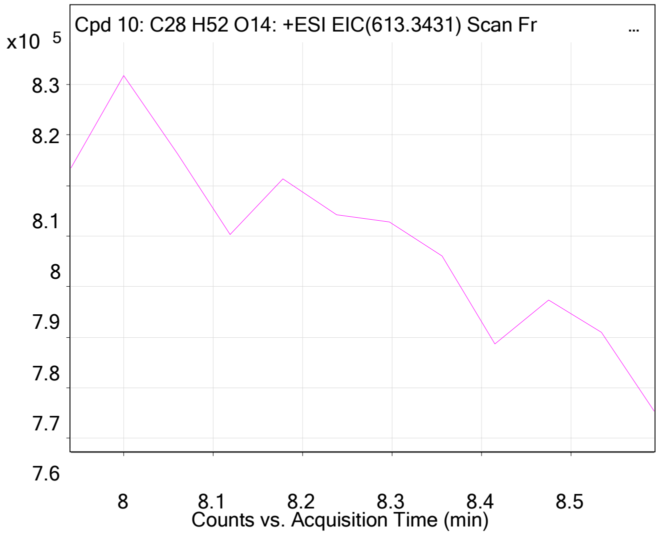

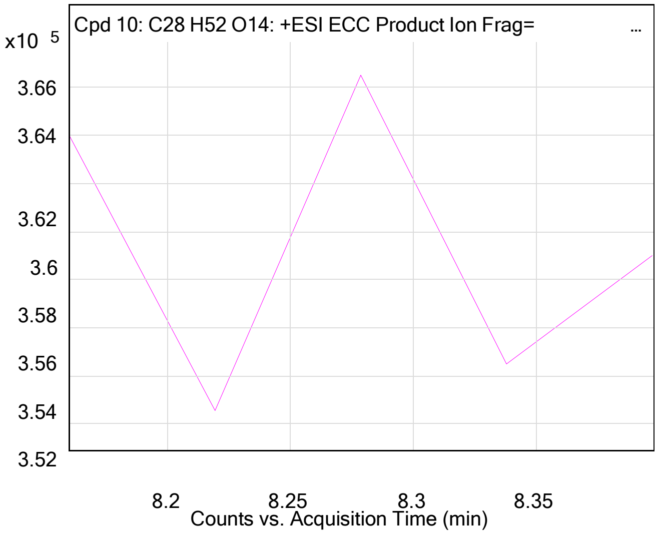

MS Spectrum

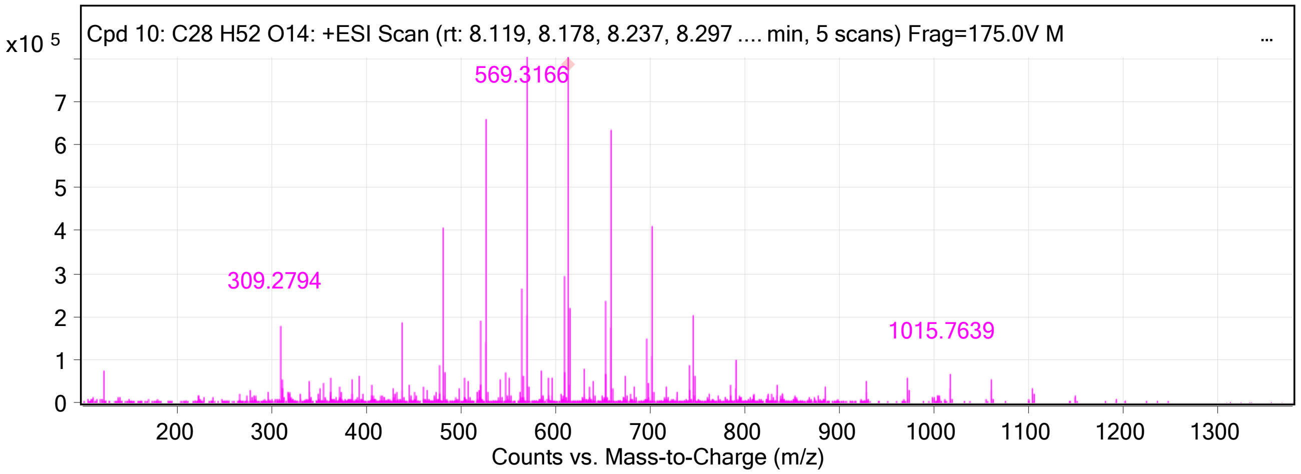

MS Zoomed Spectrum

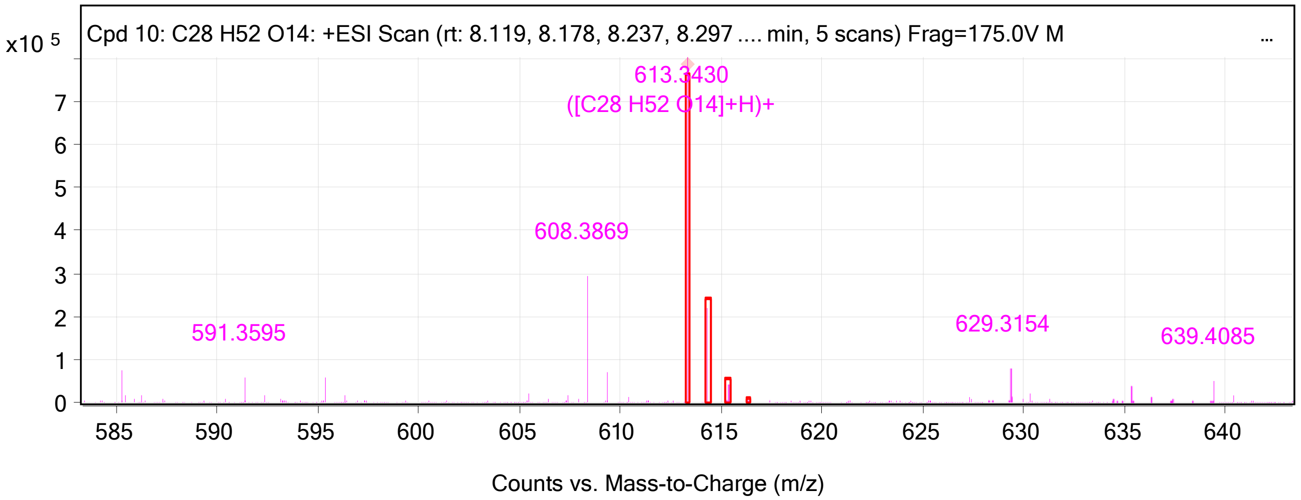

MS Spectrum Peak List

m/z	Calc *m/z*	Diff(ppm)	z	Abund	Formula	Ion
481.2633			1	406037.31		
525.2904			1	660089.81		
569.3166			1	845153.81		
608.3869			1	294750.59		
613.343	613.343	-0.03	1	802966.44	C28 H52 O14	(M+H)+
614.3456	614.3464	1.38	1	219127.25	C28 H52 O14	(M+H)+

MSMS Spectrum

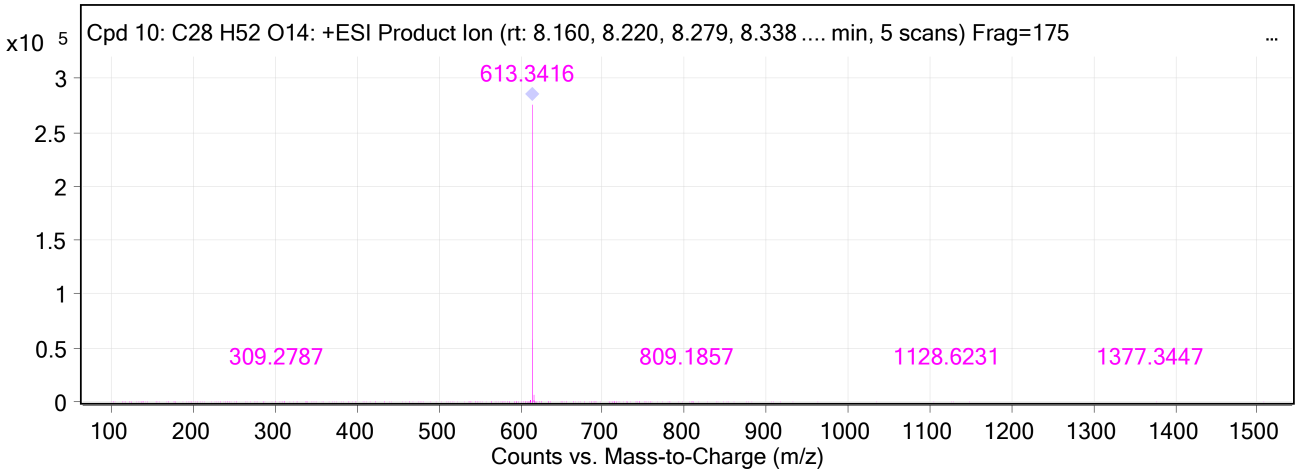

MS/MS Spectrum Peak List

m/z	*Calc m/z*	Diff(ppm)	z	Abund
133.0852	133.0859	5.15		60.94
137.0566	137.0597	22.41		19.82
177.1135	177.1121	-7.78		34.1
199.1273	199.1329	27.8		24.51
210.1158	210.1098	-28.58		24.86
309.2787	309.2788	0.41	1	146.24
611.3256	305.6634	-499999.04	2	33.3
613.3416	613.343	2.2	1	276227.25
614.3444			1	57639.21
615.3466			1	7255.1

Compound Label	*m/z*	RT	Algorithm	Mass
Cpd 11: C24 H46 N3 O12	569.3165	8.559	Auto MS/MS	568.309

Compound Chromatograms

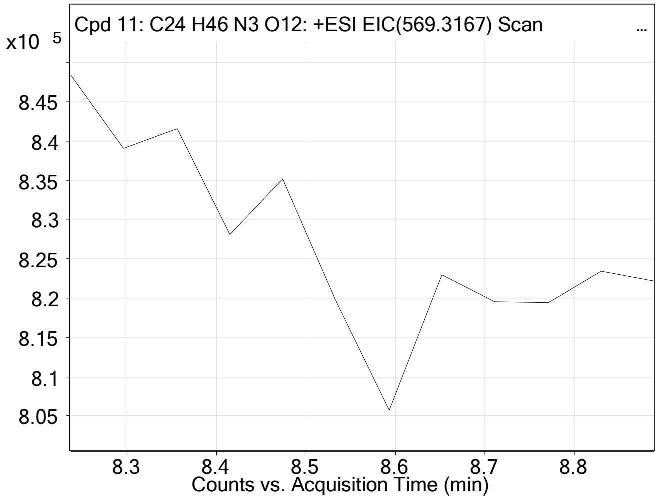

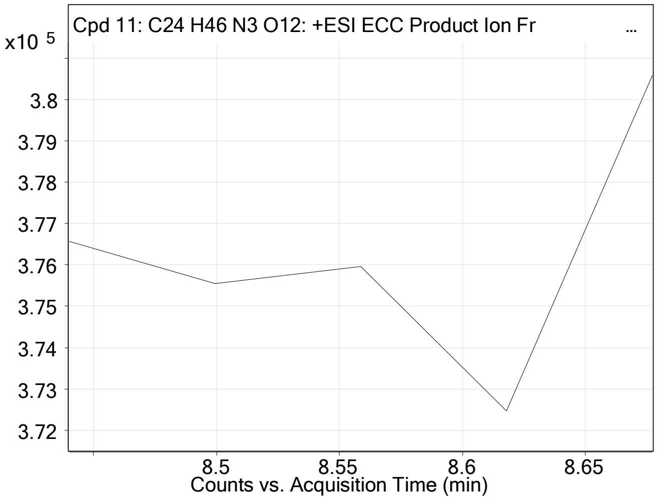

MS Spectrum

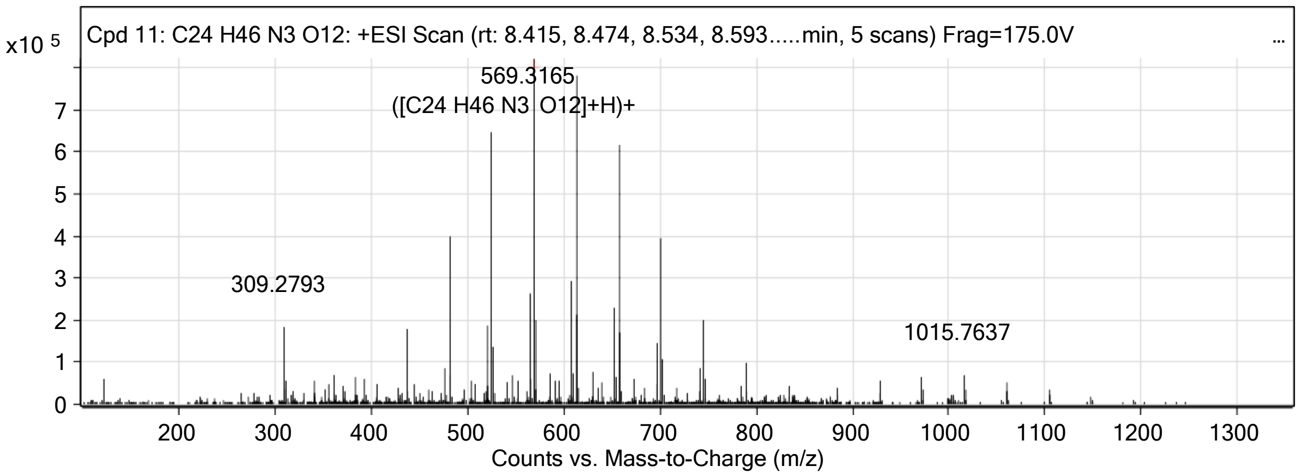

MS Zoomed Spectrum

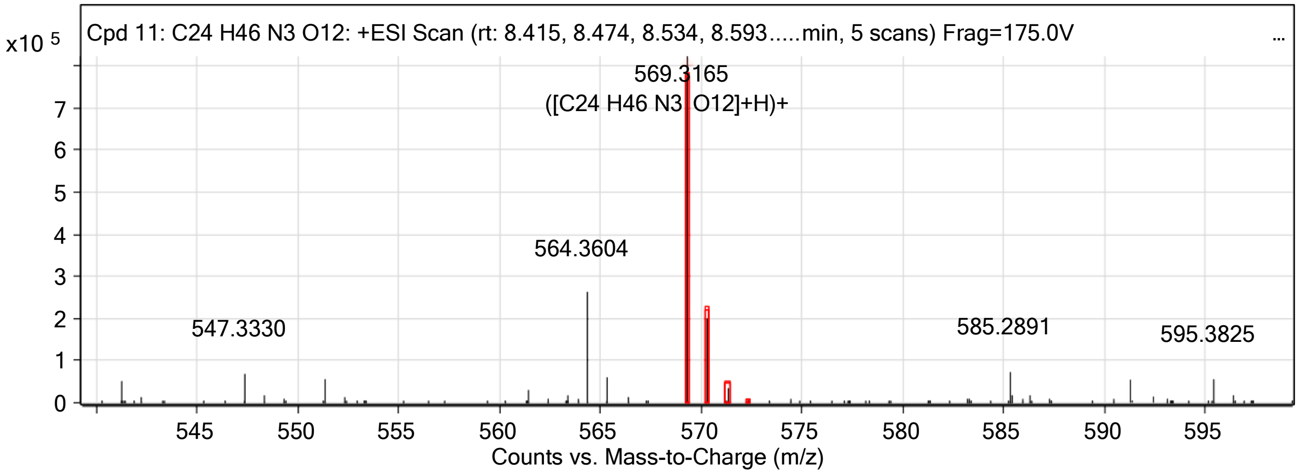

MS Spectrum Peak List

m/z	Calc *m/z*	Diff(ppm)	z	Abund	Formula	Ion
481.2631			1	400037.78		
525.2903			1	644123.63		
569.3165	569.3154	-1.81	1	822353.81	C24 H46 N3 O12	(M+H)+
570.319	570.3186	-0.62	1	198045.33	C24 H46 N3 O12	(M+H)+
571.3208	571.3209	0.31	1	34914.15	C24 H46 N3 O12	(M+H)+
572.3236	572.3235	-0.18	1	4897.41	C24 H46 N3 O12	(M+H)+
608.3868			1	291705.75		
613.3428			1	778573.56		
657.3689			1	615572.5		
701.3949			1	393962.06		

MSMS Spectrum

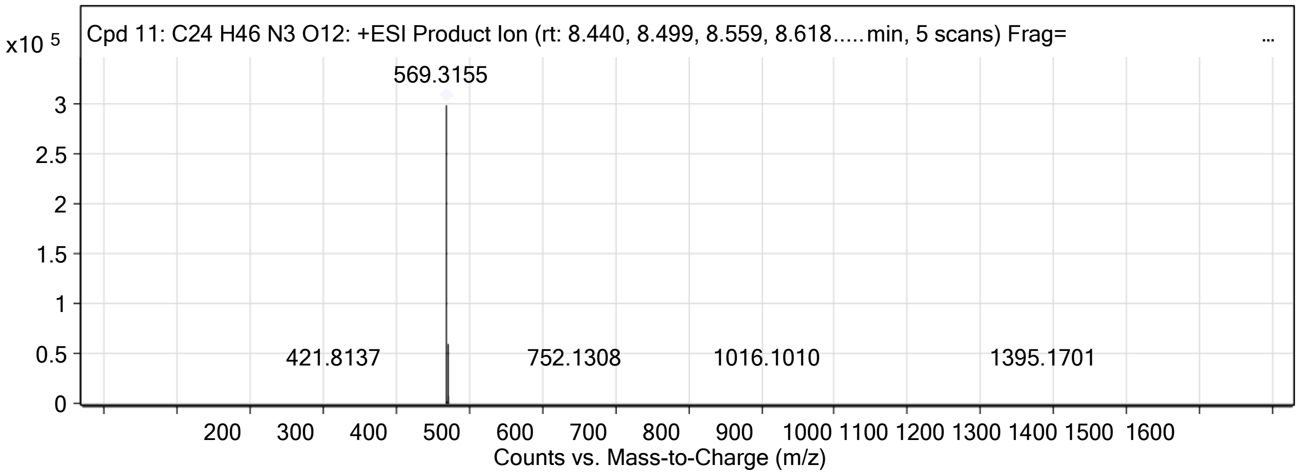

MS/MS Spectrum Peak List

m/z	*Calc m/z*	Diff(ppm)	z	Abund
113.0607	113.0597	-9.2		14.67
133.0861	133.0859	-1.07		25.69
157.122	157.1223	2.13		19.37
177.1123	177.1121	-1.15		29.62
253.1009	253.103	8.35		16.01
311.2946	311.2945	-0.49		30.64
312.2966	312.3009	13.86		21.93
431.2857	431.2878	4.68		13.67
566.2916	283.1457	-500000.2	2	46.44
569.3155	569.3154	-0.16	1	298496.94

Compound Label	*m/z*	RT	Algorithm	Mass
Cpd 12: C26 H50 N3 O13	613.3428	8.575	Auto MS/MS	612.3354

Compound Chromatograms

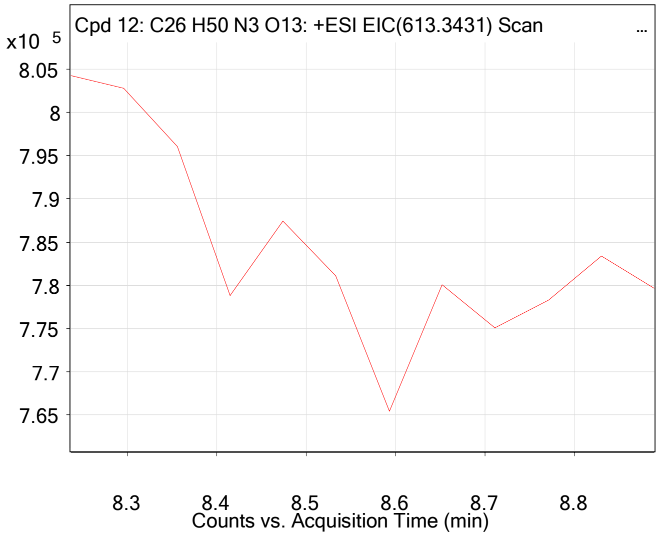

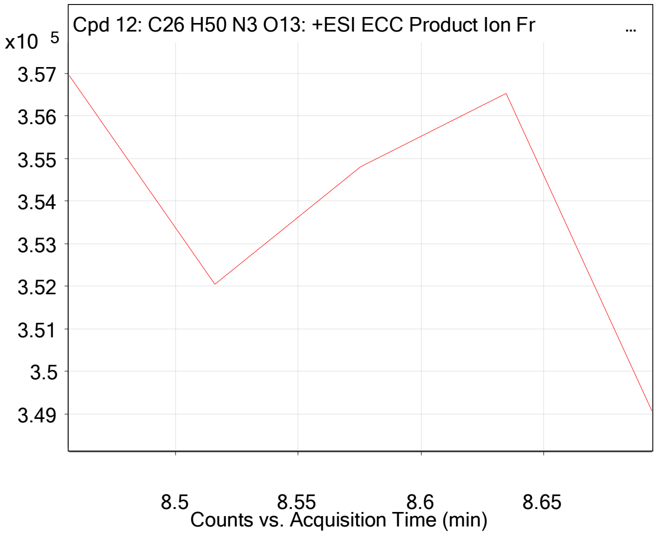

MS Spectrum

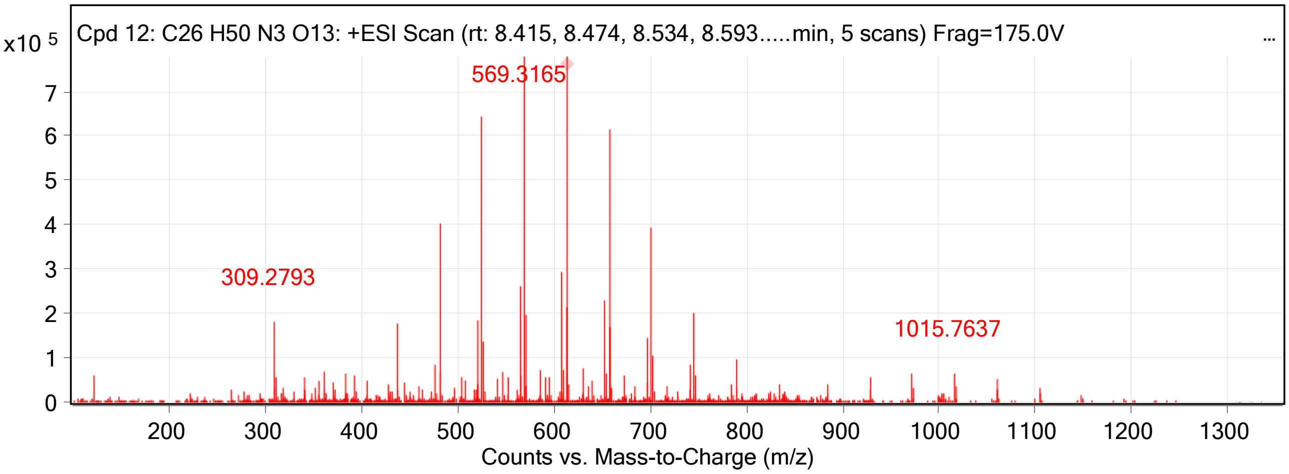

MS Zoomed Spectrum

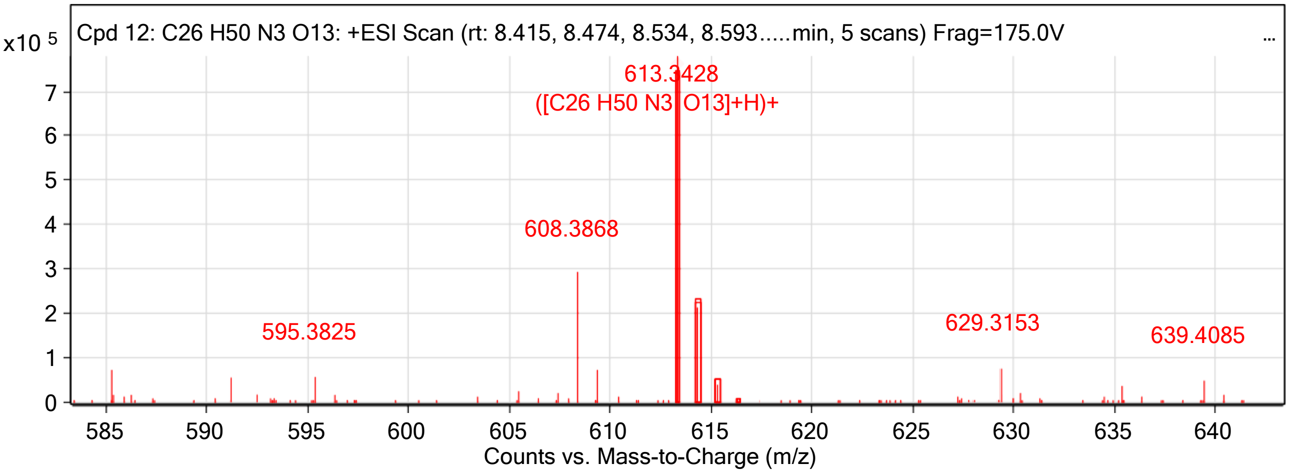

MS Spectrum Peak List

m/z	Calc *m/z*	Diff(ppm)	z	Abund	Formula	Ion
481.2631			1	400037.78		
525.2903			1	644123.63		
569.3165			1	822353.81		
608.3868			1	291705.75		
613.3428	613.3416	-1.89	1	778573.56	C26 H50 N3 O13	(M+H)+
614.3454	614.3448	-0.86	1	211248.28	C26 H50 N3 O13	(M+H)+
615.3474	615.3472	-0.22	1	39722.16	C26 H50 N3 O13	(M+H)+
616.3508	616.3498	-1.58	1	5955.18	C26 H50 N3 O13	(M+H)+
657.3689			1	615572.5		
701.3949			1	393962.06		

MSMS Spectrum

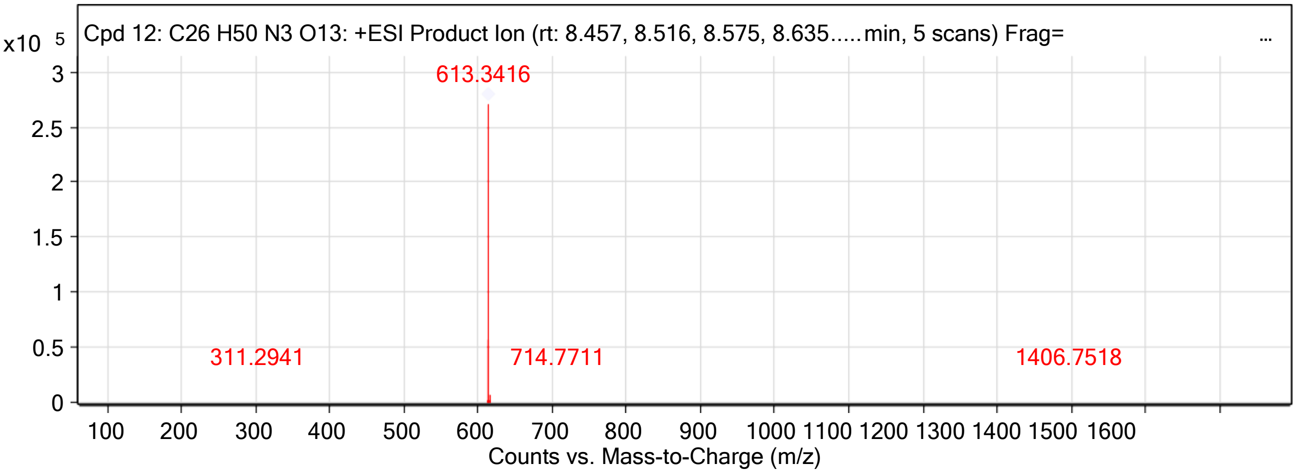

MS/MS Spectrum Peak List

m/z	*Calc m/z*	Diff(ppm)	z	Abund
123.0424	123.0427	2.8		27
133.0855	133.0859	3.34	1	48.43
137.0578	137.0584	4.03	1	38.53
195.1223	195.1214	-5.06		30.62
309.2787	309.2788	0.32	1	122
371.2273	371.2262	-2.99		26.3
612.3861			2	1597.39
613.3416	613.3416	0.14	1	270512.41
614.3443			1	56847.11
615.3464			1	7178.2

Compound Label	*m/z*	RT	Algorithm	Mass
Cpd 13: C24 H46 N3 O12	569.3162	8.855	Auto MS/MS	568.3088

Compound Chromatograms

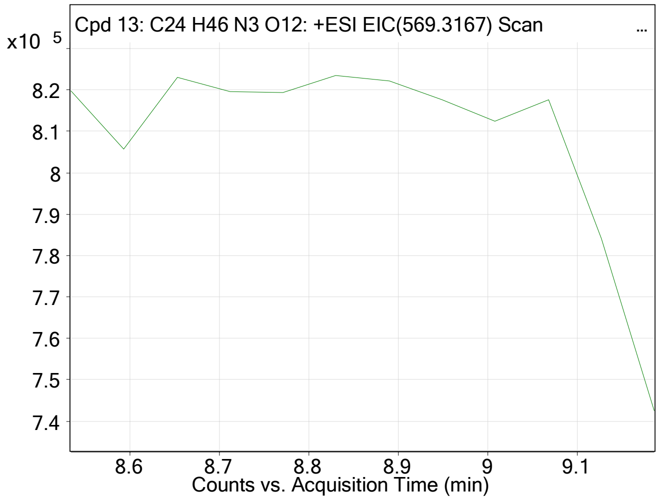

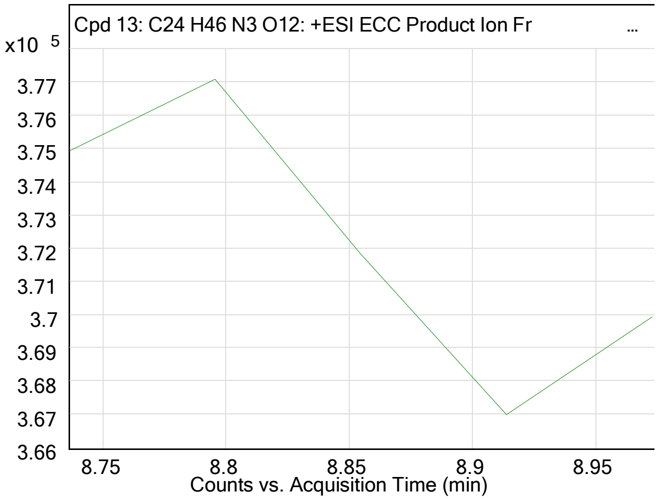

MS Spectrum

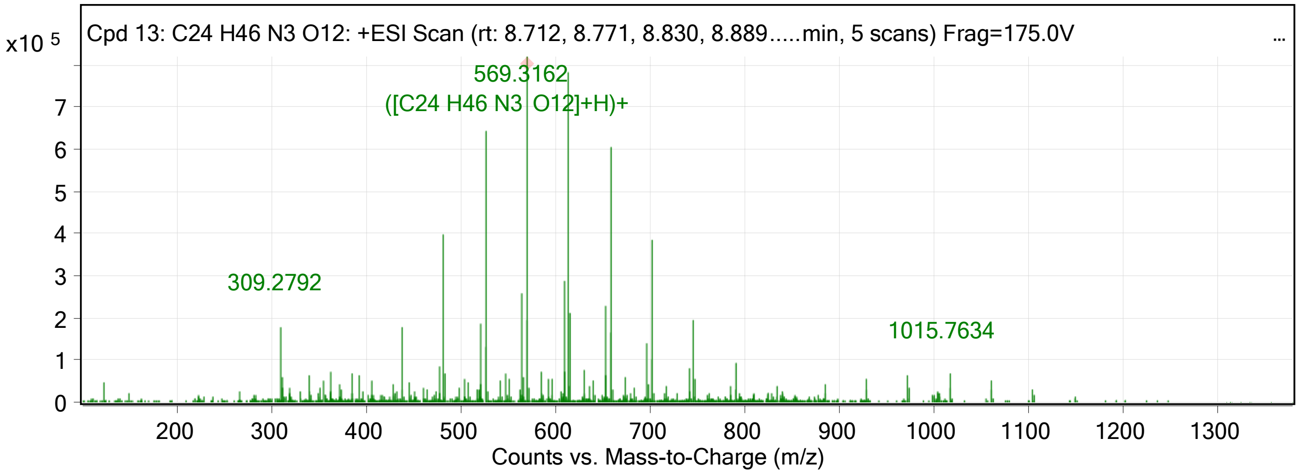

MS Zoomed Spectrum

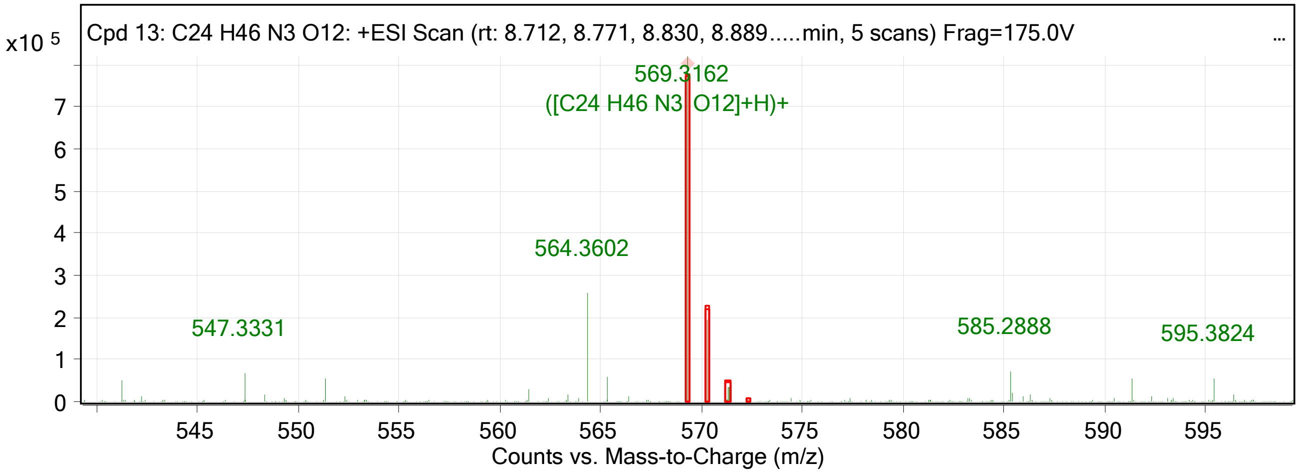

MS Spectrum Peak List

m/z	Calc *m/z*	Diff(ppm)	z	Abund	Formula	Ion
481.263			1	398211.56		
525.2901			1	643794.63		
569.3162	569.3154	-1.39	1	820458	C24 H46 N3 O12	(M+H)+
570.3188	570.3186	-0.38	1	195932.06	C24 H46 N3 O12	(M+H)+
571.3206	571.3209	0.57	1	33755.82	C24 H46 N3 O12	(M+H)+
572.3231	572.3235	0.71	1	5042.35	C24 H46 N3 O12	(M+H)+
608.3866			1	287926.59		
613.3426			1	780660.56		
657.3686			1	603059.38		

MSMS Spectrum

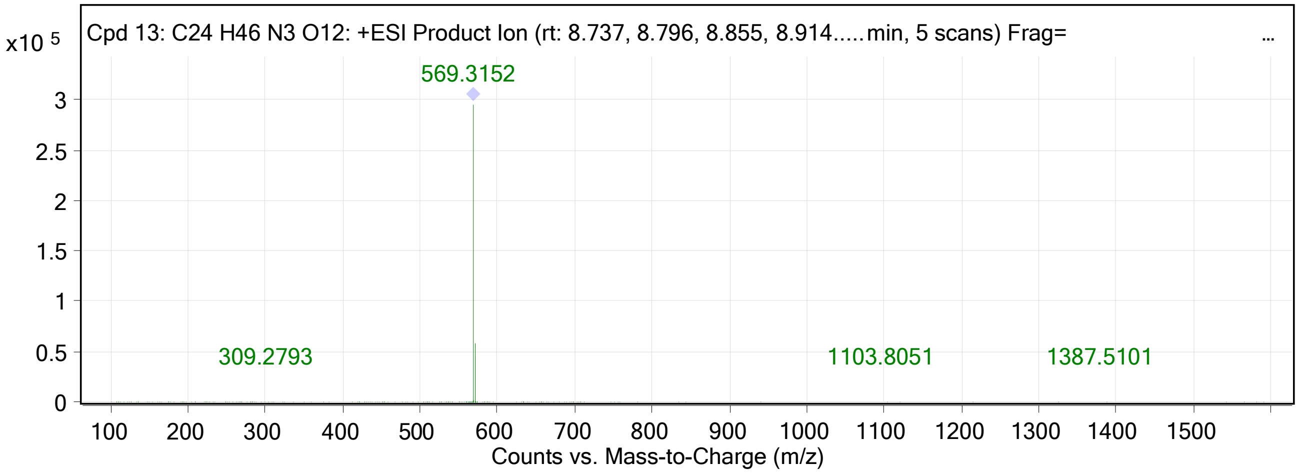

MS/MS Spectrum Peak List

m/z	*Calc m/z*	Diff(ppm)	z	Abund
101.0607	101.0597	-9.4		18.82
117.0909	117.091	1.14		15.02
121.0606	121.0608	1.14		16.07
123.0779	123.0791	9.77		12.28
209.077	209.0768	-0.82		18.45
265.1639	265.1646	2.35		17.92
309.2793	309.2788	-1.59		56.67
311.2943	311.2945	0.35		32.37
568.2999	568.3076	13.56	1	191.37
569.3152	569.3154	0.34	1	295479.19

Compound Label	*m/z*	RT	Algorithm	Mass
Cpd 14: C26 H50 N3 O13	613.3426	8.872	Auto MS/MS	612.3351

Compound Chromatograms

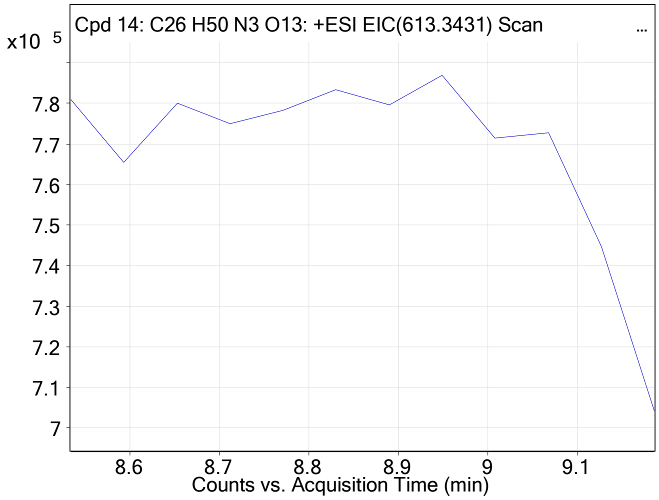

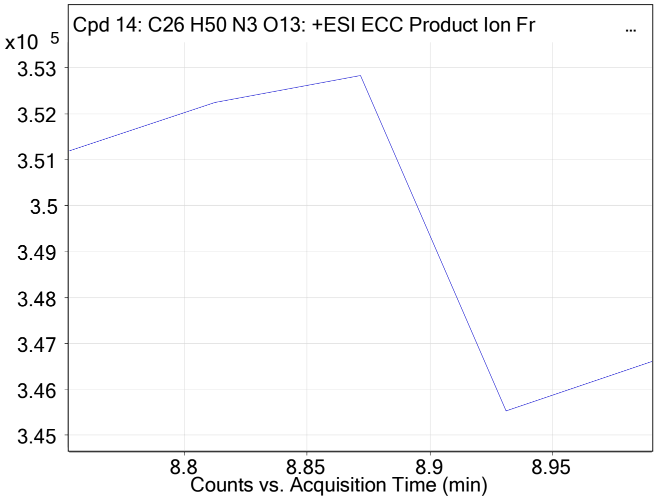

MS Spectrum

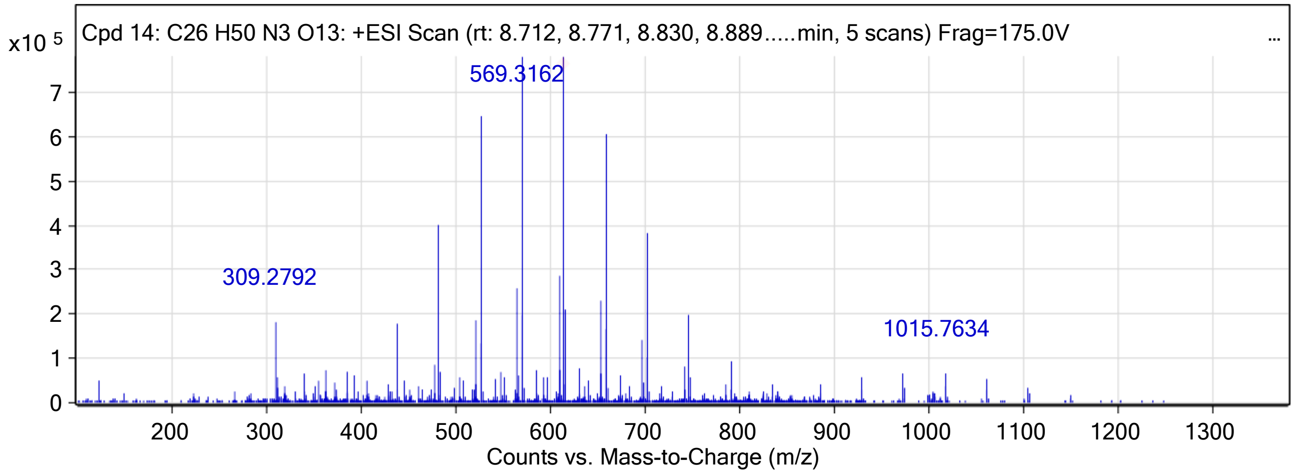

MS Zoomed Spectrum

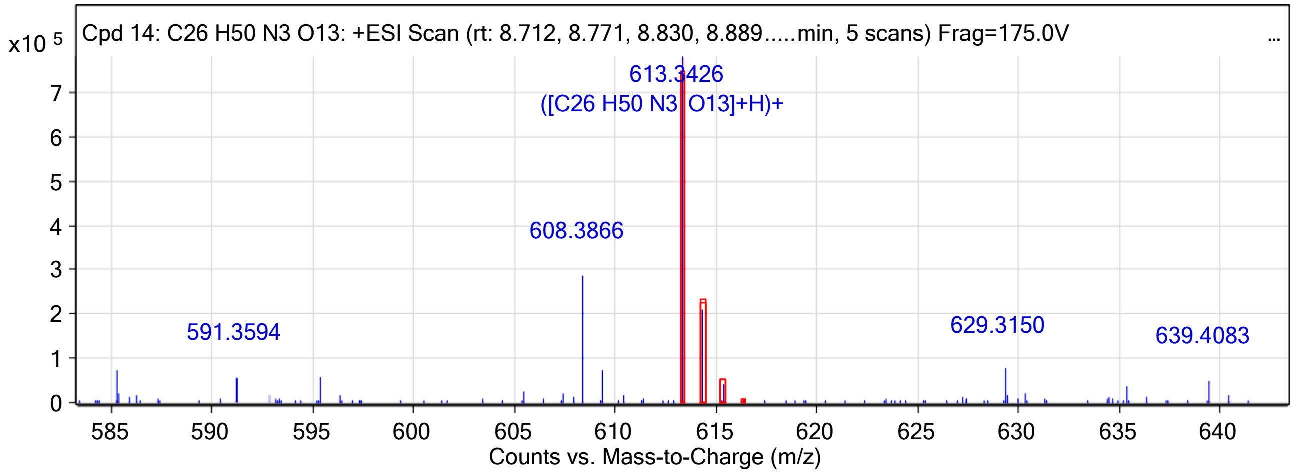

MS Spectrum Peak List

m/z	Calc *m/z*	Diff(ppm)	z	Abund	Formula	Ion
481.263			1	398211.56		
525.2901			1	643794.63		
569.3162			1	820458		
608.3866			1	287926.59		
613.3426	613.3416	-1.53	1	780660.56	C26 H50 N3 O13	(M+H)+
614.3452	614.3448	-0.64	1	210532.34	C26 H50 N3 O13	(M+H)+
615.3471	615.3472	0.25	1	39098.23	C26 H50 N3 O13	(M+H)+
616.3507	616.3498	-1.42	1	5449.26	C26 H50 N3 O13	(M+H)+
657.3686			1	603059.38		
701.3947			1	384189.75		

MSMS Spectrum

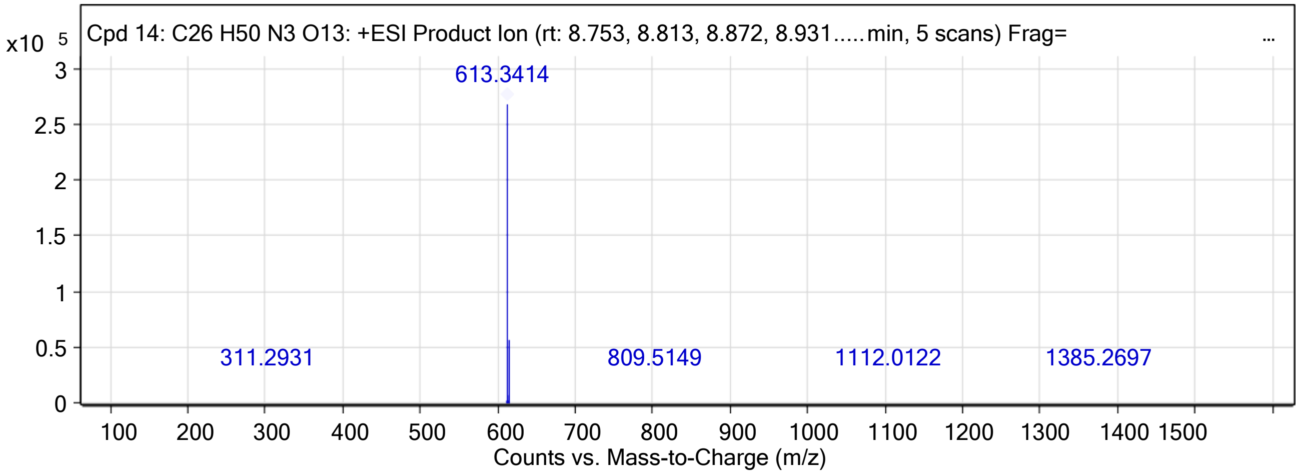

MS/MS Spectrum Peak List

m/z	*Calc m/z*	Diff(ppm)	z	Abund
133.0852	133.0846	-4.96		39.14
134.089	134.0924	25.15		20.5
144.0754	144.0768	9.24		29.95
223.0637	223.0687	22.36		19.75
239.1459	239.1476	6.98		22.58
309.2799	309.2788	-3.47	1	125.06
377.2136	377.2157	5.52		20.39
613.3414	613.3416	0.44	1	267720.41
614.3441			1	55749.68
615.3463			1	7028.53

Compound Label	*m/z*	RT	Algorithm	Mass
Cpd 15: C26 H48 O13	569.3167	9.152	Auto MS/MS	568.3092

Compound Chromatograms

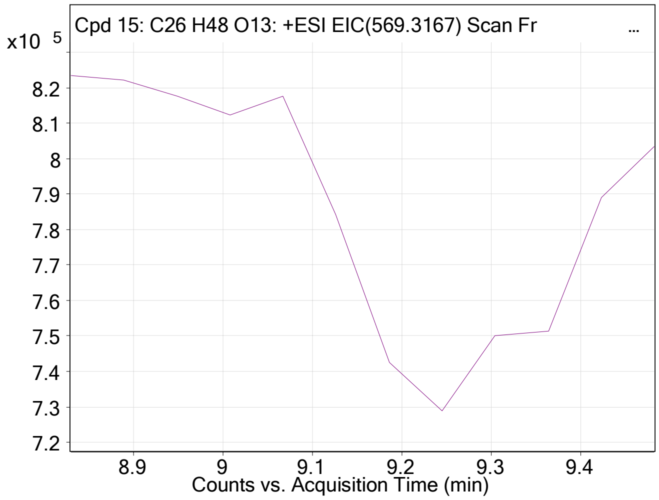

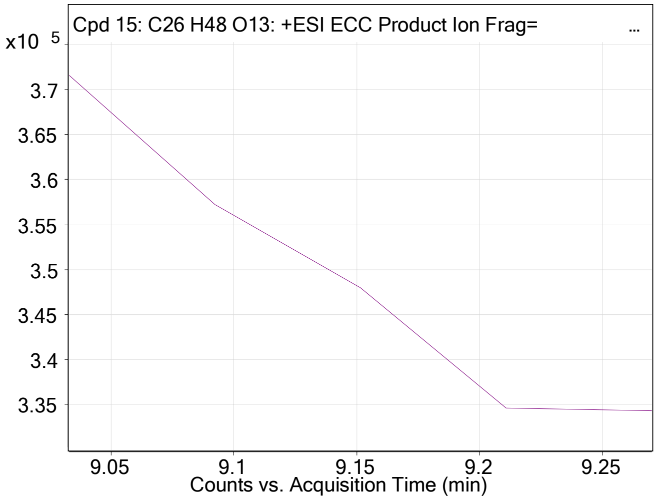

MS Spectrum

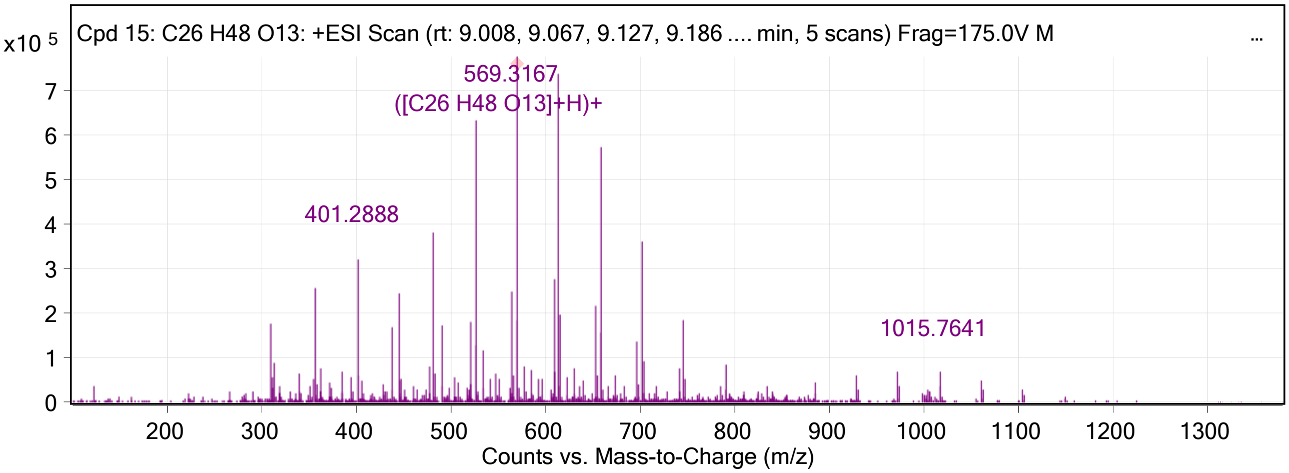

MS Zoomed Spectrum

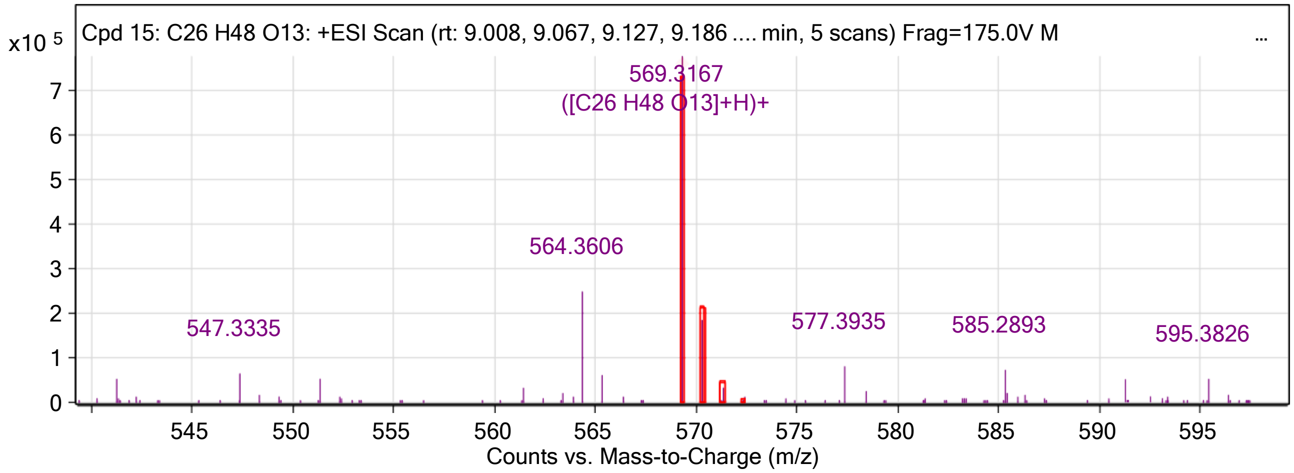

MS Spectrum Peak List

m/z	Calc *m/z*	Diff(ppm)	z	Abund	Formula	Ion
401.2888			1	322013		
481.2634			1	380988		
525.2902			1	633922.19		
569.3167	569.3168	0.11	1	777143.5	C26 H48 O13	(M+H)+
570.3192	570.3202	1.71	1	186423.81	C26 H48 O13	(M+H)+
571.3212	571.3226	2.39	1	32550.71	C26 H48 O13	(M+H)+
572.3239	572.3253	2.38	1	4694.65	C26 H48 O13	(M+H)+
613.3432			1	737040.31		
657.3692			1	572170.13		
701.3953			1	359118		

MSMS Spectrum

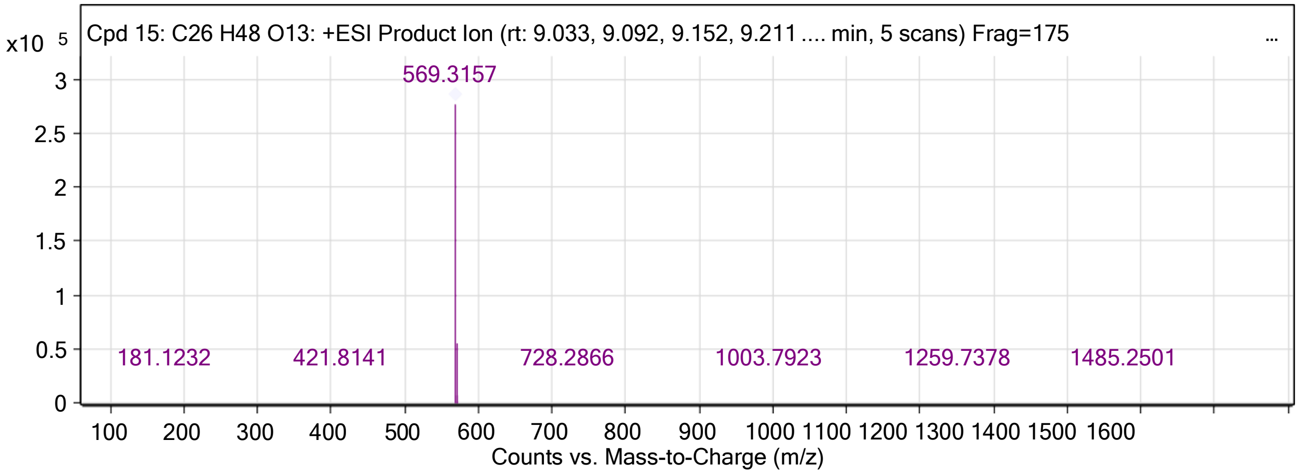

MS/MS Spectrum Peak List

m/z	Calc *m/z*	Diff(ppm)	z	Abund
133.087	133.0859	-8.2		18.81
137.0542	137.0597	40.01		15.15
175.133	175.1329	-0.78		18.83
181.1232	181.1223	-4.82		20.84
283.2637	283.2632	-1.79	1	44.42
309.277	309.2788	5.86		29.05
310.2857	310.2866	3.02	1	18.47
566.3013	283.1464	-500007.59	2	22.42
568.3026	284.1542	-499994.88	2	232.65
569.3157	569.3168	1.91	1	276593.31

Compound Label	*m/z*	RT	Algorithm	Mass
Cpd 16: C24 H46 N3 O12	569.3161	9.448	Auto MS/MS	568.3087

Compound Chromatograms

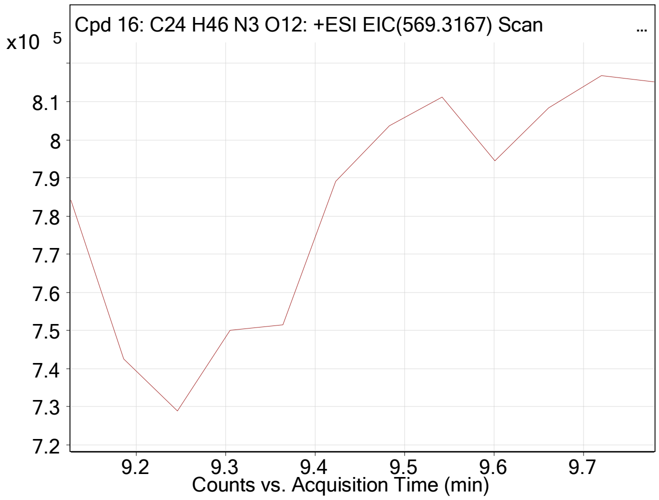

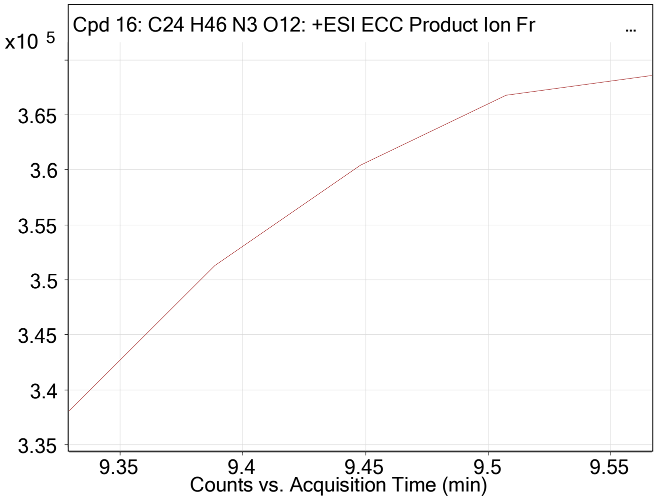

MS Spectrum

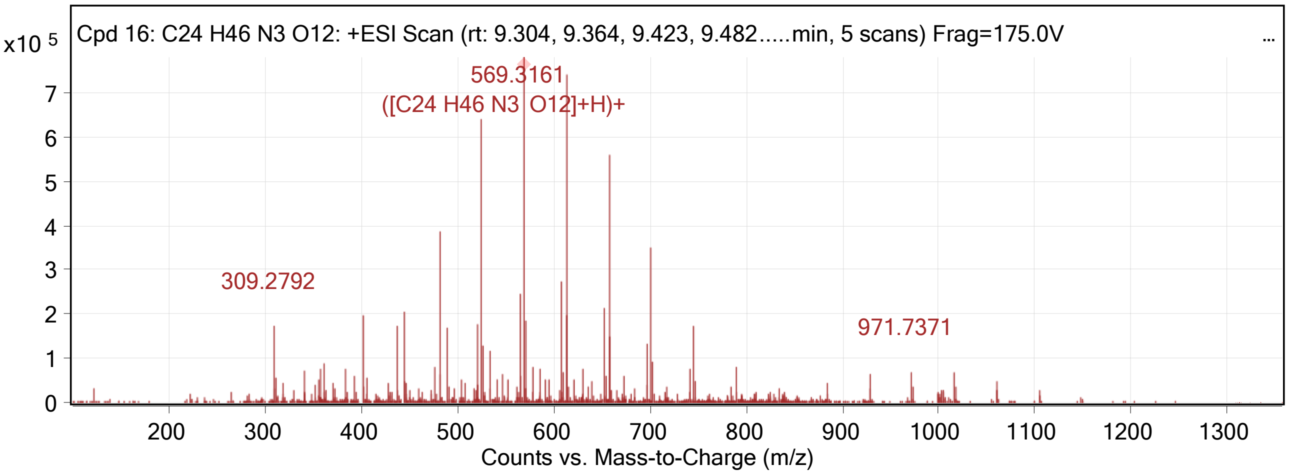

MS Zoomed Spectrum

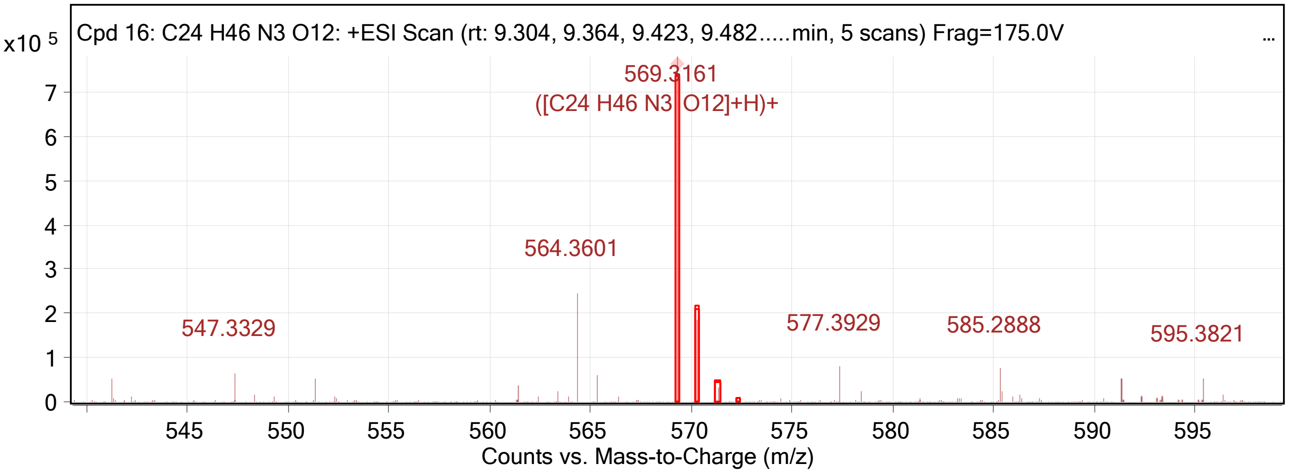

MS Spectrum Peak List

m/z	Calc *m/z*	Diff(ppm)	z	Abund	Formula	Ion
481.2629			1	386254.5		
525.2897			1	641333.38		
569.3161	569.3154	-1.25	1	781101.31	C24 H46 N3 O12	(M+H)+
570.3187	570.3186	-0.18	1	184962.52	C24 H46 N3 O12	(M+H)+
571.3205	571.3209	0.73	1	31408.57	C24 H46 N3 O12	(M+H)+
572.324	572.3235	-0.78	1	4724.56	C24 H46 N3 O12	(M+H)+
608.3865			1	273219.44		
613.3426			1	740289		

MSMS Spectrum

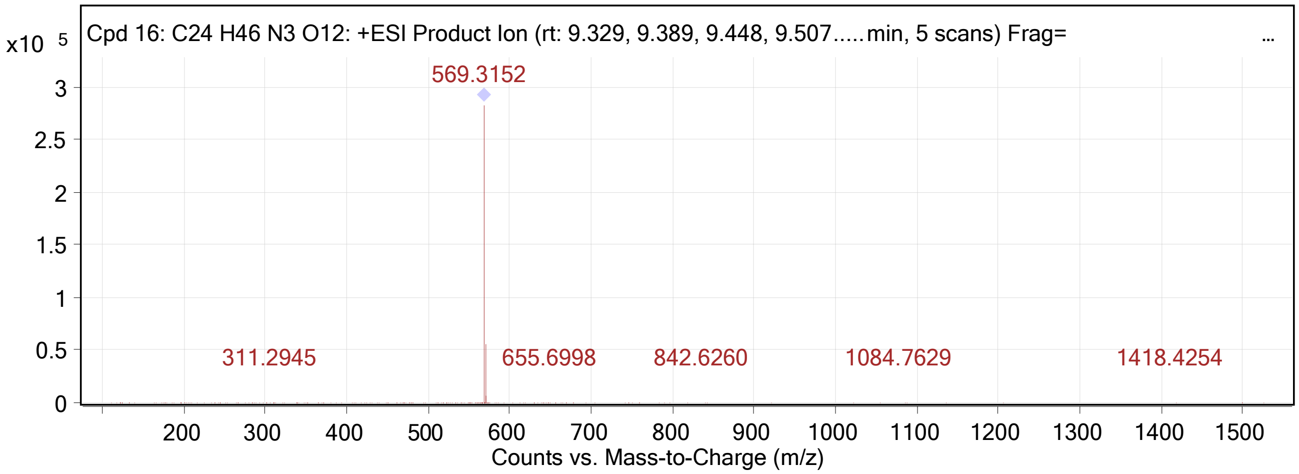

MS/MS Spectrum Peak List

m/z	*Calc m/z*	Diff(ppm)	z	Abund
111.0809	111.0804	-4.46		12.27
117.0909	117.091	0.52		25.65
175.1324	175.1329	2.55	1	26.33
209.1107	209.1132	12.18		12.63
243.1508	243.1465	-17.45		11.95
283.2621	283.2618	-0.91		23.2
309.276	309.2775	4.83		15.71
311.2945	311.2945	-0.14		33.1
408.3086	408.3082	-1.15		16.03
569.3152	569.3154	0.4	1	283153.81

Compound Label	*m/z*	RT	Algorithm	Mass
Cpd 17: C24 H46 N3 O12	569.3166	9.744	Auto MS/MS	568.3092

Compound Chromatograms

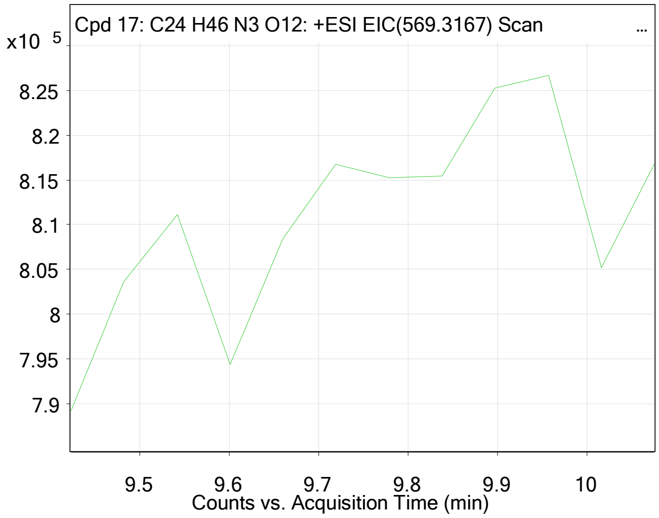

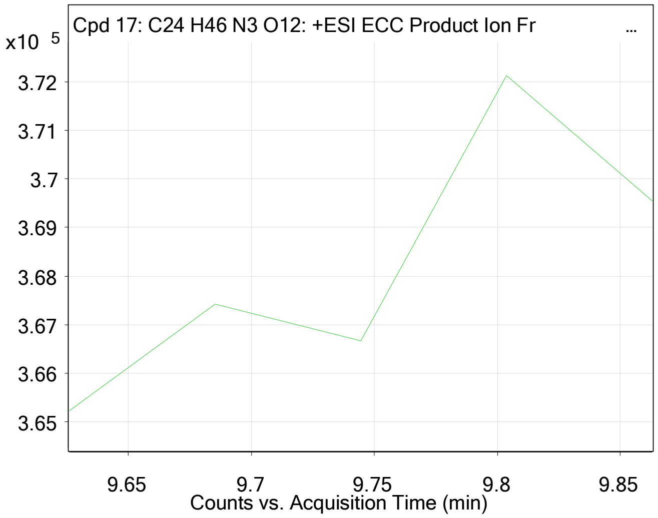

MS Spectrum

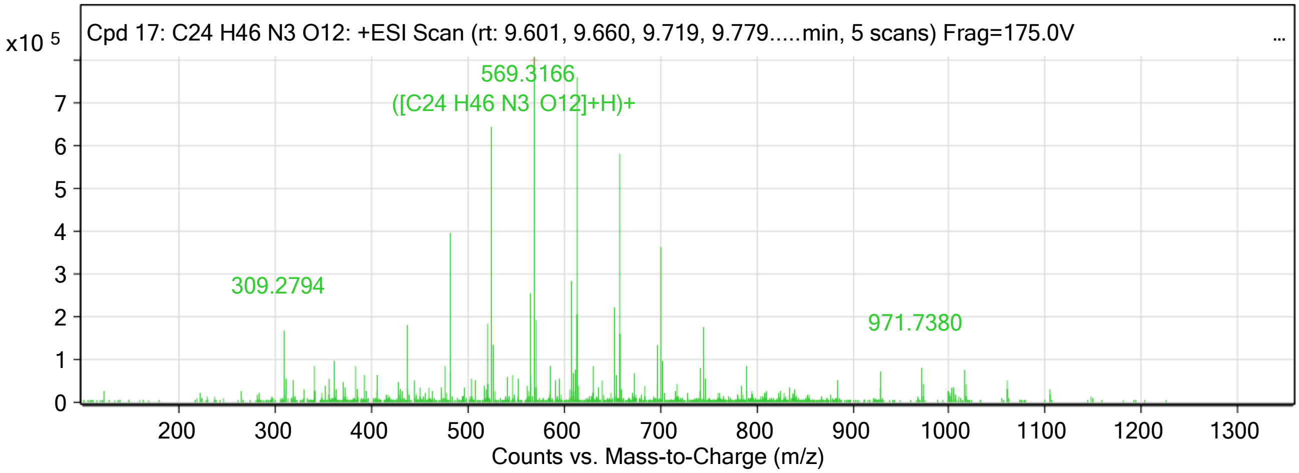

MS Zoomed Spectrum

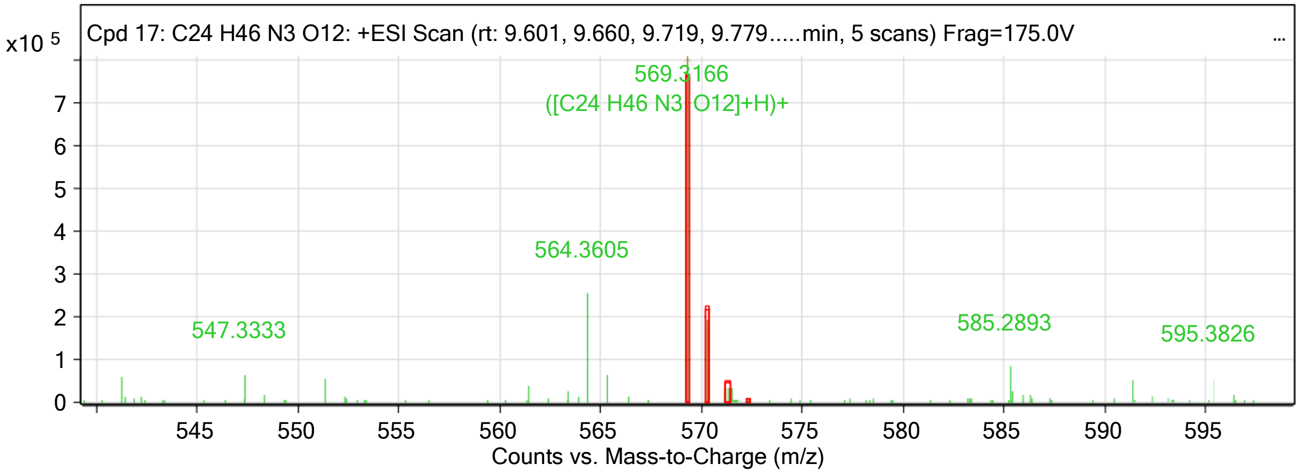

MS Spectrum Peak List

m/z	Calc *m/z*	Diff(ppm)	z	Abund	Formula	Ion
481.2633			1	396485.78		
525.2904			1	642817.88		
569.3166	569.3154	-2.09	1	810073.88	C24 H46 N3 O12	(M+H)+
570.3192	570.3186	-0.98	1	193765.47	C24 H46 N3 O12	(M+H)+
571.3211	571.3209	-0.24	1	33132.73	C24 H46 N3 O12	(M+H)+
572.3239	572.3235	-0.71	1	4814.83	C24 H46 N3 O12	(M+H)+
608.387			1	283568.19		
613.3431			1	759747.94		
657.3691			1	582333.63		
701.3952			1	362210.59		

MSMS Spectrum

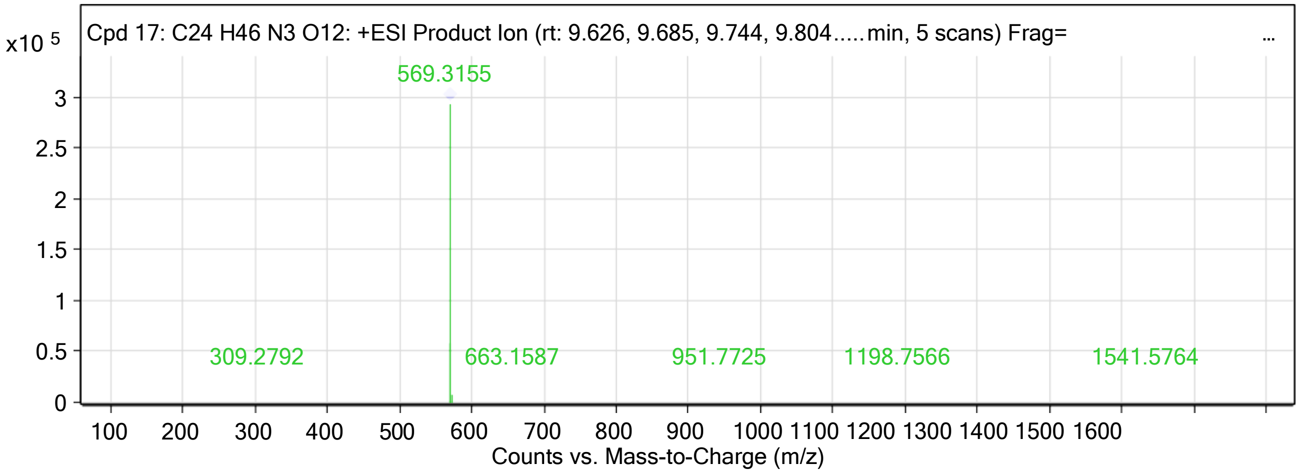

MS/MS Spectrum Peak List

m/z	*Calc m/z*	Diff(ppm)	z	Abund
115.0738	115.074	1.6		14.66
117.0912	117.091	-1.38		23.53
123.0414	123.0427	10.59		14.03
133.0865	133.0859	-4.13		14.75
143.1048	143.1053	3.73		22.19
272.0911	272.0891	-7.36		14.8
283.2635	283.2632	-1.1		24
309.2792	309.2788	-1.16	1	28.46
567.3	567.2998	-0.47		14.67
569.3155	569.3154	-0.18	1	293070.59

Compound Label	*m/z*	RT	Algorithm	Mass
Cpd 18: C28 H52 O14	613.3431	9.761	Auto MS/MS	612.3356

Compound Chromatograms

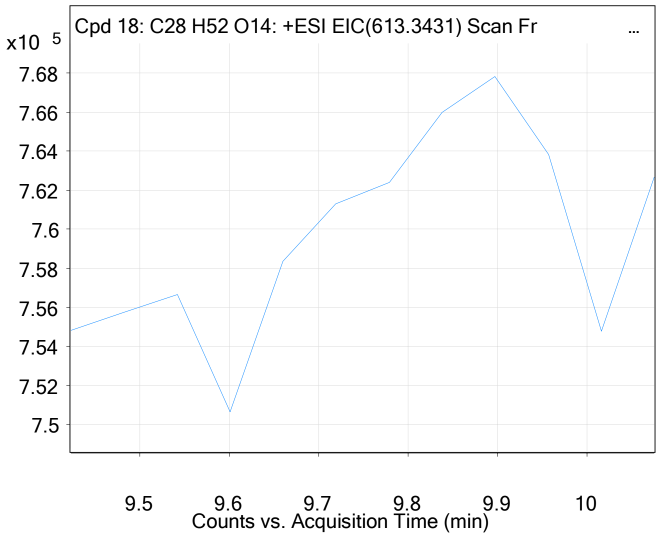

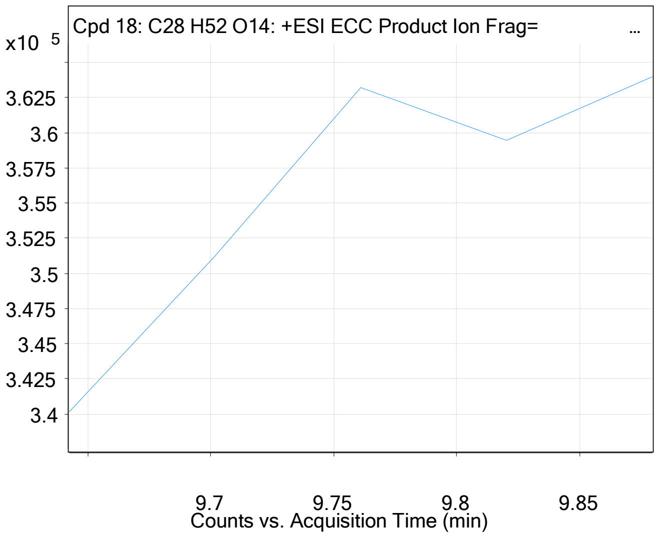

MS Spectrum

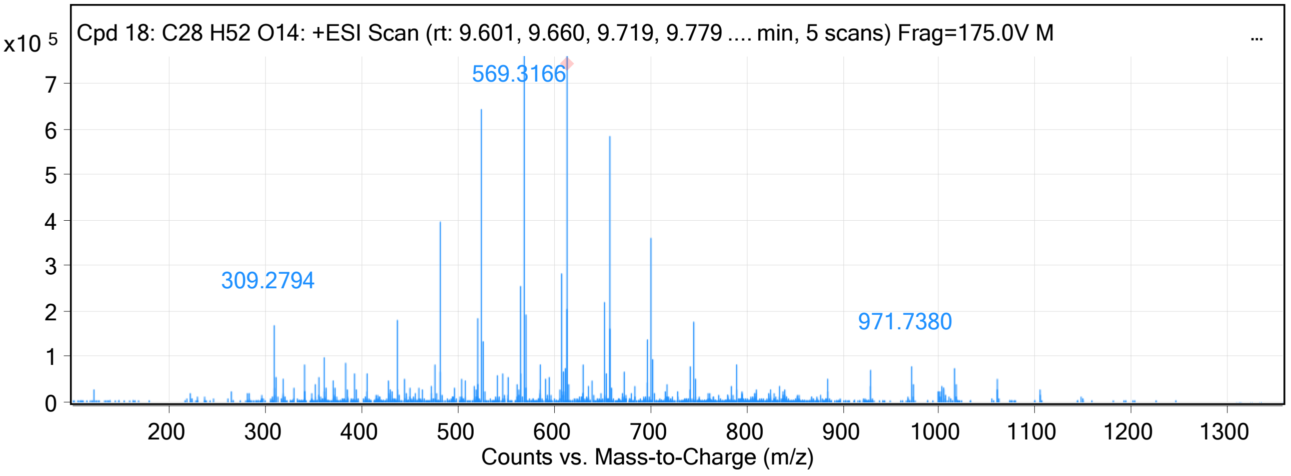

MS Zoomed Spectrum

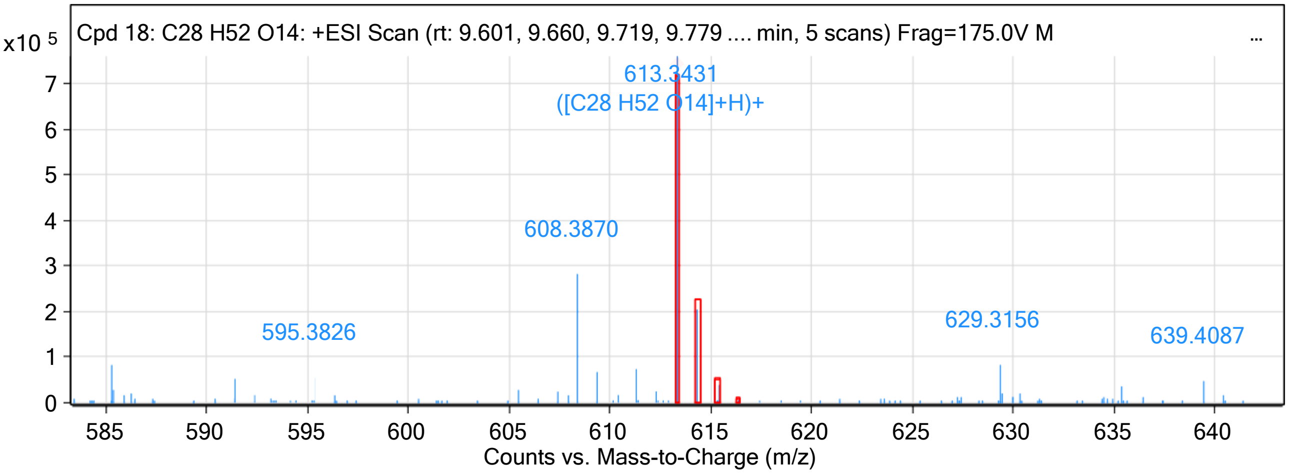

MS Spectrum Peak List

m/z	Calc *m/z*	Diff(ppm)	z	Abund	Formula	Ion
481.2633			1	396485.78		
525.2904			1	642817.88		
569.3166			1	810073.88		
613.3431	613.343	-0.18	1	759747.94	C28 H52 O14	(M+H)+
614.3456	614.3464	1.27	1	205618.77	C28 H52 O14	(M+H)+
615.3475	615.3489	2.13	1	37857.05	C28 H52 O14	(M+H)+
616.3528	616.3515	-2.07	1	5446.78	C28 H52 O14	(M+H)+
617.3642	617.354	-16.56	1	780.39	C28 H52 O14	(M+H)+
657.3691			1	582333.63		
701.3952			1	362210.59		

MSMS Spectrum

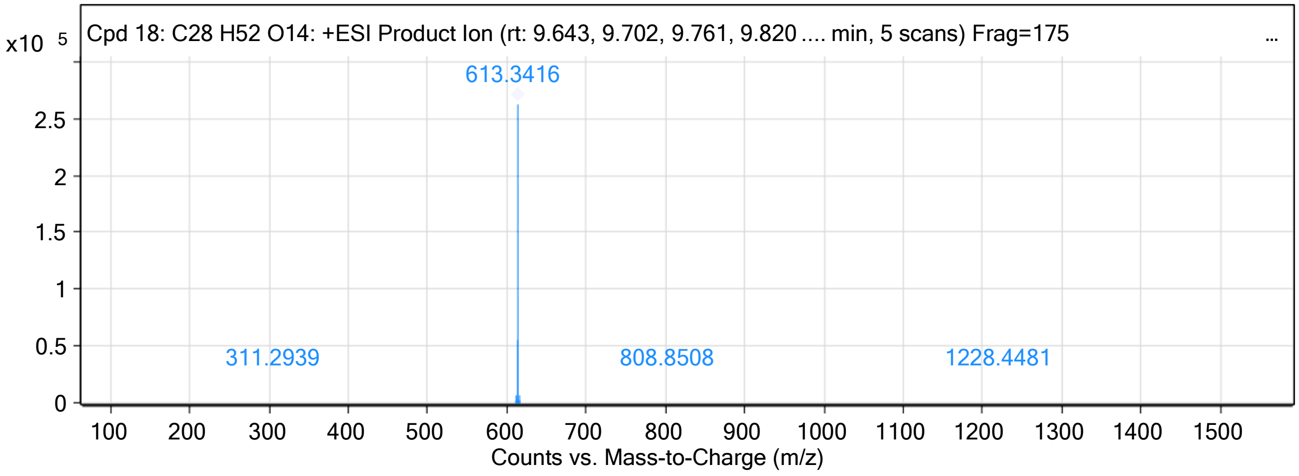

MS/MS Spectrum Peak List

m/z	Calc *m/z*	Diff(ppm)	z	Abund
133.0851	133.0859	5.97	1	42.37
309.2784	309.2788	1.47	1	119.3
312.2988	312.3023	11.21	1	36.8
357.0636	357.0664	7.87	1	28.56
612.2892			1	6226.84
612.6381				2730.84
613.2927			1	2242.29
613.3416	613.343	2.2	1	262526.81
614.3443			1	55375.81
615.3467			1	6784.2

Compound Label	*m/z*	RT	Algorithm	Mass
Cpd 19: C24 H46 N3 O12	569.3163	10.041	Auto MS/MS	568.3089

Compound Chromatograms

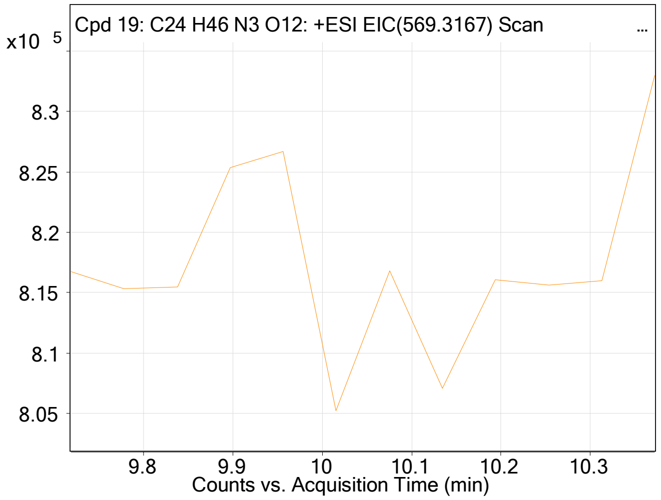

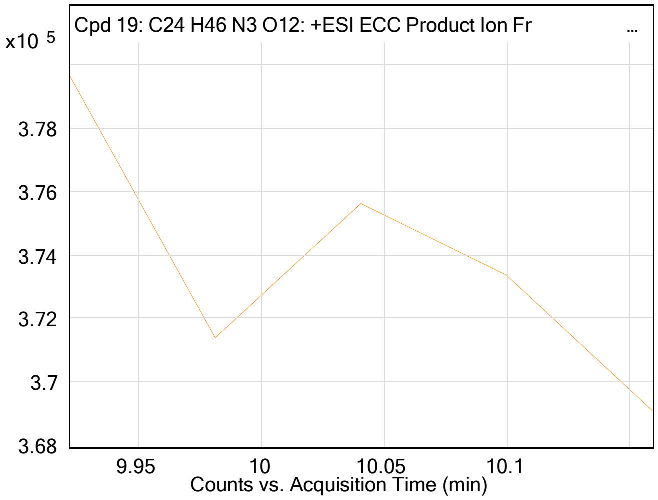

MS Spectrum

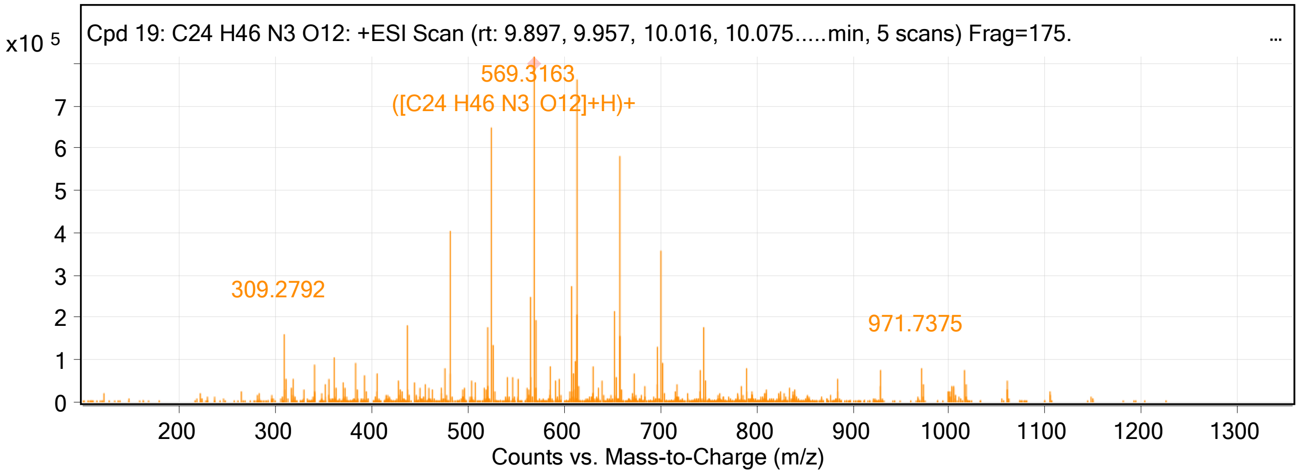

MS Zoomed Spectrum

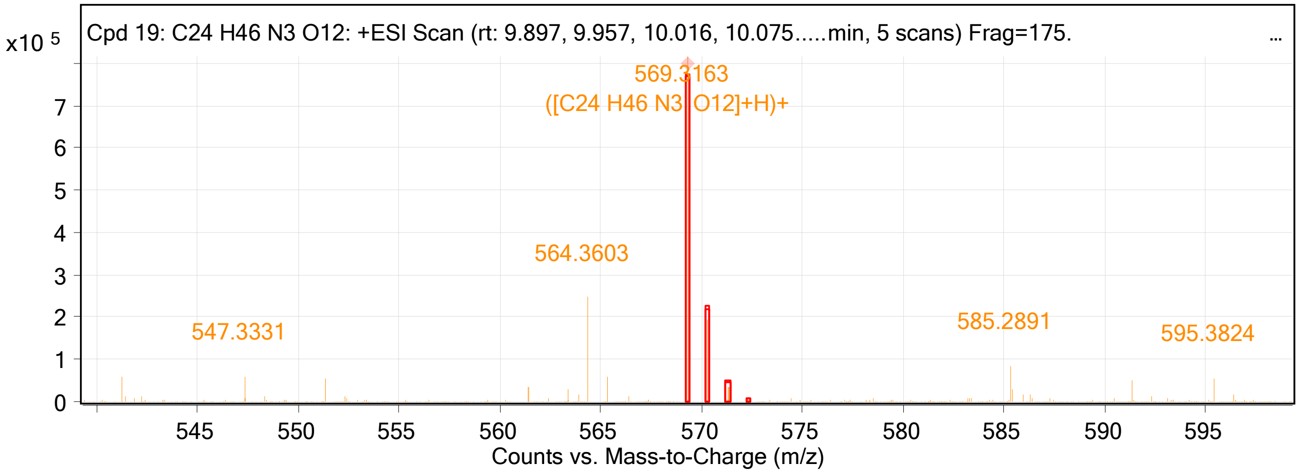

MS Spectrum Peak List

m/z	Calc *m/z*	Diff(ppm)	z	Abund	Formula	Ion
481.2631			1	402705.66		
525.2902			1	648700.75		
569.3163	569.3154	-1.59	1	816214.63	C24 H46 N3 O12	(M+H)+
570.3189	570.3186	-0.46	1	193713.91	C24 H46 N3 O12	(M+H)+
571.3208	571.3209	0.25	1	33026.93	C24 H46 N3 O12	(M+H)+
572.3234	572.3235	0.27	1	4759.2	C24 H46 N3 O12	(M+H)+
608.3867			1	274346.66		
613.3428			1	761718.25		
657.3688			1	580994.31		

MSMS Spectrum

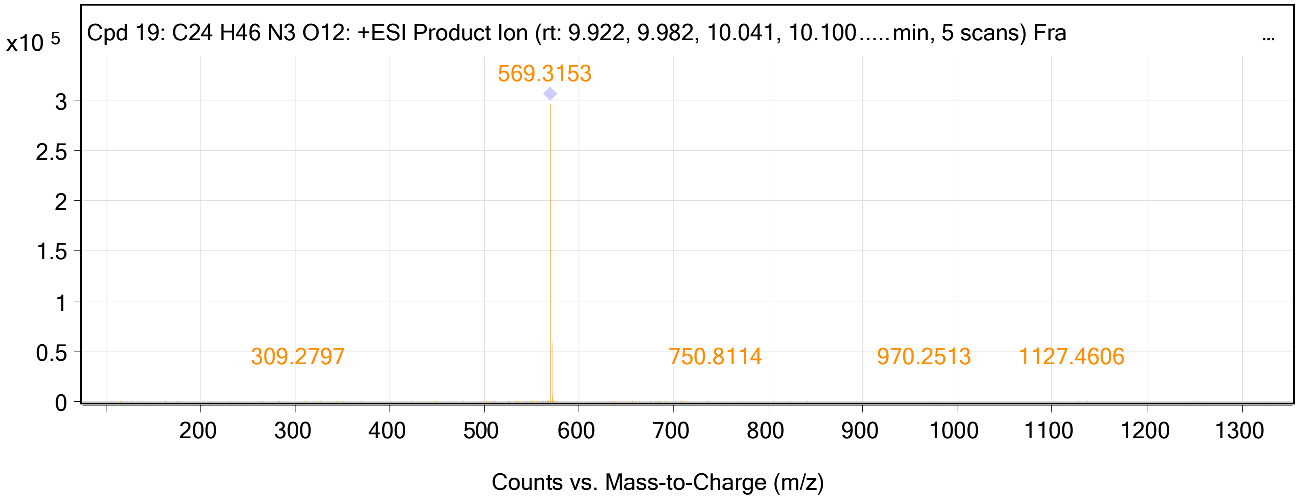

MS/MS Spectrum Peak List

m/z	*Calc m/z*	Diff(ppm)	z	Abund
123.0415	123.0427	9.76		21.01
133.0877	133.0886	6.86		25.87
208.1413	208.1418	2.39		16.84
262.1479	262.1523	16.93		18.37
282.2773	282.2791	6.43		16.8
283.2634	283.2632	-0.8		17.61
309.2797	309.2788	-3.03		35.93
538.2955	538.297	2.91		14.38
566.2915	566.292	0.78		21.27
569.3153	569.3154	0.19	1	296431.66

Compound Label	*m/z*	RT	Algorithm	Mass
Cpd 20: C26 H50 N3 O13	613.3428	10.057	Auto MS/MS	612.3353

Compound Chromatograms

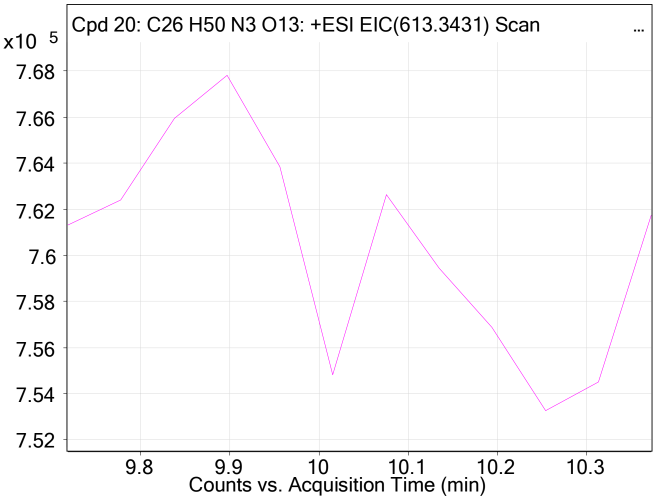

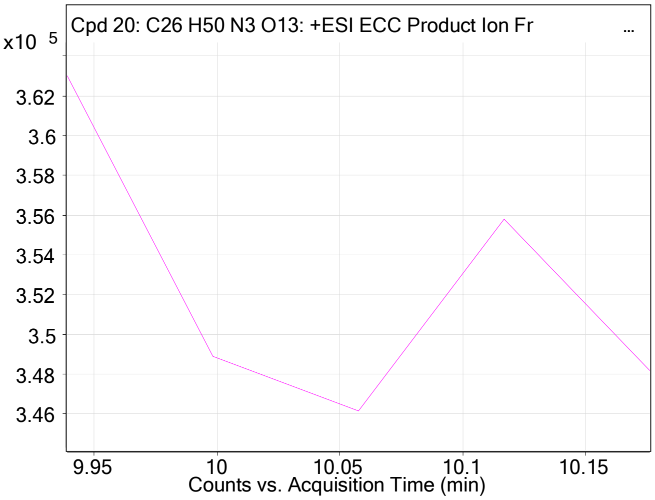

MS Spectrum

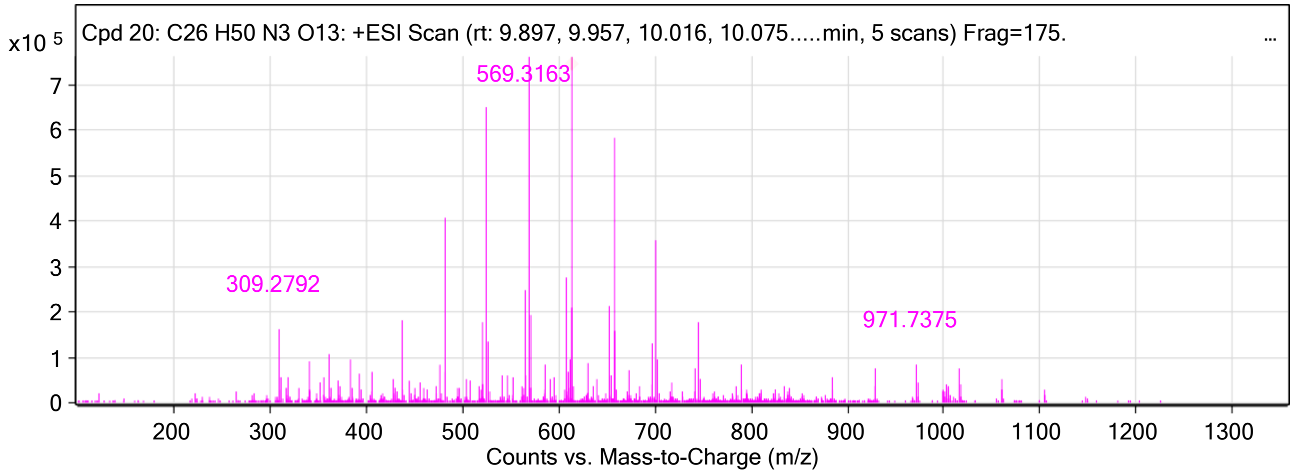

MS Zoomed Spectrum

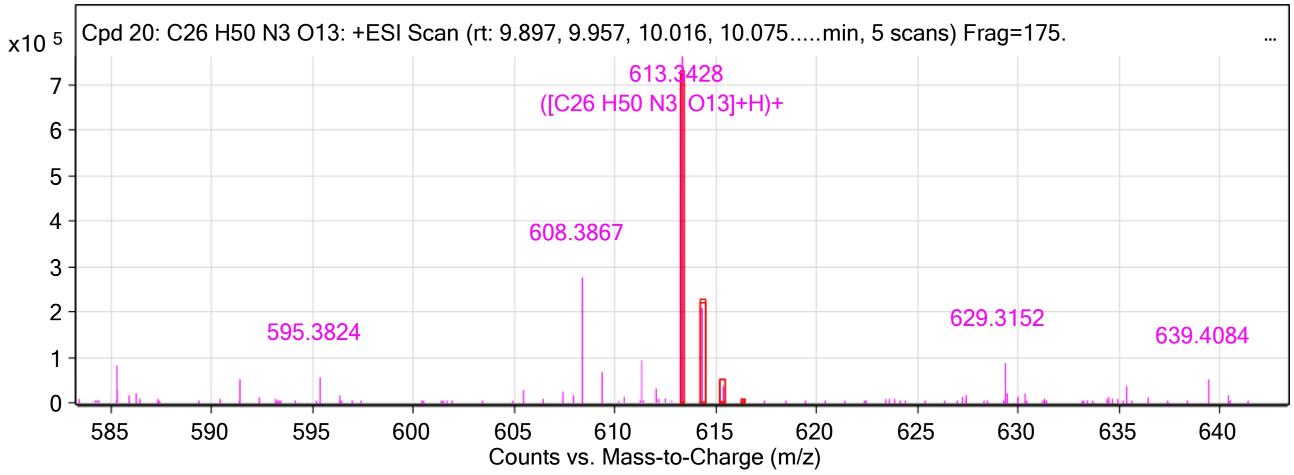

MS Spectrum Peak List

m/z	Calc *m/z*	Diff(ppm)	z	Abund	Formula	Ion
481.2631			1	402705.66		
525.2902			1	648700.75		
569.3163			1	816214.63		
608.3867			1	274346.66		
613.3428	613.3416	-1.85	1	761718.25	C26 H50 N3 O13	(M+H)+
614.3453	614.3448	-0.76	1	206921.02	C26 H50 N3 O13	(M+H)+
615.3473	615.3472	-0.1	1	36959.32	C26 H50 N3 O13	(M+H)+
616.3534	616.3498	-5.89	1	5509.92	C26 H50 N3 O13	(M+H)+
657.3688			1	580994.31		
701.3948			1	359290.19		

MSMS Spectrum

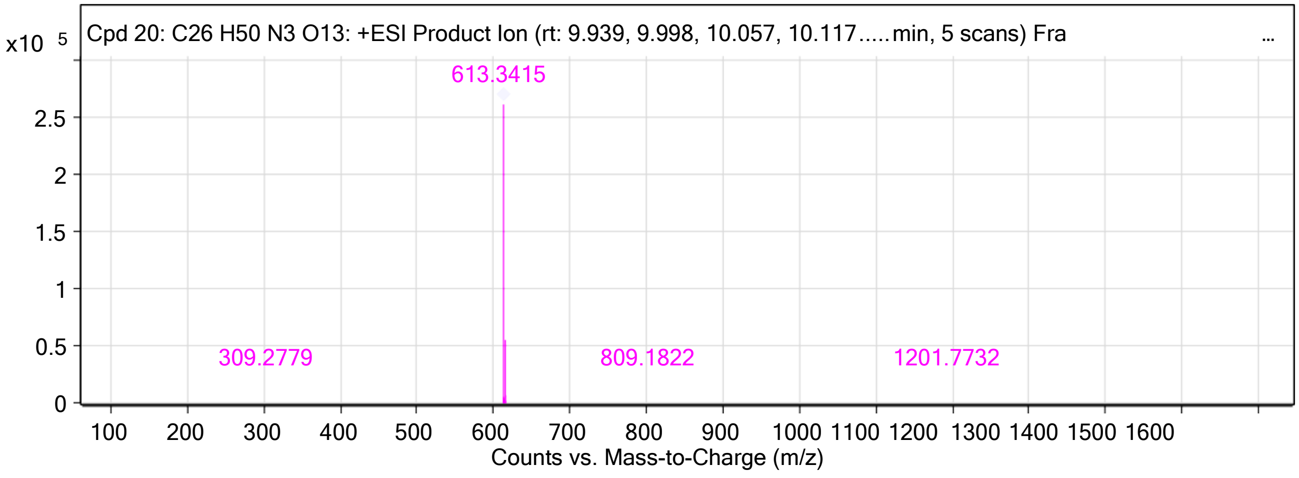

MS/MS Spectrum Peak List

m/z	*Calc m/z*	Diff(ppm)	z	Abund
309.2779	309.2775	-1.39	1	102.04
612.2895			1	4513.53
612.387			2	2239.98
612.6378				2922.68
612.8887			2	2329.08
613.1398			1	1212.11
613.2917			1	1911.88
613.3415	613.3416	0.16	1	261858.58
614.3443			1	54949.06
615.3463			1	6893.73

Compound Label	*m/z*	RT	Algorithm	Mass
Cpd 21: C24 H46 N3 O12	569.3165	10.337	Auto MS/MS	568.309

Compound Chromatograms

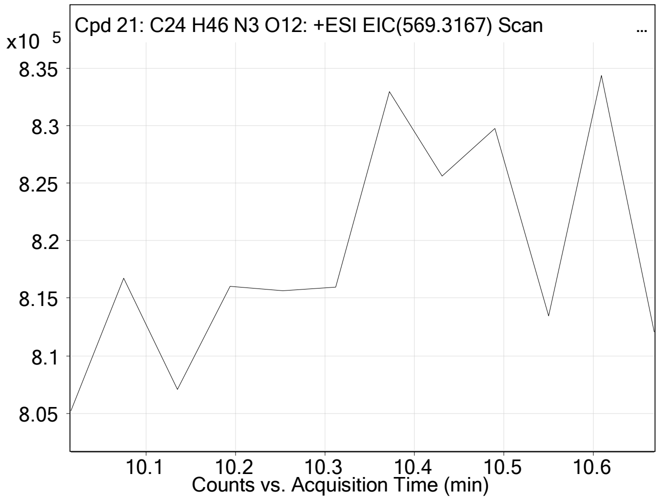

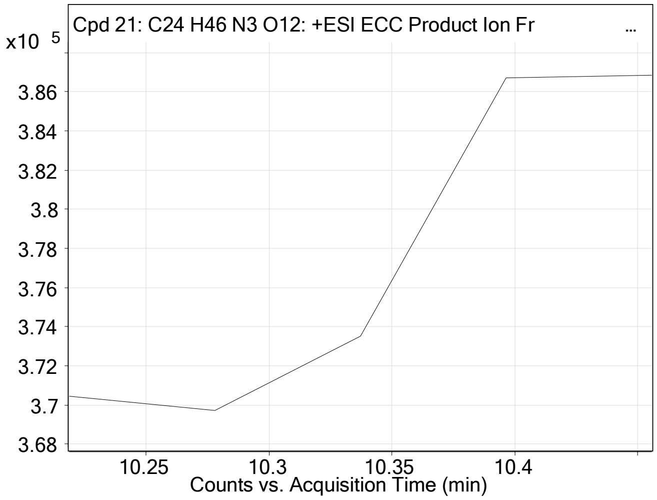

MS Spectrum

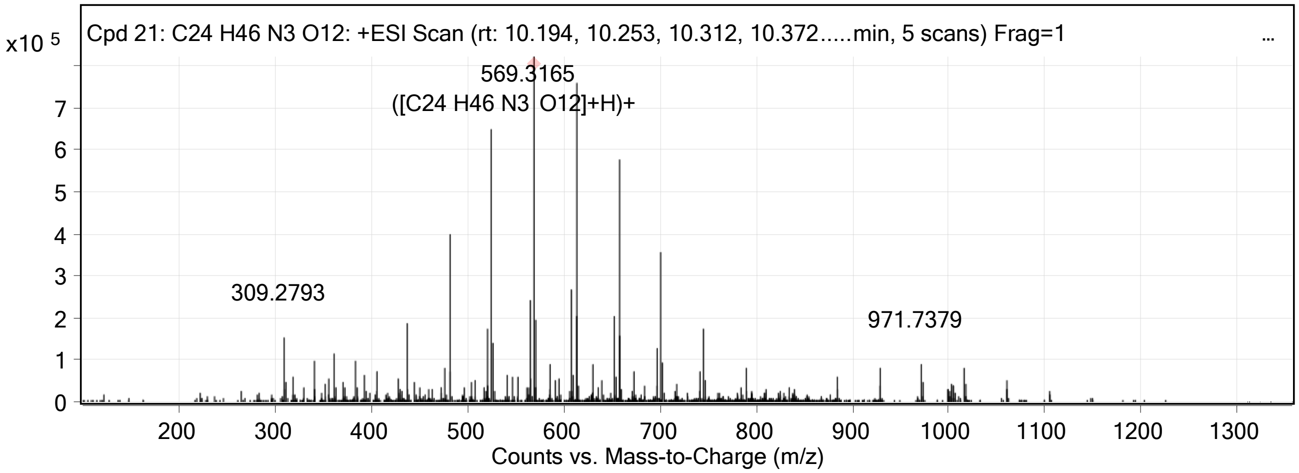

MS Zoomed Spectrum

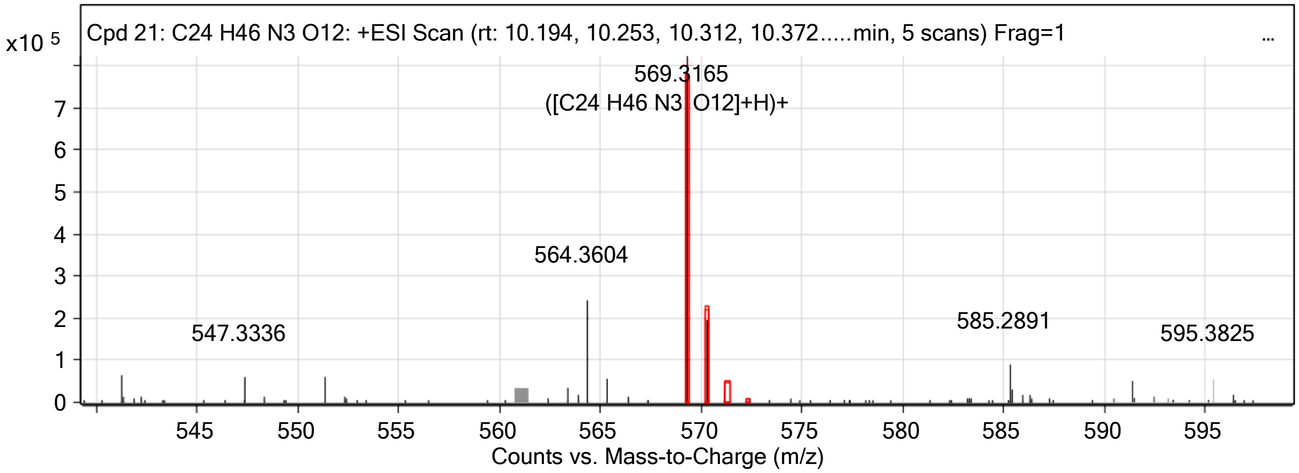

MS Spectrum Peak List

m/z	Calc *m/z*	Diff(ppm)	z	Abund	Formula	Ion
481.2632			1	399271.56		
525.2903			1	648719.69		
569.3165	569.3154	-1.86	1	821254.88	C24 H46 N3 O12	(M+H)+
570.319	570.3186	-0.73	1	195571.09	C24 H46 N3 O12	(M+H)+
571.3208	571.3209	0.18	1	32940.66	C24 H46 N3 O12	(M+H)+
572.3238	572.3235	-0.49	1	4841.96	C24 H46 N3 O12	(M+H)+
608.3868			1	266187.63		
613.343			1	758859.88		
657.3689			1	575207.69		
701.395			1	355478.13		

MSMS Spectrum

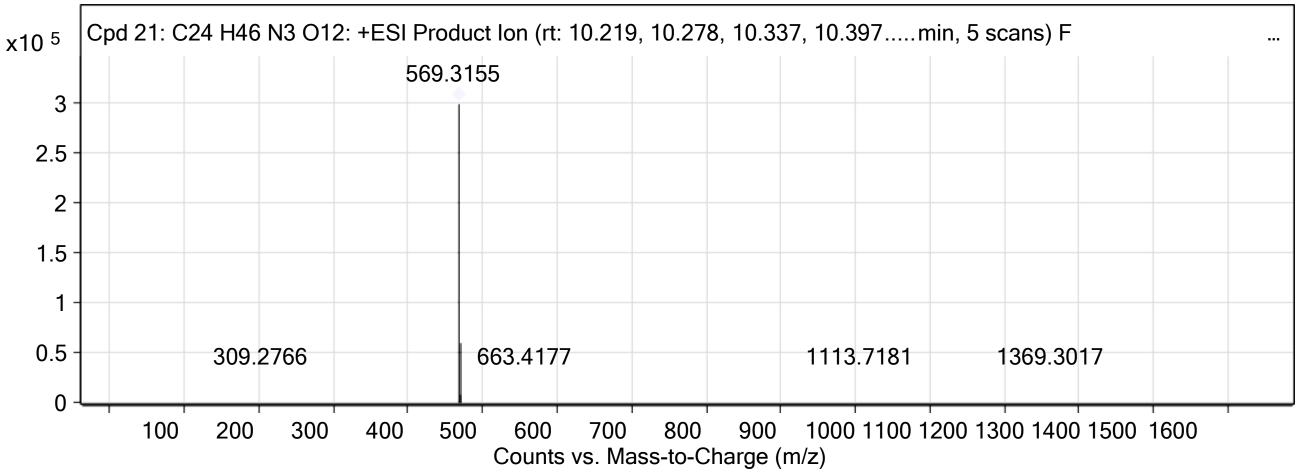

MS/MS Spectrum Peak List

m/z	*Calc m/z*	Diff(ppm)	z	Abund
117.0912	117.091	-1.92		29.71
133.0867	133.0859	-6		38.45
283.2645	283.2632	-4.82		51.88
309.2766	309.2775	2.78	1	86.28
327.1575	327.1524	-15.77		17.02
422.3108	211.1553	-500000.1	2	22.18
451.2819	451.2776	-9.56		16.92
536.2772	536.2814	7.86		17.52
566.2967	566.292	-8.43		29.78
569.3155	569.3154	-0.09	1	299229.75

Compound Label	*m/z*	RT	Algorithm	Mass
Cpd 22: C28 H52 O14	613.343	10.354	Auto MS/MS	612.3355

Compound Chromatograms

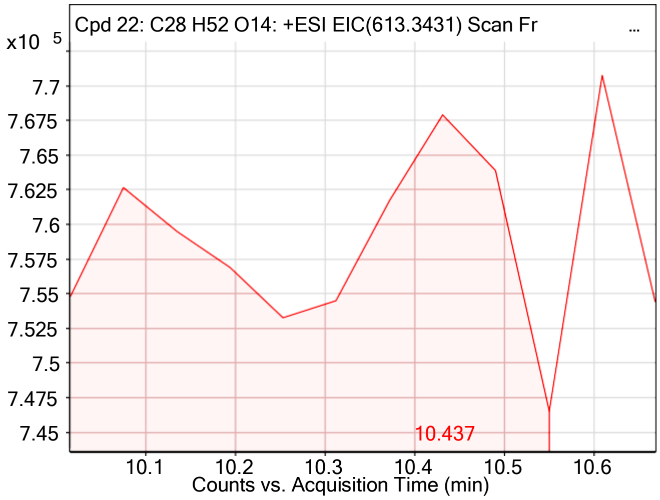

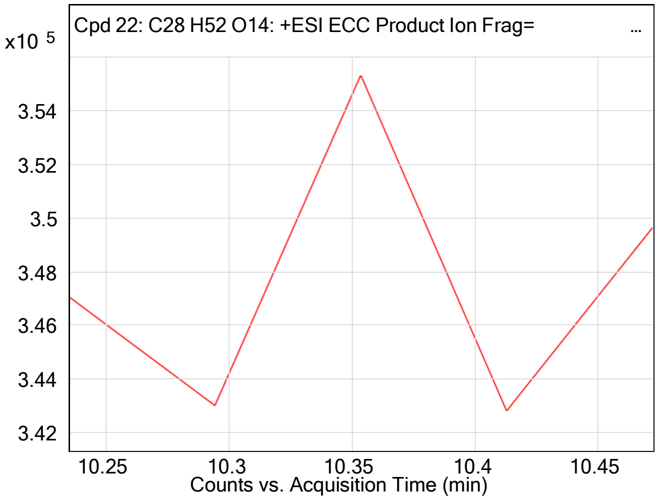

MS Spectrum

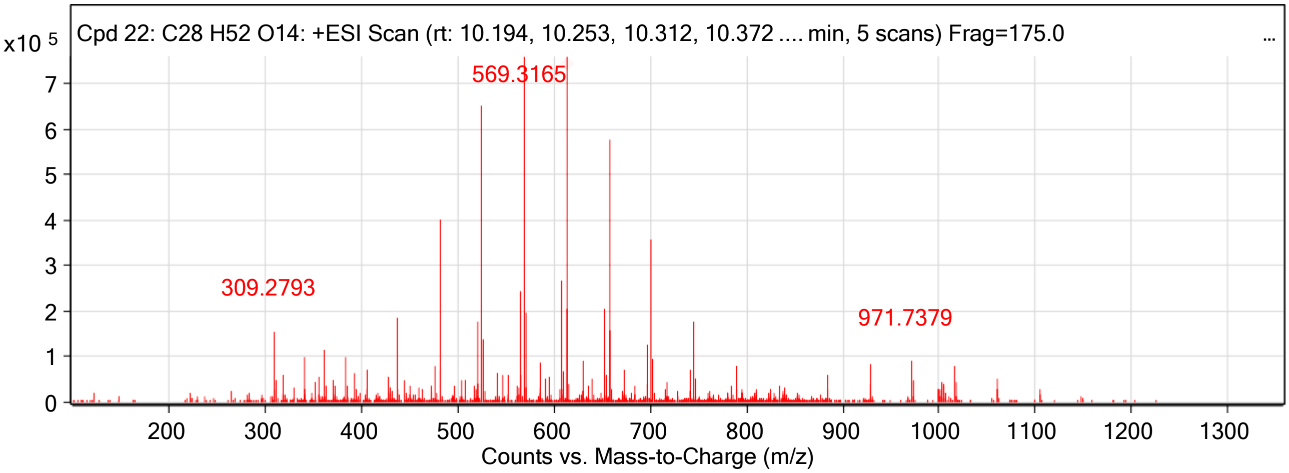

MS Zoomed Spectrum

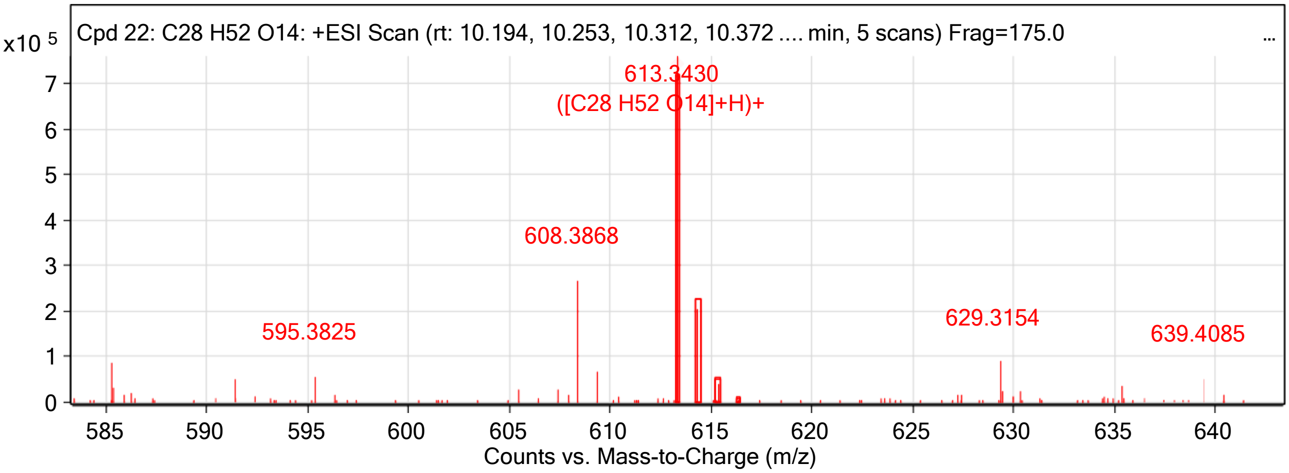

MS Spectrum Peak List

m/z	Calc *m/z*	Diff(ppm)	z	Abund	Formula	Ion
481.2632			1	399271.56		
525.2903			1	648719.69		
569.3165			1	821254.88		
608.3868			1	266187.63		
613.343	613.343	0.02	1	758859.88	C28 H52 O14	(M+H)+
614.3455	614.3464	1.47	1	205072.28	C28 H52 O14	(M+H)+
615.3475	615.3489	2.14	1	37730.02	C28 H52 O14	(M+H)+
616.3537	616.3515	-3.49	1	5407.07	C28 H52 O14	(M+H)+
657.3689			1	575207.69		

MSMS Spectrum

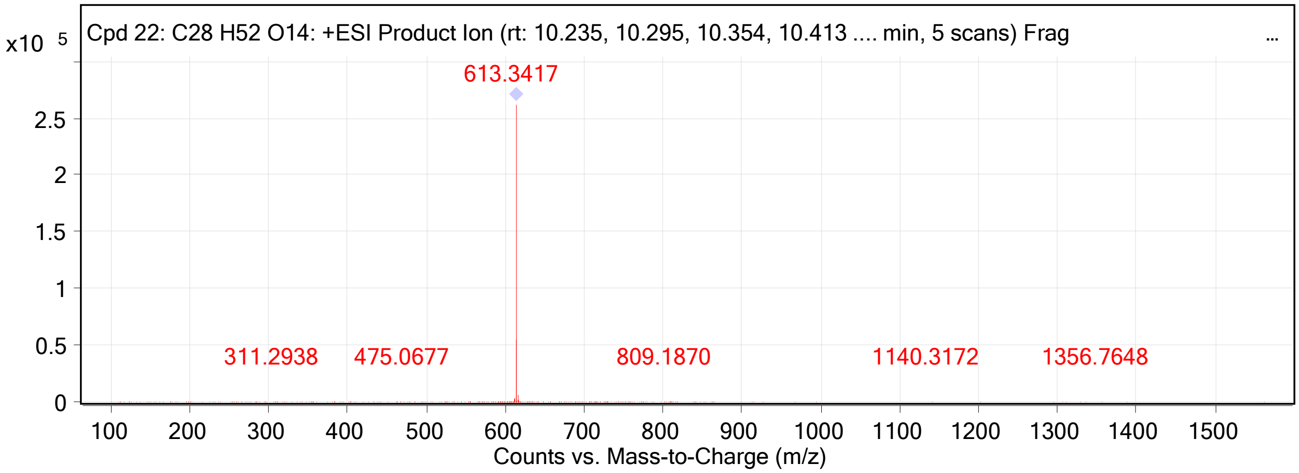

MS/MS Spectrum Peak List

m/z	*Calc m/z*	Diff(ppm)	z	Abund
133.0853	133.0859	4.69		24.51
177.1121	177.1121	0.31		26.3
254.1412	254.136	-20.29		26.97
309.2786	309.2788	0.58	1	102.41
609.3199	304.6556	-500007.19	2	57.3
612.3875			2	2468.19
612.6375				2892.04
613.3417	613.343	2.17	1	262161.13
614.3444			1	55400.41
615.3468			1	6702.06

Compound Label	*m/z*	RT	Algorithm	Mass
Cpd 23: C24 H46 N3 O12	569.3166	10.634	Auto MS/MS	568.3092

Compound Chromatograms

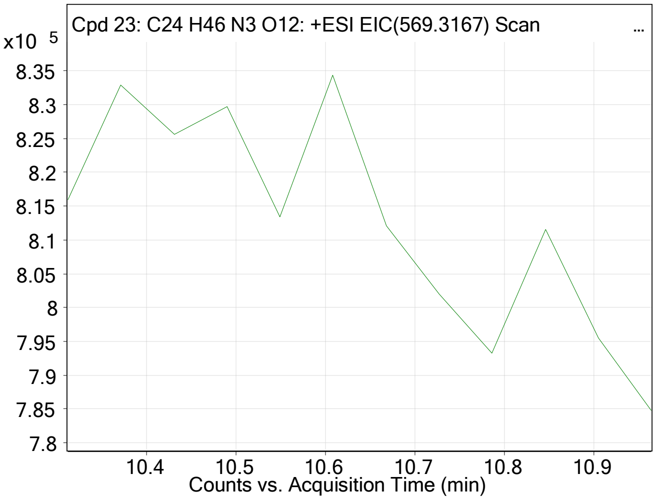

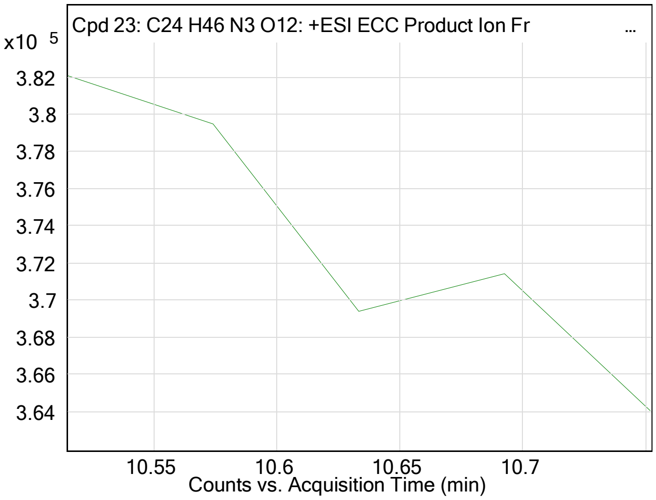

MS Spectrum

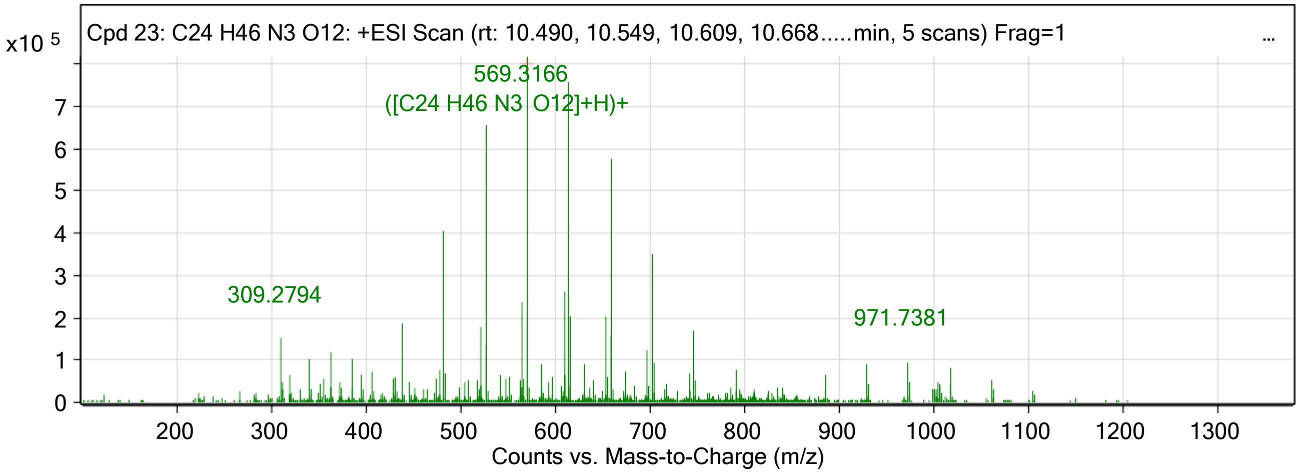

MS Zoomed Spectrum

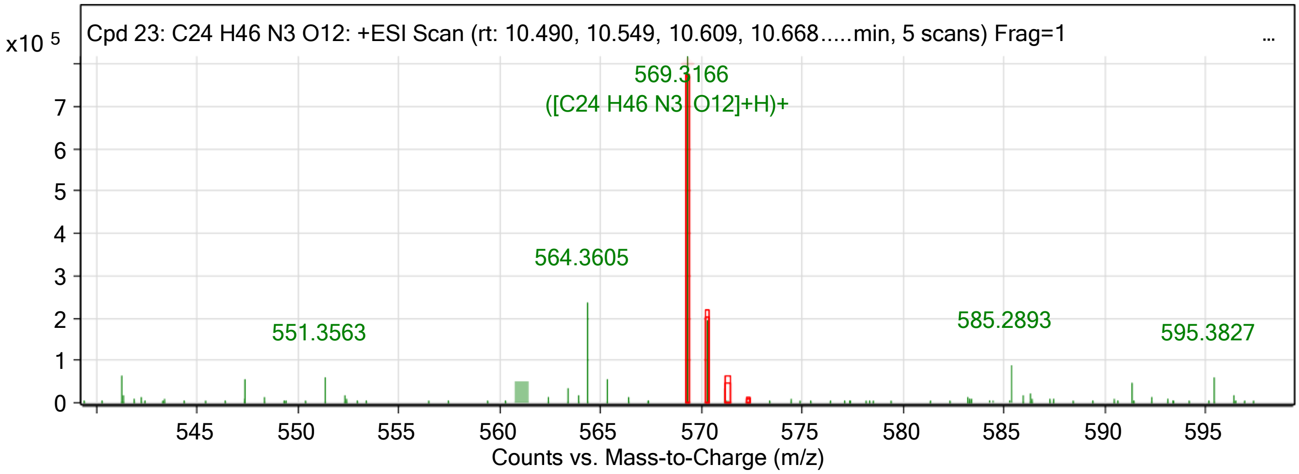

MS Spectrum Peak List

m/z	Calc *m/z*	Diff(ppm)	z	Abund	Formula	Ion
481.2633			1	405086.84		
525.2904			1	653549.44		
569.3166	569.3154	-2.13	1	818333	C24 H46 N3 O12	(M+H)+
570.3192	570.3186	-0.97	1	192433.03	C24 H46 N3 O12	(M+H)+
571.321	571.3209	-0.15	1	32762.05	C24 H46 N3 O12	(M+H)+
572.3238	572.3235	-0.47	1	4935.98	C24 H46 N3 O12	(M+H)+
608.387			1	261198.34		
613.3431			1	755781.63		
657.3691			1	573130.88		
701.3952			1	350273.47		

MSMS Spectrum

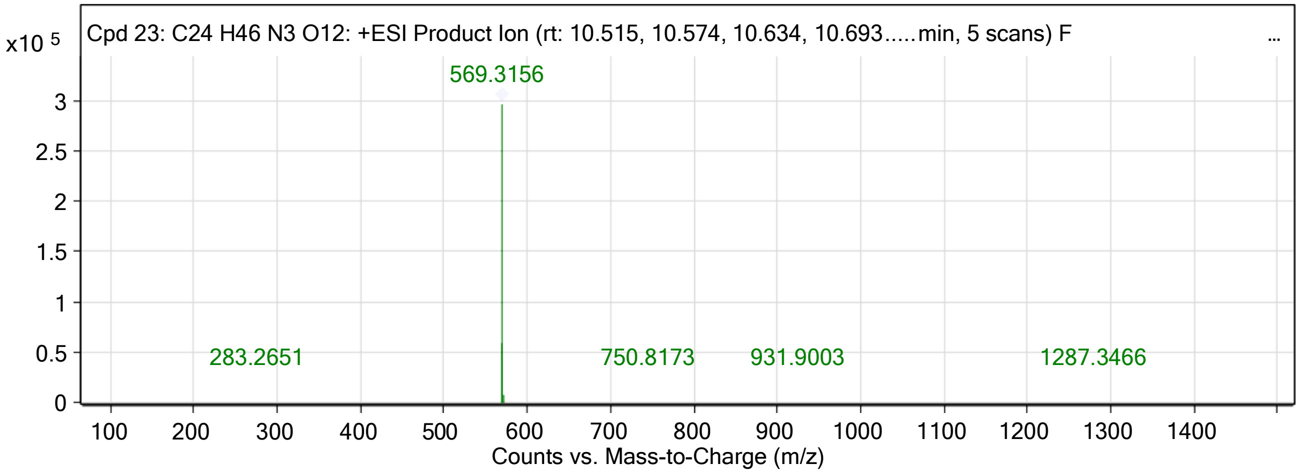

MS/MS Spectrum Peak List

m/z	*Calc m/z*	Diff(ppm)	z	Abund
133.0874	133.0886	8.91		14.4
177.1125	177.1121	-2.32		15.32
244.1683	244.1669	-5.52		13.98
283.2651	283.2632	-6.73		33.49
309.2769	309.2775	1.74		20.59
377.2659	377.2646	-3.36		13.81
381.188	381.1868	-3.3		11.8
386.2337	386.2299	-9.93		18.06
422.3096	211.1553	-499998.73	2	17.33
569.3156	569.3154	-0.26	1	296444.75

Compound Label	*m/z*	RT	Algorithm	Mass
Cpd 24: C28 H52 O14	613.3431	10.65	Auto MS/MS	612.3356

Compound Chromatograms

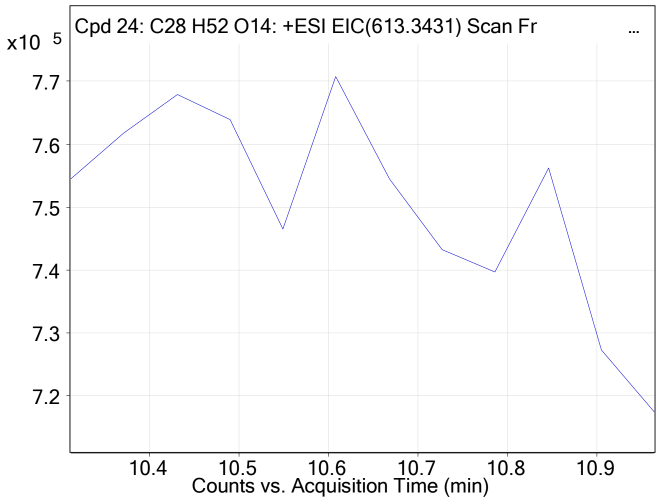

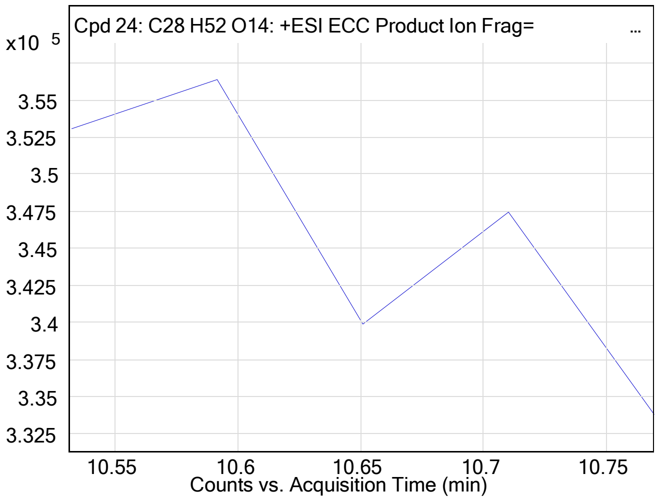

MS Spectrum

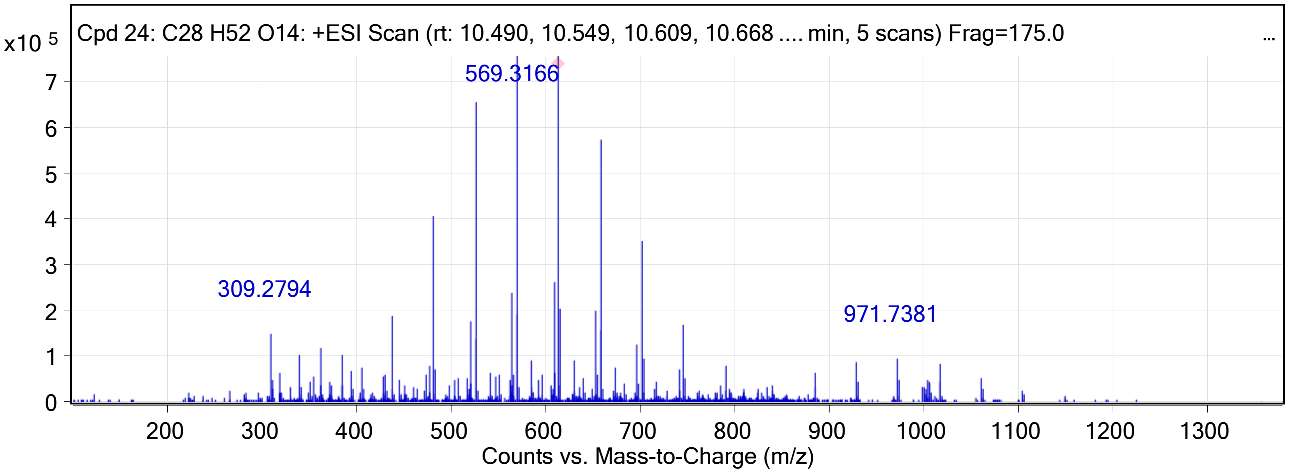

MS Zoomed Spectrum

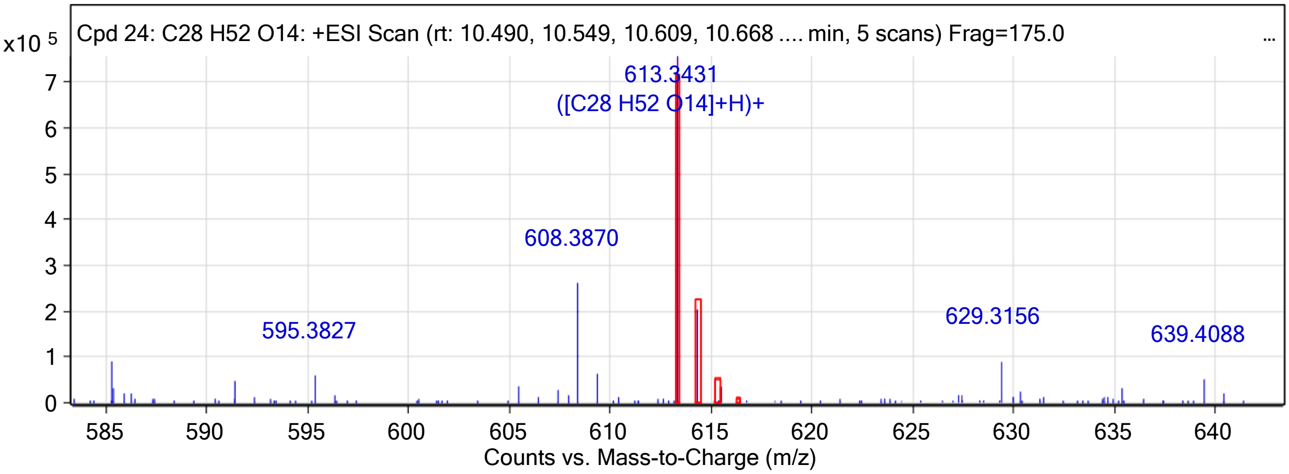

MS Spectrum Peak List

m/z	Calc *m/z*	Diff(ppm)	z	Abund	Formula	Ion
481.2633			1	405086.84		
525.2904			1	653549.44		
569.3166			1	818333		
608.387			1	261198.34		
613.3431	613.343	-0.22	1	755781.63	C28 H52 O14	(M+H)+
614.3456	614.3464	1.31	1	204375.63	C28 H52 O14	(M+H)+
615.3477	615.3489	1.95	1	36835.82	C28 H52 O14	(M+H)+
616.3542	616.3515	-4.27	1	5391.58	C28 H52 O14	(M+H)+
657.3691			1	573130.88		
701.3952			1	350273.47		

MSMS Spectrum

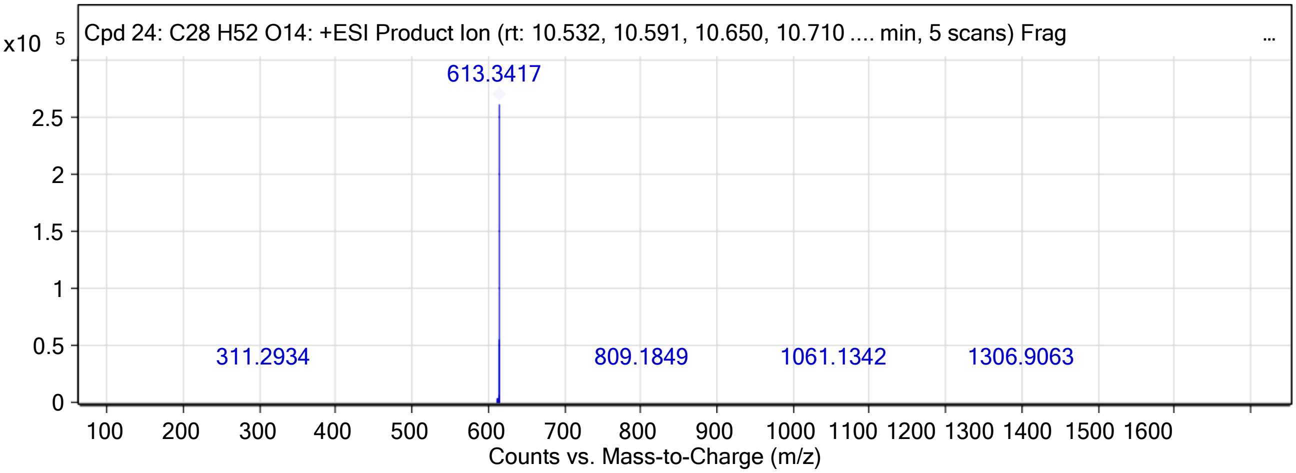

MS/MS Spectrum Peak List

m/z	*Calc m/z*	Diff(ppm)	z	Abund
309.2794	309.2788	-1.98	1	94.31
609.3103	304.6556	-499999.33	2	74.45
612.3873			2	2516.74
612.6383				3322.6
612.8889			2	2542.01
613.1399			1	1416.17
613.3417	613.343	2.02	1	260712.05
614.3445			1	53979.98
615.3467			1	6836.98
615.4121			2	858.29

Compound Label	*m/z*	RT	Algorithm	Mass
Cpd 25: C26 H48 O13	569.3169	10.93	Auto MS/MS	568.3094

Compound Chromatograms

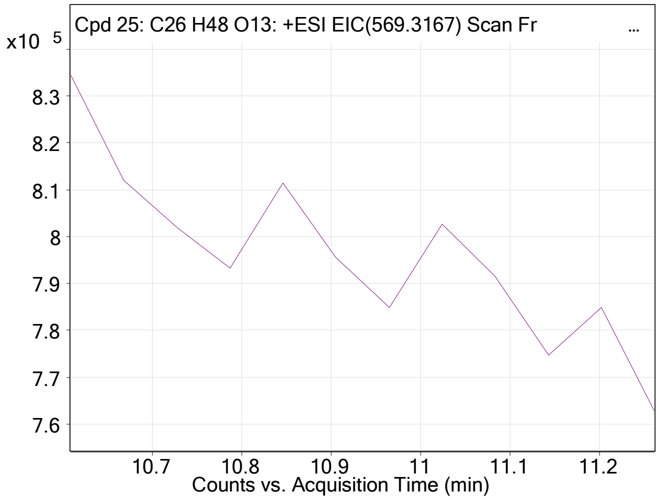

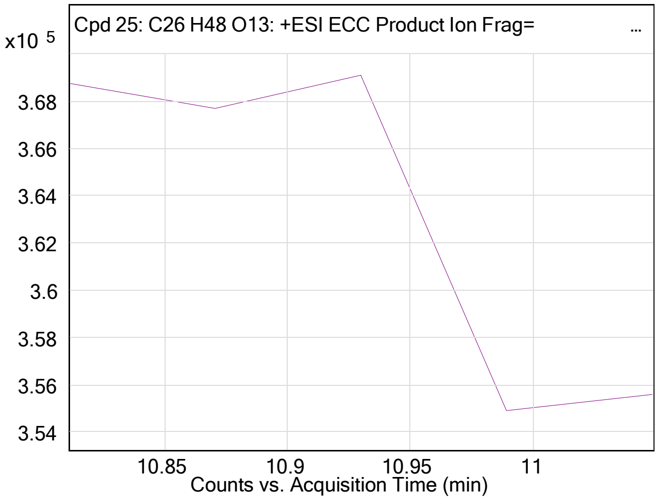

MS Spectrum

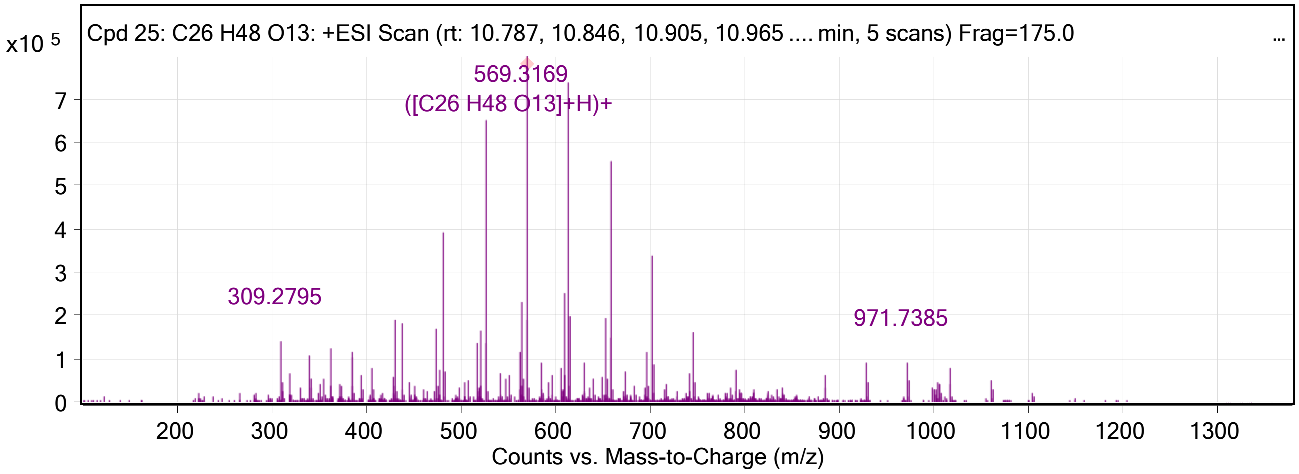

MS Zoomed Spectrum

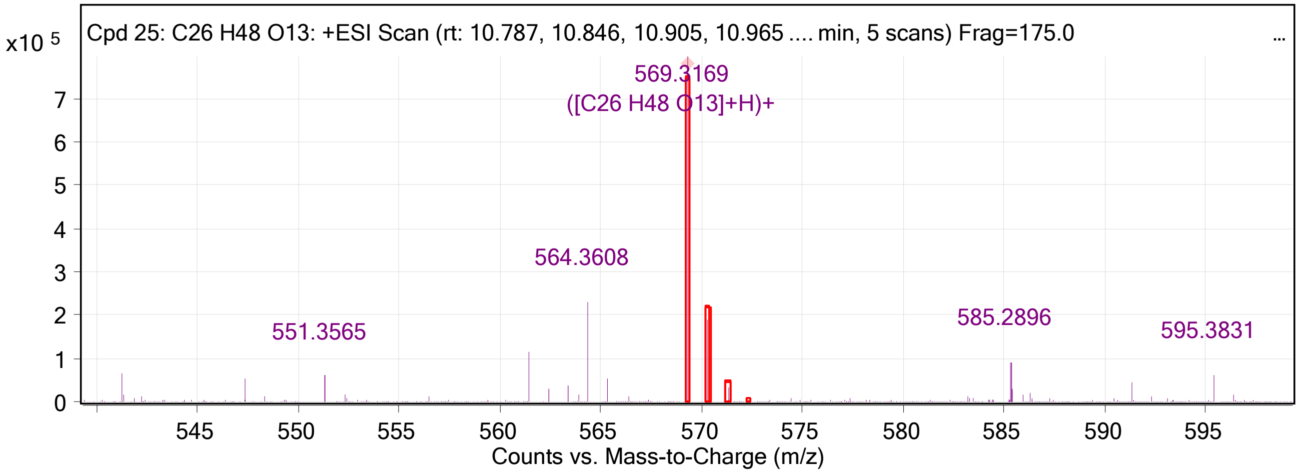

MS Spectrum Peak List

m/z	Calc *m/z*	Diff(ppm)	z	Abund	Formula	Ion
481.2636			1	392387.63		
525.2905			1	651139.31		
569.3169	569.3168	-0.31	1	797573.63	C26 H48 O13	(M+H)+
570.3195	570.3202	1.28	1	190473.33	C26 H48 O13	(M+H)+
571.3213	571.3226	2.22	1	31422.52	C26 H48 O13	(M+H)+
572.3241	572.3253	2.02	1	4700.17	C26 H48 O13	(M+H)+
608.3872			1	250745.34		
613.3434			1	734516.75		
657.3694			1	555389.63		

MSMS Spectrum

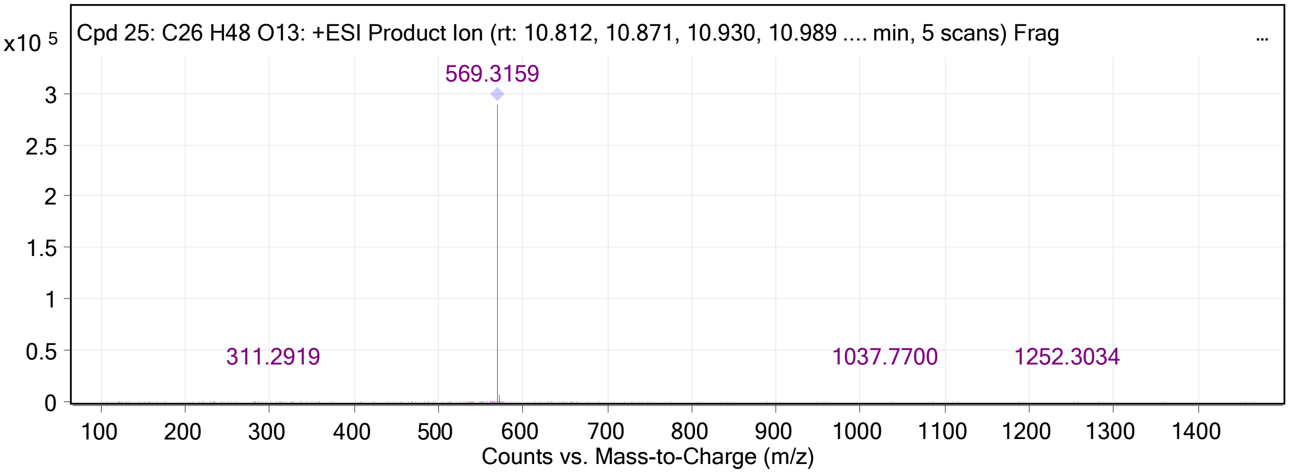

MS/MS Spectrum Peak List

m/z	*Calc m/z*	Diff(ppm)	z	Abund
133.0873	133.0859	-10.6		24.42
157.1219	157.1223	2.31		11.82
232.129	232.1305	6.44		22.89
235.1626	235.1693	28.44		12.22
311.2919	311.2945	8.09		26.41
312.2979	312.3023	13.92		21.13
495.3114	495.3164	9.98		11.36
531.3026	531.3011	-2.74		19.61
566.2986	283.1464	-500005.14	2	53.71
569.3159	569.3168	1.51	1	289283.22

Compound Label	*m/z*	RT	Algorithm	Mass
Cpd 26: C24 H46 N3 O12	569.3166	11.227	Auto MS/MS	568.3092

Compound Chromatograms

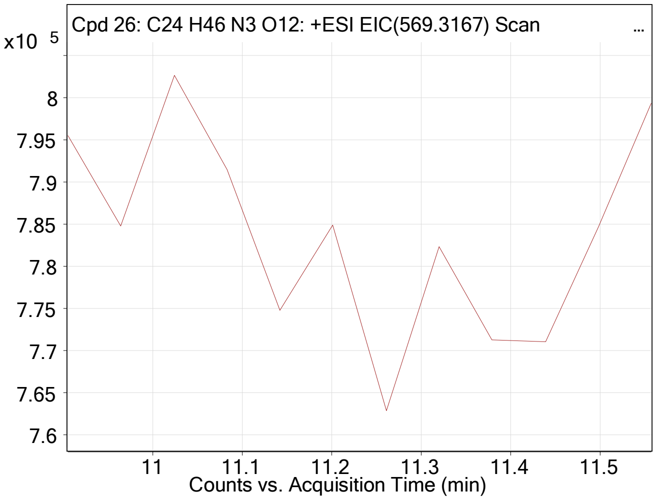

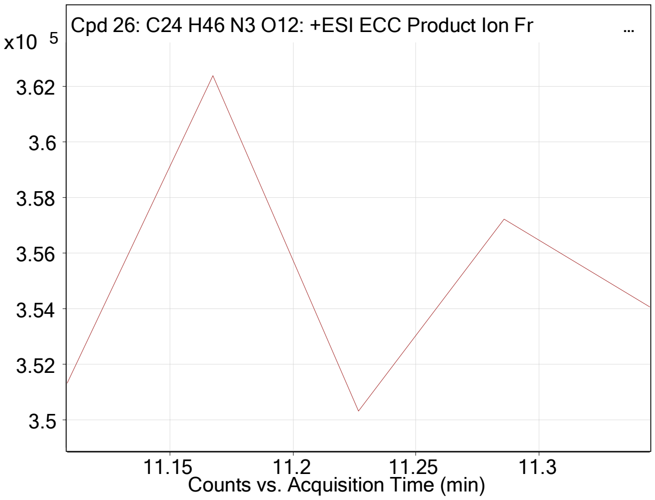

MS Spectrum

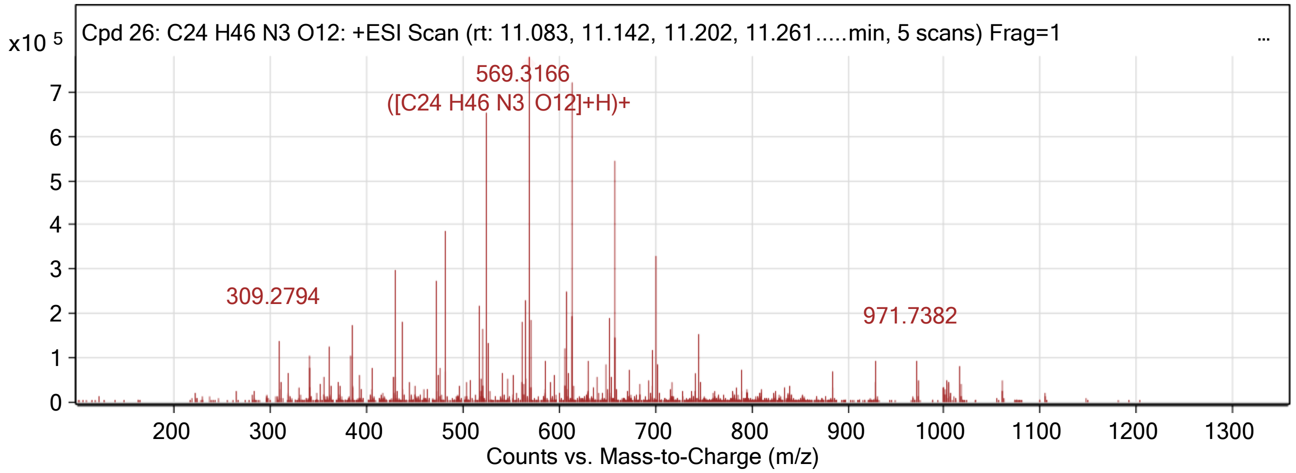

MS Zoomed Spectrum

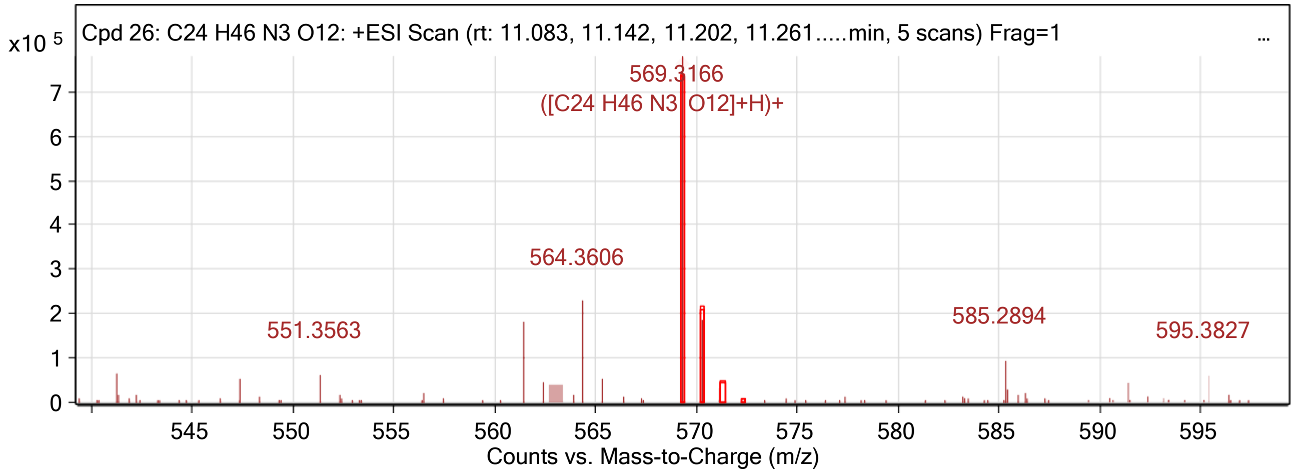

MS Spectrum Peak List

m/z	Calc *m/z*	Diff(ppm)	z	Abund	Formula	Ion
429.3199			1	296346		
481.2633			1	386939.63		
525.2901			1	649624.63		
569.3166	569.3154	-2.15	1	779273.63	C24 H46 N3 O12	(M+H)+
570.3192	570.3186	-1	1	185373.28	C24 H46 N3 O12	(M+H)+
571.3212	571.3209	-0.36	1	31541.94	C24 H46 N3 O12	(M+H)+
572.3239	572.3235	-0.65	1	4477.37	C24 H46 N3 O12	(M+H)+
613.3432			1	719369.75		
657.3691			1	542814.75		
701.3952			1	328484.91		

MSMS Spectrum

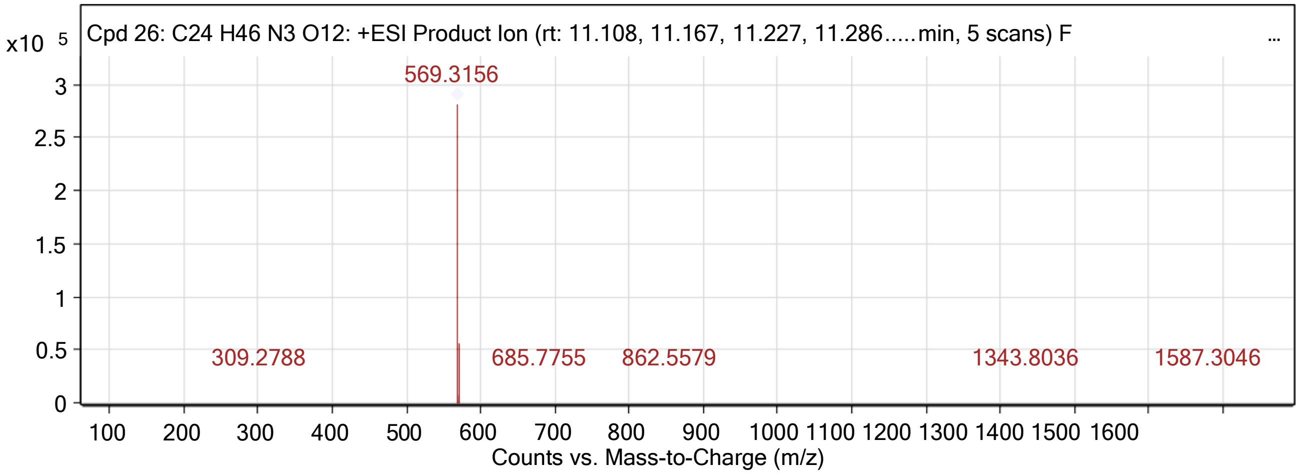

MS/MS Spectrum Peak List

m/z	*Calc m/z*	Diff(ppm)	z	Abund
265.2543	265.2526	-6.55		16.32
283.2604	283.2618	4.93		25.62
307.217	307.2142	-9.08		14.11
309.2788	309.2788	0.09		41.01
311.2951	311.2945	-1.96		12.83
312.2988	312.3009	6.97		16.92
407.3022	407.303	1.96		12.84
425.2702	425.2732	6.92		14.94
568.3006	284.1535	-499994.28	2	515.75
569.3156	569.3154	-0.39	1	281042.03

Compound Label	*m/z*	RT	Algorithm	Mass
Cpd 27: C26 H48 O13	569.317	11.523	Auto MS/MS	568.3096

Compound Chromatograms

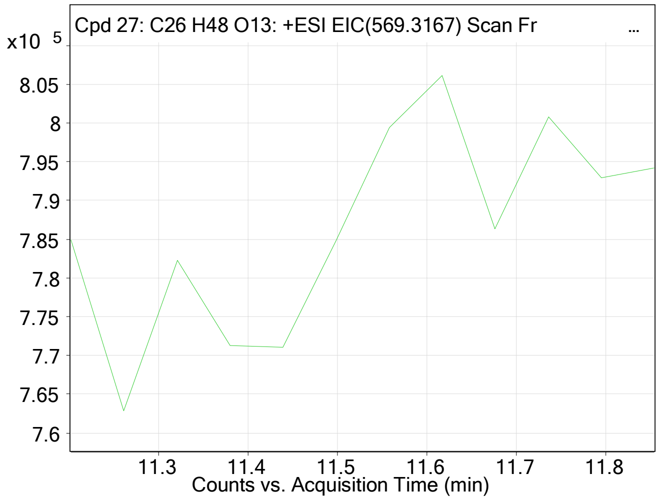

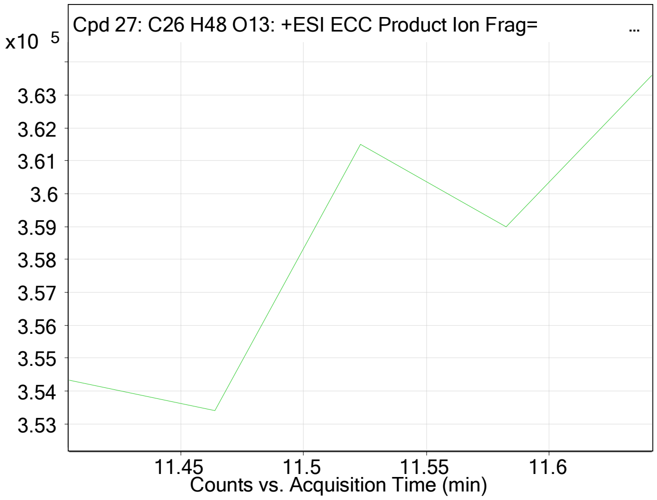

MS Spectrum

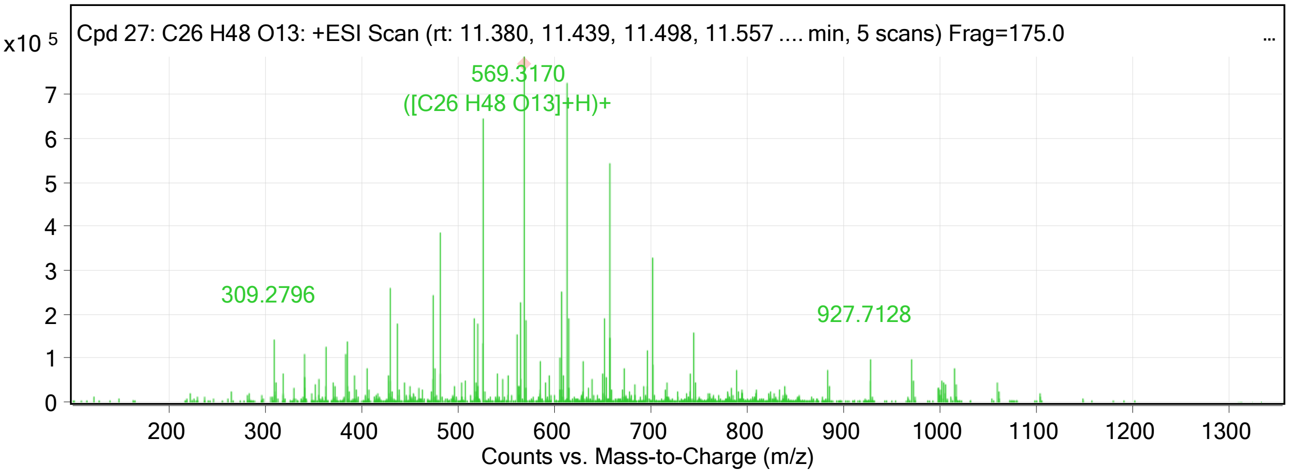

MS Zoomed Spectrum

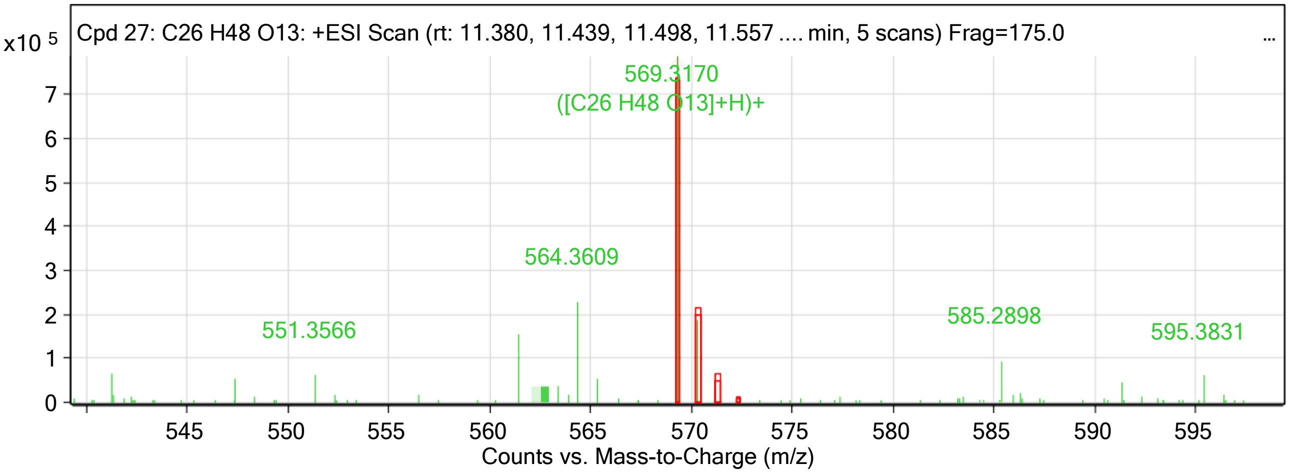

MS Spectrum Peak List

m/z	Calc *m/z*	Diff(ppm)	z	Abund	Formula	Ion
429.3201			1	257915.08		
481.2636			1	384128.44		
525.2906			1	644422.69		
569.317	569.3168	-0.49	1	786512.19	C26 H48 O13	(M+H)+
570.3196	570.3202	1.07	1	186662.63	C26 H48 O13	(M+H)+
571.3216	571.3226	1.82	1	31462.27	C26 H48 O13	(M+H)+
572.3243	572.3253	1.73	1	4529.78	C26 H48 O13	(M+H)+
613.3436			1	726811.81		
657.3695			1	543504.5		
701.3956			1	330184.31		

MSMS Spectrum

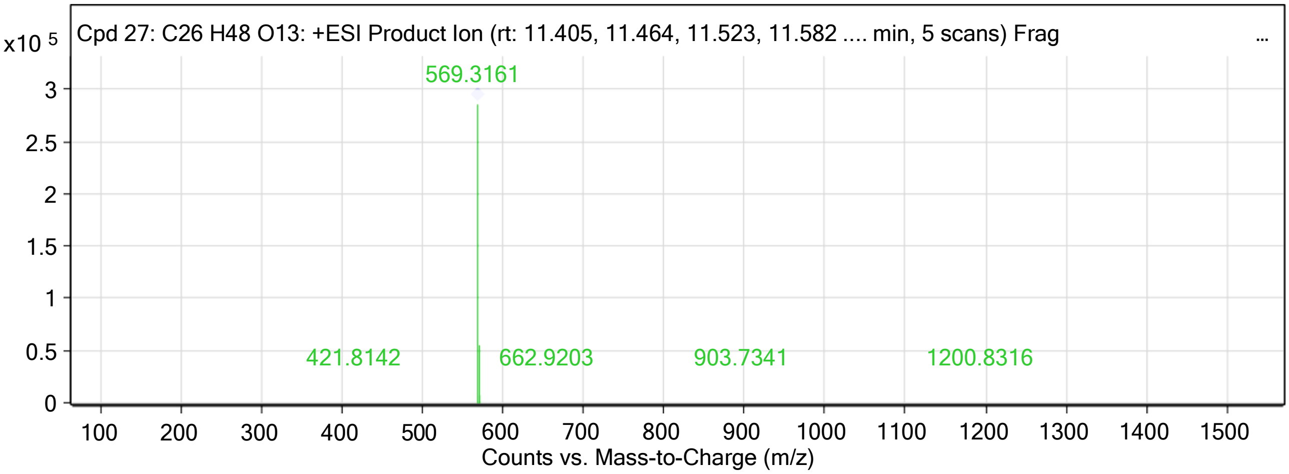

MS/MS Spectrum Peak List

m/z	Calc *m/z*	Diff(ppm)	z	Abund
101.0595	101.0597	2.02		13.36
109.1005	109.1012	6.21		14.78
111.082	111.0804	-14.03		12.14
133.0874	133.0859	-10.75		50.29
188.103	188.1043	6.87		11.21
283.2645	283.2632	-4.76		12.64
309.2768	309.2788	6.37		19.5
538.2895	269.1489	-499992.26	2	11
539.3059			2	14.57
569.3161	569.3168	1.16	1	285353.16

Compound Label	*m/z*	RT	Algorithm	Mass
Cpd 28: C26 H48 O13	569.3167	11.82	Auto MS/MS	568.3093

Compound Chromatograms

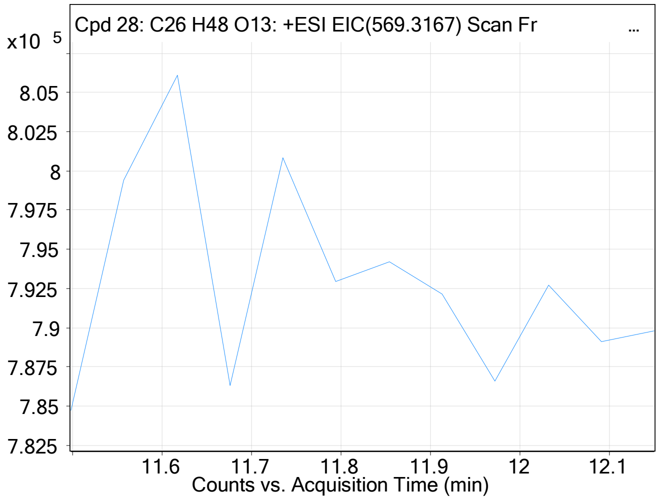

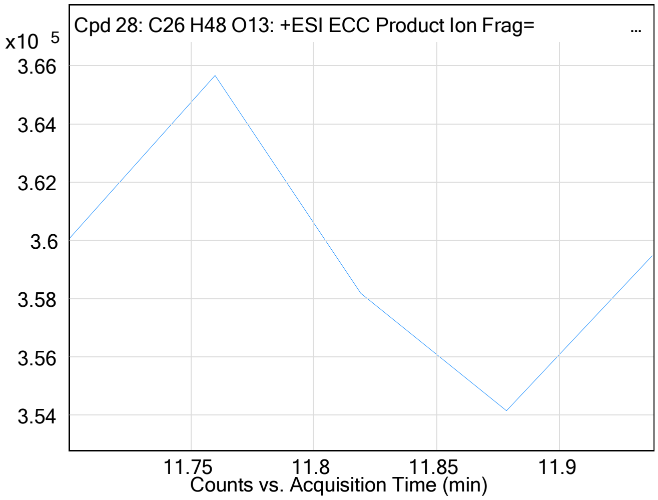

MS Spectrum

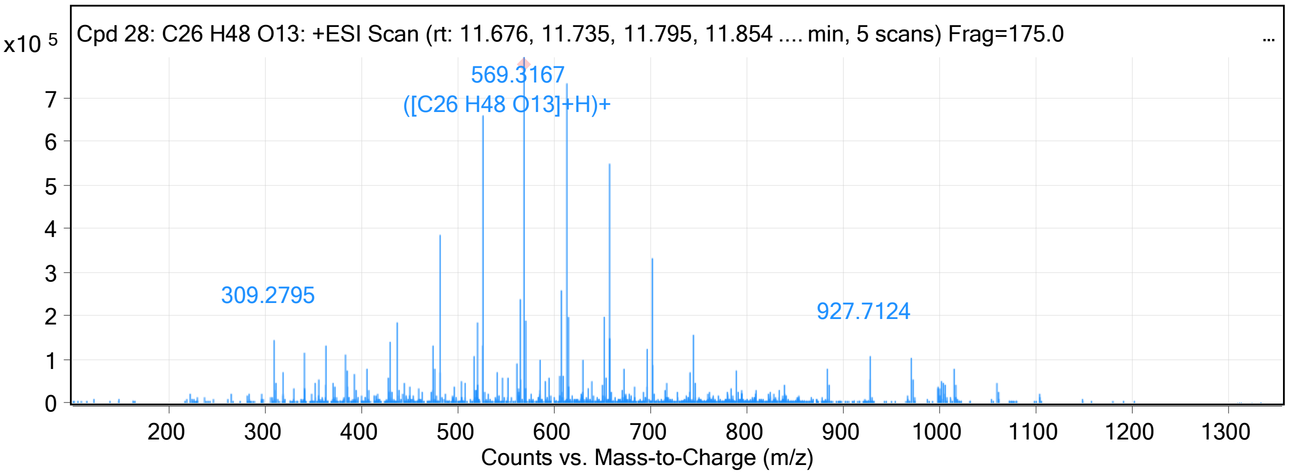

MS Zoomed Spectrum

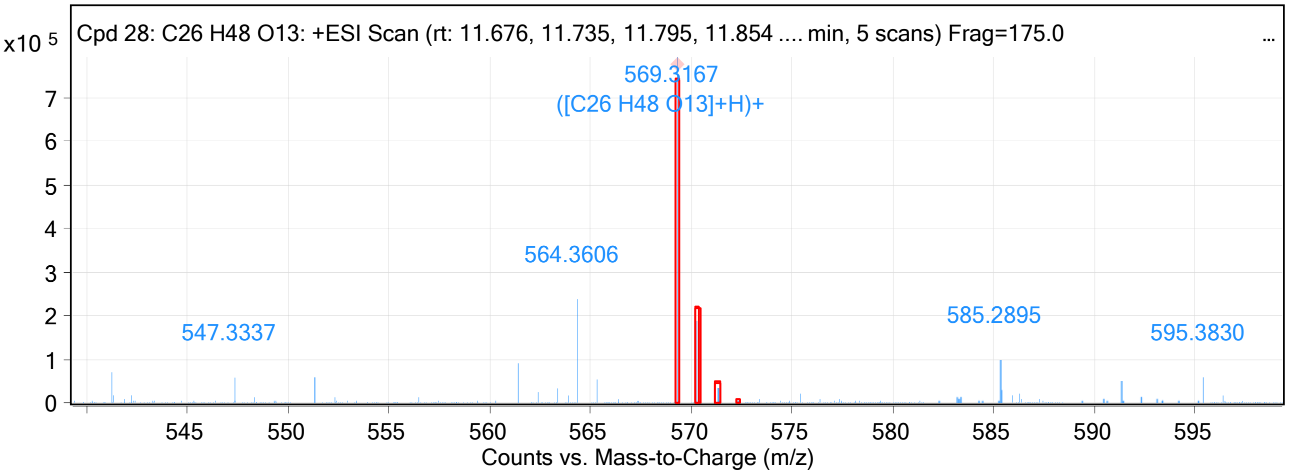

MS Spectrum Peak List

m/z	Calc *m/z*	Diff(ppm)	z	Abund	Formula	Ion
481.2633			1	385756.25		
525.2902			1	657172.19		
569.3167	569.3168	0.06	1	793300.38	C26 H48 O13	(M+H)+
570.3193	570.3202	1.58	1	187327.77	C26 H48 O13	(M+H)+
571.3215	571.3226	1.97	1	31827.95	C26 H48 O13	(M+H)+
572.3243	572.3253	1.74	1	4733.86	C26 H48 O13	(M+H)+
608.3871			1	258926.13		
613.3432			1	732383.06		

MSMS Spectrum

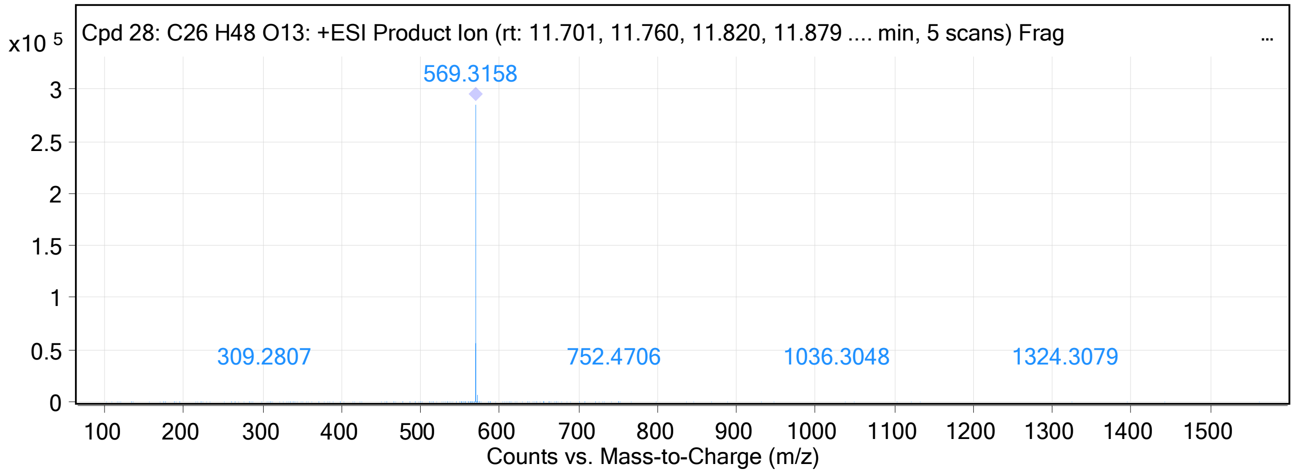

MS/MS Spectrum Peak List

m/z	*Calc m/z*	Diff(ppm)	z	Abund
109.1009	109.1012	2.6		12.5
117.0908	117.091	1.5		14.6
133.0865	133.0859	-4.59		25.79
175.1317	175.1329	6.86		22.92
177.1124	177.1121	-1.66		21.8
221.1375	221.1384	3.96		23.07
307.2195	307.2268	23.58		14.45
309.2807	309.2788	-6.23		51.96
385.206	385.2068	2.21		11.2
569.3158	569.3168	1.73	1	285414.25

Compound Label	*m/z*	RT	Algorithm	Mass
Cpd 29: C26 H48 O13	569.317	12.116	Auto MS/MS	568.3095

Compound Chromatograms

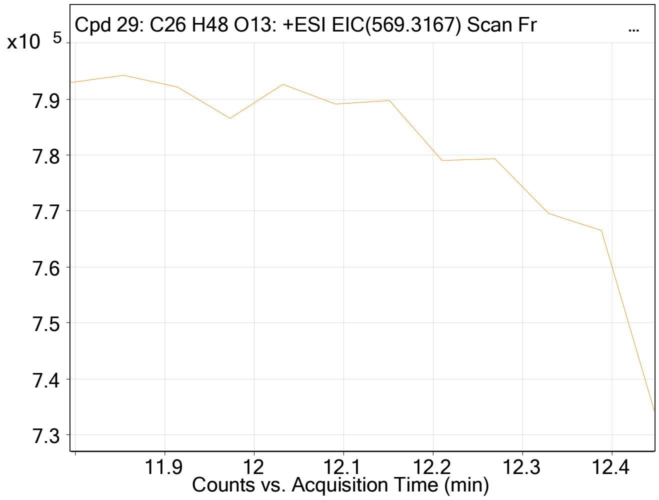

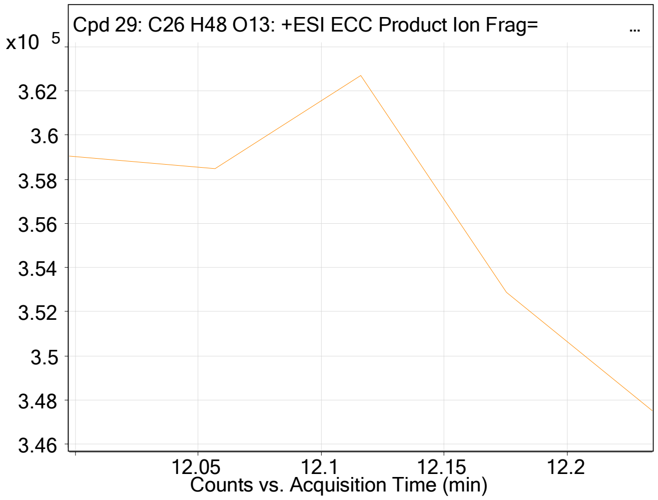

MS Spectrum

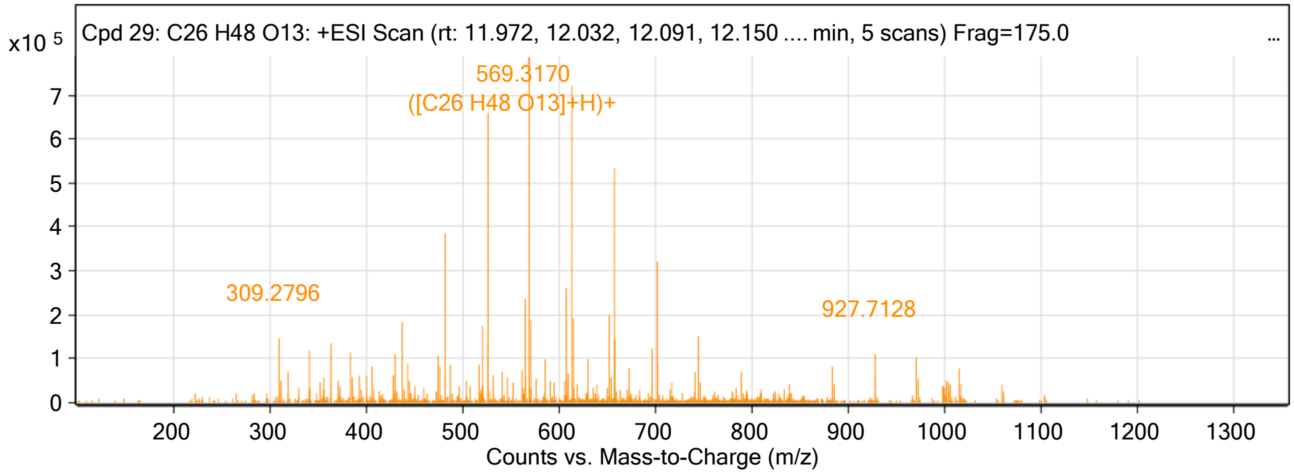

MS Zoomed Spectrum

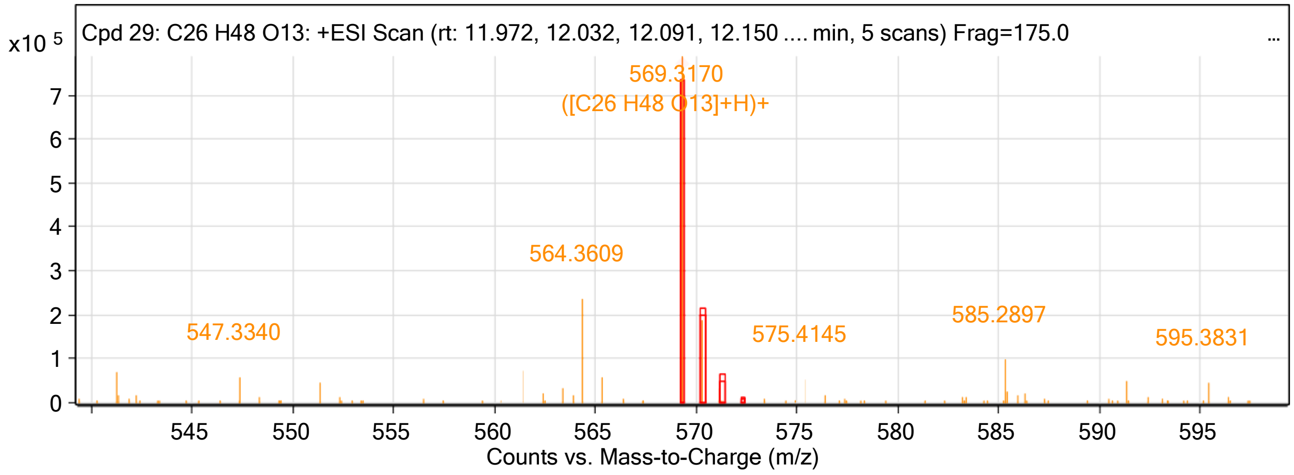

MS Spectrum Peak List

m/z	Calc *m/z*	Diff(ppm)	z	Abund	Formula	Ion
481.2636			1	386061.22		
525.2903			1	658030.38		
569.317	569.3168	-0.39	1	787438.13	C26 H48 O13	(M+H)+
570.3195	570.3202	1.17	1	186186.34	C26 H48 O13	(M+H)+
571.3217	571.3226	1.59	1	31113.96	C26 H48 O13	(M+H)+
572.3246	572.3253	1.23	1	4305.29	C26 H48 O13	(M+H)+
608.3874			1	258804.25		
613.3435			1	716779.38		
657.3695			1	533896.13		
701.3956			1	320209.78		

MSMS Spectrum

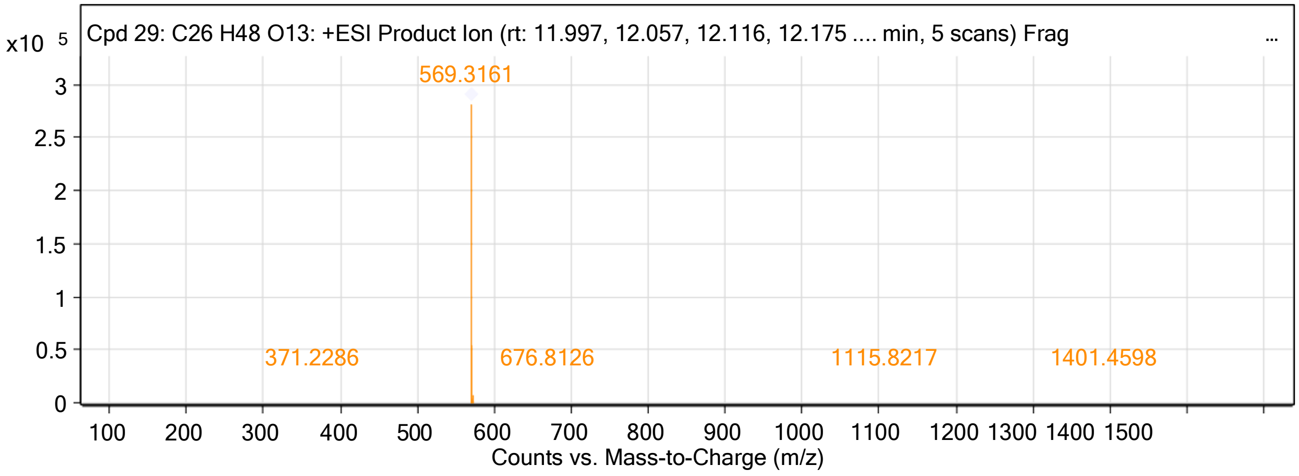

MS/MS Spectrum Peak List

m/z	*Calc m/z*	Diff(ppm)	z	Abund
102.0665	102.0675	9.97		13.63
133.0854	133.0859	3.81	1	65.15
177.1125	177.1121	-1.84		35.72
261.1324	261.1333	3.32		11.86
309.2796	309.2788	-2.66		15.97
311.2984	311.2945	-12.52		12.94
404.2419	404.2405	-3.65		12.54
539.3054	269.6528	-499999.76	2	19.22
567.2926	283.6503	-499993	2	18.99
569.3161	569.3168	1.18	1	281254.31

Compound Label	*m/z*	RT	Algorithm	Mass
Cpd 30: C26 H48 O13	569.3168	12.413	Auto MS/MS	568.3093

Compound Chromatograms

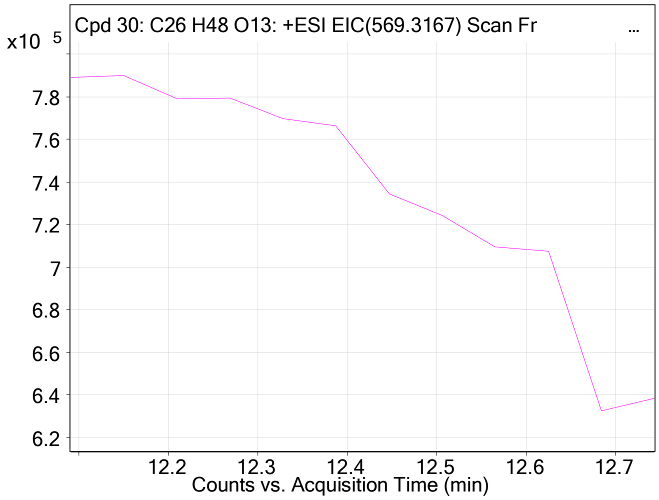

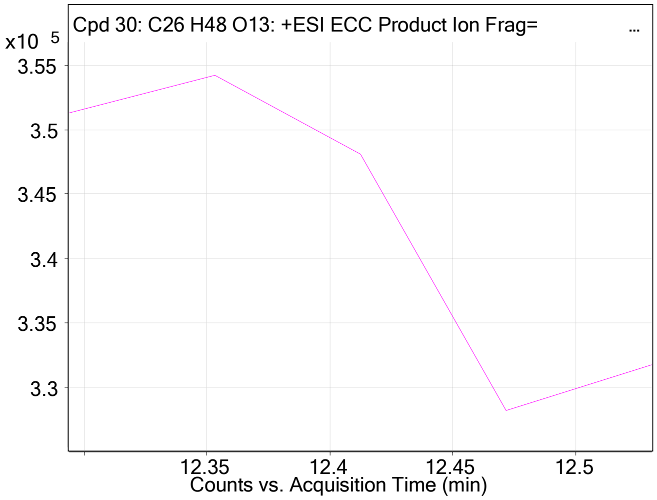

MS Spectrum

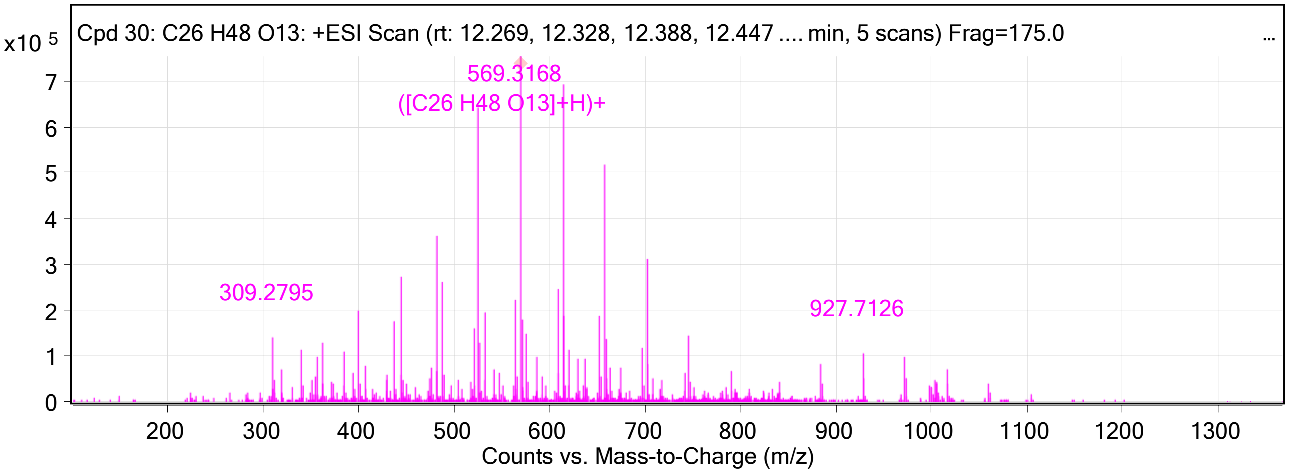

MS Zoomed Spectrum

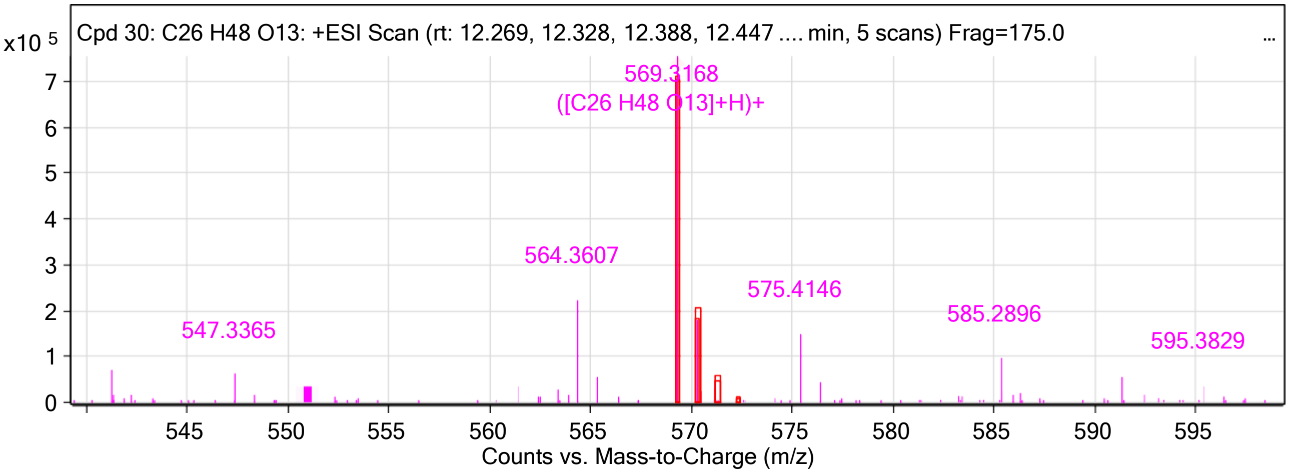

MS Spectrum Peak List

m/z	Calc *m/z*	Diff(ppm)	z	Abund	Formula	Ion
443.3356			1	271377.5		
481.2634			1	363371.97		
525.2901			1	641077.75		
569.3168	569.3168	-0.06	1	754825.5	C26 H48 O13	(M+H)+
570.3193	570.3202	1.48	1	177304.19	C26 H48 O13	(M+H)+
571.3213	571.3226	2.2	1	29733.88	C26 H48 O13	(M+H)+
572.3242	572.3253	1.84	1	4491.78	C26 H48 O13	(M+H)+
613.3433			1	692214.81		
657.3692			1	518225.81		
701.3953			1	311619.81		

MSMS Spectrum

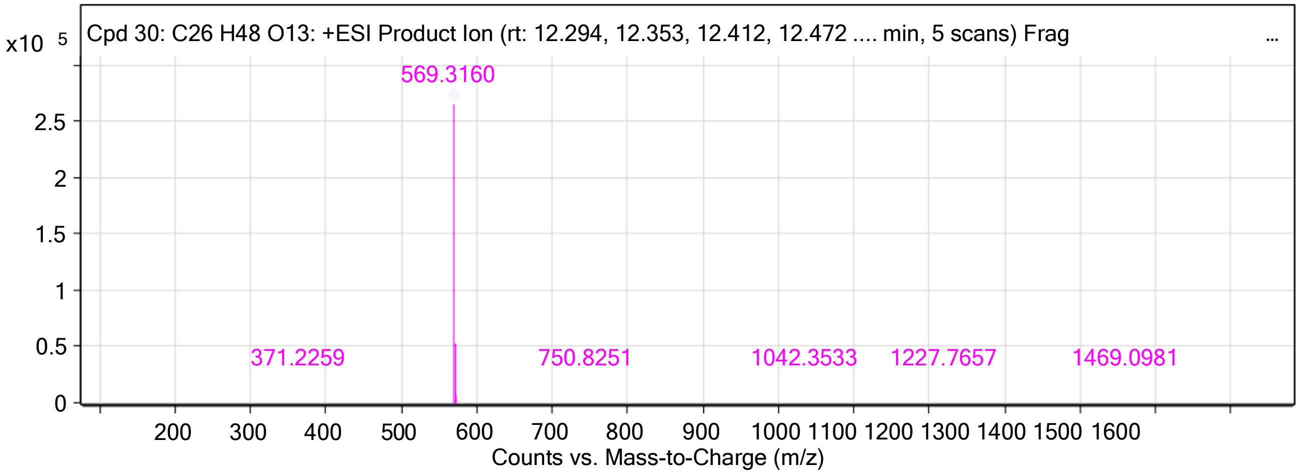

MS/MS Spectrum Peak List

m/z	Calc *m/z*	Diff(ppm)	z	Abund
123.0426	123.0441	11.82		22.05
127.1478	127.1481	2.82		13.67
133.0865	133.0859	-4.67	1	69.19
177.1112	177.1121	5.18		21.13
210.1197	210.125	25.47		17.6
228.2836	228.2812	-10.83		14.76
309.2771	309.2788	5.38		30.43
311.2958	311.2945	-4.17		25.43
444.2764	444.2718	-10.32		13.56
569.316	569.3168	1.43	1	264911.88

Compound Label	*m/z*	RT	Algorithm	Mass
Cpd 31: C25 H48 N3 O6	487.3625	12.777	Auto MS/MS	486.355

Compound Chromatograms

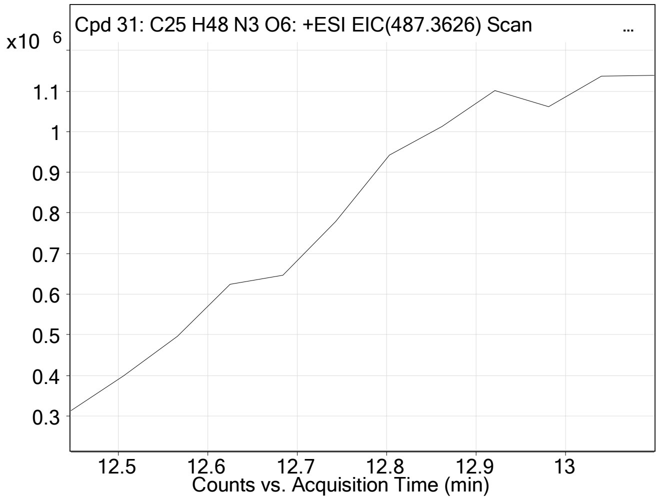

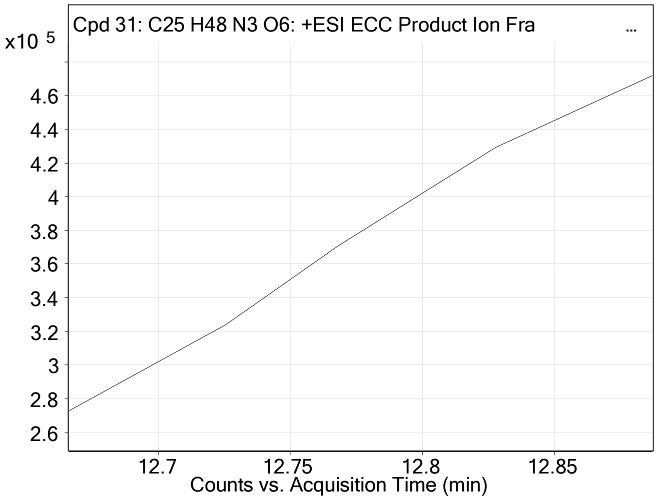

MS Spectrum

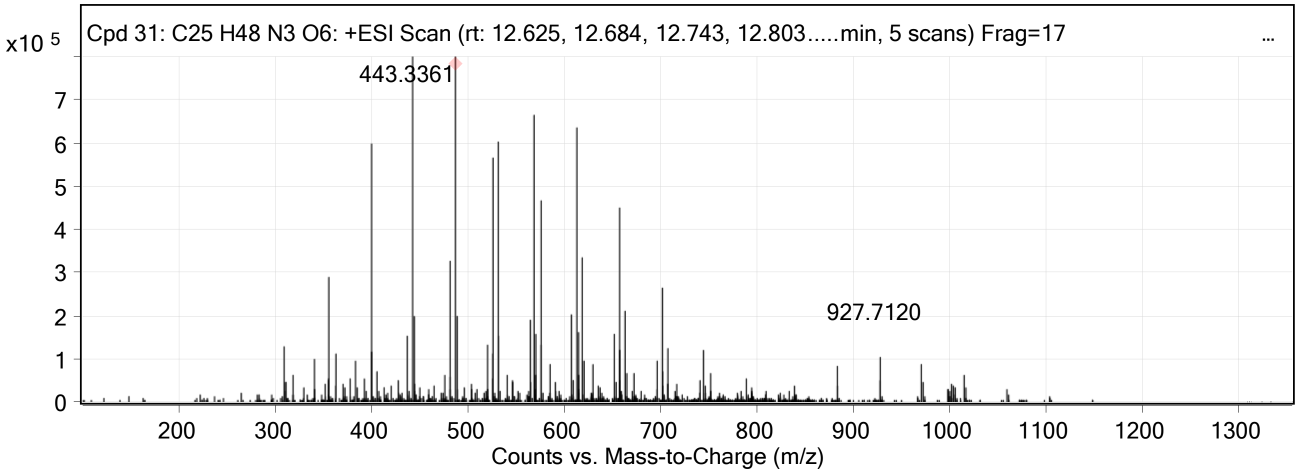

MS Zoomed Spectrum

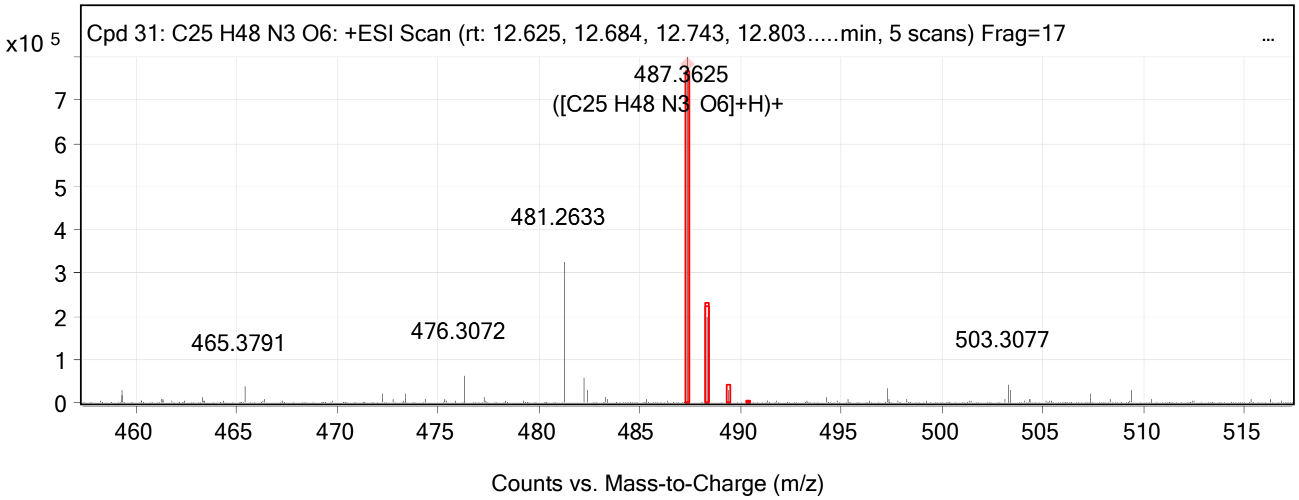

MS Spectrum Peak List

m/z	Calc *m/z*	Diff(ppm)	z	Abund	Formula	Ion
399.3095			1	599186.94		
443.3361			1	818220.13		
487.3625	487.3616	-1.8	1	801053.81	C25 H48 N3 O6	(M+H)+
488.3652	488.3648	-0.86	1	199442.89	C25 H48 N3 O6	(M+H)+
489.3668	489.3674	1.37	1	29107.23	C25 H48 N3 O6	(M+H)+
490.3682	490.3701	3.71	1	3896.41	C25 H48 N3 O6	(M+H)+
525.2899			1	564760.81		
531.3886			1	603833.94		

MSMS Spectrum

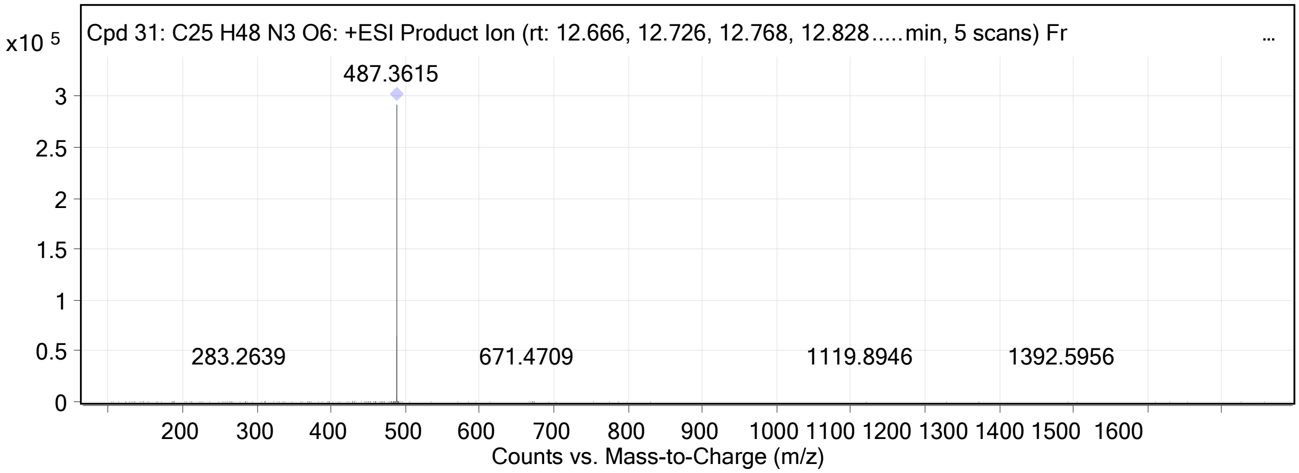

MS/MS Spectrum Peak List

m/z	*Calc m/z*	Diff(ppm)	z	Abund
133.0874	133.0886	9.18		29.05
135.1177	135.1168	-6.72		17.18
166.0881	166.0863	-10.86		25.67
265.2539	265.2526	-4.92		19.34
283.2639	283.2632	-2.56	1	219.45
309.2781	309.2775	-1.9		53.92
311.2906	311.2931	8.1		21.92
397.2629	397.2585	-11.18		47.42
471.3452	471.3429	-4.9		22.43
487.3615	487.3616	0.11	1	292393.56

Compound Label	*m/z*	RT	Algorithm	Mass
Cpd 32: C23 H44 N3 O5	443.3363	12.806	Auto MS/MS	442.3289

Compound Chromatograms

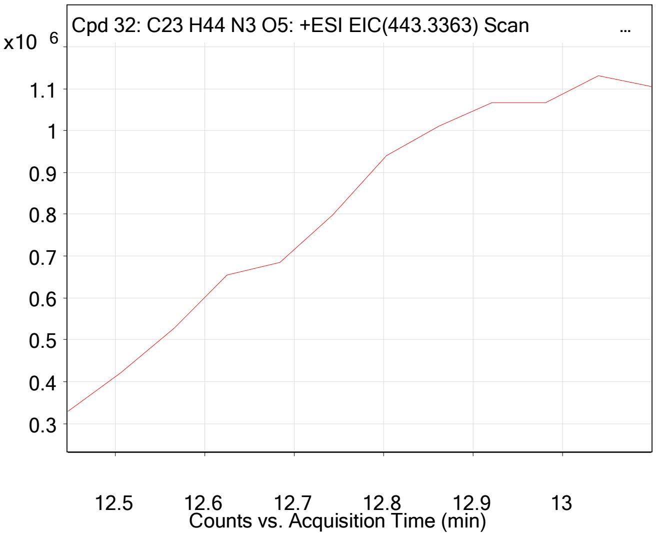

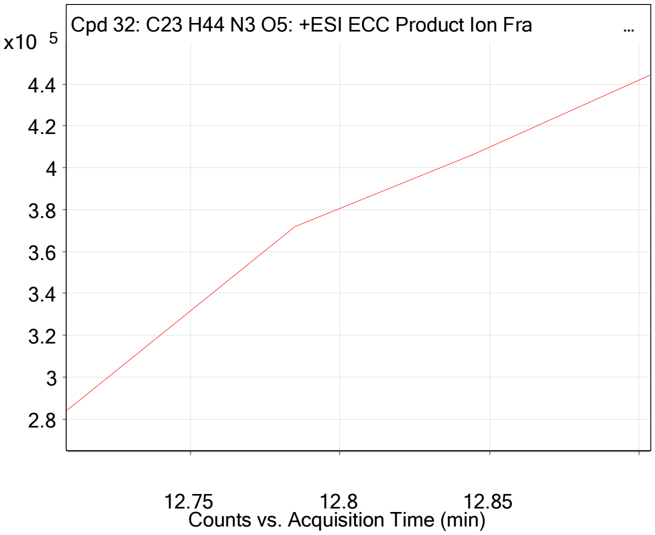

MS Spectrum

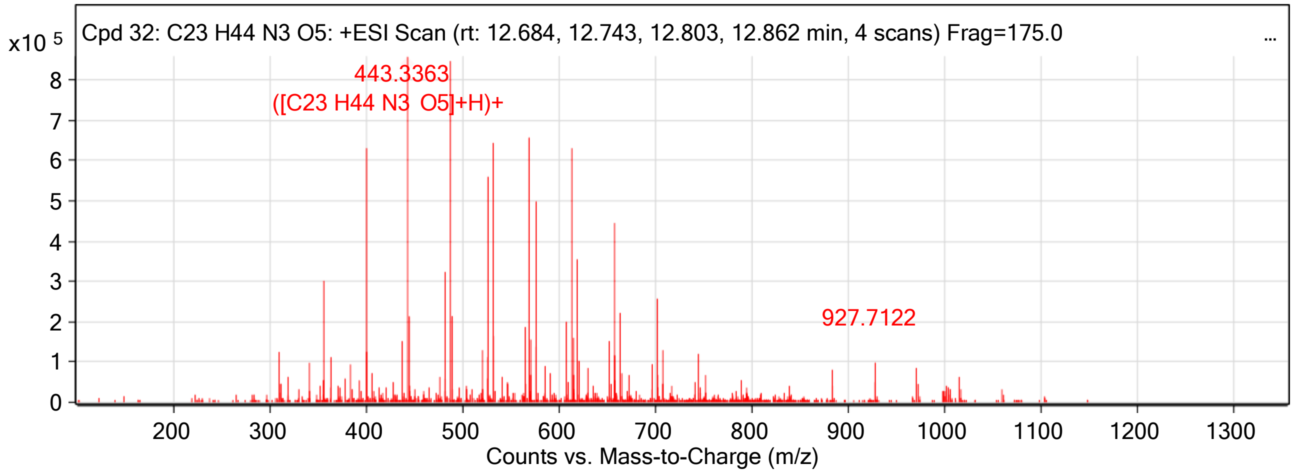

MS Zoomed Spectrum

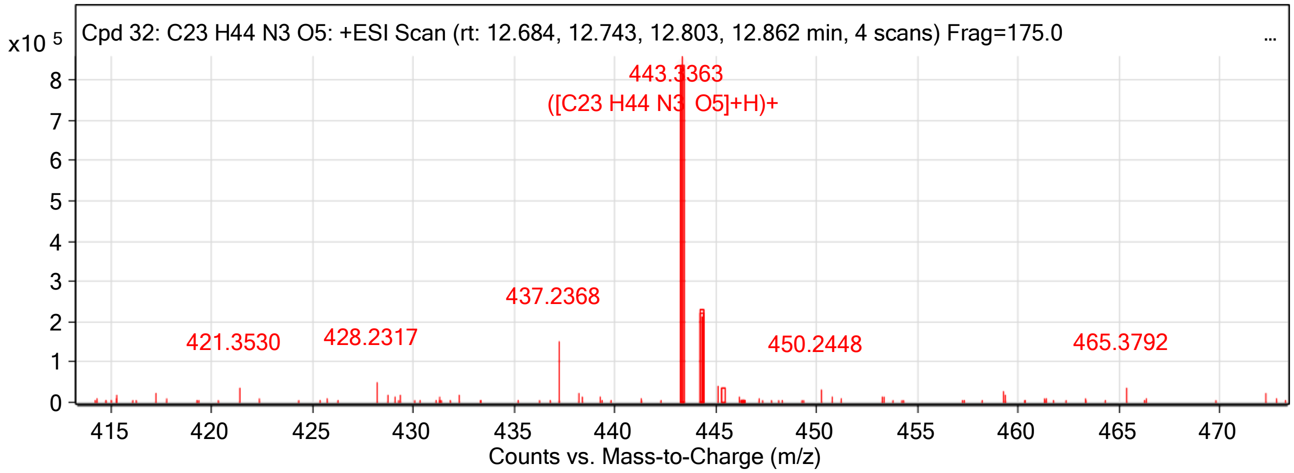

MS Spectrum Peak List

m/z	Calc *m/z*	Diff(ppm)	z	Abund	Formula	Ion
399.3096			1	629952.81		
443.3363	443.3354	-2.05	1	858966.88	C23 H44 N3 O5	(M+H)+
444.339	444.3385	-0.95	1	210670.41	C23 H44 N3 O5	(M+H)+
445.3407	445.3412	1.11	1	27400.72	C23 H44 N3 O5	(M+H)+
446.3426	446.3438	2.69	1	3261.69	C23 H44 N3 O5	(M+H)+
487.3626			1	845414.63		
525.29			1	556173.69		
531.3888			1	640274.94		
569.3167			1	654377.38		
613.3428			1	629430.5		

MSMS Spectrum

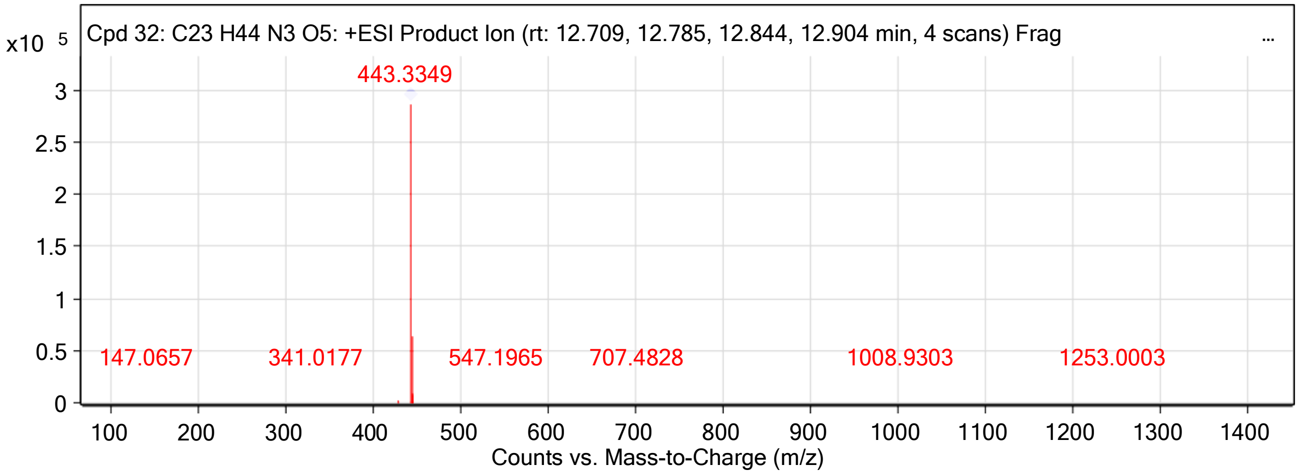

MS/MS Spectrum Peak List

m/z	*Calc m/z*	Diff(ppm)	z	Abund
117.0909	117.091	0.68		16.4
142.0972	142.0975	2.22		28.51
147.0657	147.0652	-3.7		52.16
177.1664	177.1638	-14.99		17.25
181.0864	181.0859	-2.55		16.65
191.1788	191.1794	3.3		33.54
309.2788	309.2788	0.09	1	78.13
315.1727	315.1789	19.53		16.39
442.322	442.3275	12.49		205.19
443.3349	443.3354	0.97	1	285968.34

Compound Label	*m/z*	RT	Algorithm	Mass
Cpd 33: C25 H48 N3 O6	487.3625	13.065	Auto MS/MS	486.3551

Compound Chromatograms

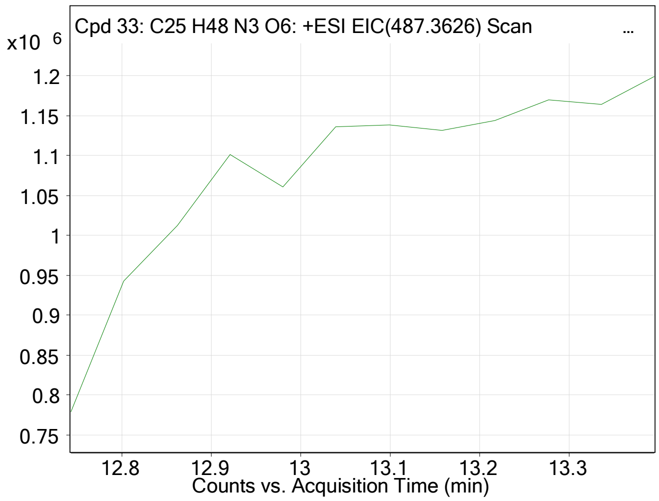

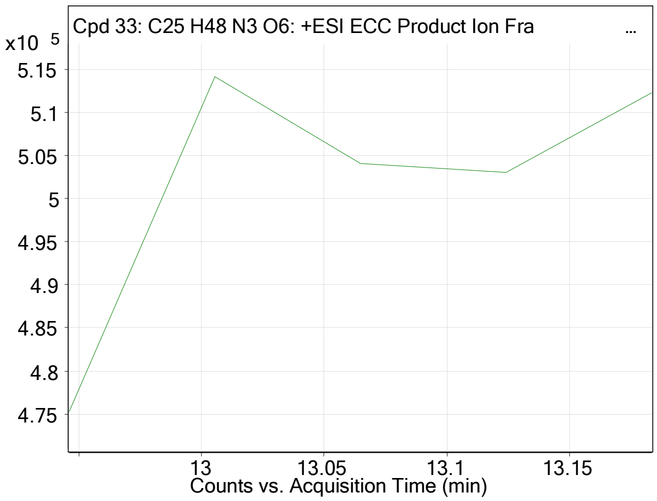

MS Spectrum

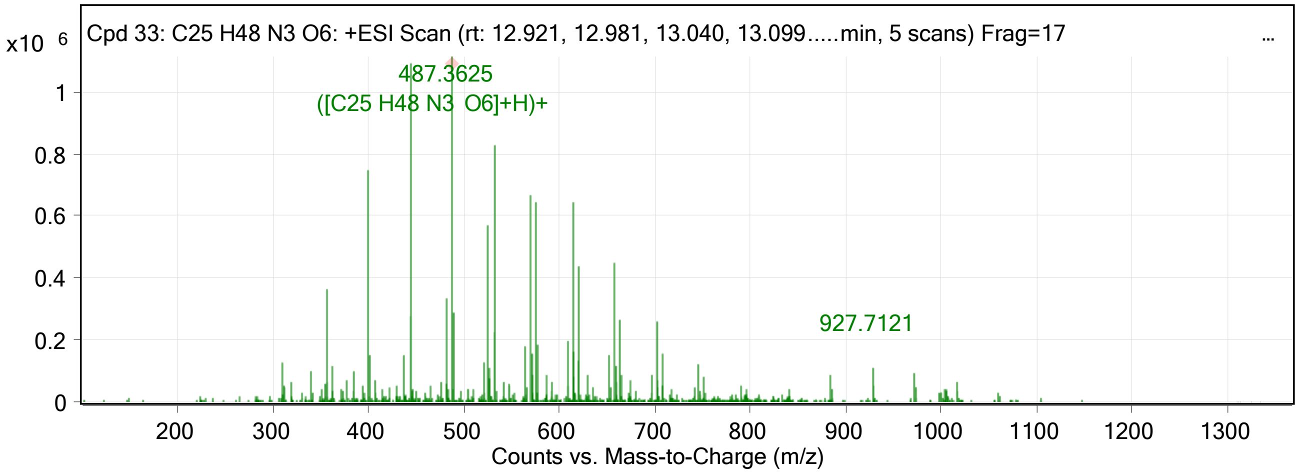

MS Zoomed Spectrum

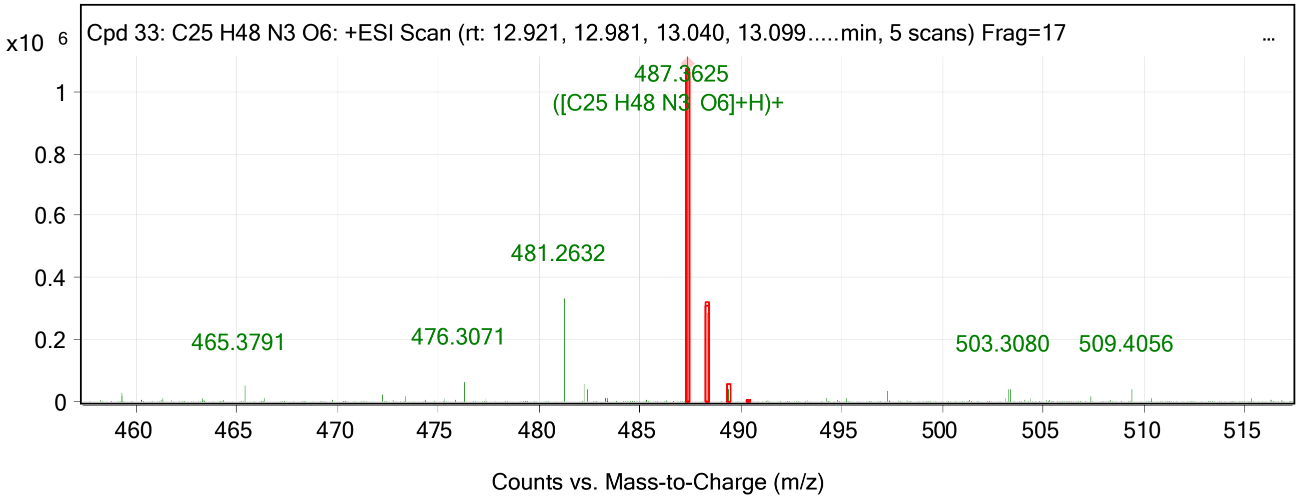

MS Spectrum Peak List

m/z	Calc *m/z*	Diff(ppm)	z	Abund	Formula	Ion
399.3096			1	745641.88		
443.3363			1	1091508		
487.3625	487.3616	-1.84	1	1113760.25	C25 H48 N3 O6	(M+H)+
488.3652	488.3648	-0.96	1	286090.41	C25 H48 N3 O6	(M+H)+
489.367	489.3674	0.88	1	41162.52	C25 H48 N3 O6	(M+H)+
490.3689	490.3701	2.36	1	5070.57	C25 H48 N3 O6	(M+H)+
531.389			1	827529.56		
569.3165			1	668916.19		
575.4148			1	643300.38		
613.3426			1	640658.88		

MSMS Spectrum

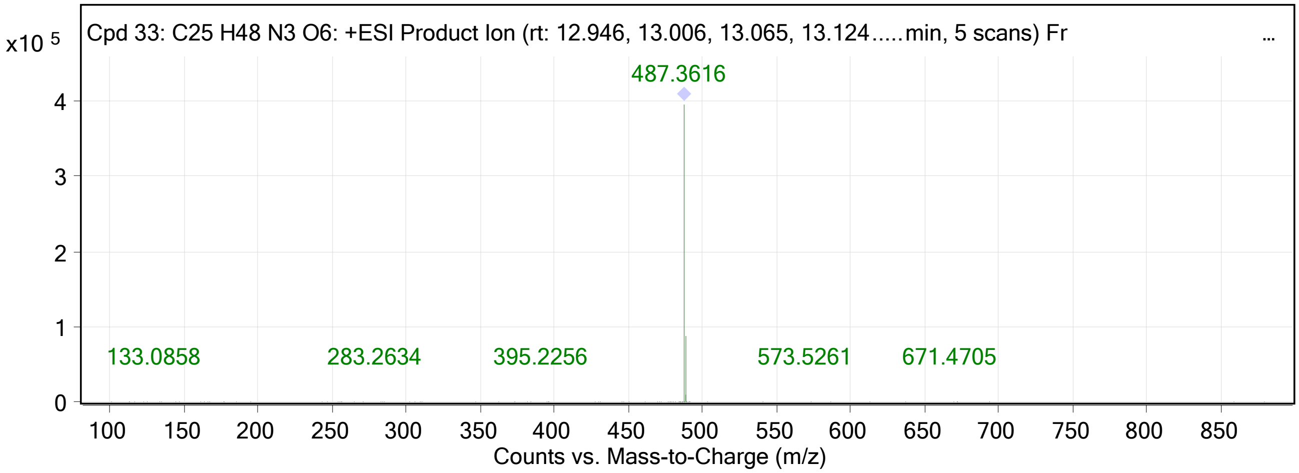

MS/MS Spectrum Peak List

m/z	Calc *m/z*	Diff(ppm)	z	Abund
133.0858	133.0859	1.06		30.29
283.2634	283.2632	-1.03	1	237.13
309.2781	309.2775	-2		89.84
486.3385			2	638.28
486.8311			2	418.49
487.3616	487.3616	-0.05	1	396278.94
488.2768			1	620.32
488.3643			1	87285.54
489.3187			2	576
489.3663			1	9552.65

Compound Label	*m/z*	RT	Algorithm	Mass
Cpd 34: C23 H44 N3 O5	443.3363	13.081	Auto MS/MS	442.3288

Compound Chromatograms

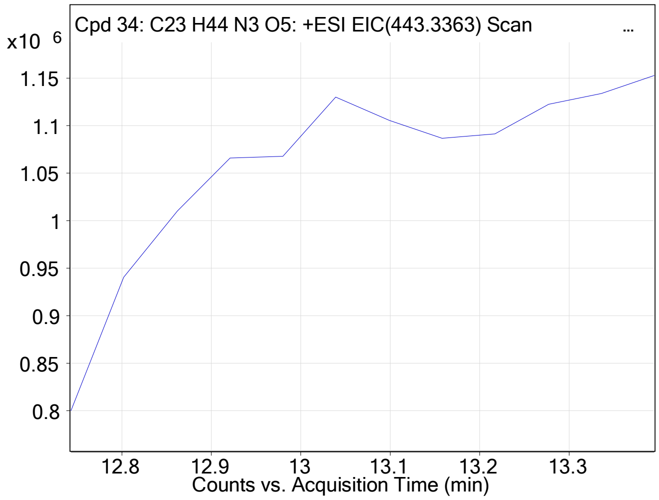

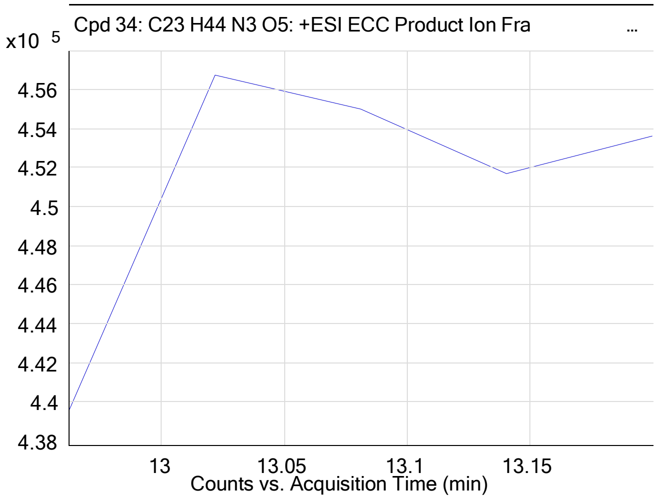

MS Spectrum

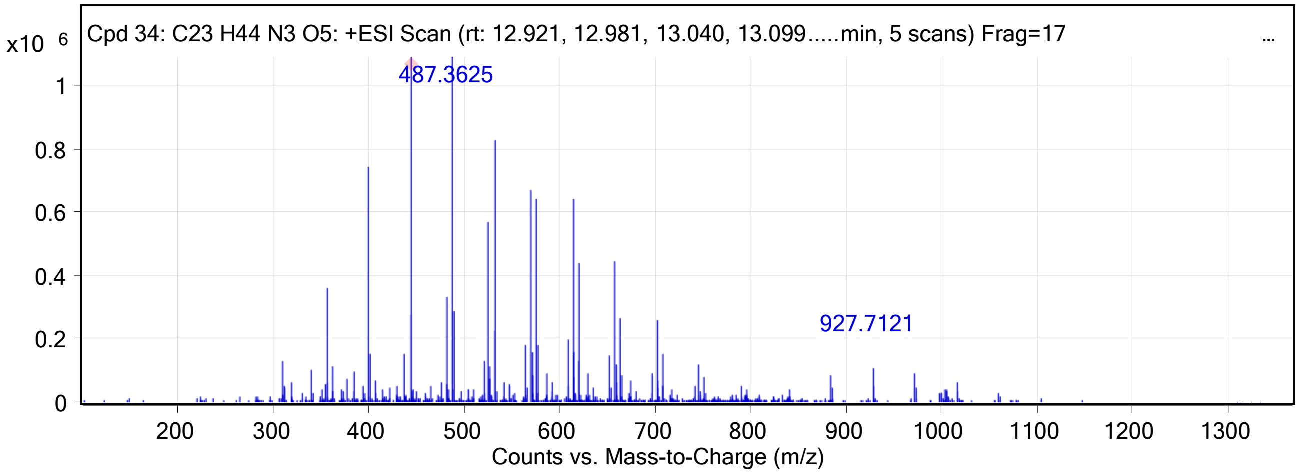

MS Zoomed Spectrum

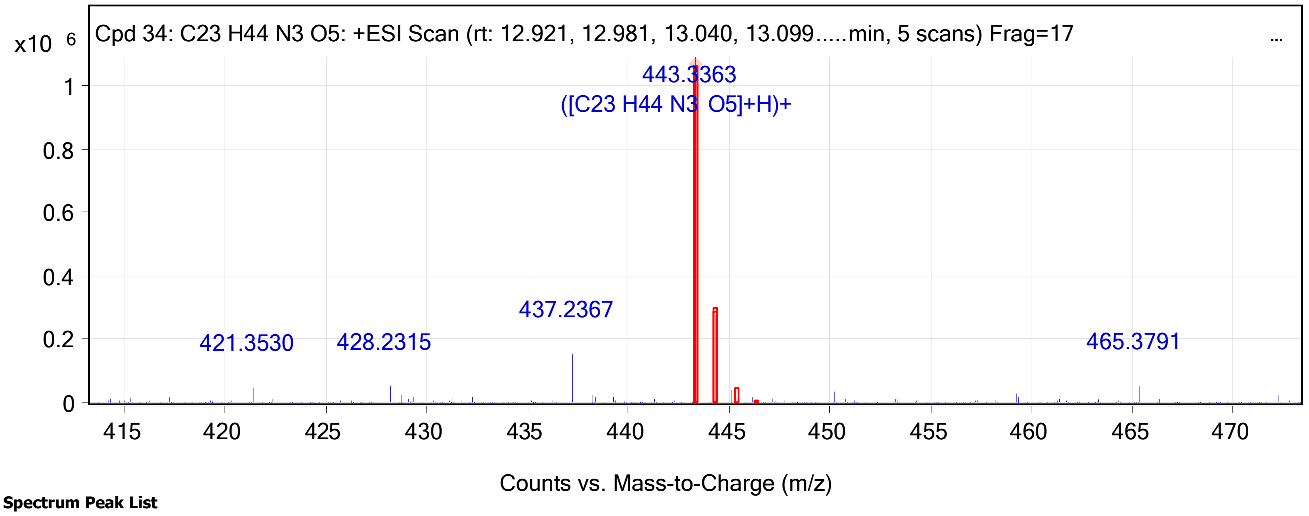

MS Spectrum Peak List

m/z	Calc *m/z*	Diff(ppm)	z	Abund	Formula	Ion
399.3096			1	745641.88		
443.3363	443.3354	-1.98	1	1091508	C23 H44 N3 O5	(M+H)+
444.339	444.3385	-0.96	1	273572.47	C23 H44 N3 O5	(M+H)+
445.3408	445.3412	0.99	1	34647.34	C23 H44 N3 O5	(M+H)+
446.343	446.3438	1.86	1	4030.78	C23 H44 N3 O5	(M+H)+
487.3625			1	1113760.25		
531.389			1	827529.56		
569.3165			1	668916.19		

MSMS Spectrum

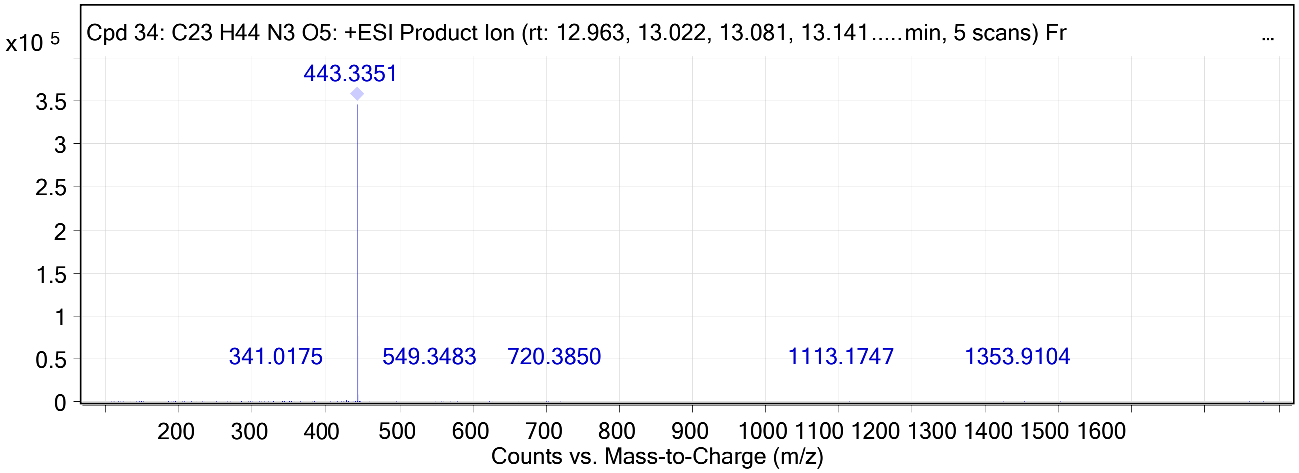

MS/MS Spectrum Peak List

m/z	*Calc m/z*	Diff(ppm)	z	Abund
119.0846	119.0855	8.18		13.3
133.0872	133.0859	-9.74		25.46
144.0754	144.0768	9.16		16.49
147.0659	147.0652	-4.73		95.47
149.134	149.1325	-10.19		14.04
283.2641	283.2632	-3.48		16.26
309.2783	309.2788	1.51		43.46
310.2792	310.2741	-16.6		21.13
311.2909	311.2931	7.08		21.74
443.3351	443.3354	0.68	1	346486.06

Compound Label	*m/z*	RT	Algorithm	Mass
Cpd 35: C25 H48 N3 O6	487.3625	13.361	Auto MS/MS	486.3551

Compound Chromatograms

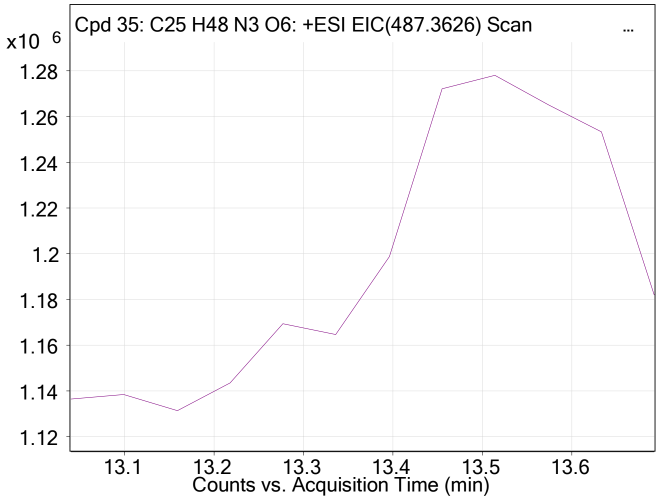

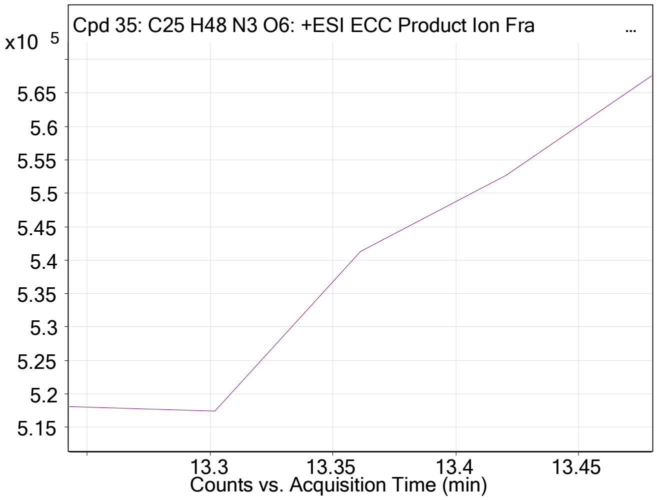

MS Spectrum

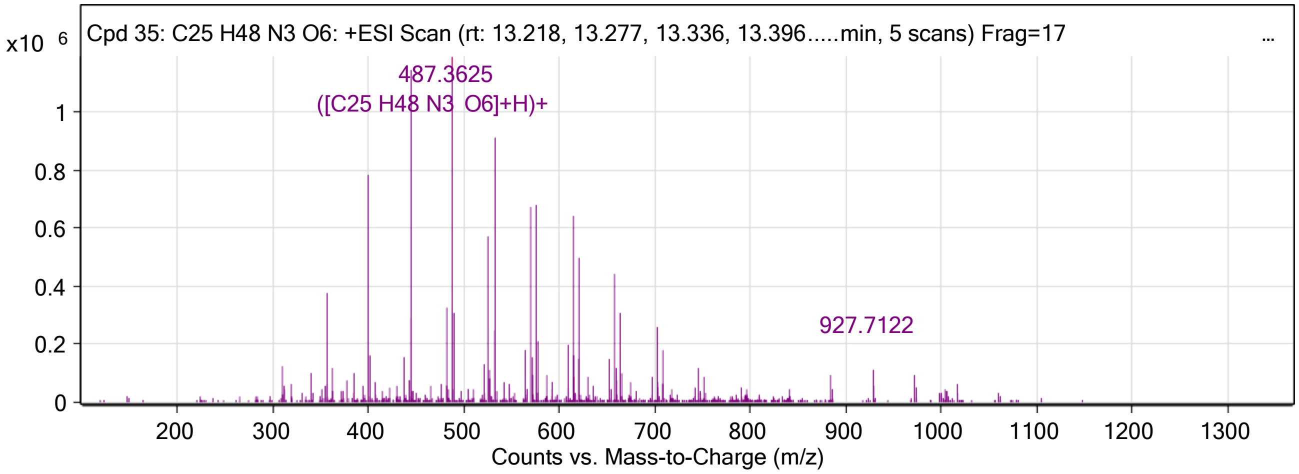

MS Zoomed Spectrum

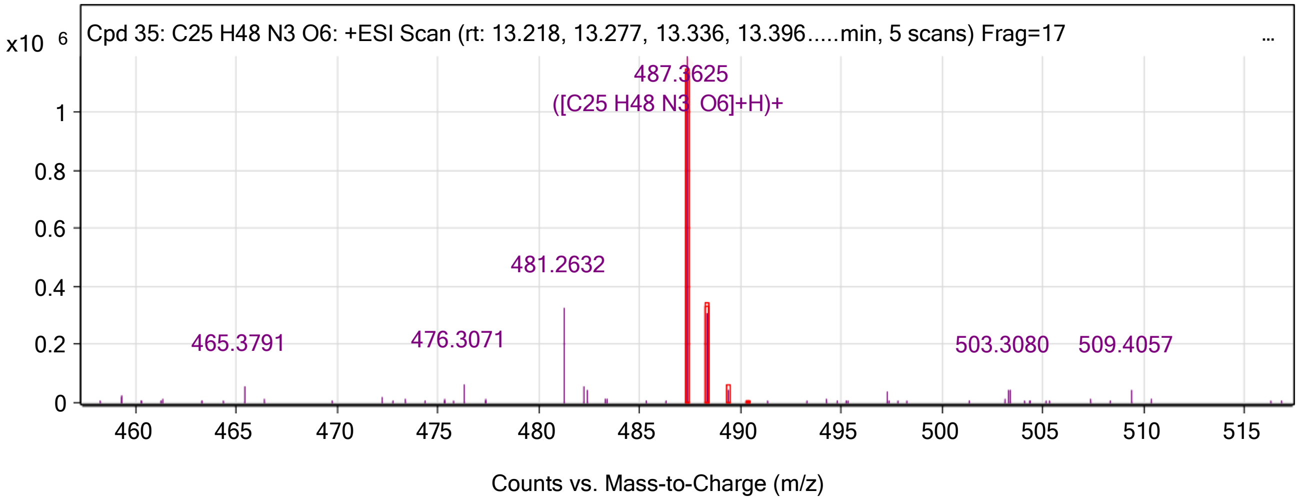

MS Spectrum Peak List

m/z	Calc *m/z*	Diff(ppm)	z	Abund	Formula	Ion
399.3097			1	782131.13		
443.3361			1	1142530.63		
487.3625	487.3616	-1.9	1	1189659.5	C25 H48 N3 O6	(M+H)+
488.3652	488.3648	-0.98	1	305906.44	C25 H48 N3 O6	(M+H)+
489.3671	489.3674	0.69	1	42169.64	C25 H48 N3 O6	(M+H)+
490.369	490.3701	2.25	1	5428.17	C25 H48 N3 O6	(M+H)+
531.3892			1	906770		
569.3165			1	668747.06		
575.4153			1	672120.31		
613.3425			1	638120.19		

MSMS Spectrum

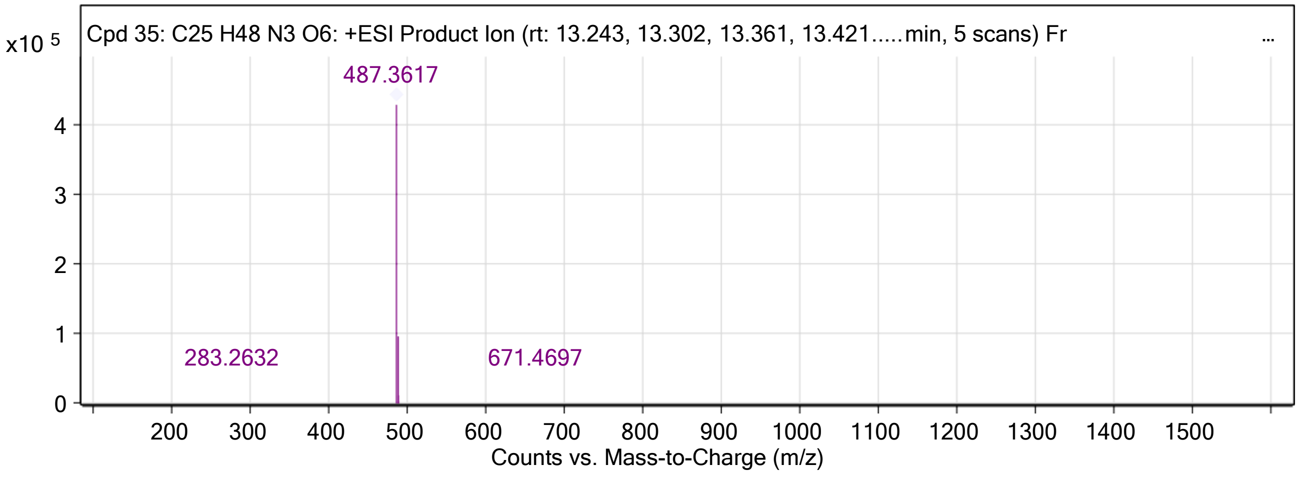

MS/MS Spectrum Peak List

m/z	*Calc m/z*	Diff(ppm)	z	Abund
283.2632	283.2632	-0.26	1	175.99
309.2785	309.2788	0.94		39.53
486.3351			2	590.86
486.6622			2	253.58
486.8297			2	452.98
487.3617	487.3616	-0.14	1	427468.41
488.3643			1	94058.11
489.318			2	436.91
489.3663			1	10103.49
490.3684			1	235.34

Compound Label	*m/z*	RT	Algorithm	Mass
Cpd 36: C23 H44 N3 O5	443.3361	13.378	Auto MS/MS	442.3288

Compound Chromatograms

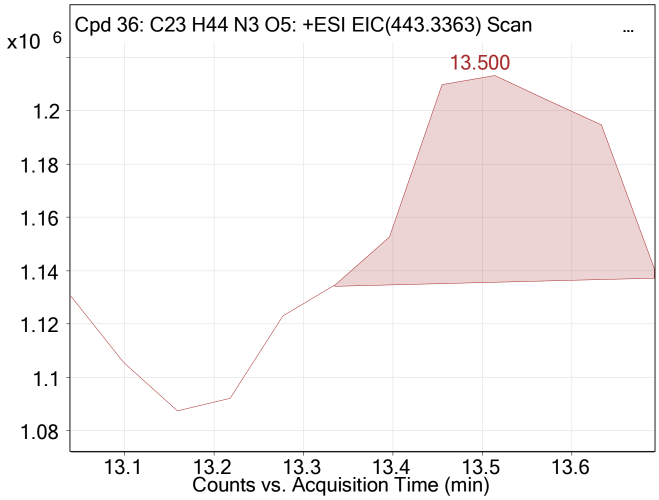

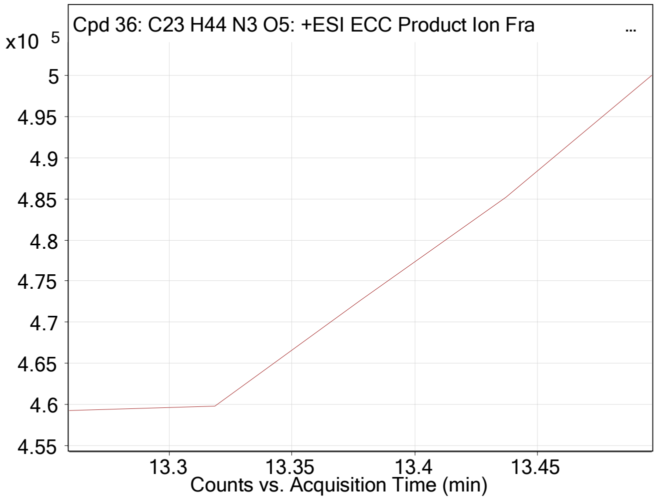

MS Spectrum

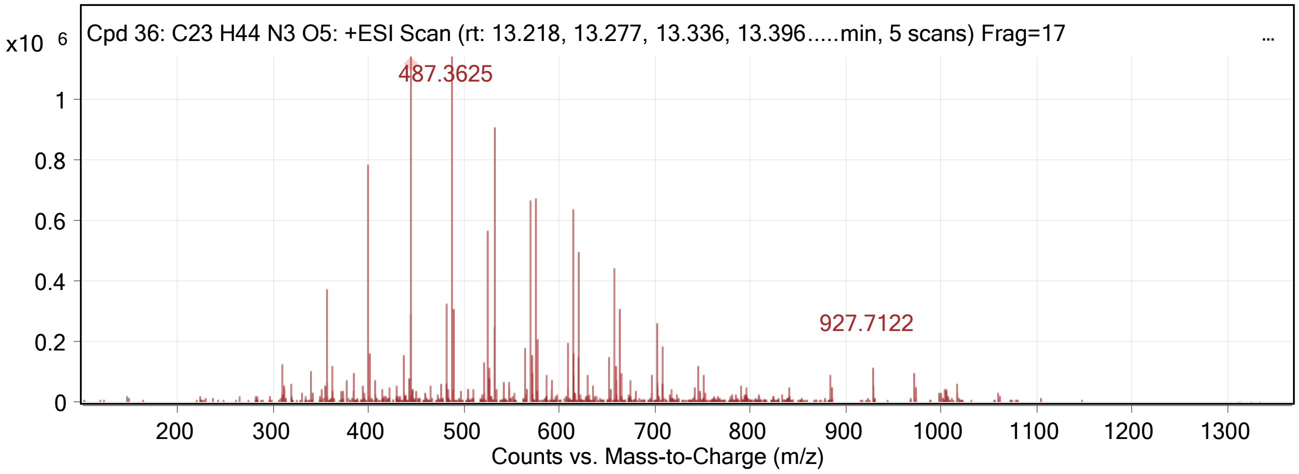

MS Zoomed Spectrum

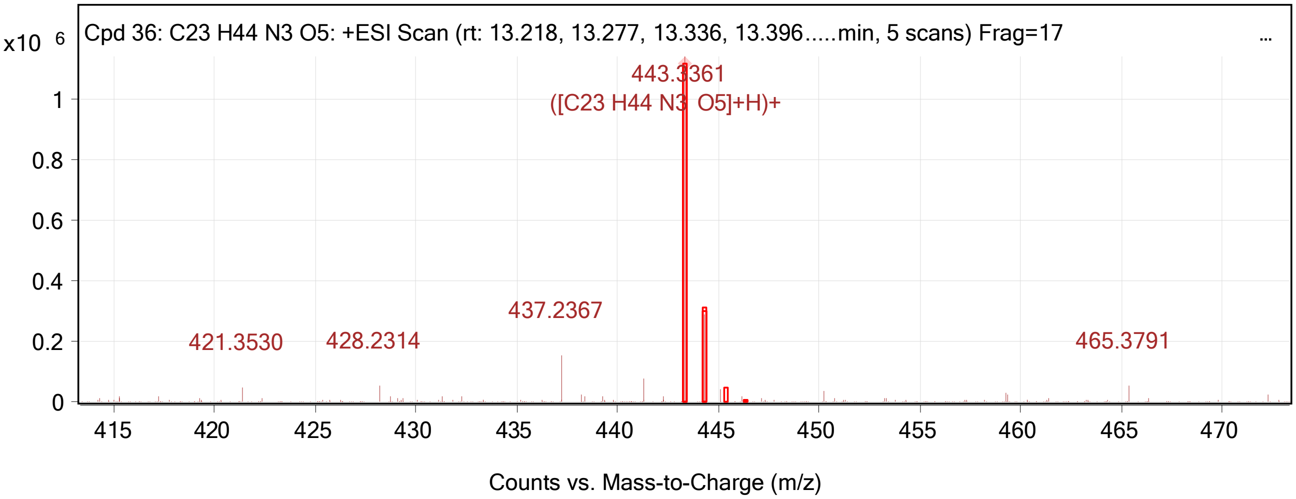

MS Spectrum Peak List

m/z	Calc *m/z*	Diff(ppm)	z	Abund	Formula	Ion
399.3097			1	782131.13		
443.3361	443.3354	-1.75	1	1142530.63	C23 H44 N3 O5	(M+H)+
444.3389	444.3385	-0.92	1	288213.31	C23 H44 N3 O5	(M+H)+
445.3408	445.3412	0.93	1	35657.84	C23 H44 N3 O5	(M+H)+
446.3433	446.3438	1.12	1	4242.09	C23 H44 N3 O5	(M+H)+
487.3625			1	1189659.5		
531.3892			1	906770		
569.3165			1	668747.06		
575.4153			1	672120.31		
613.3425			1	638120.19		

MSMS Spectrum

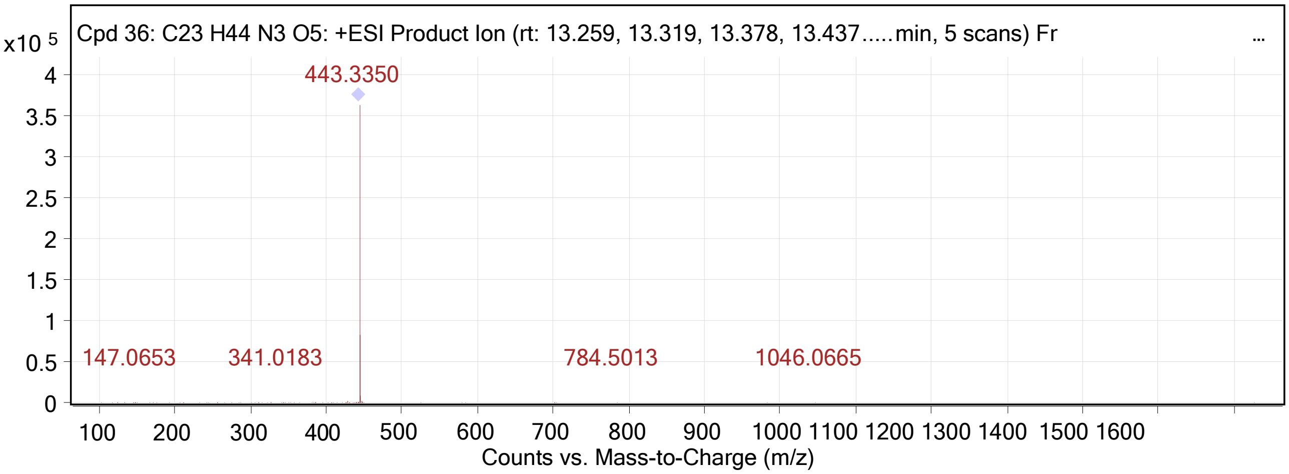

MS/MS Spectrum Peak List

m/z	Calc *m/z*	Diff(ppm)	z	Abund
117.0922	117.091	-10.55		19.8
123.1146	123.1168	18.18		13.42
133.0853	133.0859	4.64		25.66
147.0653	147.0652	-0.73	1	94.13
166.0907	166.0948	24.72		24.32
210.1178	210.121	15.2		15.02
265.2485	265.2512	10.54		18.91
309.2781	309.2775	-1.94	1	49.19
441.3276	220.6596	-500009.52	2	18.2
443.335	443.3354	0.74	1	364348.72

Compound Label	*m/z*	RT	Algorithm	Mass
Cpd 37: C25 H48 N3 O6	487.3626	13.658	Auto MS/MS	486.3552

Compound Chromatograms

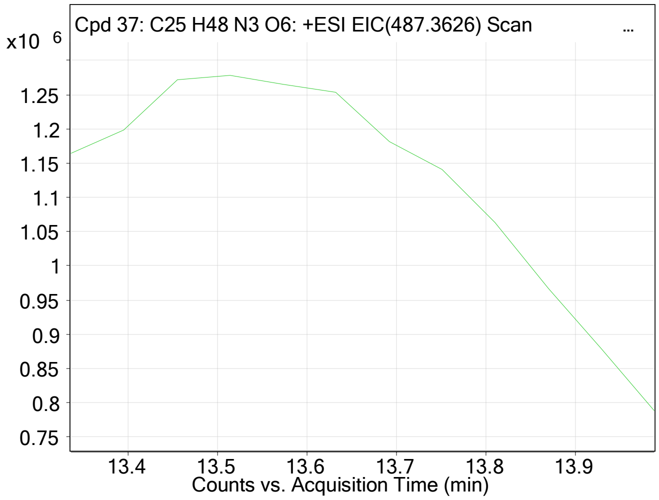

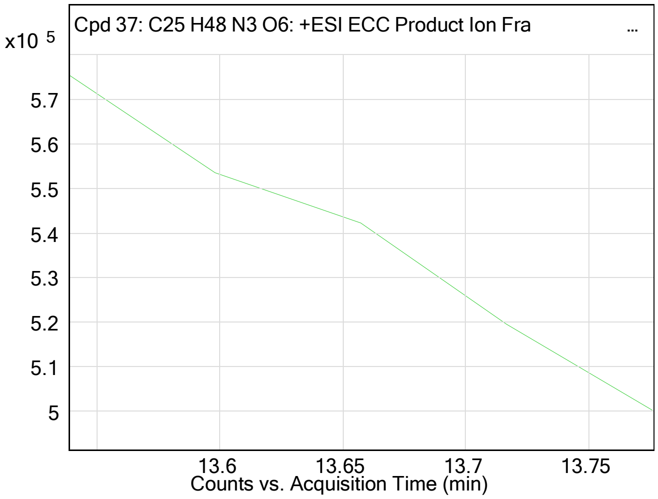

MS Spectrum

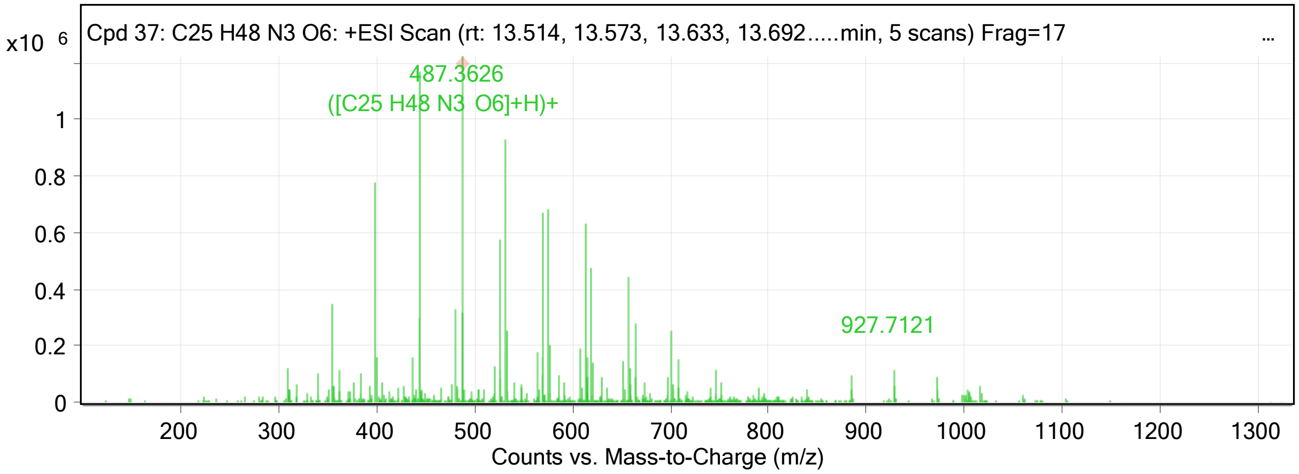

MS Zoomed Spectrum

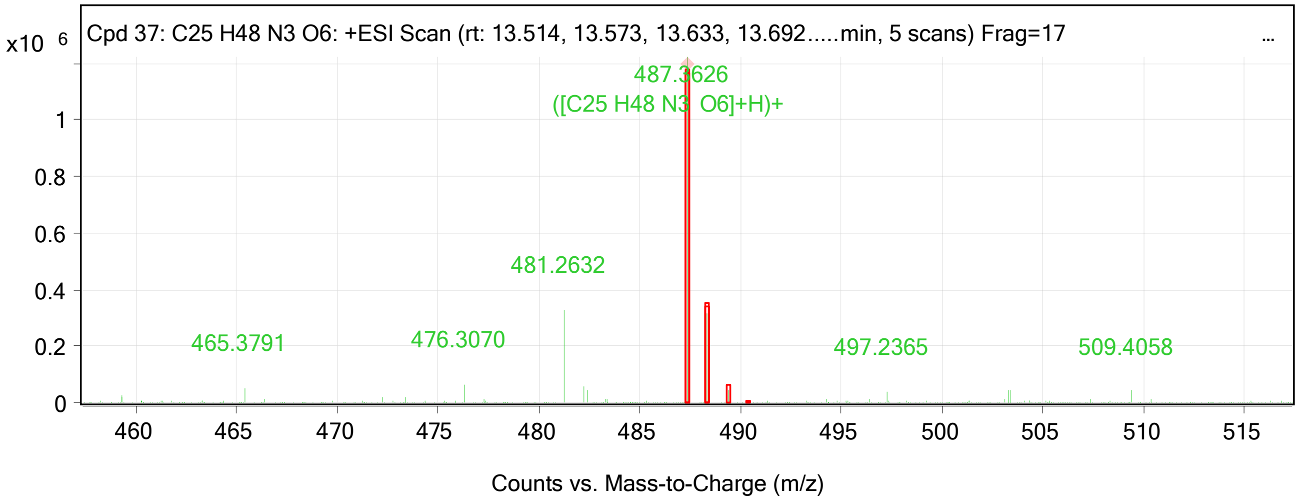

MS Spectrum Peak List

m/z	Calc *m/z*	Diff(ppm)	z	Abund	Formula	Ion
399.3097			1	774213.13		
443.3361			1	1166519.5		
487.3626	487.3616	-2.07	1	1223579	C25 H48 N3 O6	(M+H)+
488.3652	488.3648	-0.89	1	316374.06	C25 H48 N3 O6	(M+H)+
489.3671	489.3674	0.65	1	43932.23	C25 H48 N3 O6	(M+H)+
490.3691	490.3701	1.96	1	5392.98	C25 H48 N3 O6	(M+H)+
531.3892			1	924618.5		
569.3165			1	671237.88		

MSMS Spectrum

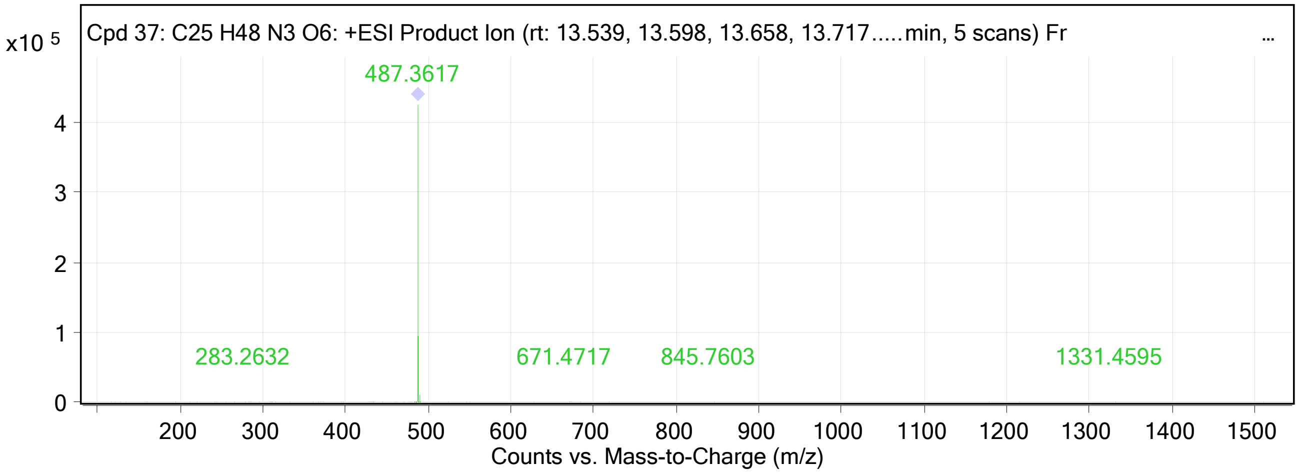

MS/MS Spectrum Peak List

m/z	*Calc m/z*	Diff(ppm)	z	Abund
283.2632	283.2632	0	1	188.25
309.2782	309.2788	1.98	1	57.01
486.3308			2	470.54
486.6625			1	309.01
486.8312			2	272.45
487.3617	487.3616	-0.2	1	426764.44
488.3644			1	93937.3
489.3198			2	519.56
489.3663			1	10124.01
490.3689			1	250.39

Compound Label	*m/z*	RT	Algorithm	Mass
Cpd 38: C23 H44 N3 O5	443.3361	13.674	Auto MS/MS	442.3288

Compound Chromatograms

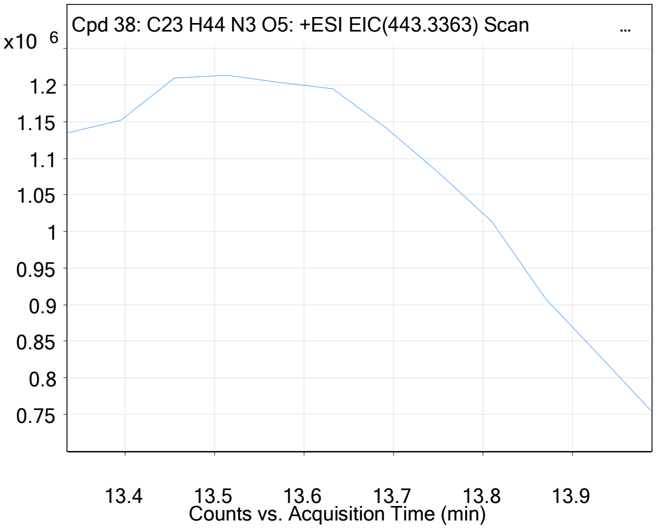

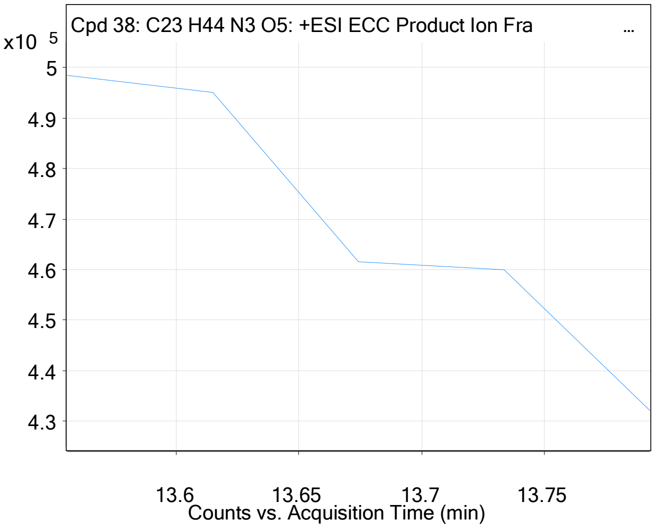

MS Spectrum

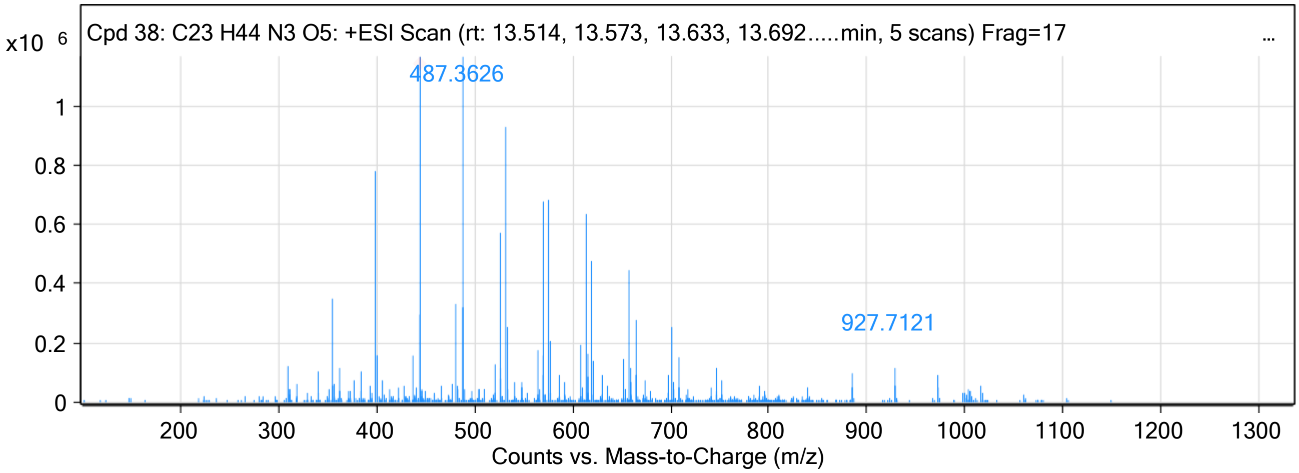

MS Zoomed Spectrum

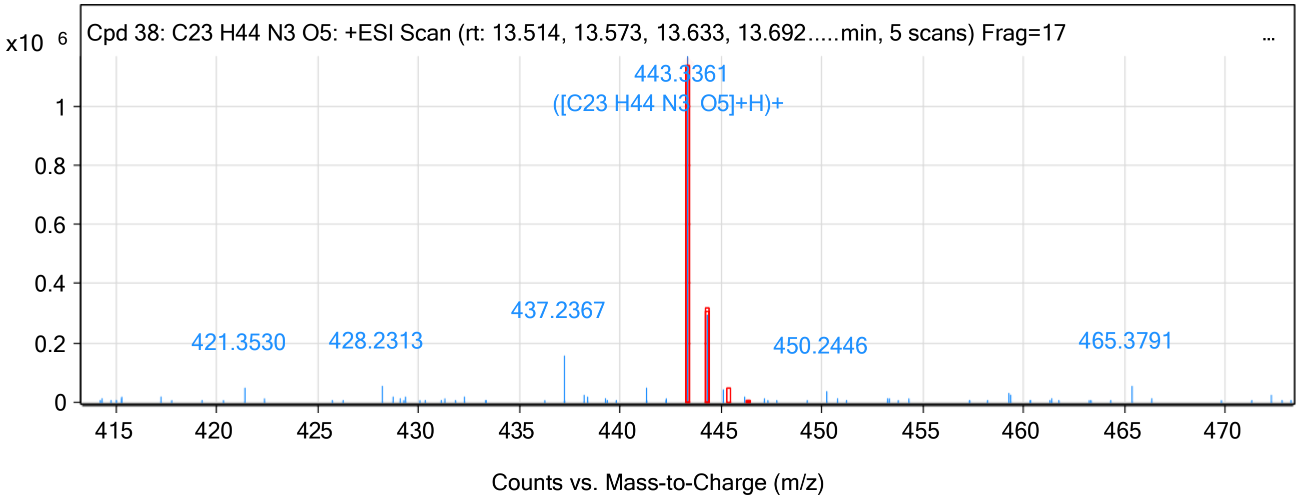

MS Spectrum Peak List

m/z	Calc *m/z*	Diff(ppm)	z	Abund	Formula	Ion
399.3097			1	774213.13		
443.3361	443.3354	-1.7	1	1166519.5	C23 H44 N3 O5	(M+H)+
444.339	444.3385	-0.95	1	293871.81	C23 H44 N3 O5	(M+H)+
445.3408	445.3412	0.99	1	36007.16	C23 H44 N3 O5	(M+H)+
446.343	446.3438	1.91	1	4085.85	C23 H44 N3 O5	(M+H)+
487.3626			1	1223579		
531.3892			1	924618.5		
569.3165			1	671237.88		
575.4151			1	679030.13		
613.3426			1	631652.81		

MSMS Spectrum

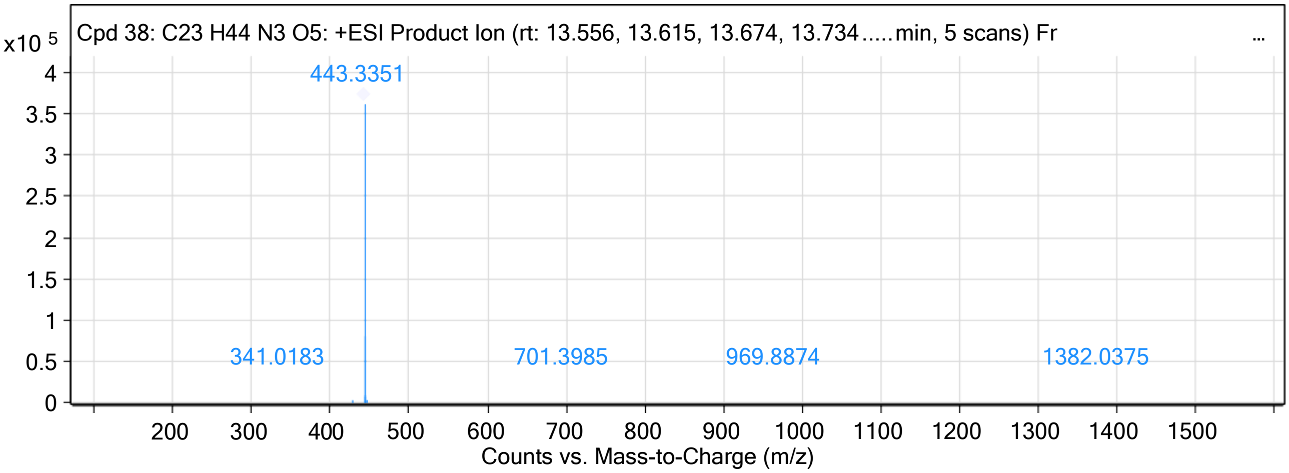

MS/MS Spectrum Peak List

m/z	*Calc m/z*	Diff(ppm)	z	Abund
133.0861	133.0859	-1.67		38.68
136.1194	136.1247	38.56		11.27
147.0656	147.0652	-3.03	1	128.64
149.045	149.0444	-3.72		14.72
174.1295	174.125	-25.79		13.21
217.1249	217.1309	27.49		12.8
283.2631	283.2632	0.21		12.24
303.2323	303.2278	-14.57		17.06
309.2794	309.2788	-1.88		77.3
443.3351	443.3354	0.64	1	361483.03

Compound Label	*m/z*	RT	Algorithm	Mass
Cpd 39: C25 H46 O6	443.3366	13.941	Auto MS/MS	442.3291

Compound Chromatograms

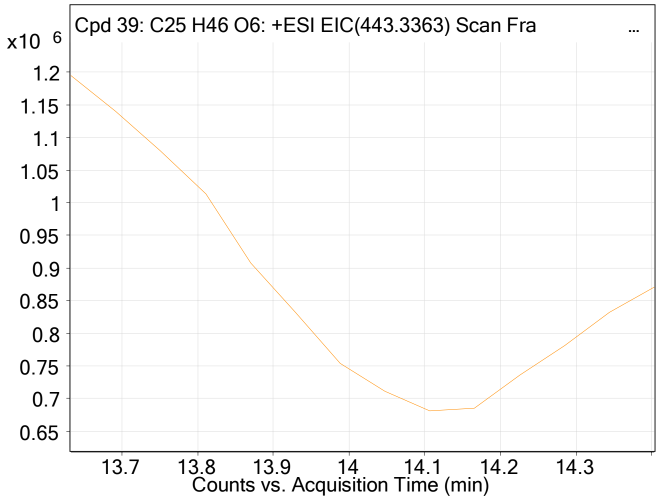

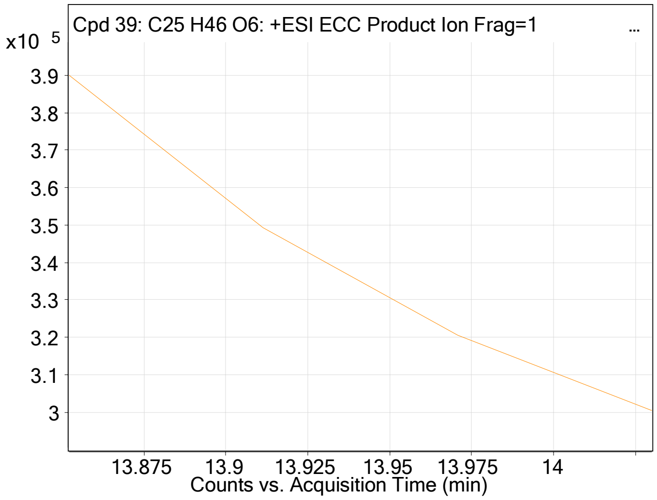

MS Spectrum

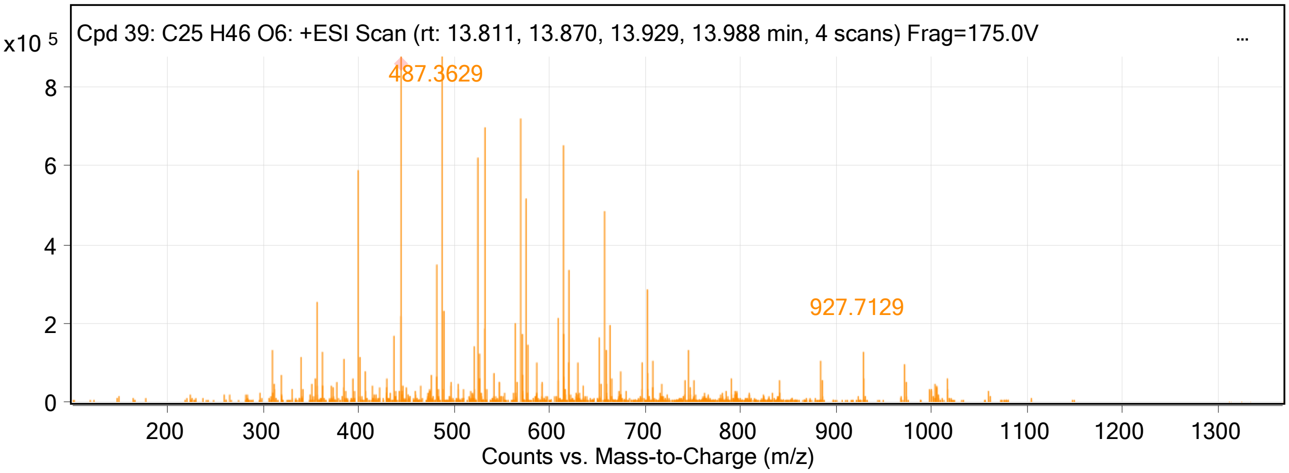

MS Zoomed Spectrum

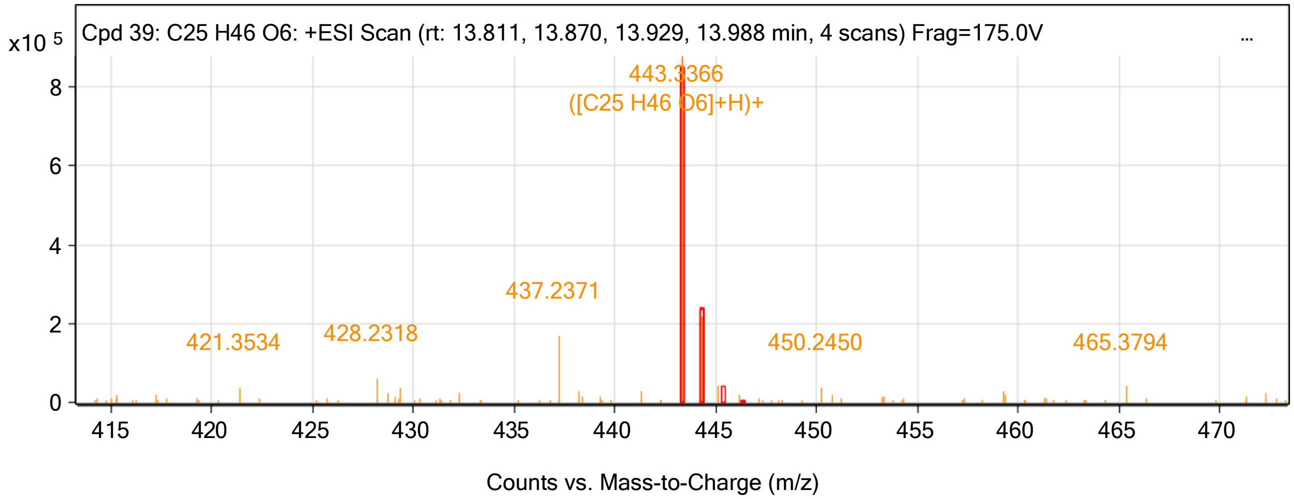

MS Spectrum Peak List

m/z	Calc *m/z*	Diff(ppm)	z	Abund	Formula	Ion
399.3099			1	587368.25		
443.3366	443.3367	0.18	1	877284.44	C25 H46 O6	(M+H)+
444.3392	444.3401	2.07	1	218162.89	C25 H46 O6	(M+H)+
445.3409	445.3429	4.42	1	27472.2	C25 H46 O6	(M+H)+
446.3435	446.3456	4.84	1	3194.58	C25 H46 O6	(M+H)+
487.3629			1	924325.81		
525.2902			1	618520.19		
531.3891			1	695860.69		
569.317			1	718938.38		
613.3435			1	651222.81		

MSMS Spectrum

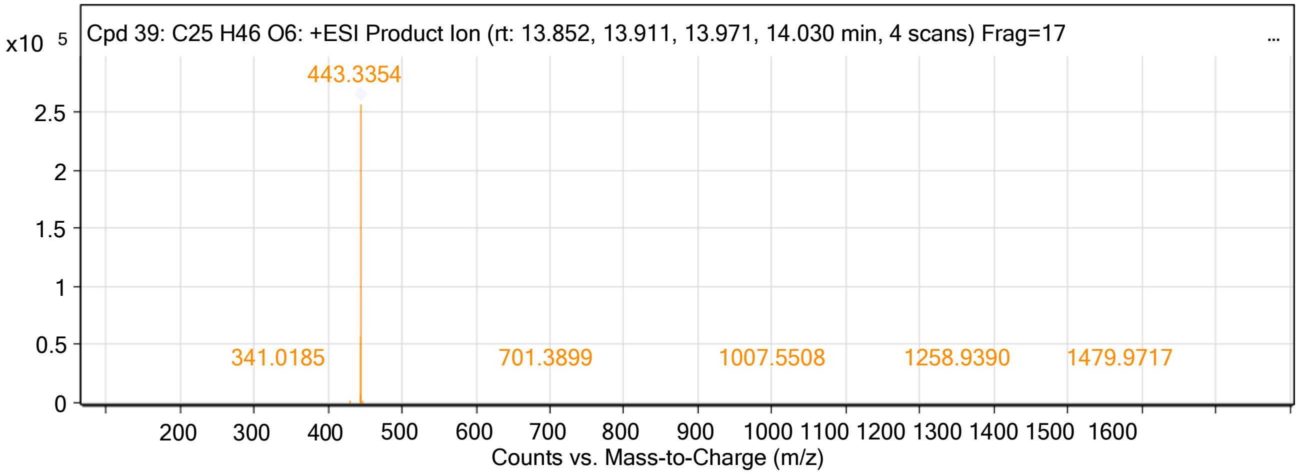

MS/MS Spectrum Peak List

m/z	Calc *m/z*	Diff(ppm)	z	Abund
133.0836	133.0859	17.4		15.88
144.076	144.0781	14.82		17.04
147.0664	147.0652	-7.93		89.39
225.1453	225.1485	14.11		10.11
271.1508	271.154	11.98		13.96
295.2316	295.2268	-16.25		10.82
309.2781	309.2788	2.25		84.31
364.3331	364.3336	1.37		11.65
426.2975	426.2976	0.1		10.02
443.3354	443.3367	3.05	1	256734.39

Compound Label	*m/z*	RT	Algorithm	Mass
Cpd 40: C25 H48 N3 O6	487.3628	13.954	Auto MS/MS	486.3553

Compound Chromatograms

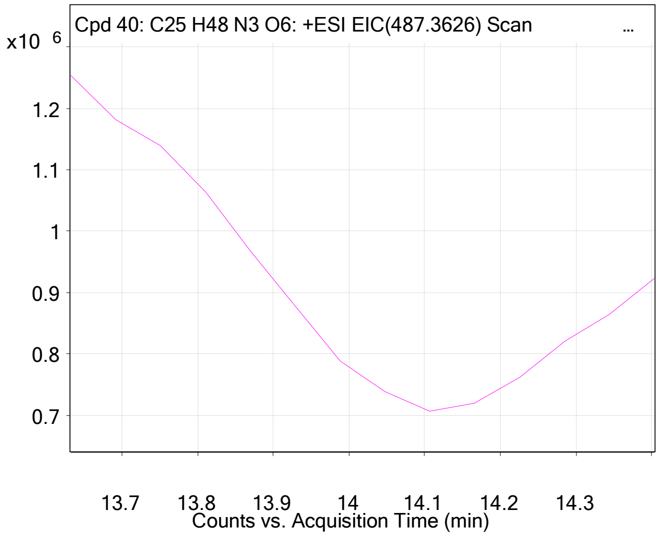

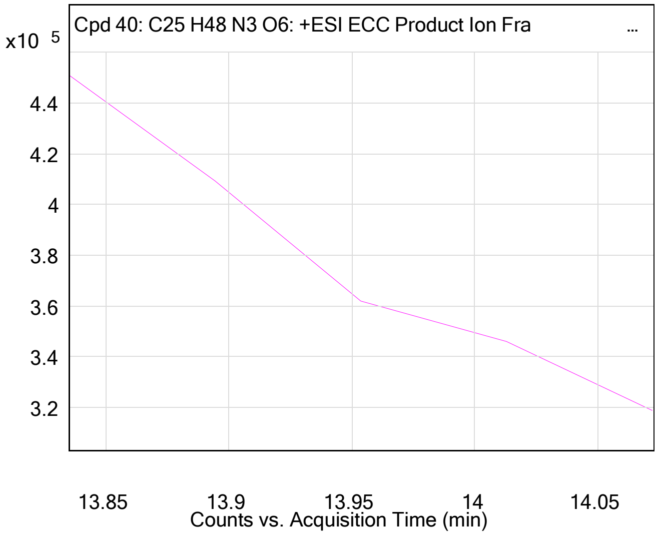

MS Spectrum

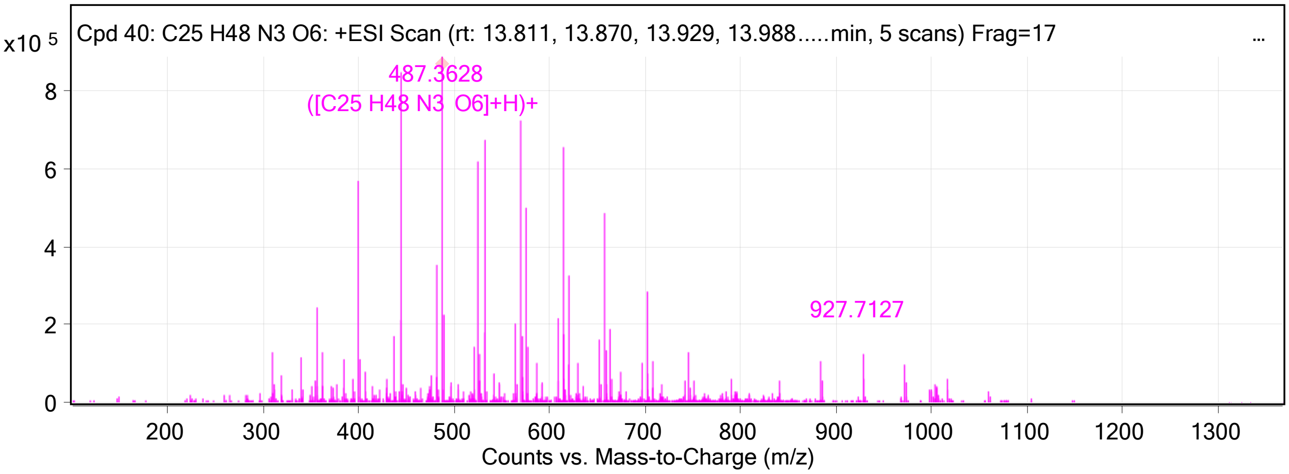

MS Zoomed Spectrum

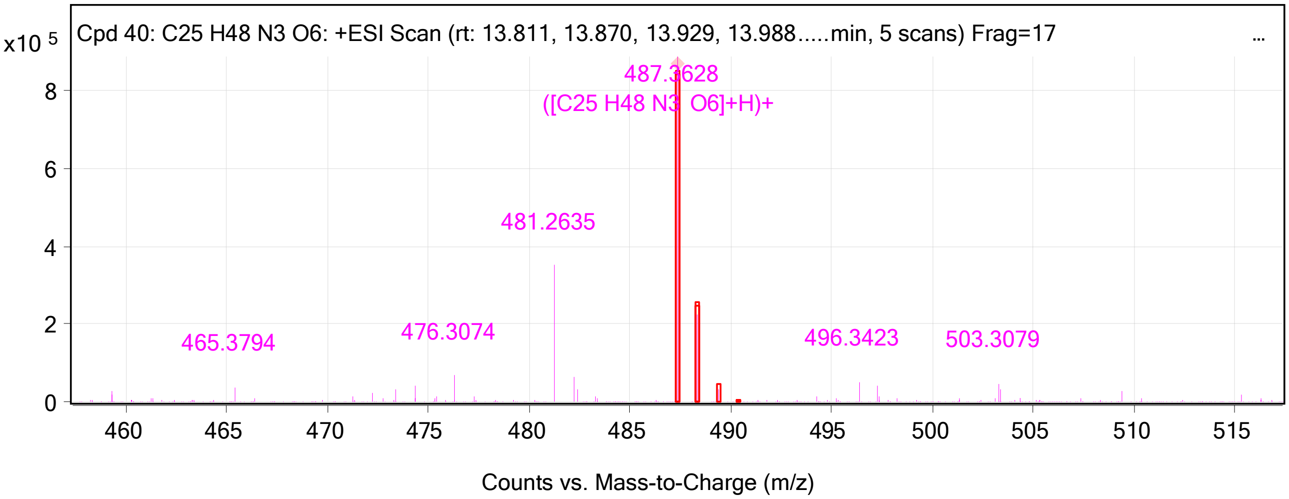

MS Spectrum Peak List

m/z	Calc *m/z*	Diff(ppm)	z	Abund	Formula	Ion
399.3098			1	569323.06		
443.3365			1	844094.88		
487.3628	487.3616	-2.4	1	887258.63	C25 H48 N3 O6	(M+H)+
488.3654	488.3648	-1.27	1	222670.92	C25 H48 N3 O6	(M+H)+
489.3674	489.3674	0.14	1	32047.56	C25 H48 N3 O6	(M+H)+
490.3692	490.3701	1.82	1	4049.34	C25 H48 N3 O6	(M+H)+
525.2901			1	619103.69		

MSMS Spectrum

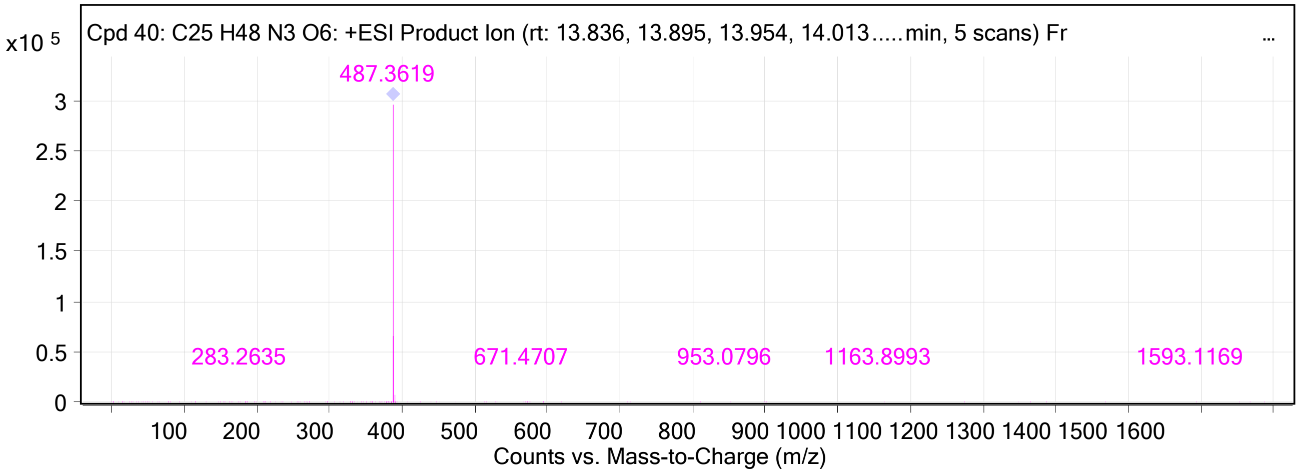

MS/MS Spectrum Peak List

m/z	*Calc m/z*	Diff(ppm)	z	Abund
133.0852	133.0846	-4.44		22.73
144.0776	144.0781	3.61		17.17
247.2408	247.242	5.11		14.65
276.1548	276.1554	2.34		15.49
283.2635	283.2632	-1.12	1	195.22
298.171	298.1761	17.15		18.4
309.2793	309.2788	-1.45	1	43.35
325.2181	325.2207	8.02		16.12
470.3335	470.335	3.36		16.26
487.3619	487.3616	-0.7	1	296290.84

Compound Label	*m/z*	RT	Algorithm	Mass
Cpd 41: C24 H46 N3 O12	569.3166	14.149	Auto MS/MS	568.3092

Compound Chromatograms

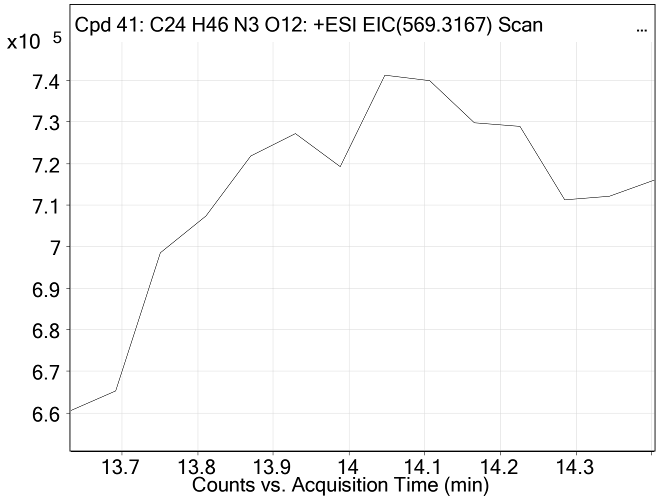

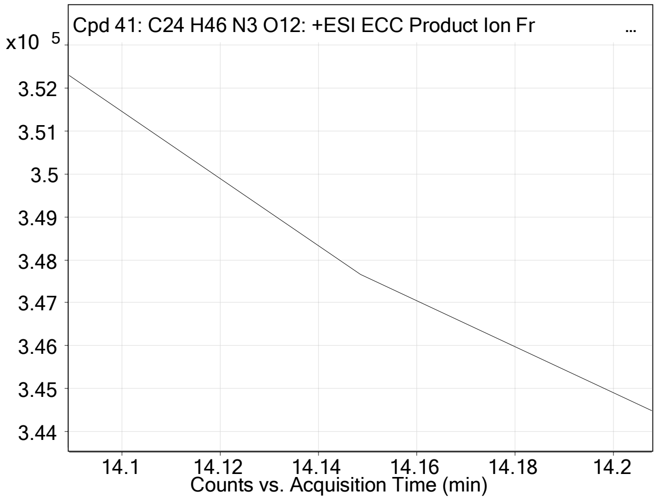

MS Spectrum

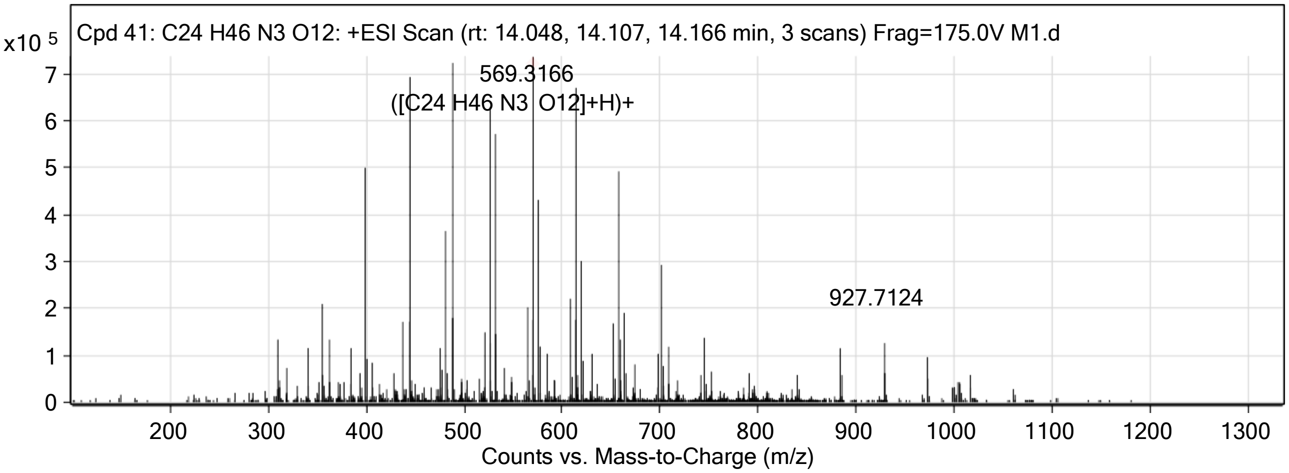

MS Zoomed Spectrum

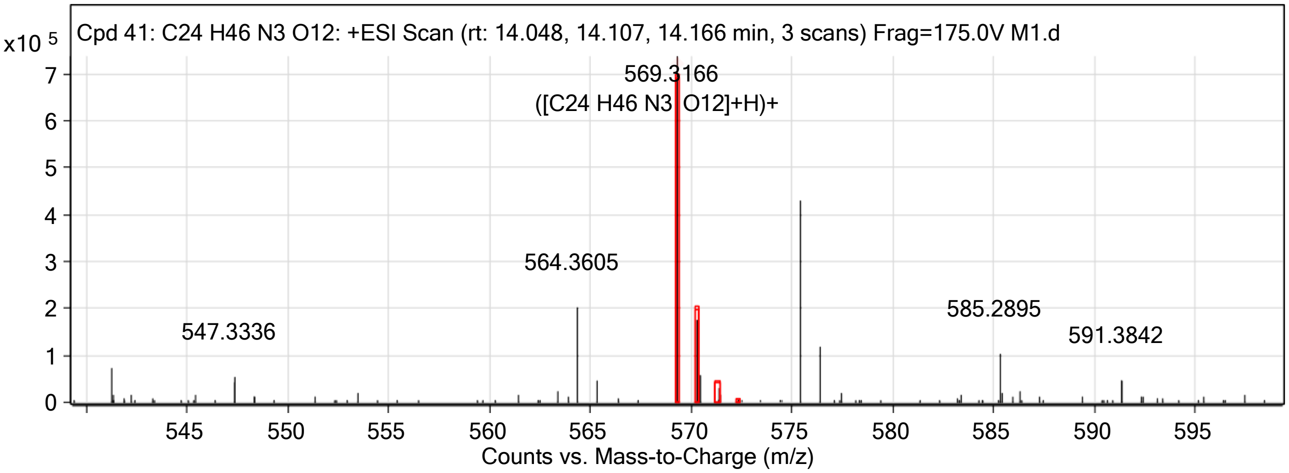

MS Spectrum Peak List

m/z	Calc *m/z*	Diff(ppm)	z	Abund	Formula	Ion
399.3095			1	496558.59		
443.3363			1	692726.75		
487.3625			1	722229.81		
525.2899			1	625476		
531.3886			1	568651.56		
569.3166	569.3154	-2.08	1	737069.81	C24 H46 N3 O12	(M+H)+
570.3192	570.3186	-1.07	1	174342.05	C24 H46 N3 O12	(M+H)+
571.3211	571.3209	-0.3	1	28989.62	C24 H46 N3 O12	(M+H)+
572.3241	572.3235	-0.97	1	4518.62	C24 H46 N3 O12	(M+H)+
613.3431			1	670204.25		

MSMS Spectrum

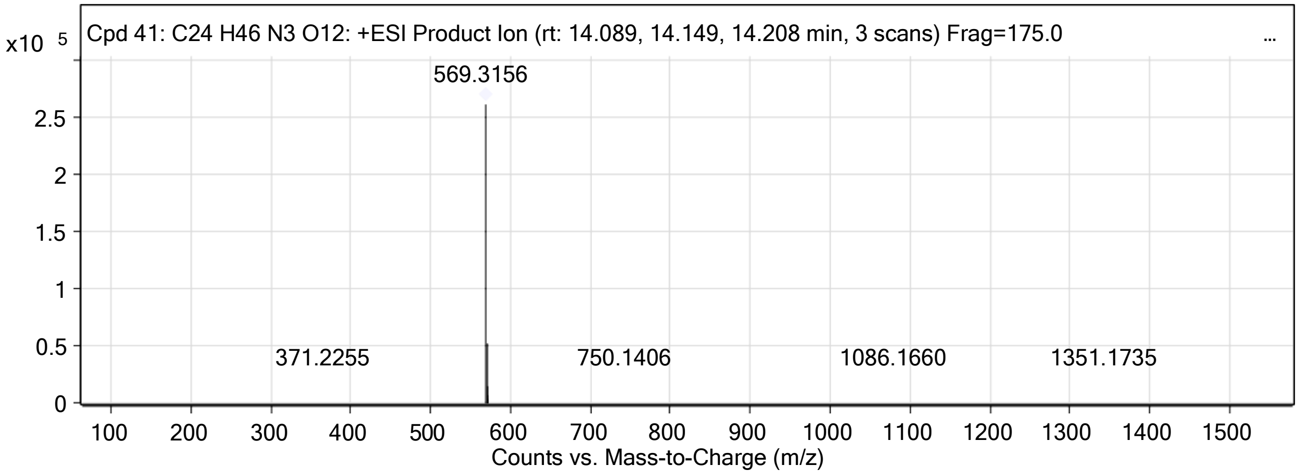

MS/MS Spectrum Peak List

m/z	*Calc m/z*	Diff(ppm)	z	Abund
117.0908	117.091	1.45		27.86
133.087	133.0859	-8.37		116.29
144.0766	144.0768	1.24		26.3
177.1109	177.1108	-0.5		47.76
221.1384	221.1384	-0.39		25.28
309.1917	309.1908	-3.1		24.04
309.2775	309.2775	-0.09		28.07
327.2013	327.2	-4.07		25.22
371.2255	371.2262	1.82	1	105.88
569.3156	569.3154	-0.37	1	261418.05

Compound Label	*m/z*	RT	Algorithm	Mass
Cpd 42: C25 H48 N3 O6	487.3625	14.251	Auto MS/MS	486.3551

Compound Chromatograms

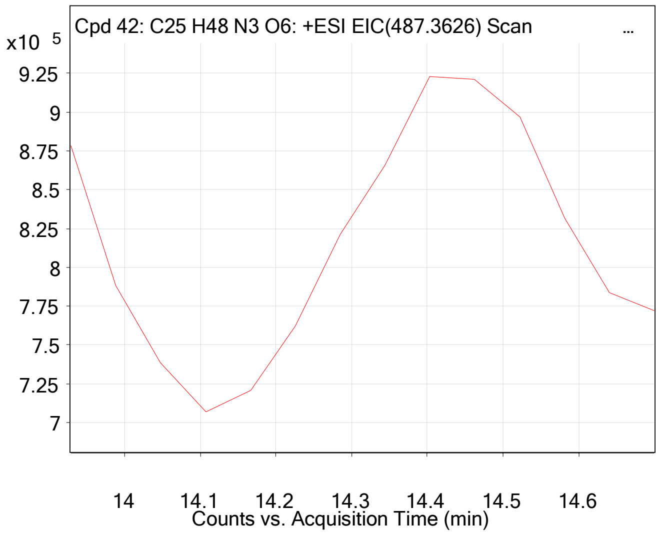

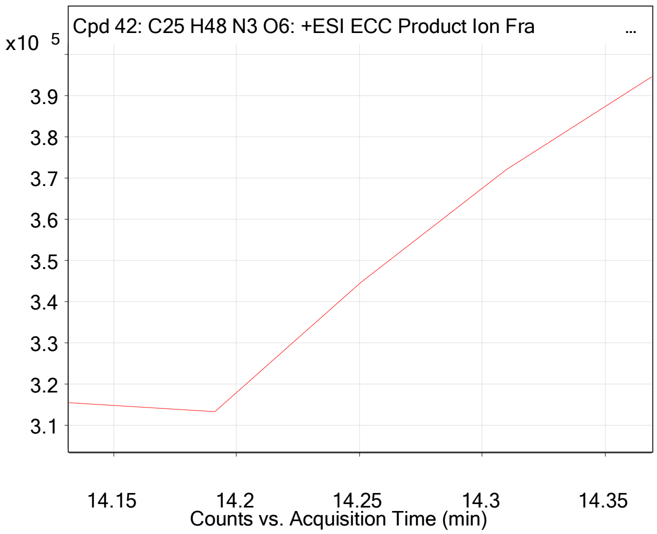

MS Spectrum

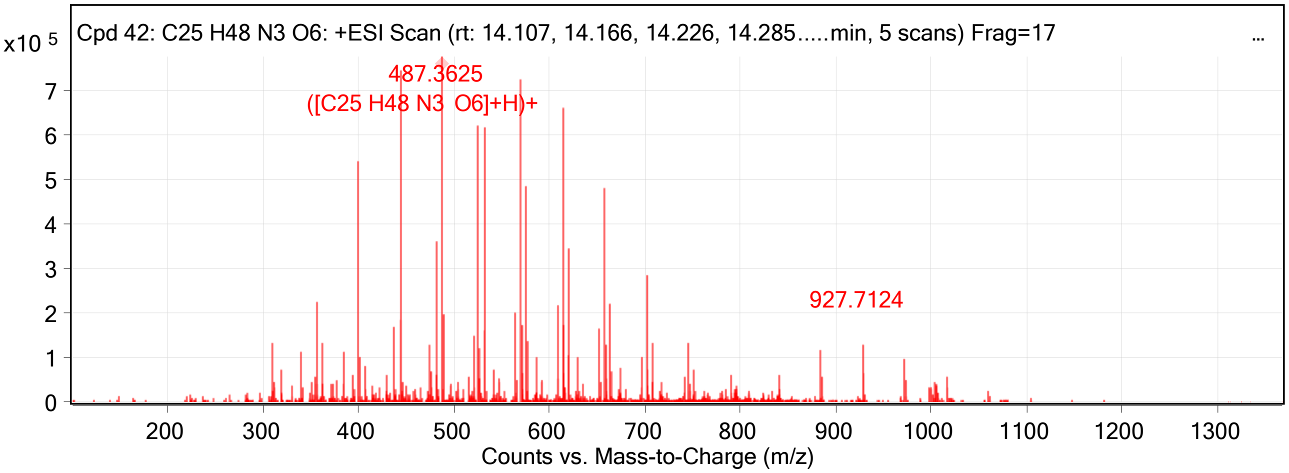

MS Zoomed Spectrum

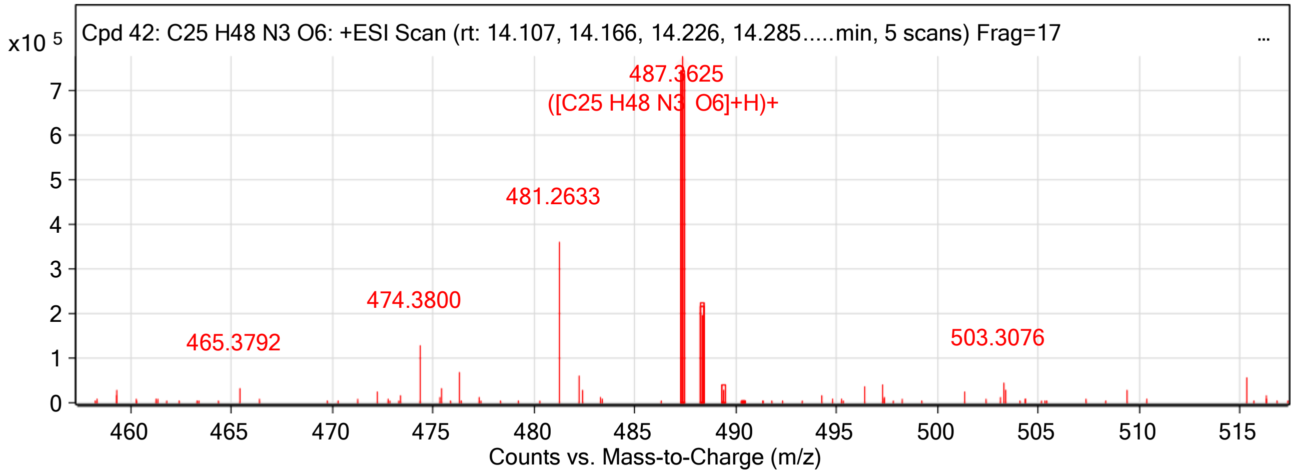

MS Spectrum Peak List

m/z	Calc *m/z*	Diff(ppm)	z	Abund	Formula	Ion
399.3095			1	539587.88		
443.3363			1	743429.88		
487.3625	487.3616	-1.92	1	775392.19	C25 H48 N3 O6	(M+H)+
488.3651	488.3648	-0.67	1	195562.28	C25 H48 N3 O6	(M+H)+
489.367	489.3674	0.82	1	27537.32	C25 H48 N3 O6	(M+H)+
490.3691	490.3701	2.02	1	3565.99	C25 H48 N3 O6	(M+H)+
525.2899			1	618476.13		
531.3887			1	615669.88		
569.3166			1	724523.06		
613.3431			1	661502.88		

MSMS Spectrum

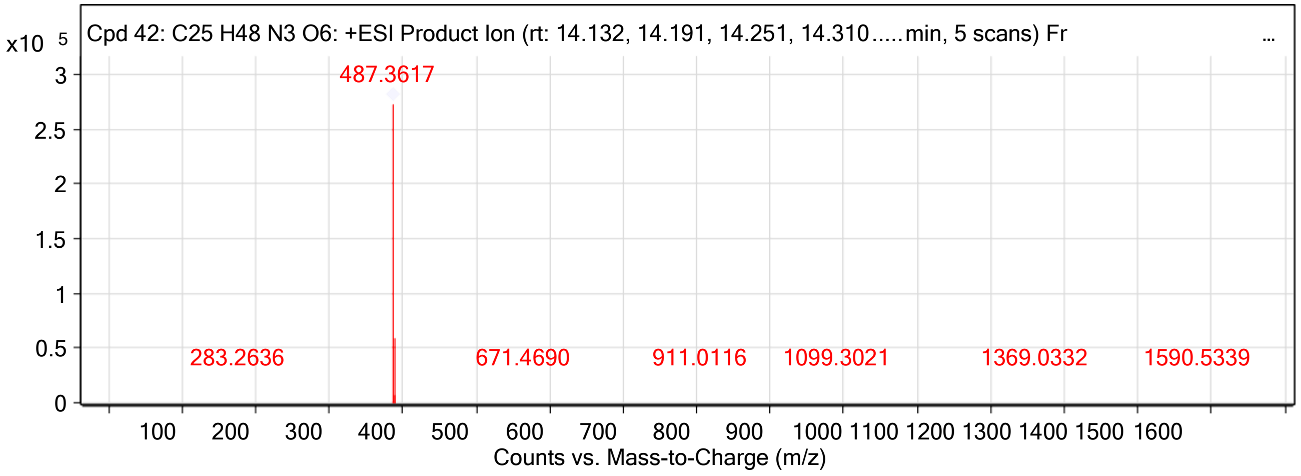

MS/MS Spectrum Peak List

m/z	*Calc m/z*	Diff(ppm)	z	Abund
133.0848	133.0846	-2		26.31
283.2636	283.2632	-1.61	1	235.92
309.279	309.2788	-0.72	1	127.05
484.3389	242.1688	-500001.34	2	69.97
486.3291			2	573.01
486.8314			2	350.36
487.3617	487.3616	-0.13	1	272653.59
488.3643			1	59493.55
489.3189			2	557.49
489.3667			1	6733.72

Compound Label	*m/z*	RT	Algorithm	Mass
Cpd 43: C23 H44 N3 O5	443.3363	14.386	Auto MS/MS	442.3289

Compound Chromatograms

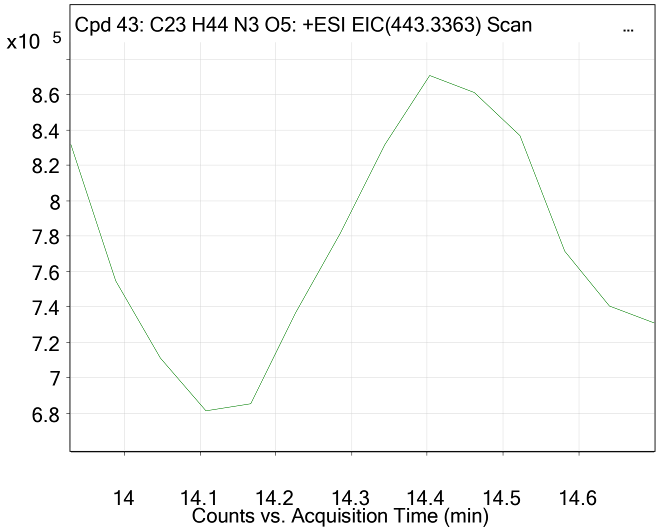

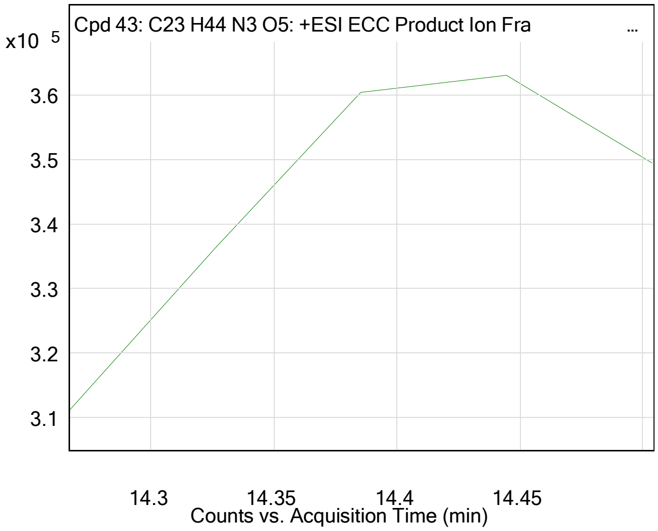

MS Spectrum

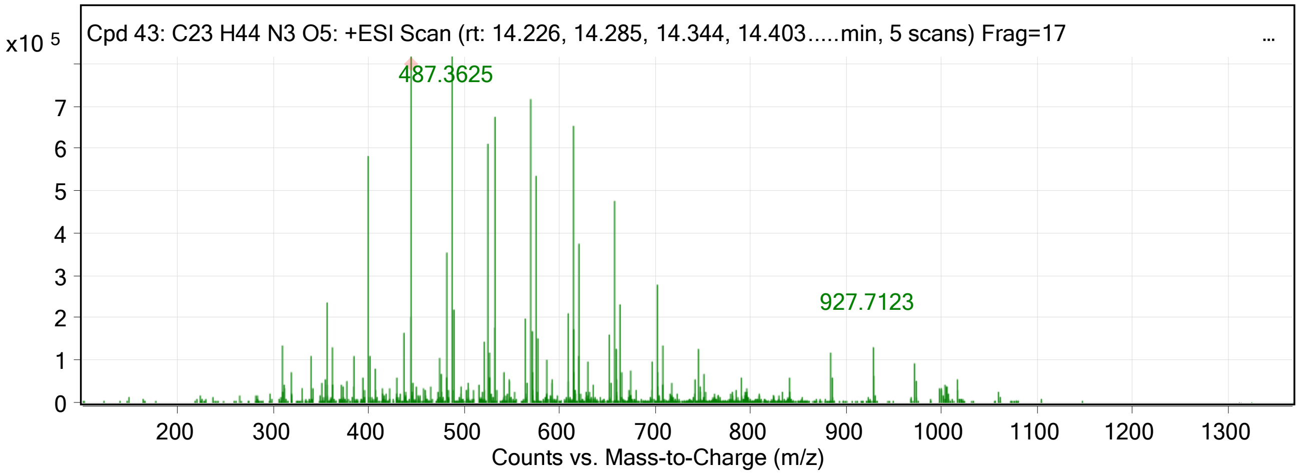

MS Zoomed Spectrum

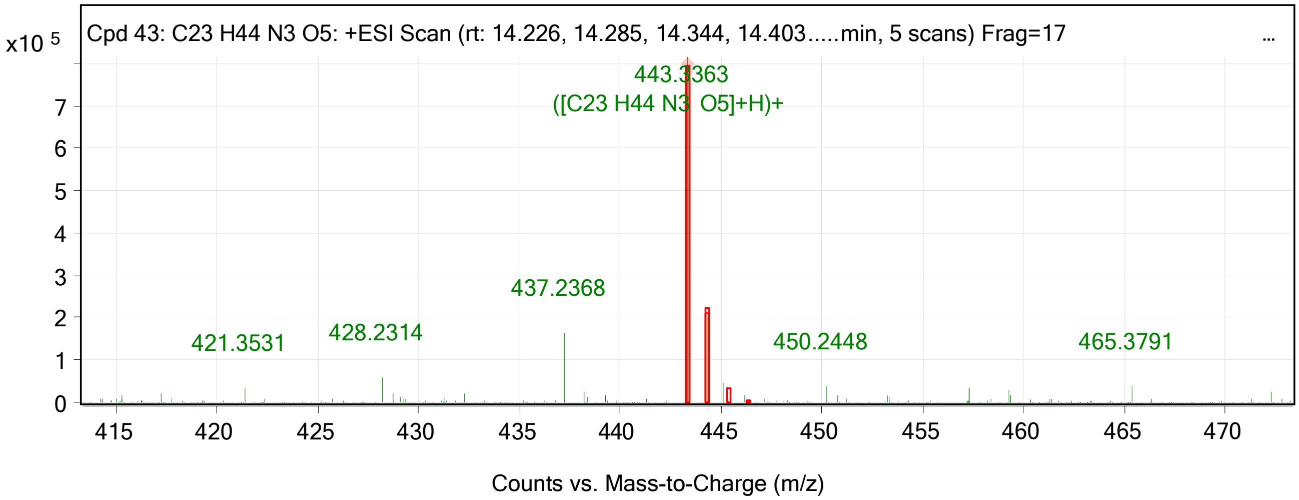

MS Spectrum Peak List

m/z	Calc *m/z*	Diff(ppm)	z	Abund	Formula	Ion
399.3095			1	579705.38		
443.3363	443.3354	-2.08	1	816424	C23 H44 N3 O5	(M+H)+
444.3389	444.3385	-0.86	1	202356.81	C23 H44 N3 O5	(M+H)+
445.3406	445.3412	1.26	1	25715.79	C23 H44 N3 O5	(M+H)+
446.3428	446.3438	2.23	1	2823	C23 H44 N3 O5	(M+H)+
487.3625			1	858582		
525.2898			1	611591.56		

MSMS Spectrum

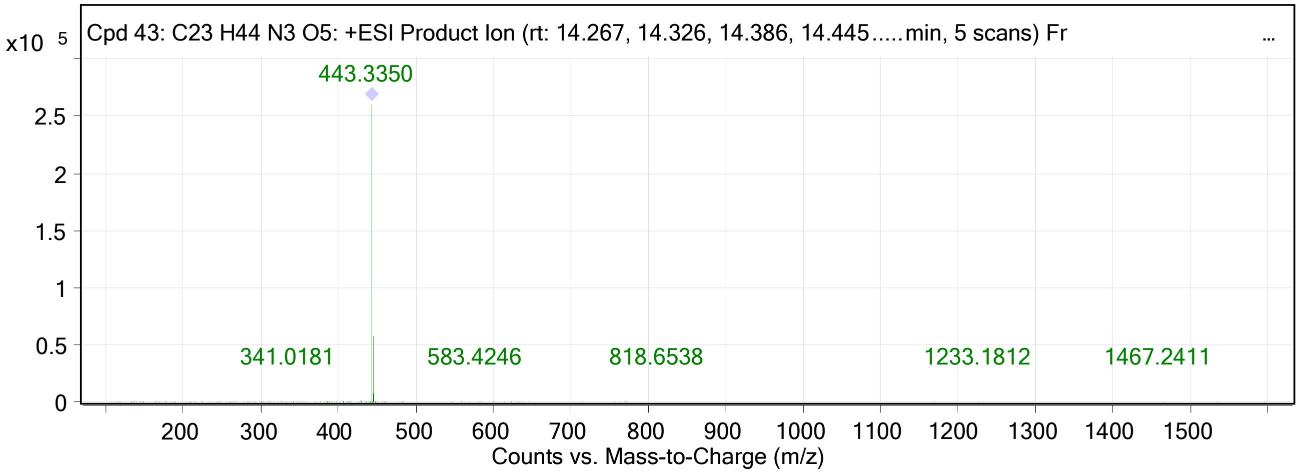

MS/MS Spectrum Peak List

m/z	*Calc m/z*	Diff(ppm)	z	Abund
113.0583	113.0584	0.17		13.63
117.0914	117.091	-3.28		14.24
137.0134	137.0193	42.72		16.41
147.0657	147.0652	-3.71		93.11
149.0452	149.0444	-5.34		13.42
177.1119	177.1121	1.47		13.57
303.2338	303.2278	-19.66		20.33
309.2779	309.2775	-1.47	1	51.53
441.3256	220.6596	-500007.32	2	13.49
443.335	443.3354	0.89	1	260292.8

Compound Label	*m/z*	RT	Algorithm	Mass
Cpd 44: C25 H48 N3 O6	487.3625	14.547	Auto MS/MS	486.3551

Compound Chromatograms

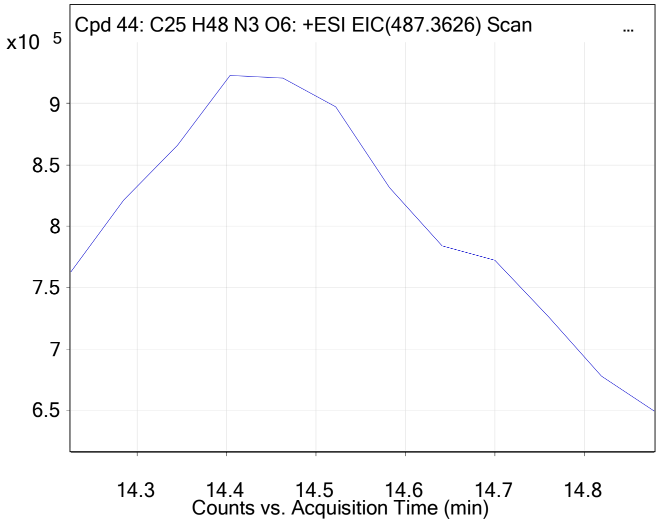

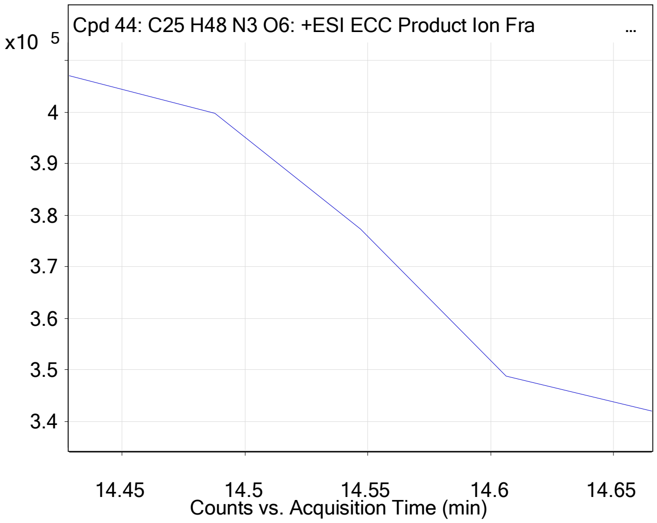

MS Spectrum

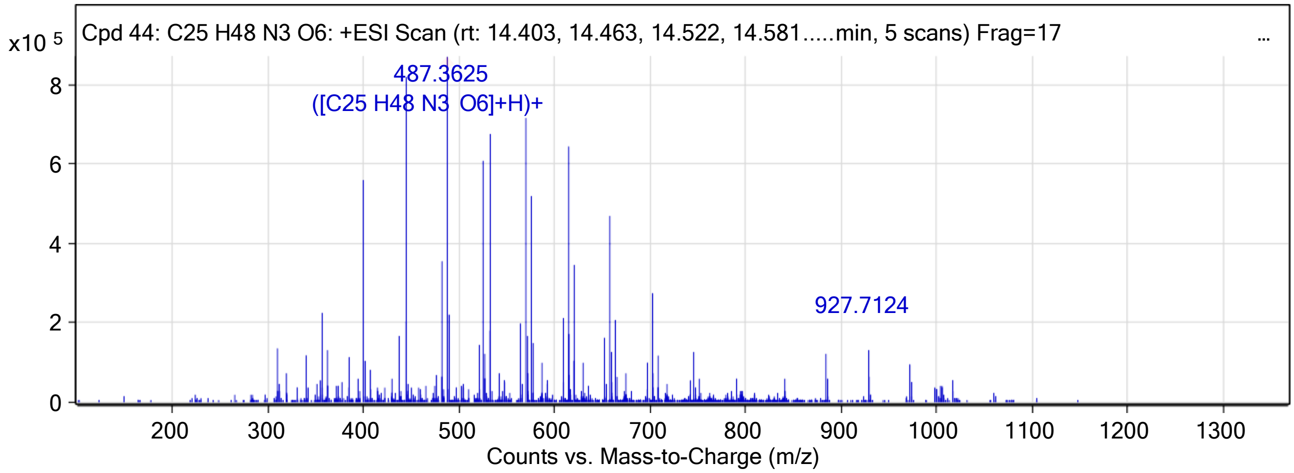

MS Zoomed Spectrum

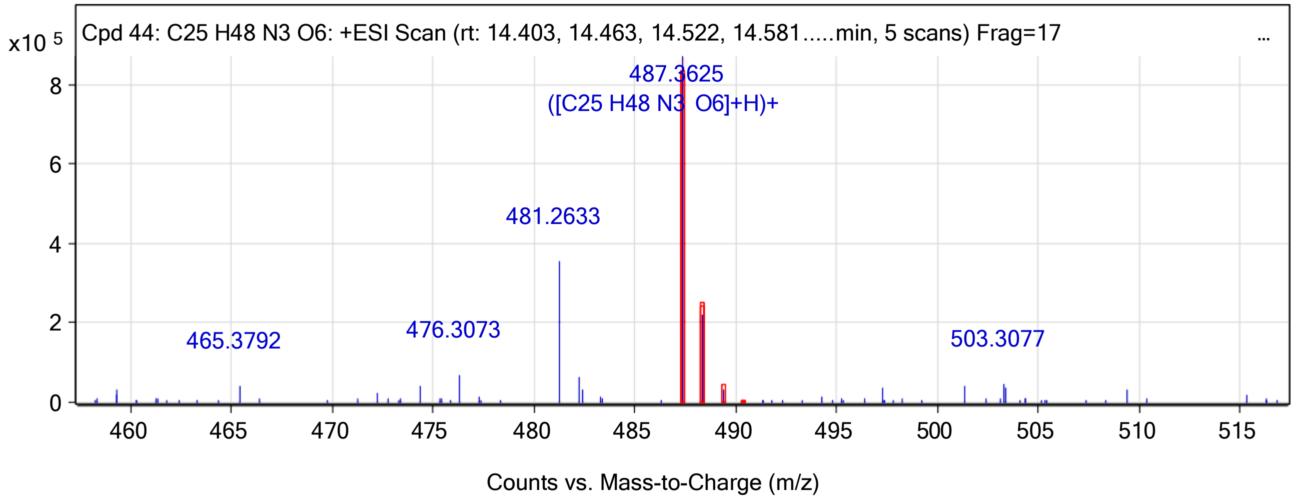

MS Spectrum Peak List

m/z	Calc *m/z*	Diff(ppm)	z	Abund	Formula	Ion
399.3095			1	556959.88		
443.3364			1	816083.69		
487.3625	487.3616	-1.94	1	871329.81	C25 H48 N3 O6	(M+H)+
488.3652	488.3648	-0.89	1	220065.41	C25 H48 N3 O6	(M+H)+
489.3672	489.3674	0.41	1	30525.55	C25 H48 N3 O6	(M+H)+
490.3692	490.3701	1.81	1	3817.85	C25 H48 N3 O6	(M+H)+
525.29			1	607888.88		
531.3888			1	674885.31		
569.3166			1	713911		
613.3431			1	642977.88		

MSMS Spectrum

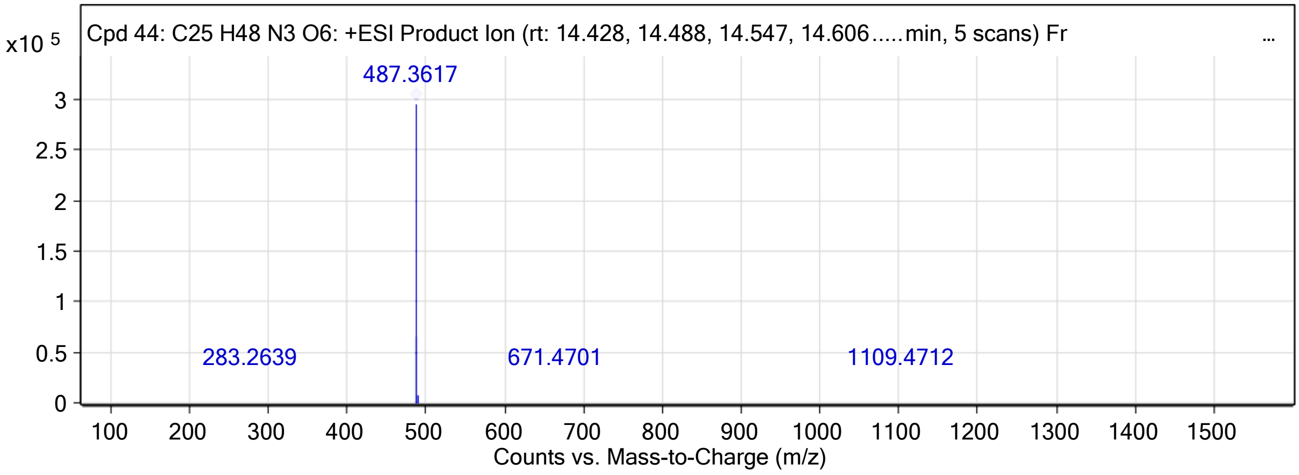

MS/MS Spectrum Peak List

m/z	*Calc m/z*	Diff(ppm)	z	Abund
210.1166	210.1125	-19.73		27.18
265.2553	265.2526	-10.24		30.16
283.2639	283.2632	-2.67	1	226.09
309.2791	309.2788	-0.86		61.34
310.2821	310.2853	10.28		35.13
486.3621	486.3538	-17.21		55.4
487.3617	487.3616	-0.27	1	294705.88
488.3644			1	64724.89
489.317			2	507.91
489.3668			1	6951.67

Compound Label	*m/z*	RT	Algorithm	Mass
Cpd 45: C26 H48 O13	569.3169	14.911	Auto MS/MS	568.3094

Compound Chromatograms

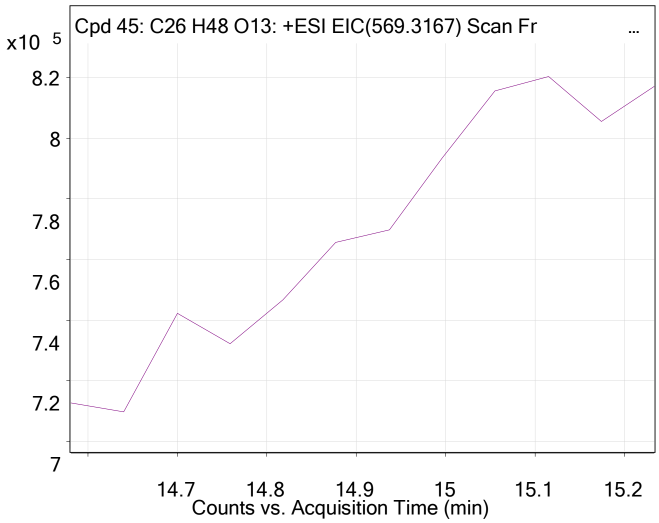

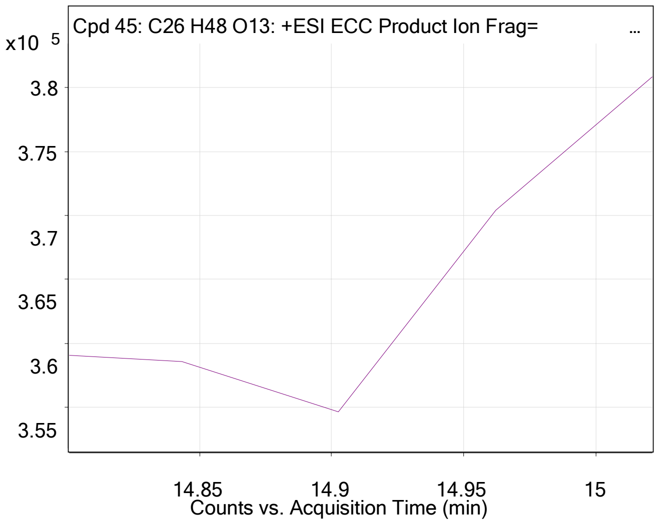

MS Spectrum

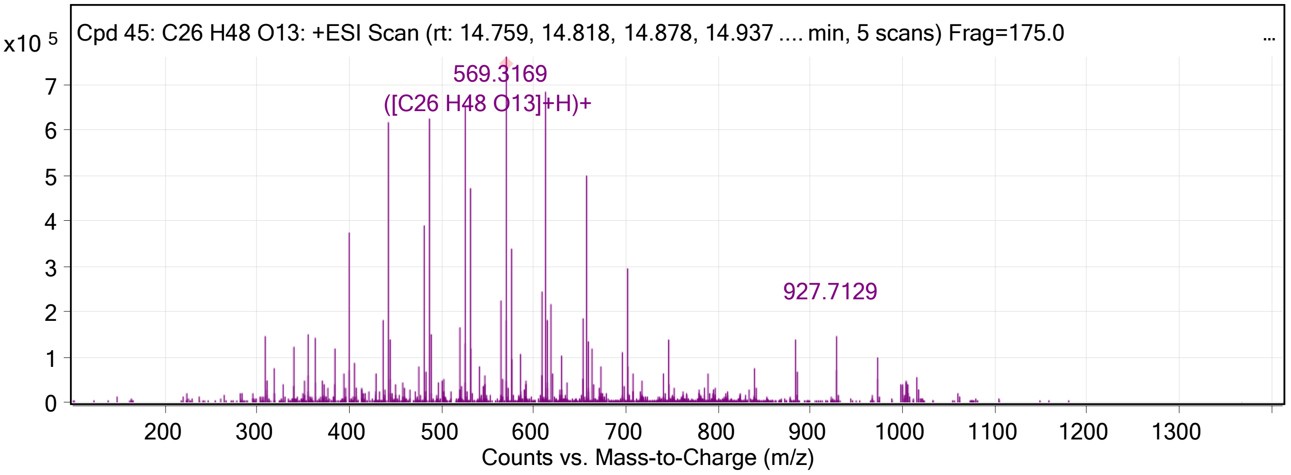

MS Zoomed Spectrum

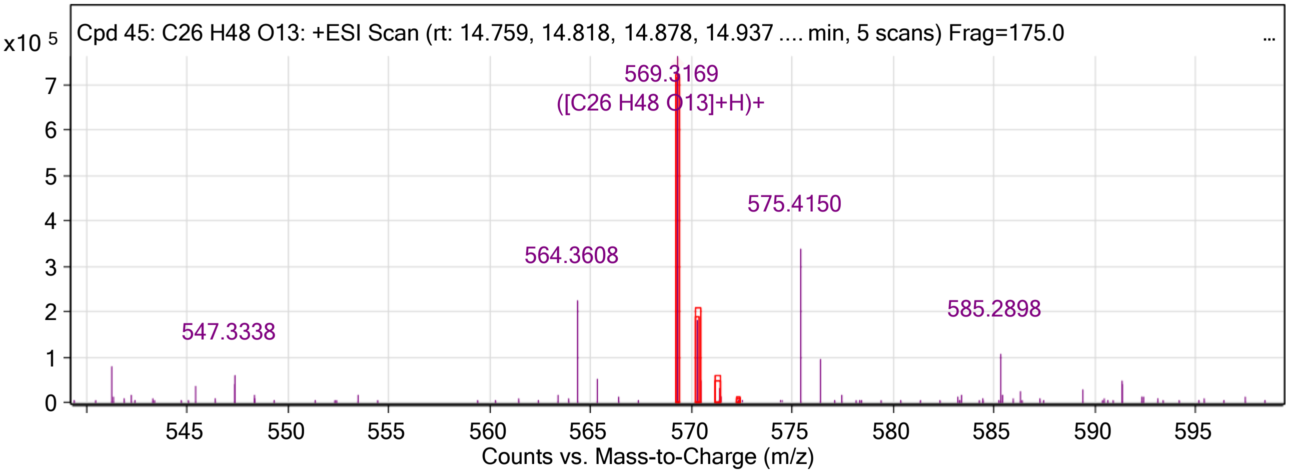

MS Spectrum Peak List

m/z	Calc *m/z*	Diff(ppm)	z	Abund	Formula	Ion
443.3361			1	618224.5		
487.3626			1	624280.56		
525.2903			1	650290.88		
531.3889			1	471339.75		
569.3169	569.3168	-0.24	1	761485.75	C26 H48 O13	(M+H)+
570.3194	570.3202	1.41	1	180016.53	C26 H48 O13	(M+H)+
571.3215	571.3226	1.98	1	30742.43	C26 H48 O13	(M+H)+
572.3248	572.3253	0.77	1	4531.95	C26 H48 O13	(M+H)+
613.3435			1	684831.44		
657.3693			1	498673		

MSMS Spectrum

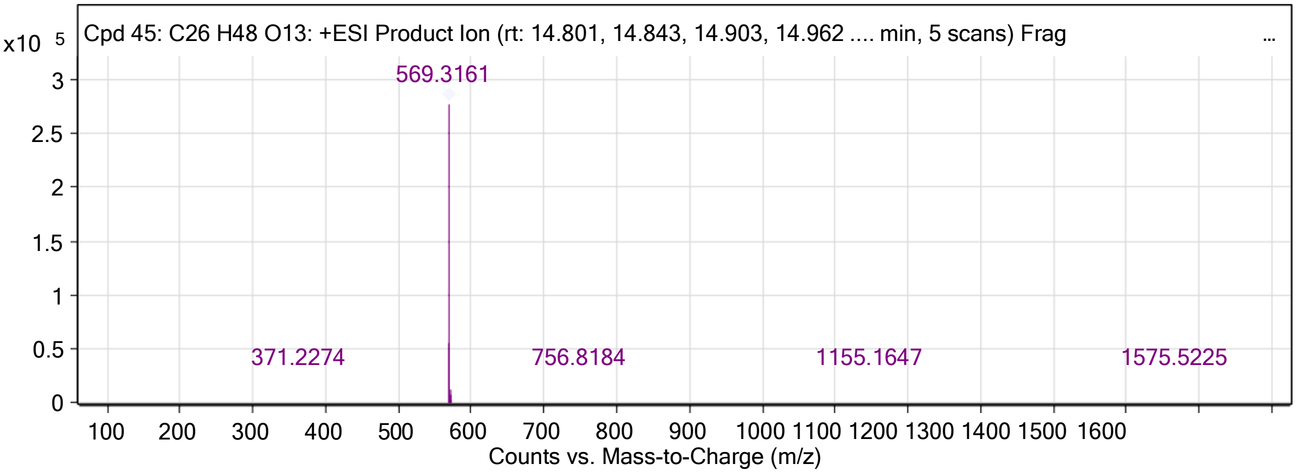

MS/MS Spectrum Peak List

m/z	Calc *m/z*	Diff(ppm)	z	Abund
102.0691	102.0675	-15.58		15.48
133.0864	133.0859	-3.56		133.05
177.1119	177.1121	1.31		84.02
221.1394	110.5689	-500003.58	2	20.11
307.217	307.2115	-17.94		17.42
309.2783	309.2788	1.54	1	34.47
509.334	509.332	-3.87		11.88
528.3148	528.314	-1.41		12.13
538.2978	269.1489	-500000	2	17.71
569.3161	569.3168	1.24	1	277176.44

Compound Label	*m/z*	RT	Algorithm	Mass
Cpd 46: C26 H48 O13	569.3169	15.199	Auto MS/MS	568.3094

Compound Chromatograms

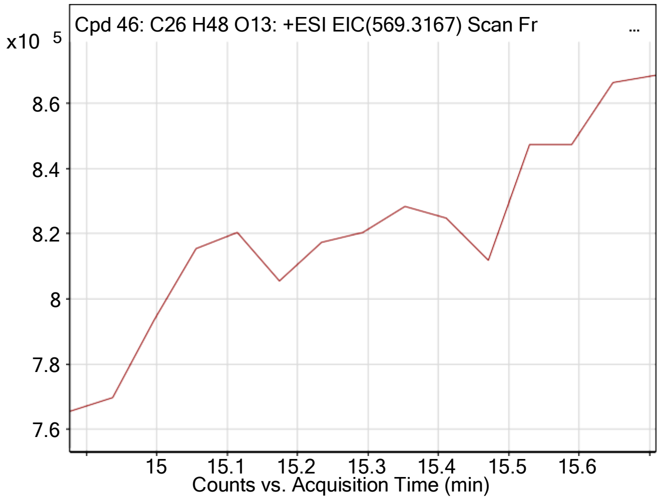

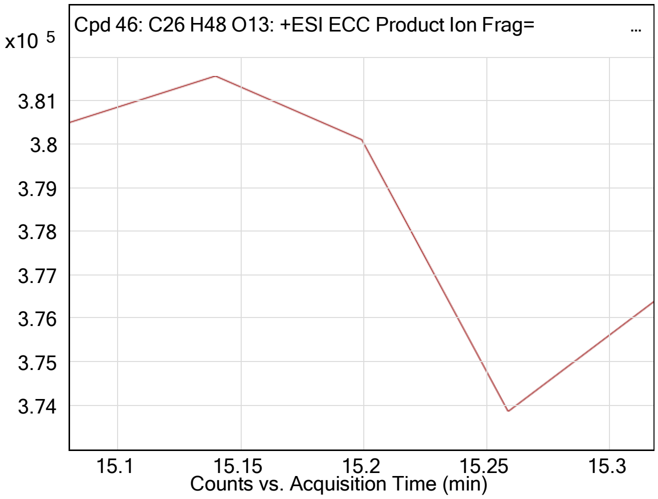

MS Spectrum

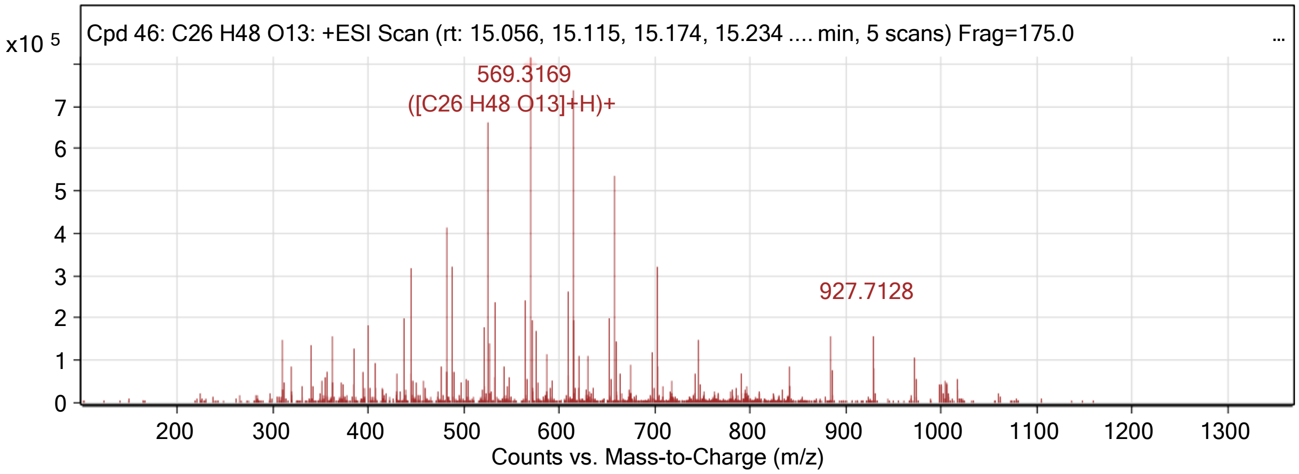

MS Zoomed Spectrum

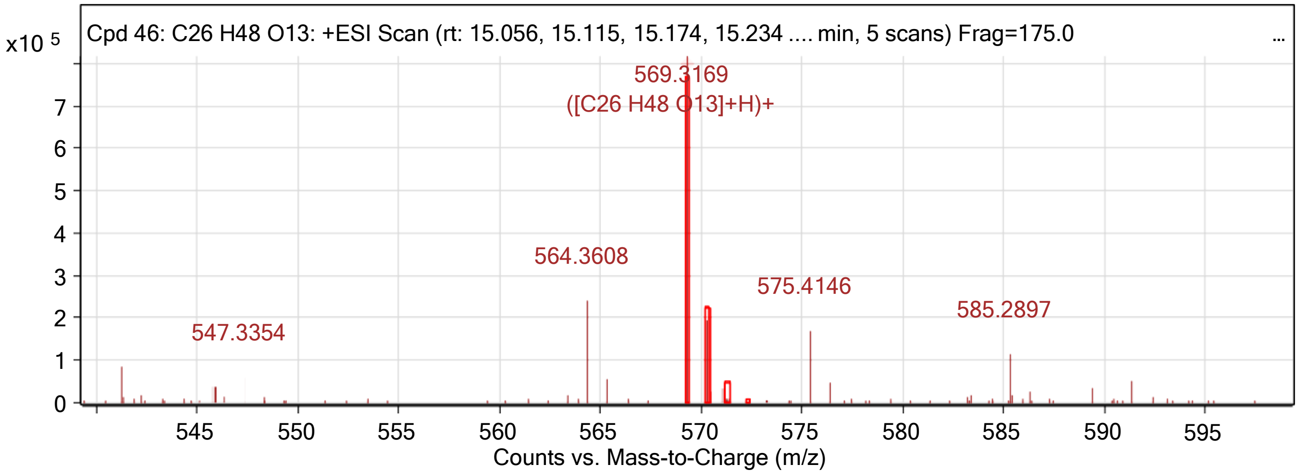

MS Spectrum Peak List

m/z	Calc *m/z*	Diff(ppm)	z	Abund	Formula	Ion
481.2635			1	411716.41		
487.3622			1	321046		
525.2906			1	660181		
569.3169	569.3168	-0.17	1	815754.63	C26 H48 O13	(M+H)+
570.3194	570.3202	1.36	1	194135.42	C26 H48 O13	(M+H)+
571.3216	571.3226	1.74	1	32651.82	C26 H48 O13	(M+H)+
572.3246	572.3253	1.25	1	4417.55	C26 H48 O13	(M+H)+
613.3434			1	734624.19		
657.3693			1	534955.63		

MSMS Spectrum

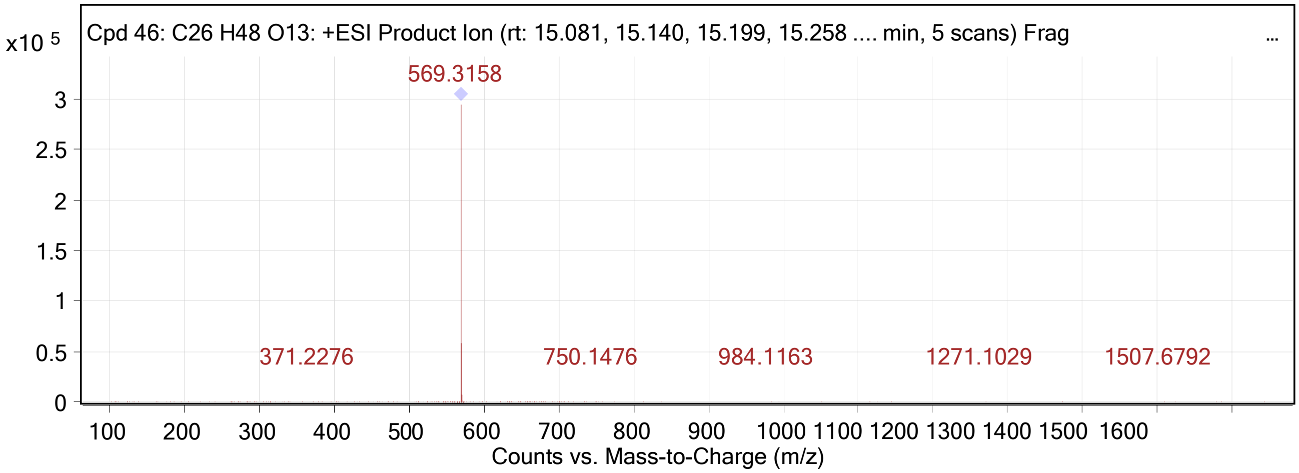

MS/MS Spectrum Peak List

m/z	*Calc m/z*	Diff(ppm)	z	Abund
111.1161	111.1168	6.68		11.16
133.0873	133.0859	-10.05		114.11
177.1113	177.1121	4.68		28.78
283.2637	283.2632	-1.77		14.99
309.2813	309.2788	-8.22		43.44
311.2952	311.2945	-2.51		30.91
513.321	513.3269	11.49		15.77
538.2968	538.2984	2.88		11.82
569.3158	569.3168	1.63	1	295036.31
570.3185			1	57478.53

Compound Label	*m/z*	RT	Algorithm	Mass
Cpd 47: C28 H52 O14	613.343	15.394	Auto MS/MS	612.3355

Compound Chromatograms

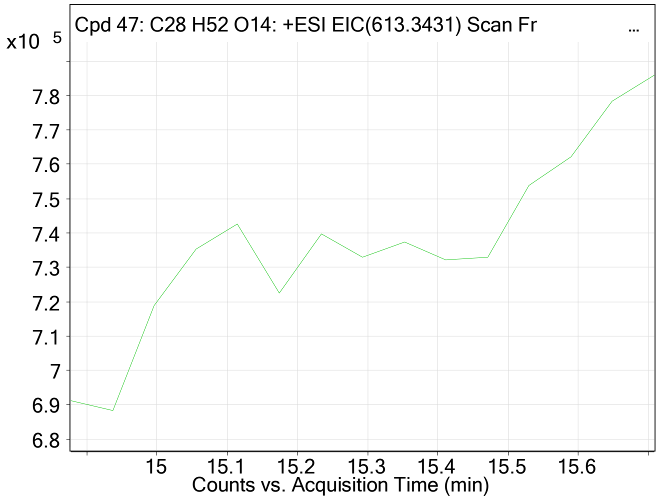

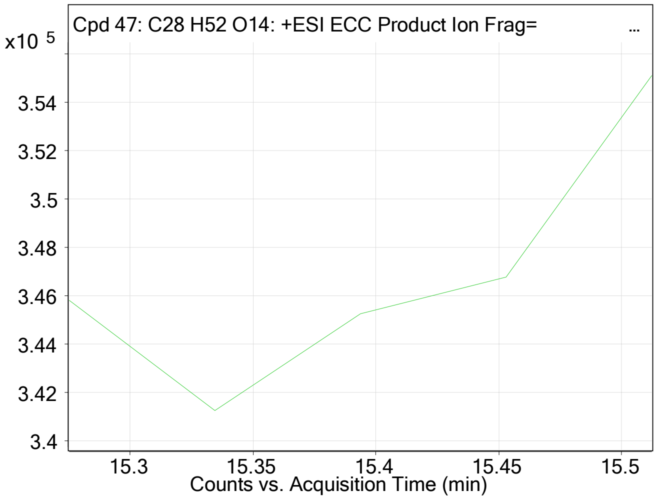

MS Spectrum

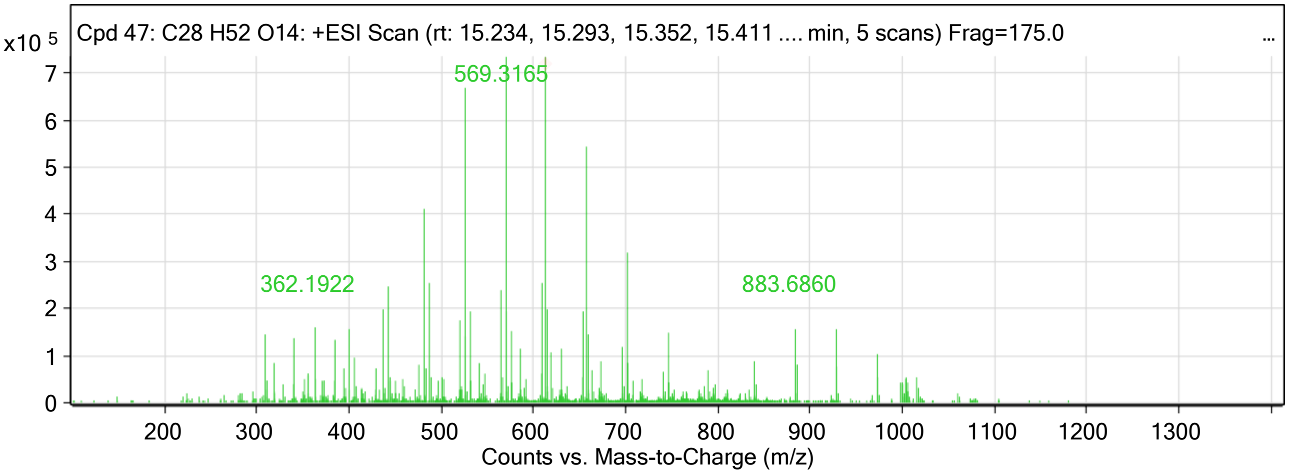

MS Zoomed Spectrum

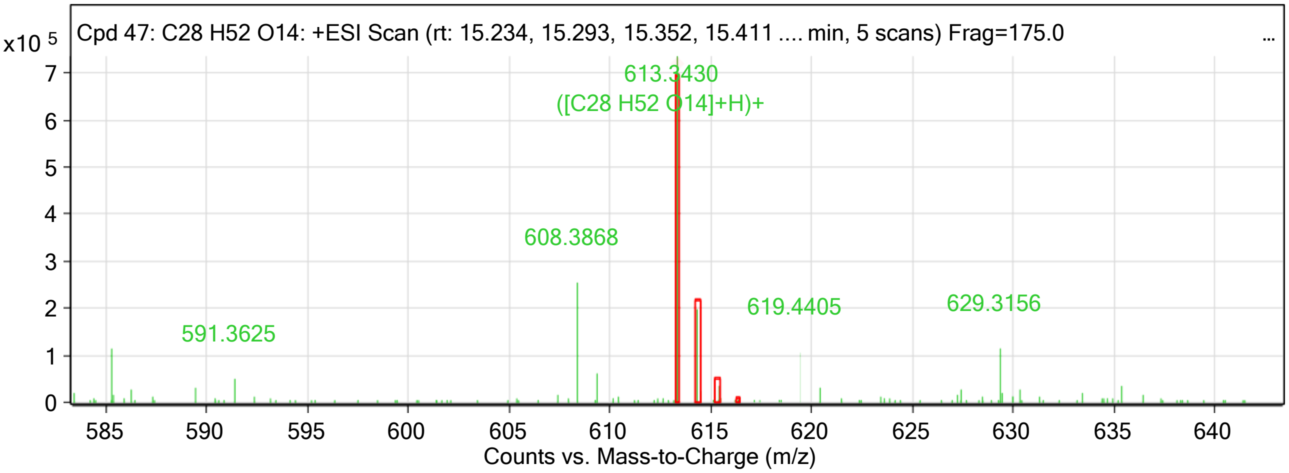

MS Spectrum Peak List

m/z	Calc *m/z*	Diff(ppm)	z	Abund	Formula	Ion
481.2632			1	407922.44		
525.2903			1	666210.88		
569.3165			1	820530.88		
608.3868			1	254049.59		
613.343	613.343	-0.06	1	735065.94	C28 H52 O14	(M+H)+
614.3454	614.3464	1.56	1	196777.53	C28 H52 O14	(M+H)+
615.3475	615.3489	2.26	1	35712.72	C28 H52 O14	(M+H)+
616.3559	616.3515	-6.99	1	5151.23	C28 H52 O14	(M+H)+
657.3689			1	541646.69		
701.395			1	318881		

MSMS Spectrum

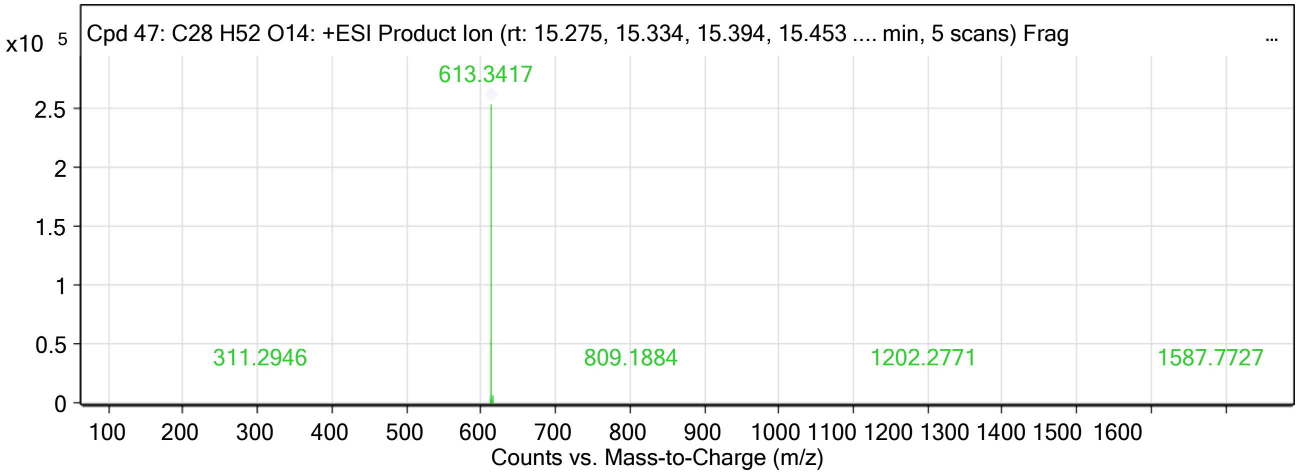

MS/MS Spectrum Peak List

m/z	*Calc m/z*	Diff(ppm)	z	Abund
133.0854	133.0859	3.86		51.01
309.2794	309.2788	-1.78	1	52.38
357.0692	357.0664	-7.93	1	30.52
609.3105	609.3117	1.97	1	103.15
612.6382				3368.23
612.8891			2	2842.77
613.3417	613.343	2.1	1	253925.75
614.3444			1	53420.11
614.4832				5713.85
615.347			1	6514.62

Compound Label	*m/z*	RT	Algorithm	Mass
Cpd 48: C26 H48 O13	569.3168	15.496	Auto MS/MS	568.3093

Compound Chromatograms

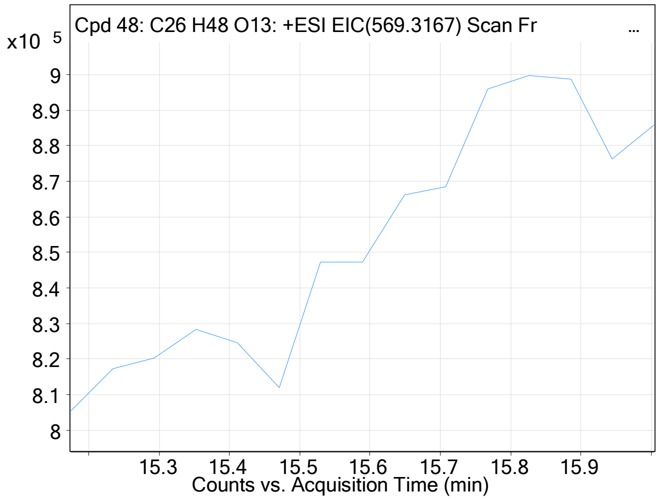

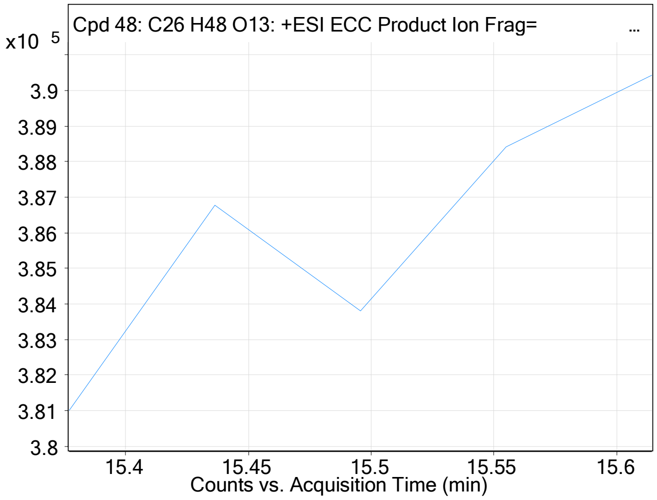

MS Spectrum

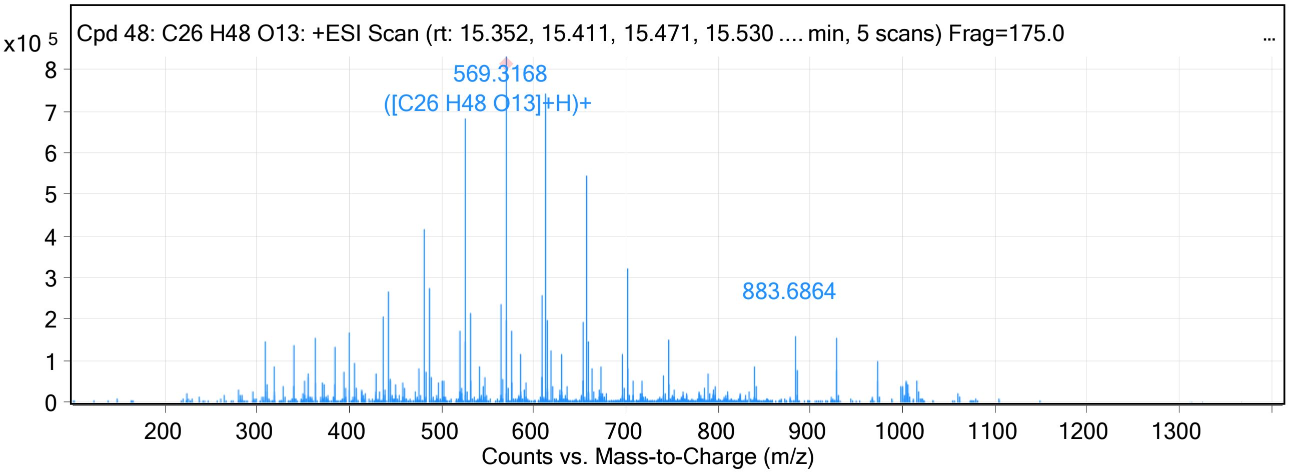

MS Zoomed Spectrum

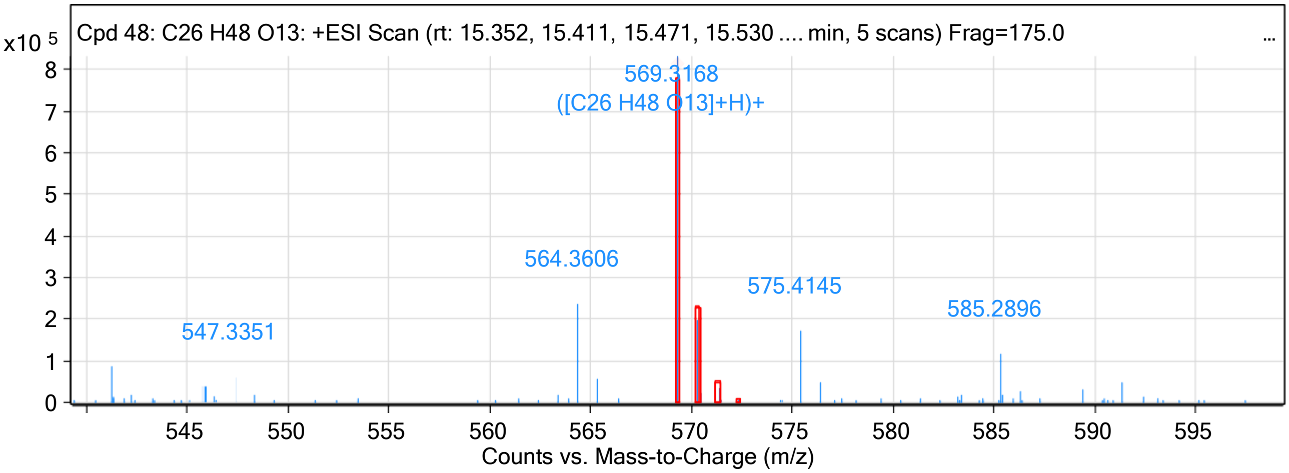

MS Spectrum Peak List

m/z	Calc *m/z*	Diff(ppm)	z	Abund	Formula	Ion
481.2634			1	417243.47		
487.362			1	273161.56		
525.2905			1	680519.88		
569.3168	569.3168	0	1	831946.94	C26 H48 O13	(M+H)+
570.3193	570.3202	1.52	1	196328.72	C26 H48 O13	(M+H)+
571.3215	571.3226	1.9	1	33250.46	C26 H48 O13	(M+H)+
572.3244	572.3253	1.52	1	4757.53	C26 H48 O13	(M+H)+
613.3433			1	743740.94		
657.3692			1	545162.56		
701.3952			1	320518.19		

MSMS Spectrum

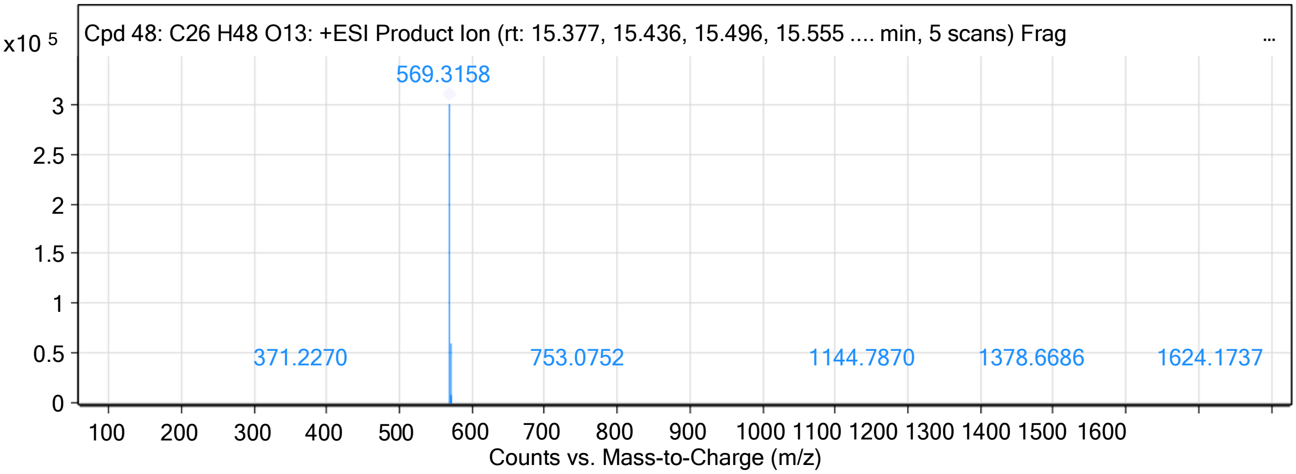

MS/MS Spectrum Peak List

m/z	Calc *m/z*	Diff(ppm)	z	Abund
117.0908	117.091	1.44		19.4
133.086	133.0859	-0.41		101.36
139.1468	139.1481	9.29		15.86
166.1292	166.1352	35.95		19.29
177.1119	177.1121	1.41		21.7
307.2164	307.2115	-15.87		15
309.2807	309.2788	-6.26		45.31
311.2943	311.2945	0.41	1	57.72
566.2893	566.2933	7.11	1	37.05
569.3158	569.3168	1.77	1	300159.47

Compound Label	*m/z*	RT	Algorithm	Mass
Cpd 49: C28 H52 O14	613.3431	15.69	Auto MS/MS	612.3356

Compound Chromatograms

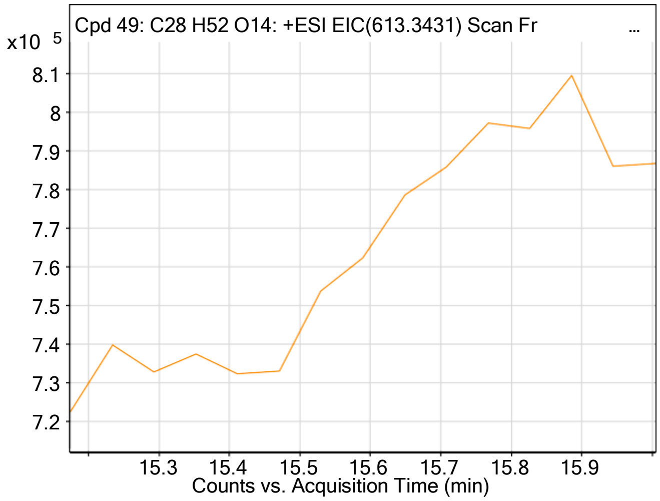

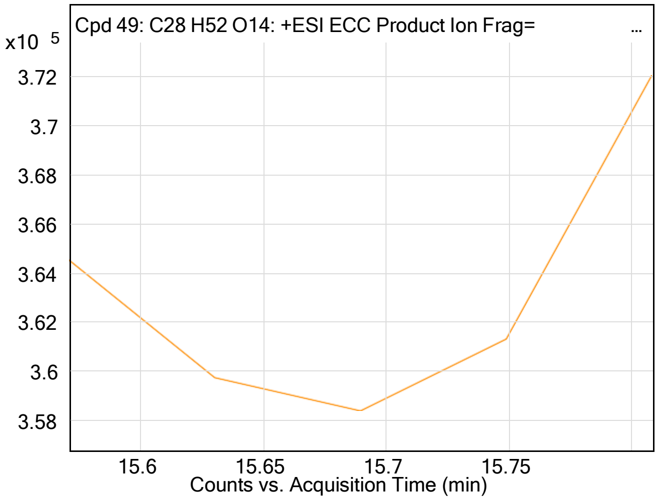

MS Spectrum

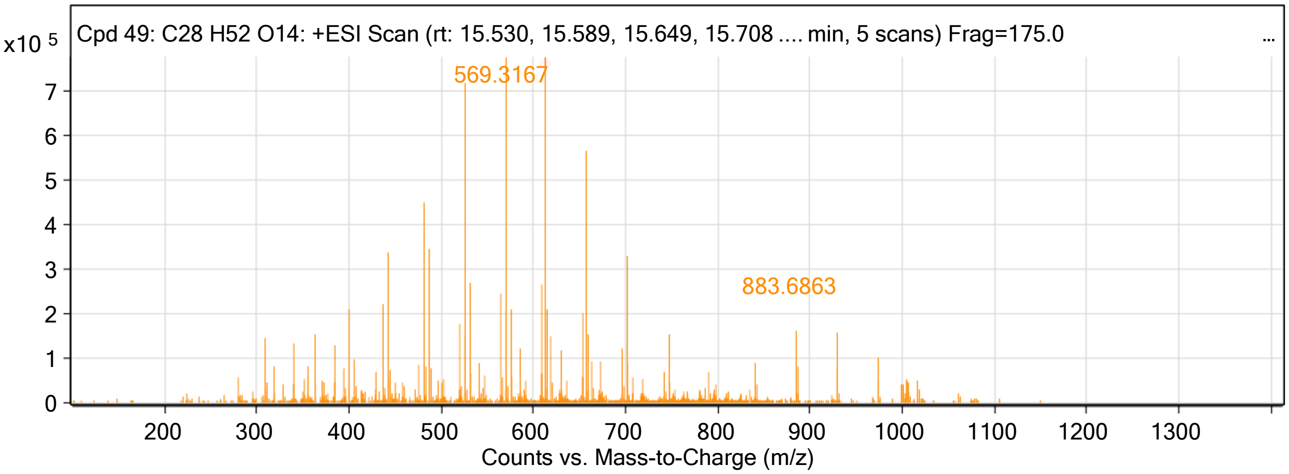

MS Zoomed Spectrum

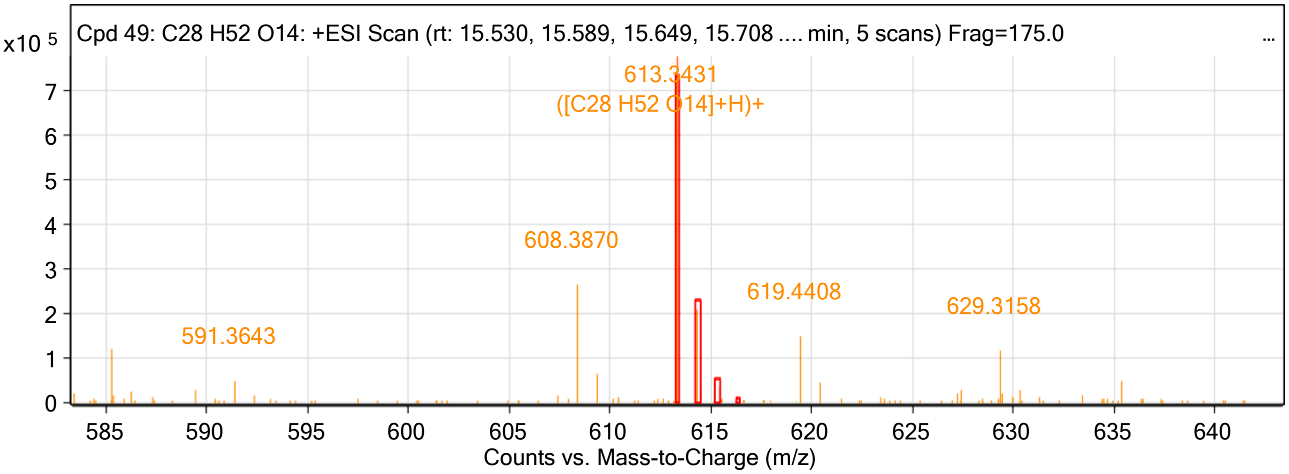

MS Spectrum Peak List

m/z	Calc *m/z*	Diff(ppm)	z	Abund	Formula	Ion
443.3356			1	335055.09		
481.2634			1	446216.91		
487.3621			1	342141.78		
525.2904			1	713908		
569.3167			1	865051.5		
613.3431	613.343	-0.22	1	775513.63	C28 H52 O14	(M+H)+
614.3457	614.3464	1.2	1	206977.95	C28 H52 O14	(M+H)+
615.3477	615.3489	1.92	1	37917.46	C28 H52 O14	(M+H)+

MSMS Spectrum

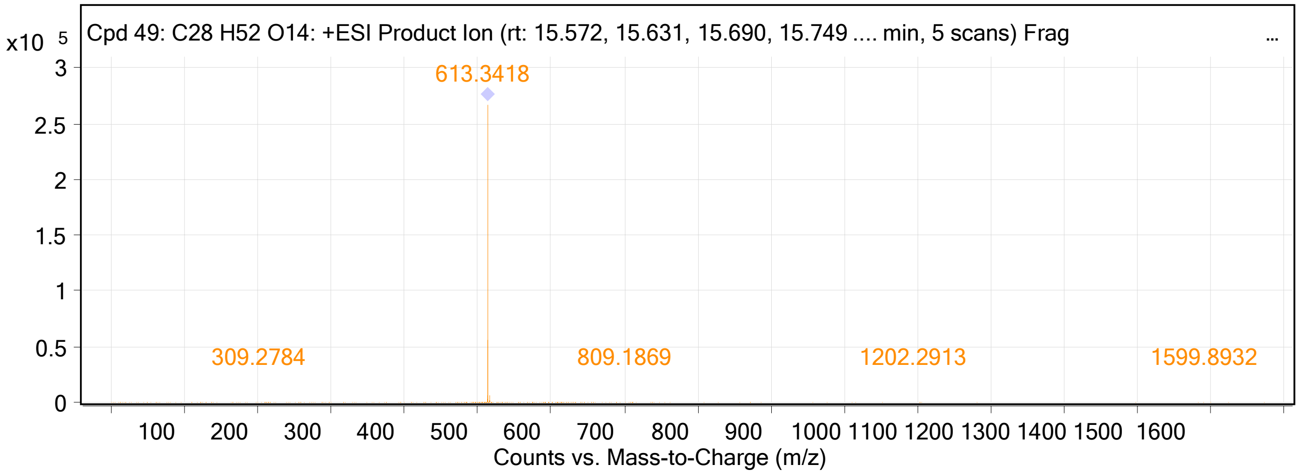

MS/MS Spectrum Peak List

m/z	*Calc m/z*	Diff(ppm)	z	Abund
133.0872	133.0859	-9.72		57.38
177.1128	177.1121	-3.77		36.55
309.2784	309.2788	1.45	1	108.52
357.0688	357.0664	-6.68	1	55.14
609.3111	304.6556	-499999.98	2	57.17
612.6383			2	3323.54
613.3418	613.343	1.85	1	267461.31
614.3446			1	56245.45
614.4835			1	6498.9
615.3469			1	6528.05

Compound Label	*m/z*	RT	Algorithm	Mass
Cpd 50: C24 H46 N3 O12	569.3165	15.792	Auto MS/MS	568.3091

Compound Chromatograms

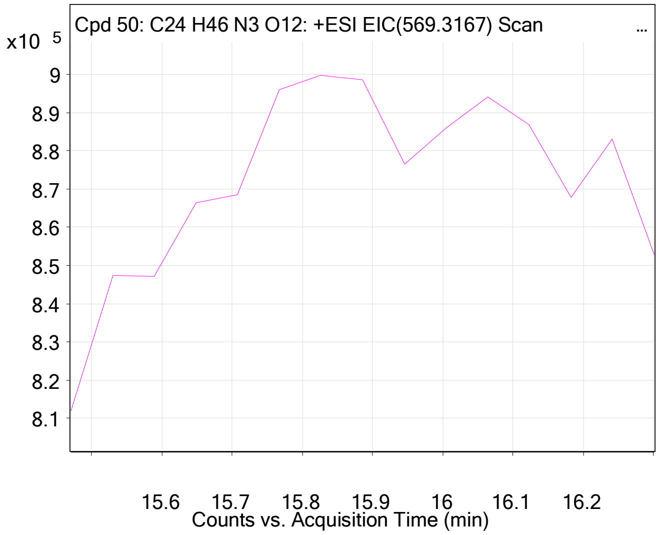

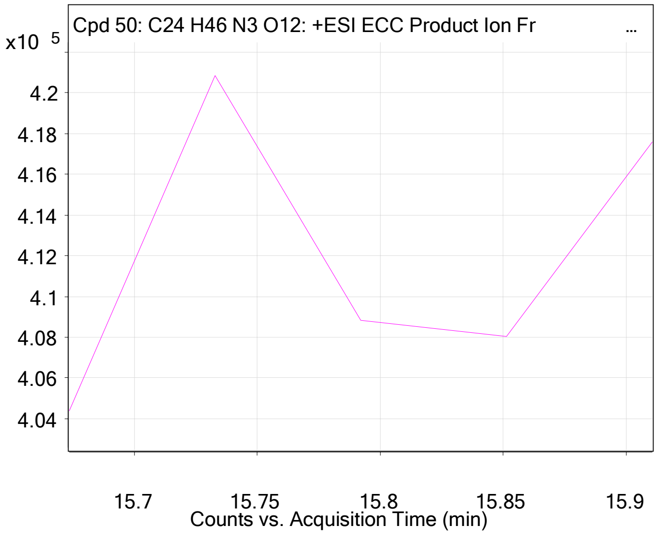

MS Spectrum

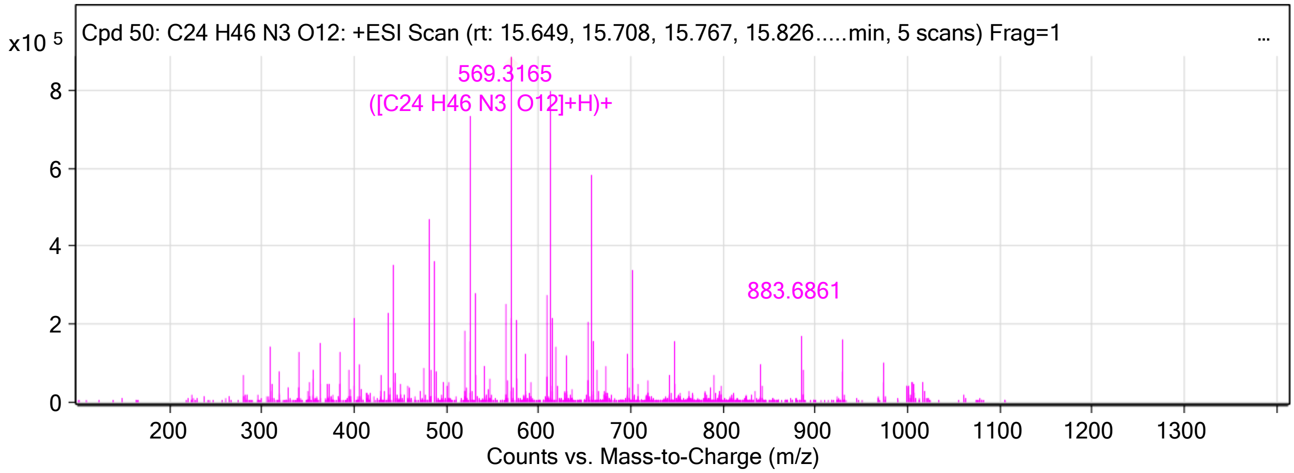

MS Zoomed Spectrum

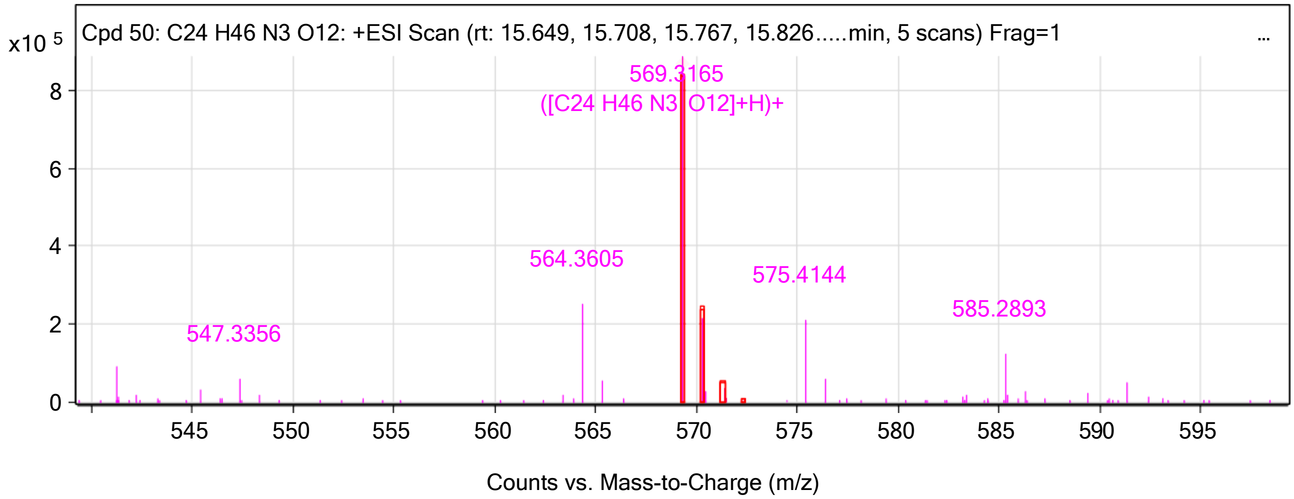

MS Spectrum Peak List

m/z	Calc *m/z*	Diff(ppm)	z	Abund	Formula	Ion
443.3355			1	353760.66		
481.2632			1	467767.5		
487.362			1	360804.44		
525.2903			1	731222.13		
569.3165	569.3154	-1.83	1	885798.38	C24 H46 N3 O12	(M+H)+
570.3191	570.3186	-0.88	1	212951.97	C24 H46 N3 O12	(M+H)+
571.3211	571.3209	-0.31	1	35697.48	C24 H46 N3 O12	(M+H)+
572.3241	572.3235	-0.98	1	4973.69	C24 H46 N3 O12	(M+H)+
613.3429			1	793334.38		
657.3689			1	578116.63		

MSMS Spectrum

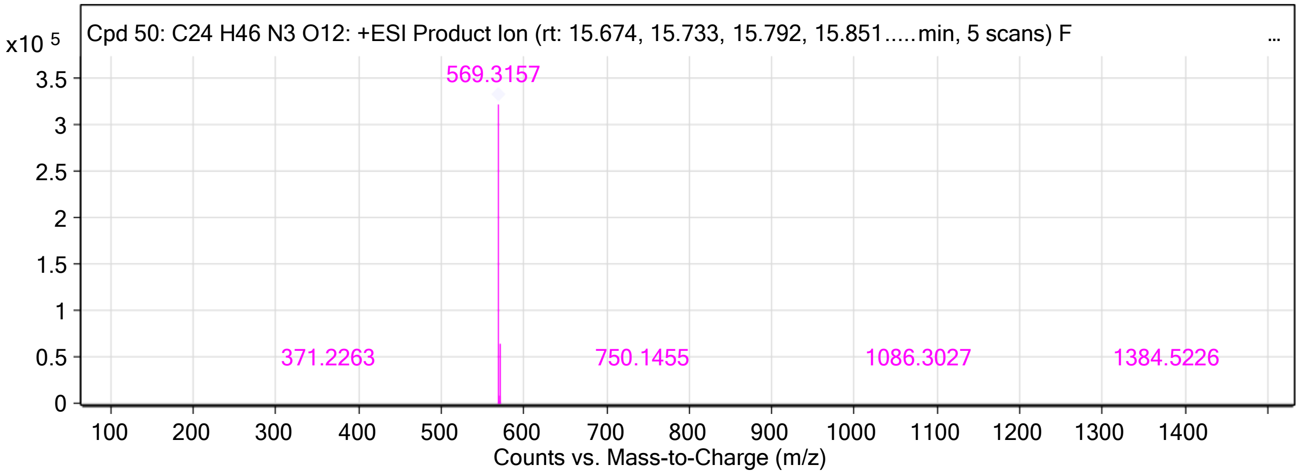

MS/MS Spectrum Peak List

m/z	*Calc m/z*	Diff(ppm)	z	Abund
117.0921	117.091	-9.46		19.21
133.0857	133.0859	1.55	1	60.26
177.1114	177.1108	-3.35		30.96
195.1221	195.1214	-3.98		18.6
309.2802	309.2788	-4.61		30.04
328.268	328.272	12.25		14.63
334.1882	334.186	-6.52		15.42
371.2263	371.2262	-0.13	1	59.79
411.2447	411.2463	3.89		19.6
569.3157	569.3154	-0.44	1	321618.25

Compound Label	*m/z*	RT	Algorithm	Mass
Cpd 51: C28 H52 O14	613.343	15.987	Auto MS/MS	612.3356

Compound Chromatograms

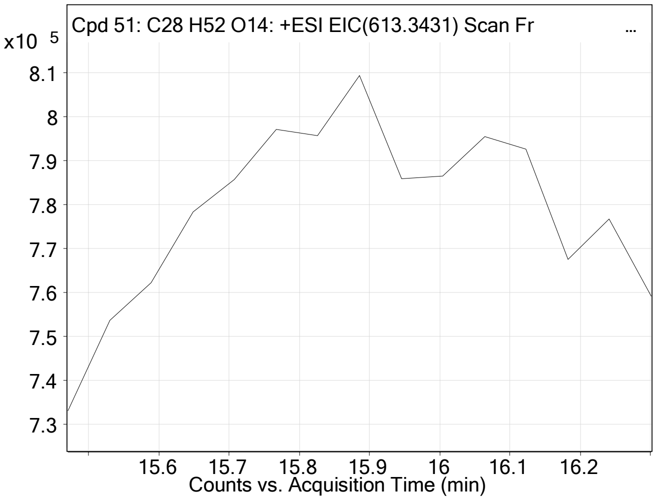

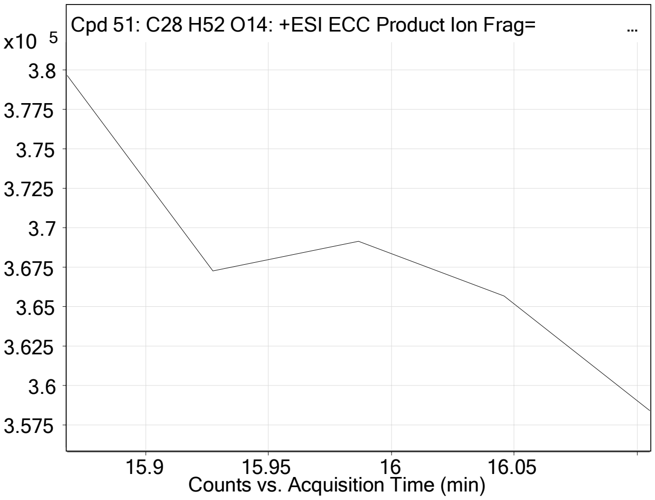

MS Spectrum

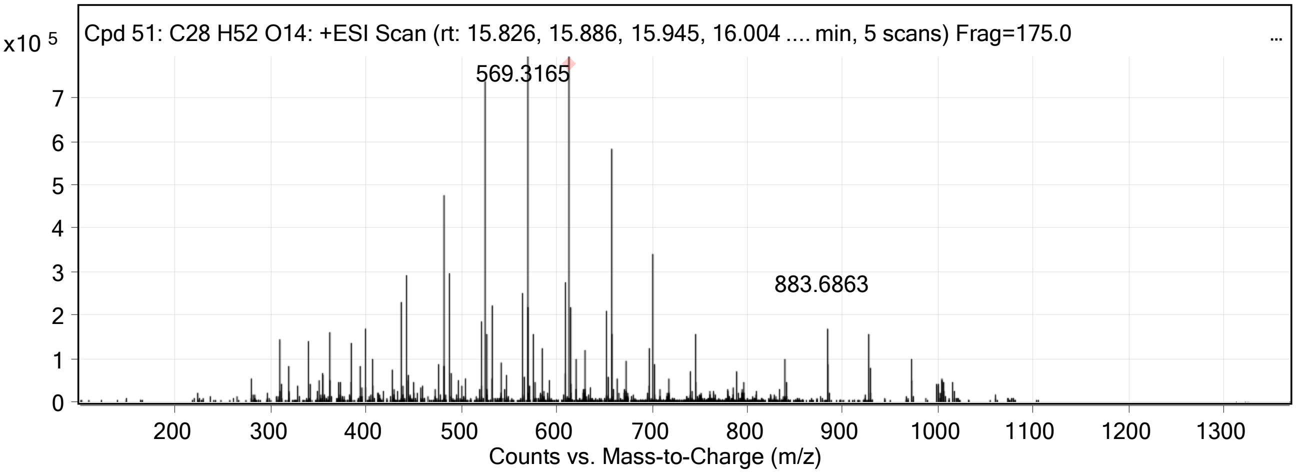

MS Zoomed Spectrum

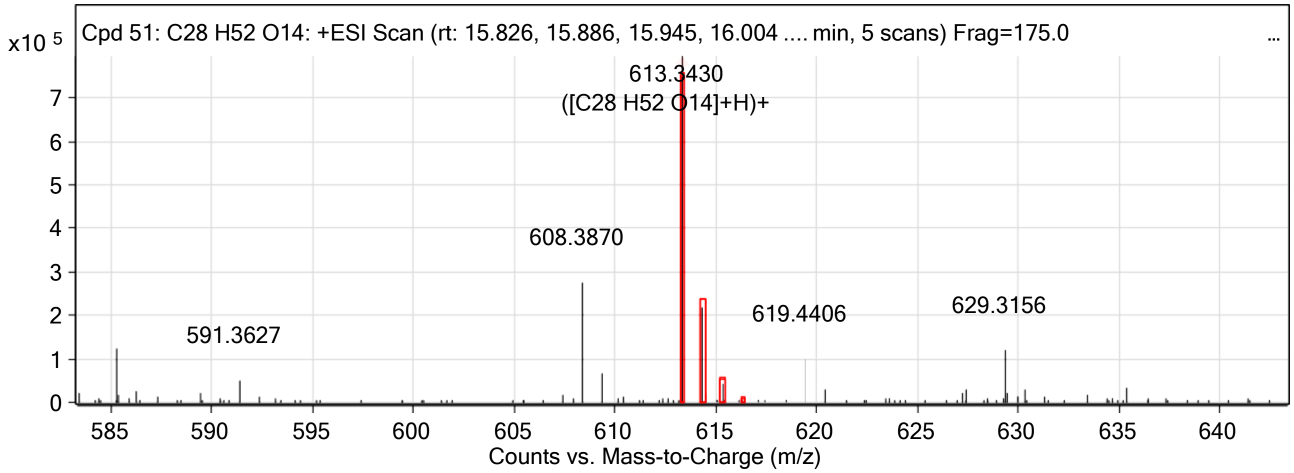

MS Spectrum Peak List

m/z	Calc *m/z*	Diff(ppm)	z	Abund	Formula	Ion
481.2633			1	475306.41		
487.362			1	297032.56		
525.2904			1	736537.88		
569.3165			1	890998.69		
613.343	613.343	-0.09	1	794630.63	C28 H52 O14	(M+H)+
614.3457	614.3464	1.21	1	216073.16	C28 H52 O14	(M+H)+
615.3476	615.3489	2.1	1	39191.81	C28 H52 O14	(M+H)+
616.3562	616.3515	-7.62	1	5441.25	C28 H52 O14	(M+H)+
657.369			1	581532.56		
701.3952			1	341566.03		

MSMS Spectrum

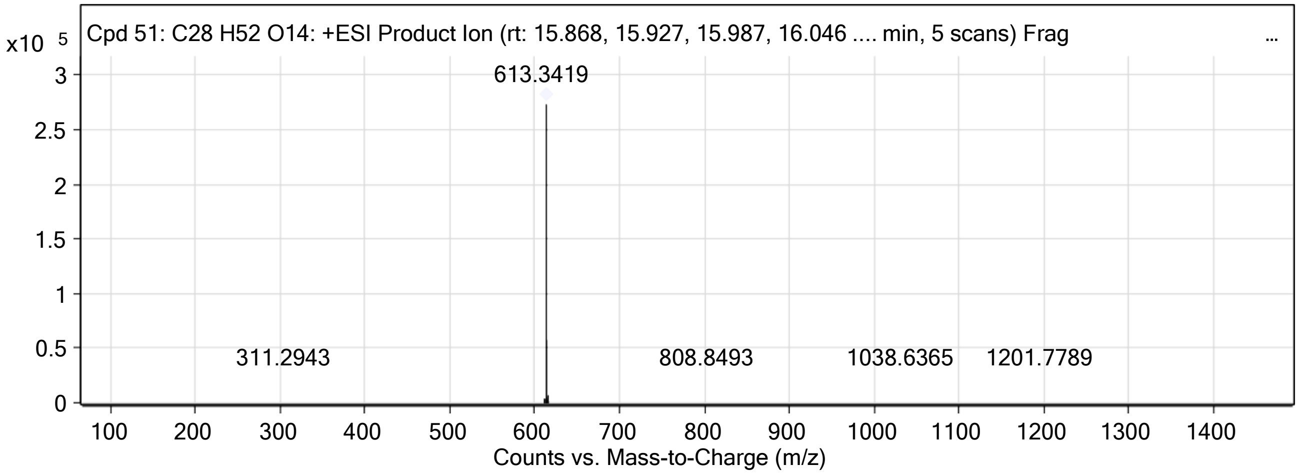

MS/MS Spectrum Peak List

m/z	*Calc m/z*	Diff(ppm)	z	Abund
133.0861	133.0859	-1.49		92.79
309.2769	309.2788	6.26	1	63.57
609.3181	609.3117	-10.59		56.38
612.3879			2	2905.51
612.6388				3381.46
612.8897			2	2849.39
613.3419	613.343	1.73	1	273187.53
614.3447			1	56952.87
614.4833				4237.55
615.347			1	6993.34

Compound Label	*m/z*	RT	Algorithm	Mass
Cpd 52: C24 H46 N3 O12	569.3165	16.089	Auto MS/MS	568.3092

Compound Chromatograms

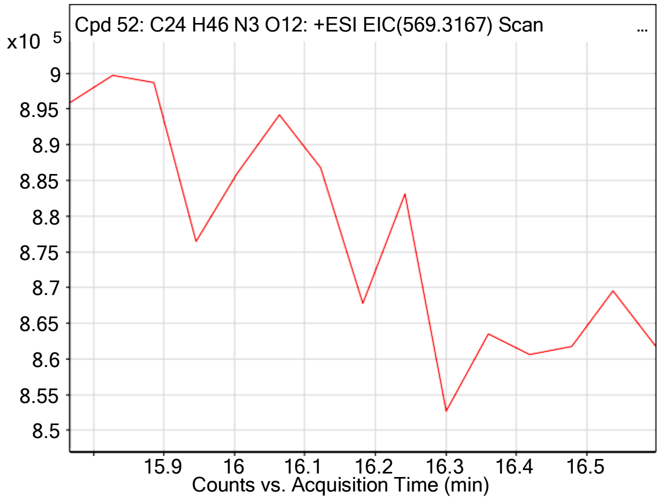

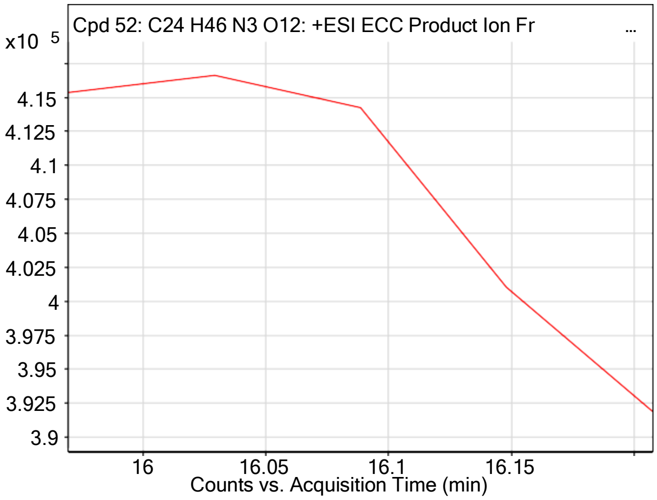

MS Spectrum

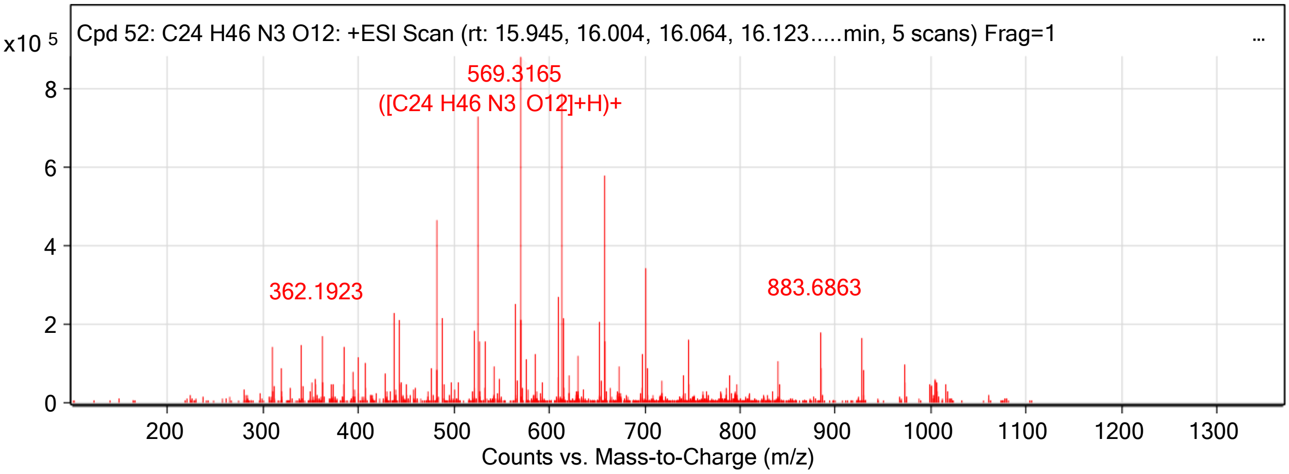

MS Zoomed Spectrum

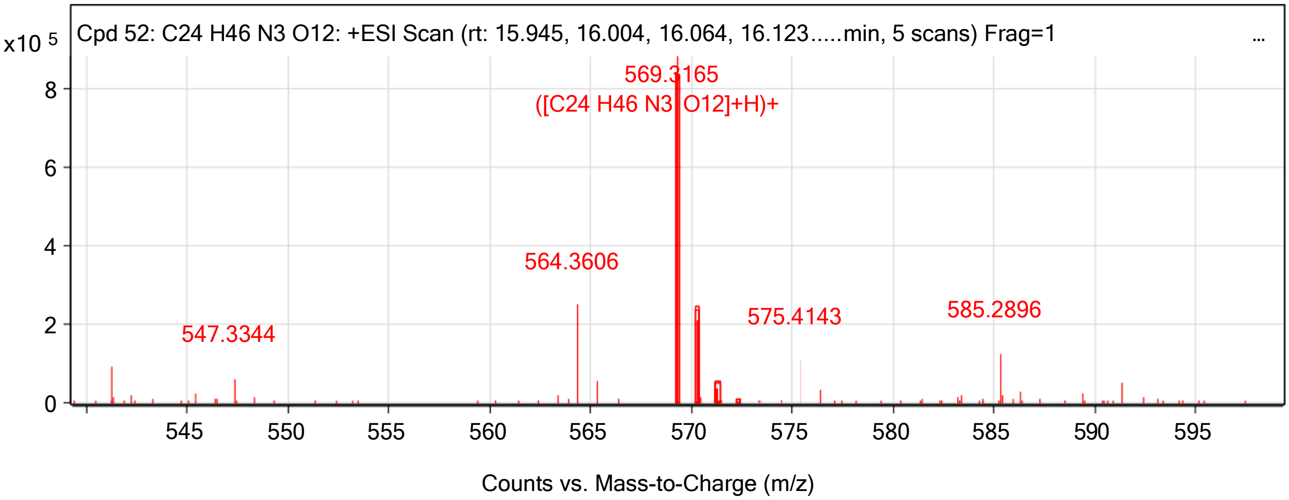

MS Spectrum Peak List

m/z	Calc *m/z*	Diff(ppm)	z	Abund	Formula	Ion
481.2634			1	462339.84		
525.2904			1	727025.63		
569.3165	569.3154	-1.96	1	882234.88	C24 H46 N3 O12	(M+H)+
570.3192	570.3186	-1.13	1	211549.2	C24 H46 N3 O12	(M+H)+
571.3214	571.3209	-0.85	1	35876.45	C24 H46 N3 O12	(M+H)+
572.3243	572.3235	-1.29	1	5218.18	C24 H46 N3 O12	(M+H)+
608.387			1	269149.22		
613.3431			1	785671.31		

MSMS Spectrum

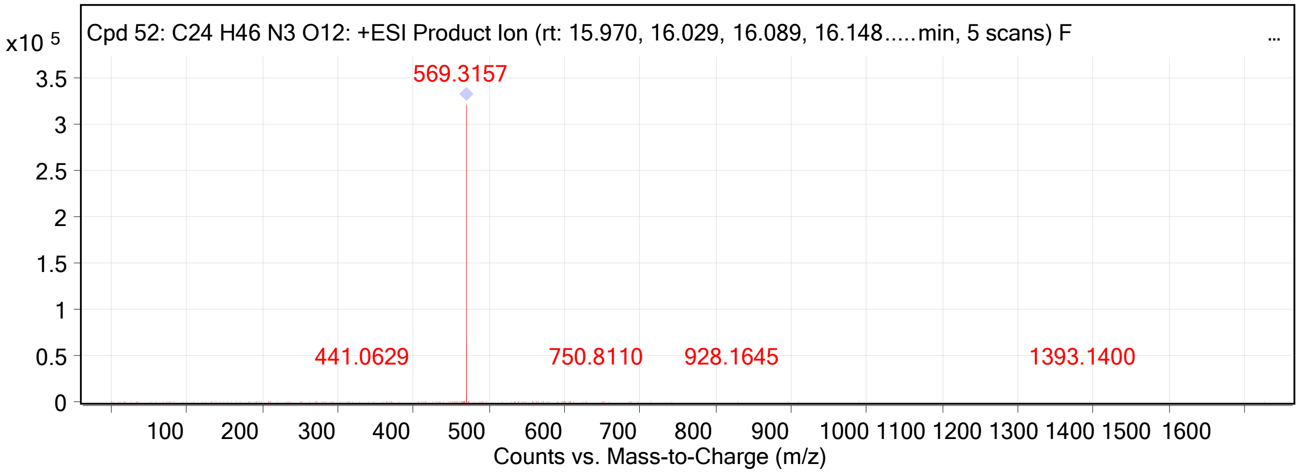

MS/MS Spectrum Peak List

m/z	*Calc m/z*	Diff(ppm)	z	Abund
101.0959	101.0961	1.77		18.97
133.0869	133.0859	-7.13		33.16
151.0972	151.0992	12.87		26.14
309.2779	309.2775	-1.54	1	30.63
370.233	370.2336	1.74		18.53
371.2258	371.2262	1.12		31.85
372.2323	372.234	4.66		25.81
394.2611	394.2561	-12.51		17.25
566.2904	283.1457	-499999.1	2	25.23
569.3157	569.3154	-0.57	1	321031.28

Compound Label	*m/z*	RT	Algorithm	Mass
Cpd 53: C28 H52 O14	613.3432	16.283	Auto MS/MS	612.3357

Compound Chromatograms

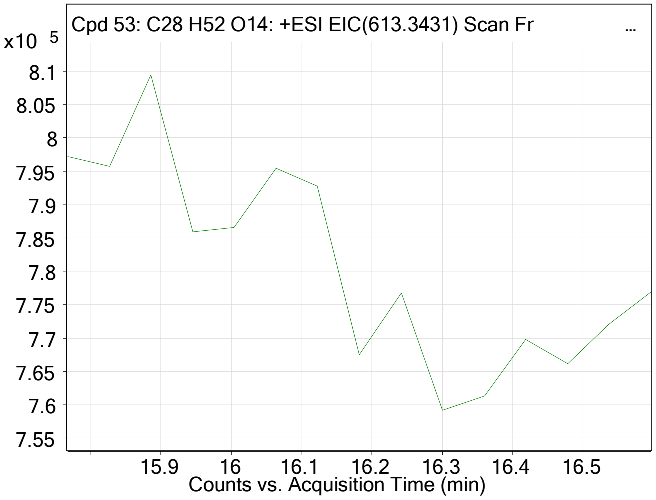

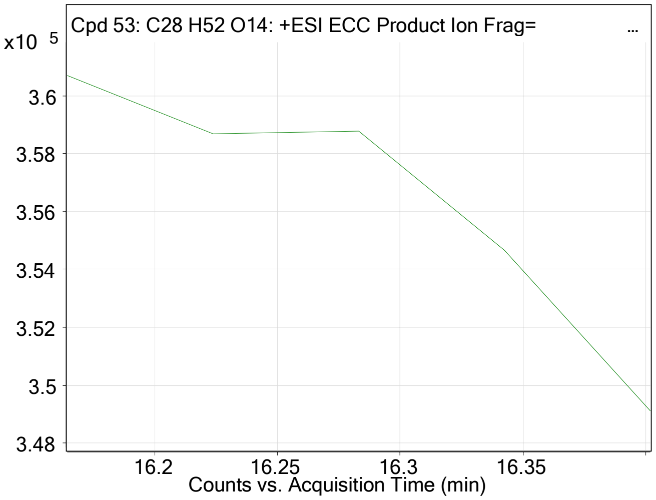

MS Spectrum

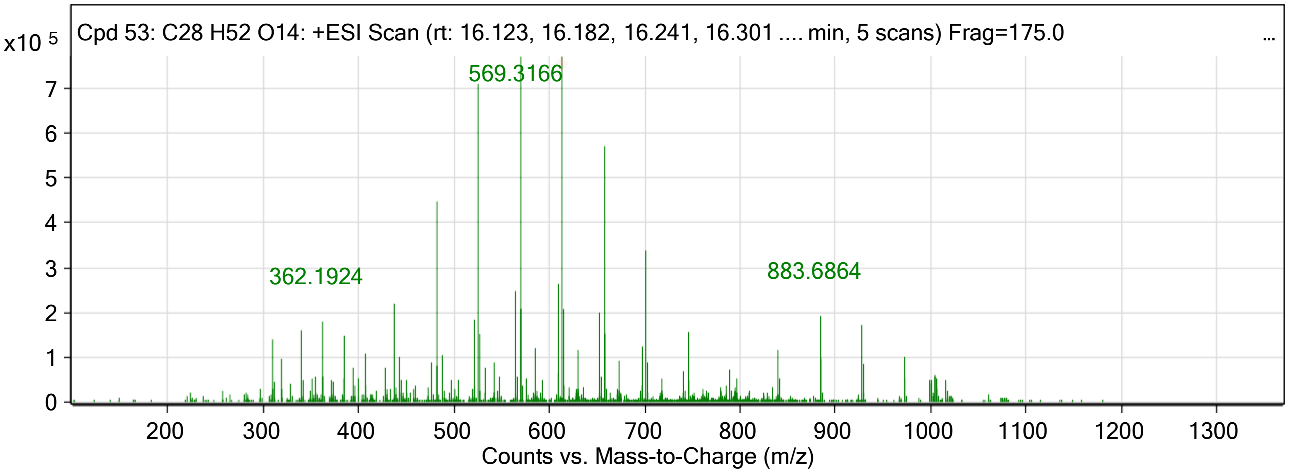

MS Zoomed Spectrum

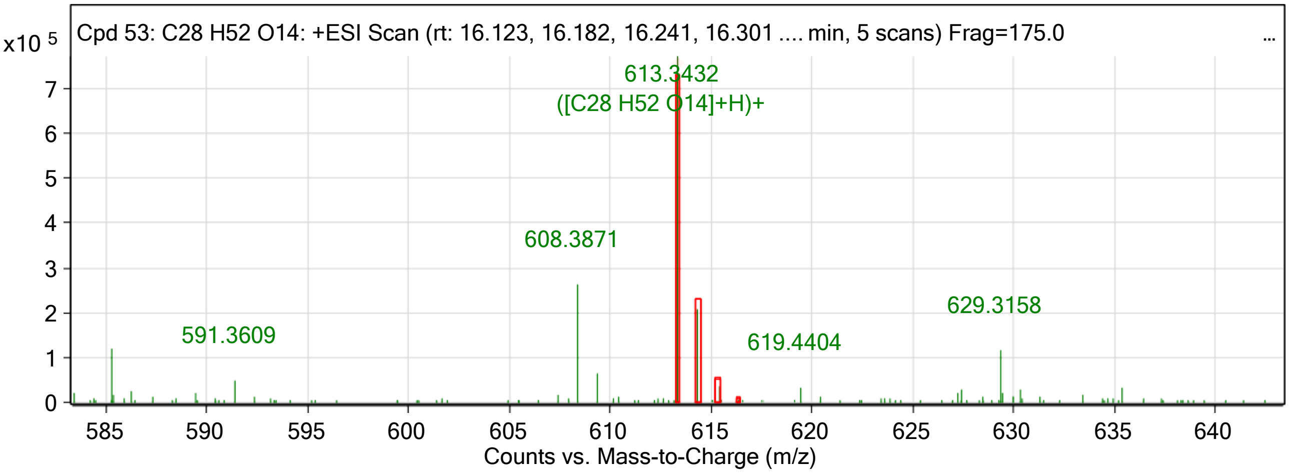

MS Spectrum Peak List

m/z	Calc *m/z*	Diff(ppm)	z	Abund	Formula	Ion
481.2634			1	445991		
525.2905			1	708394.5		
569.3166			1	870778.69		
608.3871			1	263772.59		
613.3432	613.343	-0.28	1	771518.06	C28 H52 O14	(M+H)+
614.3457	614.3464	1.21	1	209016.72	C28 H52 O14	(M+H)+
615.3478	615.3489	1.78	1	37182.54	C28 H52 O14	(M+H)+
616.3557	616.3515	-6.72	1	5346.43	C28 H52 O14	(M+H)+
657.3692			1	568138.38		
701.3952			1	336483.91		

MSMS Spectrum

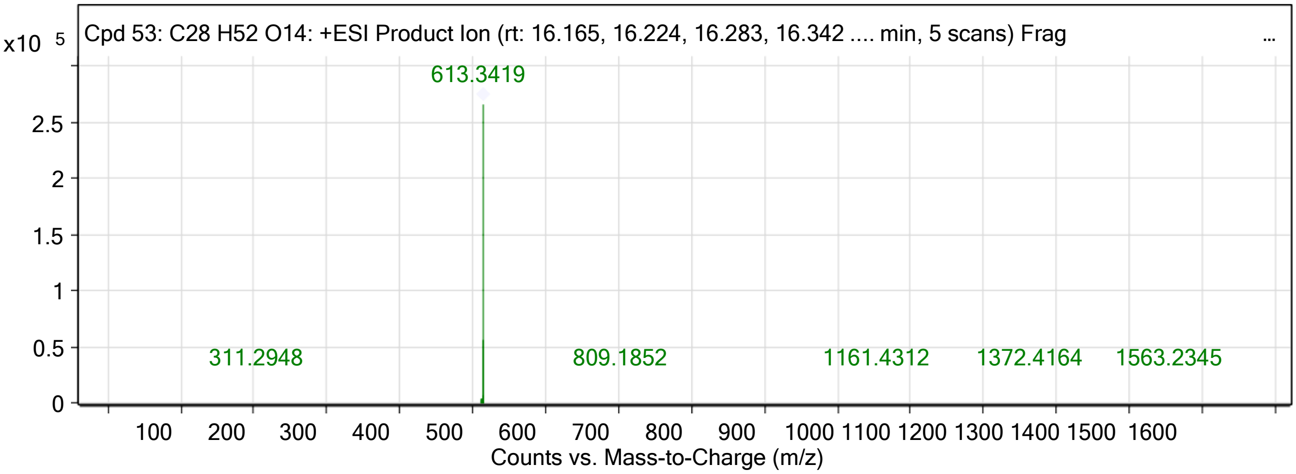

MS/MS Spectrum Peak List

m/z	*Calc m/z*	Diff(ppm)	z	Abund
133.0876	133.0859	-12.63	1	90.7
309.2791	309.2788	-1.03	1	59.55
612.3877			2	2841.78
612.6387				3837.07
612.8895			2	3112.13
613.1408			1	1445.73
613.3419	613.343	1.74	1	265418.59
614.3447			1	55271.11
615.1403			1	1147.04
615.347			1	7179.27

Compound Label	*m/z*	RT	Algorithm	Mass
Cpd 54: C24 H46 N3 O12	569.3167	16.385	Auto MS/MS	568.3093

Compound Chromatograms

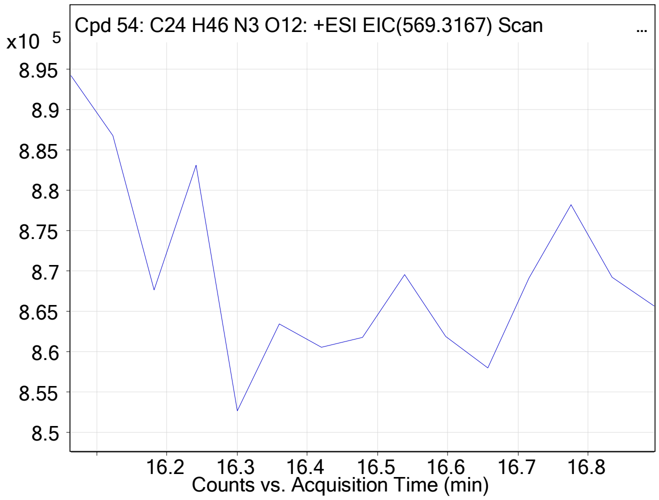

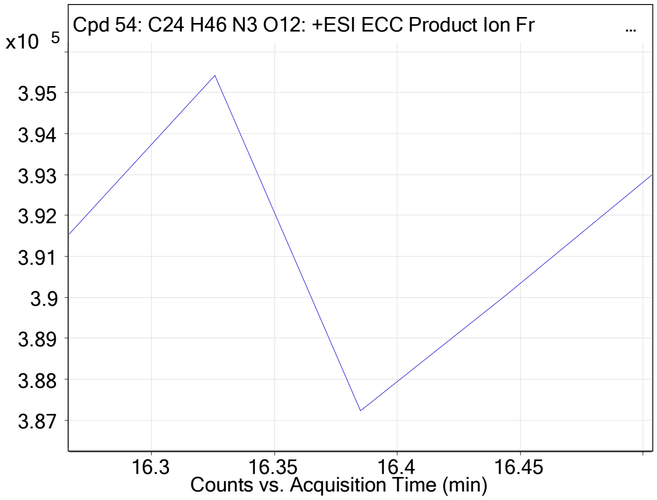

MS Spectrum

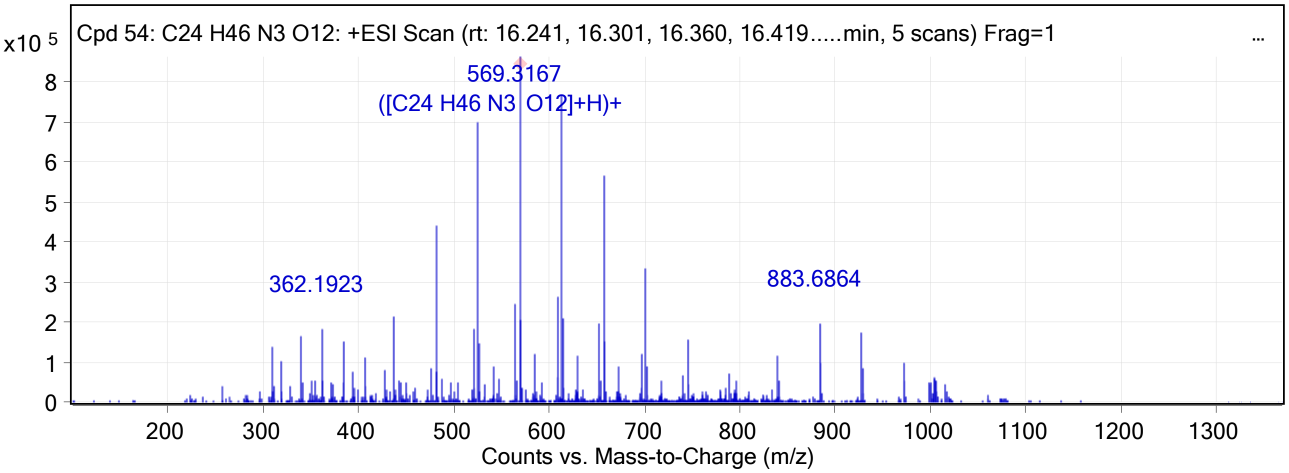

MS Zoomed Spectrum

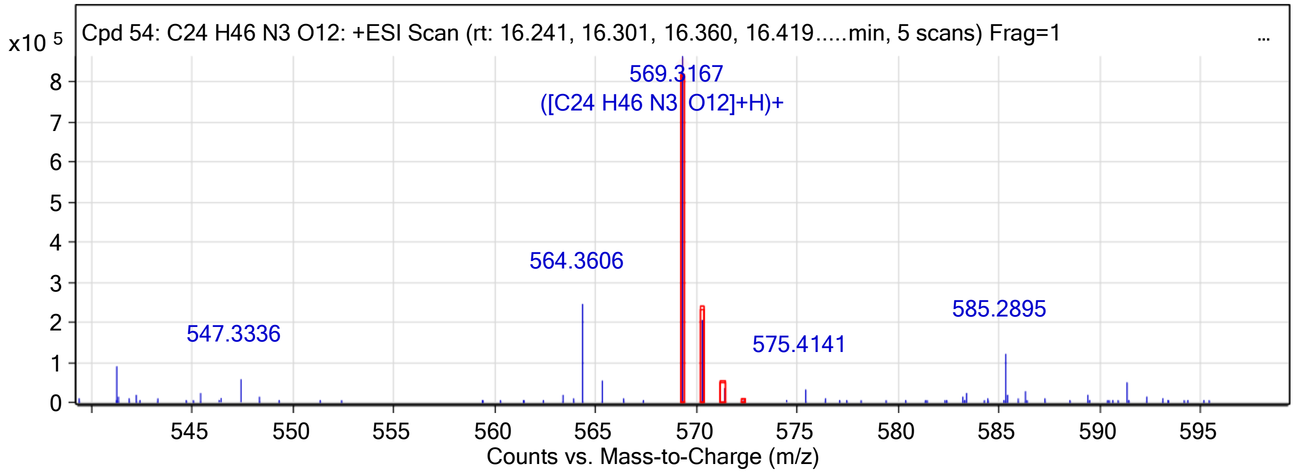

MS Spectrum Peak List

m/z	Calc *m/z*	Diff(ppm)	z	Abund	Formula	Ion
481.2633			1	439920.75		
525.2905			1	701579.63		
569.3167	569.3154	-2.19	1	864348.13	C24 H46 N3 O12	(M+H)+
570.3192	570.3186	-1.13	1	205308.03	C24 H46 N3 O12	(M+H)+
571.3213	571.3209	-0.6	1	34019.66	C24 H46 N3 O12	(M+H)+
572.3245	572.3235	-1.69	1	4779.05	C24 H46 N3 O12	(M+H)+
608.387			1	264480.69		
613.3431			1	766640.69		
657.3691			1	565226.31		
701.3952			1	335125.47		

MSMS Spectrum

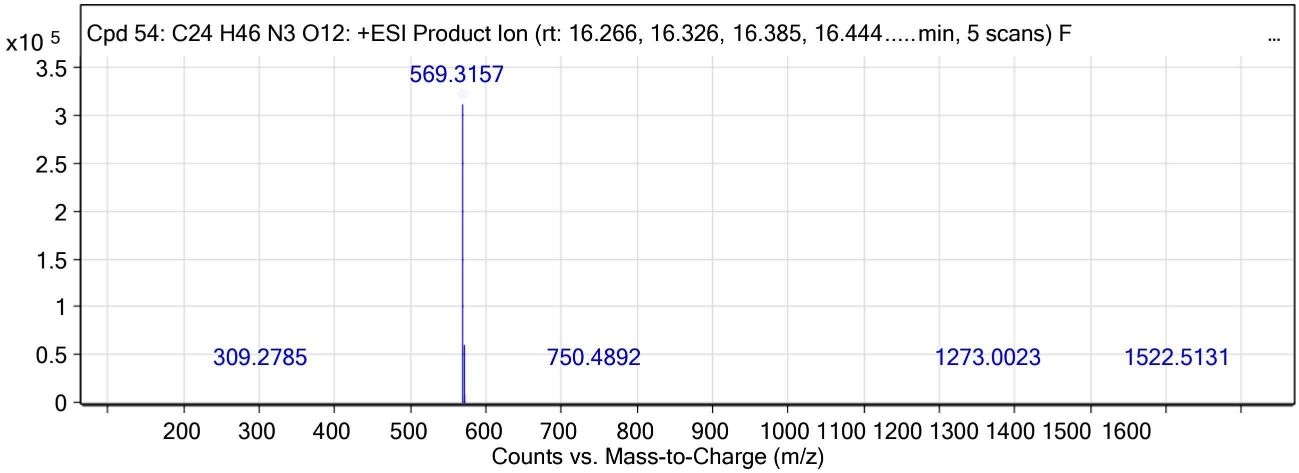

MS/MS Spectrum Peak List

m/z	Calc *m/z*	Diff(ppm)	z	Abund
151.0976	151.0992	10.29		15.02
175.1345	175.1356	5.73		17.34
177.1113	177.1108	-3.04		18.63
210.119	210.121	9.84		15.11
275.1594	275.1601	2.54		14.19
309.2785	309.2788	1.08		47.83
336.2169	336.2143	-8.01		13.44
371.2259	371.2262	0.86		43.66
428.257	428.2603	7.5		12.42
569.3157	569.3154	-0.44	1	311949.34

Compound Label	*m/z*	RT	Algorithm	Mass
Cpd 55: C26 H50 N3 O13	613.3428	16.58	Auto MS/MS	612.3354

Compound Chromatograms

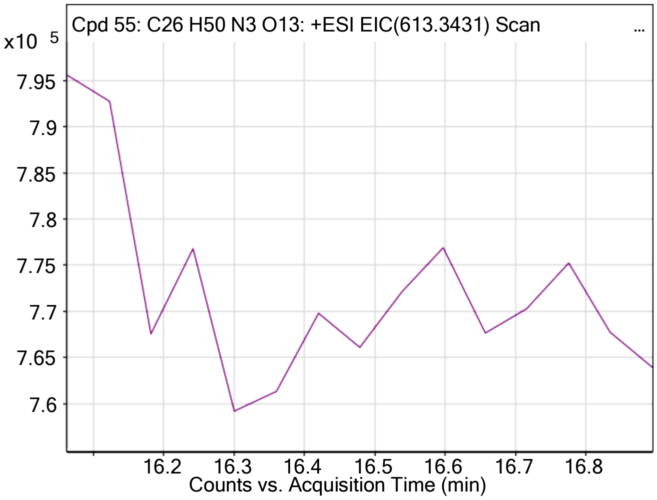

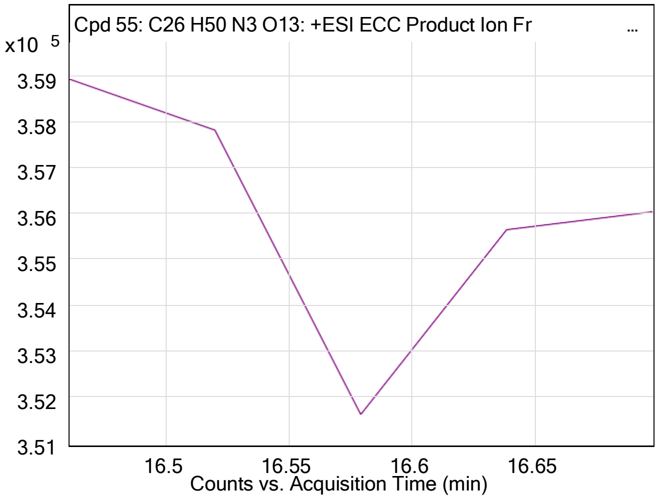

MS Spectrum

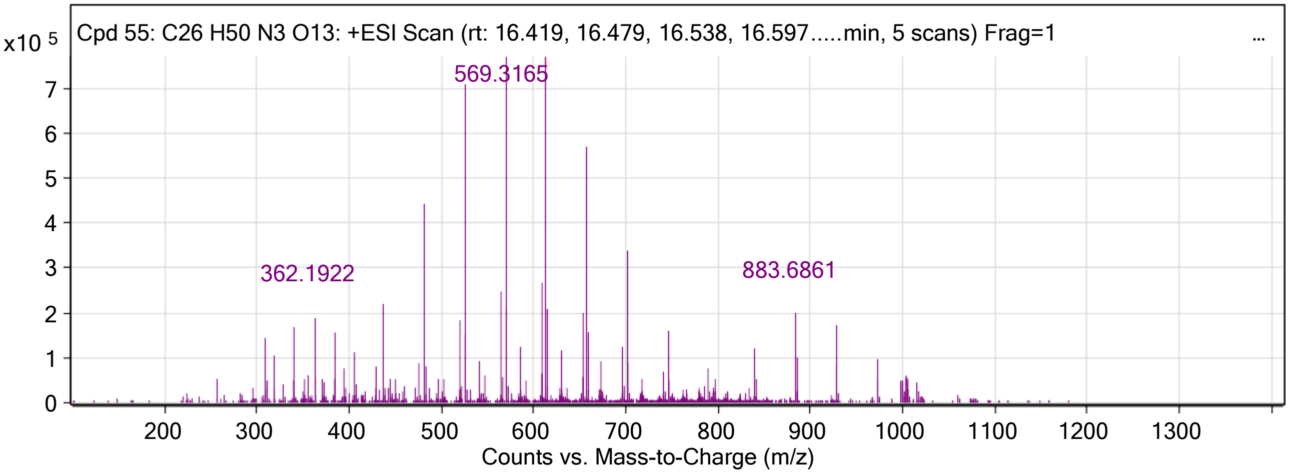

MS Zoomed Spectrum

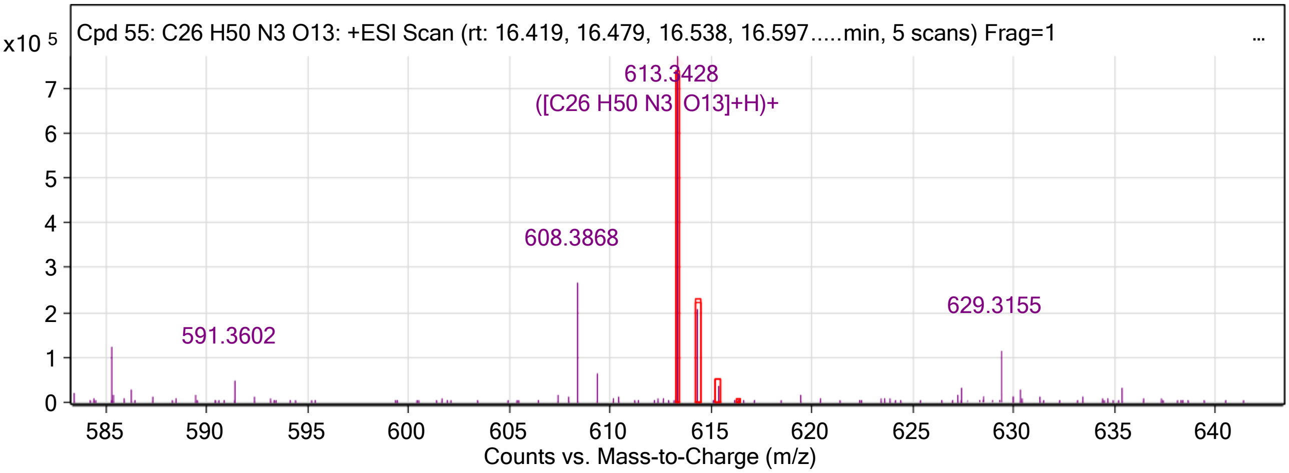

MS Spectrum Peak List

m/z	Calc *m/z*	Diff(ppm)	z	Abund	Formula	Ion
481.2632			1	441840.56		
525.2903			1	708574.19		
569.3165			1	862372.38		
608.3868			1	266458.53		
613.3428	613.3416	-1.94	1	770506.81	C26 H50 N3 O13	(M+H)+
614.3454	614.3448	-0.91	1	207956.61	C26 H50 N3 O13	(M+H)+
615.3475	615.3472	-0.46	1	37503.39	C26 H50 N3 O13	(M+H)+
616.3576	616.3498	-12.59	1	5637.1	C26 H50 N3 O13	(M+H)+
657.3689			1	568562		

MSMS Spectrum

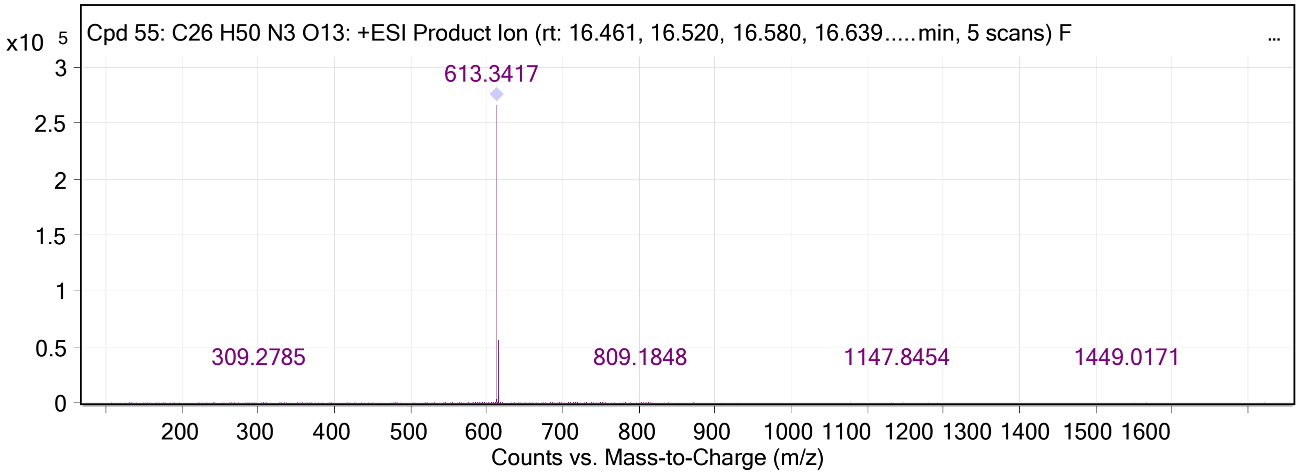

MS/MS Spectrum Peak List

m/z	*Calc m/z*	Diff(ppm)	z	Abund
133.0863	133.0859	-2.9	1	50.93
195.1217	195.1214	-1.62		43.47
309.2785	309.2788	0.91	1	96.8
355.0704	355.0745	11.51		32.52
357.0716	357.0664	-14.66		32.16
612.6379				3868.05
612.8889			2	2943.66
613.3417	613.3416	-0.09	1	266878.38
614.3444			1	56055.98
615.3467			1	6833.52

Compound Label	*m/z*	RT	Algorithm	Mass
Cpd 56: C24 H46 N3 O12	569.3166	16.682	Auto MS/MS	568.3092

Compound Chromatograms

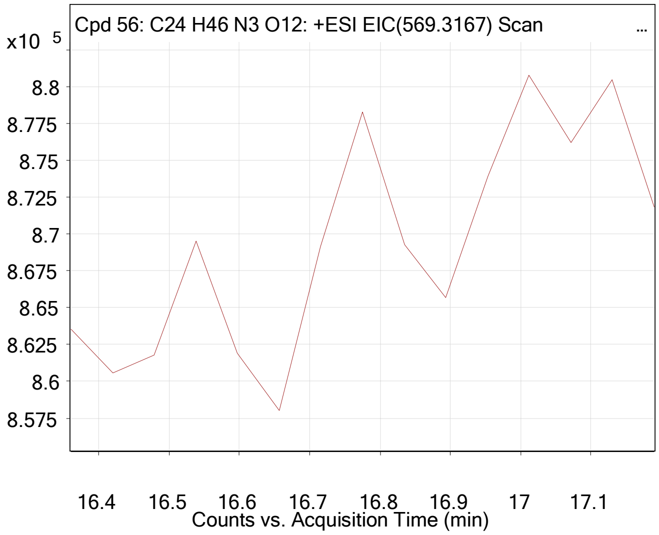

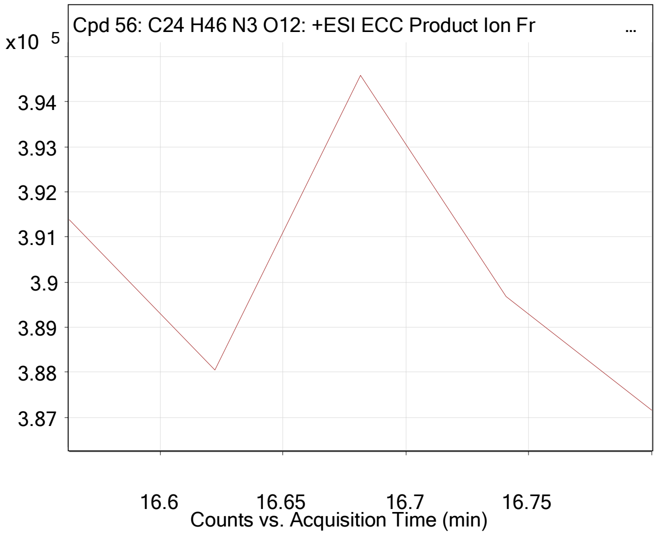

MS Spectrum

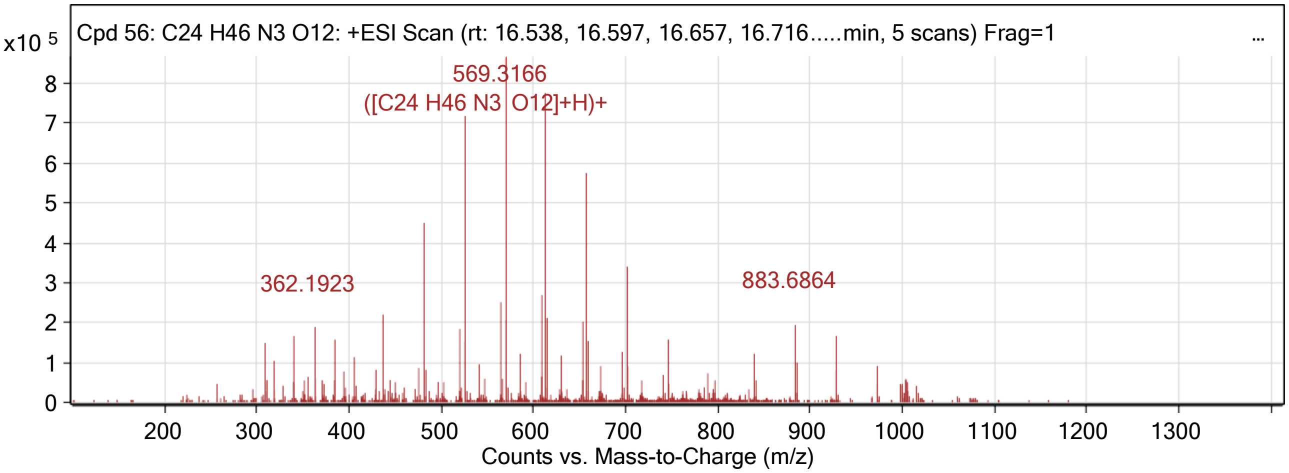

MS Zoomed Spectrum

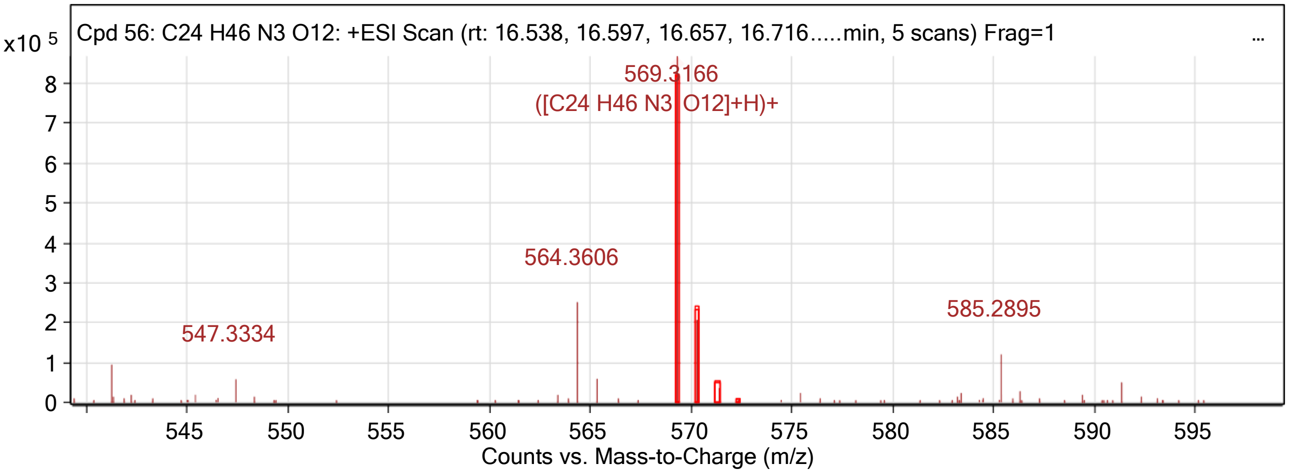

MS Spectrum Peak List

m/z	Calc *m/z*	Diff(ppm)	z	Abund	Formula	Ion
481.2633			1	446834.34		
525.2904			1	715784.5		
569.3166	569.3154	-2.1	1	867385.69	C24 H46 N3 O12	(M+H)+
570.3193	570.3186	-1.15	1	207230.66	C24 H46 N3 O12	(M+H)+
571.3212	571.3209	-0.53	1	34627.9	C24 H46 N3 O12	(M+H)+
572.3239	572.3235	-0.72	1	4742.23	C24 H46 N3 O12	(M+H)+
608.387			1	268516.19		
613.343			1	772425.63		
657.369			1	571057.38		
701.3951			1	340615.28		

MSMS Spectrum

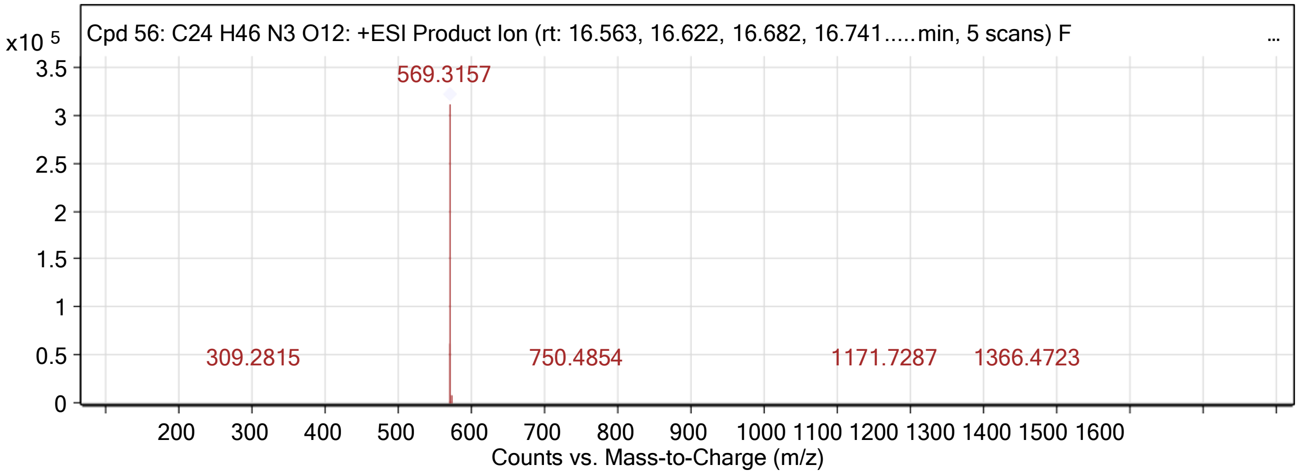

MS/MS Spectrum Peak List

m/z	*Calc m/z*	Diff(ppm)	z	Abund
117.0908	117.091	1.79		19.67
133.0866	133.0859	-5.18		43.77
175.1316	175.1315	-0.55		21.81
177.1115	177.1121	3.77		24.92
307.1738	307.1738	-0.18		16.73
309.2815	309.2788	-8.75		43.76
311.2912	311.2931	6.17		23.91
407.2553	407.2514	-9.66		21.2
440.2926	440.2966	9.27		17.08
569.3157	569.3154	-0.53	1	311193.66

Compound Label	*m/z*	RT	Algorithm	Mass
Cpd 57: C28 H52 O14	613.3431	16.876	Auto MS/MS	612.3356

Compound Chromatograms

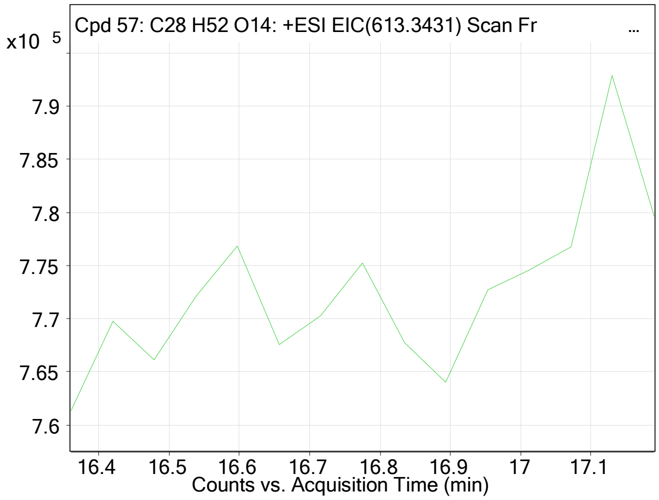

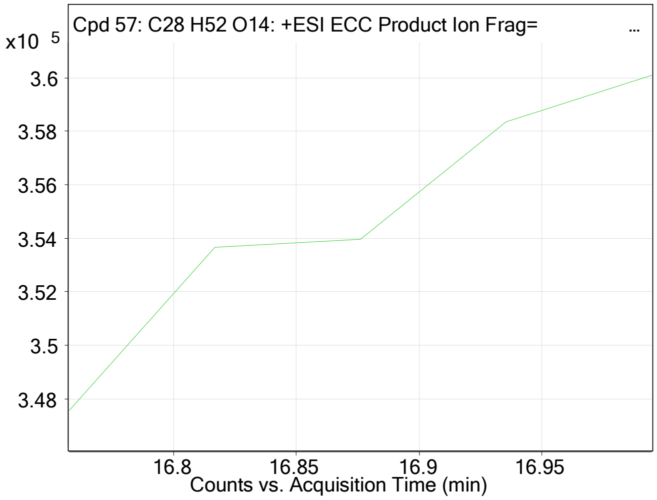

MS Spectrum

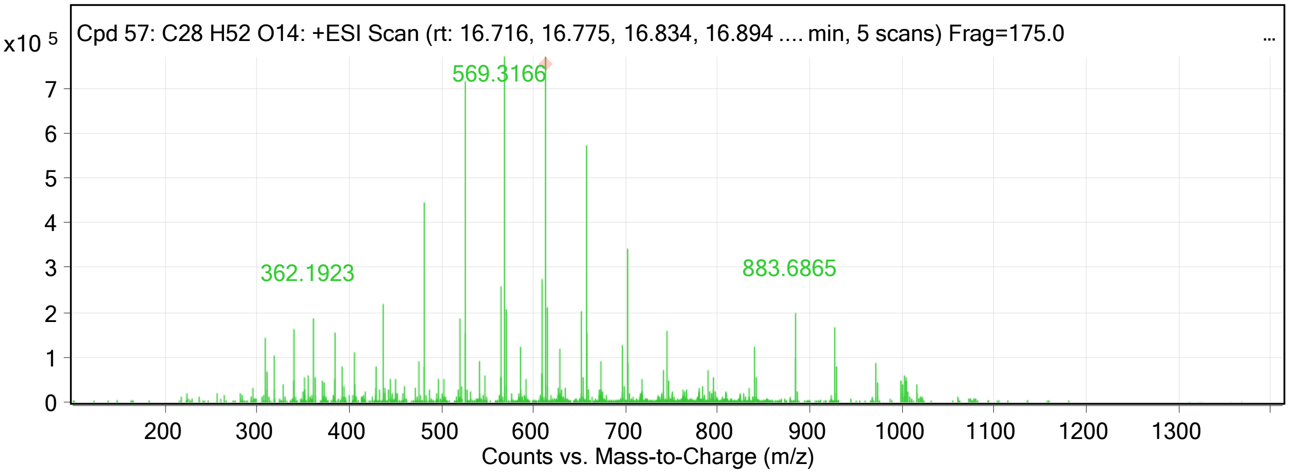

MS Zoomed Spectrum

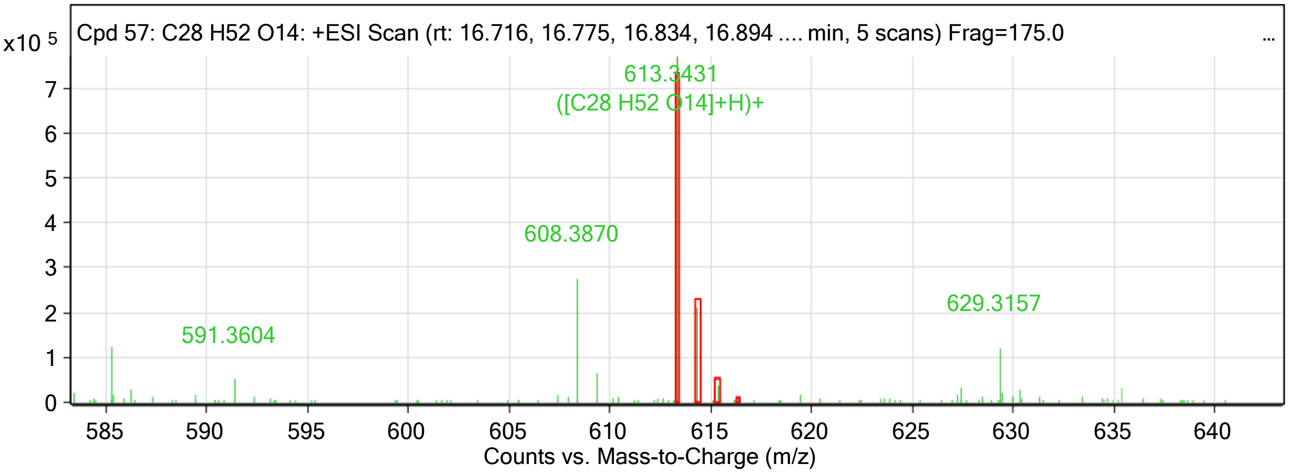

MS Spectrum Peak List

m/z	Calc *m/z*	Diff(ppm)	z	Abund	Formula	Ion
481.2634			1	445857.59		
525.2905			1	712815.94		
569.3166			1	871255.69		
608.387			1	275386.69		
613.3431	613.343	-0.2	1	769988.44	C28 H52 O14	(M+H)+
614.3456	614.3464	1.24	1	209893.92	C28 H52 O14	(M+H)+
615.3477	615.3489	1.83	1	37577.42	C28 H52 O14	(M+H)+
616.3573	616.3515	-9.4	1	5363.75	C28 H52 O14	(M+H)+
657.3691			1	571644.88		
701.3952			1	340682.06		

MSMS Spectrum

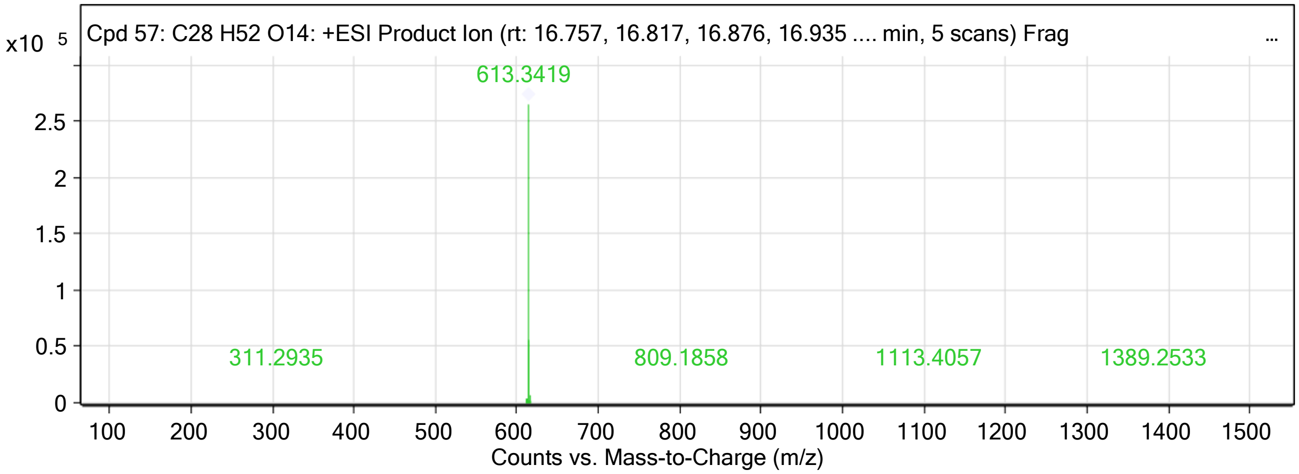

MS/MS Spectrum Peak List

m/z	Calc *m/z*	Diff(ppm)	z	Abund
133.0856	133.0859	2.06		63.59
309.2779	309.2788	2.99		69.53
311.2935	311.2945	3.14	1	97.24
612.3879			2	2679.64
612.638				3727.46
612.8889			2	2685.04
613.1409			1	1585.42
613.3419	613.343	1.83	1	265174.97
614.3446			1	56028.04
615.3469			1	6935.85

Compound Label	*m/z*	RT	Algorithm	Mass
Cpd 58: C24 H46 N3 O12	569.3165	16.978	Auto MS/MS	568.3091

Compound Chromatograms

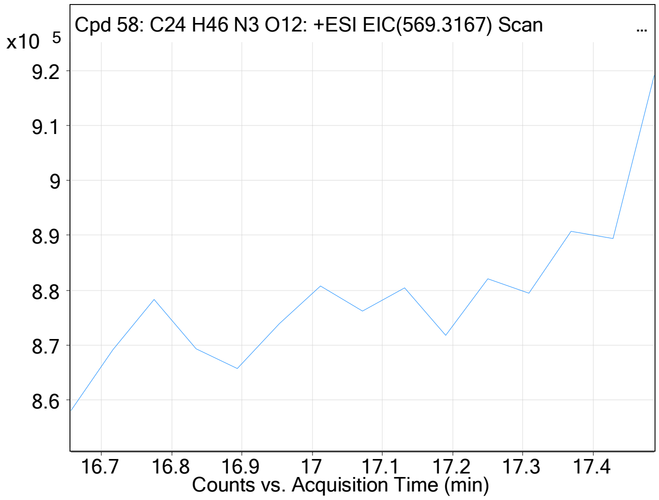

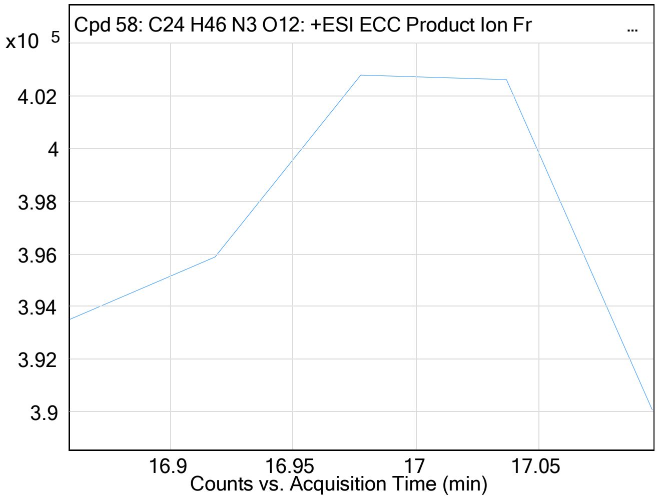

MS Spectrum

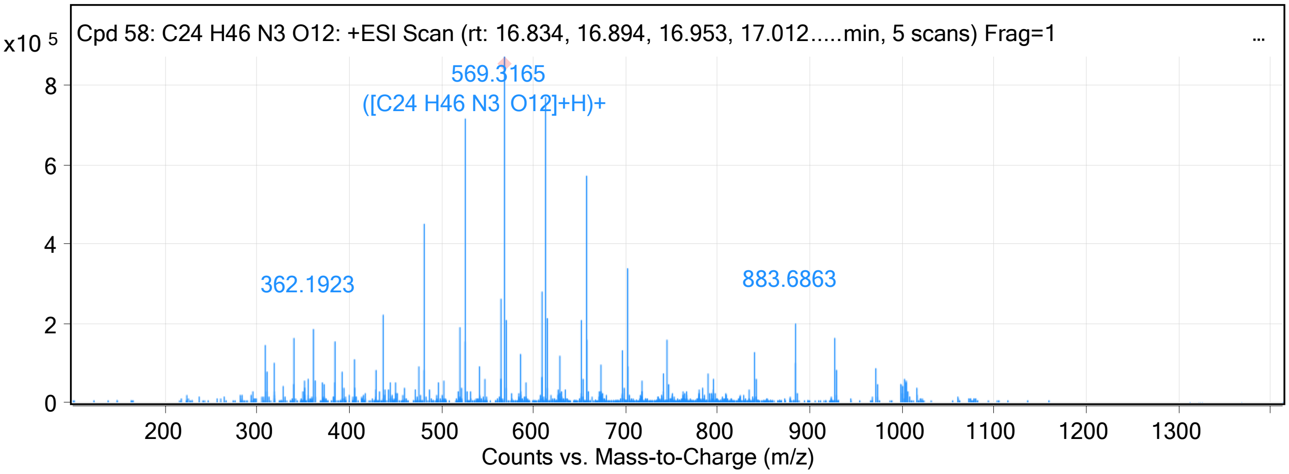

MS Zoomed Spectrum

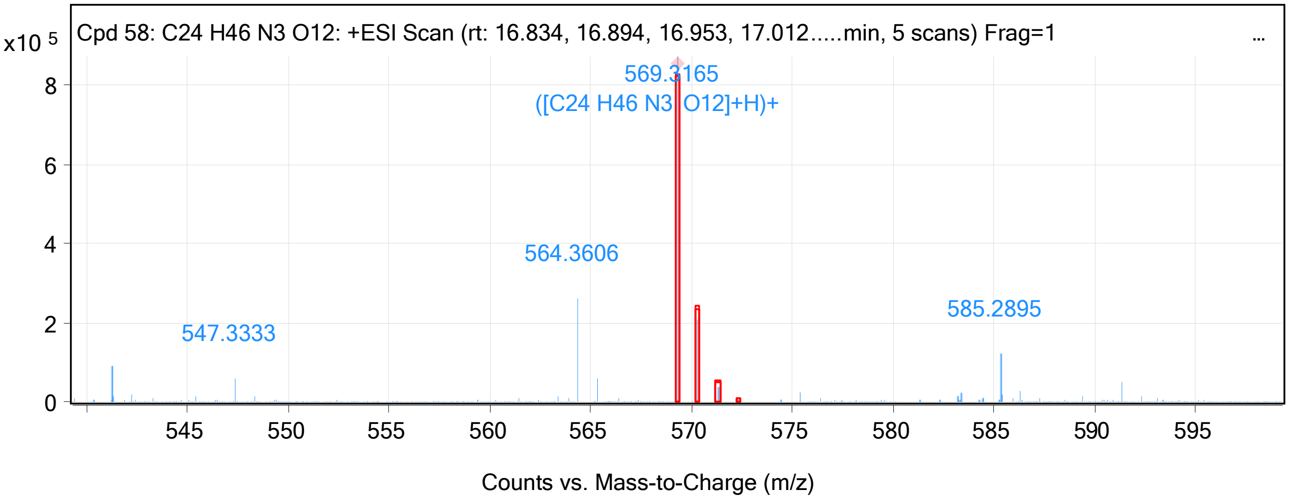

MS Spectrum Peak List

m/z	Calc *m/z*	Diff(ppm)	z	Abund	Formula	Ion
481.2633			1	450555.19		
525.2904			1	714818.63		
569.3165	569.3154	-1.83	1	873159.38	C24 H46 N3 O12	(M+H)+
570.3191	570.3186	-0.95	1	206779.22	C24 H46 N3 O12	(M+H)+
571.3213	571.3209	-0.54	1	34989.93	C24 H46 N3 O12	(M+H)+
572.3241	572.3235	-1.03	1	4937.85	C24 H46 N3 O12	(M+H)+
608.3869			1	277970.06		
613.343			1	771148.75		

MSMS Spectrum

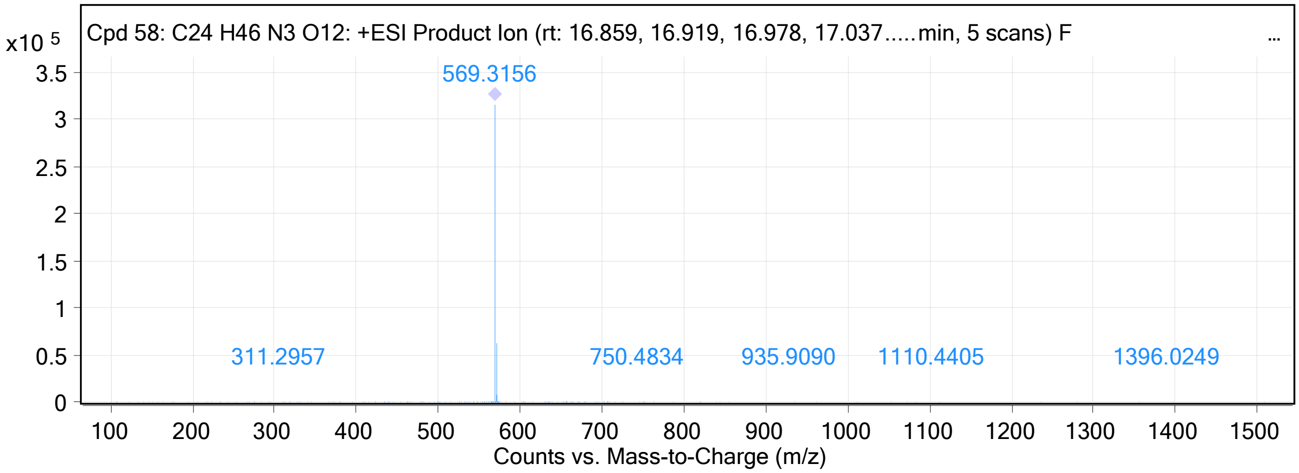

MS/MS Spectrum Peak List

m/z	*Calc m/z*	Diff(ppm)	z	Abund
133.0855	133.0859	2.83		38.32
195.1217	195.1214	-1.68		32.5
200.0609	200.0639	14.97		12.66
233.1758	233.1747	-4.52		13.3
309.2774	309.2775	0.28		50.15
311.2957	311.2945	-3.98		58.15
371.2279	371.2262	-4.55		20.08
372.2315	372.234	6.92		17.32
538.2578	538.2607	5.32		15.68
569.3156	569.3154	-0.34	1	316164

Compound Label	*m/z*	RT	Algorithm	Mass
Cpd 59: C28 H52 O14	613.3429	17.173	Auto MS/MS	612.3354

Compound Chromatograms

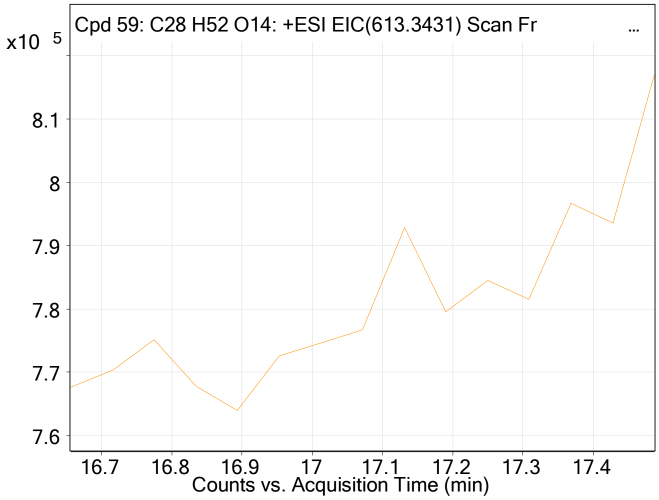

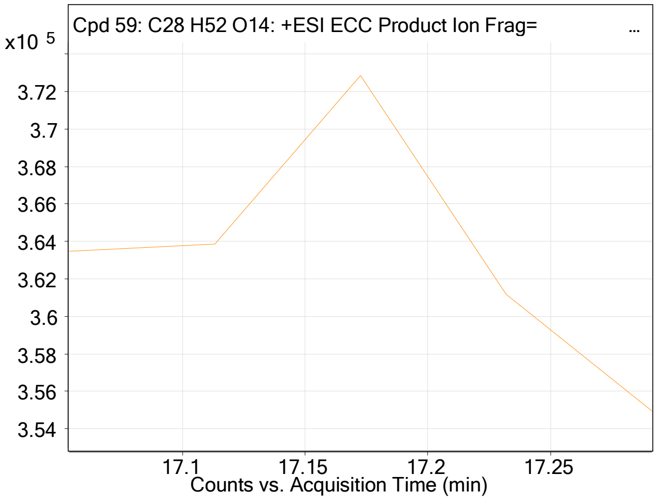

MS Spectrum

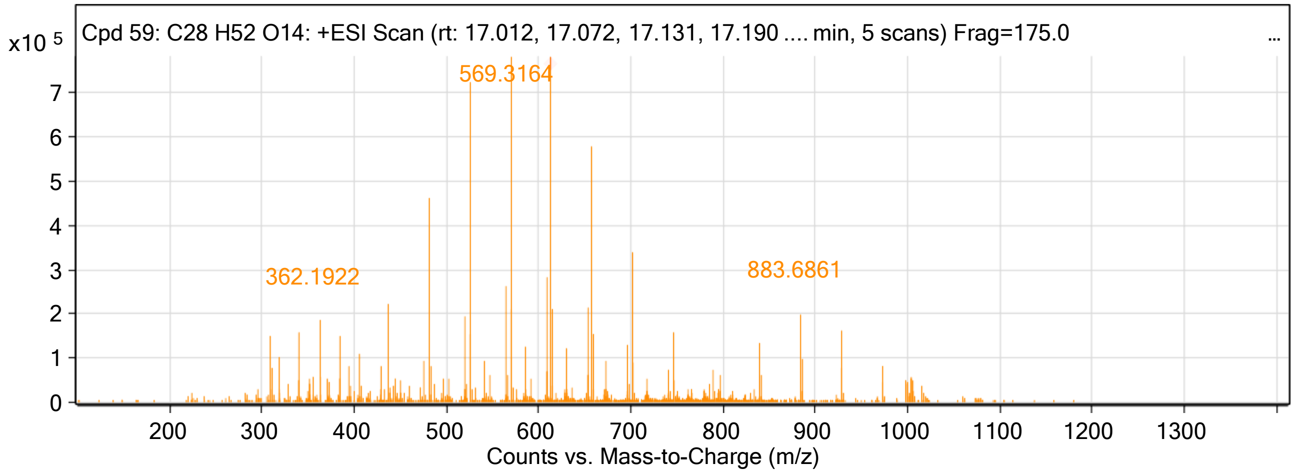

MS Zoomed Spectrum

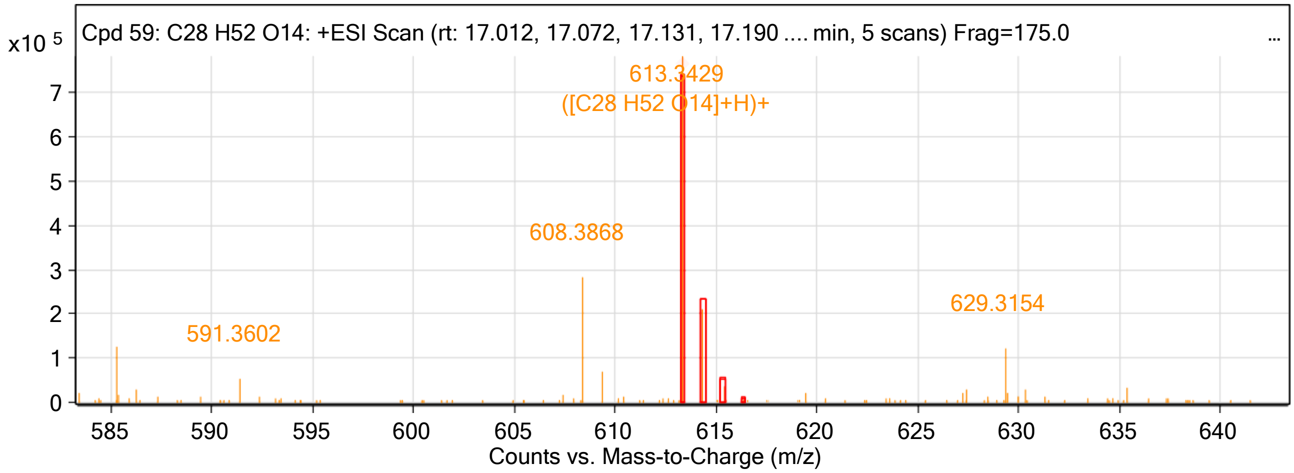

MS Spectrum Peak List

m/z	Calc *m/z*	Diff(ppm)	z	Abund	Formula	Ion
481.2632			1	459397.59		
525.2902			1	723400.19		
569.3164			1	878250.63		
608.3868			1	283154.03		
613.3429	613.343	0.18	1	781659.06	C28 H52 O14	(M+H)+
614.3454	614.3464	1.62	1	211229.8	C28 H52 O14	(M+H)+
615.3475	615.3489	2.28	1	37878.04	C28 H52 O14	(M+H)+
616.3564	616.3515	-7.84	1	5548.65	C28 H52 O14	(M+H)+
657.3688			1	575827.56		
701.3949			1	339910.03		

MSMS Spectrum

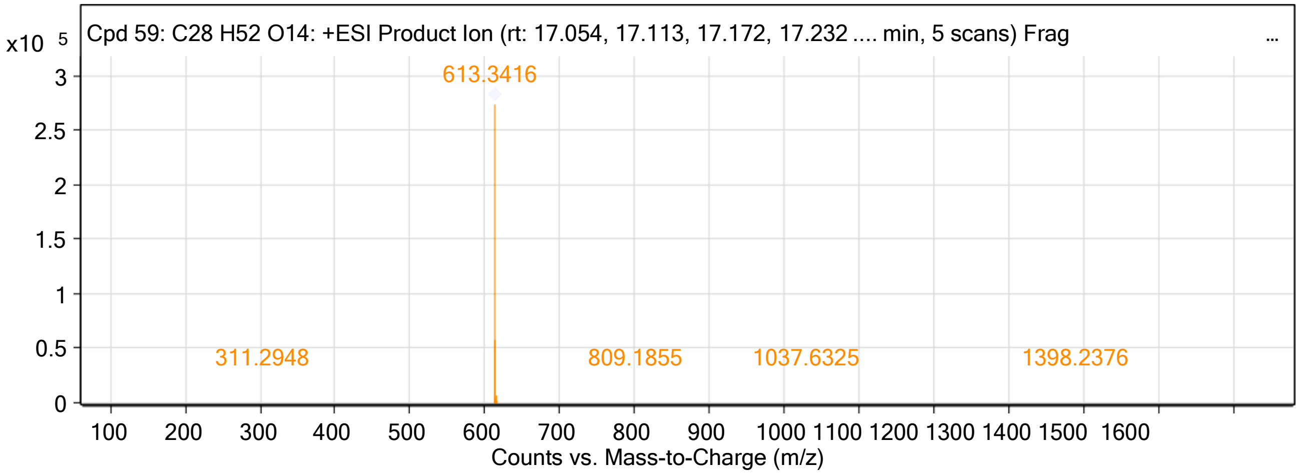

MS/MS Spectrum Peak List

m/z	*Calc m/z*	Diff(ppm)	z	Abund
133.0865	133.0859	-4.71		37.53
309.2781	309.2788	2.23		60.35
311.2948	311.2945	-1.2		101.73
357.0641	357.0664	6.4	1	44.22
612.3876			2	2824.14
612.6379				3534.99
612.8883			2	2744.48
613.3416	613.343	2.19	1	273502.72
614.3443			1	56977.68
615.3468			1	6892.29

Compound Label	*m/z*	RT	Algorithm	Mass
Cpd 60: C24 H46 N3 O12	569.3164	17.274	Auto MS/MS	568.3089

Compound Chromatograms

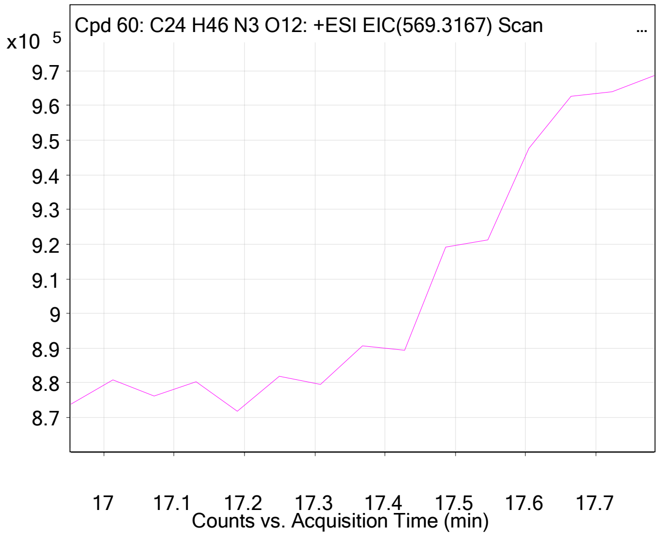

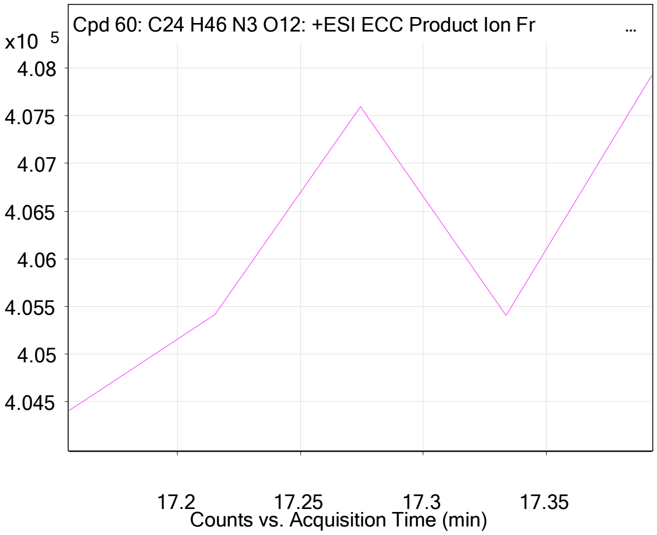

MS Spectrum

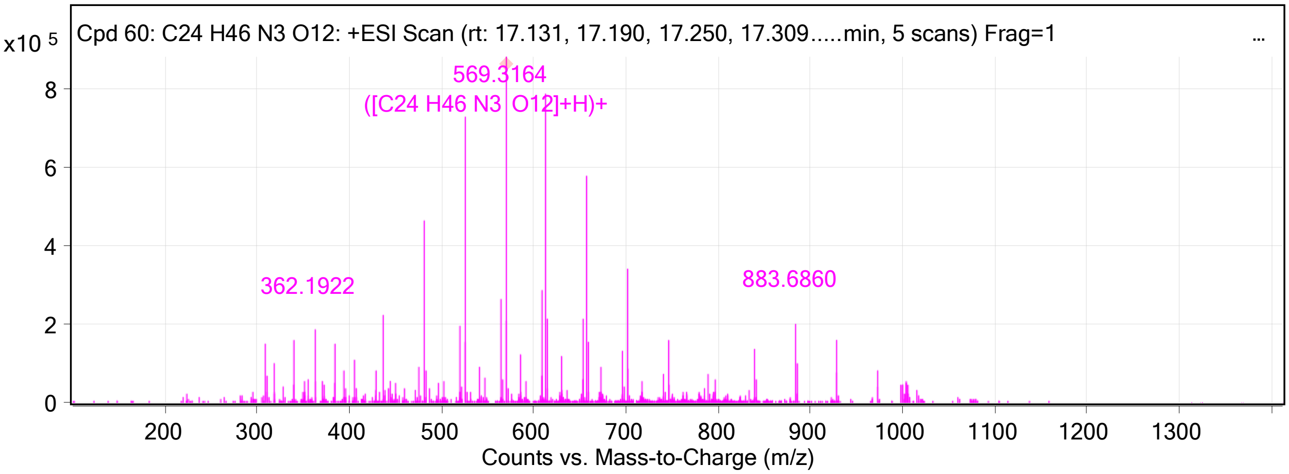

MS Zoomed Spectrum

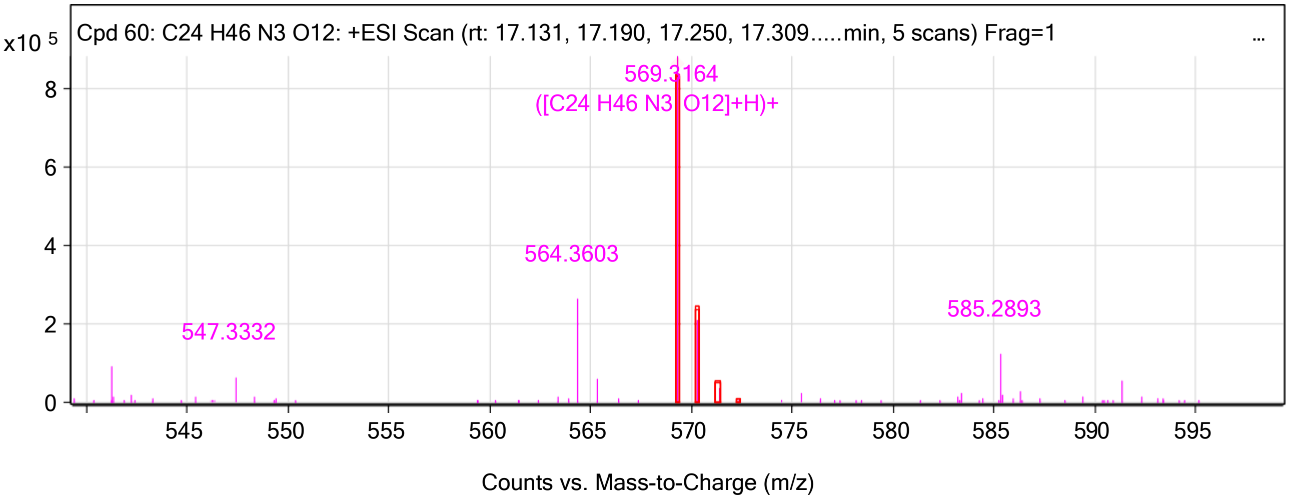

MS Spectrum Peak List

m/z	Calc *m/z*	Diff(ppm)	z	Abund	Formula	Ion
481.2631			1	462033.19		
525.2902			1	727032.81		
569.3164	569.3154	-1.64	1	880898.31	C24 H46 N3 O12	(M+H)+
570.319	570.3186	-0.63	1	210176.91	C24 H46 N3 O12	(M+H)+
571.321	571.3209	-0.09	1	34799.26	C24 H46 N3 O12	(M+H)+
572.324	572.3235	-0.85	1	5138.92	C24 H46 N3 O12	(M+H)+
608.3867			1	284964.25		
613.3428			1	787050.19		
657.3688			1	575656.5		
701.3948			1	341920.94		

MSMS Spectrum

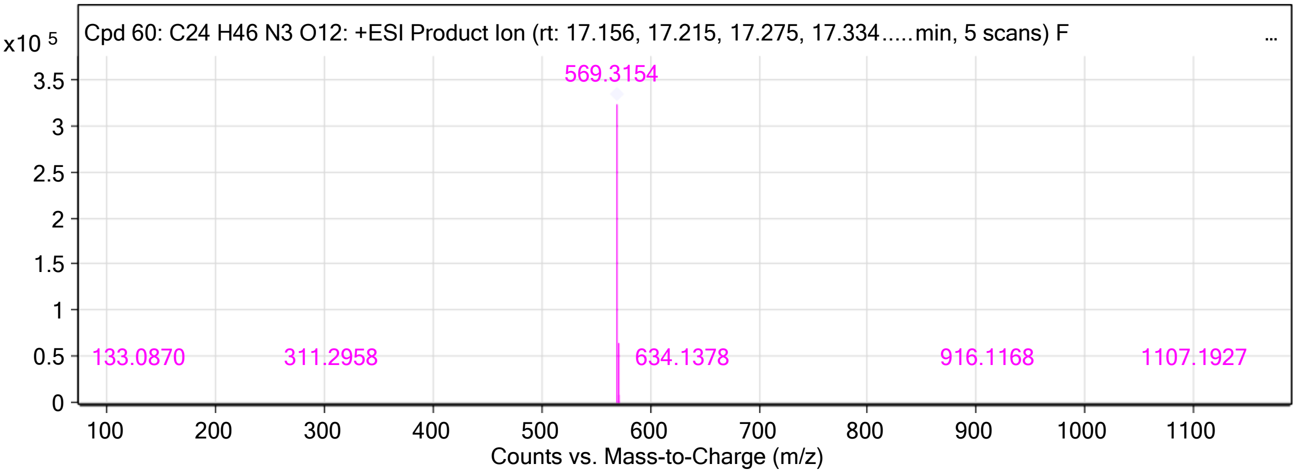

MS/MS Spectrum Peak List

m/z	Calc *m/z*	Diff(ppm)	z	Abund
117.091	117.091	0.43		14.93
133.087	133.0859	-7.91		60.09
177.1117	177.1121	2.58		11.83
208.1307	208.1305	-0.7		13.46
309.2795	309.2788	-2.12		18.04
311.2958	311.2945	-4.2	1	43.91
351.2847	351.2853	1.84		11.83
504.296	504.2916	-8.73		18
538.3019	538.297	-8.94	1	19.76
569.3154	569.3154	0.07	1	323318.41

Compound Label	*m/z*	RT	Algorithm	Mass
Cpd 61: C28 H52 O14	613.343	17.469	Auto MS/MS	612.3355

Compound Chromatograms

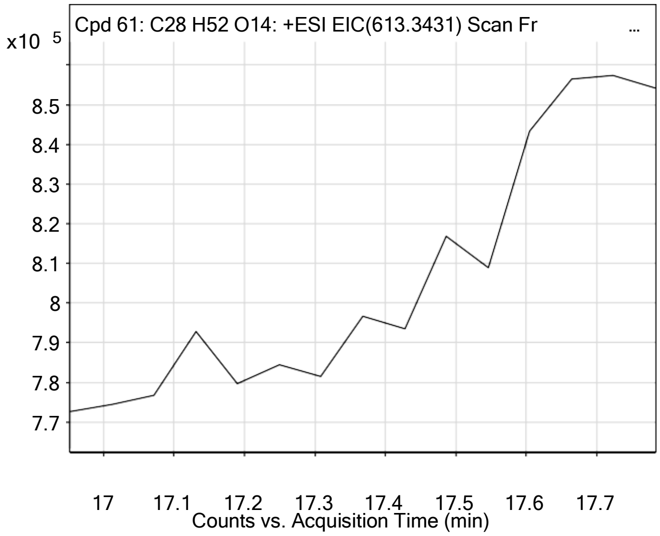

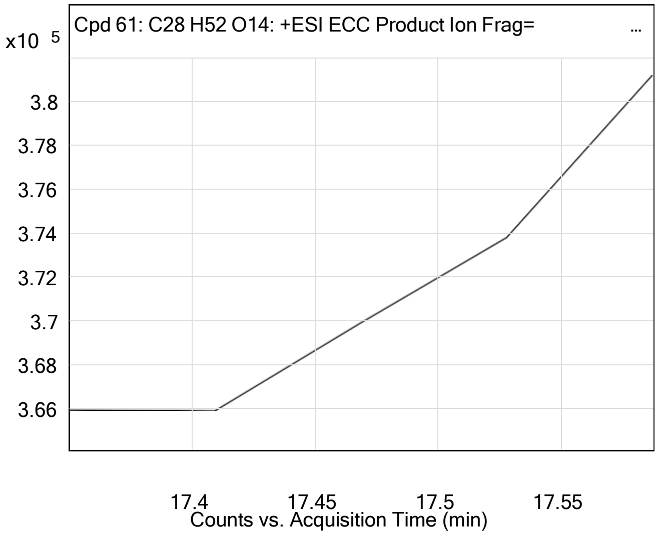

MS Spectrum

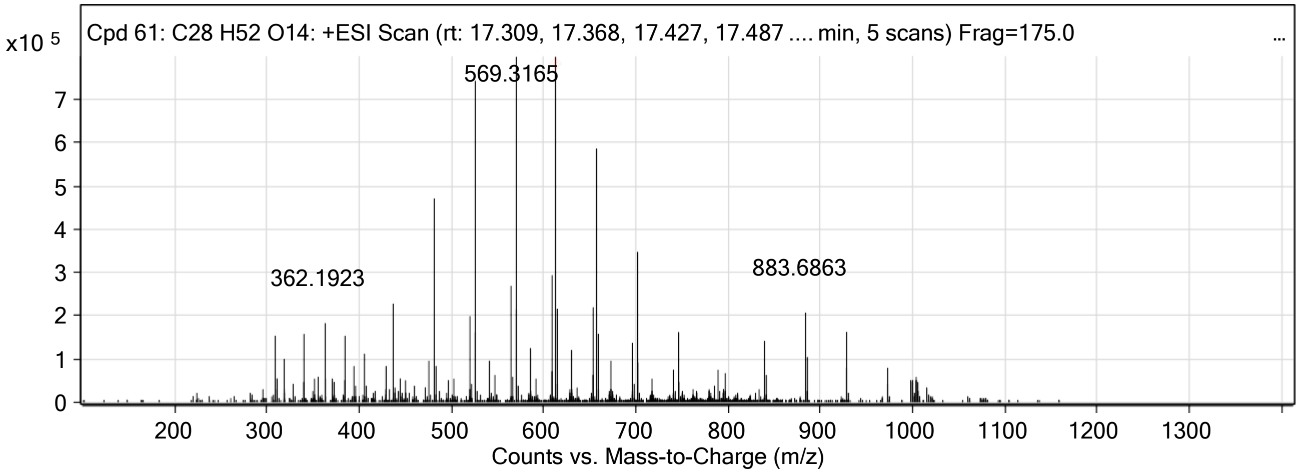

MS Zoomed Spectrum

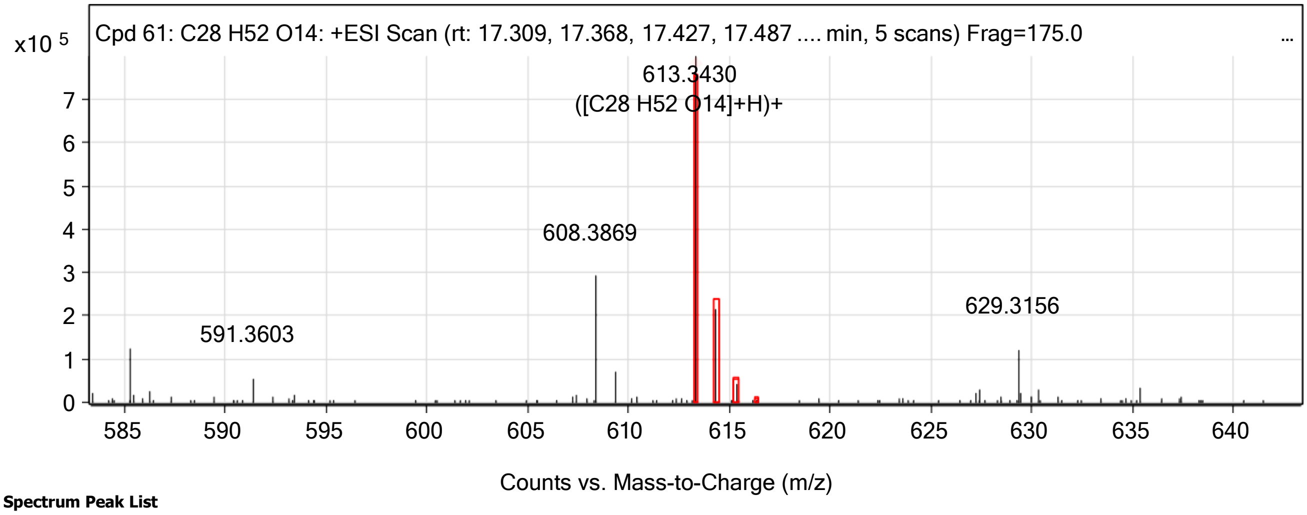

MS Spectrum Peak List

m/z	Calc *m/z*	Diff(ppm)	z	Abund	Formula	Ion
481.2633			1	469381.69		
525.2903			1	741508.13		
569.3165			1	899988.69		
608.3869			1	291407.44		
613.343	613.343	0.04	1	799522.88	C28 H52 O14	(M+H)+
614.3456	614.3464	1.37	1	216132.09	C28 H52 O14	(M+H)+
615.3476	615.3489	2.06	1	39409.12	C28 H52 O14	(M+H)+

MSMS Spectrum

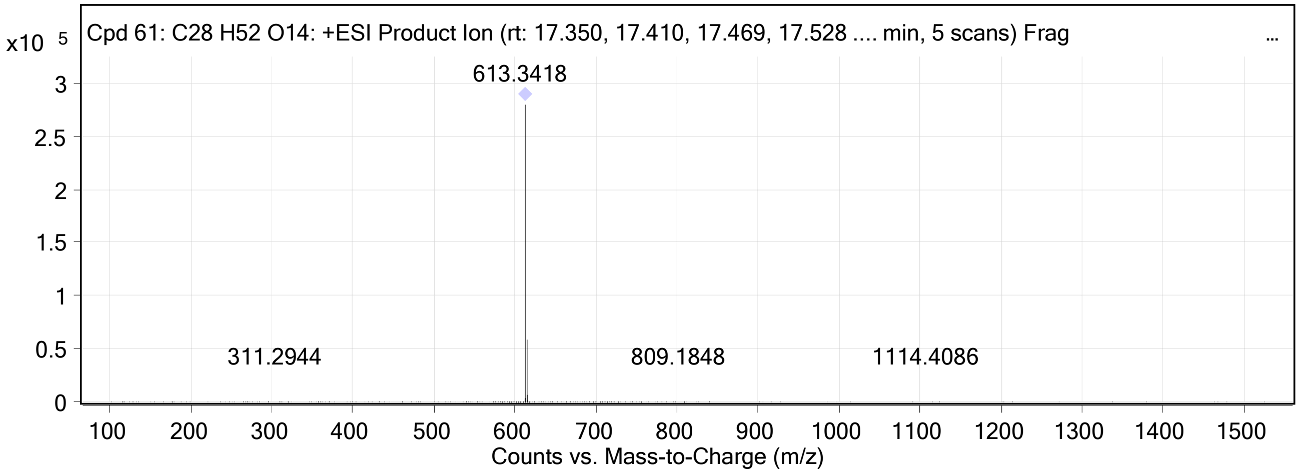

MS/MS Spectrum Peak List

m/z	*Calc m/z*	Diff(ppm)	z	Abund
309.2769	309.2788	6.2	1	89.42
357.0632	357.0664	8.9	1	42.52
612.3879			2	2889.56
612.6381			2	3354.27
612.8897			2	2820.5
613.1398			2	1373.2
613.3418	613.343	1.9	1	279875.97
614.3446			1	58874.28
615.1412			1	1175.73
615.3467			1	7152.39

Compound Label	*m/z*	RT	Algorithm	Mass
Cpd 62: C24 H46 N3 O12	569.3163	17.571	Auto MS/MS	568.309

Compound Chromatograms

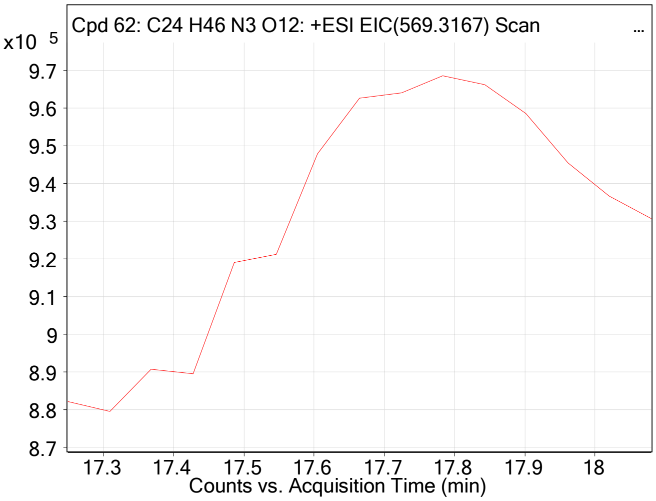

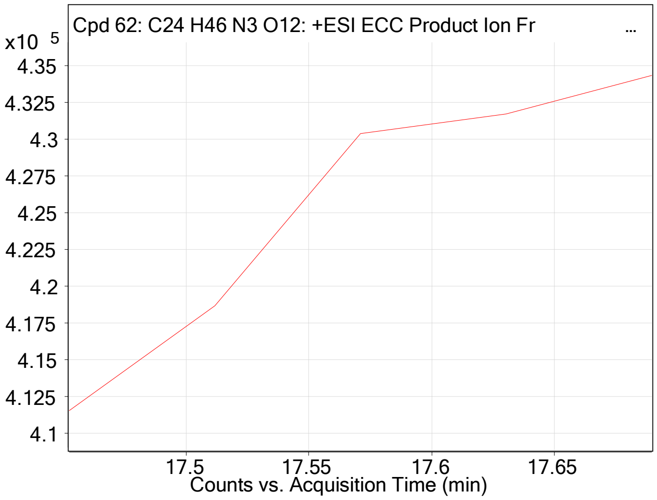

MS Spectrum

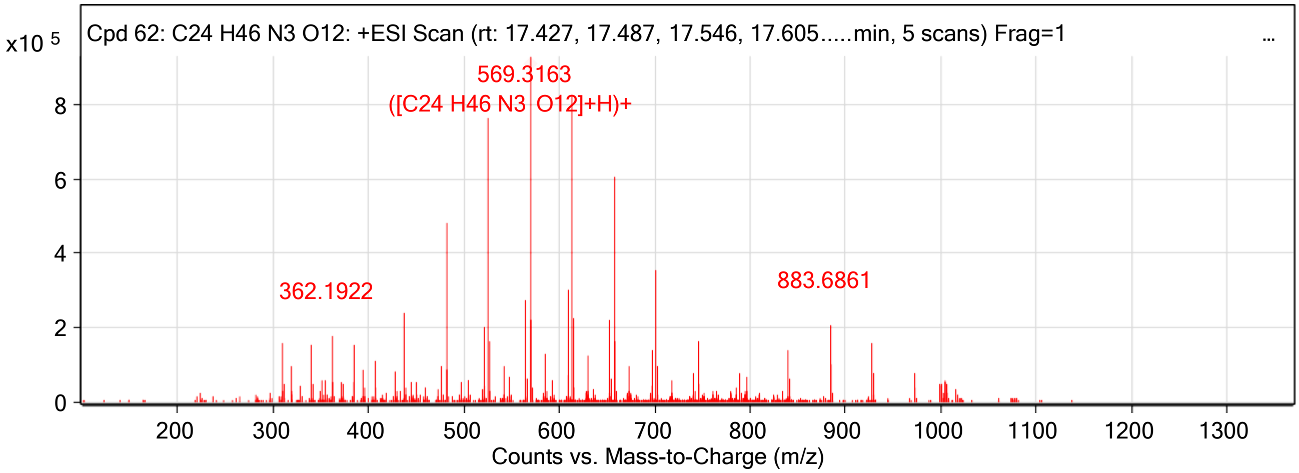

MS Zoomed Spectrum

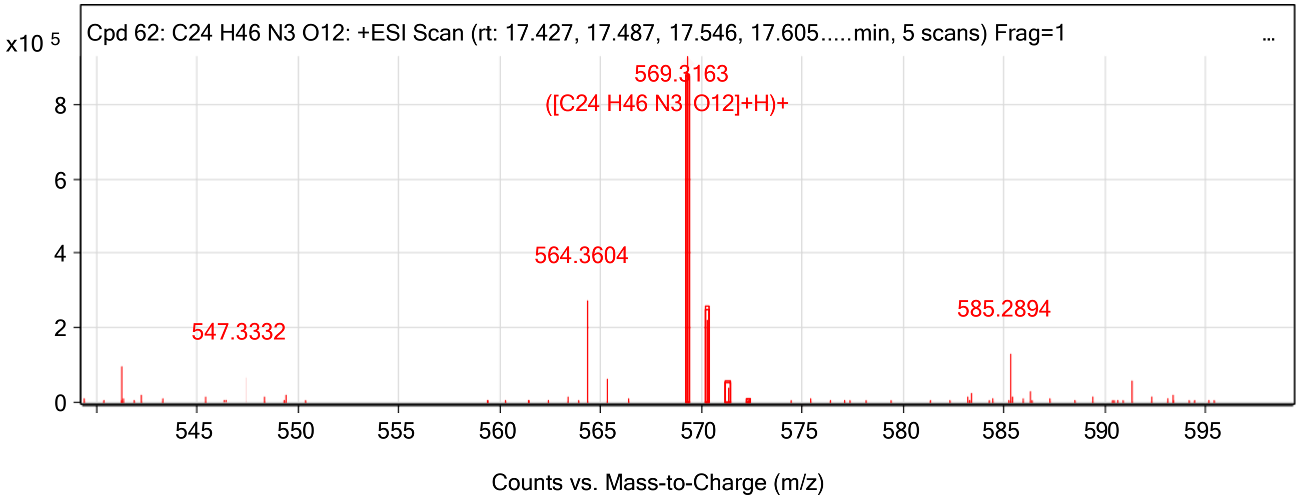

MS Spectrum Peak List

m/z	Calc *m/z*	Diff(ppm)	z	Abund	Formula	Ion
481.2632			1	480305.66		
525.2902			1	762819.88		
569.3163	569.3154	-1.6	1	928038.69	C24 H46 N3 O12	(M+H)+
570.319	570.3186	-0.78	1	220927.58	C24 H46 N3 O12	(M+H)+
571.3212	571.3209	-0.43	1	36533.21	C24 H46 N3 O12	(M+H)+
572.3239	572.3235	-0.64	1	5256.66	C24 H46 N3 O12	(M+H)+
608.3868			1	300599.34		
613.3429			1	823814.13		
657.3688			1	601934.13		
701.3948			1	355407.44		

MSMS Spectrum

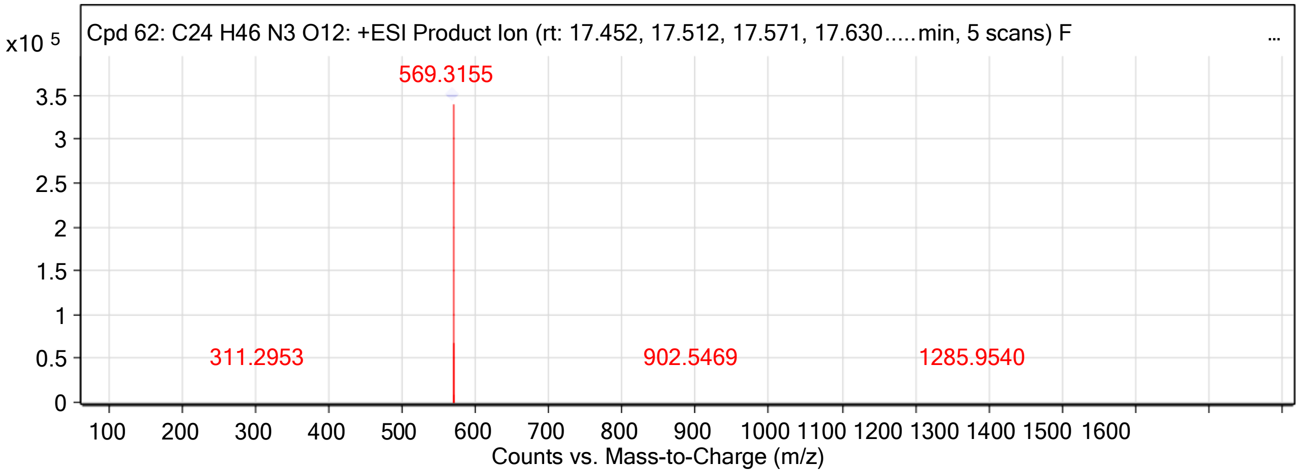

MS/MS Spectrum Peak List

m/z	*Calc m/z*	Diff(ppm)	z	Abund
124.0802	124.0757	-36.52		15.25
133.0854	133.0859	4.1		38.19
175.134	175.1329	-6.42		15.33
283.1765	283.1778	4.7		27.57
309.2764	309.2775	3.45		18.01
311.2953	311.2945	-2.84	1	37.92
371.2266	371.2262	-0.97		28.29
449.28	449.2745	-12.15		16.77
460.2609	460.2627	3.88		15.3
569.3155	569.3154	-0.07	1	339869.97

Compound Label	*m/z*	RT	Algorithm	Mass
Cpd 63: C28 H52 O14	613.3431	17.766	Auto MS/MS	612.3356

Compound Chromatograms

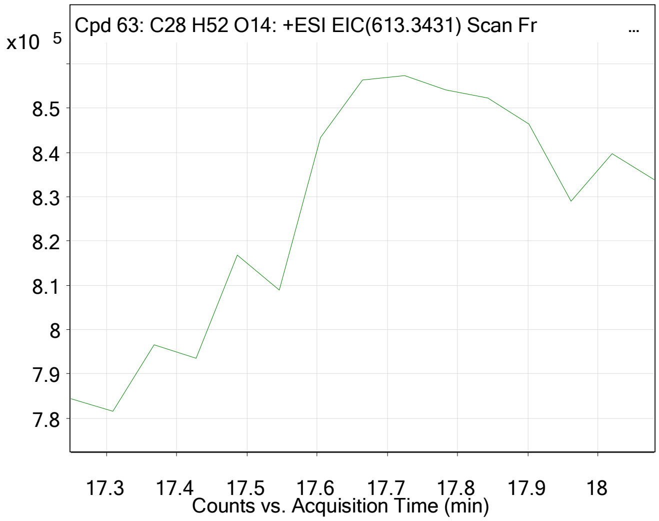

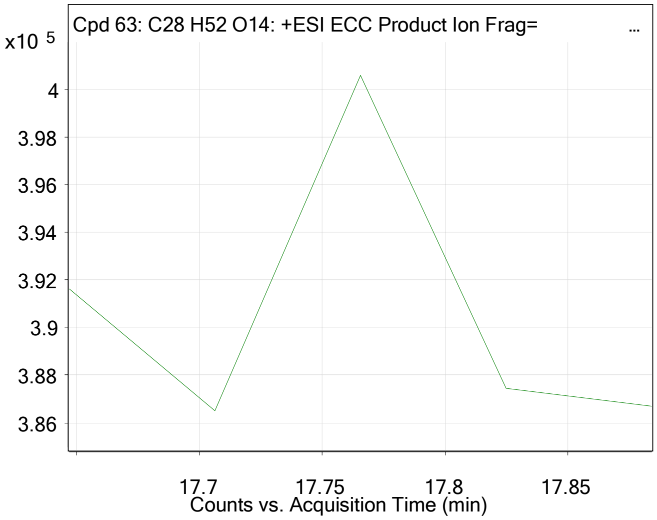

MS Spectrum

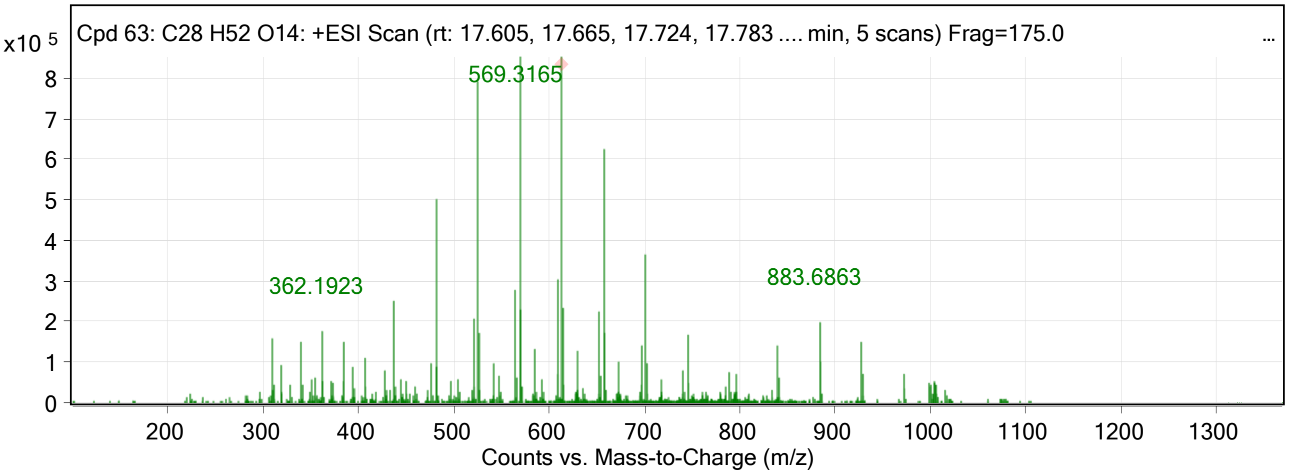

MS Zoomed Spectrum

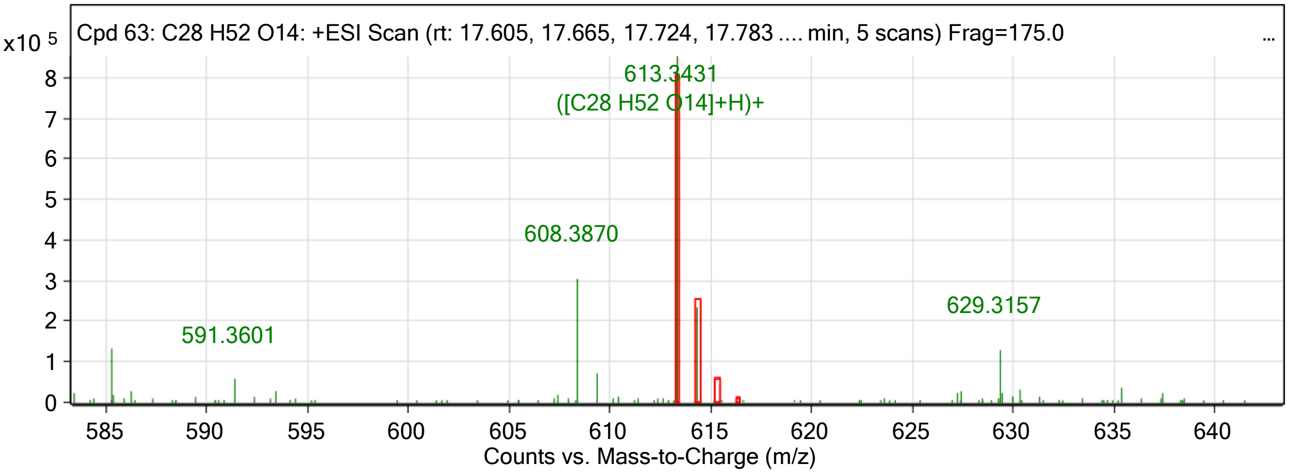

MS Spectrum Peak List

m/z	Calc *m/z*	Diff(ppm)	z	Abund	Formula	Ion
481.2633			1	499613.94		
525.2903			1	791541.38		
569.3165			1	961874.81		
608.387			1	303752.69		
613.3431	613.343	-0.13	1	852726.19	C28 H52 O14	(M+H)+
614.3457	614.3464	1.23	1	231127.03	C28 H52 O14	(M+H)+
615.3475	615.3489	2.18	1	42106.76	C28 H52 O14	(M+H)+
616.3549	616.3515	-5.42	1	5801.87	C28 H52 O14	(M+H)+
657.369			1	625370.31		
701.3951			1	367256.47		

MSMS Spectrum

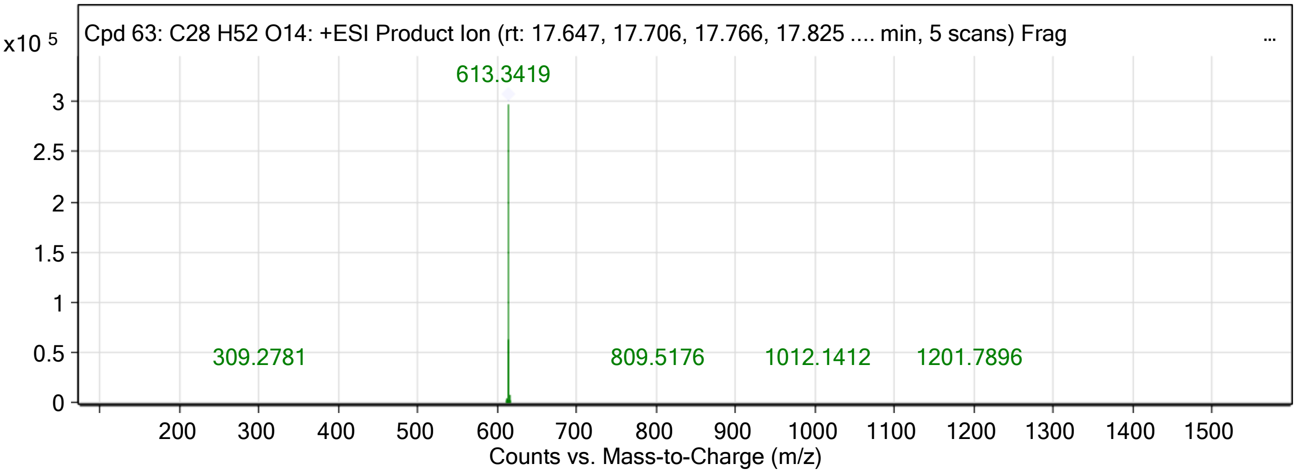

MS/MS Spectrum Peak List

m/z	*Calc m/z*	Diff(ppm)	z	Abund
133.0854	133.0859	3.87		53.02
177.1136	88.5558	-500005.63	2	39.25
309.2781	309.2788	2.41		89.59
311.2949	311.2945	-1.36	1	78.73
357.067	357.0664	-1.76		46.48
612.6385				3013.63
612.8889			2	2609.03
613.3419	613.343	1.83	1	297409.25
614.3446			1	61632.29
615.3467			1	7343.65

Compound Label	*m/z*	RT	Algorithm	Mass
Cpd 64: C26 H48 O13	569.3167	17.868	Auto MS/MS	568.3093

Compound Chromatograms

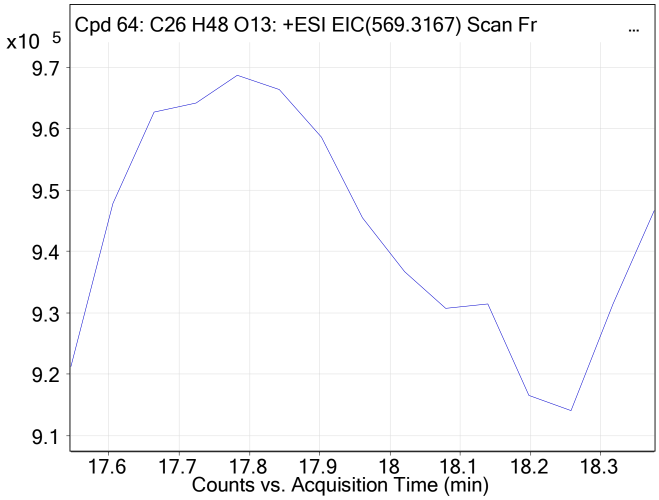

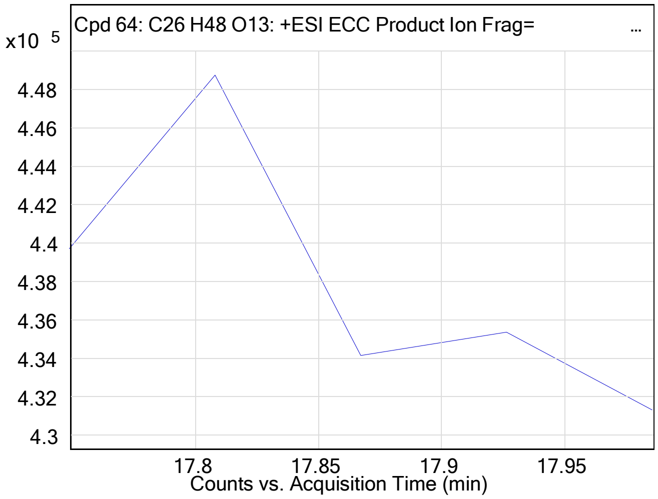

MS Spectrum

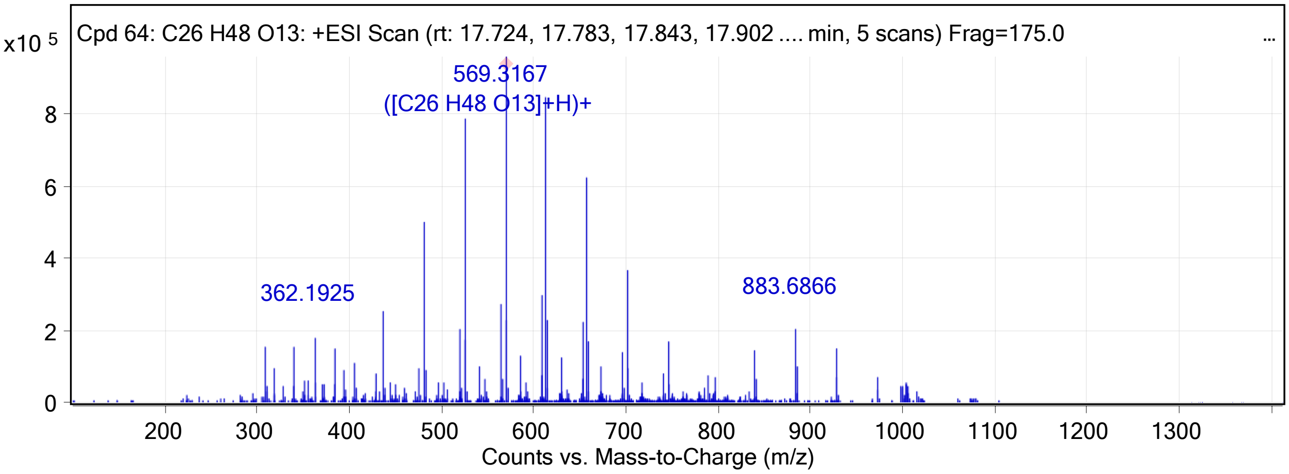

MS Zoomed Spectrum

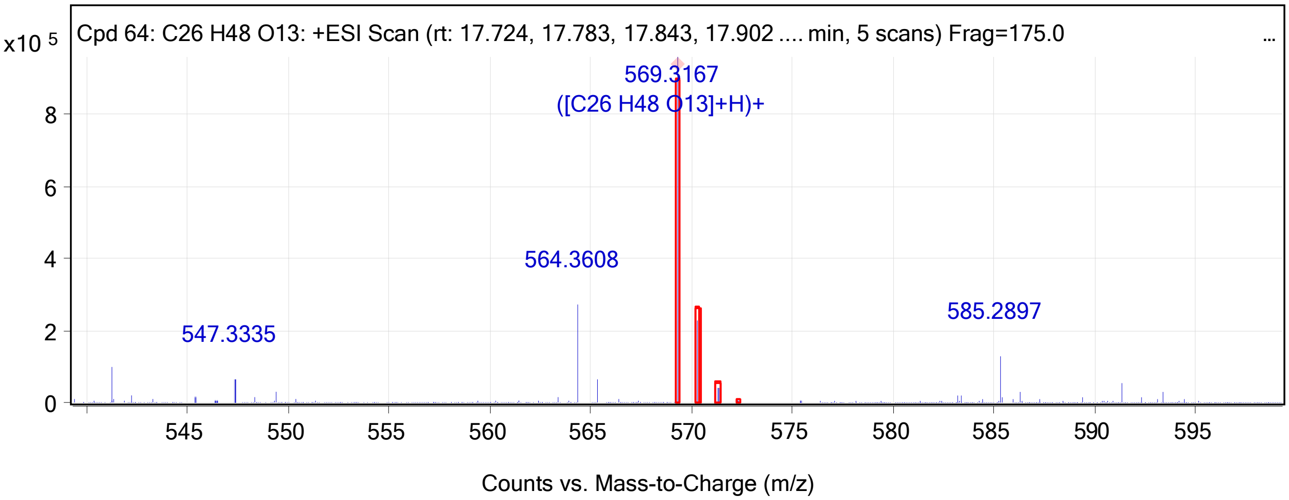

MS Spectrum Peak List

m/z	Calc *m/z*	Diff(ppm)	z	Abund	Formula	Ion
481.2635			1	498299		
525.2905			1	789769.63		
569.3167	569.3168	0.06	1	960597.19	C26 H48 O13	(M+H)+
570.3195	570.3202	1.27	1	226029.33	C26 H48 O13	(M+H)+
571.3215	571.3226	1.93	1	37727.27	C26 H48 O13	(M+H)+
572.3244	572.3253	1.57	1	5429.35	C26 H48 O13	(M+H)+
608.3872			1	297684.94		
613.3433			1	847880.63		

MSMS Spectrum

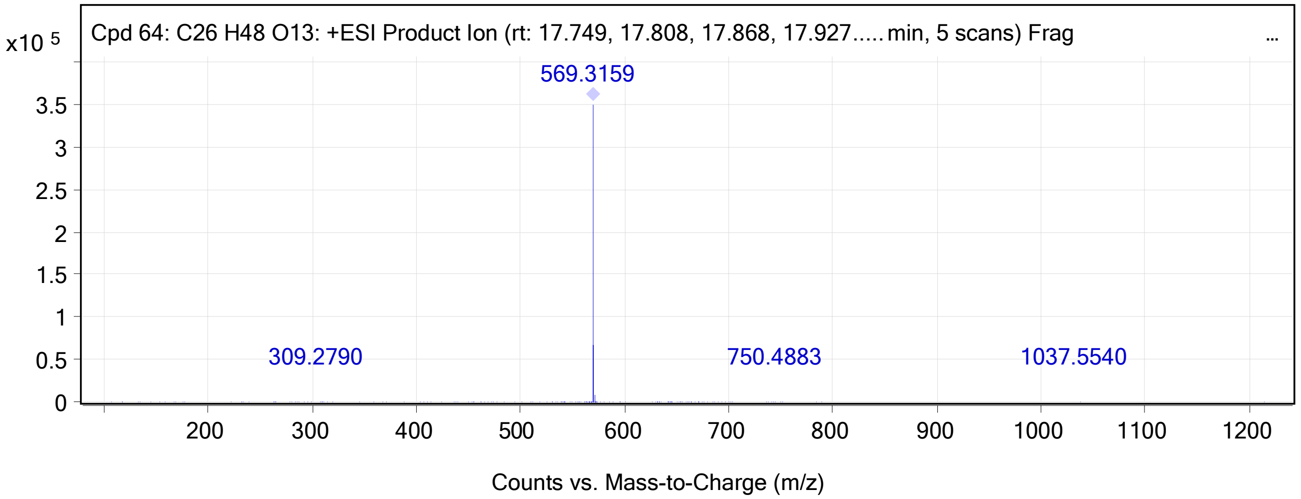

MS/MS Spectrum Peak List

m/z	*Calc m/z*	Diff(ppm)	z	Abund
117.0914	117.091	-3.2		51.96
133.0873	133.0859	-10.44		34.9
166.1296	166.1352	33.96		17.95
177.1114	177.1121	4		26.68
309.279	309.2788	-0.71		45.36
311.2966	311.2945	-6.97		43.92
454.2735	454.2772	8.16		16.58
538.2931	538.2984	9.86		19.18
566.3023	283.1464	-500008.47	2	23.46
569.3159	569.3168	1.55	1	350099.38

Compound Label	*m/z*	RT	Algorithm	Mass
Cpd 65: C28 H52 O14	613.3434	18.062	Auto MS/MS	612.3359

Compound Chromatograms

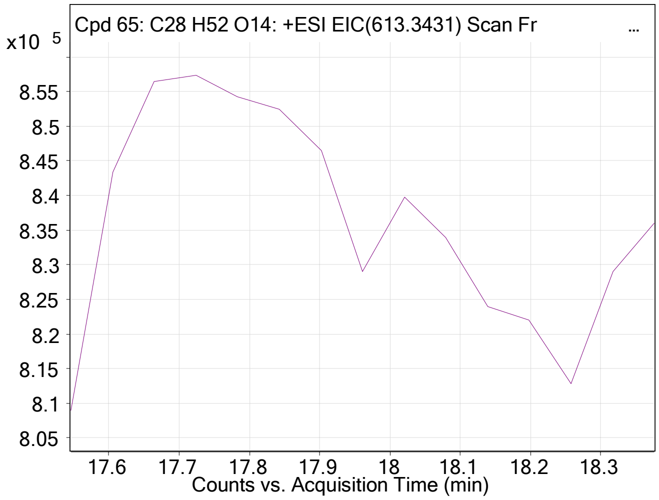

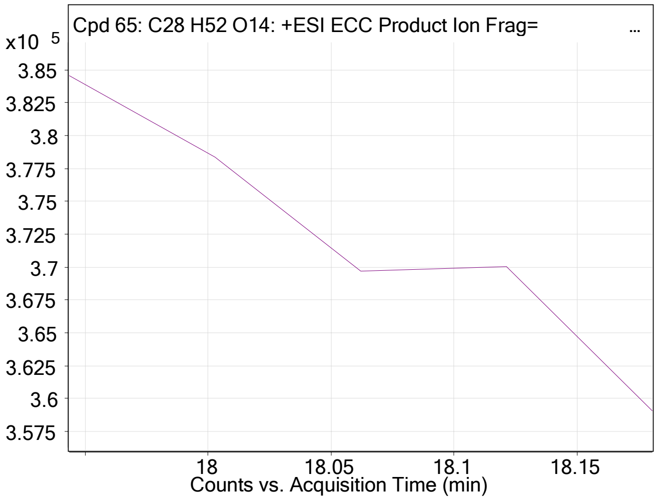

MS Spectrum

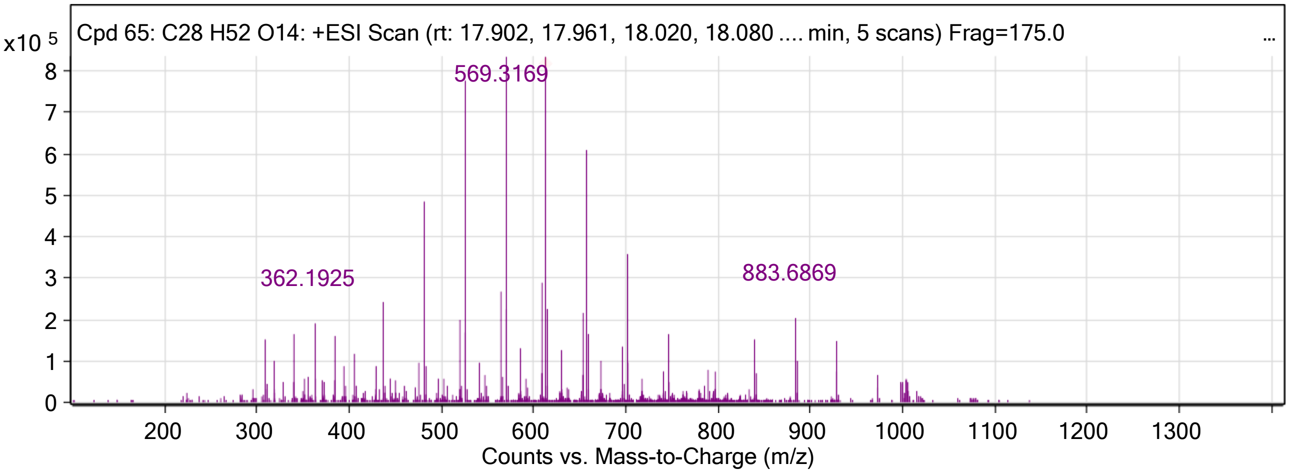

MS Zoomed Spectrum

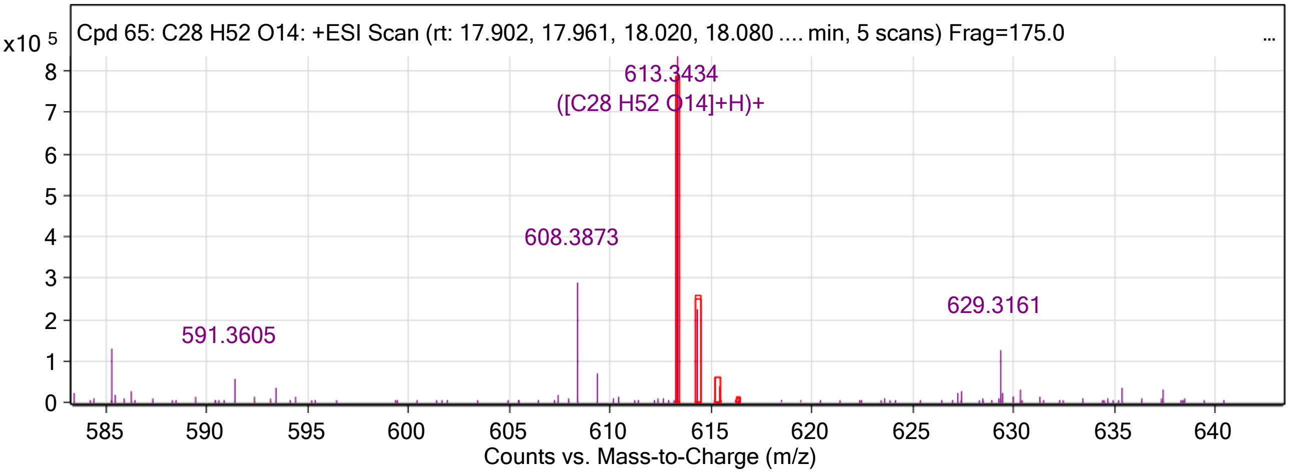

MS Spectrum Peak List

m/z	Calc *m/z*	Diff(ppm)	z	Abund	Formula	Ion
481.2636			1	482438.59		
525.2906			1	773262.13		
569.3169			1	940570.88		
608.3873			1	290243.44		
613.3434	613.343	-0.65	1	834629.63	C28 H52 O14	(M+H)+
614.346	614.3464	0.74	1	226020.25	C28 H52 O14	(M+H)+
615.3479	615.3489	1.59	1	40722.14	C28 H52 O14	(M+H)+
616.3555	616.3515	-6.46	1	5551.1	C28 H52 O14	(M+H)+
657.3694			1	608608.13		
701.3955			1	357756.56		

MSMS Spectrum

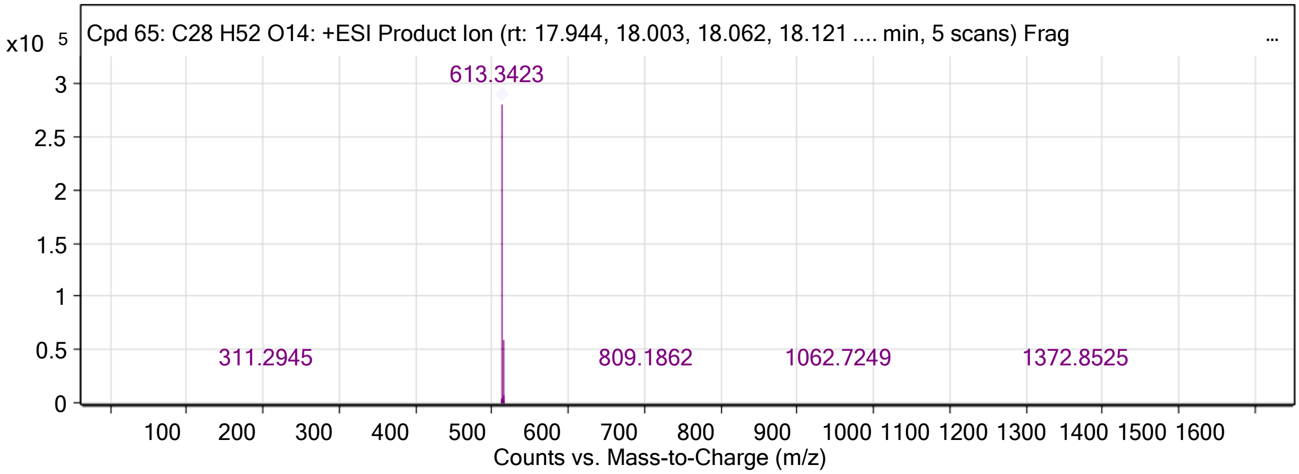

MS/MS Spectrum Peak List

m/z	*Calc m/z*	Diff(ppm)	z	Abund
133.0846	133.0859	10.17		56.85
309.2795	309.2788	-2.12		84.35
311.2945	311.2945	-0.28	1	143.46
357.0672	357.0664	-2.46	1	50.05
612.3884			2	2708.1
612.6385			2	3444.62
612.8893			2	2789.47
613.3423	613.343	1.19	1	280812.06
614.345			1	58680.13
615.347			1	7225.08

Compound Label	*m/z*	RT	Algorithm	Mass
Cpd 66: C26 H48 O13	569.3168	18.164	Auto MS/MS	568.3094

Compound Chromatograms

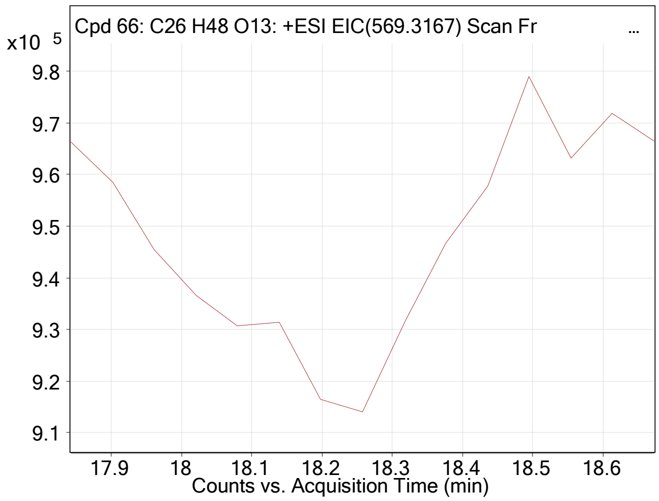

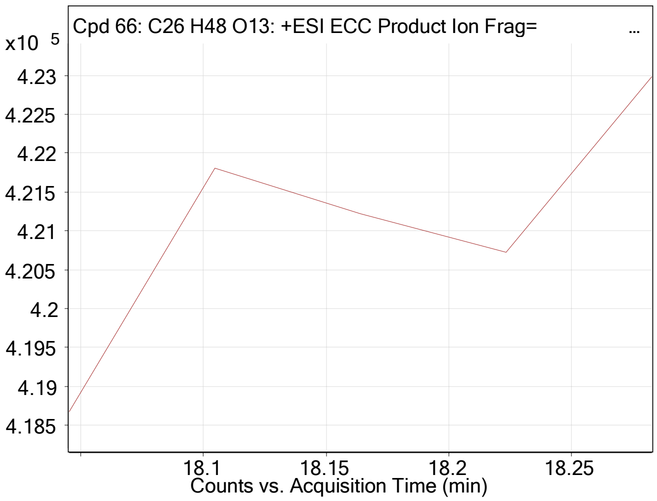

MS Spectrum

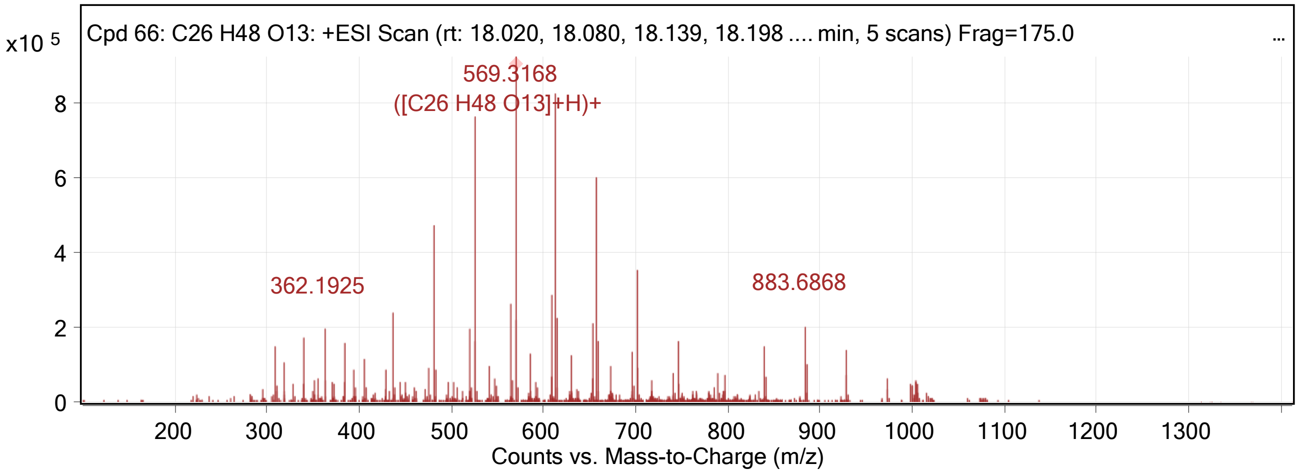

MS Zoomed Spectrum

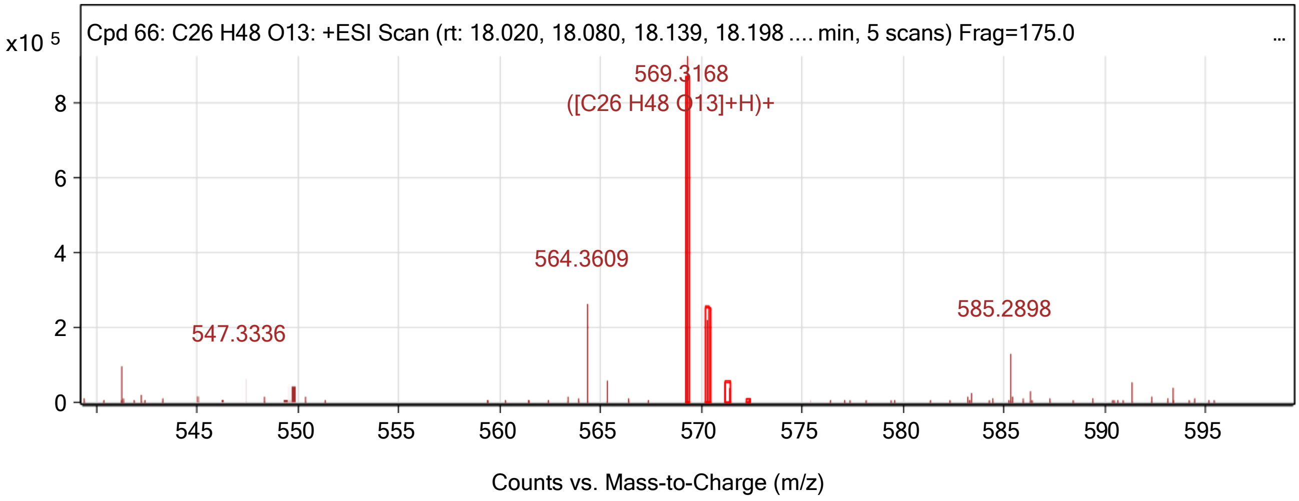

MS Spectrum Peak List

m/z	Calc *m/z*	Diff(ppm)	z	Abund	Formula	Ion
481.2636			1	473319.81		
525.2906			1	762296.13		
569.3168	569.3168	-0.06	1	925875.38	C26 H48 O13	(M+H)+
570.3195	570.3202	1.22	1	220301.63	C26 H48 O13	(M+H)+
571.3215	571.3226	1.92	1	37102.61	C26 H48 O13	(M+H)+
572.3244	572.3253	1.62	1	5044.15	C26 H48 O13	(M+H)+
608.3873			1	286128.06		
613.3433			1	826483		
657.3694			1	602299.75		
701.3955			1	355095.22		

MSMS Spectrum

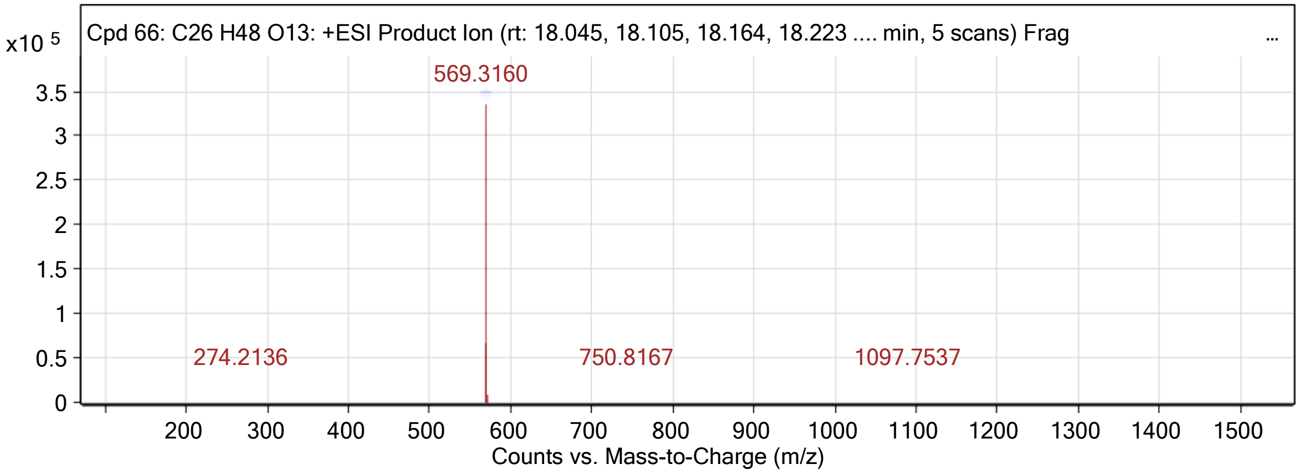

MS/MS Spectrum Peak List

m/z	Calc *m/z*	Diff(ppm)	z	Abund
133.0855	133.0859	2.84		54.79
274.2136	274.2139	1.02		30.09
309.2781	309.2788	2.37		29.13
568.3616			2	678.78
568.6124				650.81
568.8621			2	446.21
569.316	569.3168	1.37	1	335299.25
570.3185			1	66275.09
571.3207			1	7754.39
571.4074			2	844.77

Compound Label	*m/z*	RT	Algorithm	Mass
Cpd 67: C28 H52 O14	613.3431	18.359	Auto MS/MS	612.3356

Compound Chromatograms

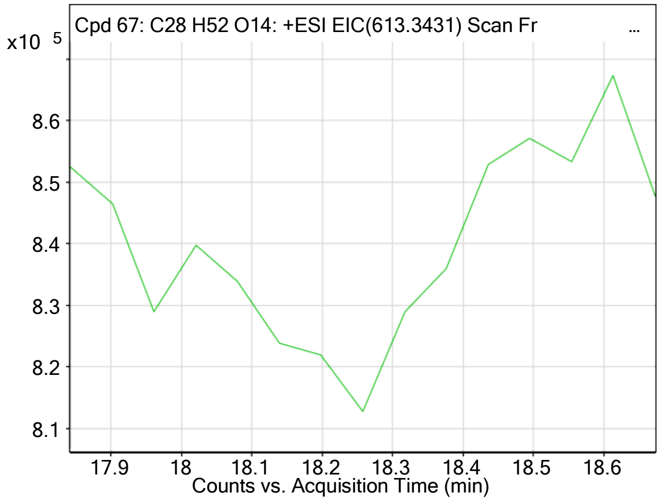

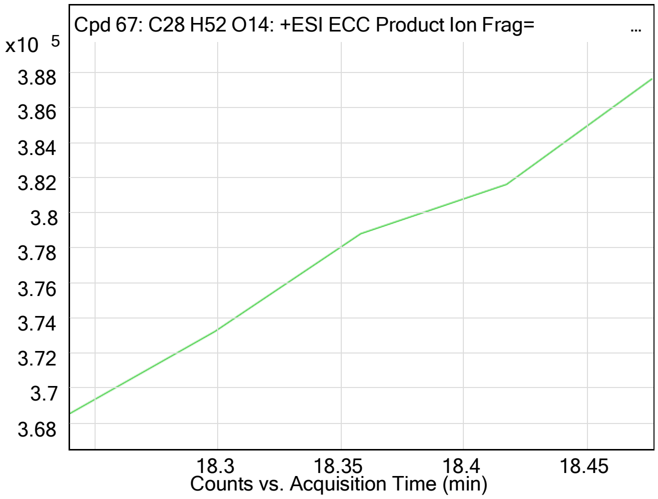

MS Spectrum

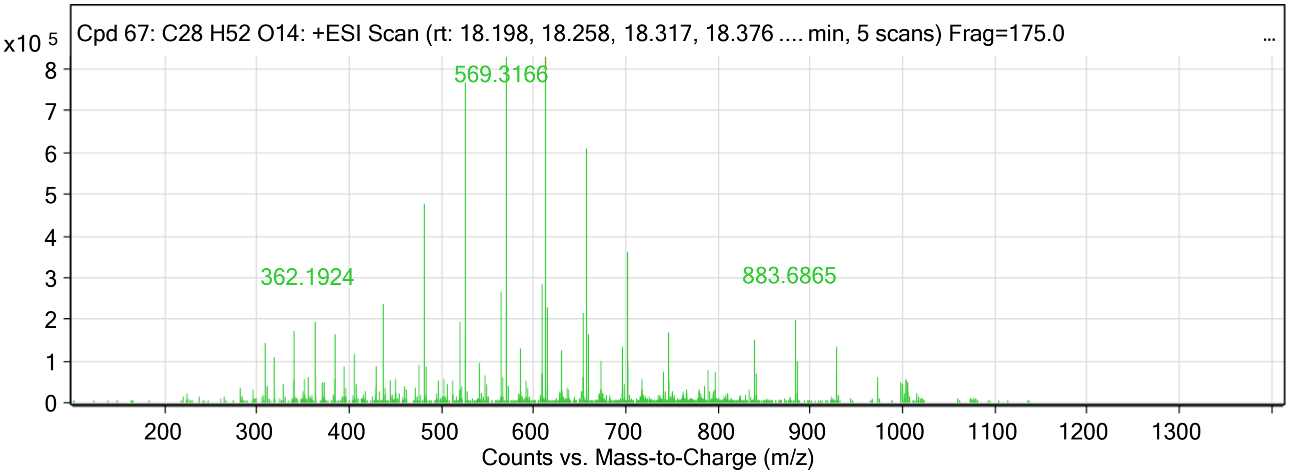

MS Zoomed Spectrum

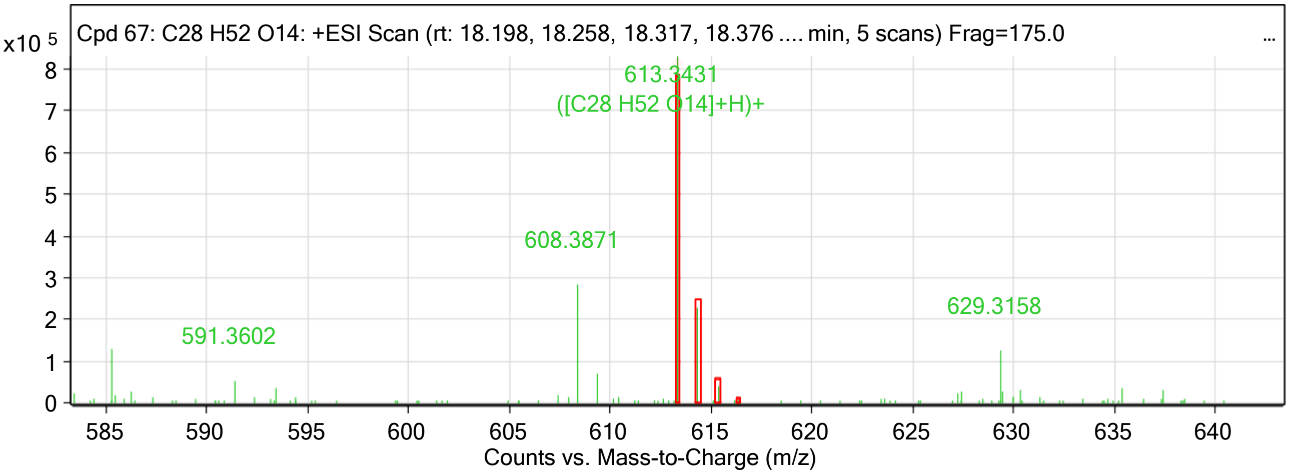

MS Spectrum Peak List

m/z	Calc *m/z*	Diff(ppm)	z	Abund	Formula	Ion
481.2634			1	474407.75		
525.2904			1	766626.19		
569.3166			1	933279.19		
608.3871			1	283249.41		
613.3431	613.343	-0.24	1	830543.88	C28 H52 O14	(M+H)+
614.3457	614.3464	1.17	1	225706.75	C28 H52 O14	(M+H)+
615.3476	615.3489	1.97	1	39549.45	C28 H52 O14	(M+H)+
616.3548	616.3515	-5.36	1	5543.01	C28 H52 O14	(M+H)+

MSMS Spectrum

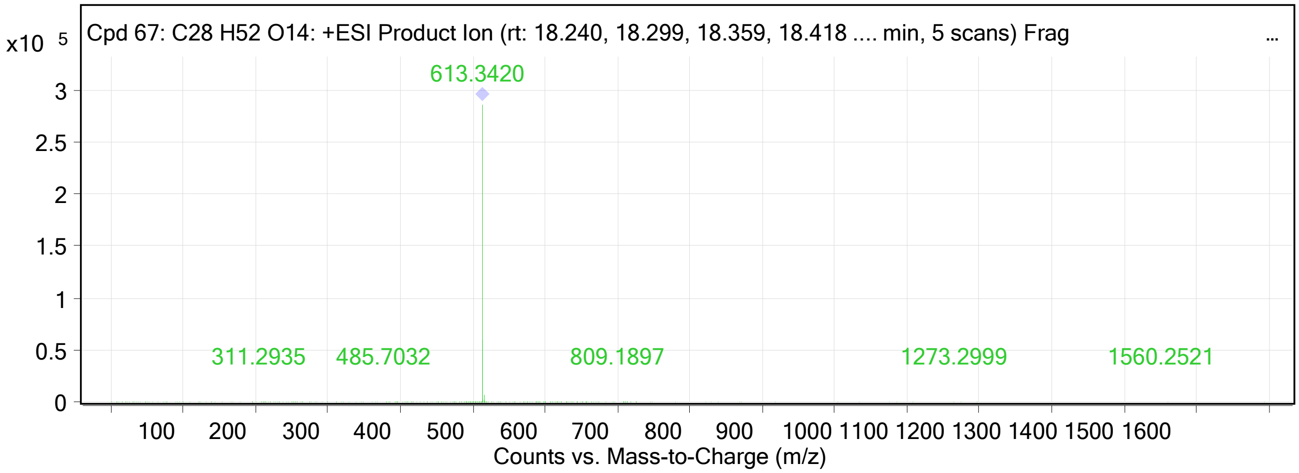

MS/MS Spectrum Peak List

m/z	*Calc m/z*	Diff(ppm)	z	Abund
309.2786	309.2788	0.67	1	72.33
612.3871			2	2623.38
612.6389				3152
612.8891			2	2734.38
613.14			1	1261.18
613.342	613.343	1.68	1	286248.44
614.3446			1	59301.96
615.1412			1	1005.97
615.347			1	7285.03
615.4246			2	923.83

Compound Label	*m/z*	RT	Algorithm	Mass
Cpd 68: C24 H46 N3 O12	569.3165	18.46	Auto MS/MS	568.3091

Compound Chromatograms

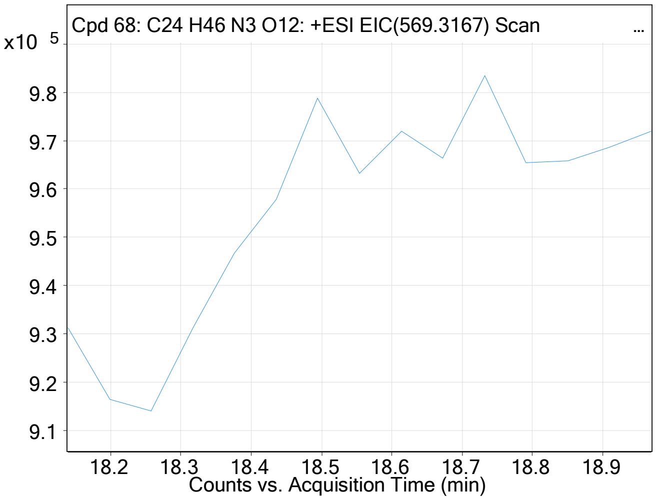

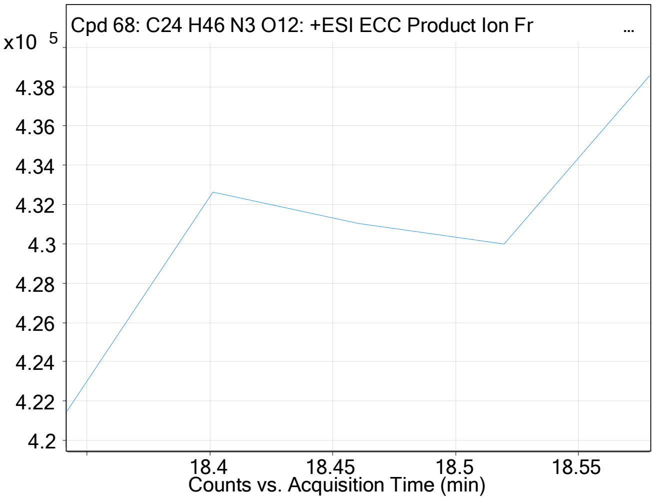

MS Spectrum

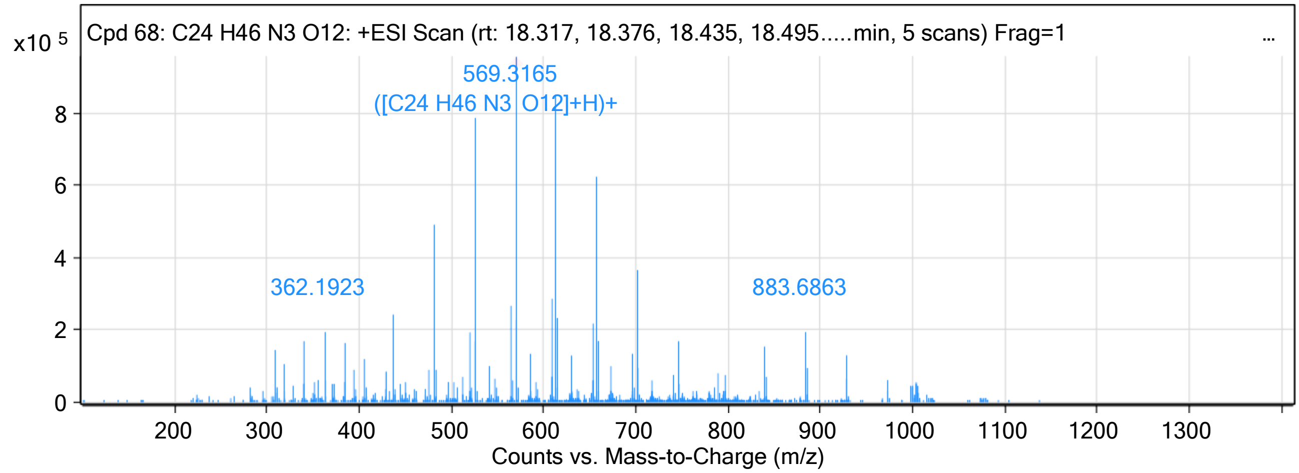

MS Zoomed Spectrum

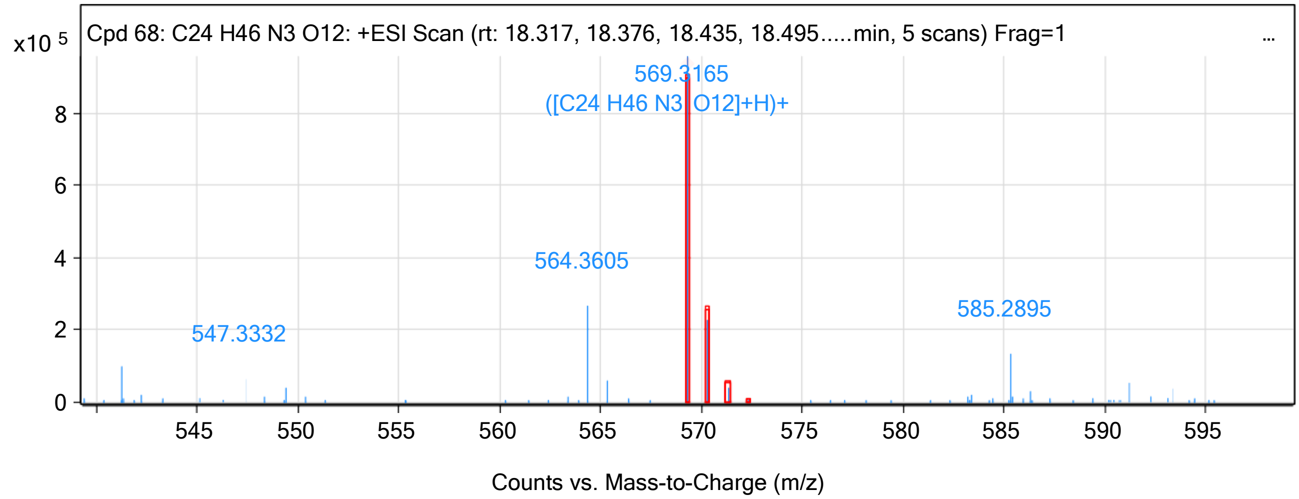

MS Spectrum Peak List

m/z	Calc *m/z*	Diff(ppm)	z	Abund	Formula	Ion
481.2633			1	486575.31		
525.2903			1	782744.13		
569.3165	569.3154	-1.89	1	955572.19	C24 H46 N3 O12	(M+H)+
570.3192	570.3186	-1.02	1	225768.09	C24 H46 N3 O12	(M+H)+
571.3213	571.3209	-0.61	1	38387.57	C24 H46 N3 O12	(M+H)+
572.3238	572.3235	-0.42	1	5402.2	C24 H46 N3 O12	(M+H)+
608.3869			1	285653.84		
613.343			1	845679.38		
657.369			1	620297.19		
701.3951			1	366203.59		

MSMS Spectrum

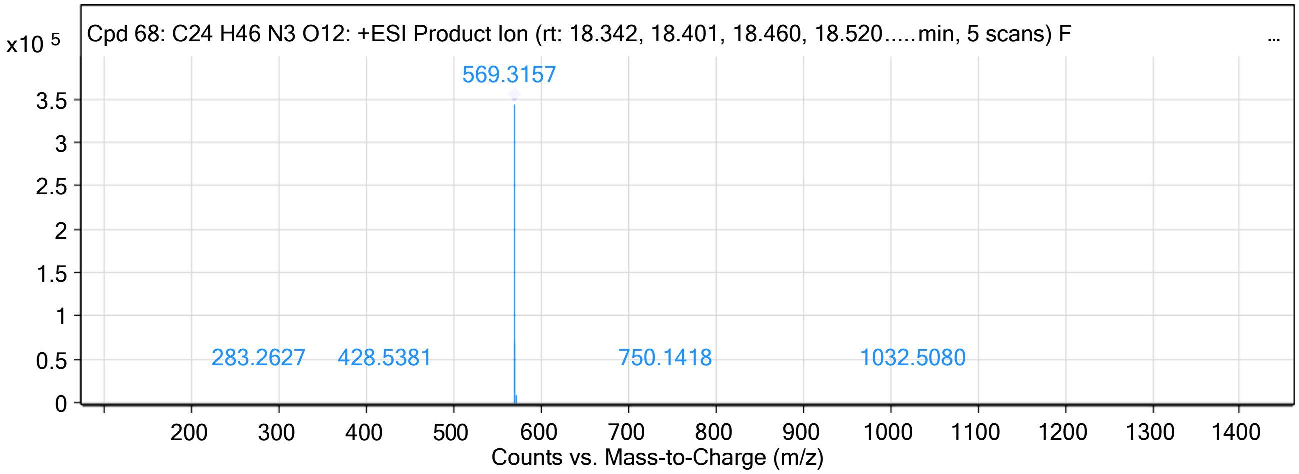

MS/MS Spectrum Peak List

m/z	*Calc m/z*	Diff(ppm)	z	Abund
133.0859	133.0859	0.08		46.51
175.1312	175.1315	1.61		17.58
177.1109	177.1108	-0.46		30.93
275.1586	275.1601	5.75		22.36
283.2627	283.2632	1.72		30.48
309.2761	309.2775	4.28		29.33
510.2696	510.2671	-4.89		17.67
513.2501	513.2528	5.39		21.71
533.3213	533.3154	-11.01	1	133.12
569.3157	569.3154	-0.43	1	343641.16

Compound Label	*m/z*	RT	Algorithm	Mass
Cpd 69: C28 H52 O14	613.3431	18.655	Auto MS/MS	612.3356

Compound Chromatograms

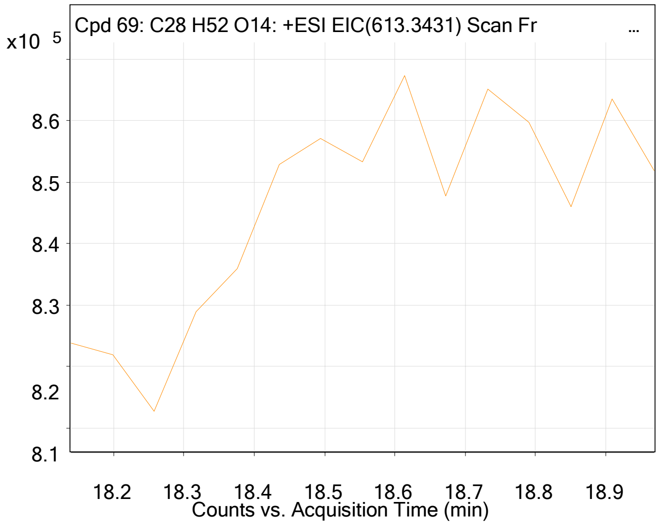

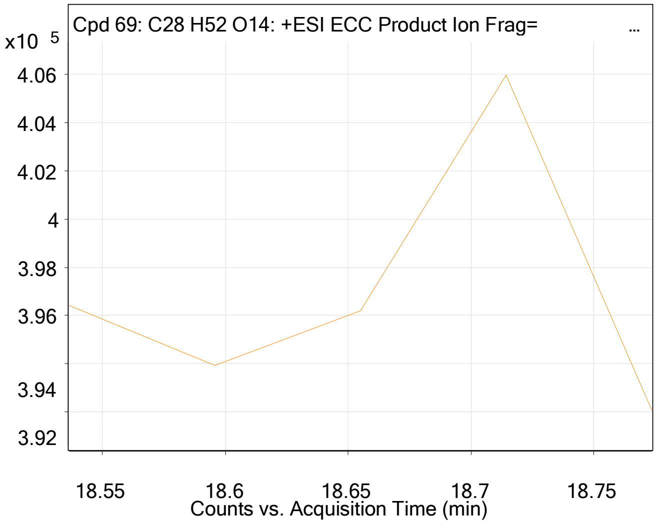

MS Spectrum

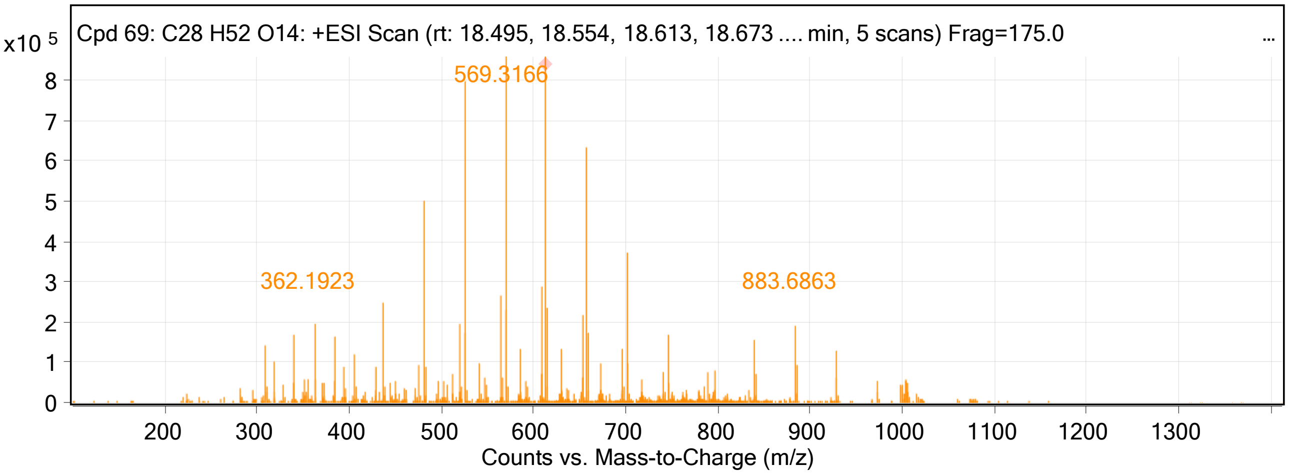

MS Zoomed Spectrum

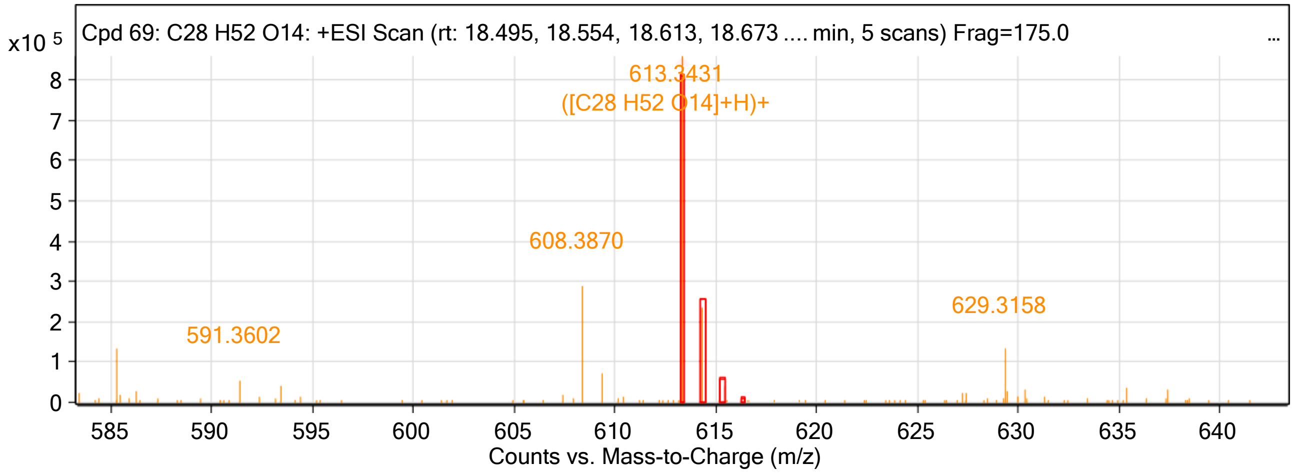

MS Spectrum Peak List

m/z	Calc *m/z*	Diff(ppm)	z	Abund	Formula	Ion
481.2634			1	500254.44		
525.2903			1	796595.44		
569.3166			1	972753.63		
608.387			1	288078.69		
613.3431	613.343	-0.14	1	858146.5	C28 H52 O14	(M+H)+
614.3457	614.3464	1.22	1	233178.63	C28 H52 O14	(M+H)+
615.3476	615.3489	2.08	1	42080.64	C28 H52 O14	(M+H)+
616.3551	616.3515	-5.72	1	5899.26	C28 H52 O14	(M+H)+
657.3691			1	633993.44		
701.3952			1	372688.16		

MSMS Spectrum

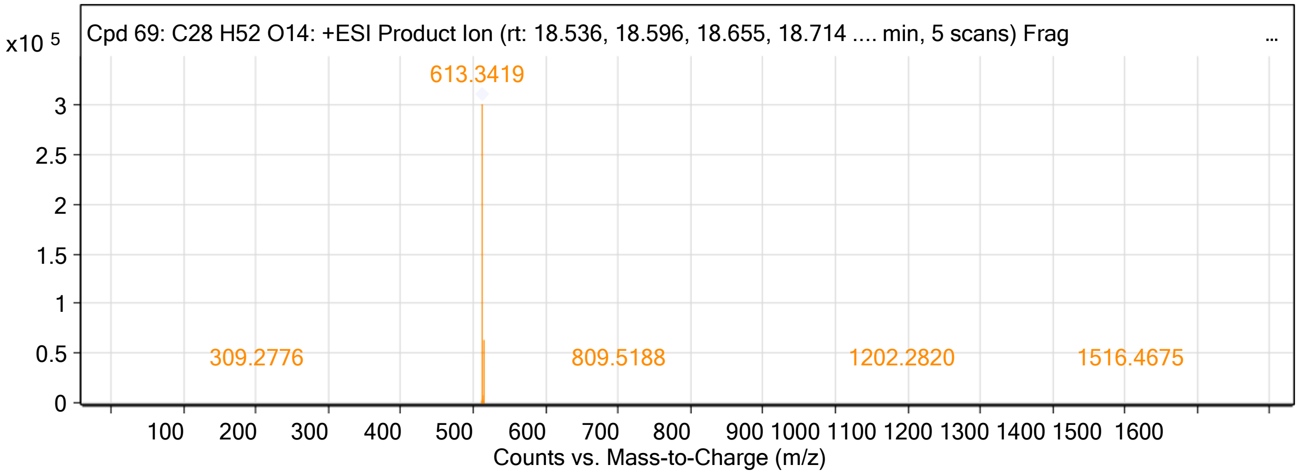

MS/MS Spectrum Peak List

m/z	*Calc m/z*	Diff(ppm)	z	Abund
117.0911	117.091	-1.12		41.13
309.2776	309.2788	4.02		104.61
311.2936	311.2945	2.66	1	95.57
612.3882			2	2598.53
612.6389			2	3131.89
612.8892			2	2535.5
613.1391			2	1406.79
613.3419	613.343	1.79	1	301087.75
614.3446			1	63135.57
615.3469			1	7681.89

Compound Label	*m/z*	RT	Algorithm	Mass
Cpd 70: C24 H46 N3 O12	569.3166	18.757	Auto MS/MS	568.3092

Compound Chromatograms

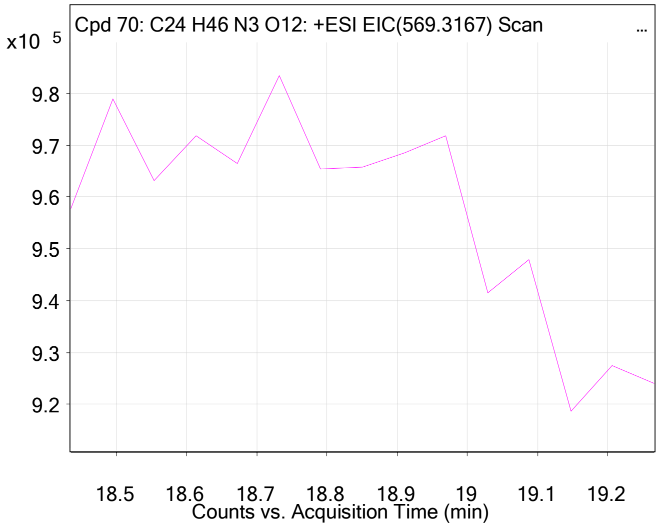

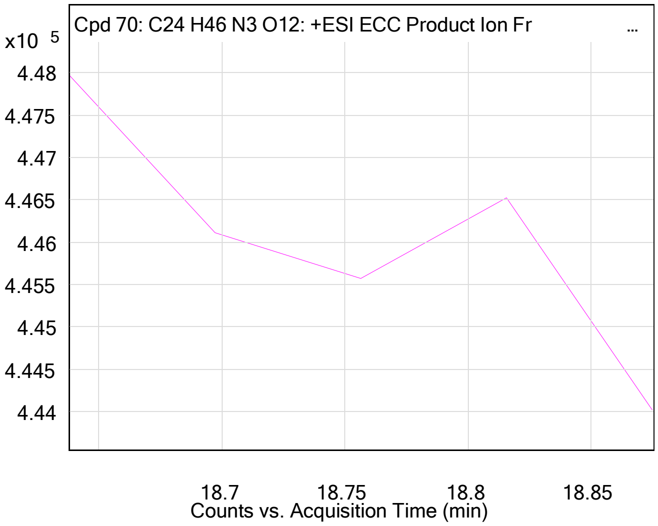

MS Spectrum

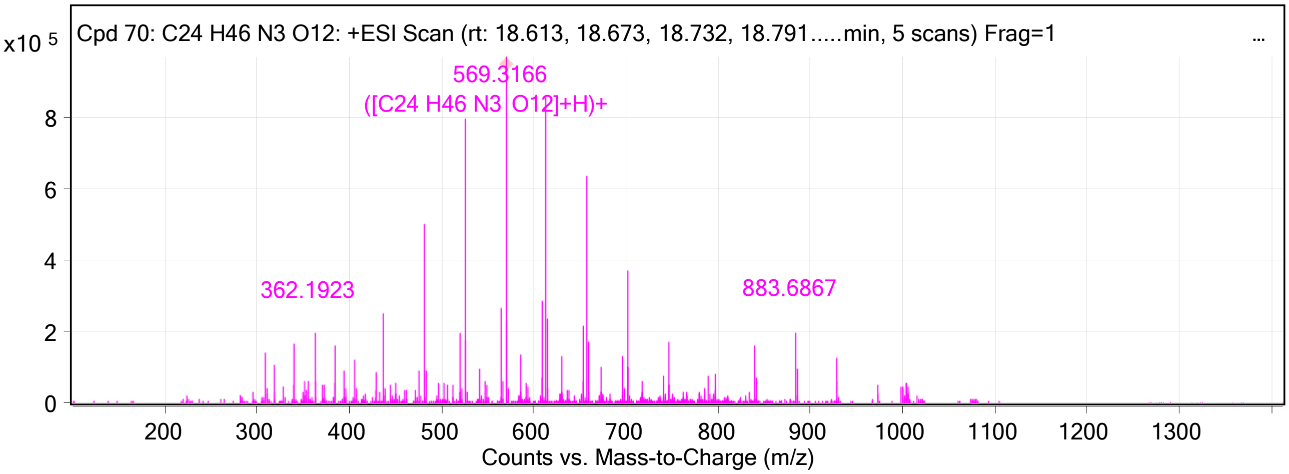

MS Zoomed Spectrum

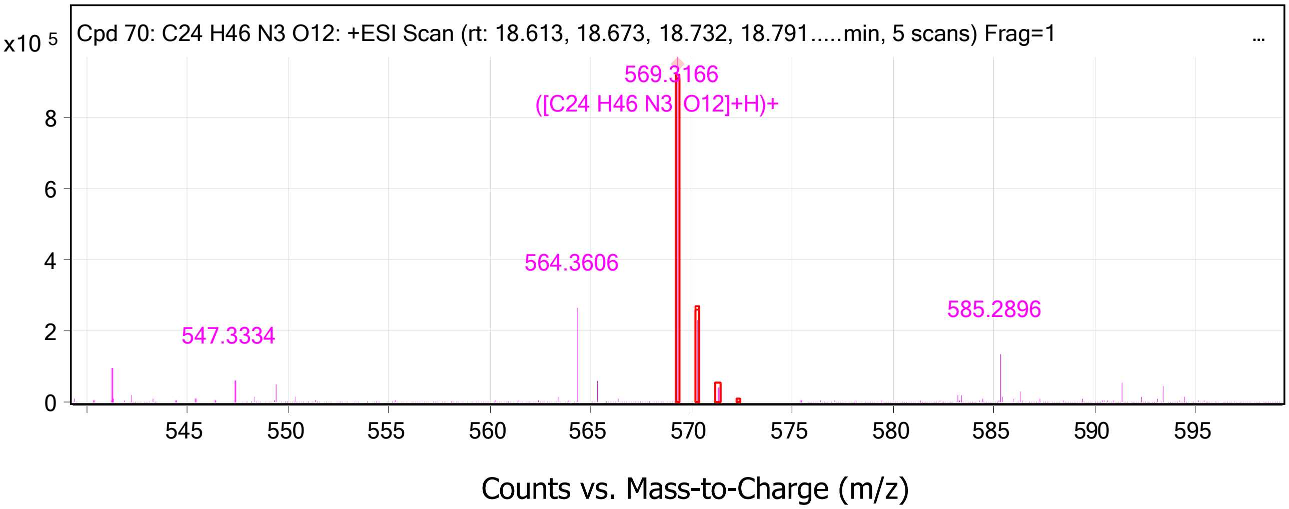

MS Spectrum Peak List

m/z	Calc *m/z*	Diff(ppm)	z	Abund	Formula	Ion
481.2634			1	501283.59		
525.2903			1	794213.06		
569.3166	569.3154	-2.14	1	970616.38	C24 H46 N3 O12	(M+H)+
570.3193	570.3186	-1.25	1	228159.33	C24 H46 N3 O12	(M+H)+
571.3214	571.3209	-0.86	1	38808.07	C24 H46 N3 O12	(M+H)+
572.3243	572.3235	-1.29	1	5550.11	C24 H46 N3 O12	(M+H)+
608.3871			1	286012.56		
613.3432			1	857215.81		
657.3692			1	635595.81		

MSMS Spectrum

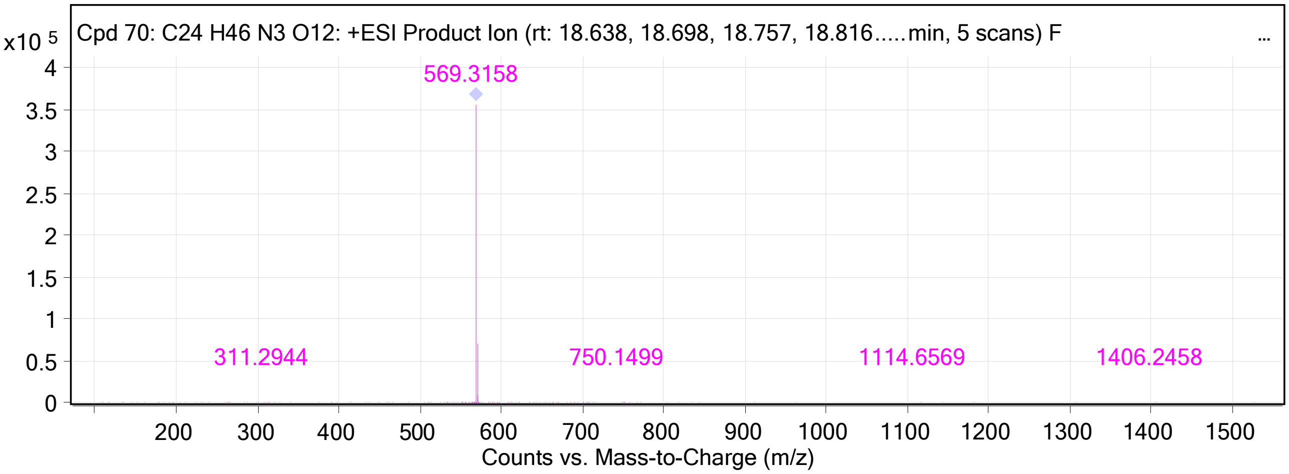

MS/MS Spectrum Peak List

m/z	*Calc m/z*	Diff(ppm)	z	Abund
108.0863	108.0808	-50.89		20.95
144.0794	144.0781	-9.39		16.67
195.1221	195.1214	-3.75		22.58
195.9877	195.985	-13.96		33.69
264.1953	264.1958	1.94		16.1
287.1865	287.1853	-4.12		22.37
311.2944	311.2945	0.05		54.31
412.2986	412.3017	7.68		17.38
533.3227	533.3154	-13.58	1	66.36
569.3158	569.3154	-0.63	1	355951.53

Compound Label	*m/z*	RT	Algorithm	Mass
Cpd 71: C28 H52 O14	613.3431	18.951	Auto MS/MS	612.3357

Compound Chromatograms

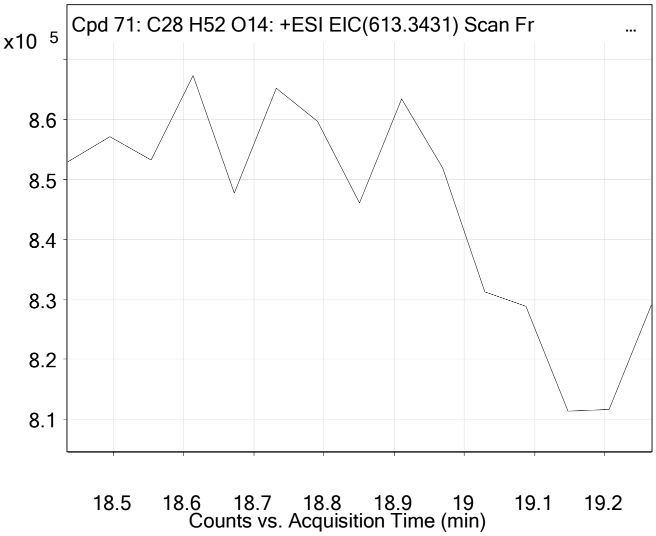

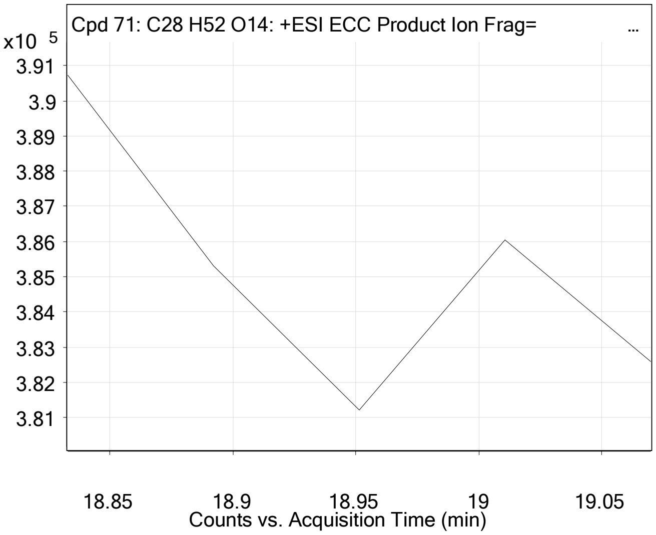

MS Spectrum

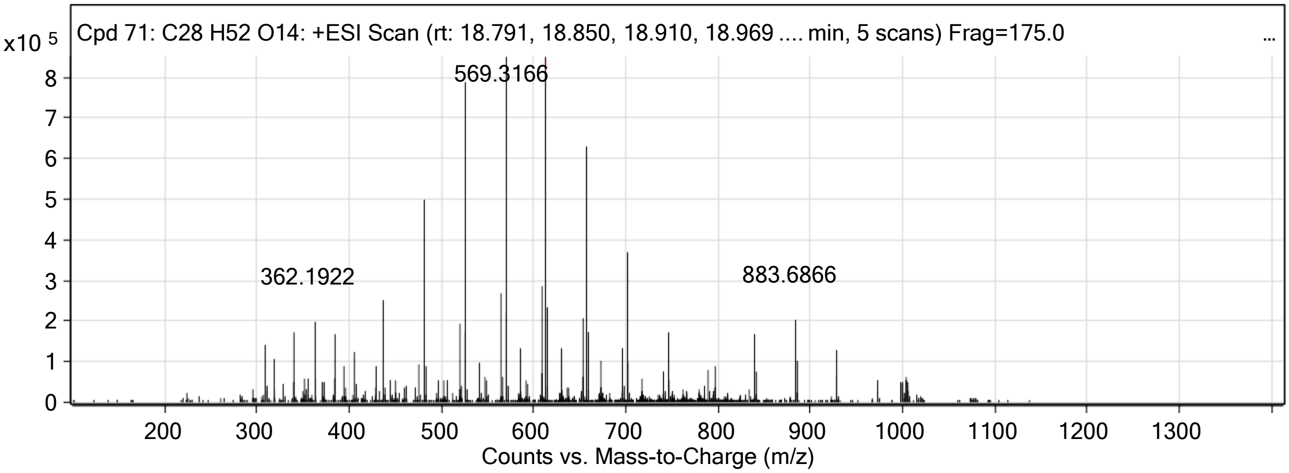

MS Zoomed Spectrum

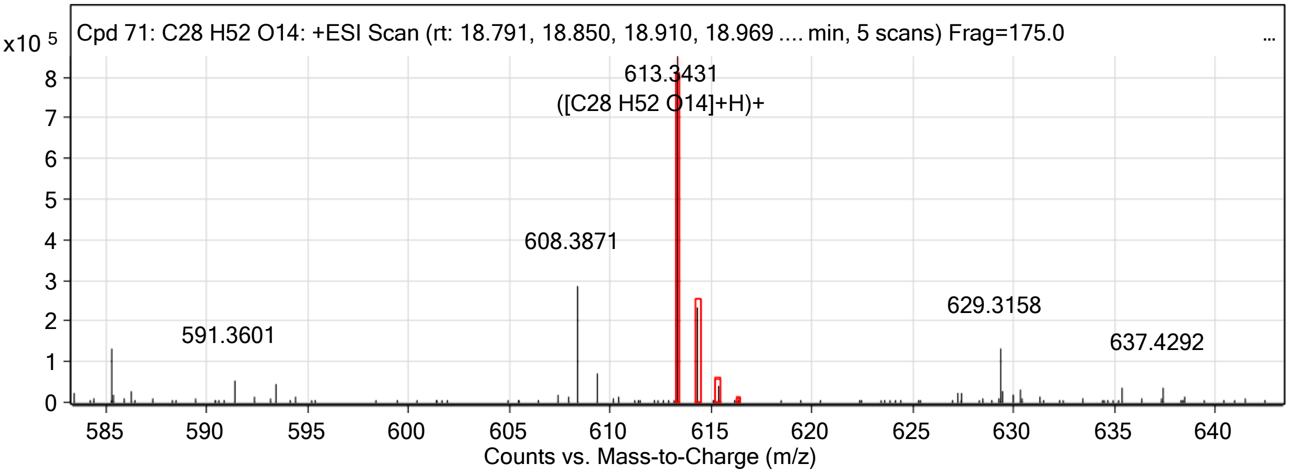

MS Spectrum Peak List

m/z	Calc *m/z*	Diff(ppm)	z	Abund	Formula	Ion
481.2634			1	494913.94		
525.2903			1	785217.5		
569.3166			1	962696.5		
608.3871			1	284059.41		
613.3431	613.343	-0.27	1	850508.81	C28 H52 O14	(M+H)+
614.3457	614.3464	1.17	1	232425.7	C28 H52 O14	(M+H)+
615.3476	615.3489	1.97	1	41270.75	C28 H52 O14	(M+H)+
616.3538	616.3515	-3.72	1	6052.58	C28 H52 O14	(M+H)+
657.3692			1	627421.81		
701.3953			1	367611.69		

MSMS Spectrum

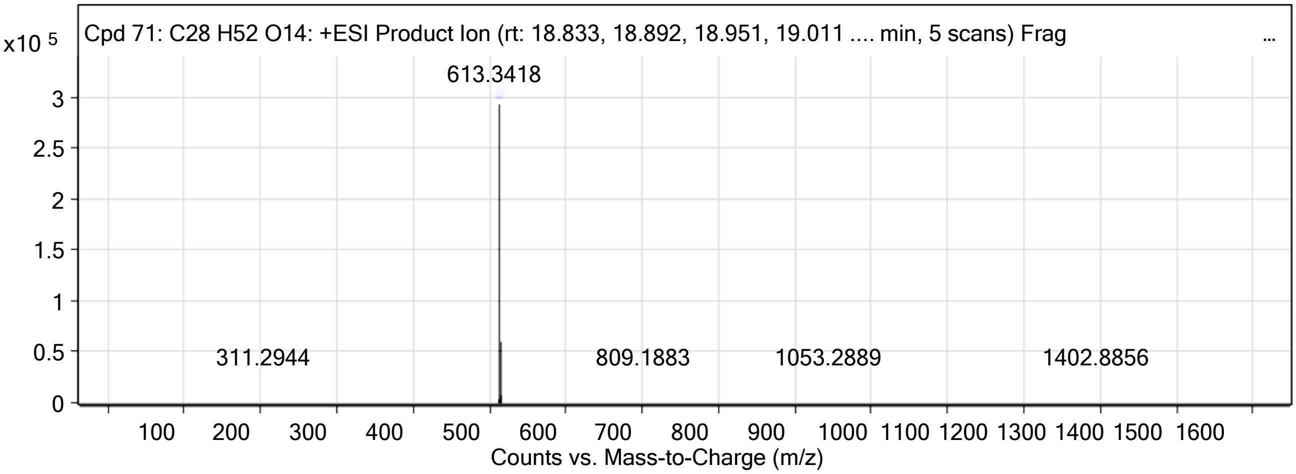

MS/MS Spectrum Peak List

m/z	*Calc m/z*	Diff(ppm)	z	Abund
133.0856	133.0859	2.04		90.52
309.2797	309.2788	-2.81		50.3
311.2944	311.2945	0.19	1	105.22
357.0666	357.0664	-0.55		56.3
609.3047	304.6556	-499994.69	2	42.72
612.3876			2	2706.39
612.6381				3273.33
613.3418	613.343	1.91	1	292827.75
614.3446			1	60240.28
615.3469			1	7703.74

Compound Label	*m/z*	RT	Algorithm	Mass
Cpd 72: C24 H46 N3 O12	569.3166	19.053	Auto MS/MS	568.3092

Compound Chromatograms

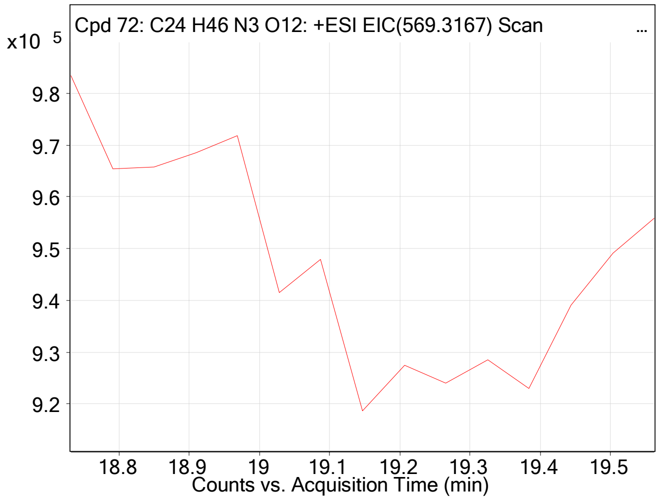

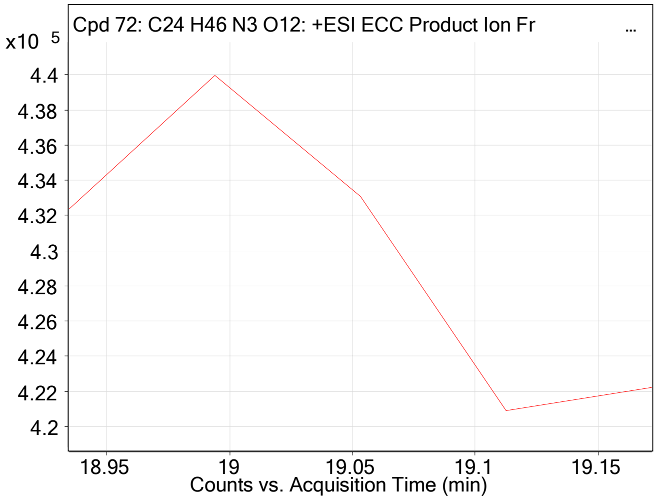

MS Spectrum

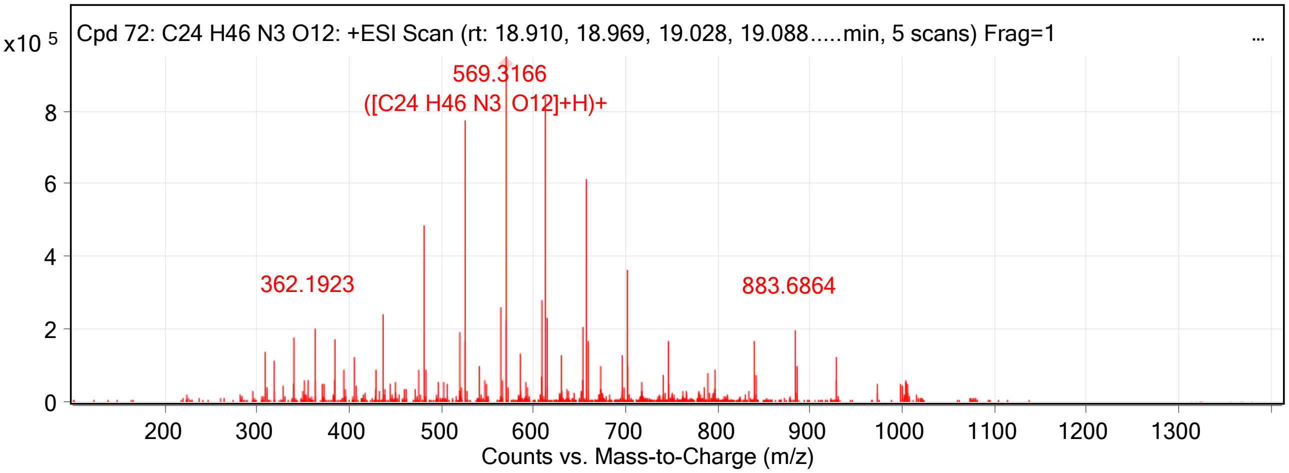

MS Zoomed Spectrum

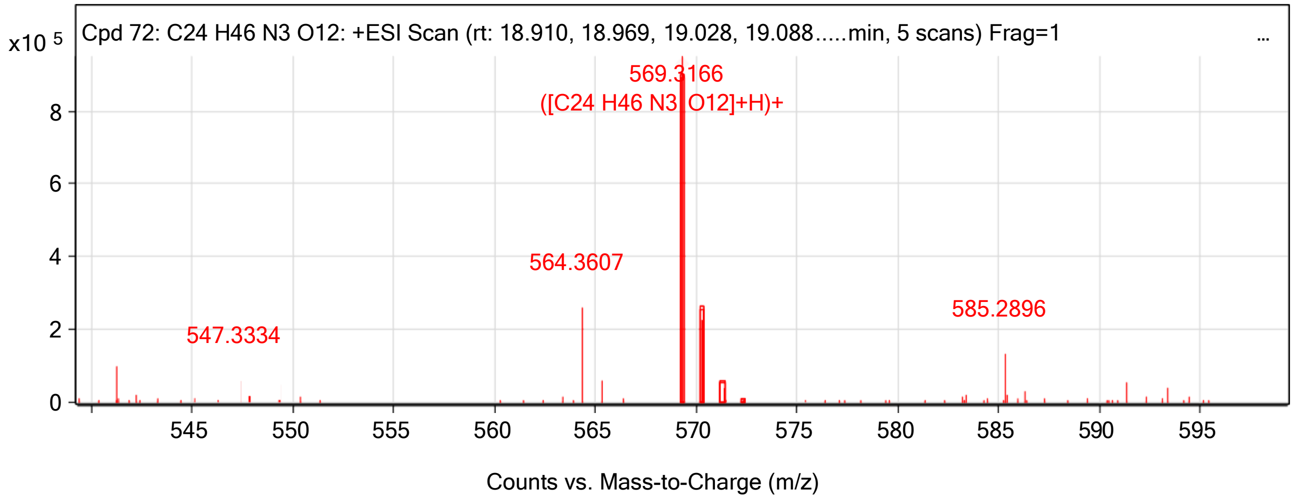

MS Spectrum Peak List

m/z	Calc *m/z*	Diff(ppm)	z	Abund	Formula	Ion
481.2634			1	484513.91		
525.2904			1	774252.88		
569.3166	569.3154	-2.15	1	949752.31	C24 H46 N3 O12	(M+H)+
570.3193	570.3186	-1.22	1	224682	C24 H46 N3 O12	(M+H)+
571.3214	571.3209	-0.73	1	37767.02	C24 H46 N3 O12	(M+H)+
572.3242	572.3235	-1.13	1	5356.59	C24 H46 N3 O12	(M+H)+
608.3871			1	278416.25		
613.3431			1	837392		
657.3691			1	611239.38		
701.3953			1	361517.56		

MSMS Spectrum

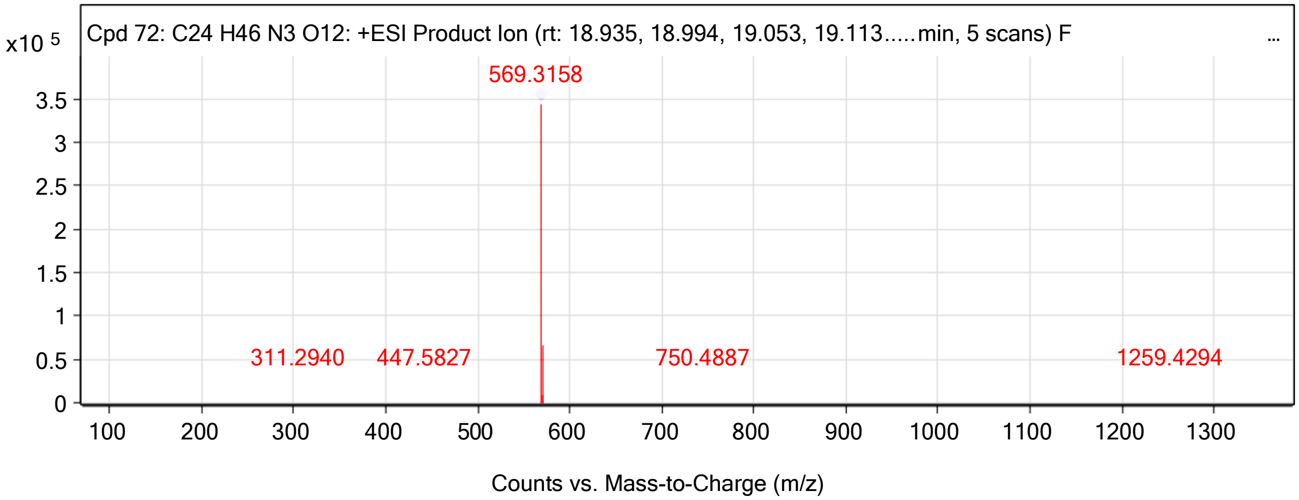

MS/MS Spectrum Peak List

m/z	*Calc m/z*	Diff(ppm)	z	Abund
117.0909	117.091	0.63		26.76
133.0866	133.0859	-4.82		35.61
177.111	177.1108	-1.42		28.56
195.122	195.1214	-3.15		15.99
195.146	195.1492	16.58		16.2
307.1739	307.1738	-0.22		14.4
311.294	311.2945	1.33		50.57
312.2973	312.3009	11.73		21.65
371.2245	371.2262	4.74		19.67
569.3158	569.3154	-0.65	1	343717.03

Compound Label	*m/z*	RT	Algorithm	Mass
Cpd 73: C28 H52 O14	613.3429	19.248	Auto MS/MS	612.3354

Compound Chromatograms

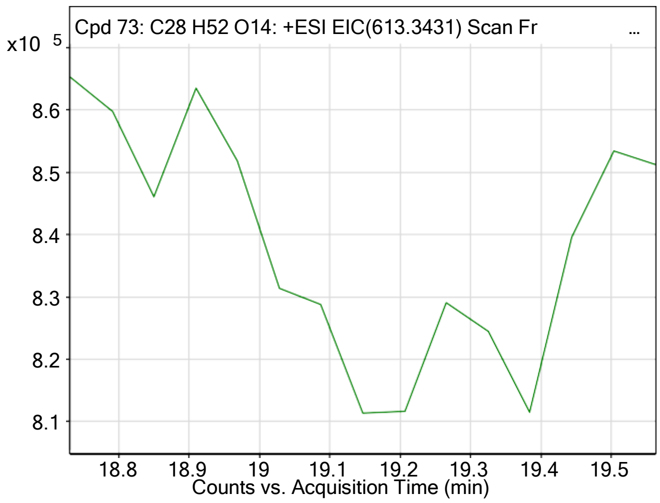

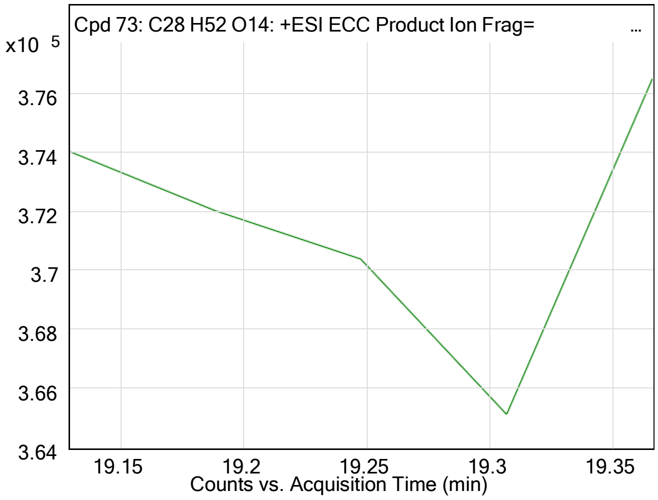

MS Spectrum

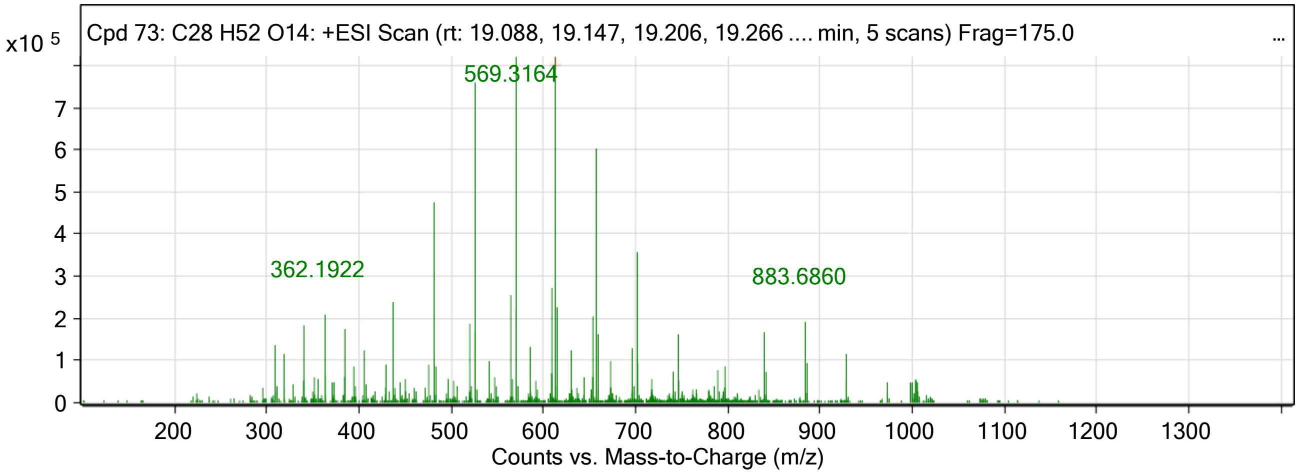

MS Zoomed Spectrum

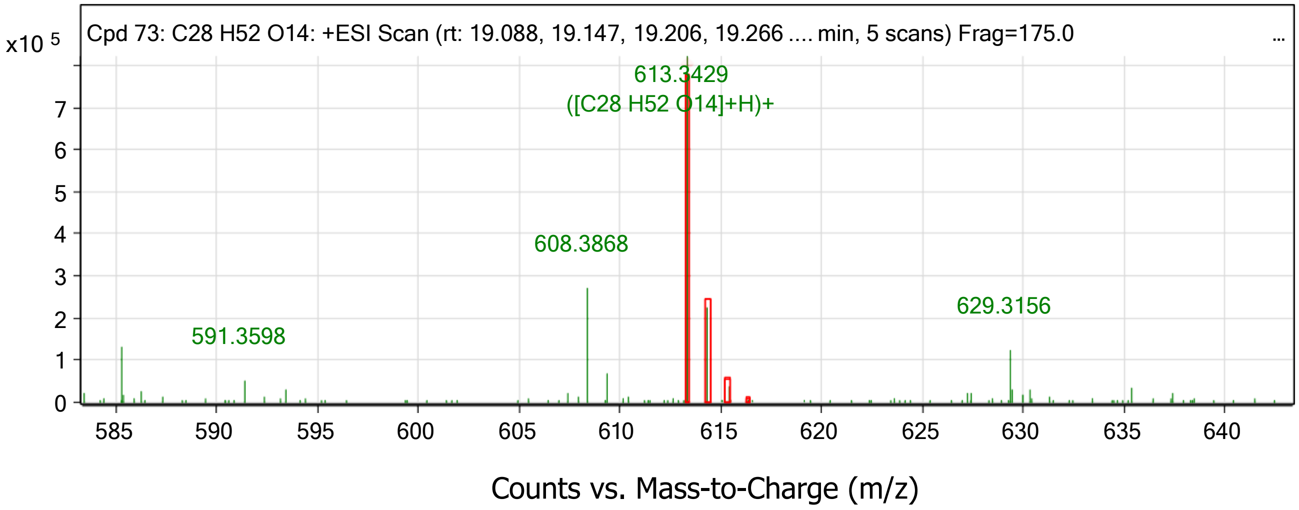

MS Spectrum Peak List

m/z	Calc *m/z*	Diff(ppm)	z	Abund	Formula	Ion
481.2632			1	474488.19		
525.2902			1	757965.38		
569.3164			1	929368.38		
608.3868			1	272291.59		
613.3429	613.343	0.14	1	821093.81	C28 H52 O14	(M+H)+
614.3455	614.3464	1.55	1	223164.05	C28 H52 O14	(M+H)+
615.3474	615.3489	2.29	1	39371.34	C28 H52 O14	(M+H)+
616.3553	616.3515	-6.11	1	5464.25	C28 H52 O14	(M+H)+

MSMS Spectrum

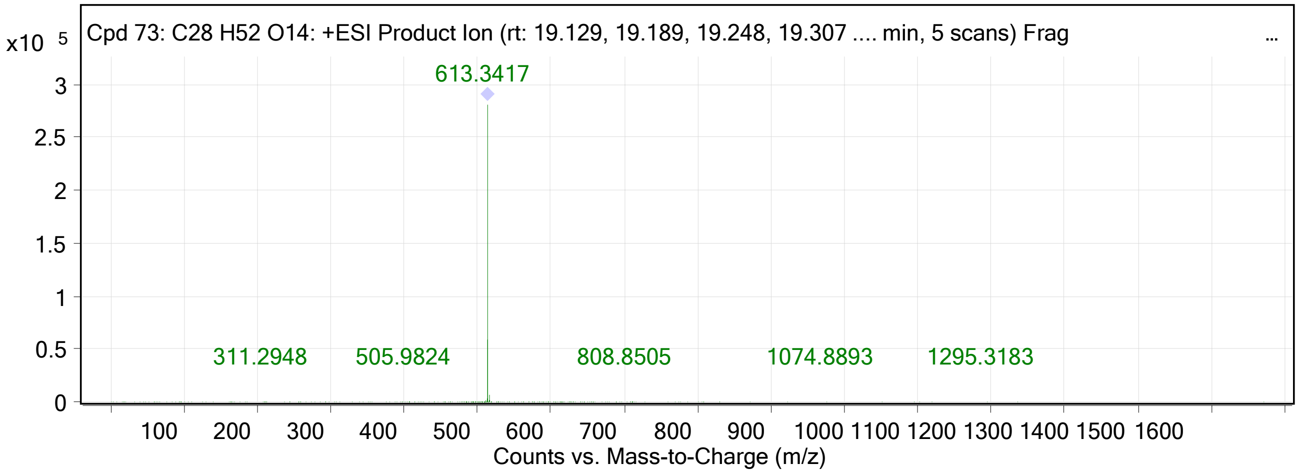

MS/MS Spectrum Peak List

m/z	*Calc m/z*	Diff(ppm)	z	Abund
311.2948	311.2945	-1.23		86.26
312.2977	312.3023	14.79		50.95
357.0689	357.0664	-7.13	1	40.89
612.3875			2	2479.21
612.6384			2	3085.39
612.8885			2	2631.04
613.1406			2	1446.79
613.3417	613.343	2.11	1	281031.31
614.3444			1	59240.95
615.3467			1	7176.95

Compound Label	*m/z*	RT	Algorithm	Mass
Cpd 74: C24 H46 N3 O12	569.3163	19.35	Auto MS/MS	568.3089

Compound Chromatograms

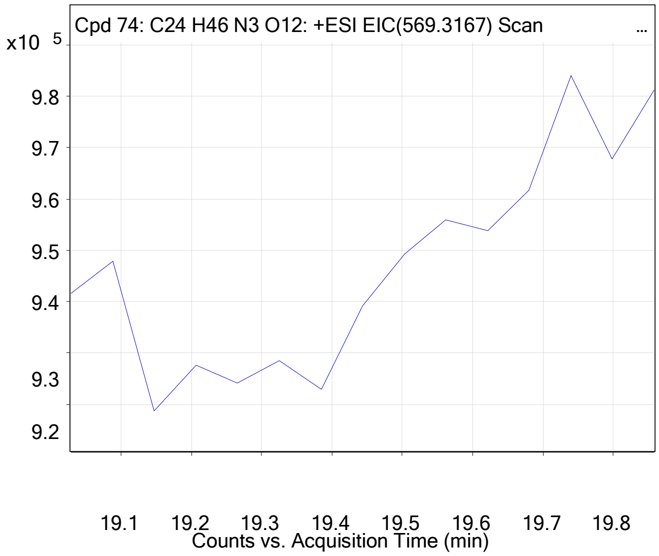

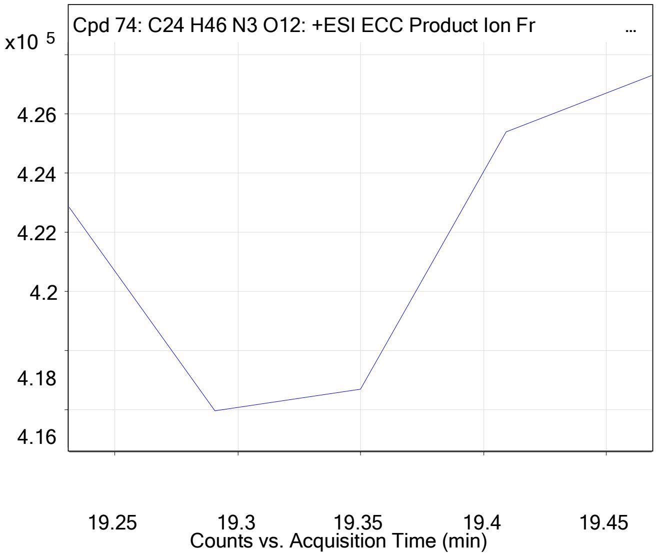

MS Spectrum

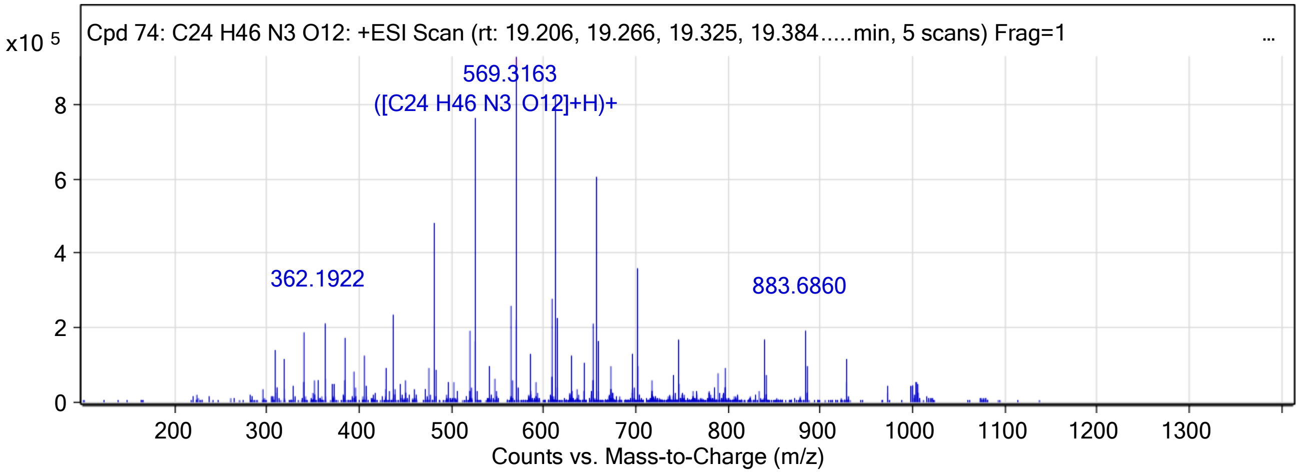

MS Zoomed Spectrum

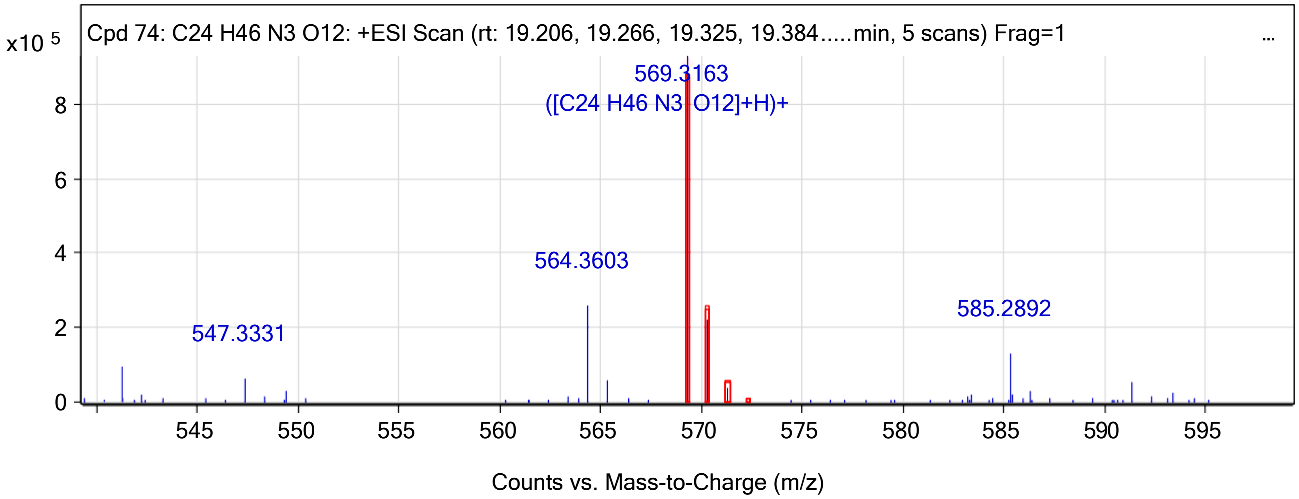

MS Spectrum Peak List

m/z	Calc *m/z*	Diff(ppm)	z	Abund	Formula	Ion
481.2632			1	478007		
525.2901			1	759178.31		
569.3163	569.3154	-1.55	1	928466.31	C24 H46 N3 O12	(M+H)+
570.319	570.3186	-0.71	1	221630.09	C24 H46 N3 O12	(M+H)+
571.3211	571.3209	-0.25	1	37321.54	C24 H46 N3 O12	(M+H)+
572.3238	572.3235	-0.42	1	5126.16	C24 H46 N3 O12	(M+H)+
608.3867			1	277992.91		
613.3428			1	823280.13		
657.3688			1	603302		
701.395			1	359565.44		

MSMS Spectrum

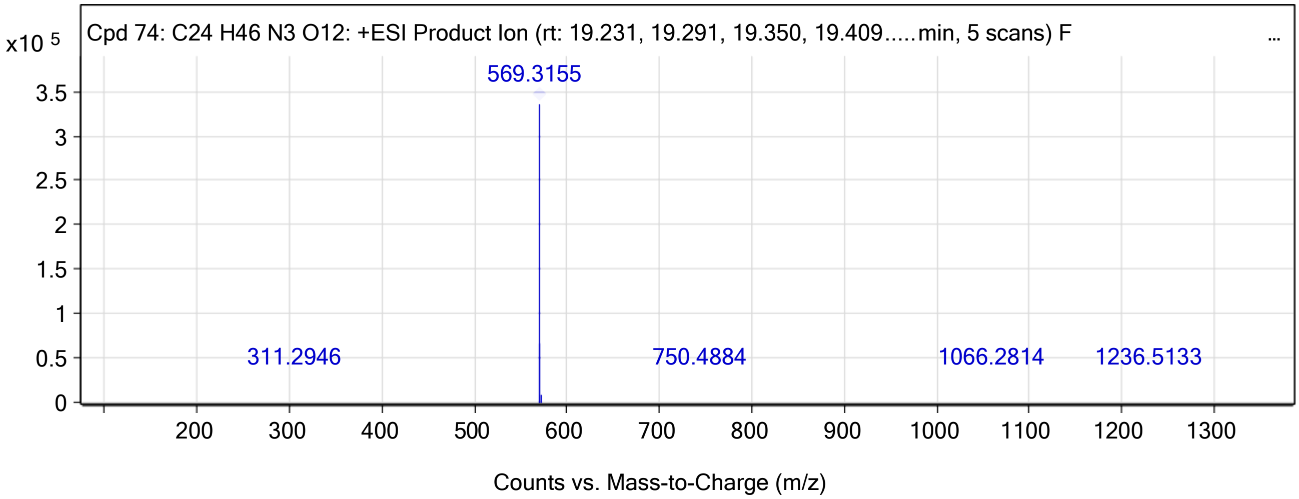

MS/MS Spectrum Peak List

m/z	*Calc m/z*	Diff(ppm)	z	Abund
151.0963	151.0951	-7.8		23.34
195.1222	195.1214	-4.23		27.97
254.1423	254.1387	-14.23		14.03
262.1958	262.1927	-11.53		24.17
283.2633	283.2632	-0.41		18
309.2783	309.2788	1.78		30.83
311.2946	311.2945	-0.5		61.25
566.2857	283.1457	-499994.95	2	43.04
569.3155	569.3154	-0.14	1	335627
570.318			1	66946.32

Compound Label	*m/z*	RT	Algorithm	Mass
Cpd 75: C28 H52 O14	613.343	19.544	Auto MS/MS	612.3355

Compound Chromatograms

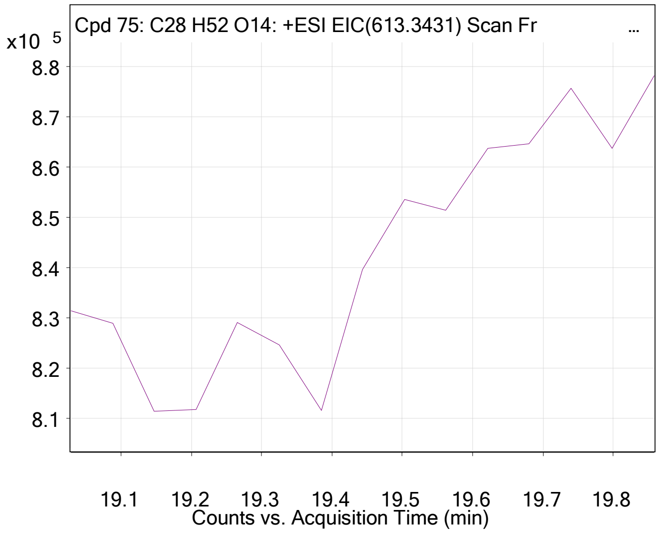

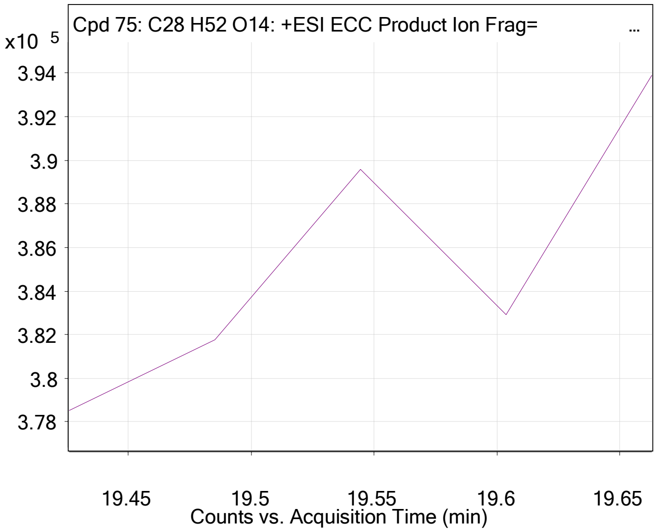

MS Spectrum

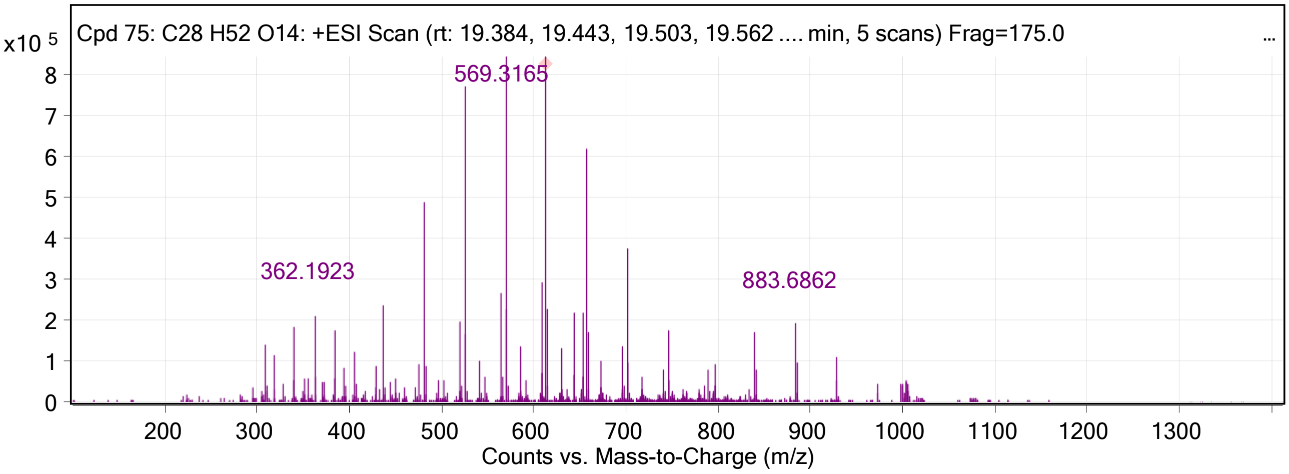

MS Zoomed Spectrum

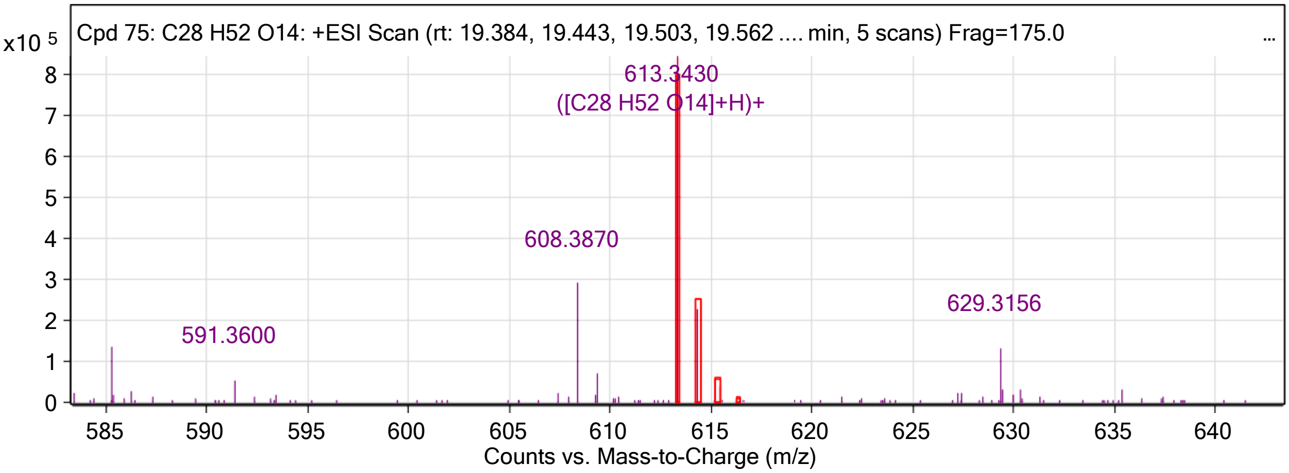

MS Spectrum Peak List

m/z	Calc *m/z*	Diff(ppm)	z	Abund	Formula	Ion
481.2633			1	486582.91		
525.2902			1	771782.38		
569.3165			1	944226		
608.387			1	289551.69		
613.343	613.343	0.05	1	843929.81	C28 H52 O14	(M+H)+
614.3455	614.3464	1.4	1	228473.59	C28 H52 O14	(M+H)+
615.3475	615.3489	2.19	1	41469.36	C28 H52 O14	(M+H)+
616.3549	616.3515	-5.52	1	5574.86	C28 H52 O14	(M+H)+
657.369			1	620132.19		
701.3952			1	372892.25		

MSMS Spectrum

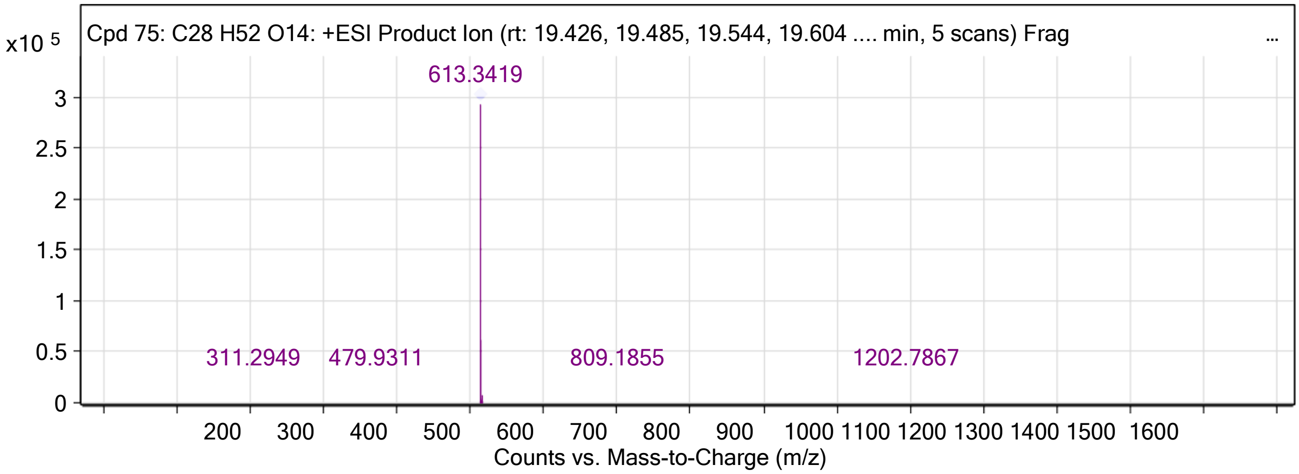

MS/MS Spectrum Peak List

m/z	*Calc m/z*	Diff(ppm)	z	Abund
133.0863	133.0859	-3.02		53.48
309.2781	309.2788	2.21	1	54.37
612.3881			2	2459.06
612.6386				3053.22
612.8893			2	2529.44
613.1408			1	1383.53
613.3419	613.343	1.77	1	292887.53
614.3445			1	61240.73
615.3469			1	7404.53
615.4246			2	1123.99

Compound Label	*m/z*	RT	Algorithm	Mass
Cpd 76: C24 H46 N3 O12	569.3166	19.646	Auto MS/MS	568.3092

Compound Chromatograms

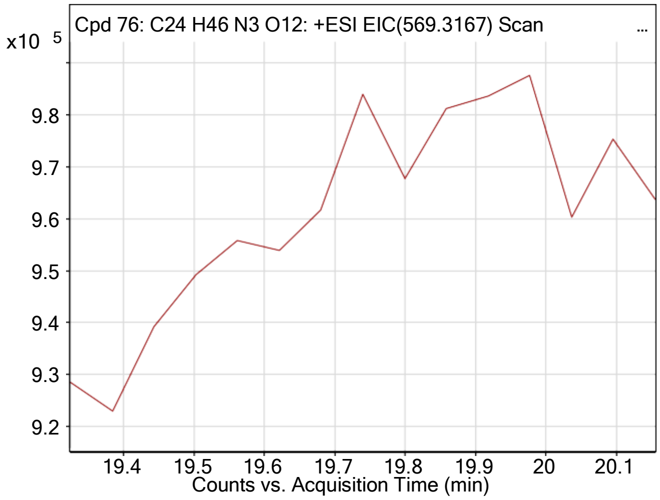

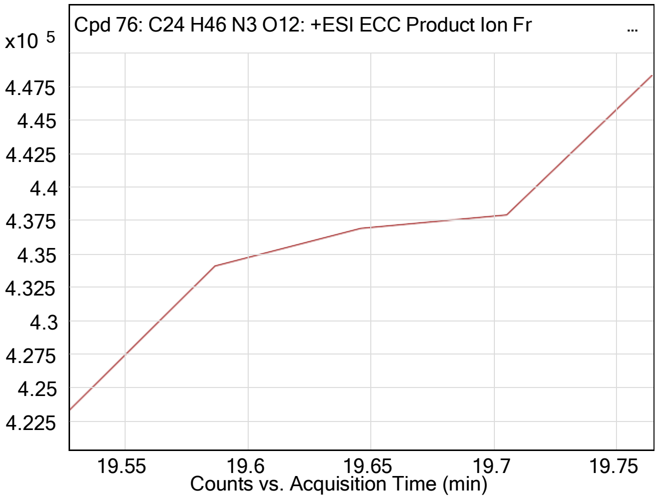

MS Spectrum

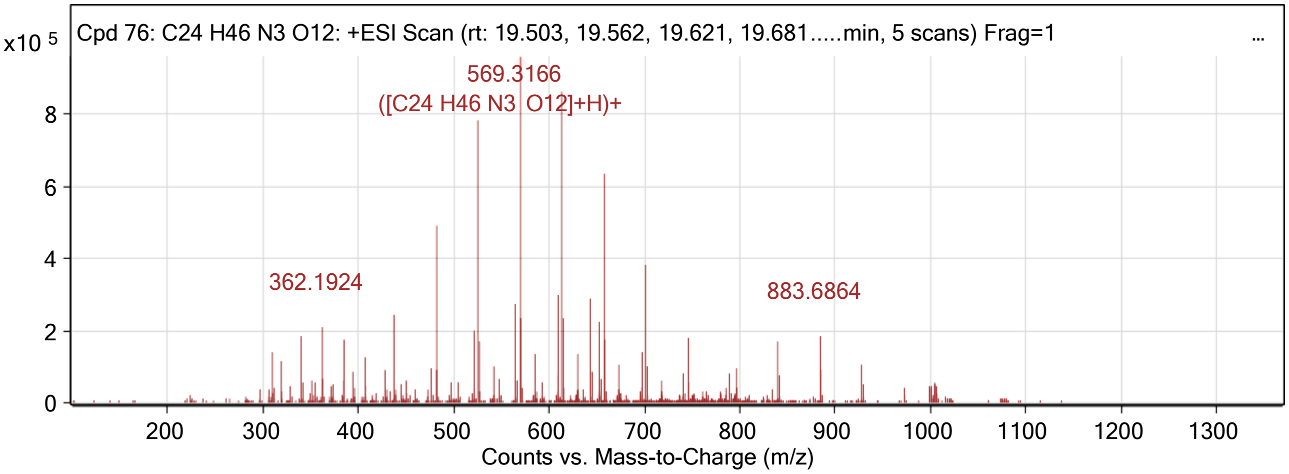

MS Zoomed Spectrum

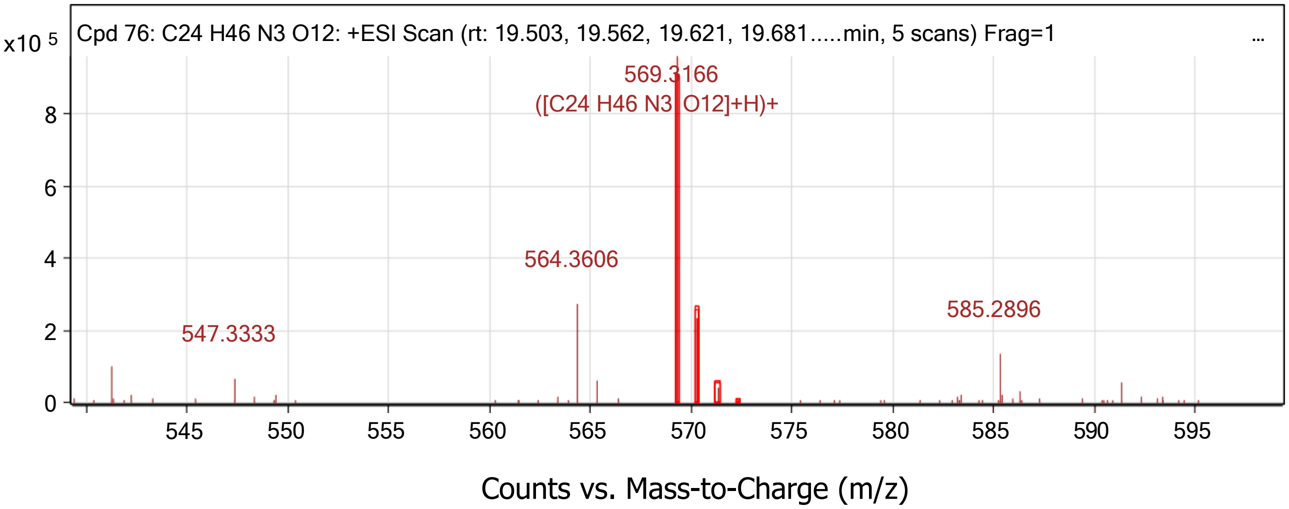

MS Spectrum Peak List

m/z	Calc *m/z*	Diff(ppm)	z	Abund	Formula	Ion
481.2634			1	490302.56		
525.2903			1	782116.38		
569.3166	569.3154	-2.02	1	960951.88	C24 H46 N3 O12	(M+H)+
570.3193	570.3186	-1.16	1	231245.83	C24 H46 N3 O12	(M+H)+
571.3214	571.3209	-0.73	1	38435.08	C24 H46 N3 O12	(M+H)+
572.3244	572.3235	-1.51	1	5164.91	C24 H46 N3 O12	(M+H)+
608.3871			1	297885.19		
613.3431			1	861766.88		
657.3691			1	635402.88		

MSMS Spectrum

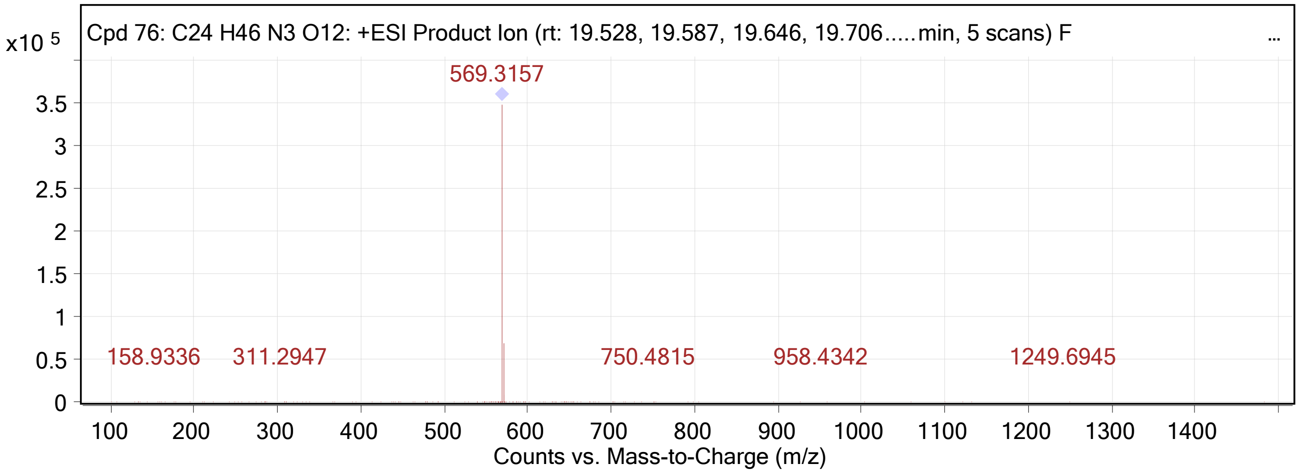

MS/MS Spectrum Peak List

m/z	*Calc m/z*	Diff(ppm)	z	Abund
133.0867	133.0859	-5.49		49.59
177.1112	177.1108	-2.08		18.38
195.1219	195.1214	-2.68		30.79
241.136	241.1394	13.97		13.8
274.2152	274.2139	-4.82		15.99
284.2664	284.2696	11.48		15.2
309.2761	309.2775	4.45		14.02
311.2947	311.2945	-0.9		62.71
329.2271	329.2309	11.63		28.66
569.3157	569.3154	-0.53	1	348678.03

Compound Label	*m/z*	RT	Algorithm	Mass
Cpd 77: C28 H52 O14	613.3429	19.841	Auto MS/MS	612.3355

Compound Chromatograms

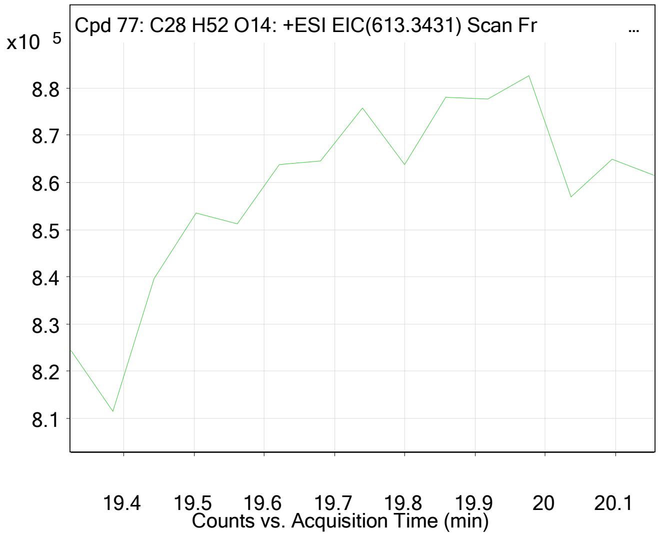

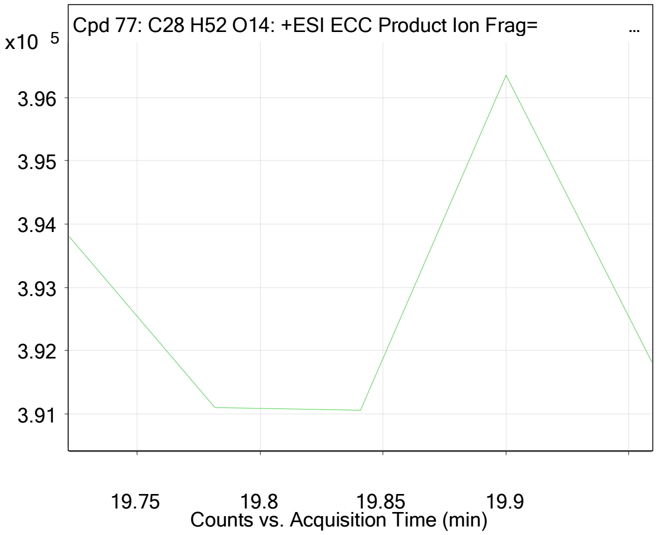

MS Spectrum

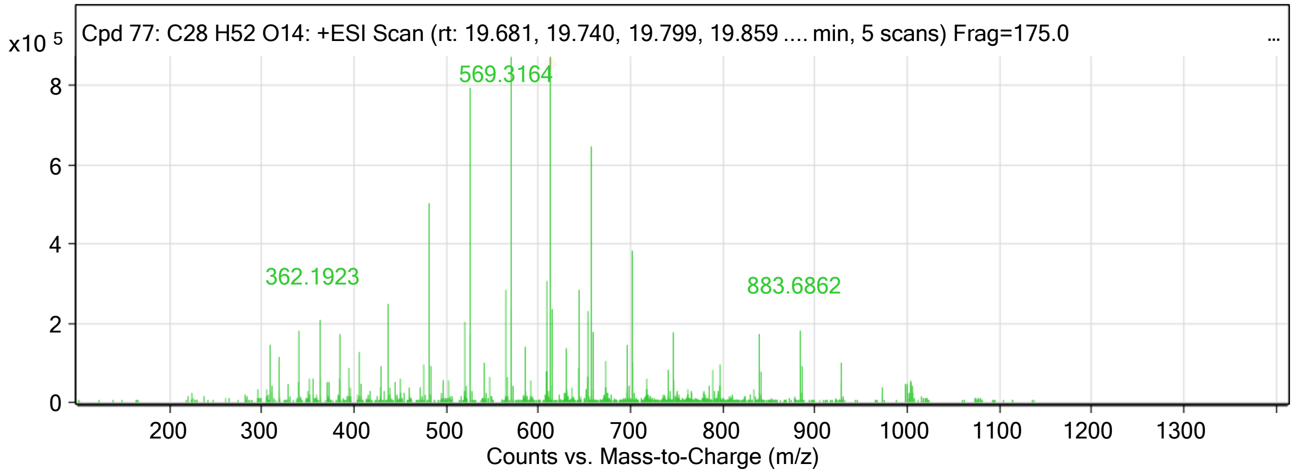

MS Zoomed Spectrum

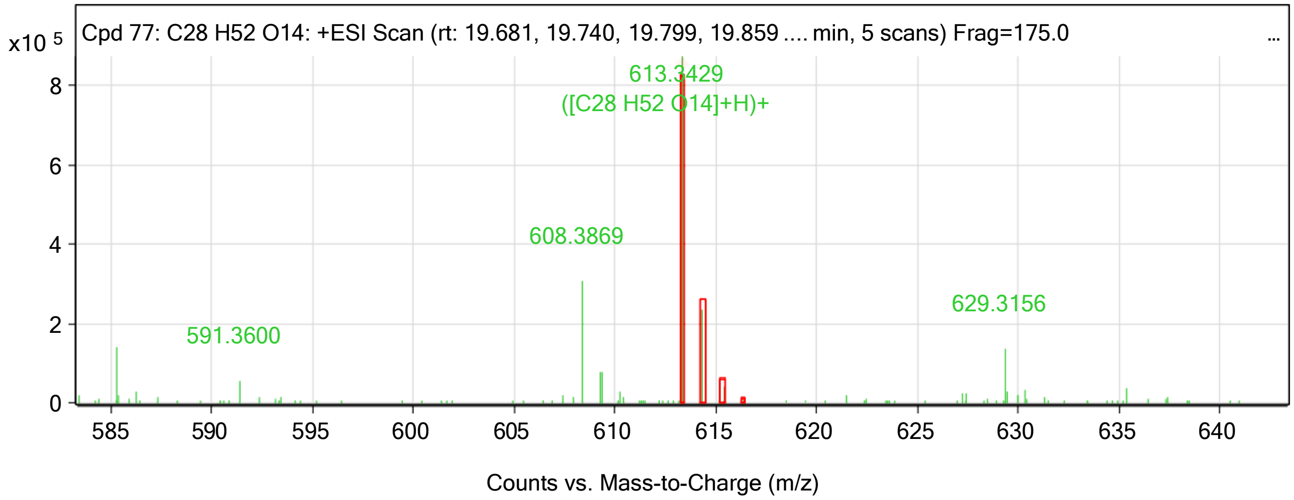

MS Spectrum Peak List

m/z	Calc *m/z*	Diff(ppm)	z	Abund	Formula	Ion
481.2633			1	498434.34		
525.2902			1	792185.38		
569.3164			1	975695.81		
608.3869			1	306253.81		
613.3429	613.343	0.06	1	871966.63	C28 H52 O14	(M+H)+
614.3455	614.3464	1.42	1	235697.72	C28 H52 O14	(M+H)+
615.3474	615.3489	2.31	1	42572.23	C28 H52 O14	(M+H)+
616.3543	616.3515	-4.42	1	5851.39	C28 H52 O14	(M+H)+
657.369			1	641540.13		
701.3953			1	382727.38		

MSMS Spectrum

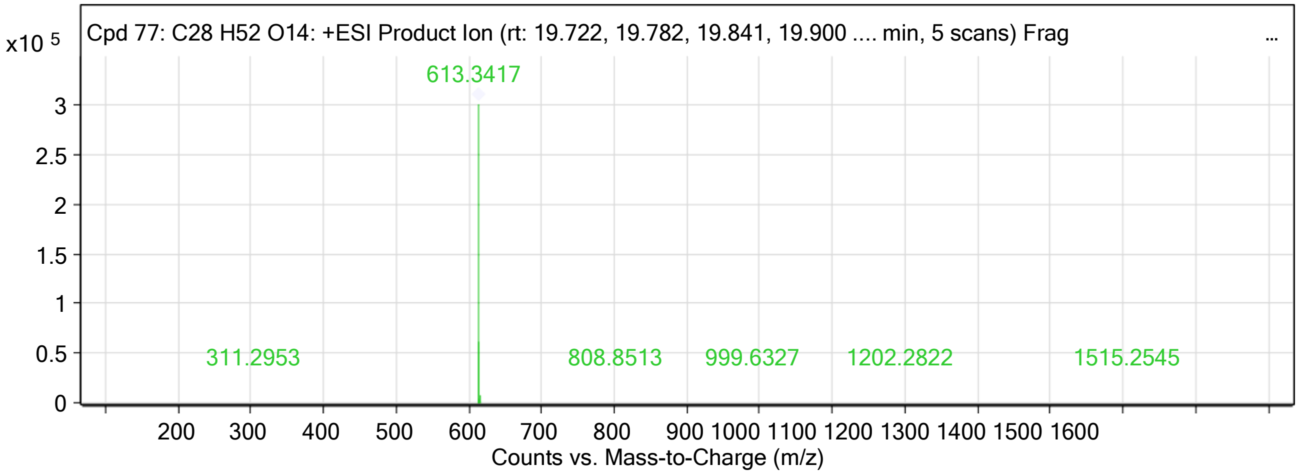

MS/MS Spectrum Peak List

m/z	*Calc m/z*	Diff(ppm)	z	Abund
133.0859	133.0859	0.28		60.58
309.2787	309.2788	0.27		83.32
311.2953	311.2945	-2.8	1	88.89
612.3888			2	2205.02
612.638			2	3024.56
612.889			2	2411.54
613.1418			2	1252.13
613.3417	613.343	2.07	1	301169.94
614.3444			1	61472.11
615.3467			1	7617.62

Compound Label	*m/z*	RT	Algorithm	Mass
Cpd 78: C24 H46 N3 O12	569.3166	19.943	Auto MS/MS	568.3092

Compound Chromatograms

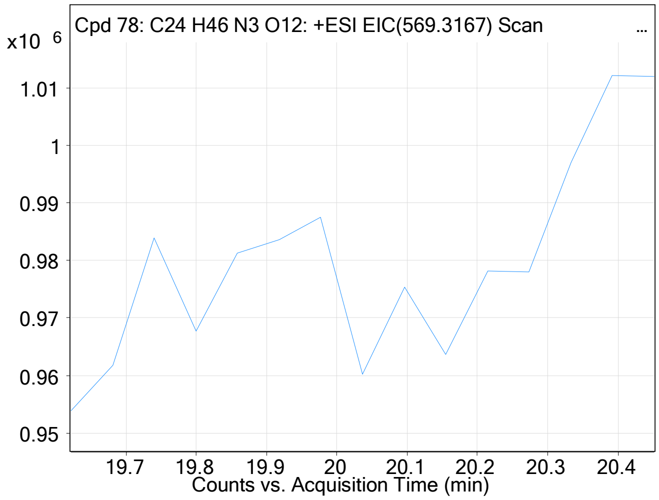

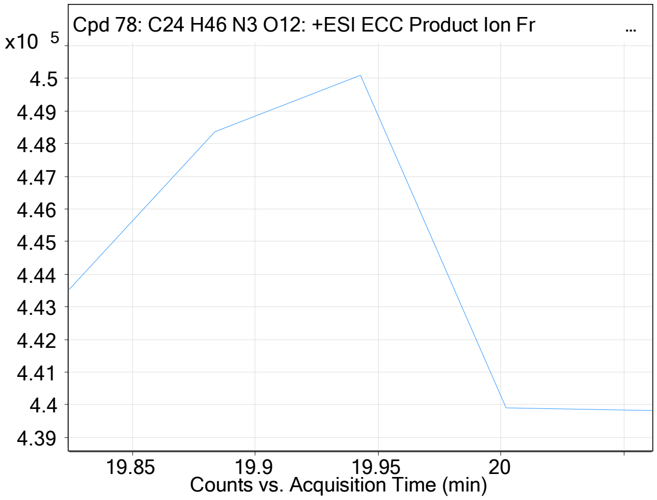

MS Spectrum

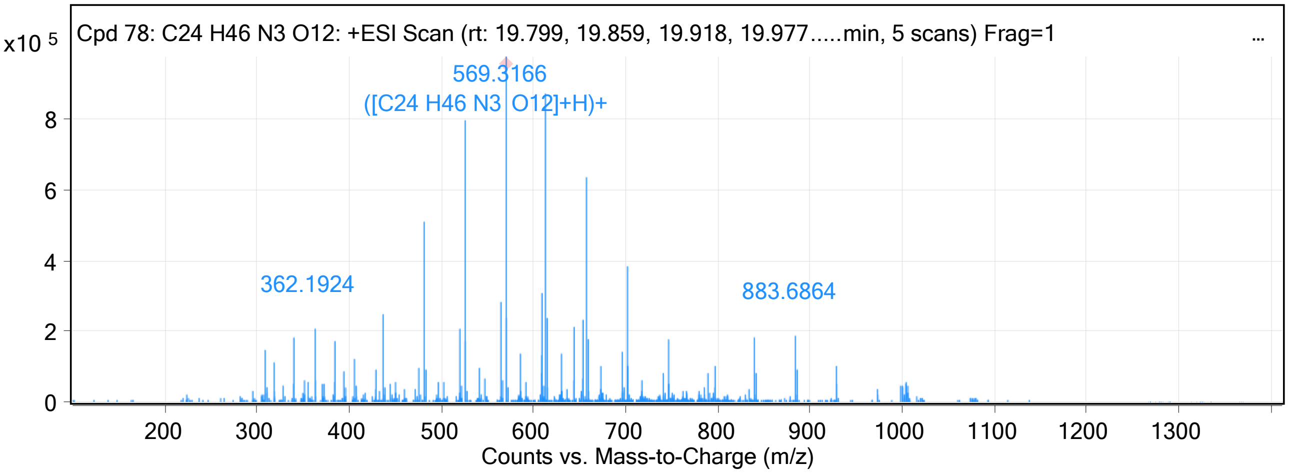

MS Zoomed Spectrum

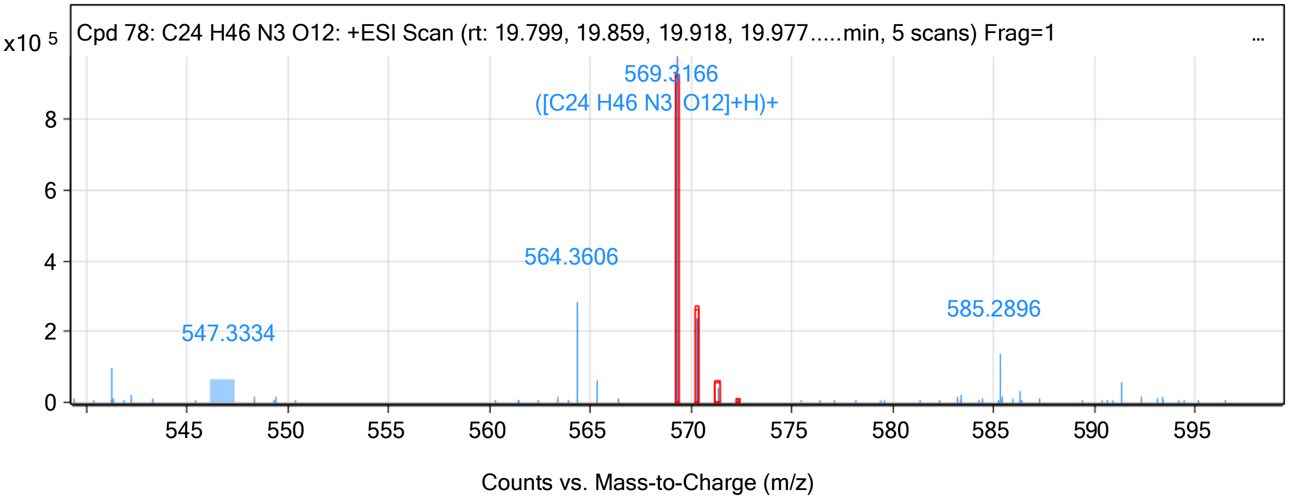

MS Spectrum Peak List

m/z	Calc *m/z*	Diff(ppm)	z	Abund	Formula	Ion
481.2634			1	509704.59		
525.2904			1	797159.13		
569.3166	569.3154	-2.06	1	976116.31	C24 H46 N3 O12	(M+H)+
570.3194	570.3186	-1.31	1	234469	C24 H46 N3 O12	(M+H)+
571.3214	571.3209	-0.72	1	38682.89	C24 H46 N3 O12	(M+H)+
572.3245	572.3235	-1.63	1	5424.57	C24 H46 N3 O12	(M+H)+
608.3871			1	307247.16		
613.3431			1	871787.63		
657.3692			1	635472.63		
701.3954			1	383051.31		

MSMS Spectrum

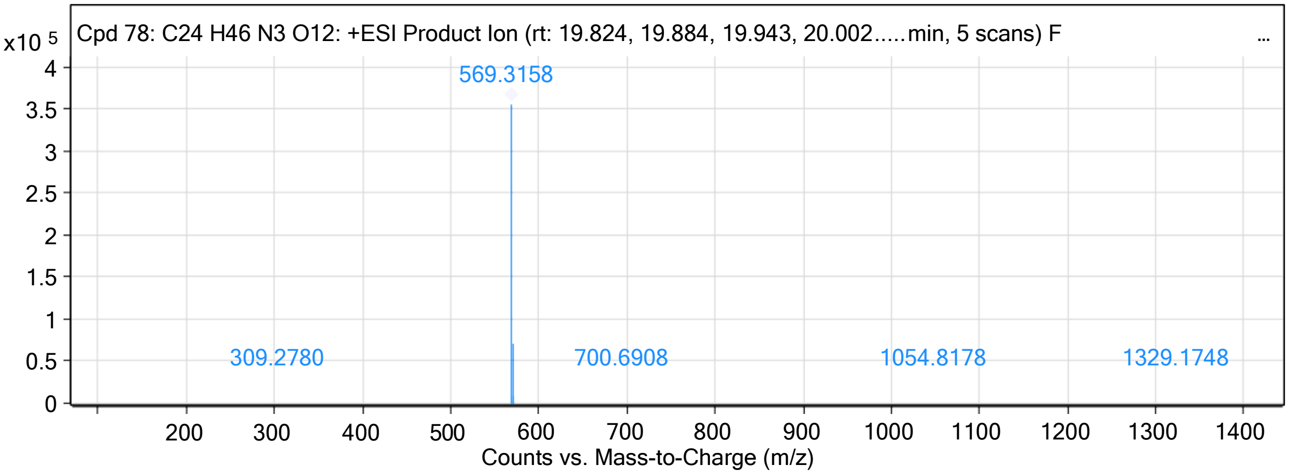

MS/MS Spectrum Peak List

m/z	Calc *m/z*	Diff(ppm)	z	Abund
107.0707	107.0689	-16.11		21.74
133.0856	133.0859	2.61		32.49
151.0977	151.0992	9.98		21.91
195.123	195.1214	-8.27		16.01
283.2639	283.2632	-2.72		17.65
285.2066	285.206	-1.86		35.76
309.278	309.2775	-1.72		47.54
374.2319	374.2299	-5.34		14.46
459.3021	459.3065	9.61		16.83
569.3158	569.3154	-0.68	1	354972.22

Compound Label	*m/z*	RT	Algorithm	Mass
Cpd 79: C28 H52 O14	613.3433	20.137	Auto MS/MS	612.3358

Compound Chromatograms

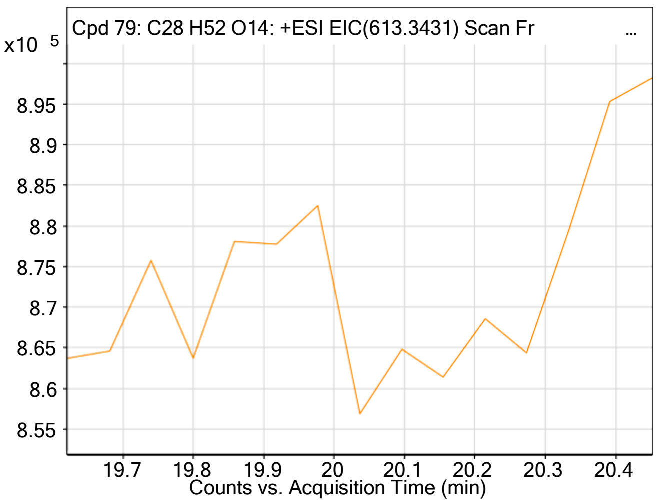

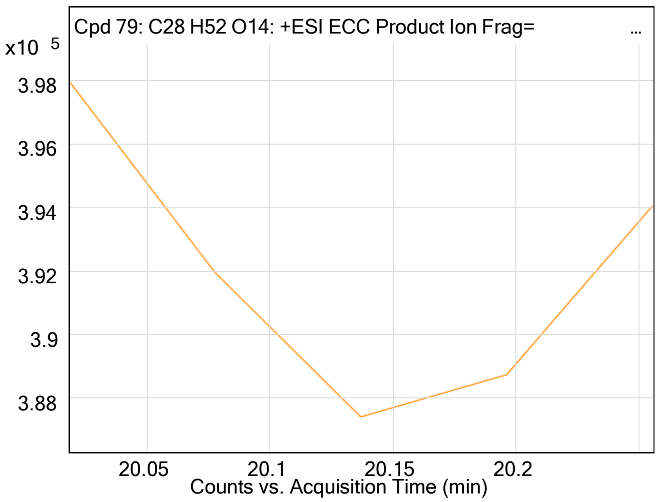

MS Spectrum

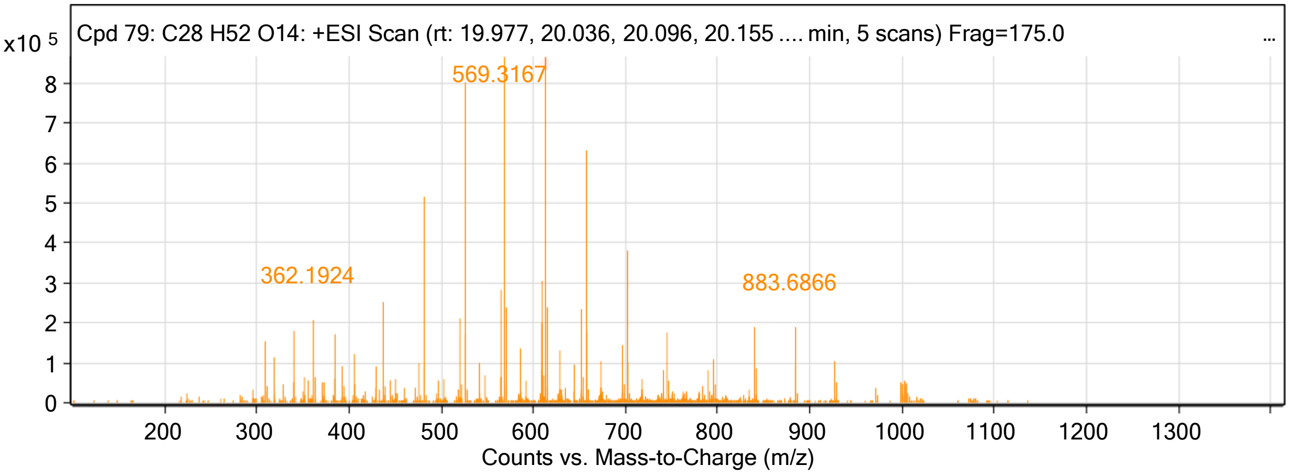

MS Zoomed Spectrum

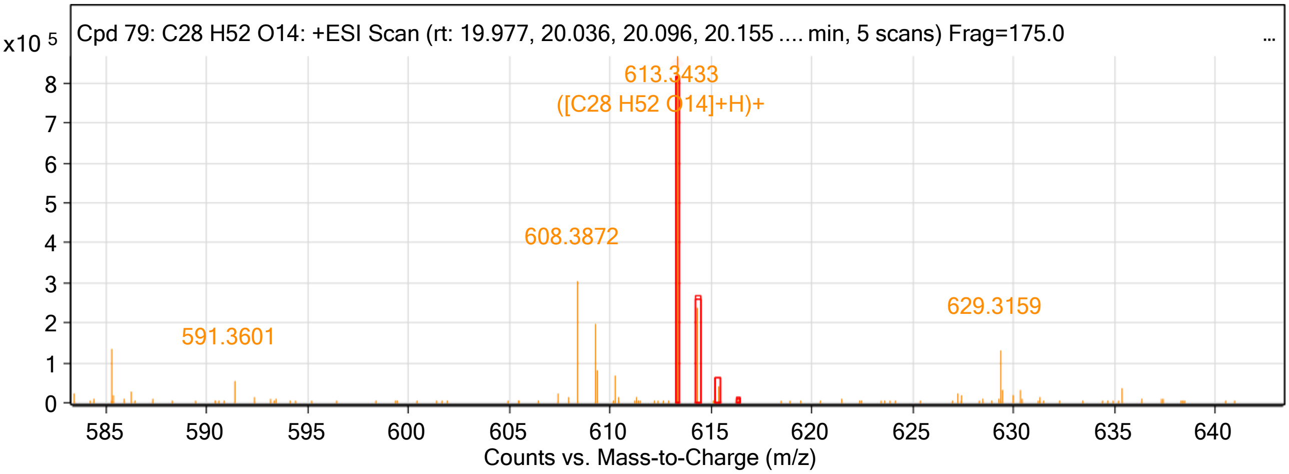

MS Spectrum Peak List

m/z	Calc *m/z*	Diff(ppm)	z	Abund	Formula	Ion
481.2635			1	513905.94		
525.2905			1	800544.13		
569.3167			1	973019.69		
608.3872			1	306251.97		
613.3433	613.343	-0.47	1	866863.5	C28 H52 O14	(M+H)+
614.3459	614.3464	0.86	1	236219.7	C28 H52 O14	(M+H)+
615.3478	615.3489	1.7	1	42276.55	C28 H52 O14	(M+H)+
616.3549	616.3515	-5.5	1	5742.26	C28 H52 O14	(M+H)+
657.3693			1	631557.38		

MSMS Spectrum

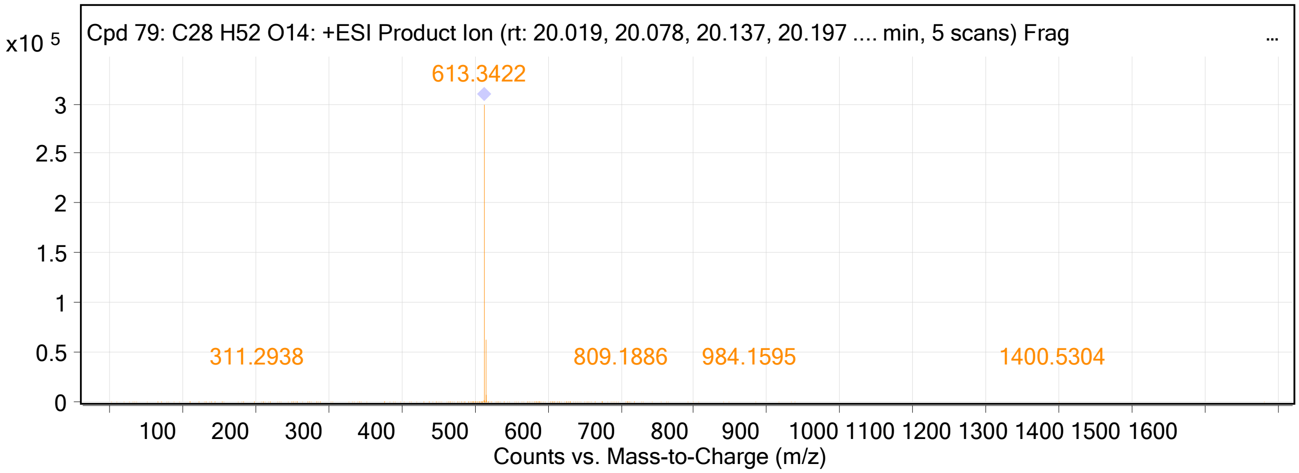

MS/MS Spectrum Peak List

m/z	*Calc m/z*	Diff(ppm)	z	Abund
133.0861	133.0859	-1.71		45.44
309.2785	309.2788	0.99	1	82.46
357.0658	357.0664	1.62		48.27
610.3133	610.3195	10.15	1	40.56
612.3883			2	2292.89
612.6382				2747.36
612.8896			2	2450.2
613.3422	613.343	1.36	1	299439.34
614.3448			1	62780.96
615.3472			1	7390.79

Compound Label	*m/z*	RT	Algorithm	Mass
Cpd 80: C26 H48 O13	569.3166	20.239	Auto MS/MS	568.3092

Compound Chromatograms

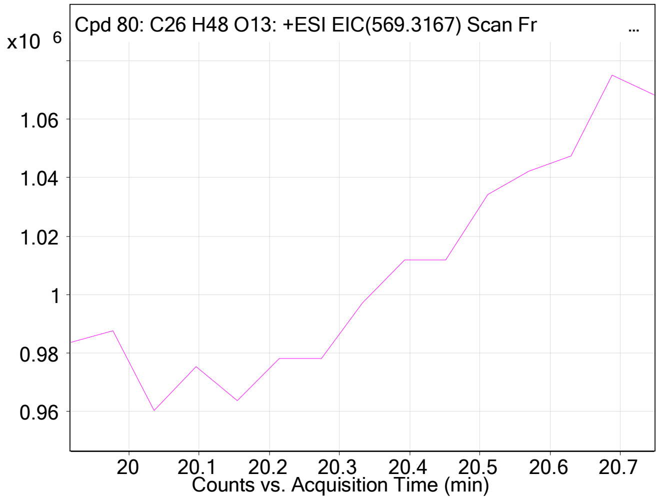

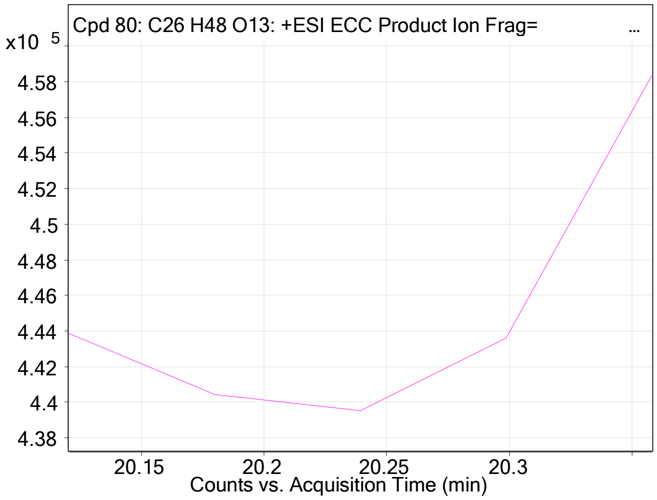

MS Spectrum

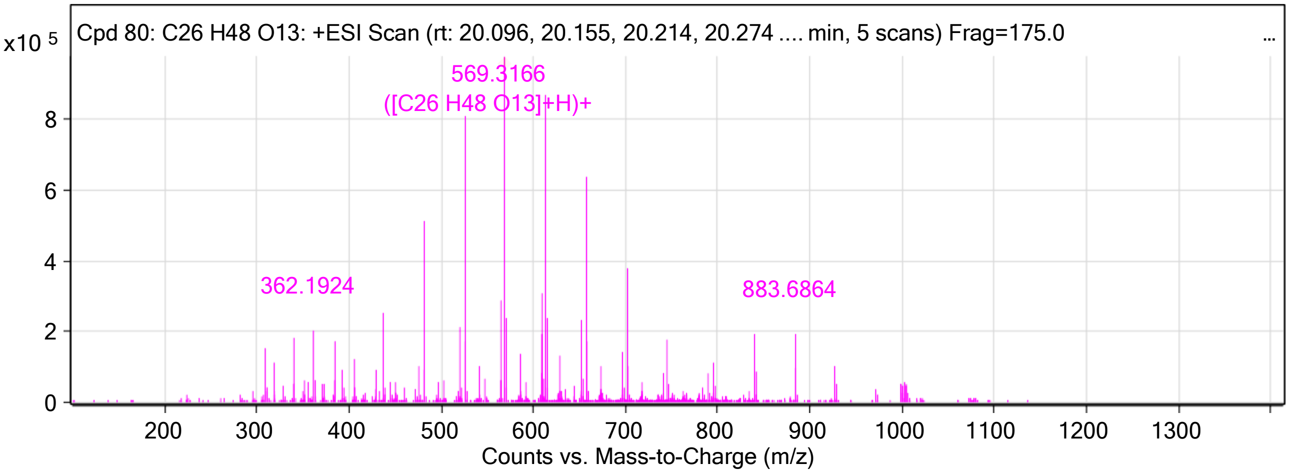

MS Zoomed Spectrum

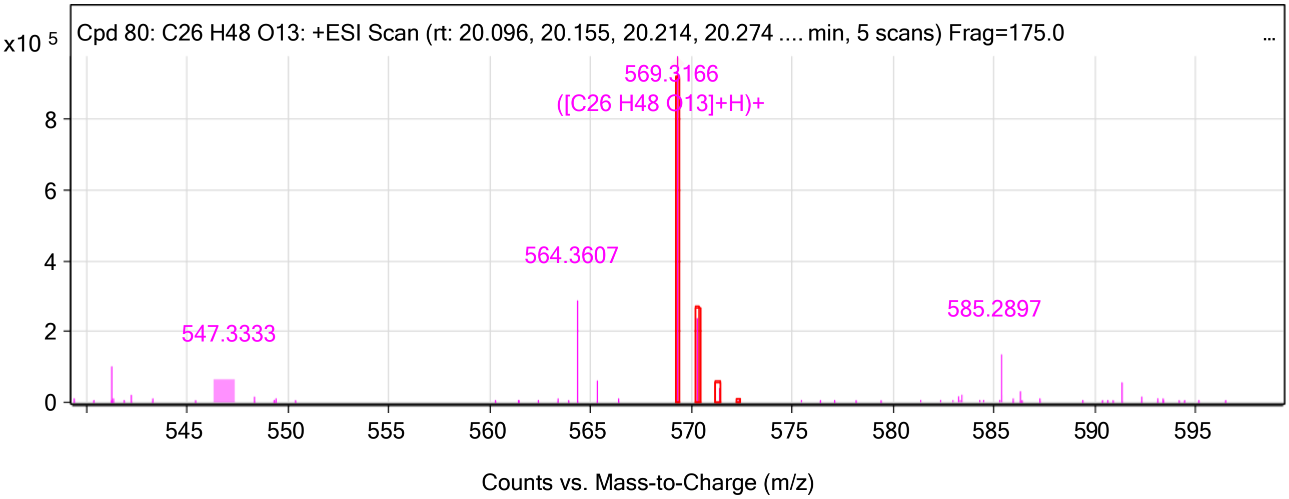

MS Spectrum Peak List

m/z	Calc *m/z*	Diff(ppm)	z	Abund	Formula	Ion
481.2635			1	510358.81		
525.2904			1	805427.81		
569.3166	569.3168	0.25	1	978470.13	C26 H48 O13	(M+H)+
570.3193	570.3202	1.49	1	236060.72	C26 H48 O13	(M+H)+
571.3214	571.3226	2.1	1	38583.9	C26 H48 O13	(M+H)+
572.324	572.3253	2.33	1	5249.86	C26 H48 O13	(M+H)+
608.3871			1	307935.81		
613.3432			1	867755		
657.3692			1	636366.69		
701.3953			1	378011.25		

MSMS Spectrum

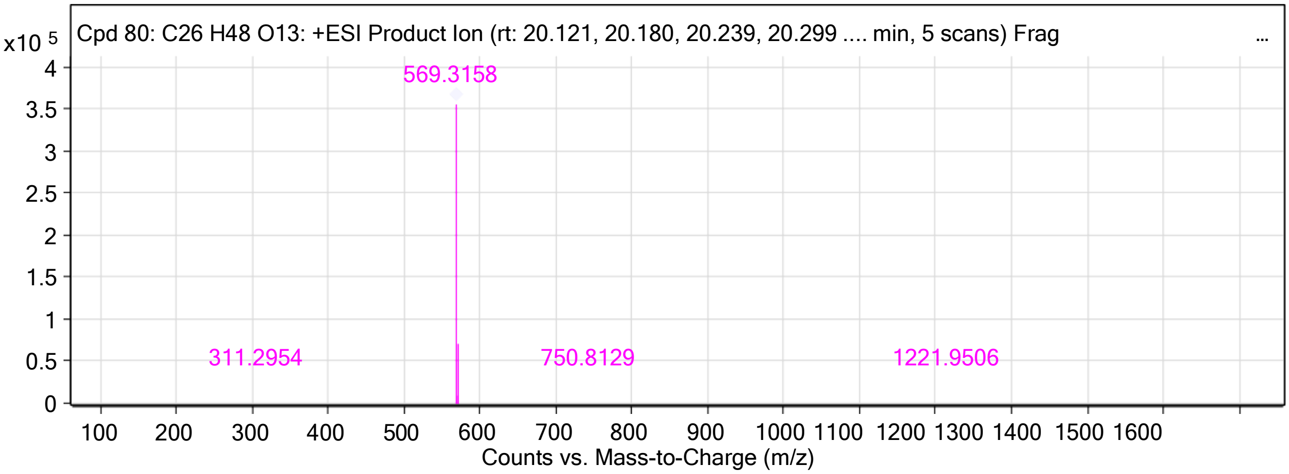

MS/MS Spectrum Peak List

m/z	Calc m/z	Diff(ppm)	z	Abund
117.0908	117.091	1.9		13.8
133.0865	133.0859	-4.49		25.18
165.0856	165.091	32.55		12.52
177.1127	177.1121	-3.25		35.51
221.1397	221.1384	-6.02		21.23
309.2799	309.2788	-3.49		30.91
311.2954	311.2945	-2.92	1	59.34
318.2403	318.2401	-0.78		14.44
569.3158	569.3168	1.69	1	354797.84
570.3183			1	70302.67

Compound Label	m/z	RT	Algorithm	Mass
Cpd 81: C28 H52 O14	613.3431	20.434	Auto MS/MS	612.3356

Compound Chromatograms

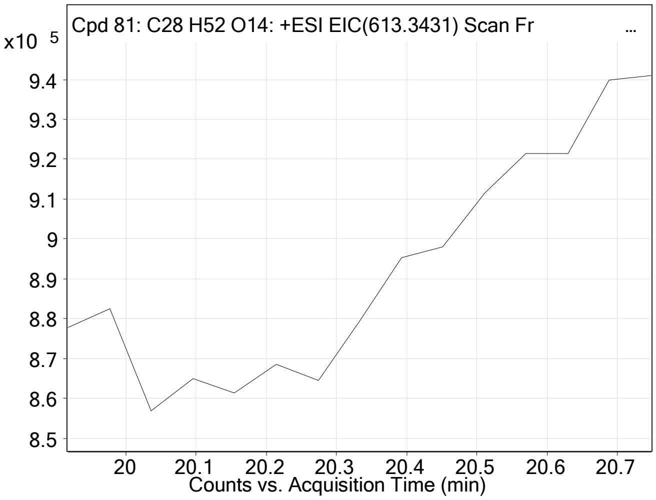

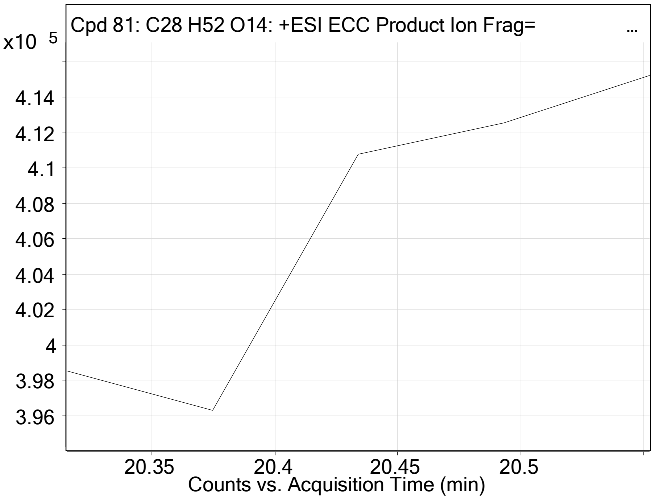

MS Spectrum

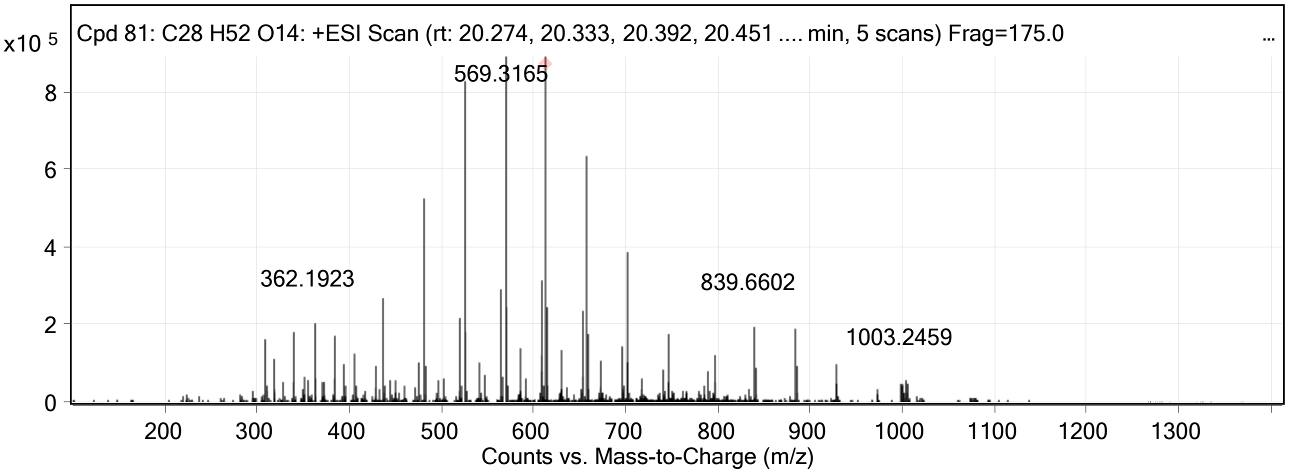

MS Zoomed Spectrum

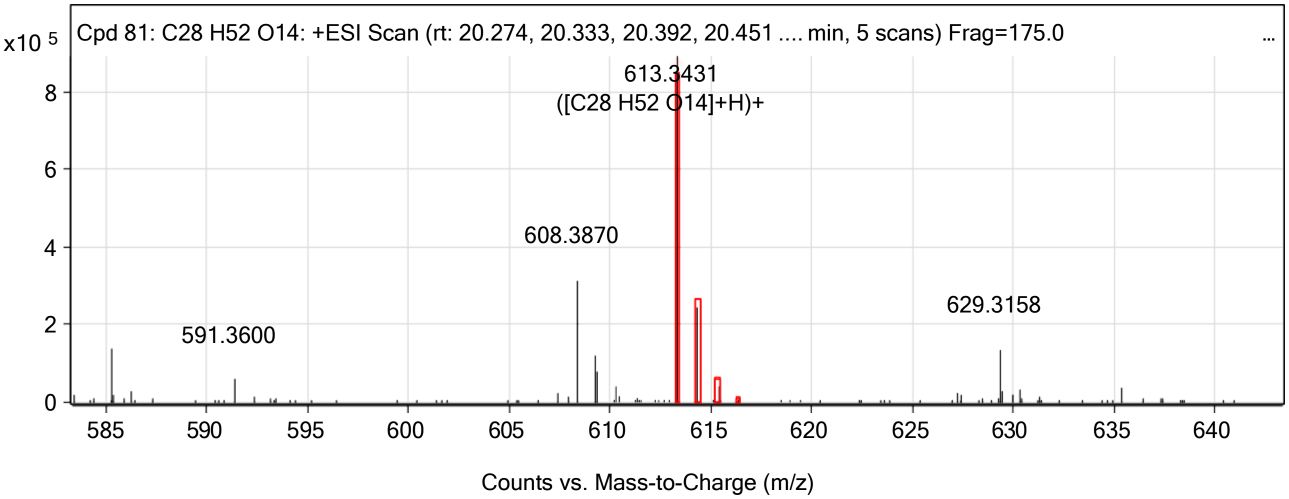

MS Spectrum Peak List

m/z	Calc *m/z*	Diff(ppm)	z	Abund	Formula	Ion
481.2634			1	522086.69		
525.2903			1	827616.75		
569.3165			1	1006700		
608.387			1	311904.03		
613.3431	613.343	-0.15	1	889775.63	C28 H52 O14	(M+H)+
614.3457	614.3464	1.19	1	243484.47	C28 H52 O14	(M+H)+
615.3477	615.3489	1.93	1	43194.82	C28 H52 O14	(M+H)+
616.354	616.3515	-3.93	1	6013.37	C28 H52 O14	(M+H)+
657.3692			1	631542.38		
701.3952			1	383540.03		

MSMS Spectrum

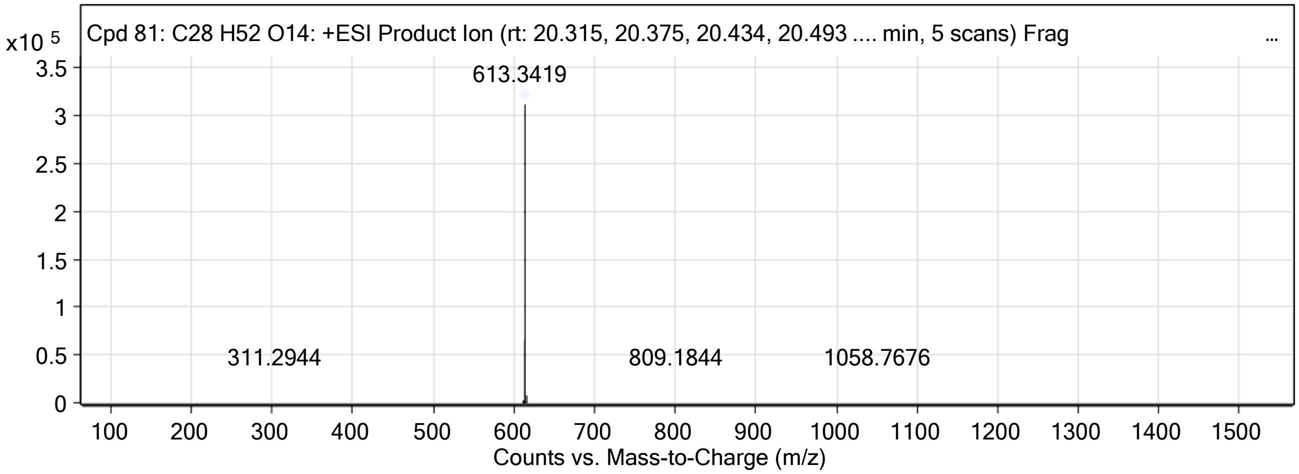

MS/MS Spectrum Peak List

m/z	Calc *m/z*	Diff(ppm)	z	Abund
133.0863	133.0859	-2.89		24.13
296.1515	296.1466	-16.66		29.67
309.2776	309.2788	4.06	1	64.43
612.3887			2	2133.87
612.638			2	2731.26
612.8892			2	2030.21
613.1402			2	1287.45
613.3419	613.343	1.7	1	311985.41
614.3446			1	64535.2
615.3467			1	7810.14

Compound Label	*m/z*	RT	Algorithm	Mass
Cpd 82: C24 H46 N3 O12	569.3165	20.536	Auto MS/MS	568.3091

Compound Chromatograms

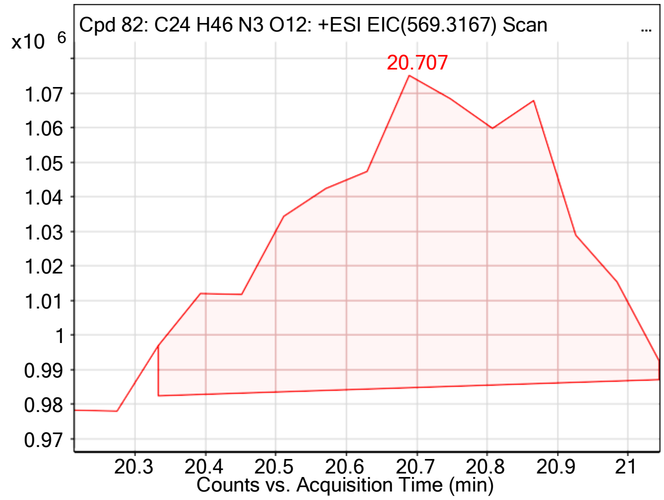

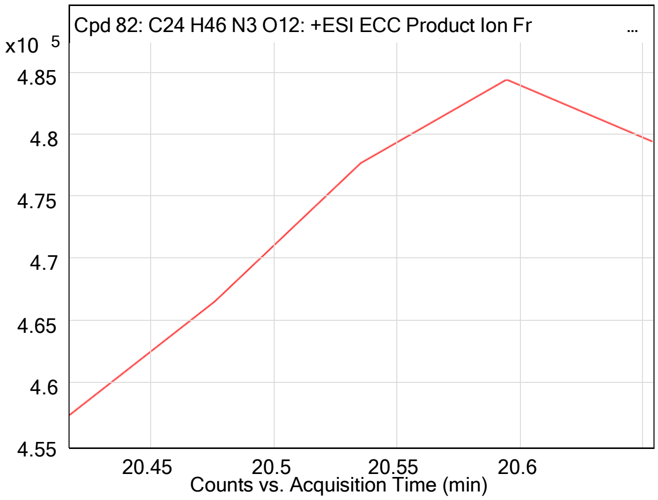

MS Spectrum

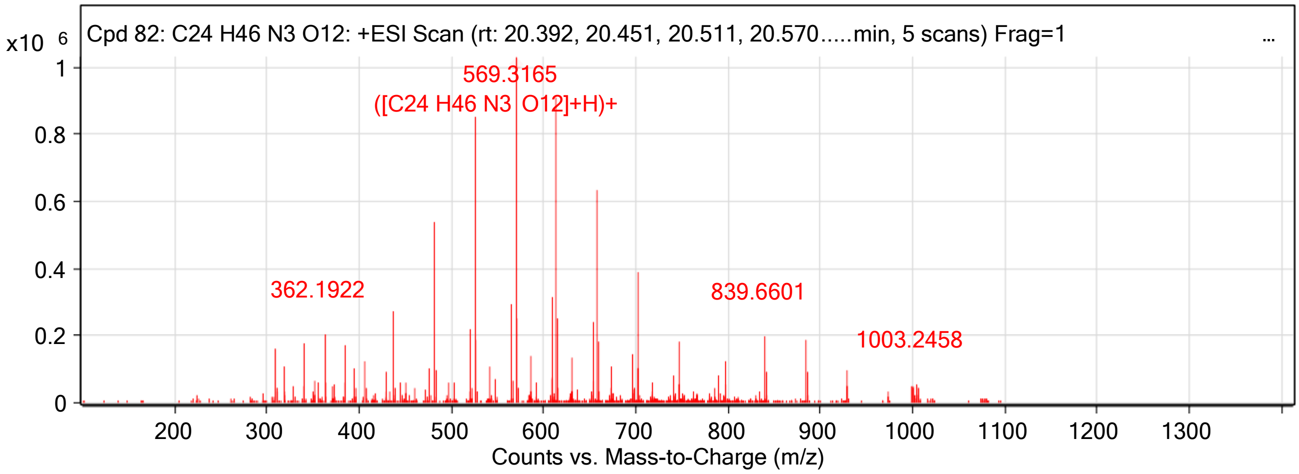

MS Zoomed Spectrum

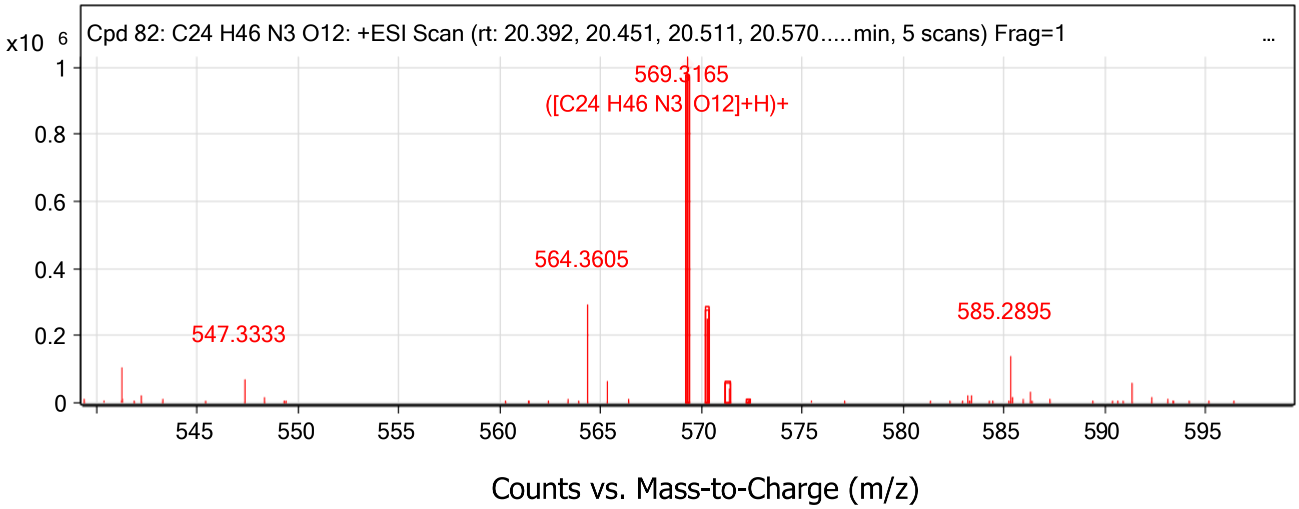

MS Spectrum Peak List

m/z	Calc *m/z*	Diff(ppm)	z	Abund	Formula	Ion
481.2633			1	538703.06		
525.2902			1	849161		
569.3165	569.3154	-1.87	1	1029638.38	C24 H46 N3 O12	(M+H)+
570.3192	570.3186	-1.01	1	248469.63	C24 H46 N3 O12	(M+H)+
571.3212	571.3209	-0.47	1	40797.7	C24 H46 N3 O12	(M+H)+
572.324	572.3235	-0.9	1	5532.4	C24 H46 N3 O12	(M+H)+
608.387			1	314592.16		
613.343			1	909564		
657.3691			1	633909.88		

MSMS Spectrum

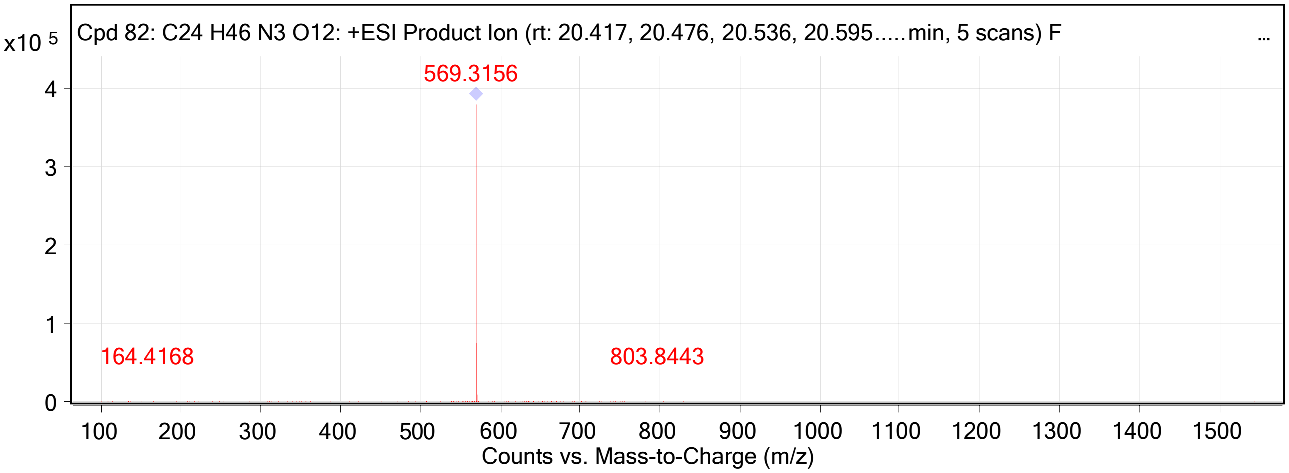

MS/MS Spectrum Peak List

m/z	Calc m/z	Diff(ppm)	z	Abund
133.087	133.0859	-8.36		36.38
221.1369	221.137	0.31		13.5
309.2801	309.2788	-4.07		42.62
311.2948	311.2945	-1.19		50.39
566.2951	566.292	-5.64		19.33
568.3613			2	615.56
568.8614			2	604.71
569.3156	569.3154	-0.38	1	379015.69
570.3182			1	74985.11
571.3204			1	8635.67

Compound Label	m/z	RT	Algorithm	Mass
Cpd 83: C28 H52 O14	613.3431	20.73	Auto MS/MS	612.3356

Compound Chromatograms

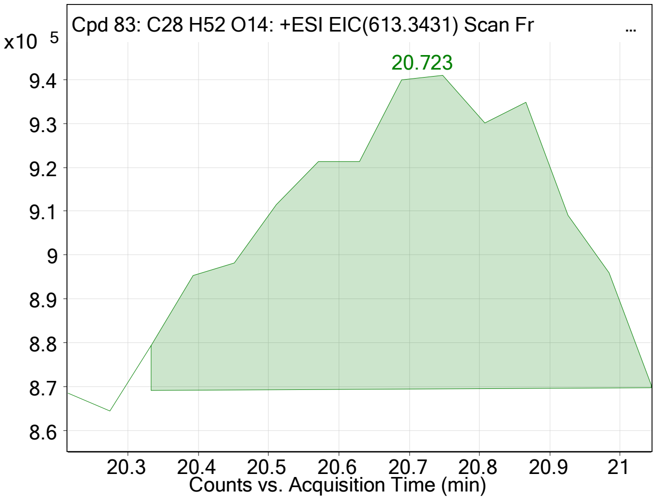

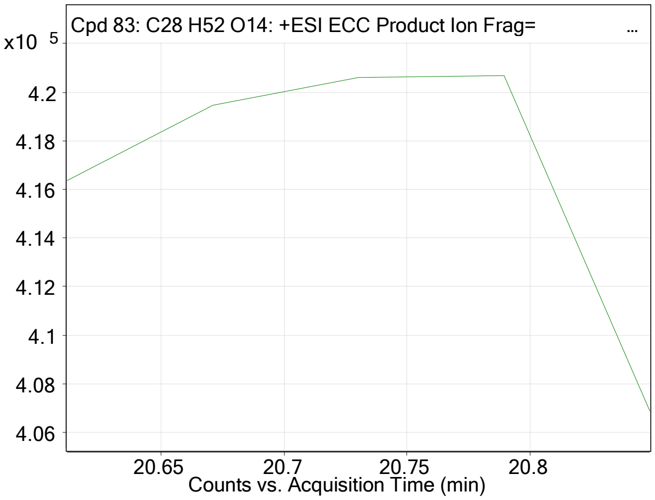

MS Spectrum

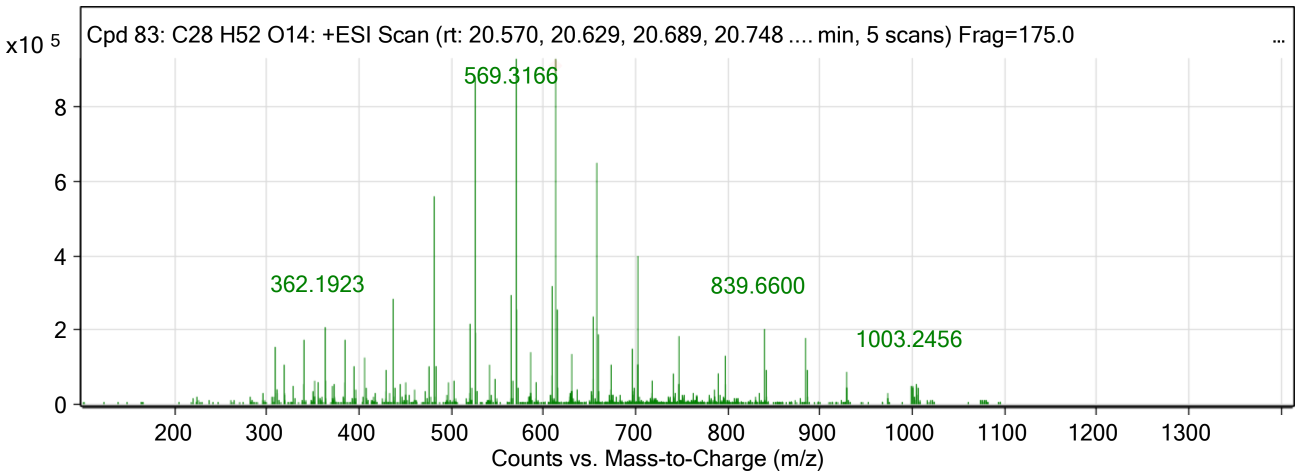

MS Zoomed Spectrum

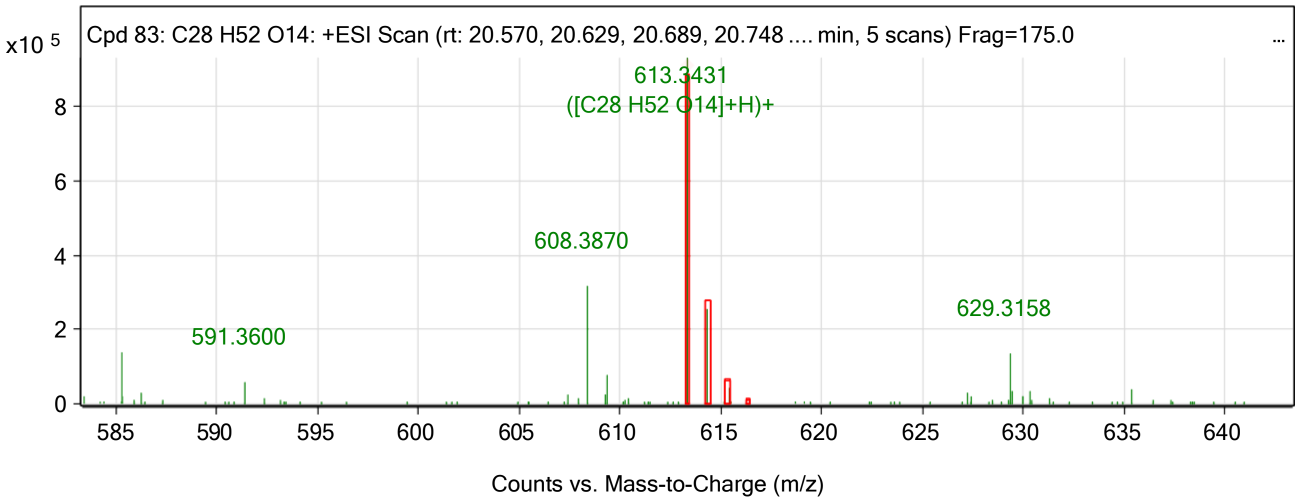

MS Spectrum Peak List

m/z	Calc *m/z*	Diff(ppm)	z	Abund	Formula	Ion
481.2634			1	557861.13		
525.2903			1	878224.63		
569.3166			1	1058599.75		
608.387			1	317239.75		
613.3431	613.343	-0.17	1	930779	C28 H52 O14	(M+H)+
614.3457	614.3464	1.21	1	254644.34	C28 H52 O14	(M+H)+
615.3476	615.3489	2.09	1	45512.98	C28 H52 O14	(M+H)+
616.3537	616.3515	-3.42	1	6275.49	C28 H52 O14	(M+H)+
657.3692			1	648769.31		
701.3952			1	397825.75		

MSMS Spectrum

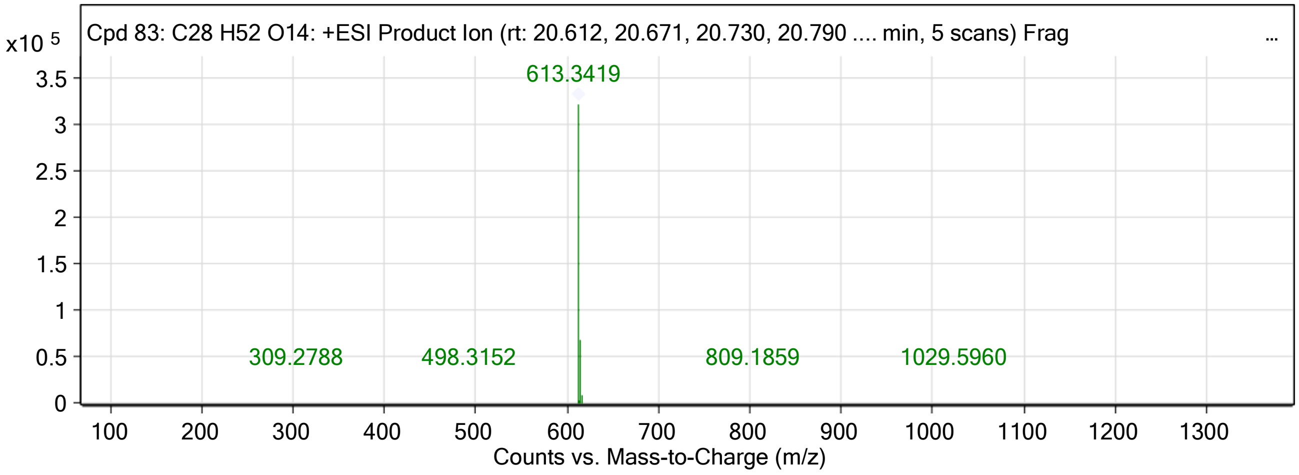

MS/MS Spectrum Peak List

m/z	*Calc m/z*	Diff(ppm)	z	Abund
133.0861	133.0859	-1.69	1	52.8
177.1119	177.1121	1.35		36.9
233.175	233.1747	-1.22		28.16
309.2788	309.2788	-0.08	1	79.65
610.3182	305.1595	-499999.35	2	35.14
611.3278	305.6634	-500000.79	2	26.92
612.6384			2	2383.24
613.3419	613.343	1.71	1	321444.41
614.3447			1	66908.69
615.3468			1	8240.13

Compound Label	*m/z*	RT	Algorithm	Mass
Cpd 84: C26 H48 O13	569.3167	20.832	Auto MS/MS	568.3092

Compound Chromatograms

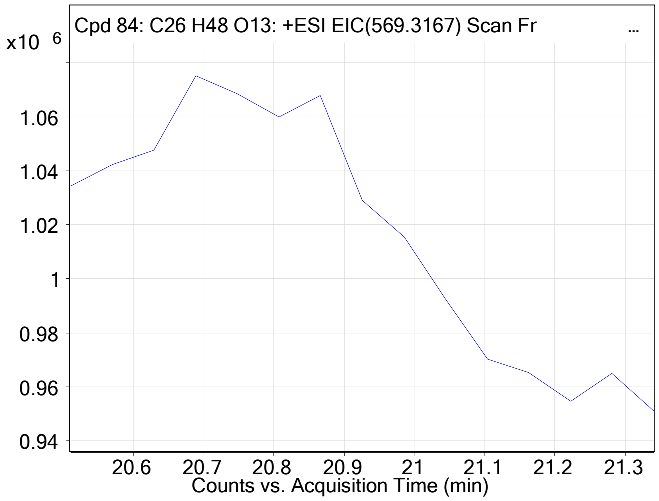

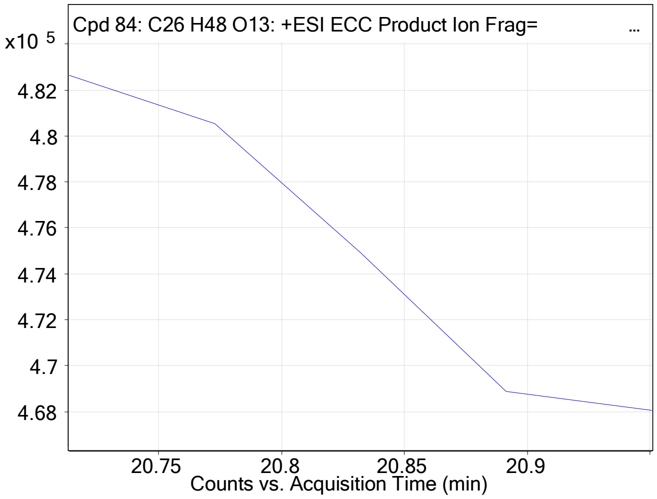

MS Spectrum

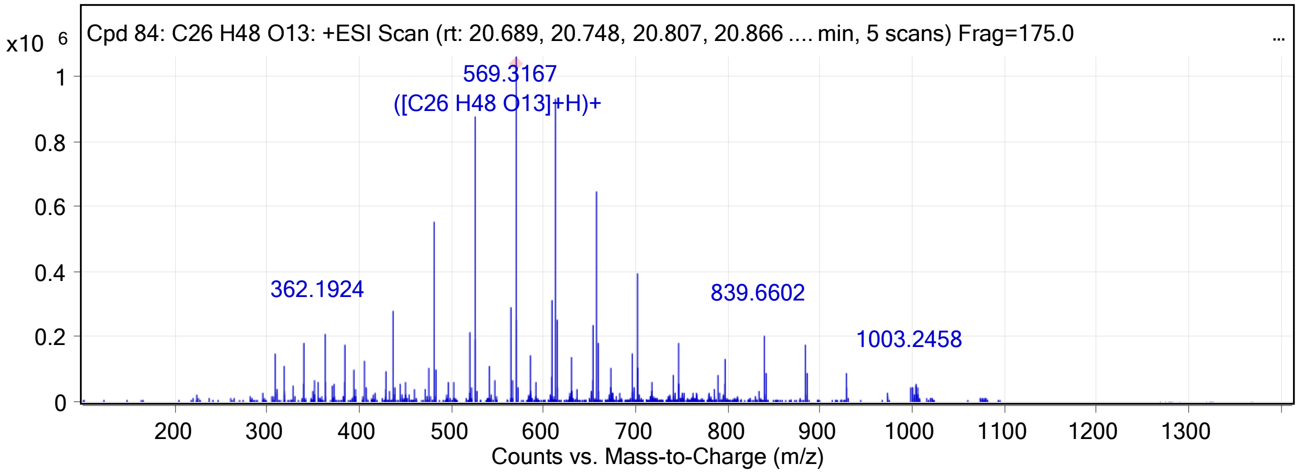

MS Zoomed Spectrum

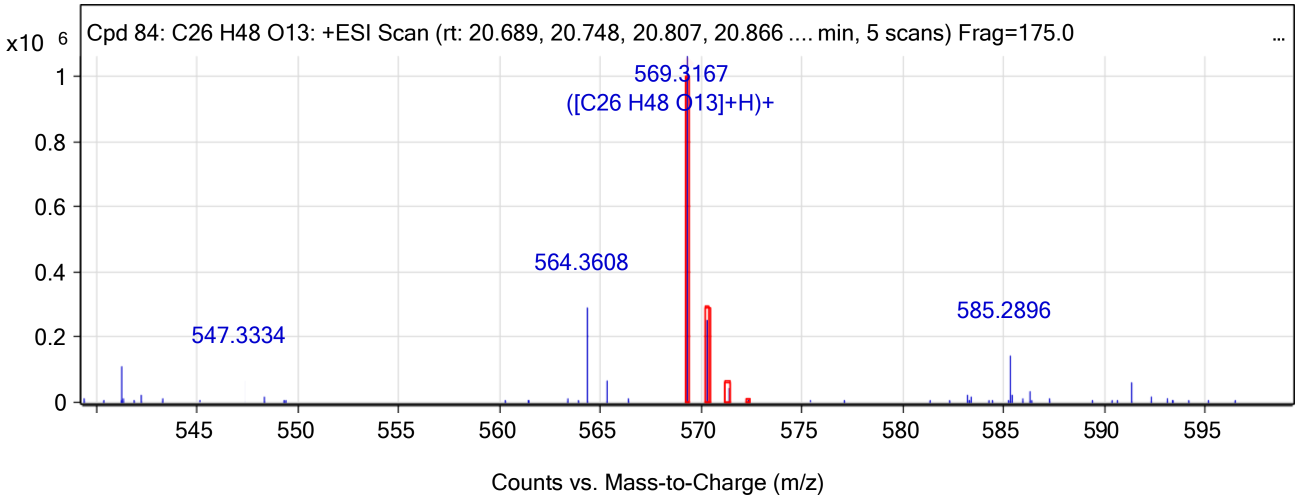

MS Spectrum Peak List

m/z	Calc *m/z*	Diff(ppm)	z	Abund	Formula	Ion
481.2635			1	553781.75		
525.2904			1	874875.31		
569.3167	569.3168	0.13	1	1060016	C26 H48 O13	(M+H)+
570.3194	570.3202	1.43	1	253619.45	C26 H48 O13	(M+H)+
571.3214	571.3226	2.08	1	42225.76	C26 H48 O13	(M+H)+
572.3238	572.3253	2.58	1	5681.76	C26 H48 O13	(M+H)+
608.3872			1	312799.56		
613.3432			1	931009.88		
657.3693			1	646027.63		
701.3953			1	396088.44		

MSMS Spectrum

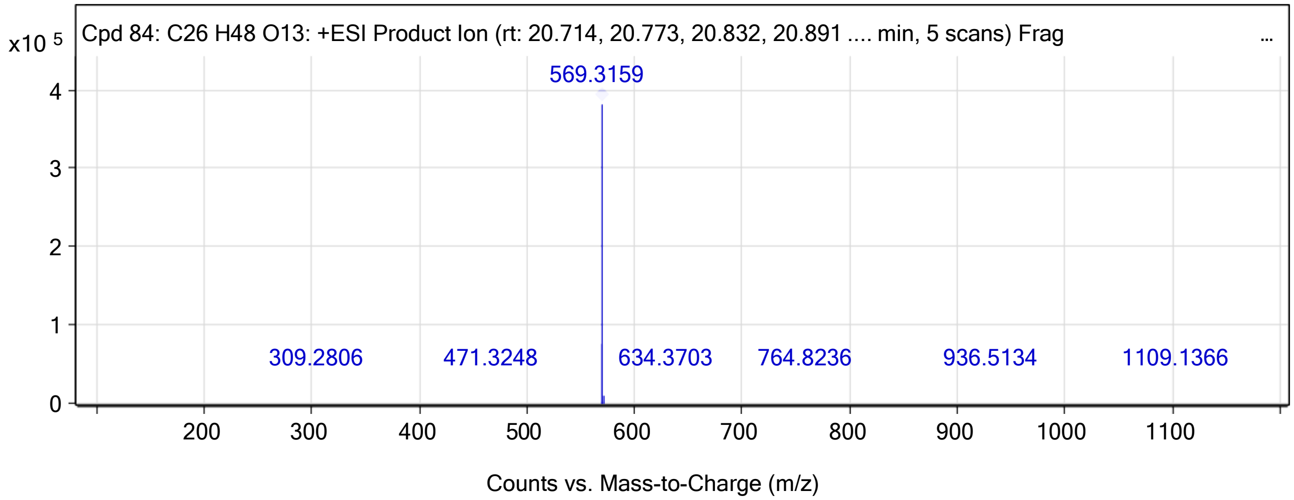

MS/MS Spectrum Peak List

m/z	Calc *m/z*	Diff(ppm)	z	Abund
146.0956	146.0937	-13.02		13.88
210.1136	210.1098	-18.04		18.66
265.2493	265.2526	12.42		13.34
309.2806	309.2788	-5.81		37.72
471.3248	471.3316	14.53		17.02
538.2903	269.1489	-499992.98	2	20.78
568.6122			2	793.97
569.3159	569.3168	1.53	1	381023.56
570.3185			1	74487.7
571.3206			1	8772.77

Compound Label	*m/z*	RT	Algorithm	Mass
Cpd 85: C28 H52 O14	613.3432	21.027	Auto MS/MS	612.3357

Compound Chromatograms

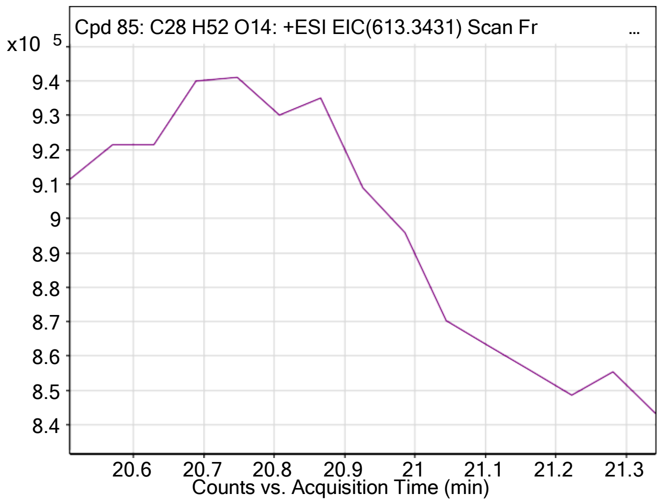

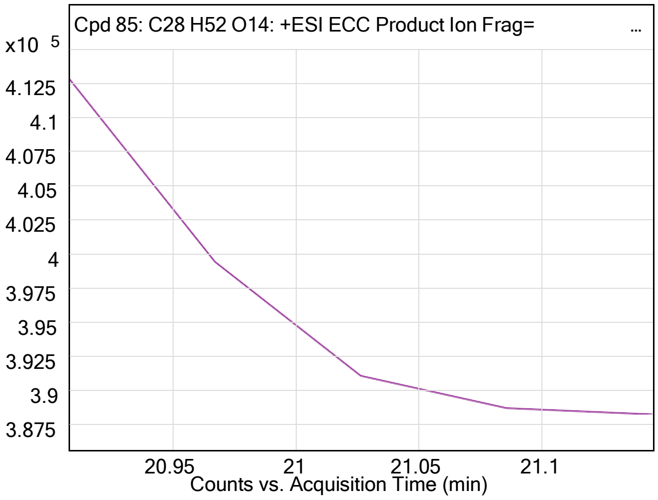

MS Spectrum

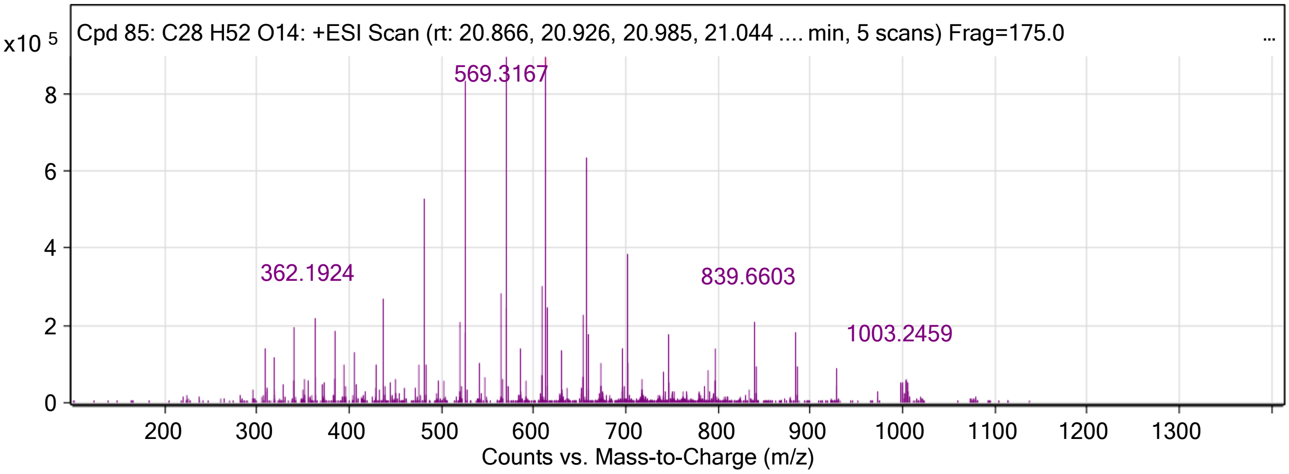

MS Zoomed Spectrum

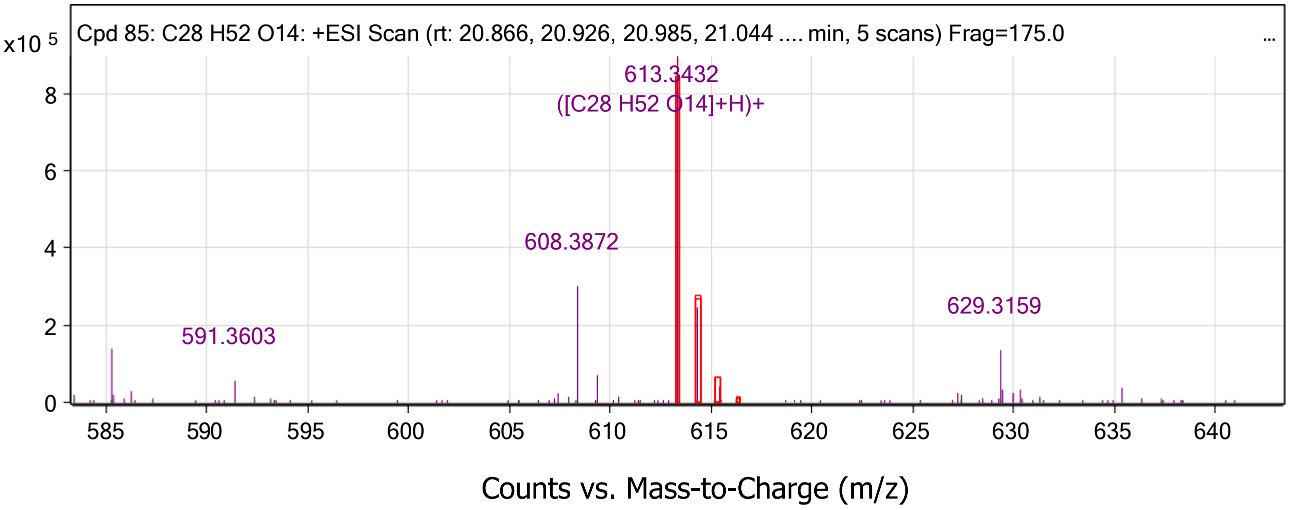

MS Spectrum Peak List

m/z	Calc *m/z*	Diff(ppm)	z	Abund	Formula	Ion
481.2635			1	527171.88		
525.2904			1	832653.13		
569.3167			1	1015013.69		
608.3872			1	301009.06		
613.3432	613.343	-0.37	1	894690.69	C28 H52 O14	(M+H)+
614.3458	614.3464	1.03	1	243687.55	C28 H52 O14	(M+H)+
615.3478	615.3489	1.8	1	43336.34	C28 H52 O14	(M+H)+
616.354	616.3515	-3.96	1	6045.07	C28 H52 O14	(M+H)+

MSMS Spectrum

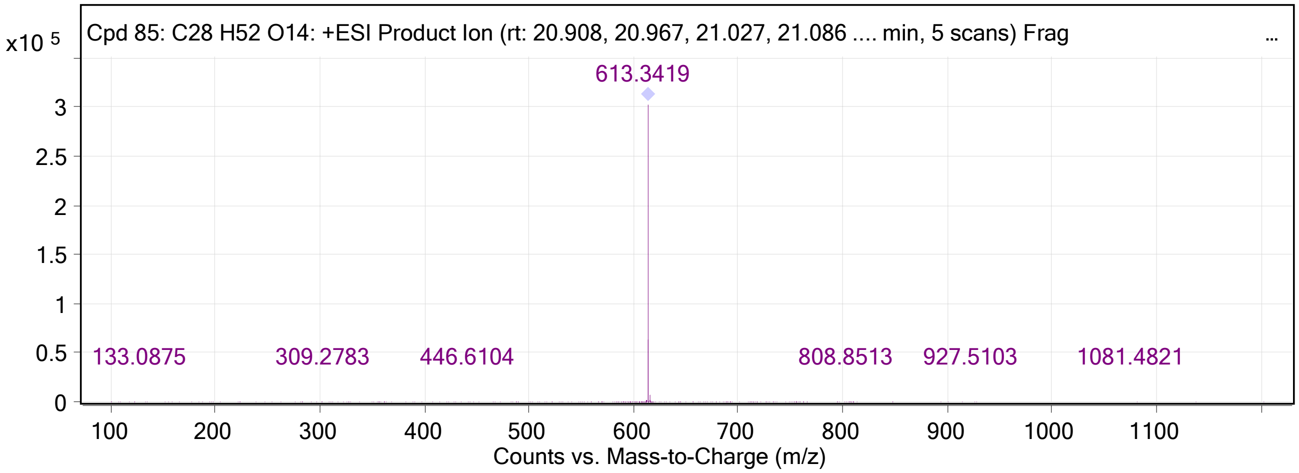

MS/MS Spectrum Peak List

m/z	*Calc m/z*	Diff(ppm)	z	Abund
133.0875	133.0859	-11.78		33.99
309.2783	309.2788	1.73	1	49.36
612.3874			2	1944.05
612.6384			2	2364.05
612.8896			2	2199.9
613.1403			2	1087.17
613.3419	613.343	1.7	1	302760.25
614.3446			1	64078.77
615.3469			1	7836.38
615.4157			2	1133.17

Compound Label	*m/z*	RT	Algorithm	Mass
Cpd 86: C24 H46 N3 O12	569.3166	21.129	Auto MS/MS	568.3092

Compound Chromatograms

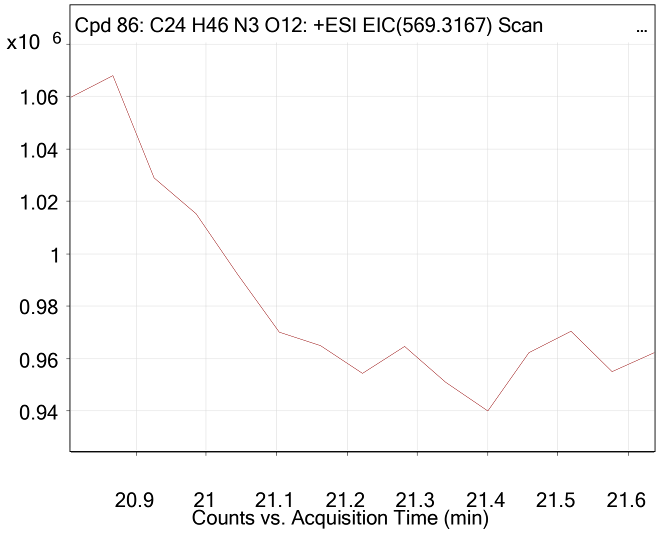

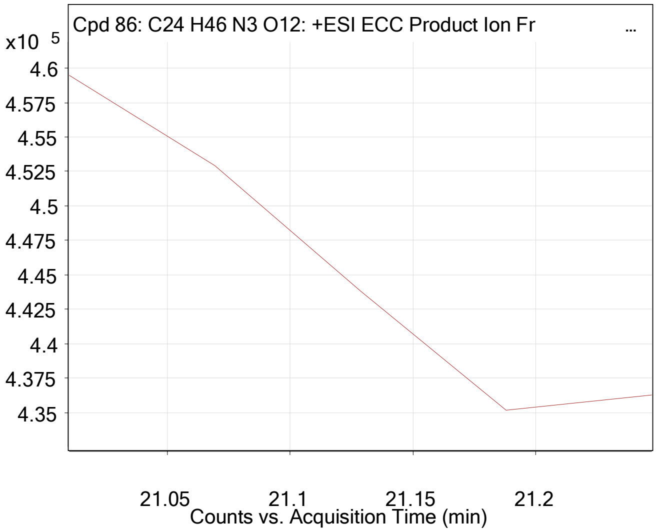

MS Spectrum

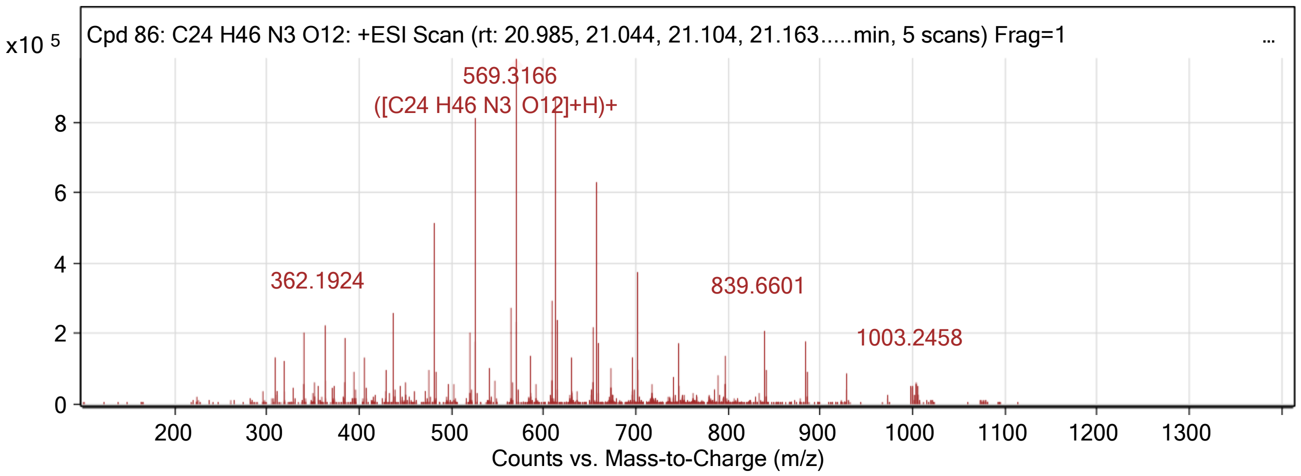

MS Zoomed Spectrum

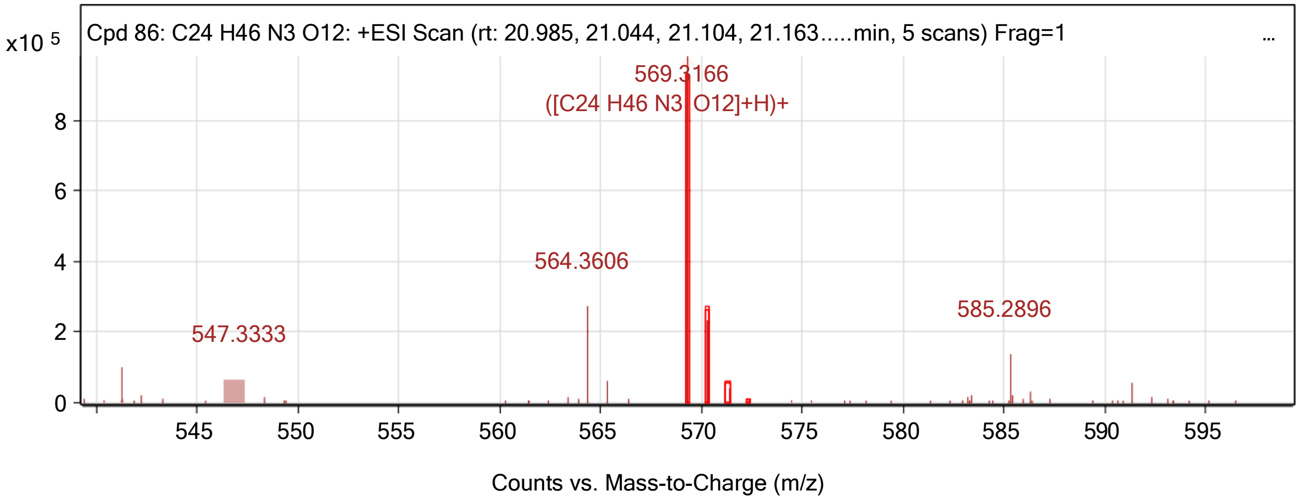

MS Spectrum Peak List

m/z	Calc *m/z*	Diff(ppm)	z	Abund	Formula	Ion
481.2634			1	512403.81		
525.2903			1	807483.13		
569.3166	569.3154	-2.03	1	979597.38	C24 H46 N3 O12	(M+H)+
570.3193	570.3186	-1.14	1	235036.47	C24 H46 N3 O12	(M+H)+
571.3213	571.3209	-0.64	1	38795.34	C24 H46 N3 O12	(M+H)+
572.3243	572.3235	-1.43	1	5420.92	C24 H46 N3 O12	(M+H)+
608.387			1	292471.78		
613.3431			1	866851.38		
657.3691			1	624298.75		
701.3952			1	373002.5		

MSMS Spectrum

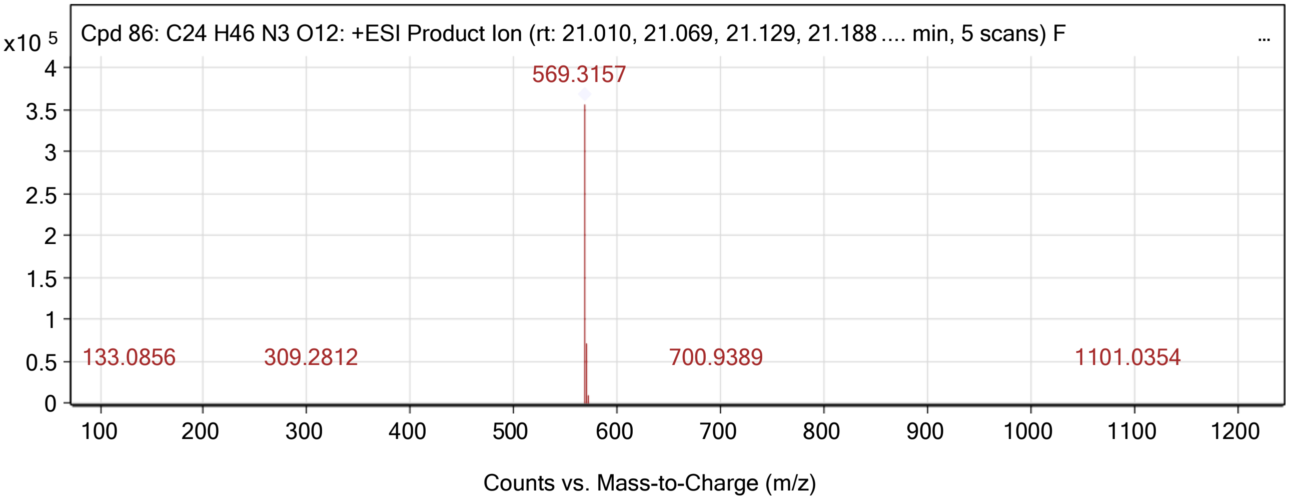

MS/MS Spectrum Peak List

m/z	*Calc m/z*	Diff(ppm)	z	Abund
107.0709	107.0689	-18.3		19.09
133.0856	133.0859	2.5		51.15
153.002	153.0016	-2.32		19.47
195.1221	195.1214	-3.69		29.77
263.1894	131.5937	-500003.8	2	27.15
283.1766	283.1778	4.33		18.3
309.2812	309.2788	-7.7		40.12
311.2924	311.2931	2.28		35.35
515.3063	257.6528	-500000.61	2	17.81
569.3157	569.3154	-0.5	1	356144.59

Compound Label	*m/z*	RT	Algorithm	Mass
Cpd 87: C28 H52 O14	613.3433	21.323	Auto MS/MS	612.3358

Compound Chromatograms

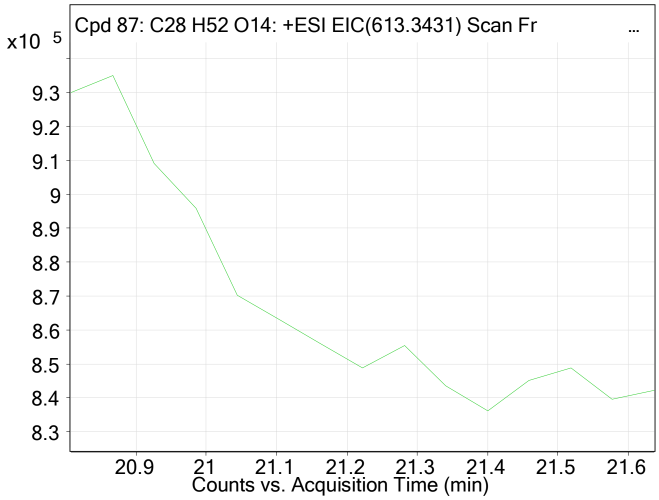

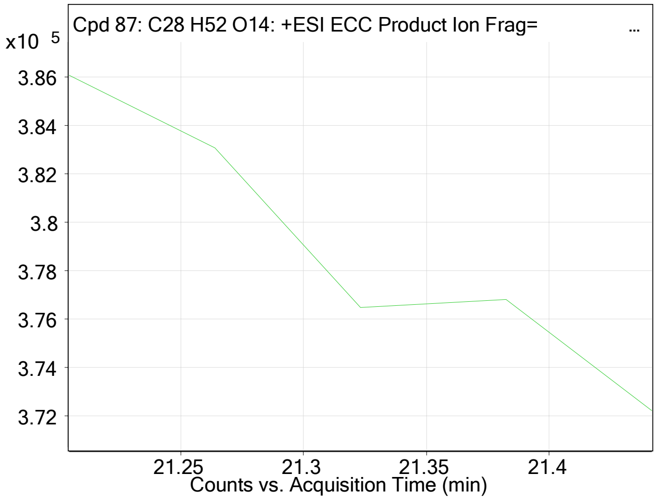

MS Spectrum

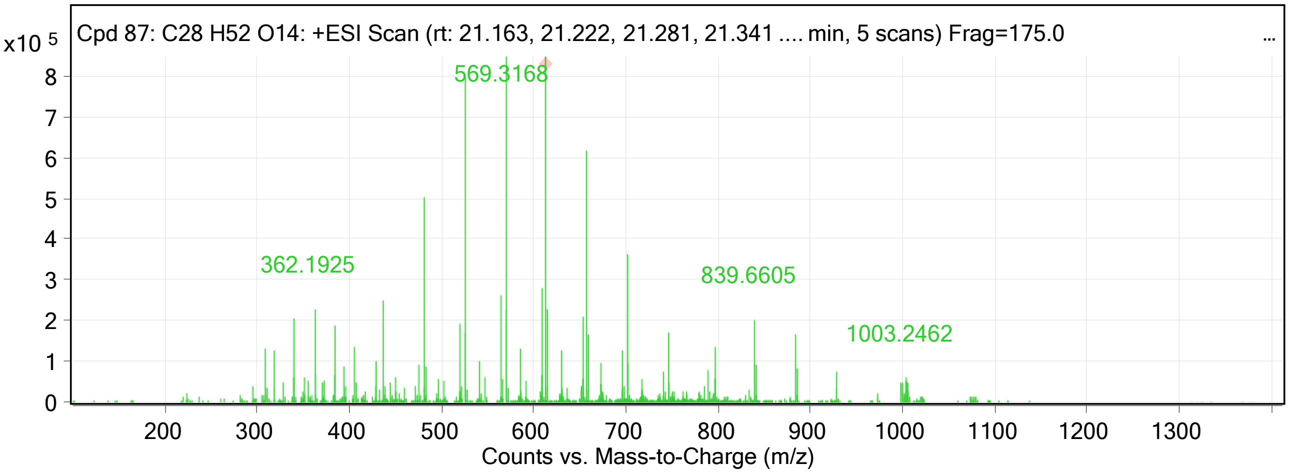

MS Zoomed Spectrum

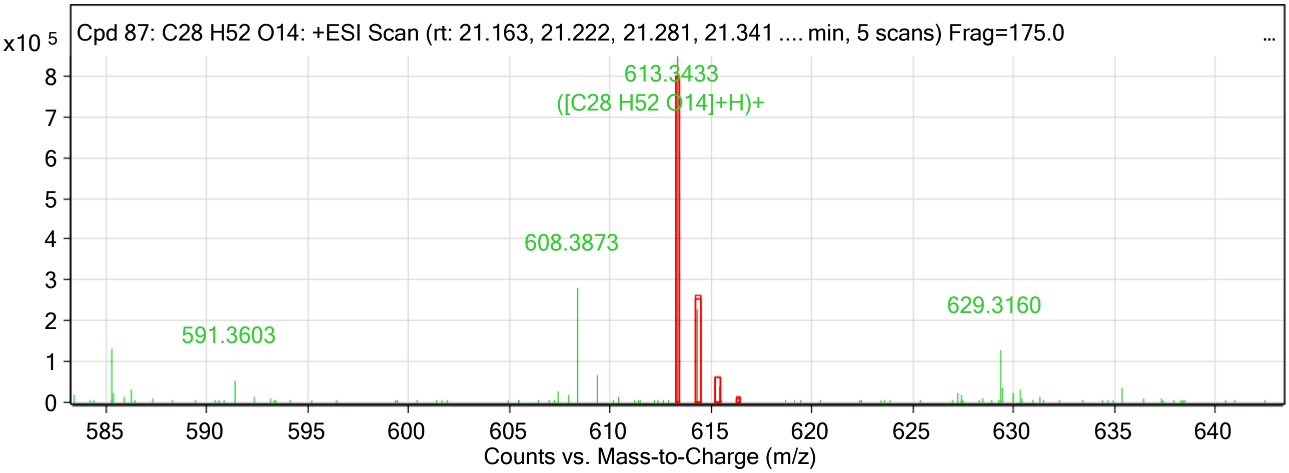

MS Spectrum Peak List

m/z	Calc *m/z*	Diff(ppm)	z	Abund	Formula	Ion
481.2636			1	501663.06		
525.2905			1	794739.75		
569.3168			1	955154.13		
608.3873			1	281792.47		
613.3433	613.343	-0.54	1	847972.63	C28 H52 O14	(M+H)+
614.3459	614.3464	0.8	1	229623.83	C28 H52 O14	(M+H)+
615.3479	615.3489	1.54	1	40640.29	C28 H52 O14	(M+H)+
616.3544	616.3515	-4.58	1	5610.51	C28 H52 O14	(M+H)+
657.3693			1	614591.44		
701.3955			1	364782.47		

MSMS Spectrum

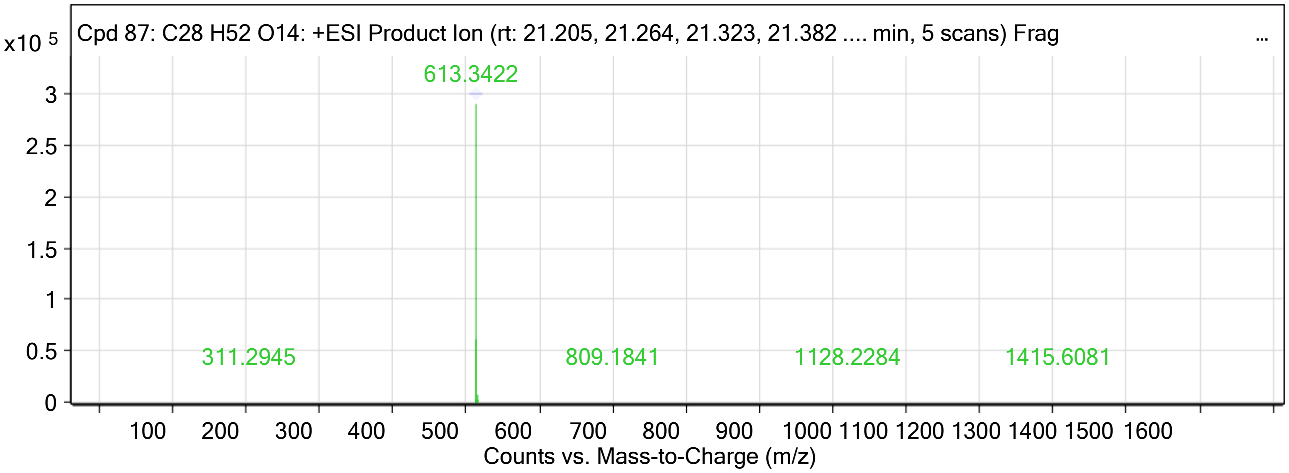

MS/MS Spectrum Peak List

m/z	Calc *m/z*	Diff(ppm)	z	Abund
131.0682	131.0703	16.06		20.29
133.0875	133.0859	-11.55		44.14
175.1317	175.1329	6.91		43.81
177.1131	177.1121	-5.52		28.17
309.279	309.2788	-0.6		62.57
311.2945	311.2945	-0.04		65.64
612.6382				2521.64
613.3422	613.343	1.24	1	289984.38
614.345			1	60023.27
615.3472			1	7287.14

Compound Label	*m/z*	RT	Algorithm	Mass
Cpd 88: C26 H48 O13	569.3169	21.425	Auto MS/MS	568.3095

Compound Chromatograms

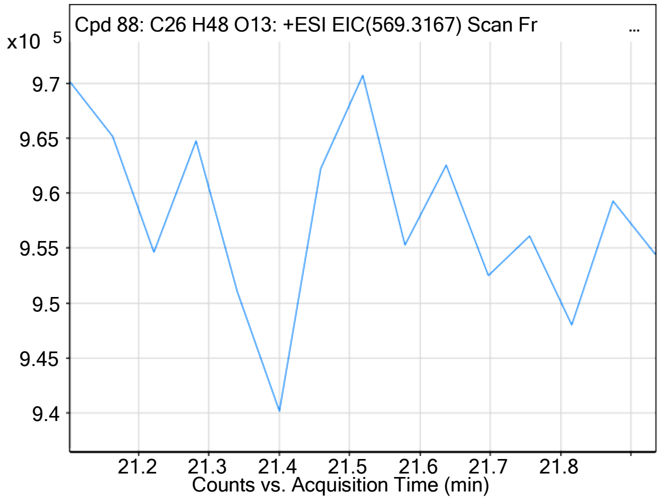

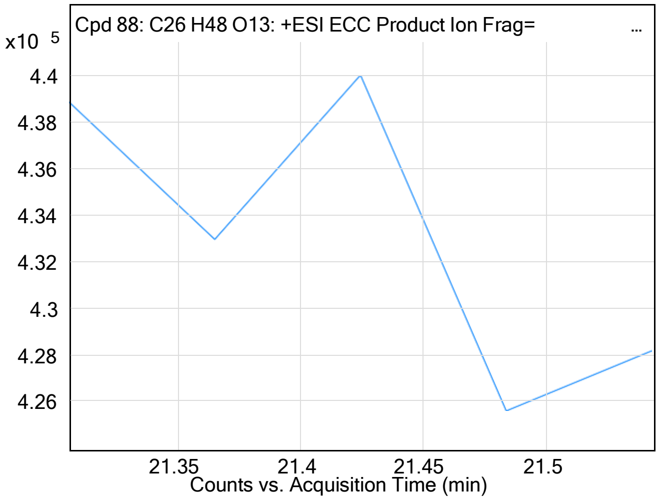

MS Spectrum

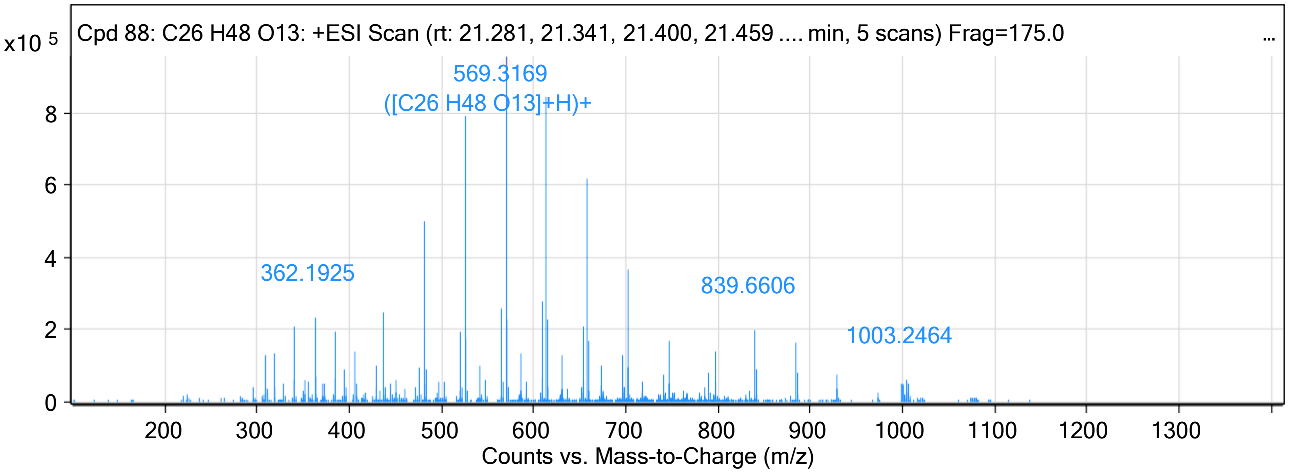

MS Zoomed Spectrum

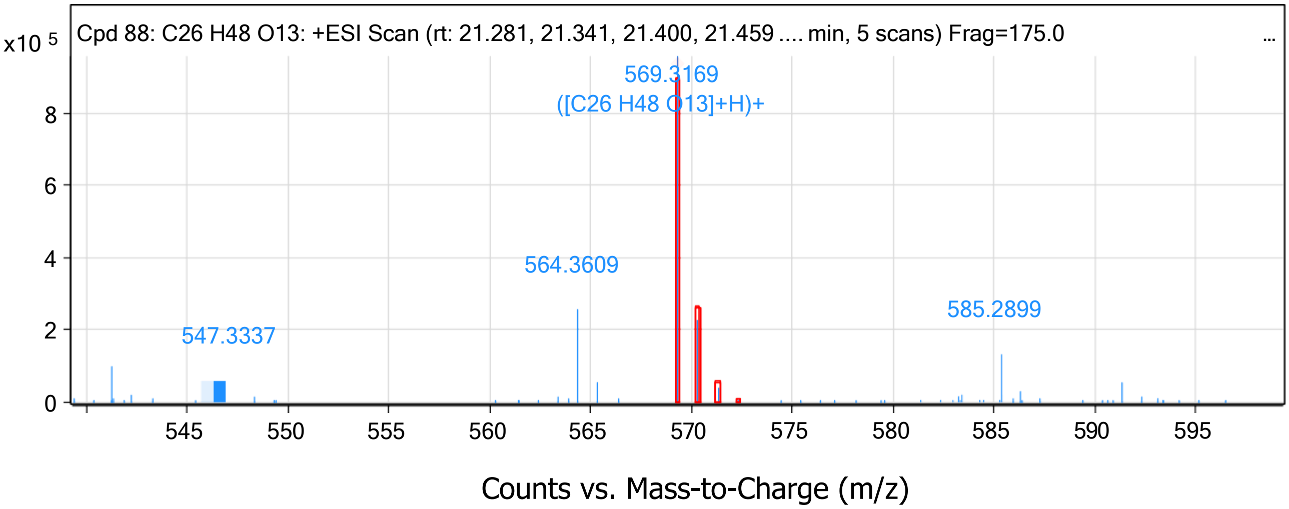

MS Spectrum Peak List

m/z	Calc *m/z*	Diff(ppm)	z	Abund	Formula	Ion
481.2637			1	500838.84		
525.2906			1	791898.19		
569.3169	569.3168	-0.22	1	957778.31	C26 H48 O13	(M+H)+
570.3196	570.3202	0.99	1	227518.22	C26 H48 O13	(M+H)+
571.3216	571.3226	1.74	1	37412.02	C26 H48 O13	(M+H)+
572.3247	572.3253	1.05	1	5202.72	C26 H48 O13	(M+H)+
608.3874			1	277338.25		
613.3434			1	845831.63		
657.3695			1	615305.69		

MSMS Spectrum

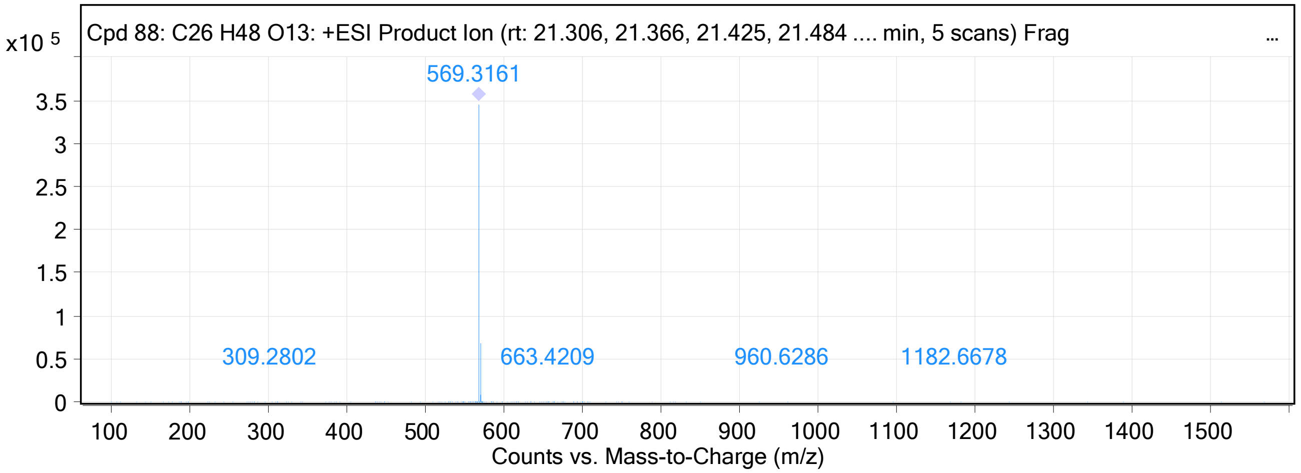

MS/MS Spectrum Peak List

m/z	*Calc m/z*	Diff(ppm)	z	Abund
133.0857	133.0859	1.41		38.3
177.1114	177.1121	4.29		27.08
233.1783	233.1747	-15.35		14.86
283.2585	283.2632	16.49		14.11
309.2802	309.2788	-4.58		37.76
311.2951	311.2945	-2.05	1	34.4
329.2276	329.2323	14.2		22.14
538.2895	538.2984	16.56		14.54
569.3161	569.3168	1.23	1	345400.5
570.3187			1	67577.54

Compound Label	*m/z*	RT	Algorithm	Mass
Cpd 89: C28 H52 O14	613.343	21.62	Auto MS/MS	612.3355

Compound Chromatograms

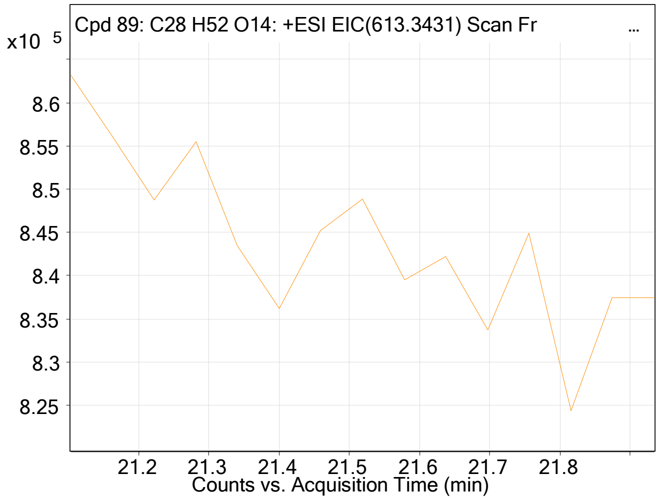

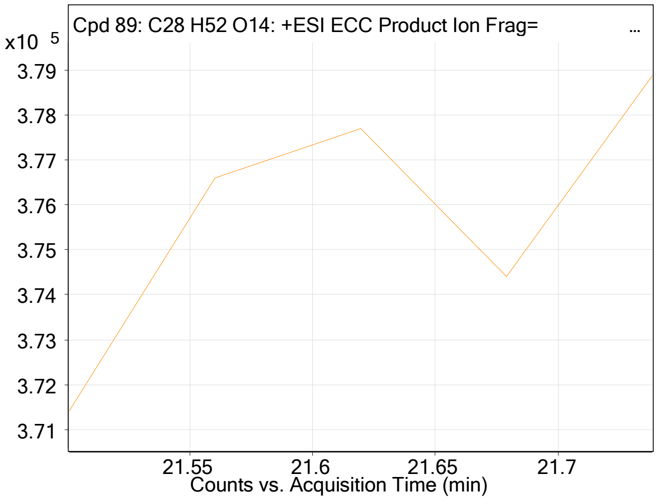

MS Spectrum

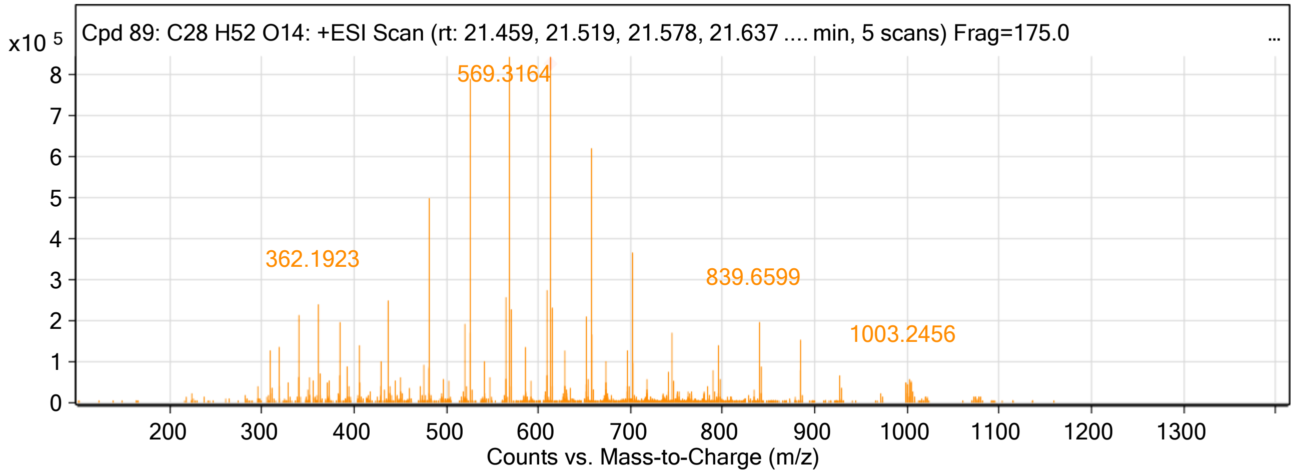

MS Zoomed Spectrum

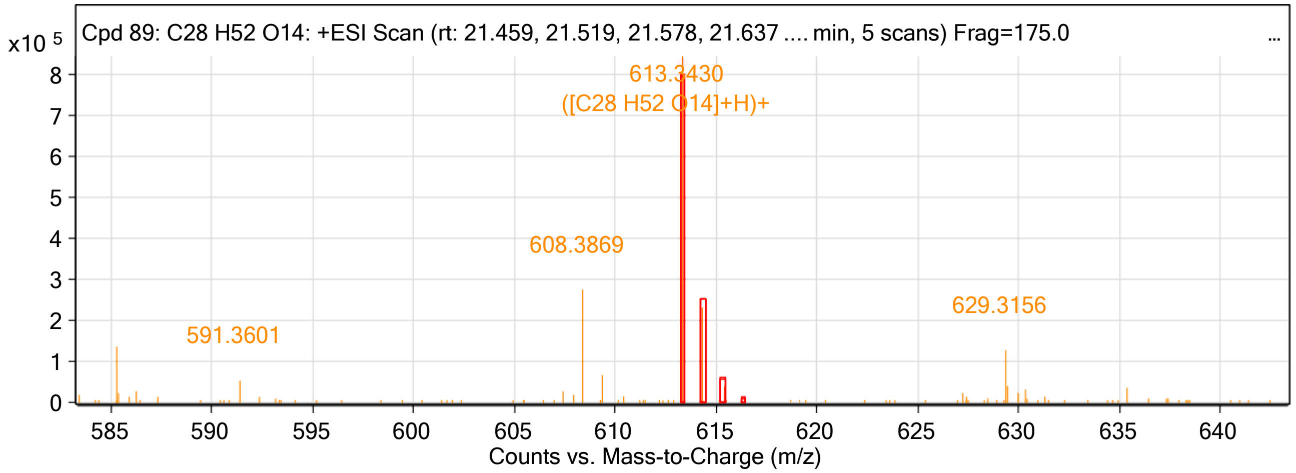

MS Spectrum Peak List

m/z	Calc *m/z*	Diff(ppm)	z	Abund	Formula	Ion
481.2633			1	495701.75		
525.2902			1	787241.63		
569.3164			1	960648		
608.3869			1	274058.81		
613.343	613.343	0.04	1	841898.31	C28 H52 O14	(M+H)+
614.3455	614.3464	1.45	1	228213.03	C28 H52 O14	(M+H)+
615.3476	615.3489	2.09	1	40824.43	C28 H52 O14	(M+H)+
616.3542	616.3515	-4.26	1	5683.57	C28 H52 O14	(M+H)+
657.3689			1	616528.19		
701.395			1	363027.31		

MSMS Spectrum

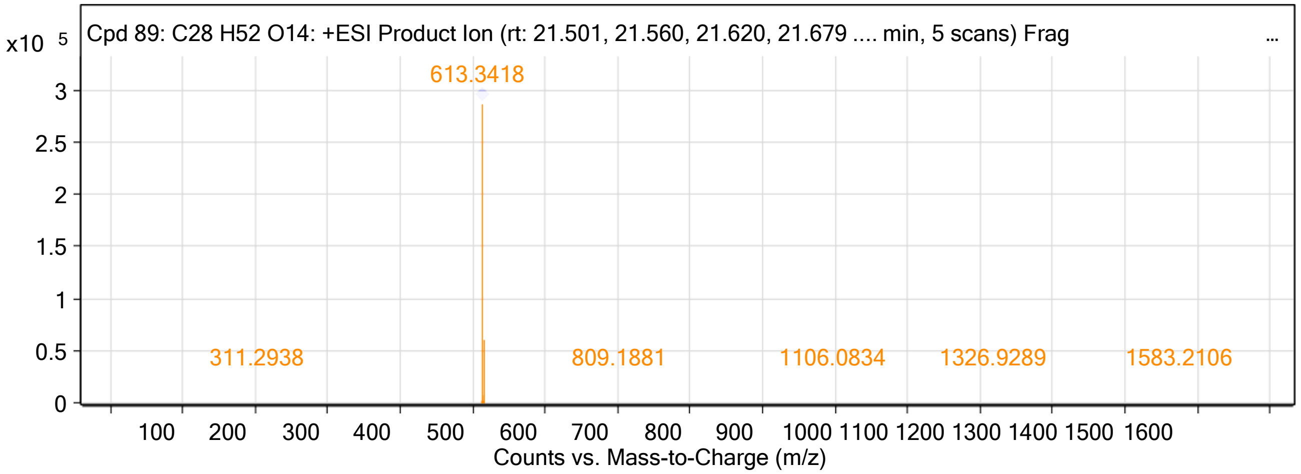

MS/MS Spectrum Peak List

m/z	Calc m/z	Diff(ppm)	z	Abund
177.1116	177.1121	2.75		37.38
309.277	309.2788	5.73	1	57.22
612.3876			2	2152.49
612.638				2497.91
612.8889			2	1930.78
613.1399			1	1067.59
613.3418	613.343	1.86	1	286156.59
614.3445			1	60805.26
615.347			1	7120.01
615.414			2	1270.65

Compound Label	m/z	RT	Algorithm	Mass
Cpd 90: C24 H46 N3 O12	569.3162	21.722	Auto MS/MS	568.3088

Compound Chromatograms

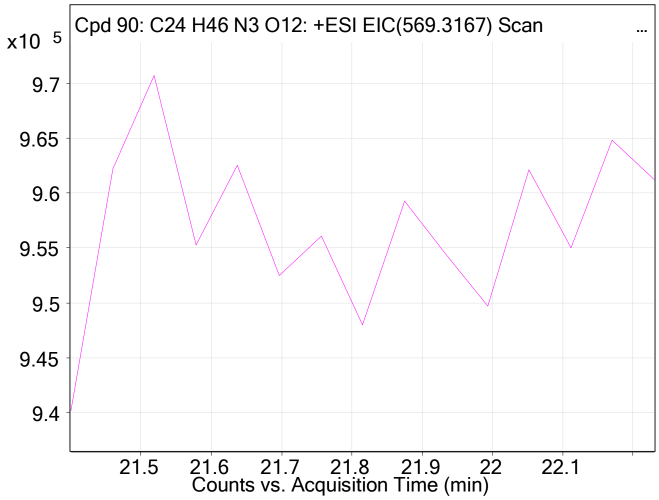

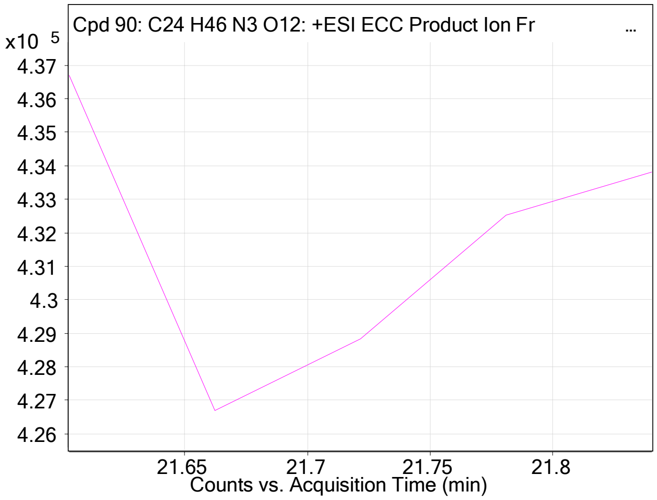

MS Spectrum

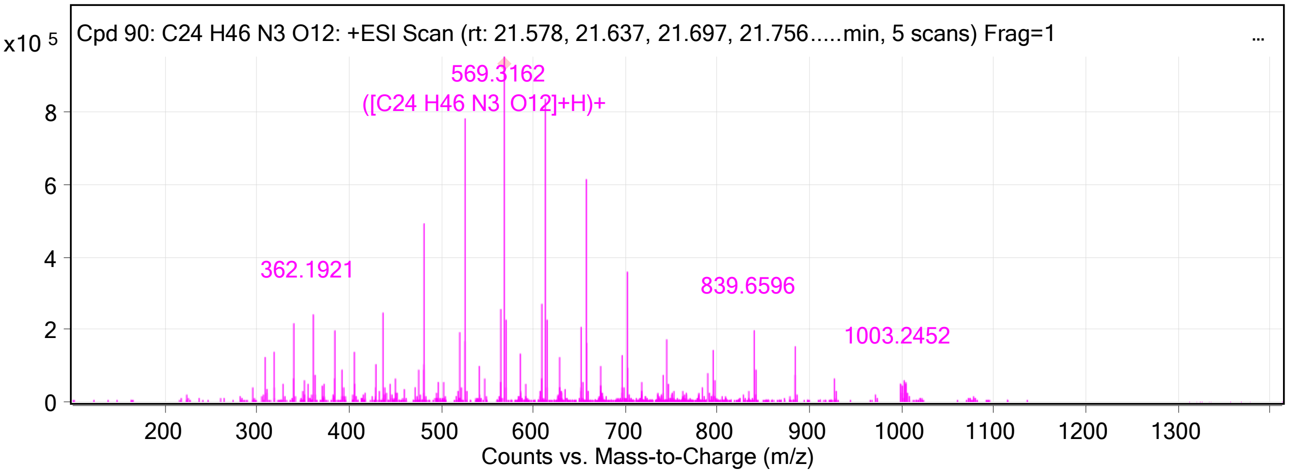

MS Zoomed Spectrum

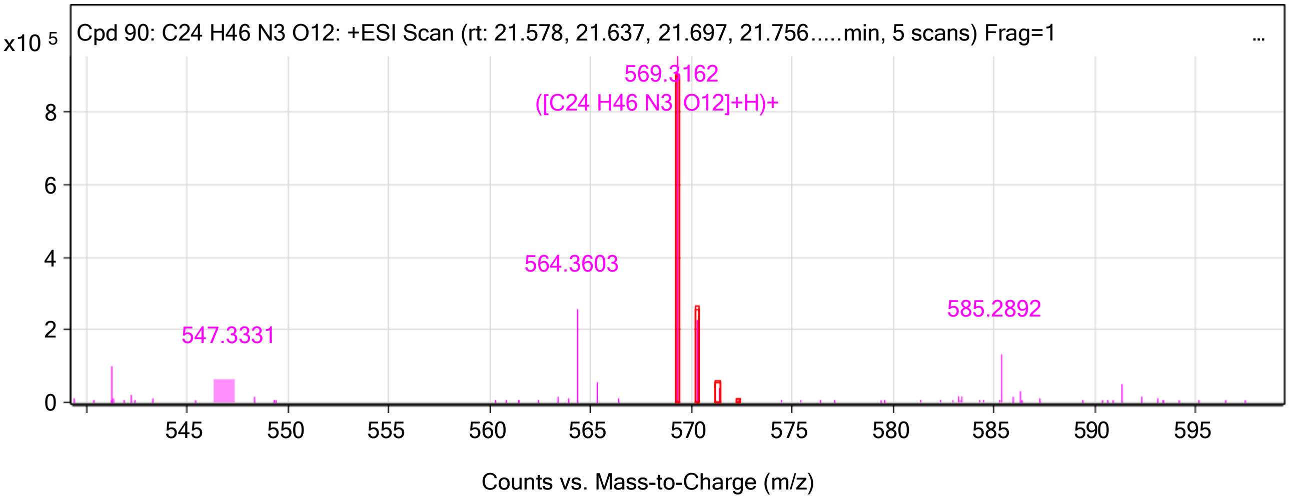

MS Spectrum Peak List

m/z	Calc *m/z*	Diff(ppm)	z	Abund	Formula	Ion
481.2631			1	492522.69		
525.29			1	784522.88		
569.3162	569.3154	-1.38	1	954876	C24 H46 N3 O12	(M+H)+
570.3189	570.3186	-0.53	1	227012.47	C24 H46 N3 O12	(M+H)+
571.321	571.3209	-0.08	1	37717.49	C24 H46 N3 O12	(M+H)+
572.3237	572.3235	-0.32	1	5193.49	C24 H46 N3 O12	(M+H)+
608.3867			1	272816.72		
613.3428			1	836940.69		
657.3687			1	613375.44		
701.3948			1	360622.69		

MSMS Spectrum

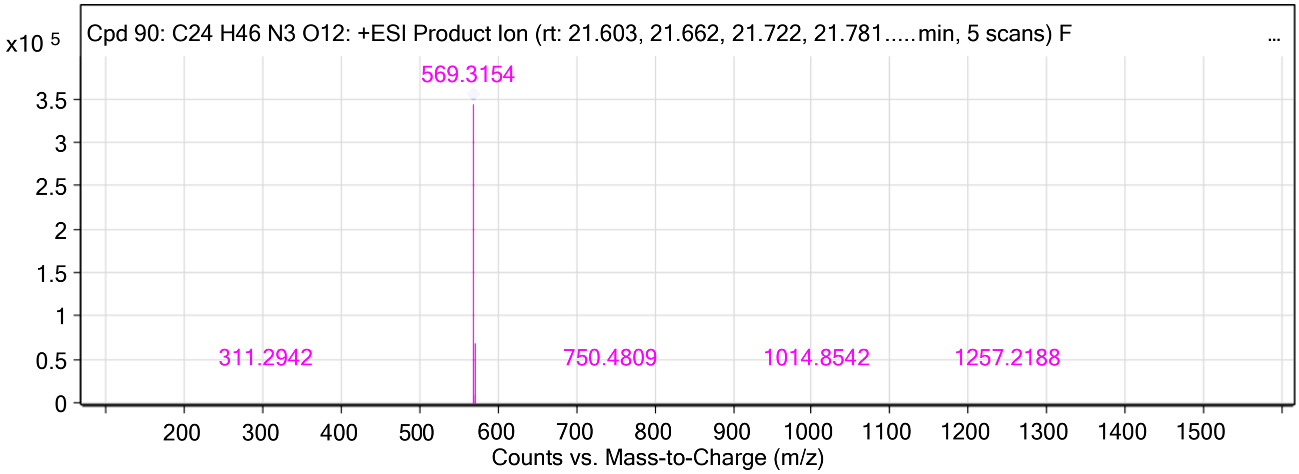

MS/MS Spectrum Peak List

m/z	Calc *m/z*	Diff(ppm)	z	Abund
108.0866	108.0808	-53.49		19.86
133.0852	133.0846	-5.02		33.76
239.1473	239.1476	1.33		15.3
311.2942	311.2945	0.79		50
387.2802	193.6424	-499994.09	2	16.05
538.2942	269.1482	-499997.83	2	32.71
568.6116				737.61
569.3154	569.3154	-0.03	1	343816.41
570.3179			1	67716.4
571.3203			1	8382.67

Compound Label	*m/z*	RT	Algorithm	Mass
Cpd 91: C28 H52 O14	613.3433	21.916	Auto MS/MS	612.3358

Compound Chromatograms

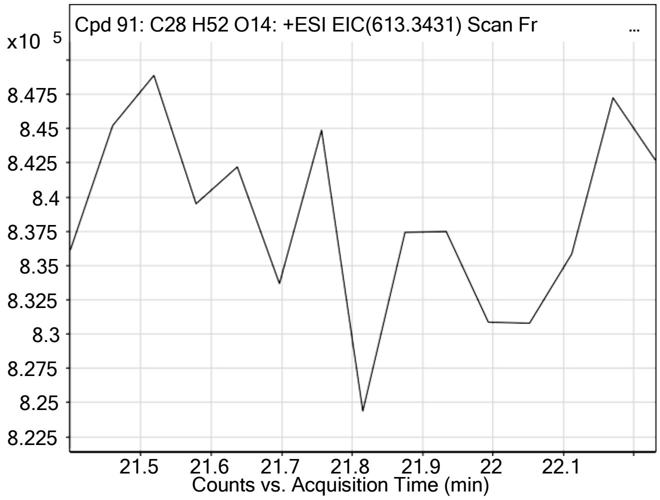

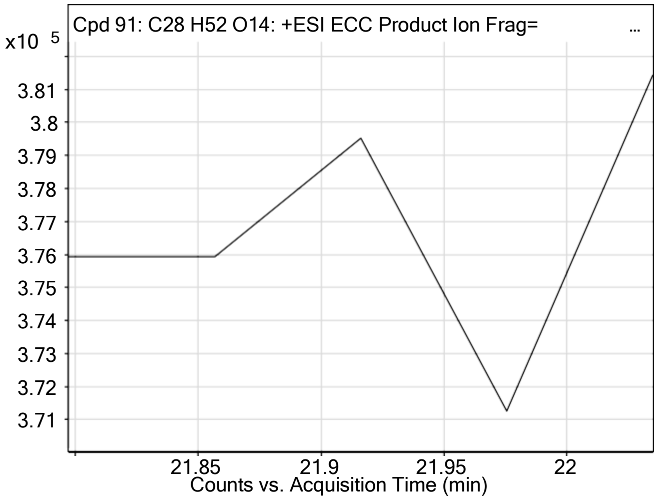

MS Spectrum

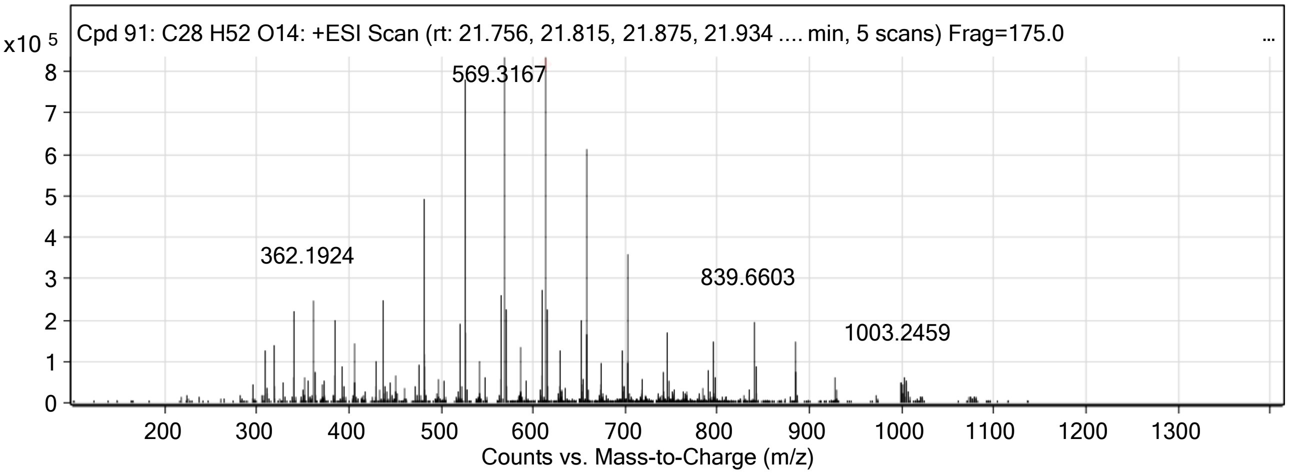

MS Zoomed Spectrum

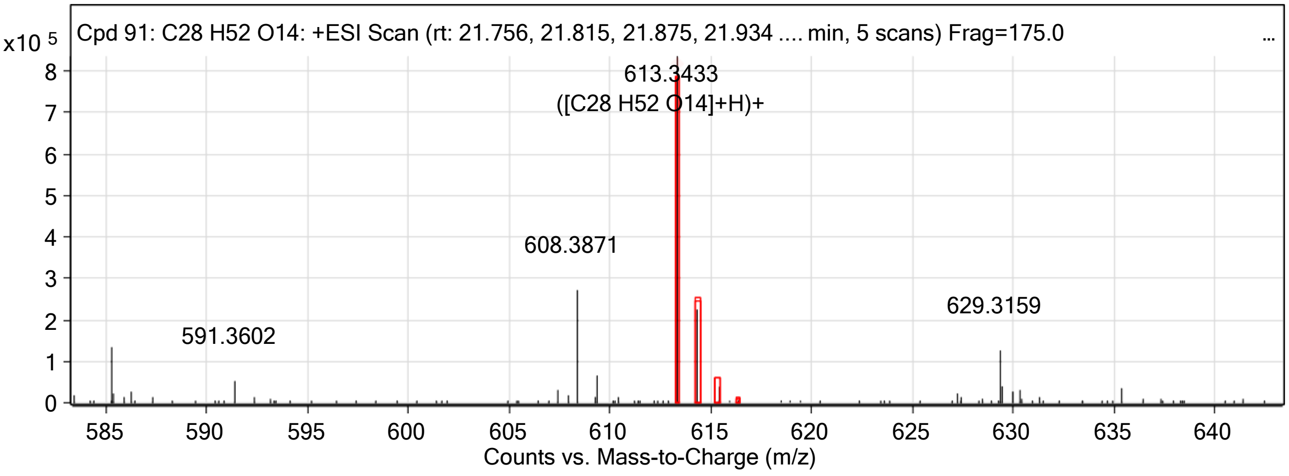

MS Spectrum Peak List

m/z	Calc *m/z*	Diff(ppm)	z	Abund	Formula	Ion
481.2635			1	491774.94		
525.2904			1	781088.13		
569.3167			1	953497		
608.3871			1	272284.03		
613.3433	613.343	-0.45	1	835016.13	C28 H52 O14	(M+H)+
614.3458	614.3464	1.02	1	224694.7	C28 H52 O14	(M+H)+
615.3478	615.3489	1.7	1	40432.45	C28 H52 O14	(M+H)+
616.3555	616.3515	-6.42	1	5626.62	C28 H52 O14	(M+H)+
657.3692			1	609712.44		

MSMS Spectrum

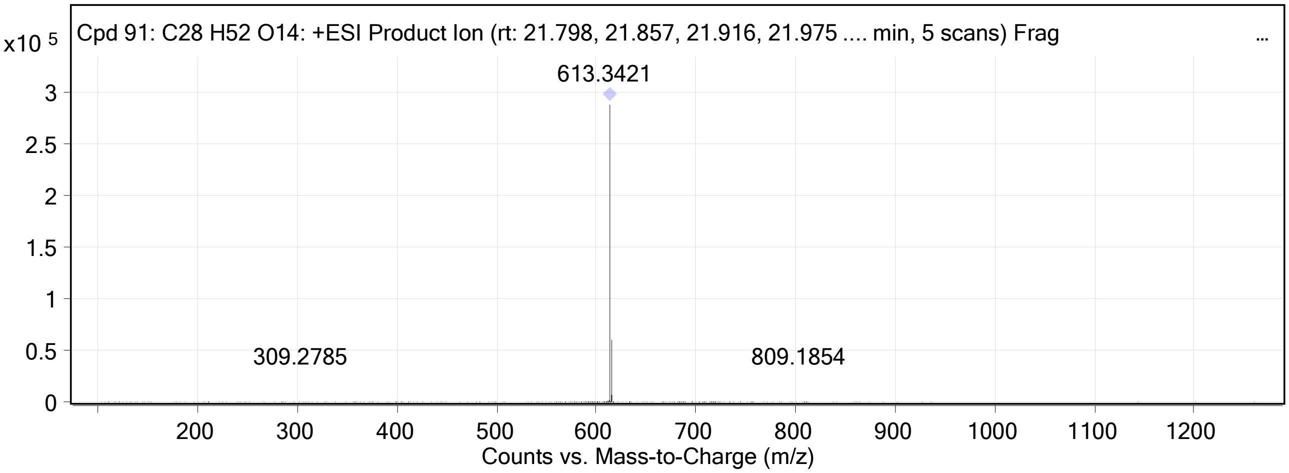

MS/MS Spectrum Peak List

m/z	*Calc m/z*	Diff(ppm)	z	Abund
199.1264	199.1329	32.42		26
309.2785	309.2788	1.1		73.19
311.2939	311.2945	1.79		68.23
357.0677	357.0664	-3.76	1	22.36
609.3124	304.6556	-500001.05	2	59.81
612.3281	612.3352	11.47		45.27
612.6386			2	2308.37
613.3421	613.343	1.47	1	287998.41
614.3448			1	59611.01
615.347			1	7294.69

Compound Label	*m/z*	RT	Algorithm	Mass
Cpd 92: C24 H46 N3 O12	569.3166	22.018	Auto MS/MS	568.3092

Compound Chromatograms

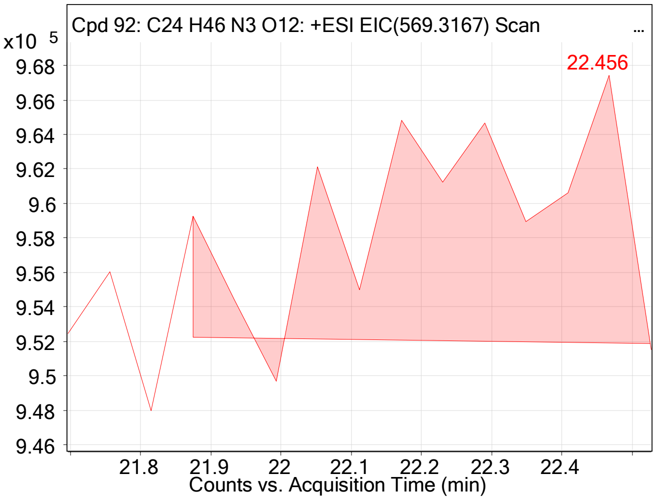

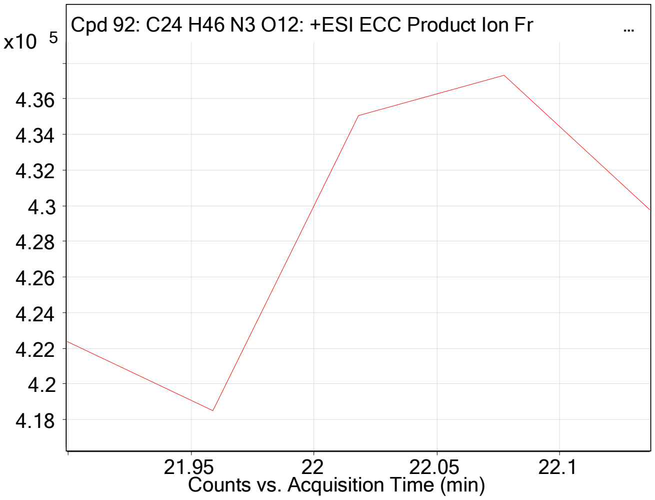

MS Spectrum

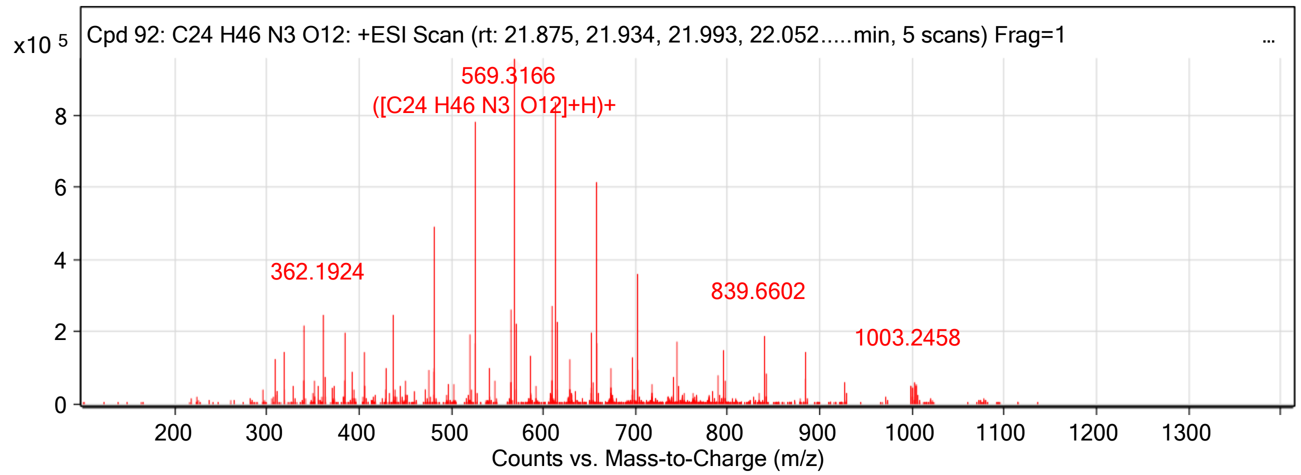

MS Zoomed Spectrum

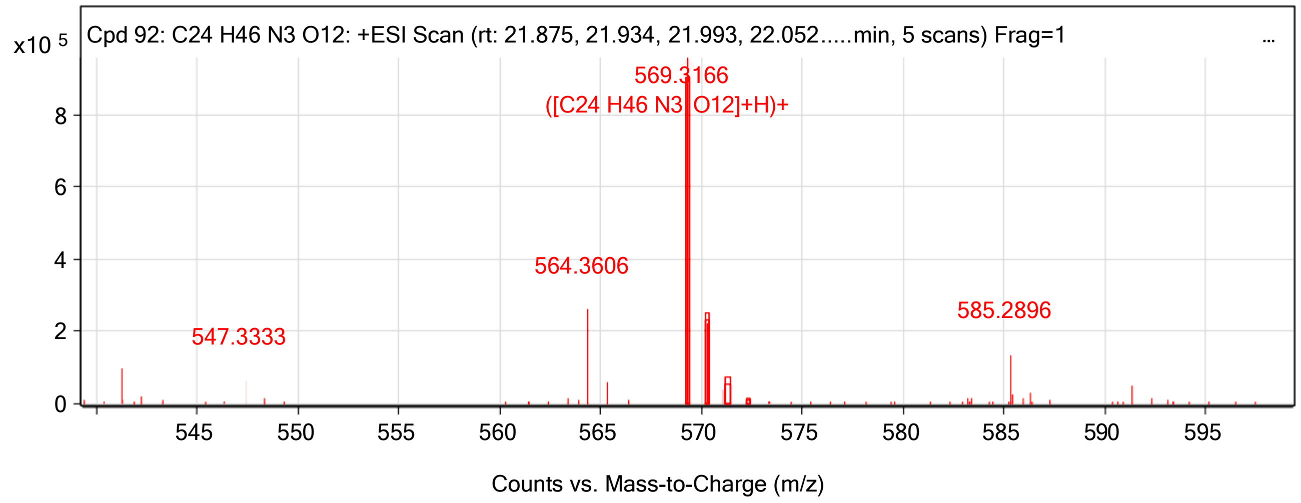

MS Spectrum Peak List

m/z	Calc *m/z*	Diff(ppm)	z	Abund	Formula	Ion
481.2634			1	489483.41		
525.2904			1	781387.13		
569.3166	569.3154	-2.1	1	956116.19	C24 H46 N3 O12	(M+H)+
570.3193	570.3186	-1.2	1	221844.59	C24 H46 N3 O12	(M+H)+
571.3214	571.3209	-0.79	1	38110.2	C24 H46 N3 O12	(M+H)+
572.324	572.3235	-0.82	1	5196.88	C24 H46 N3 O12	(M+H)+
608.387			1	273800.06		
613.3432			1	834492.63		
657.3692			1	612728.56		
701.3952			1	360556.22		

MSMS Spectrum

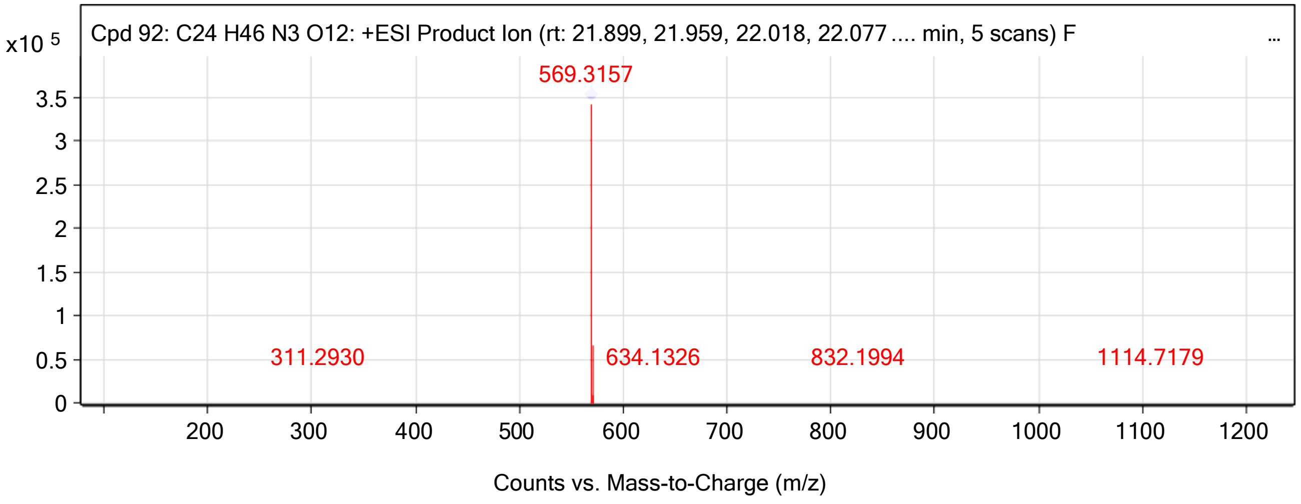

MS/MS Spectrum Peak List

m/z	*Calc m/z*	Diff(ppm)	z	Abund
107.0706	107.0689	-15.76		15.48
133.0864	133.0859	-3.8		67.99
221.1389	221.1384	-2.39		21.3
283.2634	283.2632	-0.76		21.59
309.2773	309.2775	0.69		16.59
311.293	311.2931	0.43		50.43
538.294	269.1482	-499997.69	2	22.24
539.2932			2	19.45
566.2861	566.292	10.35		31.04
569.3157	569.3154	-0.44	1	342000.69

Compound Label	*m/z*	RT	Algorithm	Mass
Cpd 93: C28 H52 O14	613.3431	22.213	Auto MS/MS	612.3356

Compound Chromatograms

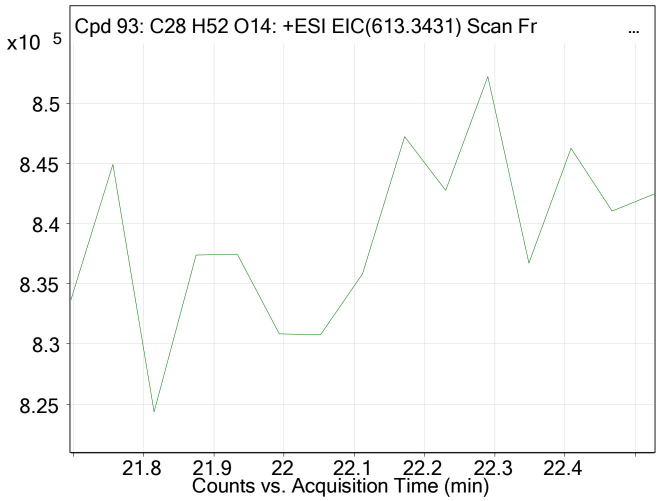

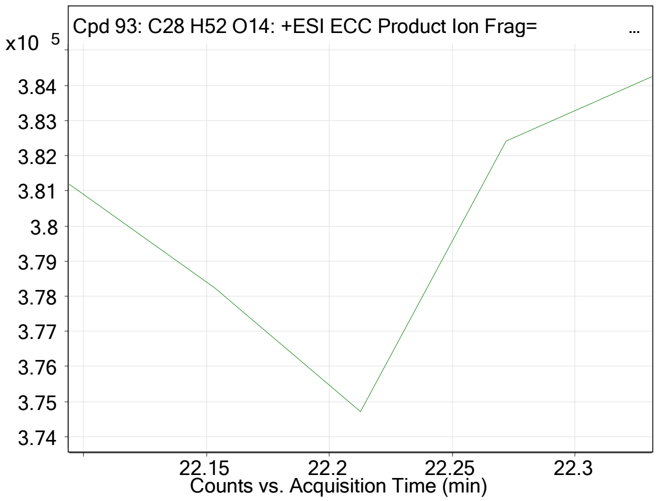

MS Spectrum

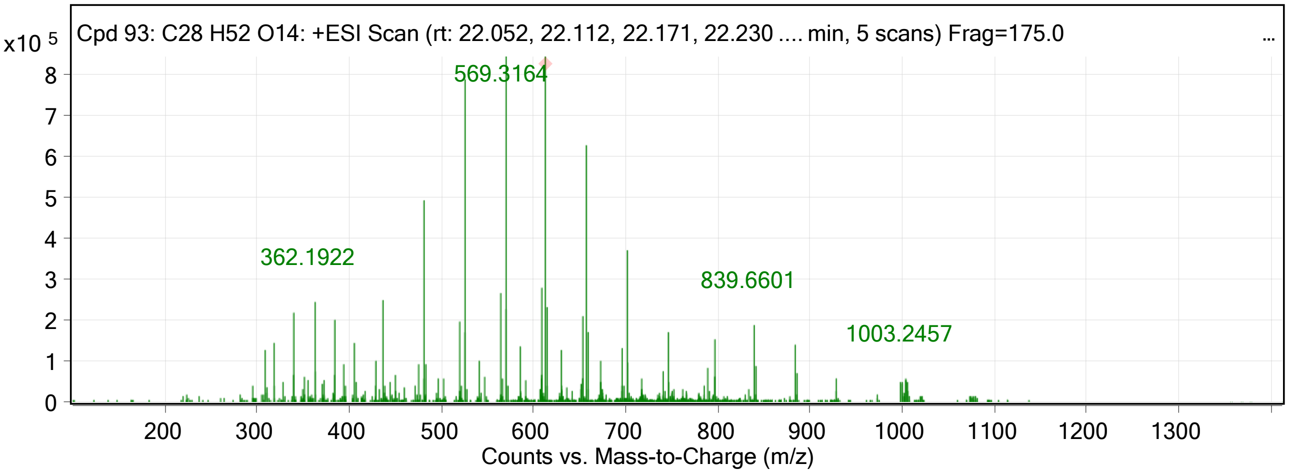

MS Zoomed Spectrum

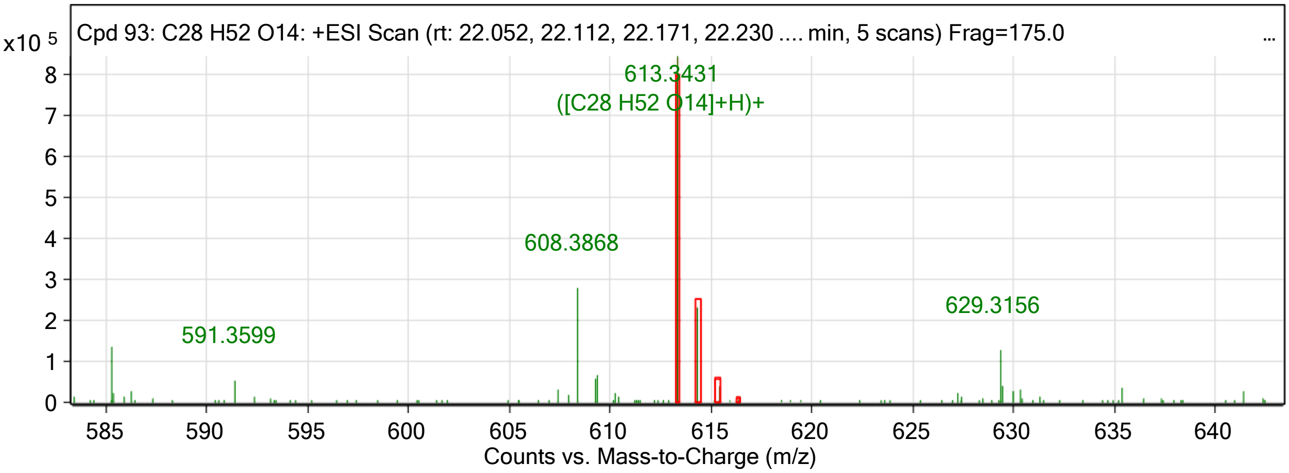

MS Spectrum Peak List

m/z	*Calc m/z*	Diff(ppm)	z	Abund	Formula	Ion
481.2632			1	492512.5		
525.2902			1	786330.5		
569.3164			1	961577.81		
608.3868			1	278985.5		
613.3431	613.343	-0.12	1	841771.38	C28 H52 O14	(M+H)+
614.3456	614.3464	1.32	1	228470.91	C28 H52 O14	(M+H)+
615.3474	615.3489	2.29	1	40553.1	C28 H52 O14	(M+H)+
616.3535	616.3515	-3.2	1	5969.89	C28 H52 O14	(M+H)+
657.369			1	624067.44		
701.395			1	366925.84		

MSMS Spectrum

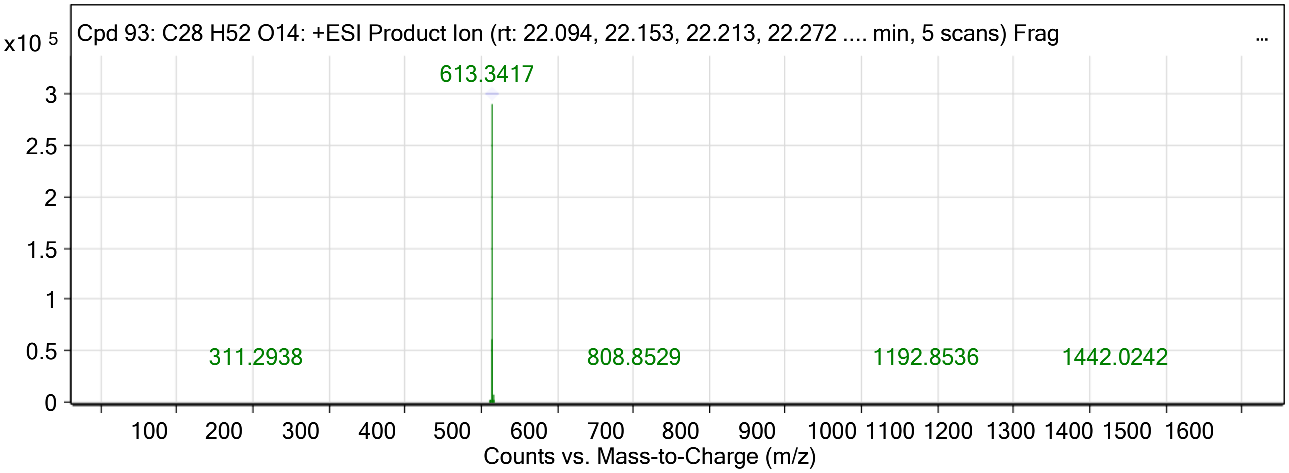

MS/MS Spectrum Peak List

m/z	*Calc m/z*	Diff(ppm)	z	Abund
133.0851	133.0859	6.06		53.17
309.2788	309.2788	-0.06	1	46.9
612.3877			2	1962.45
612.6376			2	2428.47
612.8896			2	1866.38
613.1412			2	1092.05
613.3417	613.343	2.14	1	290183.84
614.3444			1	60950.44
615.3468			1	7448.76
615.4139			2	1115.01

Compound Label	*m/z*	RT	Algorithm	Mass
Cpd 94: C26 H48 O13	569.3168	22.315	Auto MS/MS	568.3093

Compound Chromatograms

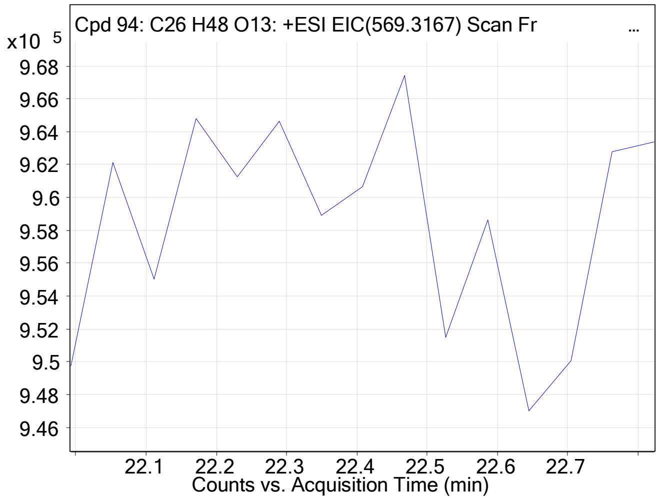

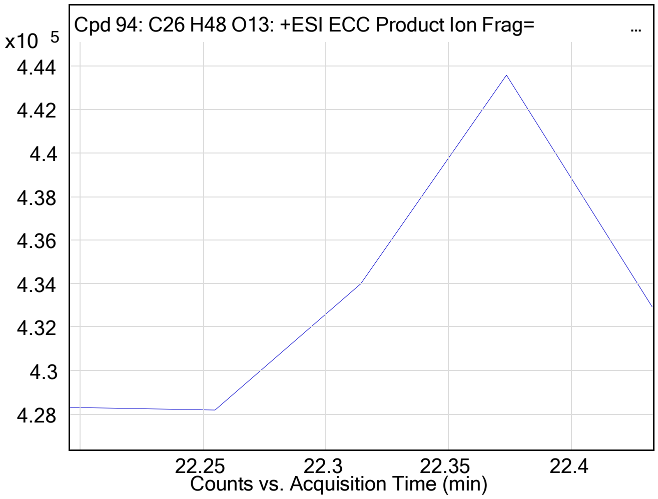

MS Spectrum

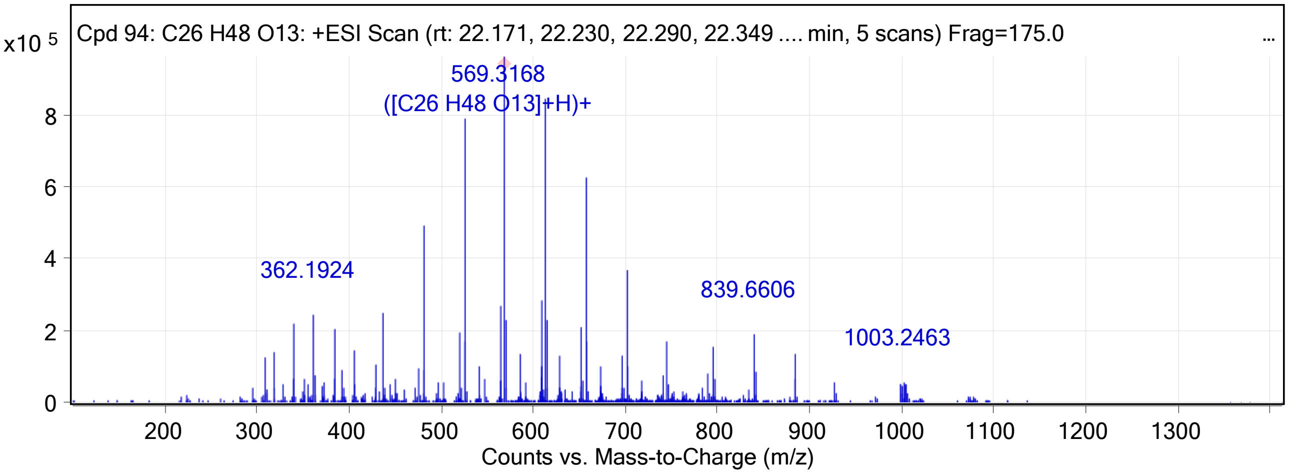

MS Zoomed Spectrum

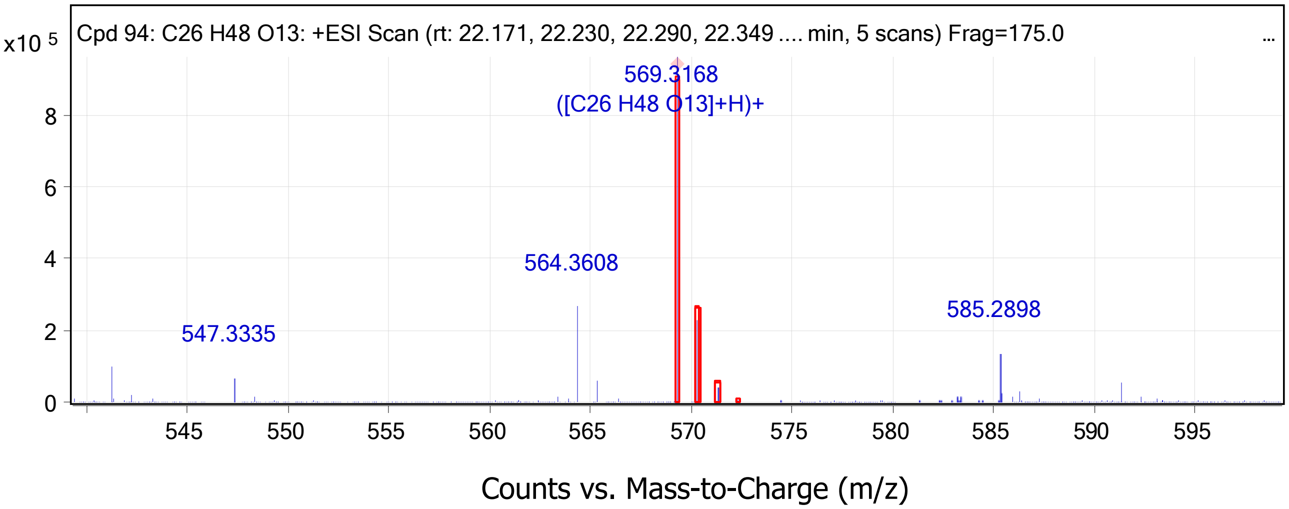

MS Spectrum Peak List

m/z	Calc *m/z*	Diff(ppm)	z	Abund	Formula	Ion
481.2635			1	493414.44		
525.2905			1	788289.56		
569.3168	569.3168	-0.06	1	962063.19	C26 H48 O13	(M+H)+
570.3195	570.3202	1.21	1	227453	C26 H48 O13	(M+H)+
571.3215	571.3226	1.95	1	38805.34	C26 H48 O13	(M+H)+
572.3244	572.3253	1.54	1	5585.56	C26 H48 O13	(M+H)+
608.3873			1	282260.56		
613.3435			1	845043		
657.3695			1	626398.5		

MSMS Spectrum

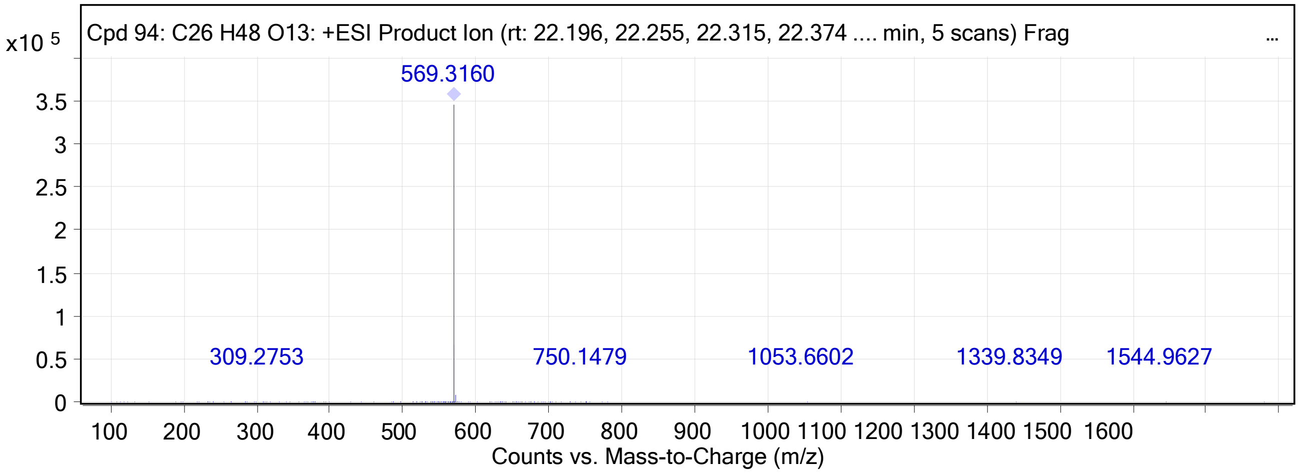

MS/MS Spectrum Peak List

m/z	*Calc m/z*	Diff(ppm)	z	Abund
101.096	101.0961	0.96		11.26
133.0855	133.0859	3.24		38.72
188.1027	188.1043	8.39		14.1
265.1639	265.1646	2.66		11.62
285.2009	285.206	18.01		11.55
309.2753	309.2788	11.43		32.25
311.2948	311.2945	-1.14	1	27.74
318.24	318.2401	0.24		11.62
443.2661	443.2639	-4.9		11.31
569.316	569.3168	1.39	1	346291.81

Compound Label	*m/z*	RT	Algorithm	Mass
Cpd 95: C28 H52 O14	613.3434	22.509	Auto MS/MS	612.3359

Compound Chromatograms

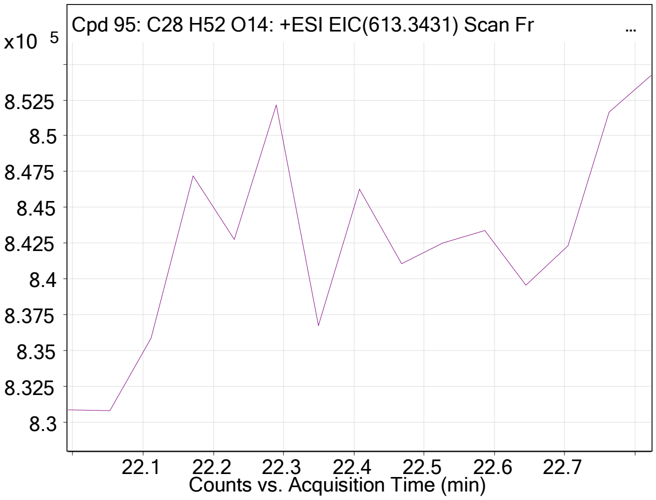

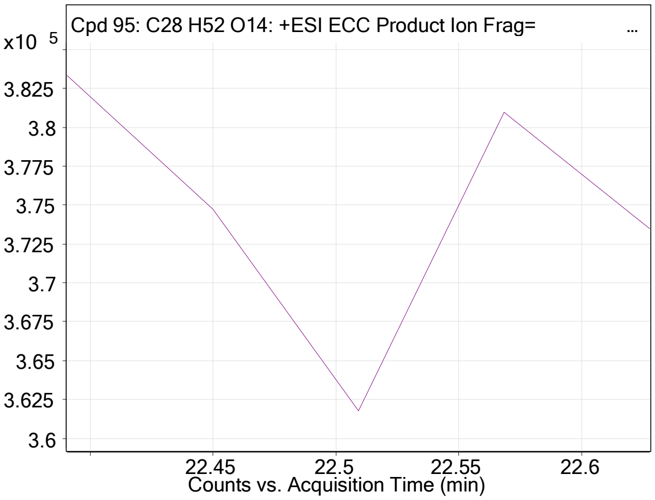

MS Spectrum

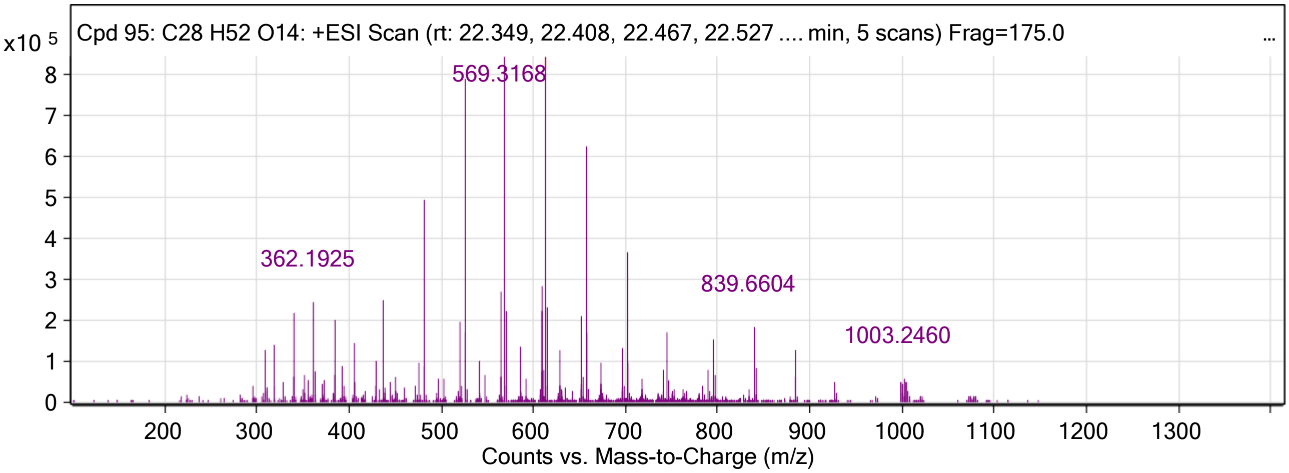

MS Zoomed Spectrum

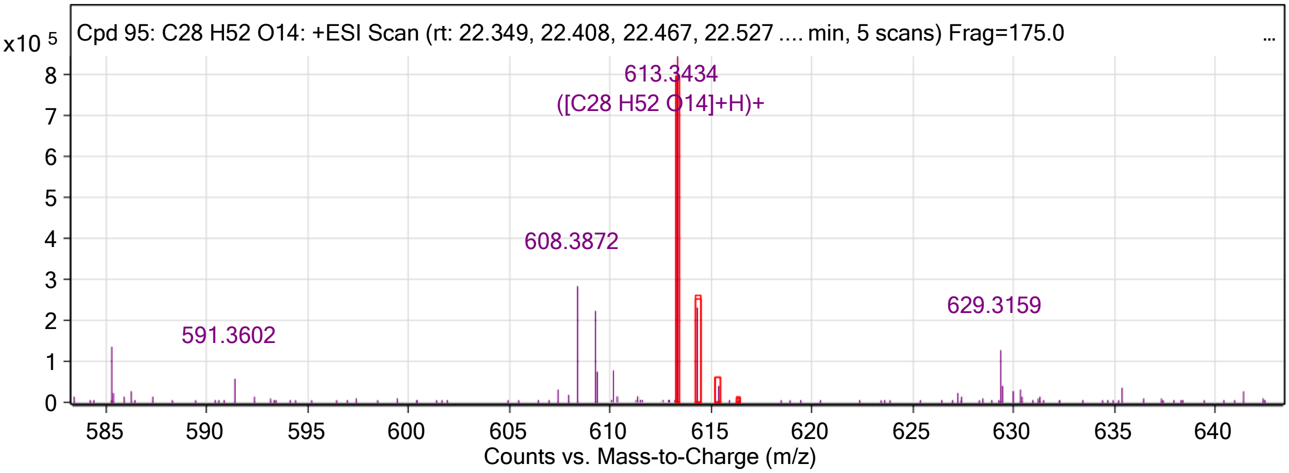

MS Spectrum Peak List

m/z	Calc *m/z*	Diff(ppm)	z	Abund	Formula	Ion
481.2636			1	490970.91		
525.2905			1	786835.38		
569.3168			1	959434.88		
608.3872			1	284346.44		
613.3434	613.343	-0.62	1	841986.88	C28 H52 O14	(M+H)+
614.3459	614.3464	0.87	1	228758.45	C28 H52 O14	(M+H)+
615.3478	615.3489	1.68	1	41237.84	C28 H52 O14	(M+H)+
616.3538	616.3515	-3.71	1	5973.26	C28 H52 O14	(M+H)+
657.3694			1	619504.88		
701.3954			1	366502.94		

MSMS Spectrum

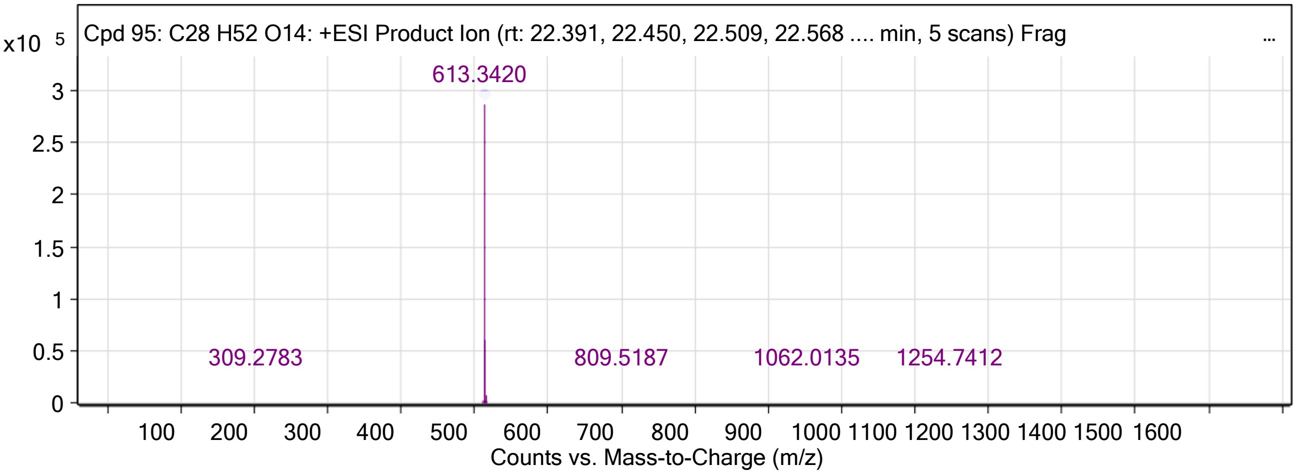

MS/MS Spectrum Peak List

m/z	*Calc m/z*	Diff(ppm)	z	Abund
117.0926	117.091	-13.21		15.83
296.1526	296.1466	-20.26		27.7
309.2783	309.2788	1.78		44.72
311.2954	311.2945	-3.04		31.51
357.0705	357.0664	-11.5		25.14
609.3142	304.6556	-500002.55	2	55.48
612.3396	612.3352	-7.24		45.62
613.342	613.343	1.57	1	286754.16
614.3447			1	59750.12
615.347			1	7428.98

Compound Label	*m/z*	RT	Algorithm	Mass
Cpd 96: C24 H46 N3 O12	569.3165	22.611	Auto MS/MS	568.3091

Compound Chromatograms

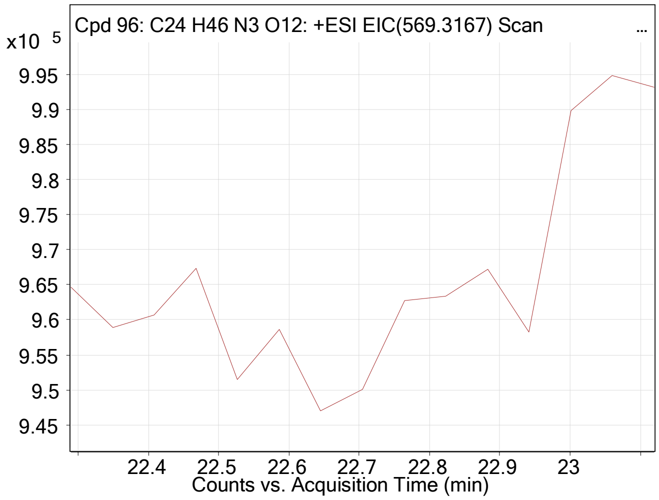

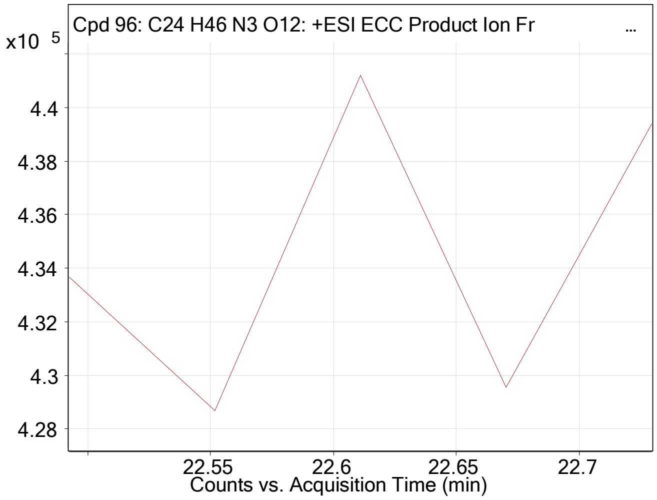

MS Spectrum

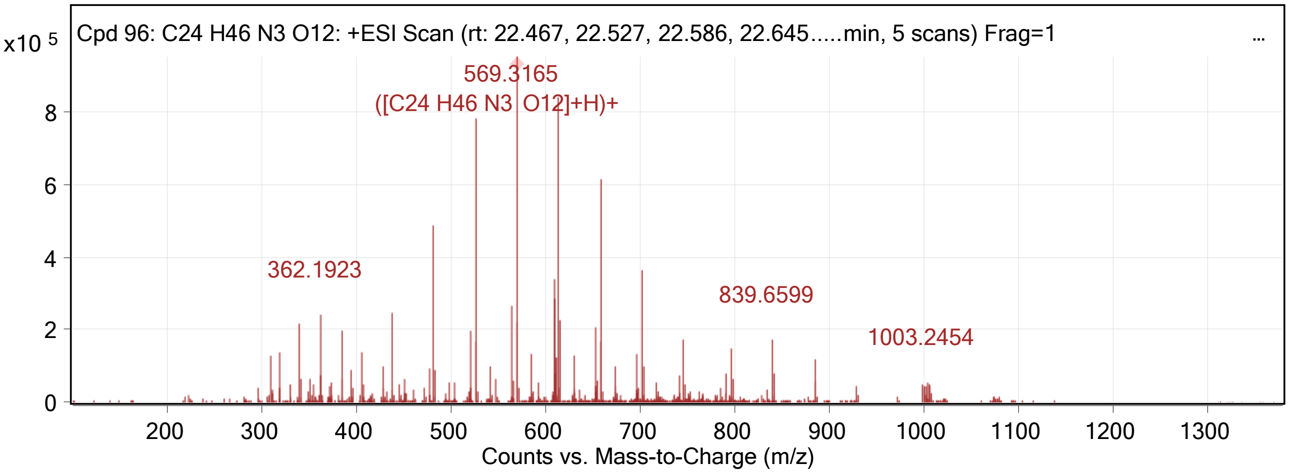

MS Zoomed Spectrum

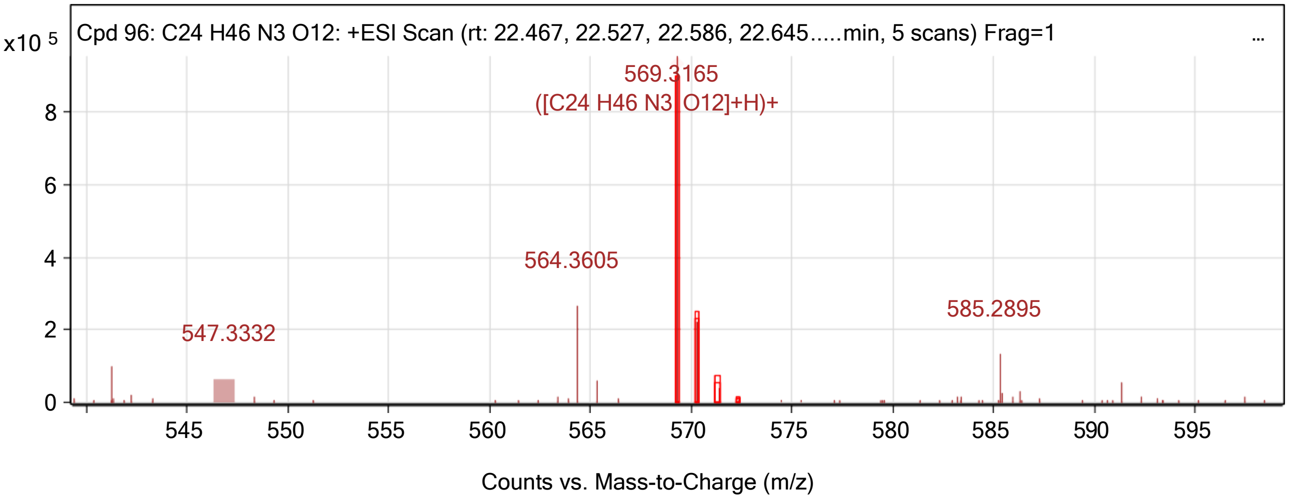

MS Spectrum Peak List

m/z	Calc *m/z*	Diff(ppm)	z	Abund	Formula	Ion
481.2633			1	487133.31		
525.2902			1	782508.81		
569.3165	569.3154	-1.87	1	954932	C24 H46 N3 O12	(M+H)+
570.3192	570.3186	-0.97	1	221608.91	C24 H46 N3 O12	(M+H)+
571.3213	571.3209	-0.54	1	38150.71	C24 H46 N3 O12	(M+H)+
572.3239	572.3235	-0.73	1	5204.28	C24 H46 N3 O12	(M+H)+
609.2725			1	342275.94		
613.343			1	841752.31		
657.369			1	617424.5		
701.395			1	366533.5		

MSMS Spectrum

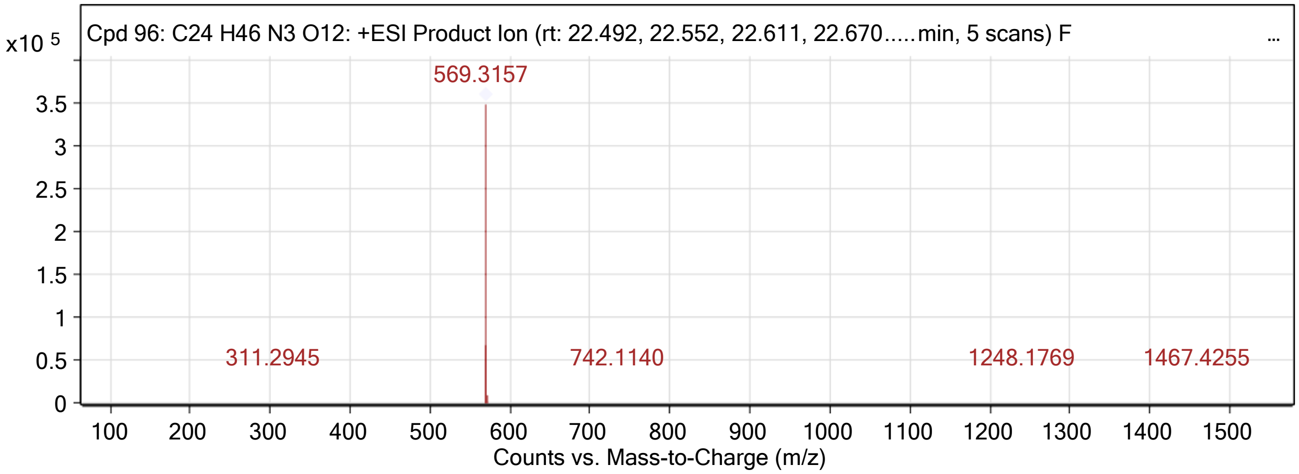

MS/MS Spectrum Peak List

m/z	Calc *m/z*	Diff(ppm)	z	Abund
133.0849	133.0846	-2.34		25.78
151.0974	151.0992	11.73		22.42
195.1217	195.1214	-1.81		18.37
309.2779	309.2775	-1.29		32.25
311.2945	311.2945	-0.22		52.8
568.3619			2	740.84
568.6119			2	640.99
569.3157	569.3154	-0.41	1	348047.66
570.3183			1	67213.73
571.3205			1	8028.4

Compound Label	*m/z*	RT	Algorithm	Mass
Cpd 97: C28 H52 O14	613.3429	22.806	Auto MS/MS	612.3354

Compound Chromatograms

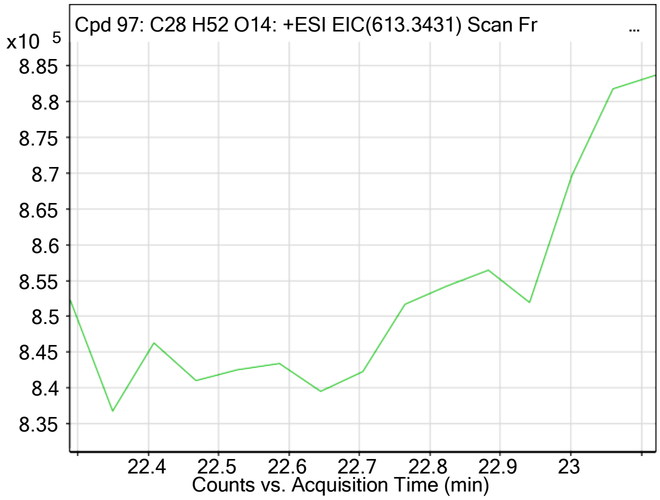

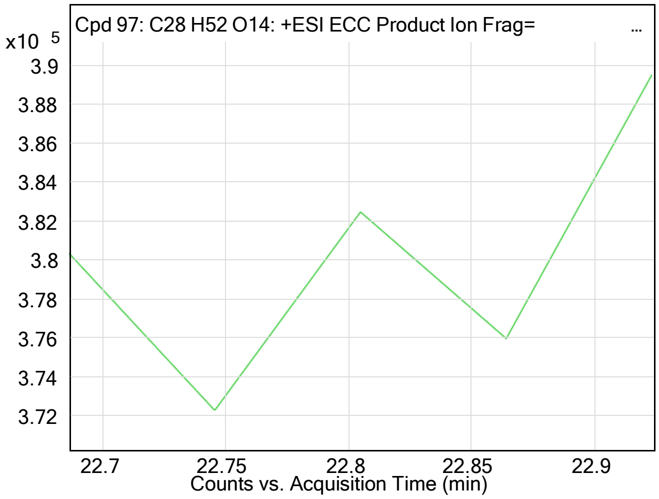

MS Spectrum

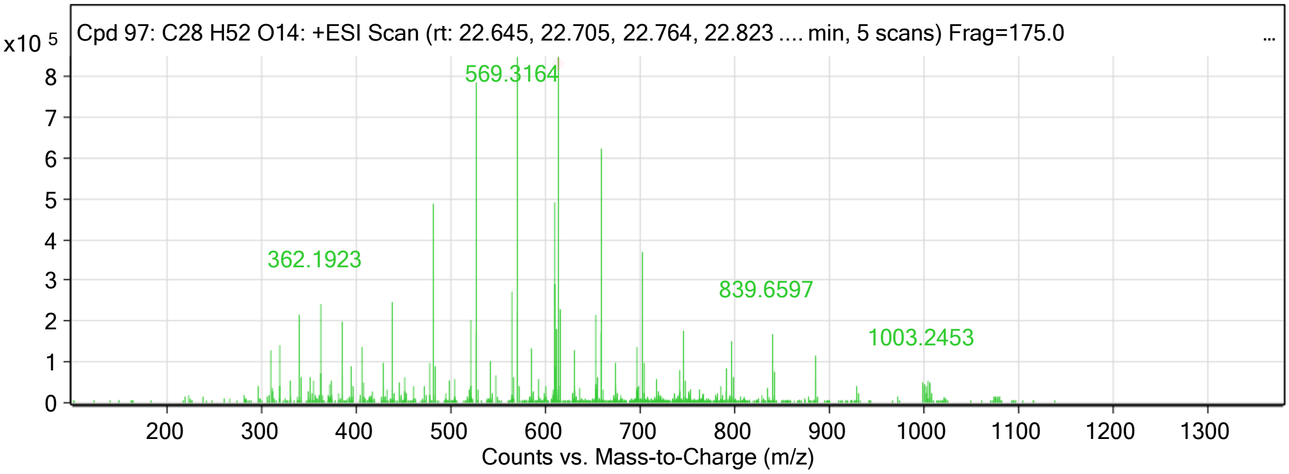

MS Zoomed Spectrum

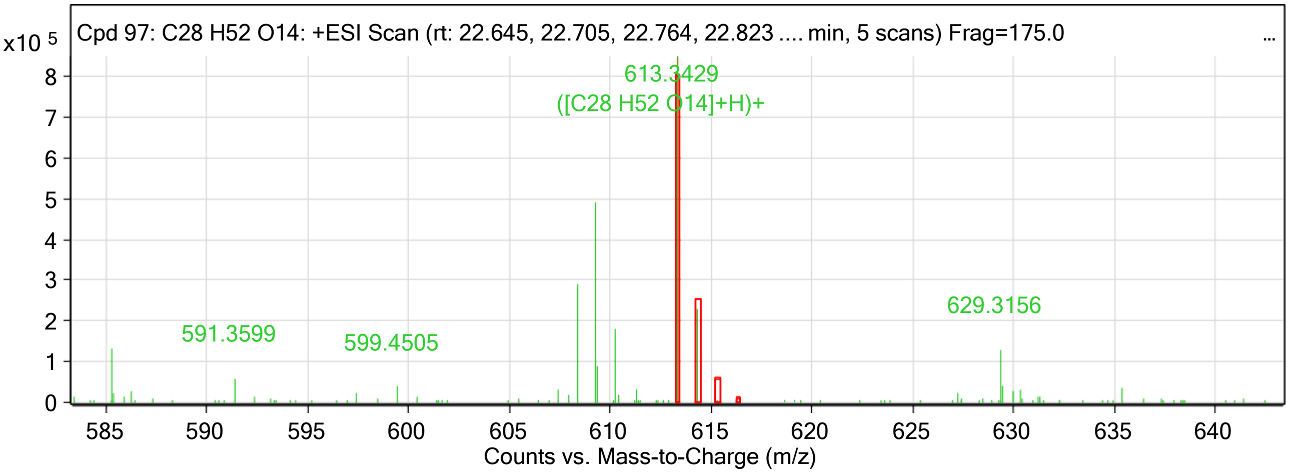

MS Spectrum Peak List

m/z	Calc *m/z*	Diff(ppm)	z	Abund	Formula	Ion
481.2632			1	485446.81		
525.2902			1	784033.25		
569.3164			1	958102.31		
609.2725			1	488988.25		
613.3429	613.343	0.11	1	848836	C28 H52 O14	(M+H)+
614.3454	614.3464	1.63	1	229507.75	C28 H52 O14	(M+H)+
615.3474	615.3489	2.38	1	41096.92	C28 H52 O14	(M+H)+
616.3546	616.3515	-5.03	1	5609.77	C28 H52 O14	(M+H)+
657.369			1	621833.5		

MSMS Spectrum

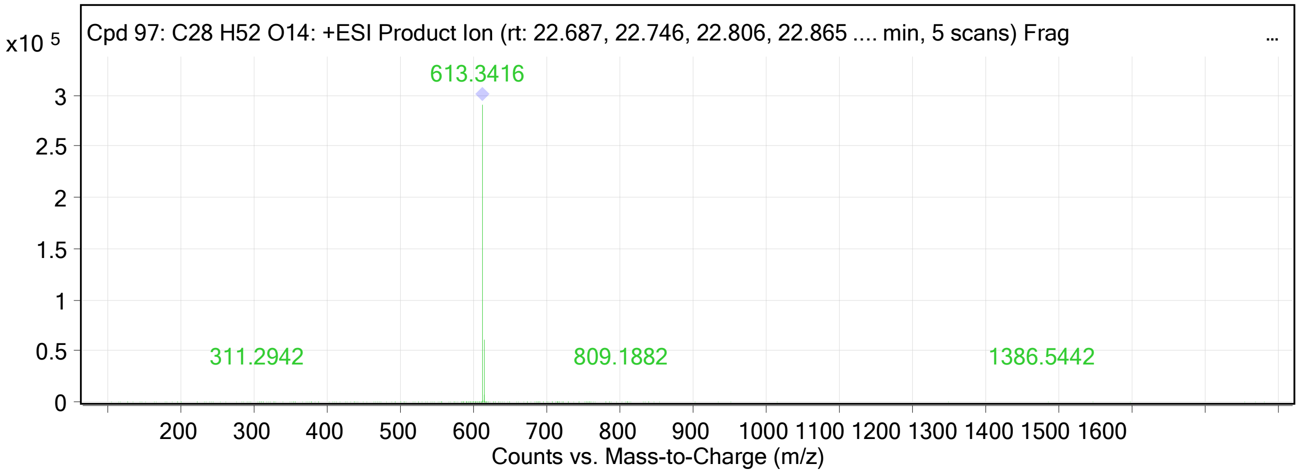

MS/MS Spectrum Peak List

m/z	*Calc m/z*	Diff(ppm)	z	Abund
133.0864	133.0859	-3.41		22.74
309.2791	309.2788	-0.96	1	56.34
357.0683	357.0664	-5.43		23.23
612.3871			2	1844.39
612.6385			2	2283.4
612.8883			2	1780.79
613.3416	613.343	2.28	1	291127
614.3444			1	60440.22
615.3468			1	7321.81
615.417			2	1338.46

Compound Label	*m/z*	RT	Algorithm	Mass
Cpd 98: C24 H46 N3 O12	569.3164	22.907	Auto MS/MS	568.3091

Compound Chromatograms

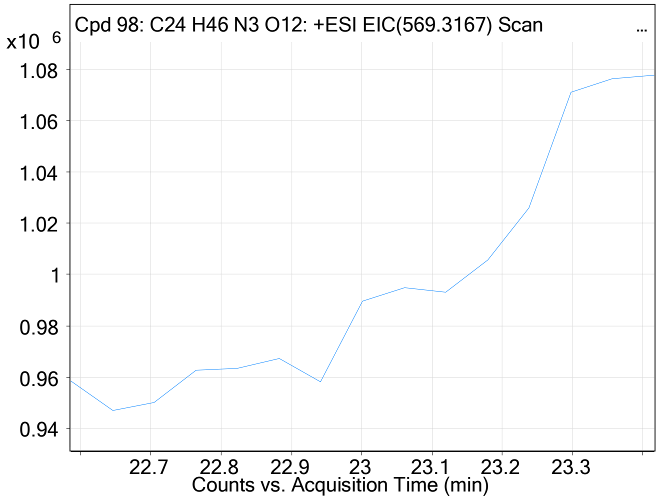

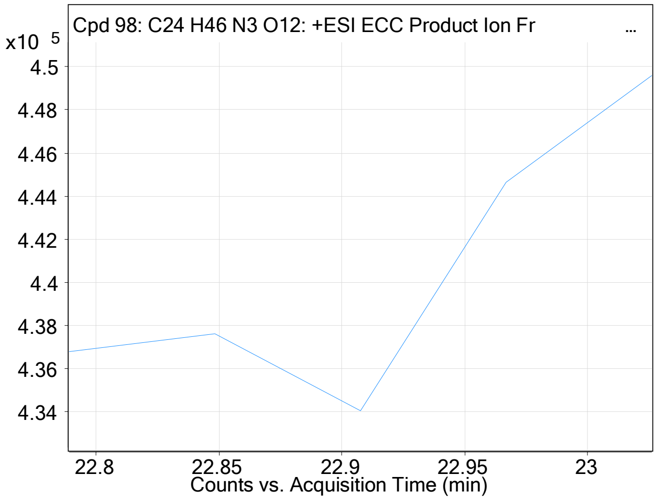

MS Spectrum

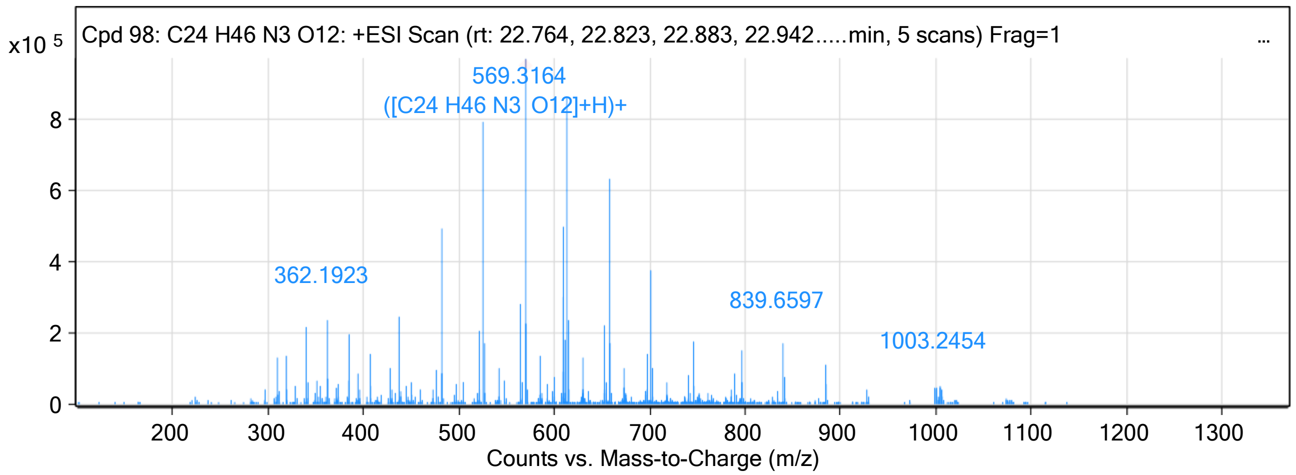

MS Zoomed Spectrum

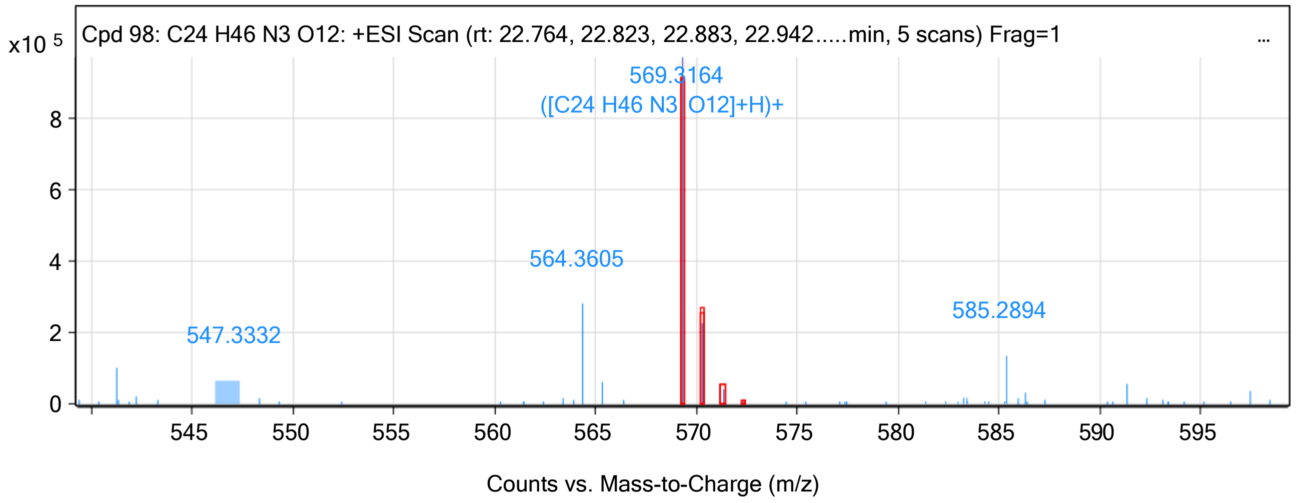

MS Spectrum Peak List

m/z	Calc *m/z*	Diff(ppm)	z	Abund	Formula	Ion
481.2633			1	488323.66		
525.2902			1	790957.81		
569.3164	569.3154	-1.79	1	968311.81	C24 H46 N3 O12	(M+H)+
570.3192	570.3186	-0.97	1	226846.58	C24 H46 N3 O12	(M+H)+
571.3212	571.3209	-0.46	1	38698.39	C24 H46 N3 O12	(M+H)+
572.3239	572.3235	-0.66	1	5489.7	C24 H46 N3 O12	(M+H)+
609.2726			1	492113.16		
613.3429			1	856787.19		
657.369			1	627767		
701.395			1	373394.44		

MSMS Spectrum

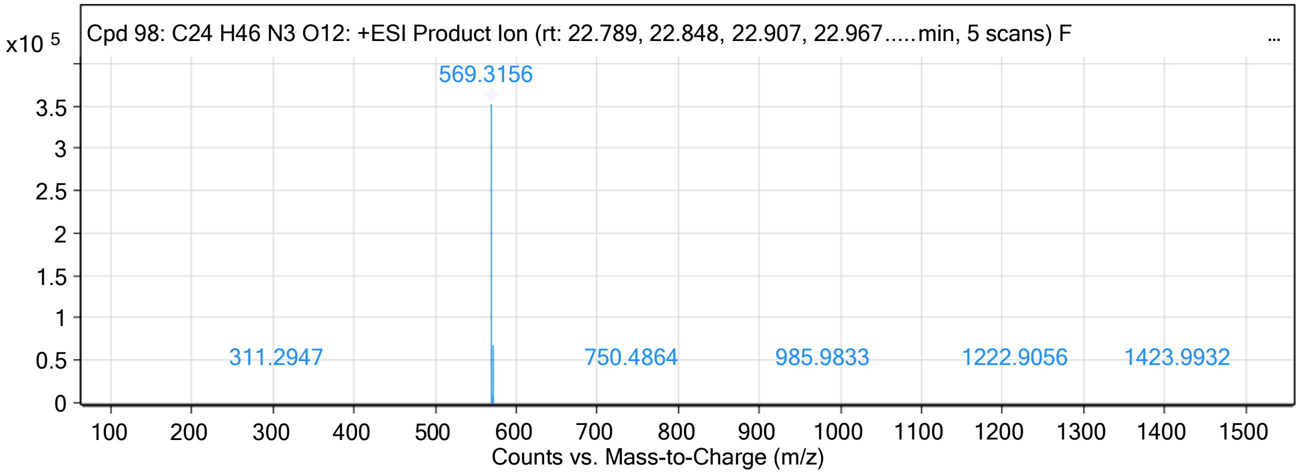

MS/MS Spectrum Peak List

m/z	*Calc m/z*	Diff(ppm)	z	Abund
133.0869	133.0859	-7.72		38.63
195.1227	195.1214	-7.04		32.82
311.2947	311.2945	-0.73		71.95
566.2843	283.1457	-499993.74	2	42.4
568.3611			2	789.61
568.6114			2	735.24
568.8625			2	737.53
569.3156	569.3154	-0.23	1	351660.06
570.3181			1	68387
571.3207			1	8418.54

Compound Label	*m/z*	RT	Algorithm	Mass
Cpd 99: C28 H52 O14	613.3431	23.102	Auto MS/MS	612.3356

Compound Chromatograms

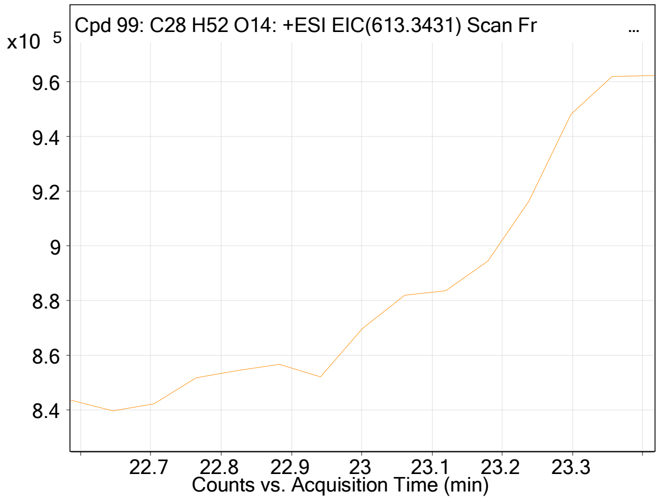

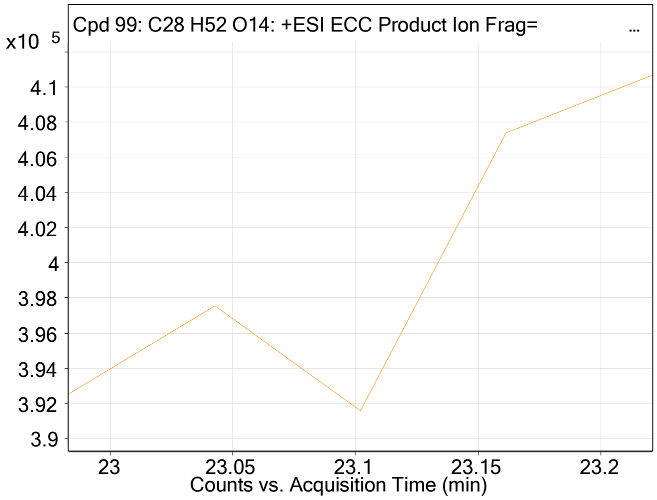

MS Spectrum

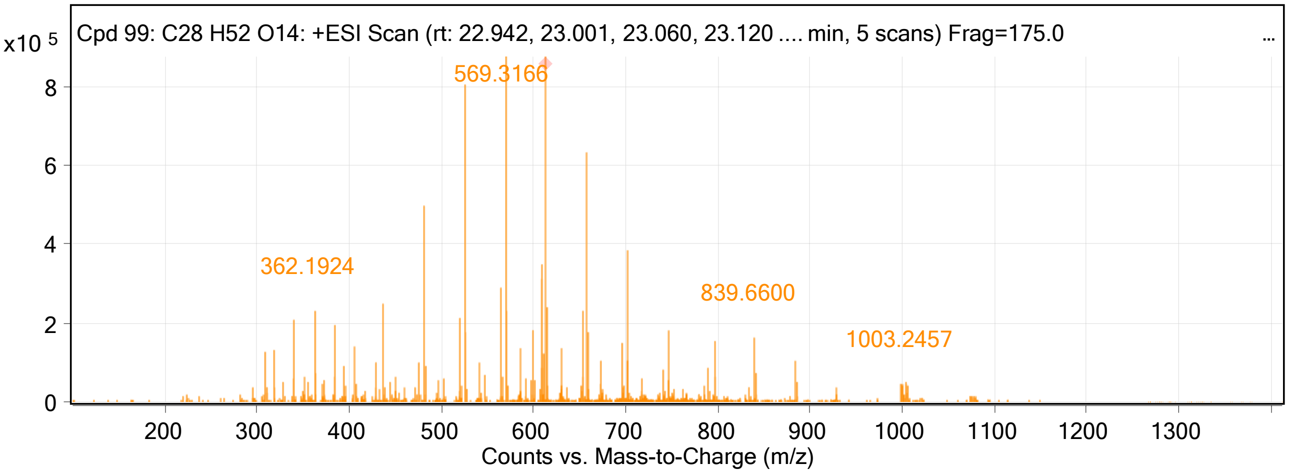

MS Zoomed Spectrum

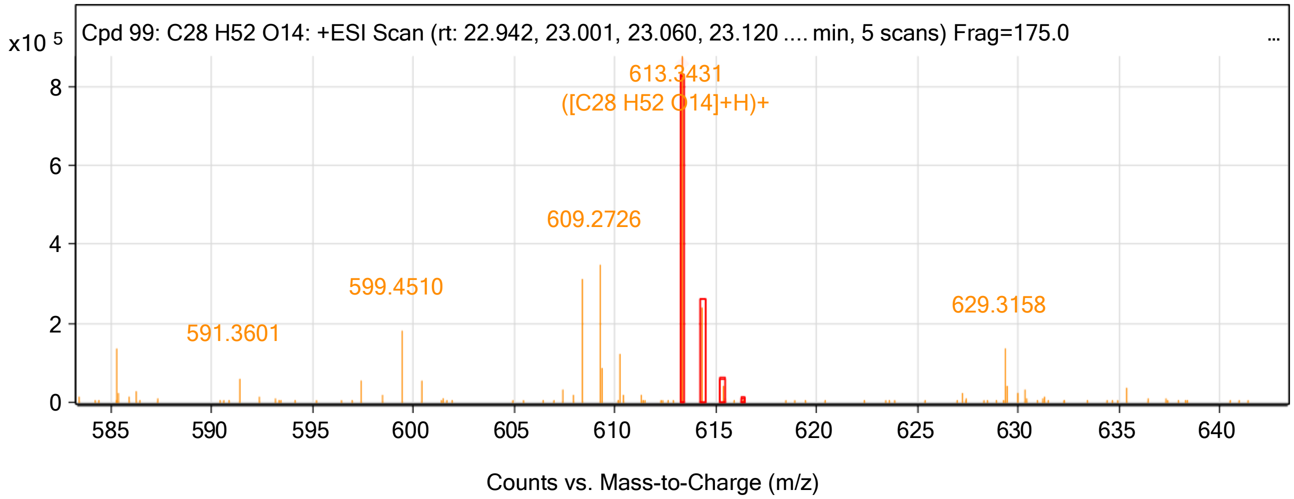

MS Spectrum Peak List

m/z	Calc *m/z*	Diff(ppm)	z	Abund	Formula	Ion
481.2634			1	496112.75		
525.2904			1	805220.19		
569.3166			1	988407		
609.2726			1	346863.41		
613.3431	613.343	-0.26	1	876264.69	C28 H52 O14	(M+H)+
614.3457	614.3464	1.17	1	238717.63	C28 H52 O14	(M+H)+
615.3478	615.3489	1.8	1	42226.77	C28 H52 O14	(M+H)+
616.3527	616.3515	-1.92	1	5883.56	C28 H52 O14	(M+H)+
657.3693			1	631444.63		
701.3952			1	383830.31		

MSMS Spectrum

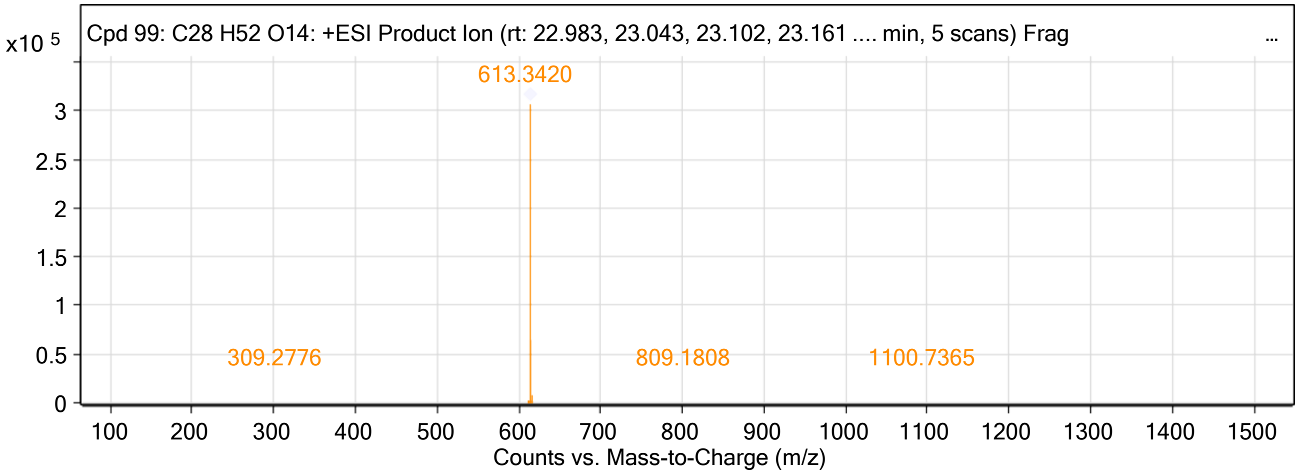

MS/MS Spectrum Peak List

m/z	Calc *m/z*	Diff(ppm)	z	Abund
133.0862	133.0859	-2.24		46.74
177.1127	177.1121	-3.05		18.88
283.2635	283.2632	-1.23		28.6
309.2776	309.2788	3.77	1	97.84
612.6382			2	2177.19
612.8897			2	1749.86
613.342	613.343	1.62	1	306716.06
614.3447			1	63915.05
615.347			1	7511.7
615.42			2	2216.45

Compound Label	*m/z*	RT	Algorithm	Mass
Cpd 100: C24 H46 N3 O12	569.3166	23.204	Auto MS/MS	568.3092

Compound Chromatograms

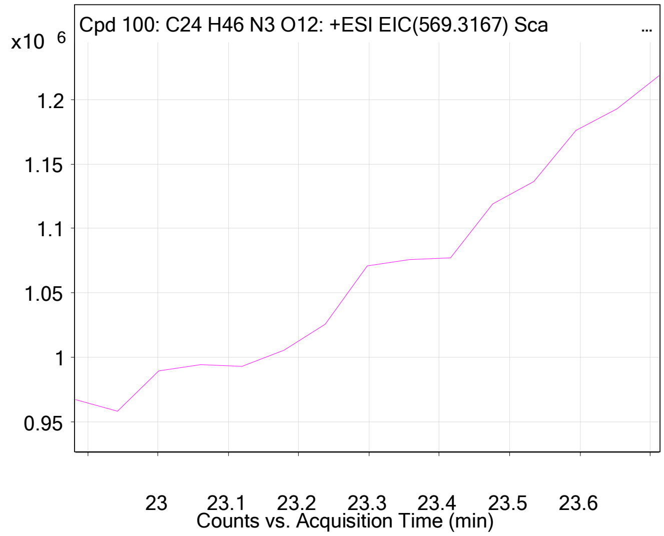

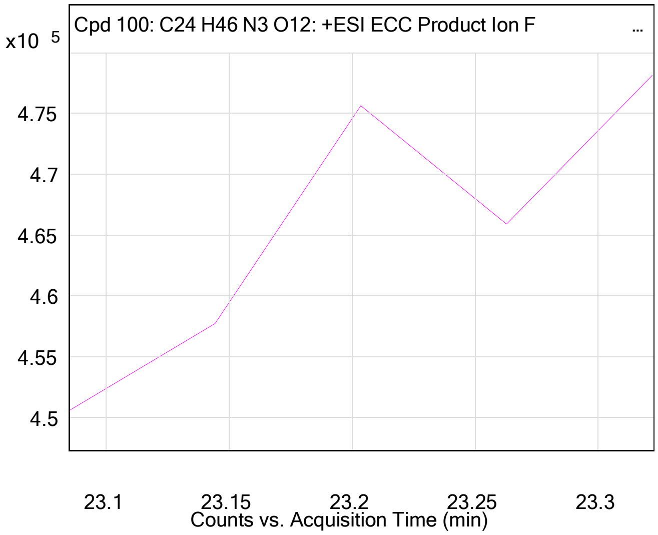

MS Spectrum

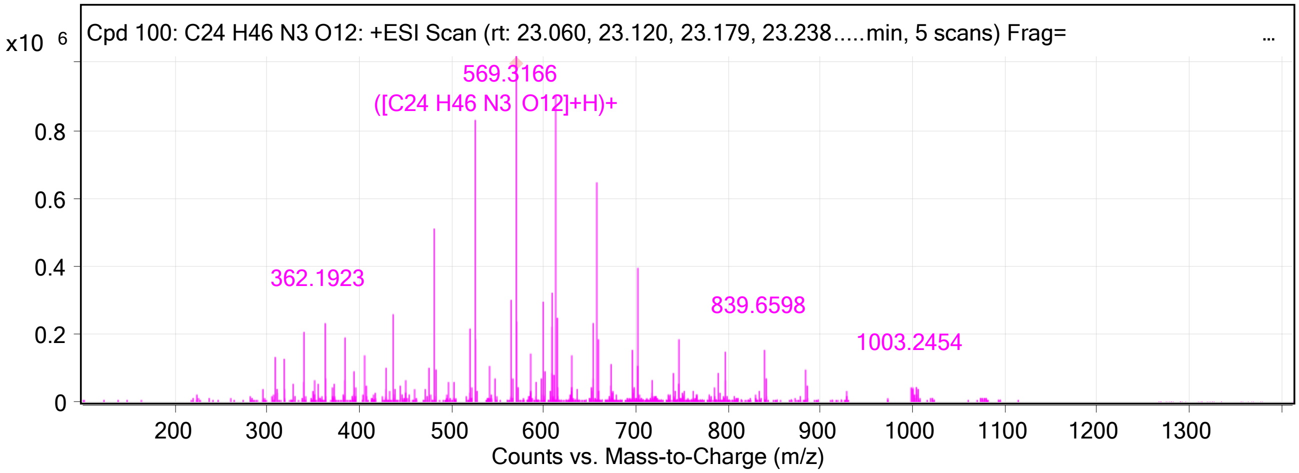

MS Zoomed Spectrum

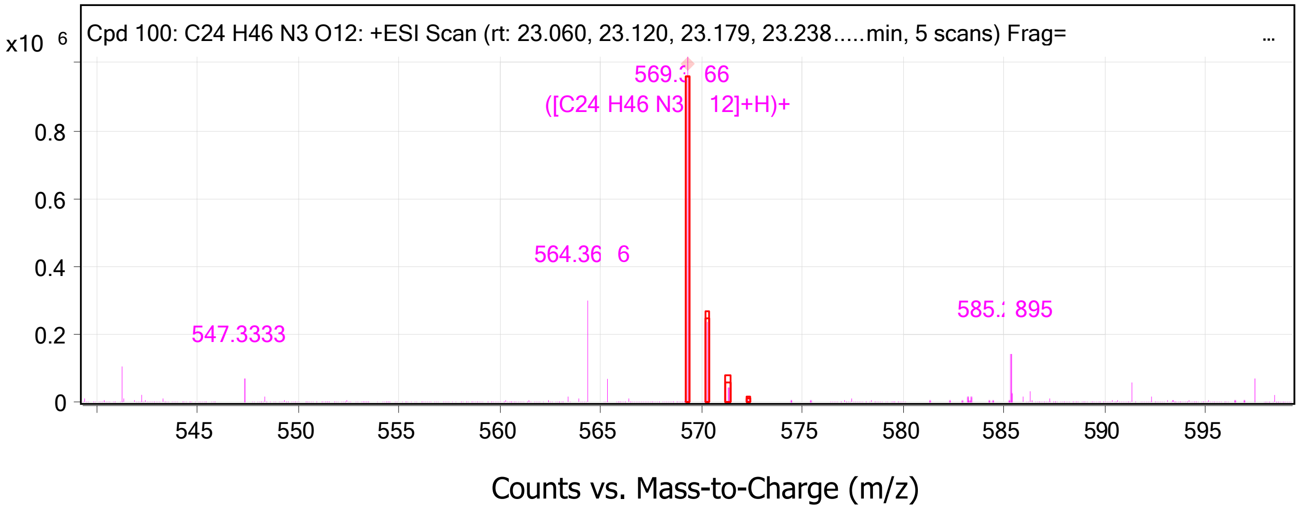

MS Spectrum Peak List

m/z	Calc *m/z*	Diff(ppm)	z	Abund	Formula	Ion
481.2634			1	511095.25		
525.2903			1	827998.94		
569.3166	569.3154	-1.98	1	1018169.81	C24 H46 N3 O12	(M+H)+
570.3192	570.3186	-1.05	1	235847.03	C24 H46 N3 O12	(M+H)+
571.3213	571.3209	-0.66	1	40767.01	C24 H46 N3 O12	(M+H)+
572.324	572.3235	-0.85	1	5589.57	C24 H46 N3 O12	(M+H)+
608.387			1	318837.56		

MSMS Spectrum

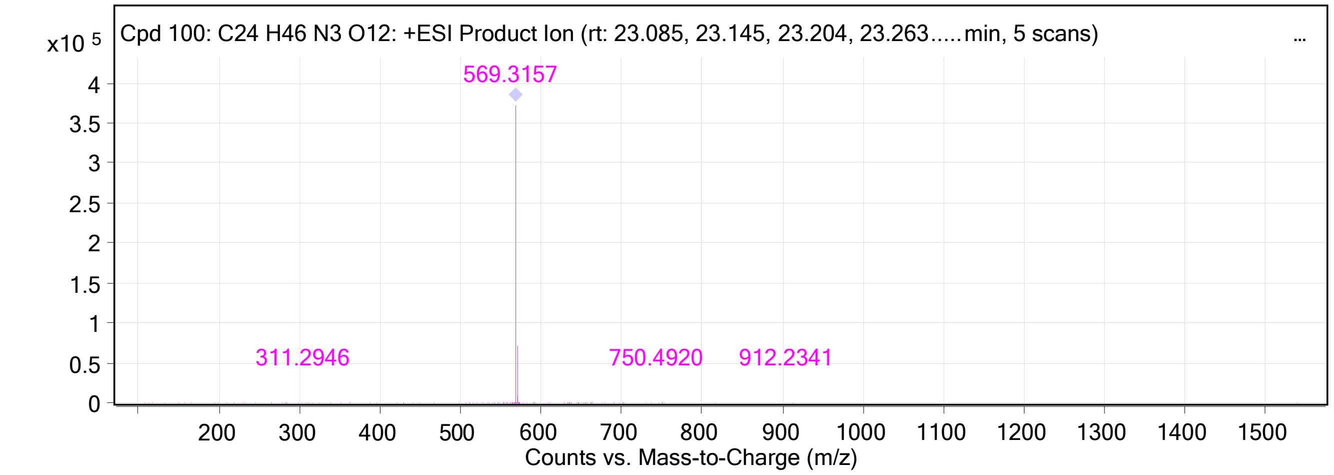

MS/MS Spectrum Peak List

m/z	*Calc m/z*	Diff(ppm)	z	Abund
133.0857	133.0859	1.53		25.66
232.1283	232.1292	3.79		21.99
283.1766	283.1778	4.25		15.16
283.2627	283.2632	1.68		21.41
307.2219	307.2227	2.9		13.14
309.2801	309.2788	-4.26		30.51
311.2946	311.2945	-0.38		54.59
337.2355	337.236	1.34		17.16
569.3157	569.3154	-0.5	1	372855.81
570.3183			1	72597.39

Compound Label	*m/z*	RT	Algorithm	Mass
Cpd 101: C28 H52 O14	613.3432	23.398	Auto MS/MS	612.3357

Compound Chromatograms

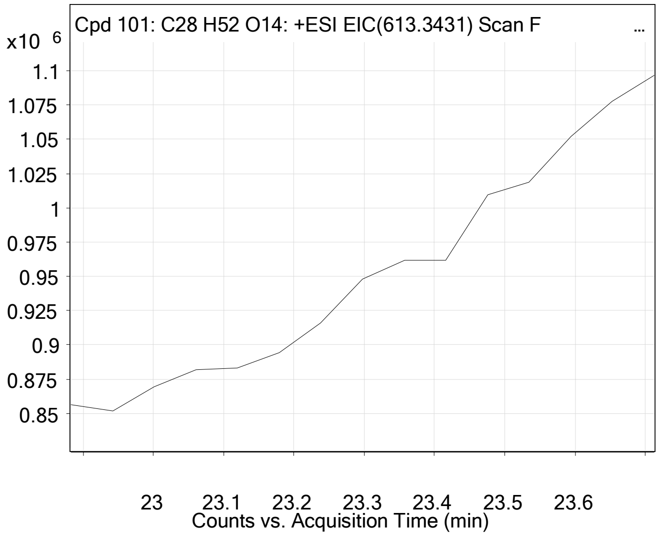

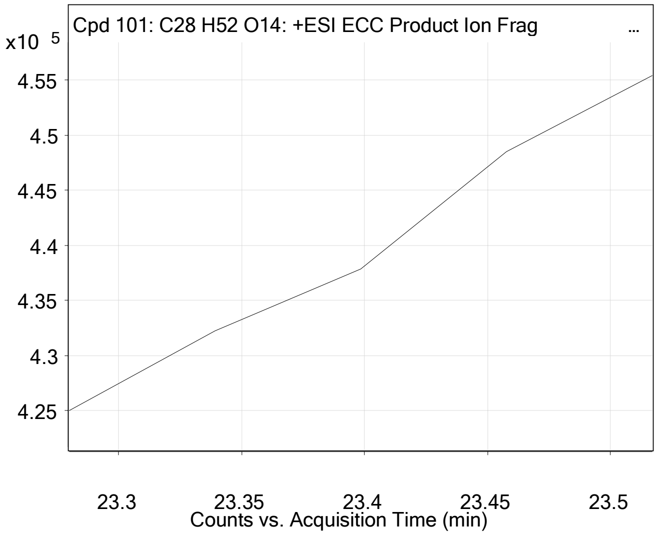

MS Spectrum

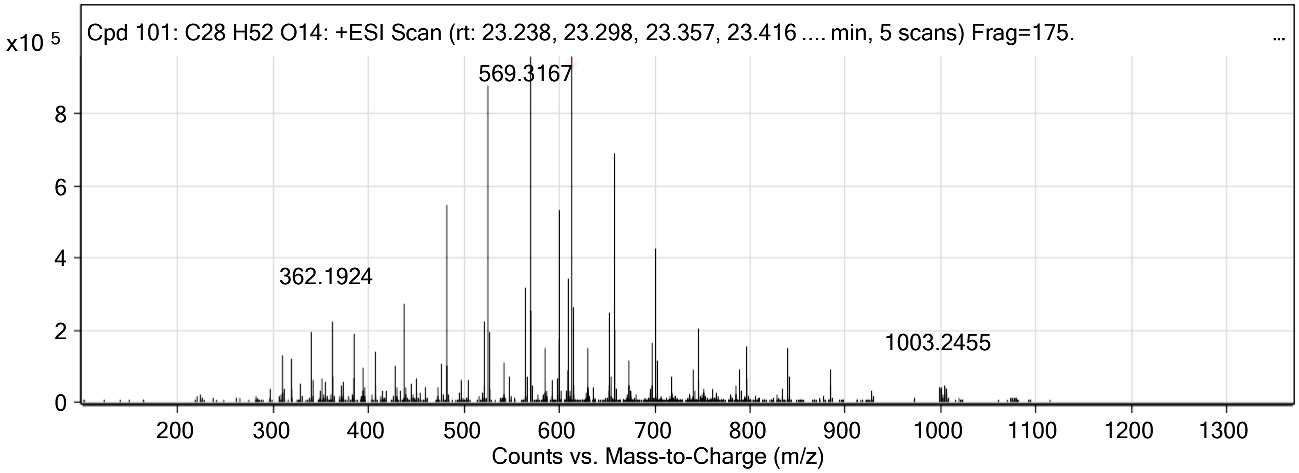

MS Zoomed Spectrum

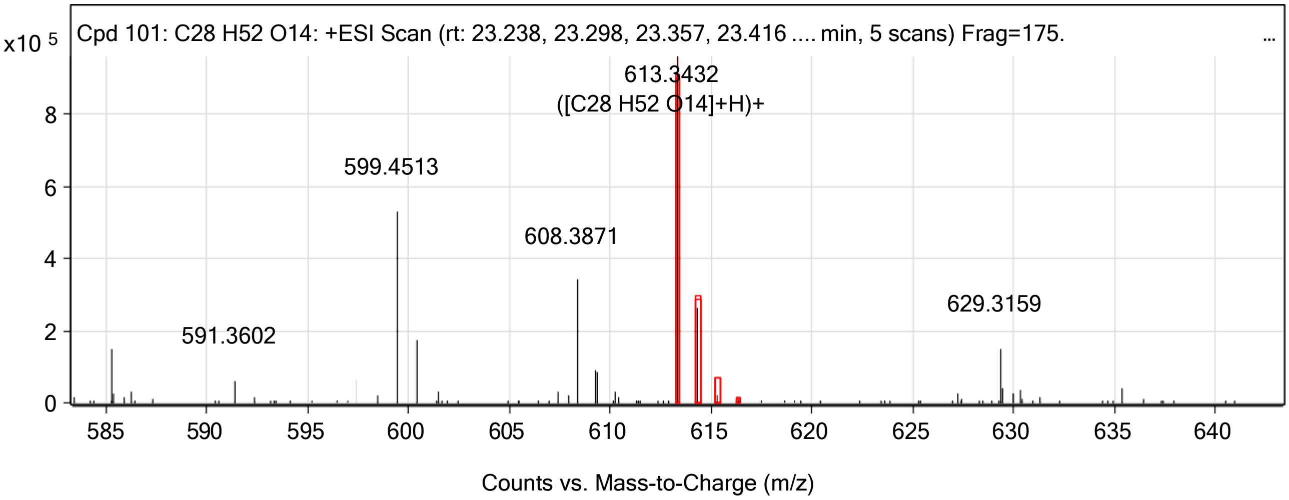

MS Spectrum Peak List

m/z	Calc *m/z*	Diff(ppm)	z	Abund	Formula	Ion
481.2635			1	544845.5		
525.2904			1	875429.81		
569.3167			1	1074035.63		
599.4513			1	528271.88		
613.3432	613.343	-0.31	1	959554.19	C28 H52 O14	(M+H)+
614.3458	614.3464	1.05	1	264389.44	C28 H52 O14	(M+H)+
615.3477	615.3489	1.94	1	46233.35	C28 H52 O14	(M+H)+
616.3521	616.3515	-0.92	1	6540.59	C28 H52 O14	(M+H)+
657.3696			1	686650.44		
701.3953			1	424762.81		

MSMS Spectrum

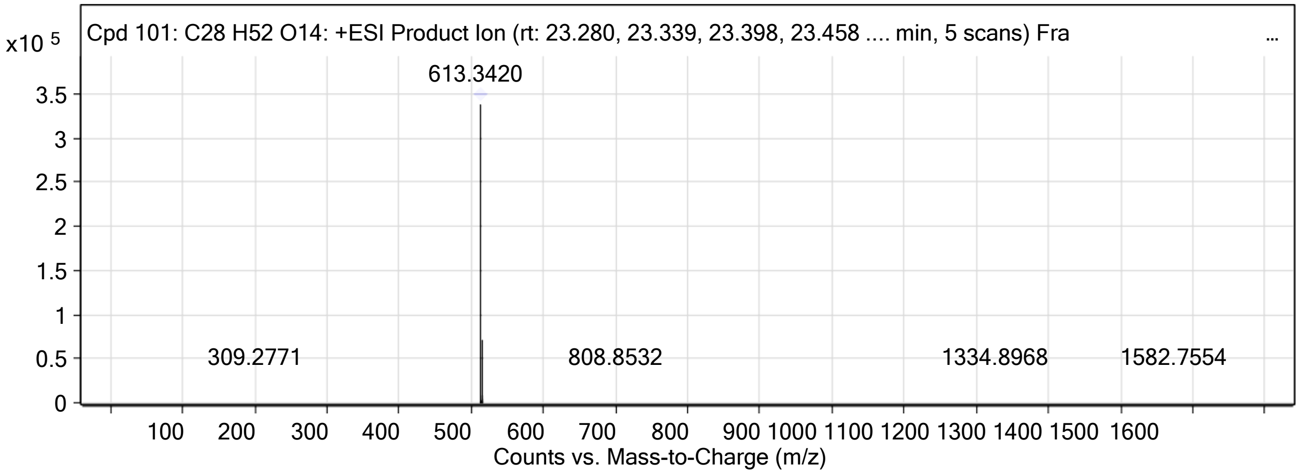

MS/MS Spectrum Peak List

m/z	*Calc m/z*	Diff(ppm)	z	Abund
133.0858	133.0859	0.59		26.51
309.2771	309.2788	5.53		62.21
311.2949	311.2945	-1.43	1	50.48
609.3105	304.6556	-499999.48	2	36.99
612.638			2	2056.73
612.8888			2	1530.2
613.342	613.343	1.55	1	338187.56
614.3447			1	70925.89
615.3469			1	8607.74
615.4218			1	4116.97

Compound Label	*m/z*	RT	Algorithm	Mass
Cpd 102: C26 H48 O13	569.3171	23.5	Auto MS/MS	568.3097

Compound Chromatograms

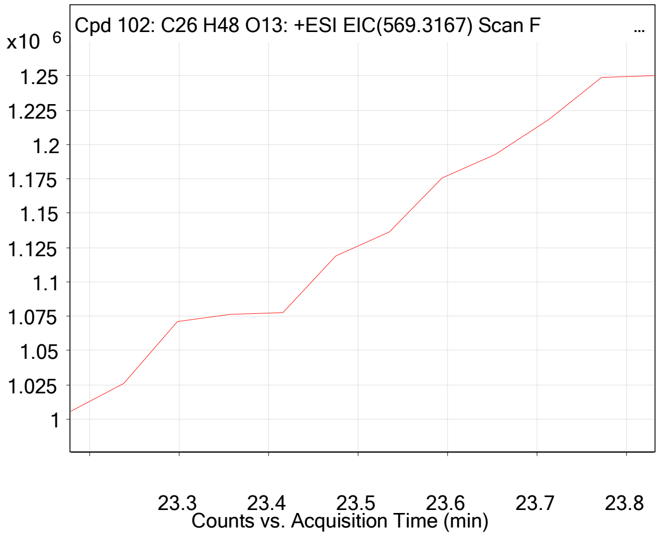

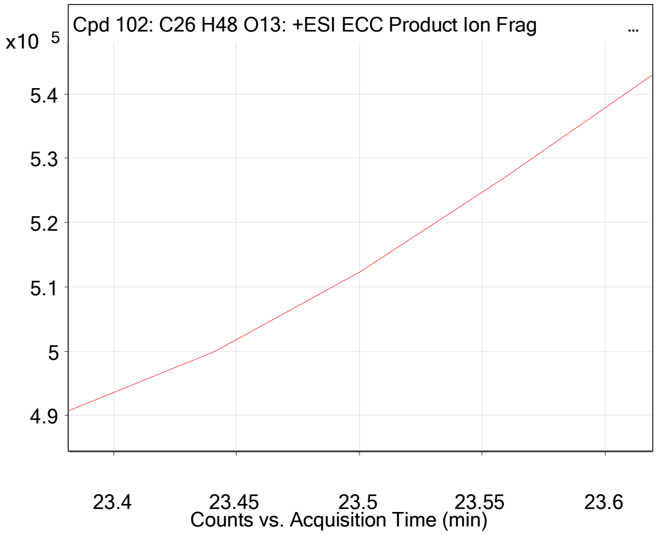

MS Spectrum

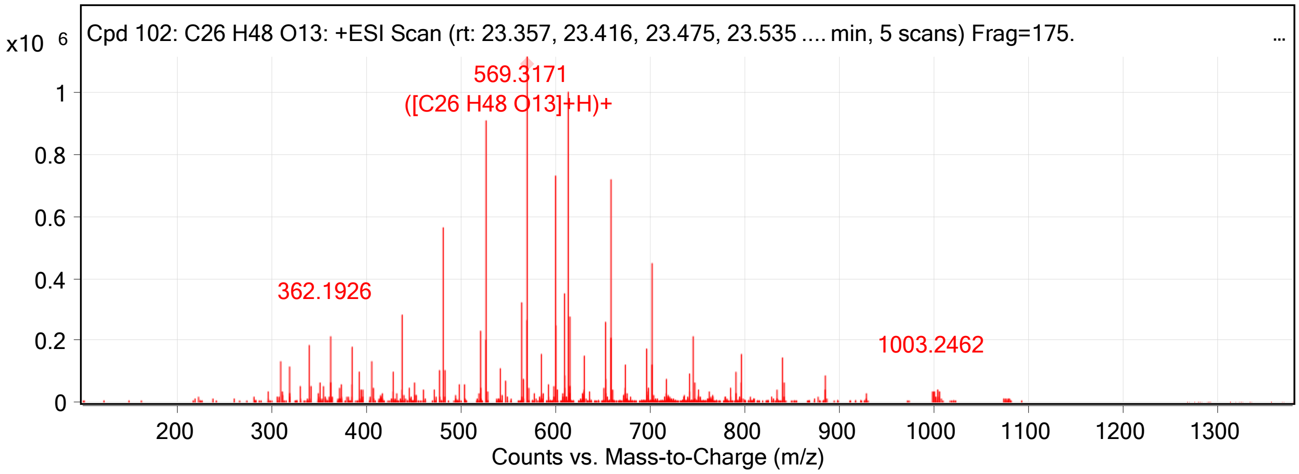

MS Zoomed Spectrum

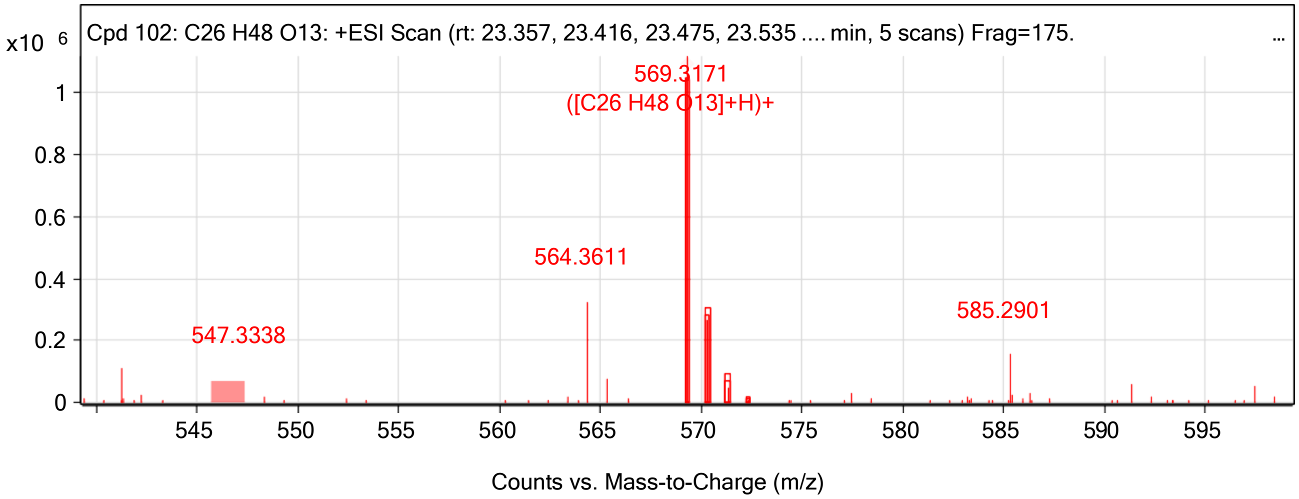

MS Spectrum Peak List

m/z	Calc *m/z*	Diff(ppm)	z	Abund	Formula	Ion
481.2638			1	567055.19		
525.2908			1	909620.69		
569.3171	569.3168	-0.61	1	1117186.25	C26 H48 O13	(M+H)+
570.3198	570.3202	0.77	1	266097.56	C26 H48 O13	(M+H)+
571.3218	571.3226	1.39	1	45469.69	C26 H48 O13	(M+H)+
572.3244	572.3253	1.48	1	6041.84	C26 H48 O13	(M+H)+
599.4521			1	729352.38		
613.3436			1	1001034.63		
657.3701			1	719228.38		
701.3958			1	448247.31		

MSMS Spectrum

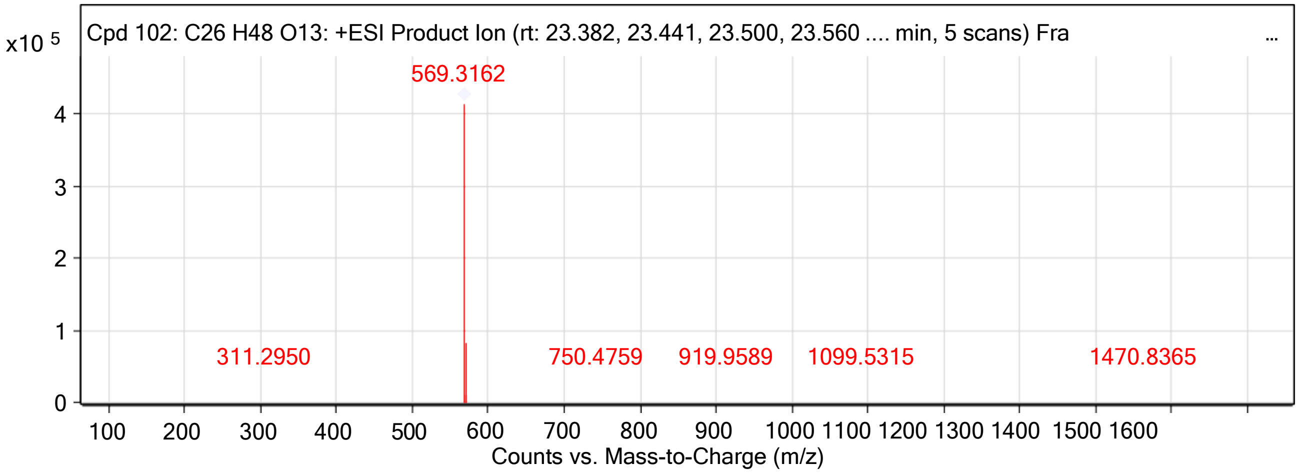

MS/MS Spectrum Peak List

m/z	Calc *m/z*	Diff(ppm)	z	Abund
133.0868	133.0859	-6.42		36.74
144.0795	144.0781	-9.92		12.87
221.1352	221.1384	14.24		11.82
307.2226	307.2268	13.71		13.42
309.2772	309.2788	5.12		35.01
311.295	311.2945	-1.76		54.09
524.3145	524.3191	8.84		13.1
569.3162	569.3168	0.99	1	412894.56
570.3188			1	81381.33
571.321			1	9714.04

Compound Label	*m/z*	RT	Algorithm	Mass
Cpd 103: C28 H52 O14	613.3439	23.606	Auto MS/MS	612.3364

Compound Chromatograms

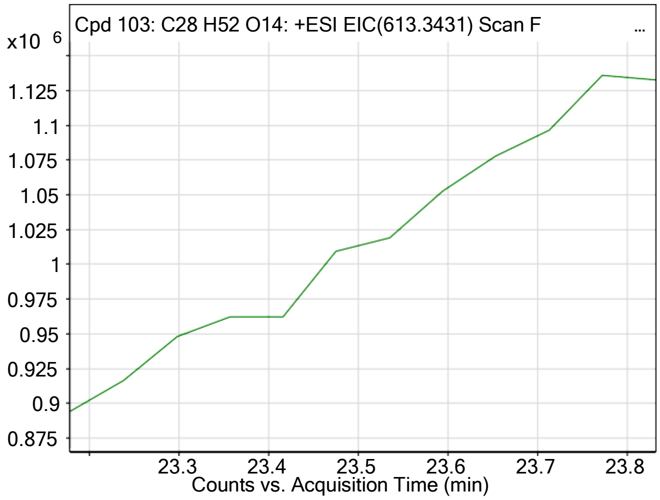

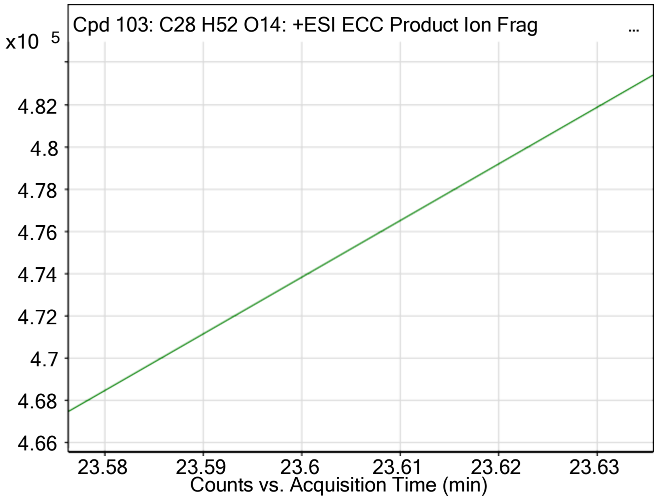

MS Spectrum

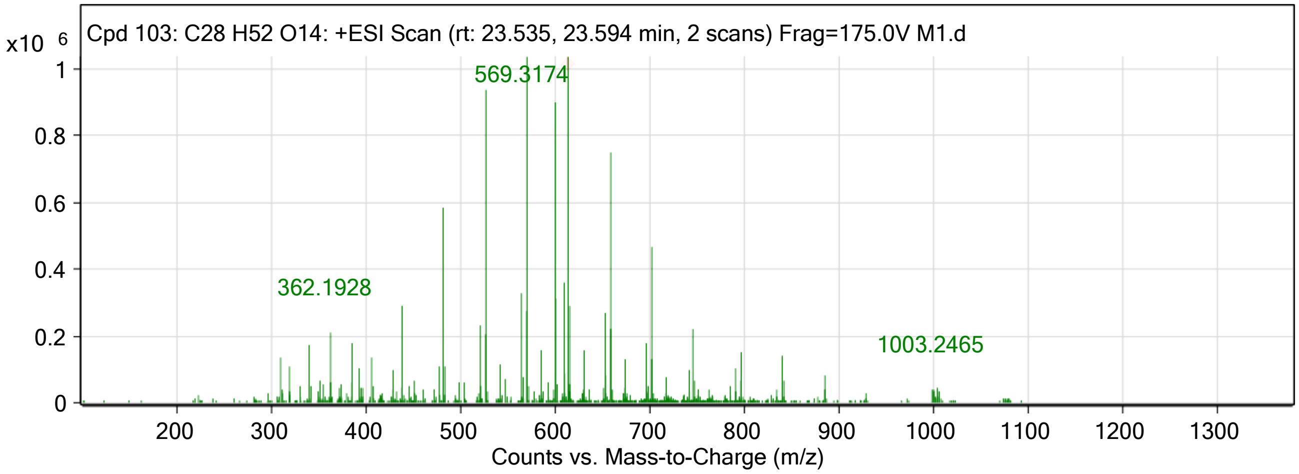

MS Zoomed Spectrum

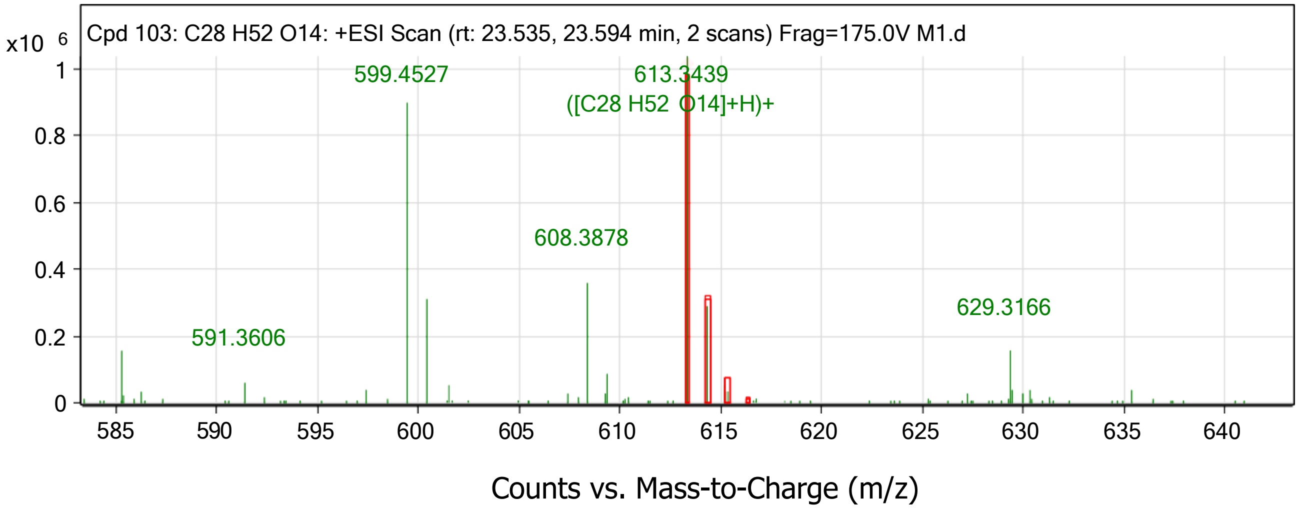

MS Spectrum Peak List

m/z	Calc *m/z*	Diff(ppm)	z	Abund	Formula	Ion
481.264			1	582307.13		
525.291			1	937137.38		
569.3174			1	1156344.13		
599.4527			1	897568.38		
613.3439	613.343	-1.43	1	1035820.31	C28 H52 O14	(M+H)+
614.3464	614.3464	-0.04	1	287938.81	C28 H52 O14	(M+H)+
615.3483	615.3489	0.84	1	51901.53	C28 H52 O14	(M+H)+
616.3524	616.3515	-1.36	1	7134.15	C28 H52 O14	(M+H)+
657.3704			1	747197.56		

MSMS Spectrum

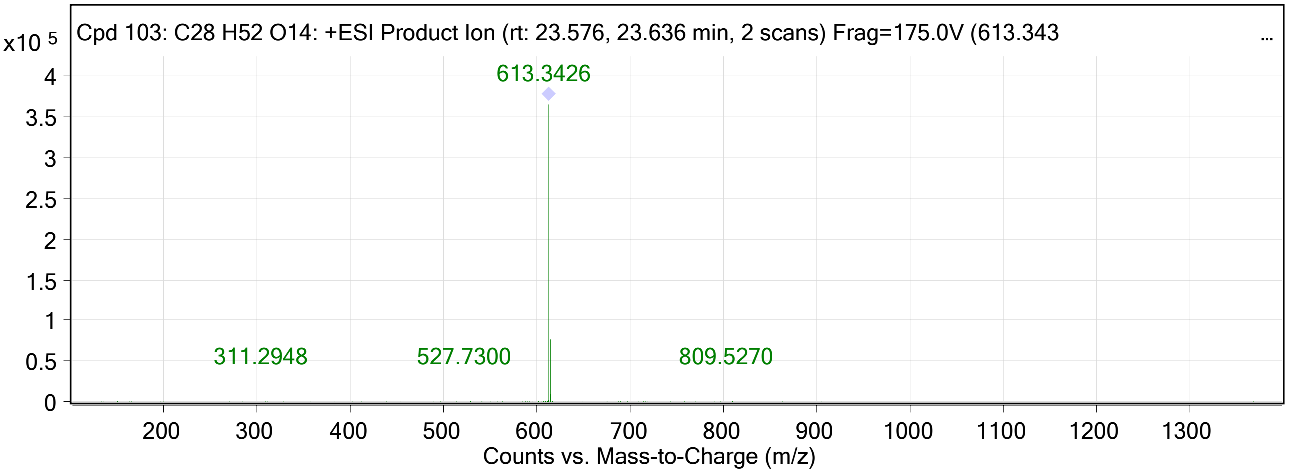

MS/MS Spectrum Peak List

m/z	*Calc m/z*	Diff(ppm)	z	Abund
133.0848	133.0859	8.5		49.9
166.0896	166.0836	-36.54		39.06
309.2779	309.2788	2.87		55.92
311.2948	311.2945	-1.07		105.16
600.3332	600.3352	3.3		38.5
612.3877			2	1590.68
613.3426	613.343	0.64	1	366254.19
614.3454			1	77266.01
615.3474			1	9642.66
615.4231			1	5856.19

Compound Label	*m/z*	RT	Algorithm	Mass
Cpd 104: C26 H48 O13	569.3169	23.776	Auto MS/MS	568.3094

Compound Chromatograms

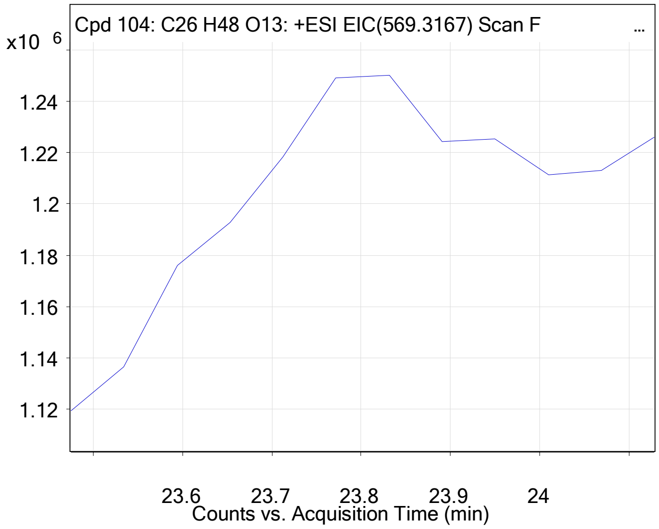

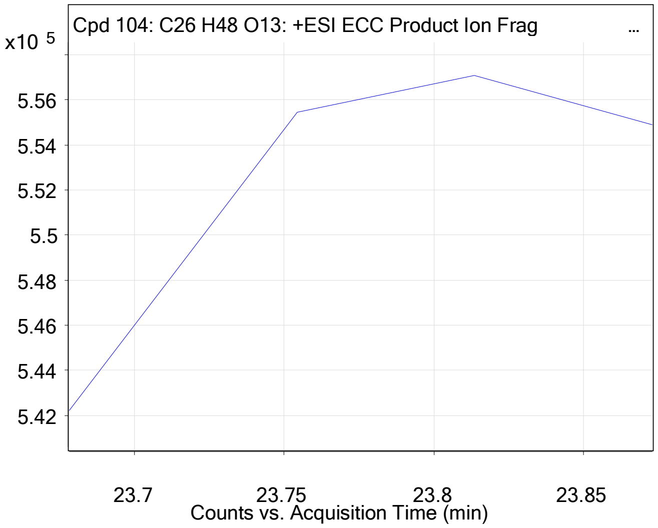

MS Spectrum

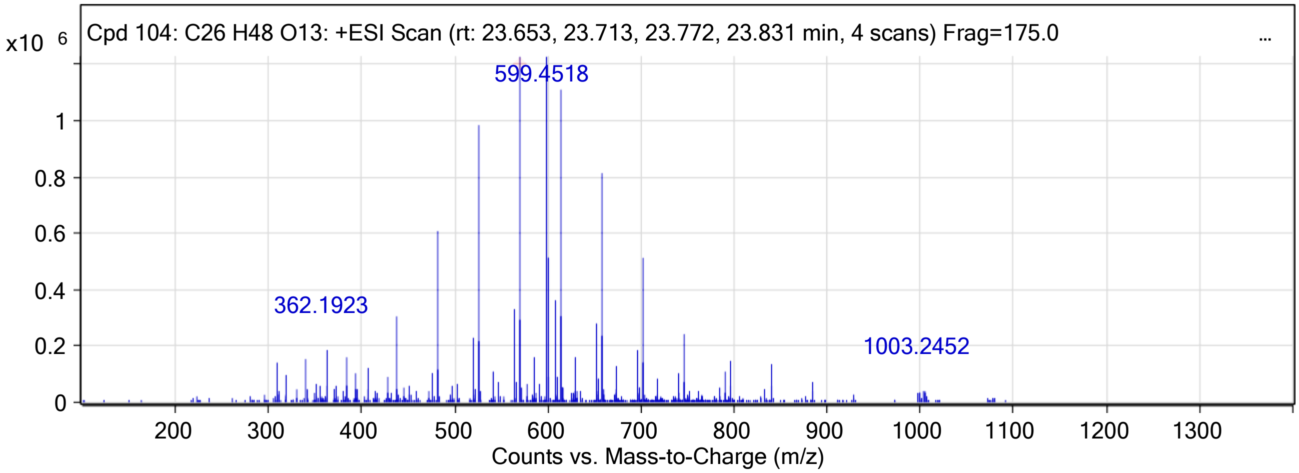

MS Zoomed Spectrum

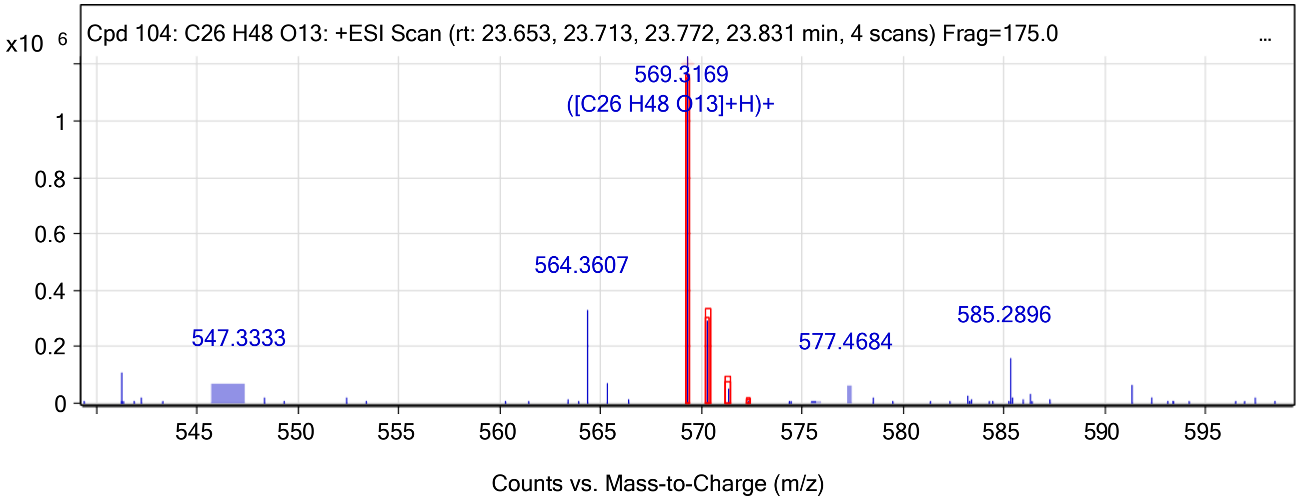

MS Spectrum Peak List

m/z	Calc *m/z*	Diff(ppm)	z	Abund	Formula	Ion
481.2635			1	608362.13		
525.2904			1	984300.31		
569.3169	569.3168	-0.25	1	1227655.13	C26 H48 O13	(M+H)+
570.3194	570.3202	1.44	1	293305.69	C26 H48 O13	(M+H)+
571.3213	571.3226	2.3	1	49291.32	C26 H48 O13	(M+H)+
572.3238	572.3253	2.57	1	6827.15	C26 H48 O13	(M+H)+
599.4518			1	1439994.5		
600.4549			1	511647.69		
613.3431			1	1110704.75		
657.3698			1	807785.19		

MSMS Spectrum

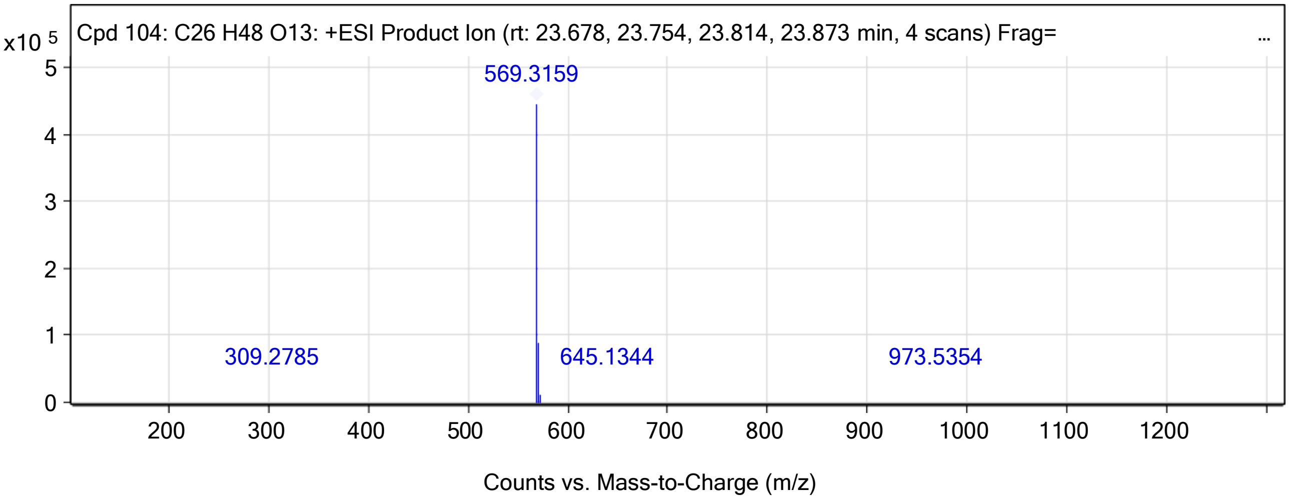

MS/MS Spectrum Peak List

m/z	*Calc m/z*	Diff(ppm)	z	Abund
133.0866	133.0859	-4.73		30.57
144.0755	144.0781	18.02		25.8
177.1112	177.1121	5.18		24.66
279.2311	279.2319	2.58		19.01
309.2785	309.2788	0.85		35.99
311.2947	311.2945	-0.72		25.76
318.2441	318.2401	-12.57		29.43
426.2838	426.2823	-3.4		18.49
566.2956	566.2933	-4.02	1	43.27
569.3159	569.3168	1.59	1	444493.88

Compound Label	Name	*m/z*	RT	Algorithm	Mass
Cpd 105: 1-(O-alpha-D-glucopyranosyl)-(1,3R,25R)-hexacosanetriol	**1-(O-alpha-D-glucopyranosyl)-(1,3R,25R)-hexacosanetriol**	599.4517	23.805	Auto MS/MS	576.4623

Compound Chromatograms

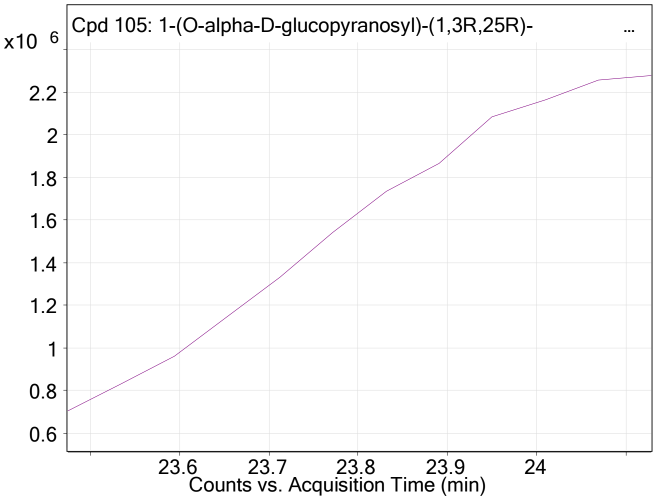

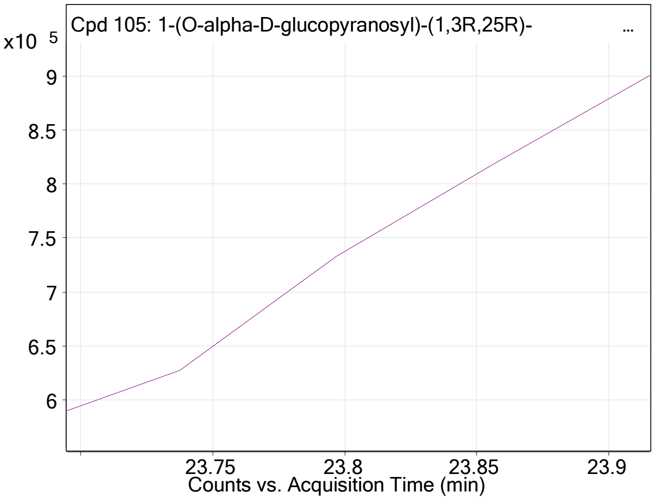

MS Spectrum

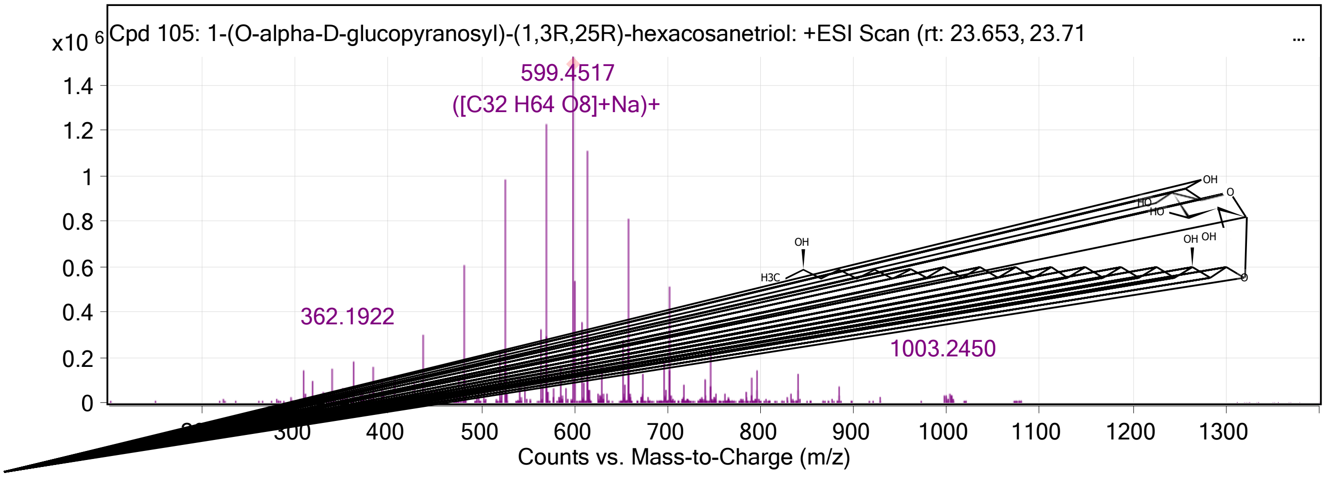

MS Zoomed Spectrum

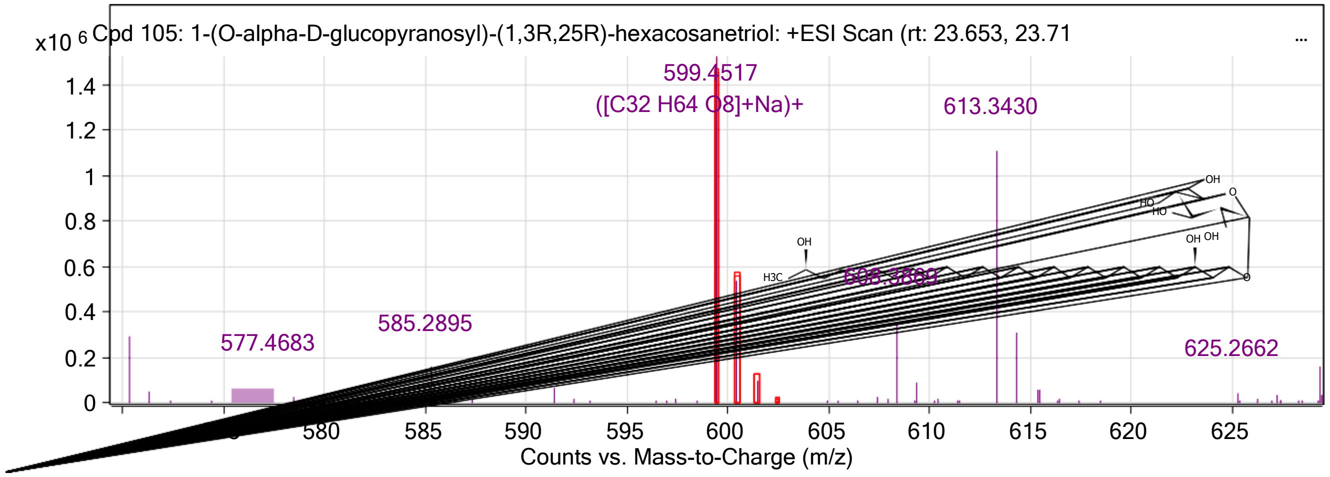

MS Spectrum Peak List

m/z	Calc *m/z*	Diff(ppm)	z	Abund	Formula	Ion
481.2634			1	607703		
525.2903			1	983993.69		
569.3168			1	1227019.25		
599.4517	599.4493	-3.9	1	1525543.38	C32 H64 O8	(M+Na)+
600.4548	600.4528	-3.44	1	538817.63	C32 H64 O8	(M+Na)+
601.4569	601.4556	-2.04	1	94558.28	C32 H64 O8	(M+Na)+
602.4591	602.4584	-1.05	1	11598.64	C32 H64 O8	(M+Na)+
613.343			1	1112483.5		
657.3697			1	808548.56		
701.3951			1	510865.91		

MSMS Spectrum

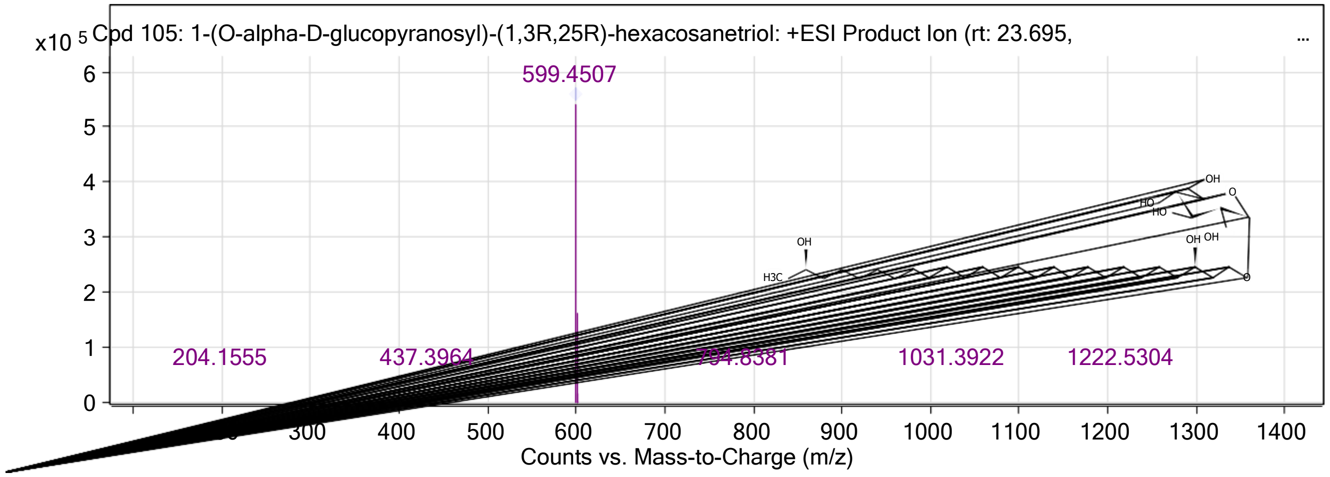

MS/MS Spectrum Peak List

m/z	Calc *m/z*	Diff(ppm)	z	Abund
133.0858	133.0859	0.78		43.81
437.3964	437.3989	5.65	1	45.34
596.4369	596.4283	-14.52	1	82.17
598.4338			1	1181.01
599.4507	599.4517	1.71	1	540810.13
600.3798				1046.93
600.4538			1	161387.34
601.3829			2	1331.04
601.4559			1	16905.85
601.6333			2	1025.77

Compound Structure

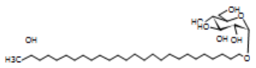

Compound Label	*m/z*	RT	Algorithm	Mass
Cpd 106: C26 H48 O13	569.3169	24.051	Auto MS/MS	568.3094

Compound Chromatograms

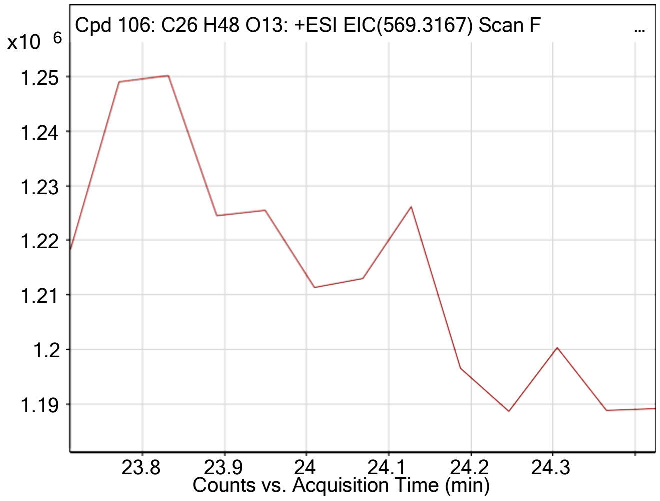

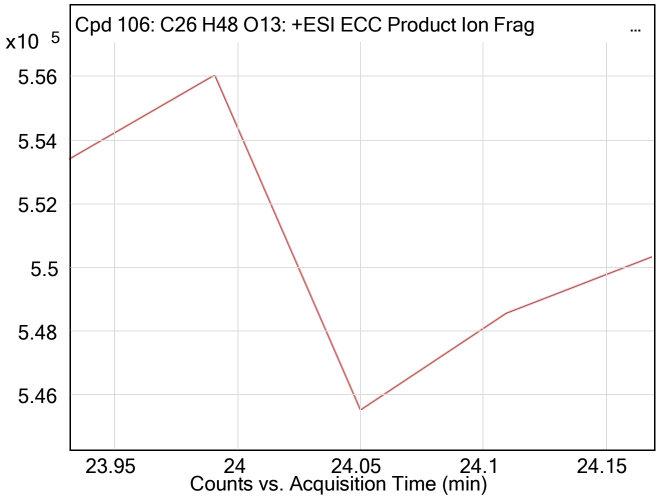

MS Spectrum

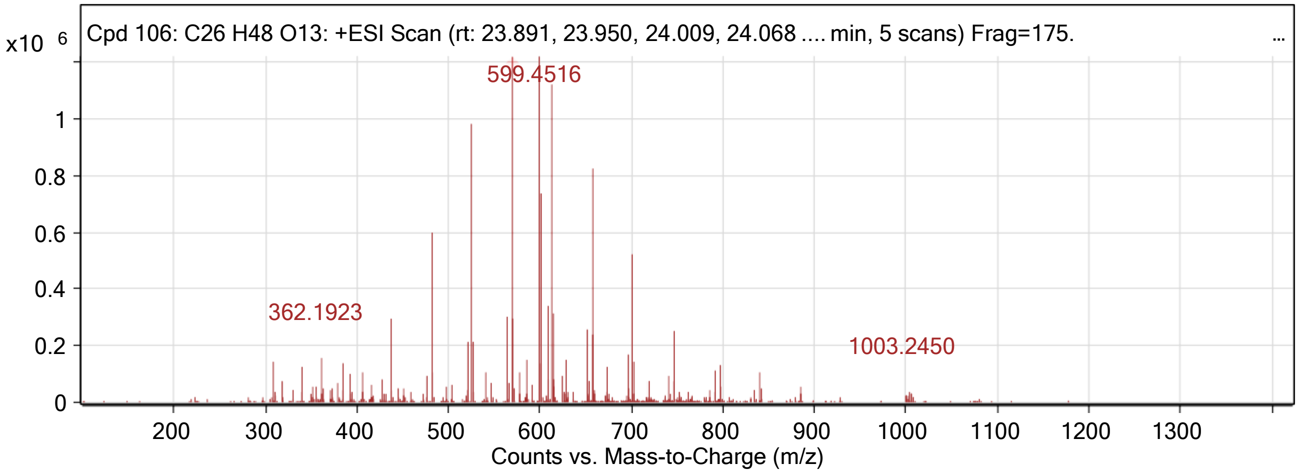

MS Zoomed Spectrum

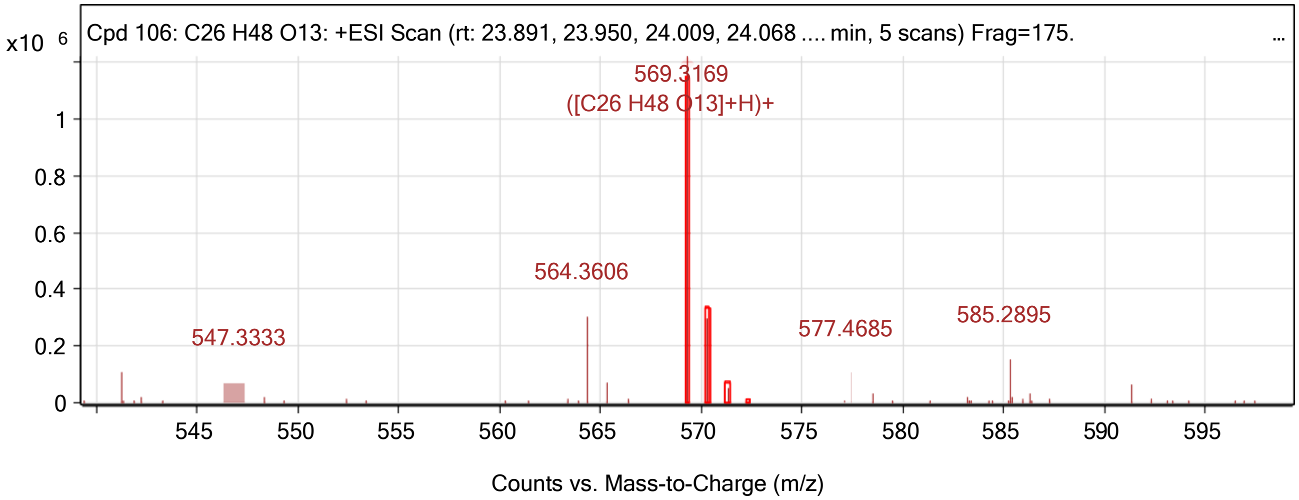

 Printed at 11:53 AM on 24-Dec-2018

MS Spectrum Peak List

m/z	Calc *m/z*	Diff(ppm)	z	Abund	Formula	Ion
481.2634			1	598518.56		
525.2903			1	978449.81		
569.3169	569.3168	-0.18	1	1220115.25	C26 H48 O13	(M+H)+
570.3194	570.3202	1.47	1	293695.94	C26 H48 O13	(M+H)+
571.3214	571.3226	2.18	1	49484.75	C26 H48 O13	(M+H)+
572.3241	572.3253	2.15	1	7024.04	C26 H48 O13	(M+H)+
599.4516			1	2130492.25		
600.4551			1	738615.44		
613.343			1	1118903.75		
657.3699			1	821897.38		

MSMS Spectrum

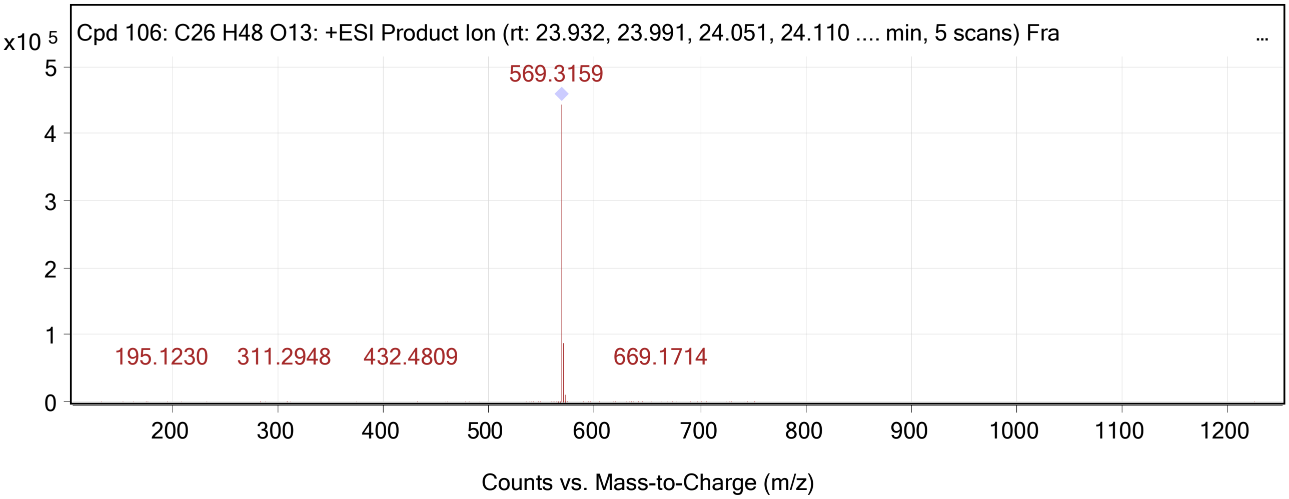

MS/MS Spectrum Peak List

m/z	*Calc m/z*	Diff(ppm)	z	Abund
133.0865	133.0859	-4.14		42.47
175.1311	175.1329	9.85		10.45
177.1111	177.1121	5.67		12.05
208.1325	208.1305	-9.35		14.52
283.2691	283.2632	-21.02		10.81
309.2781	309.2788	2.27		17.79
311.2948	311.2945	-1.11		73.84
481.2654	481.2643	-2.22		11
566.3025	283.1464	-500008.59	2	10.61
569.3159	569.3168	1.56	1	443825.69

Compound Label	Name	*m/z*	RT	Algorithm	Mass
Cpd 107: 1-(O-alpha-D-glucopyranosyl)-(1,3R,25R)-hexacosanetriol	**1-(O-alpha-D-glucopyranosyl)-(1,3R,25R)-hexacosanetriol**	599.452	24.093	Auto MS/MS	576.4627

Compound Chromatograms

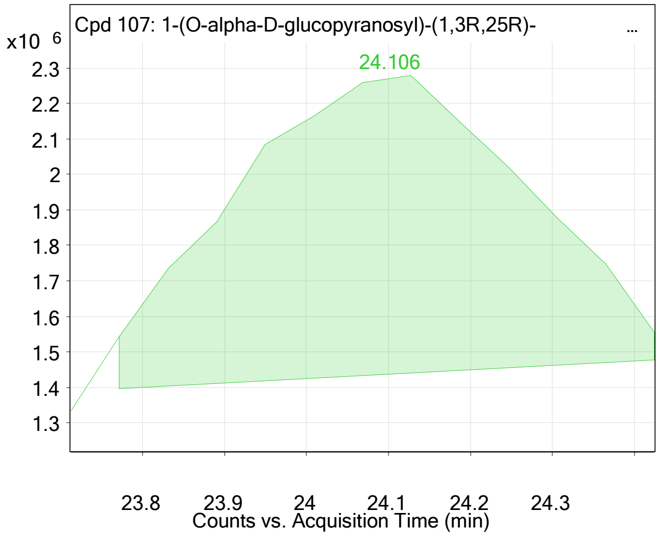

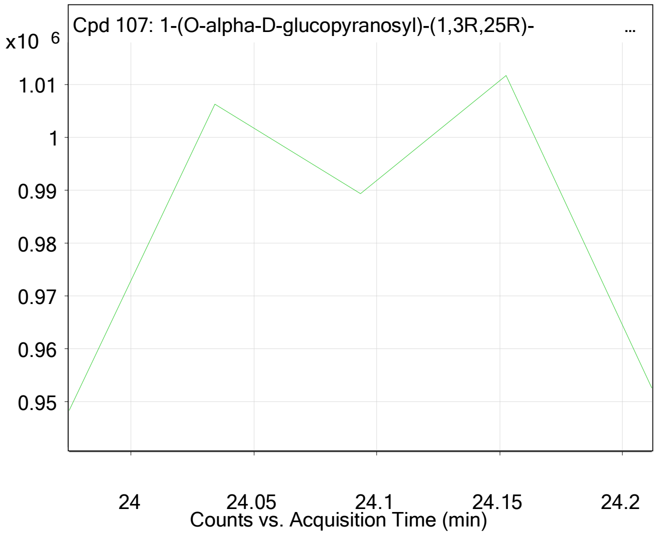

MS Spectrum

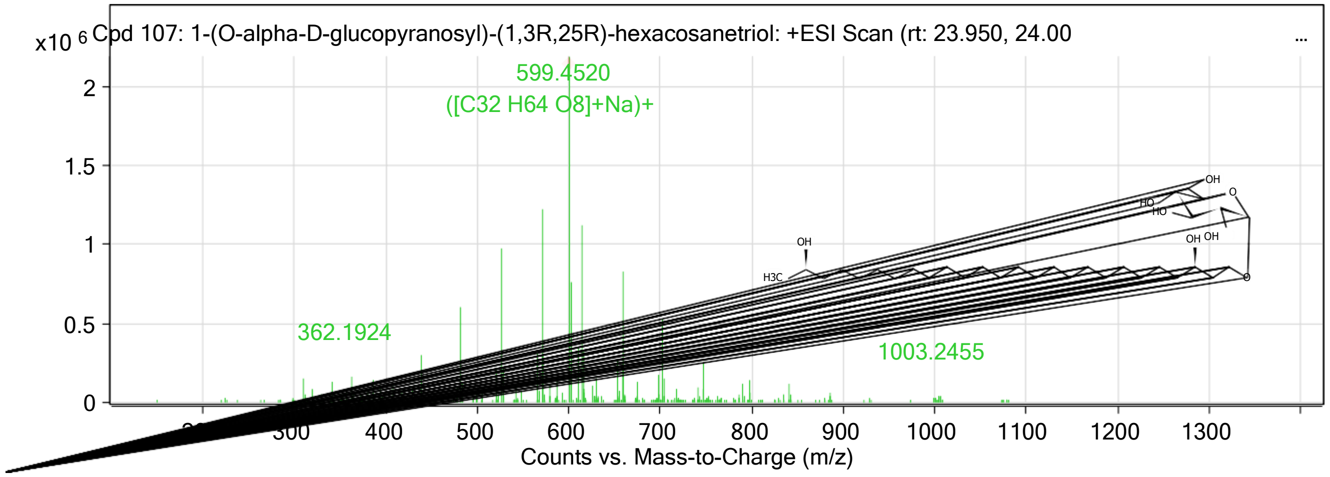

MS Zoomed Spectrum

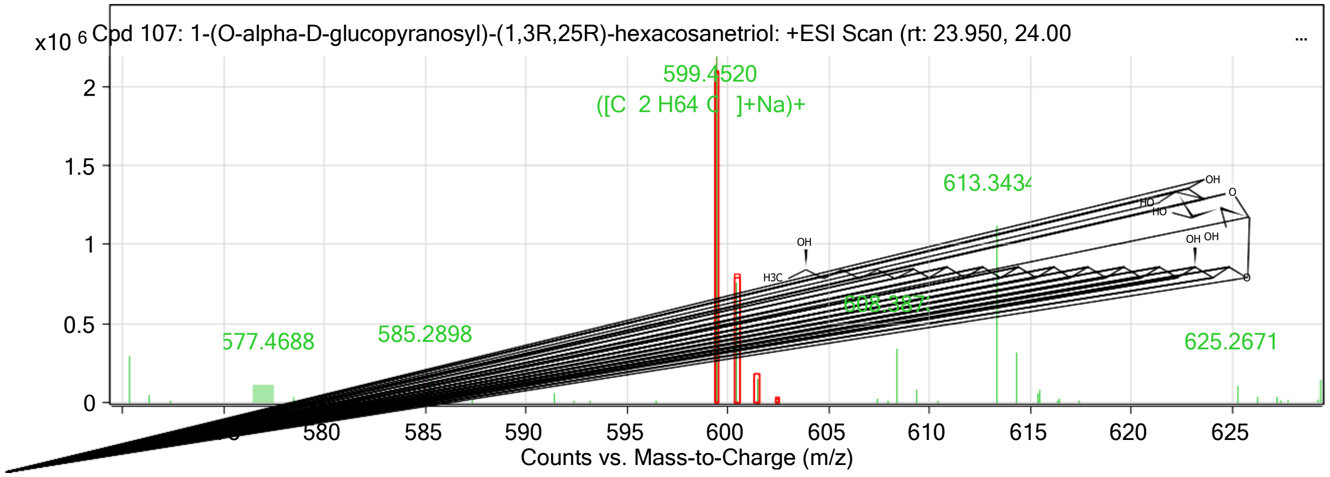

MS Spectrum Peak List

m/z	Calc *m/z*	Diff(ppm)	z	Abund	Formula	Ion
481.2636			1	595879.81		
525.2906			1	973428.5		
569.3172			1	1214531.5		
599.452	599.4493	-4.38	1	2186233.5	C32 H64 O8	(M+Na)+
600.4554	600.4528	-4.44	1	756813.38	C32 H64 O8	(M+Na)+
601.4575	601.4556	-3.17	1	141985.89	C32 H64 O8	(M+Na)+
602.4595	602.4584	-1.78	1	16831.85	C32 H64 O8	(M+Na)+
613.3434			1	1112025.75		
657.3703			1	818650.31		
701.3956			1	520570.16		

MSMS Spectrum

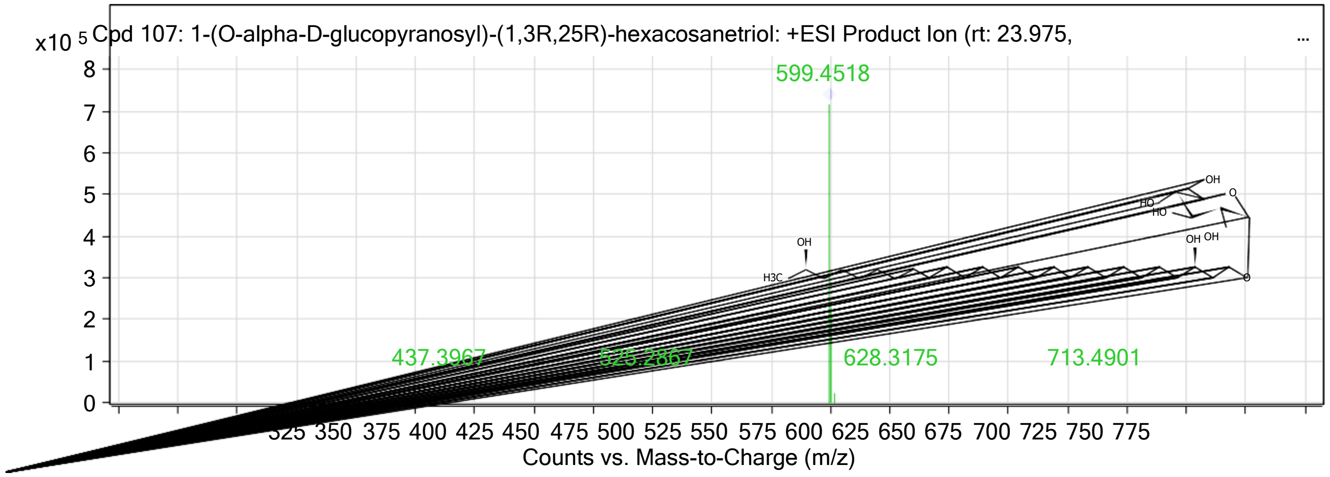

MS/MS Spectrum Peak List

m/z	*Calc m/z*	Diff(ppm)	z	Abund
309.2823	309.2788	-11.34		16.72
311.2945	311.2945	0		31.16
437.3967	437.3989	5.18		29.71
485.383	485.3837	1.34		16.13
595.4194	595.4204	1.7		108.91
599.4518	599.4517	-0.16	1	717009.44
600.4544			1	228363.16
601.3841			2	1105.52
601.4564			1	23486.63
601.6329			2	1111.56

Compound Structure

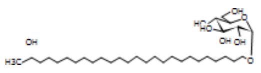

Compound Label	*m/z*	RT	Algorithm	Mass
Cpd 108: C26 H48 O13	569.3171	24.347	Auto MS/MS	568.3096

Compound Chromatograms

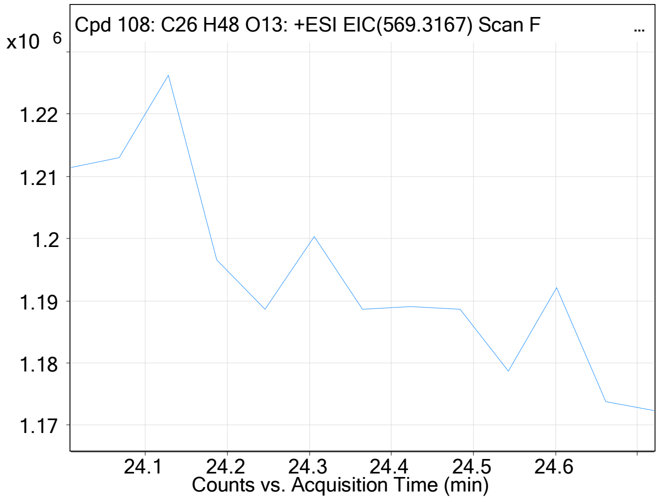

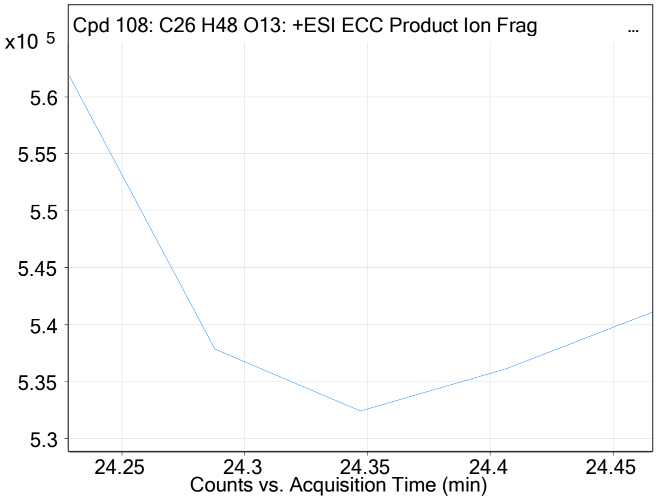

MS Spectrum

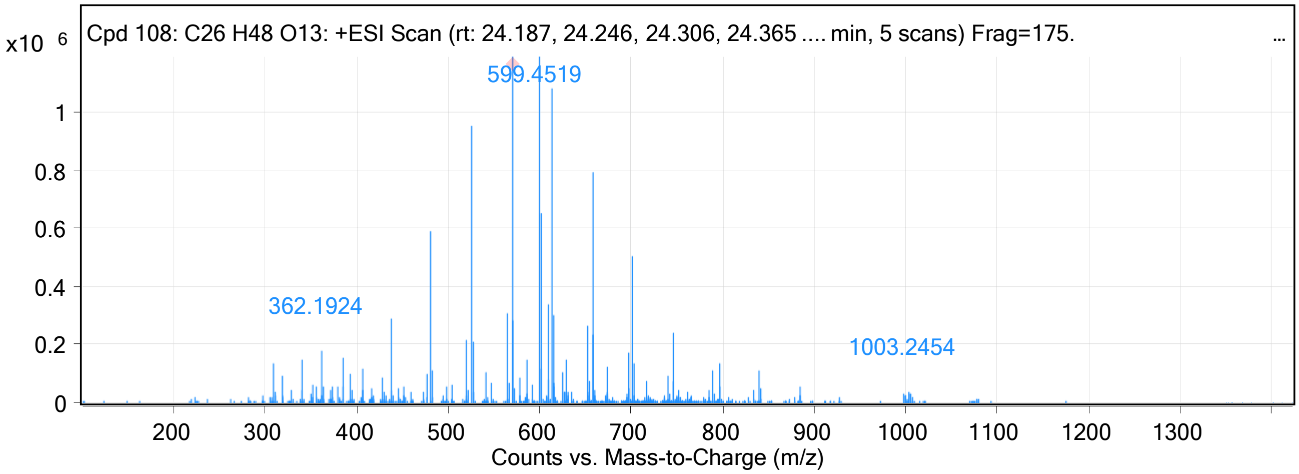

MS Zoomed Spectrum

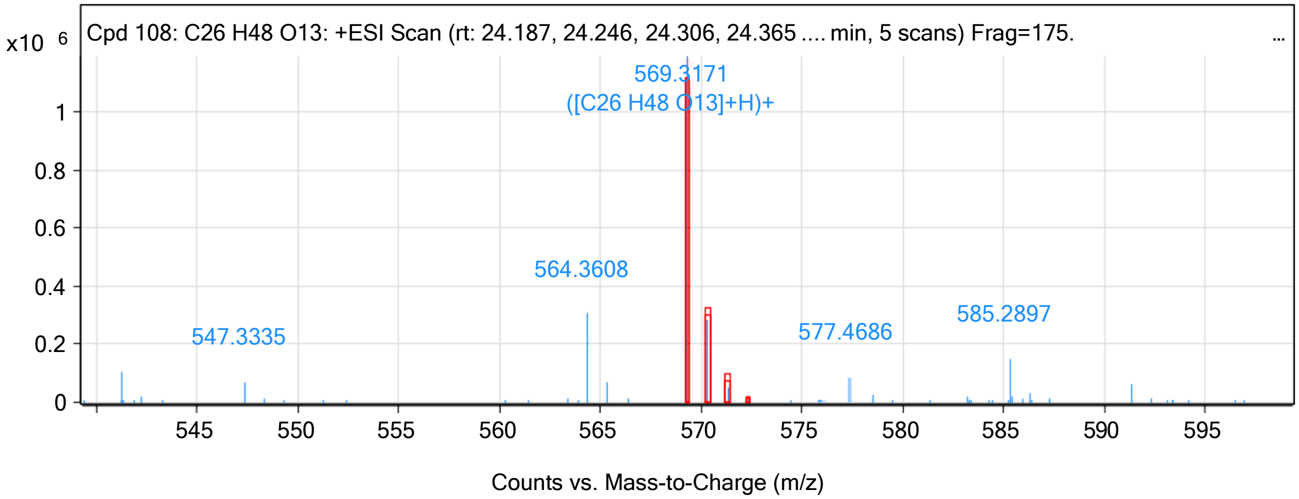

MS Spectrum Peak List

m/z	*Calc m/z*	Diff(ppm)	z	Abund	Formula	Ion
481.2636			1	593076.63		
525.2905			1	955259.31		
569.3171	569.3168	-0.55	1	1192680.13	C26 H48 O13	(M+H)+
570.3195	570.3202	1.17	1	283467.19	C26 H48 O13	(M+H)+
571.3215	571.3226	1.97	1	48371.6	C26 H48 O13	(M+H)+
572.3238	572.3253	2.54	1	6457.95	C26 H48 O13	(M+H)+
599.4519			1	1870128.25		
600.4552			1	651010.94		
613.3433			1	1082561.38		
657.3702			1	790539.25		

MSMS Spectrum

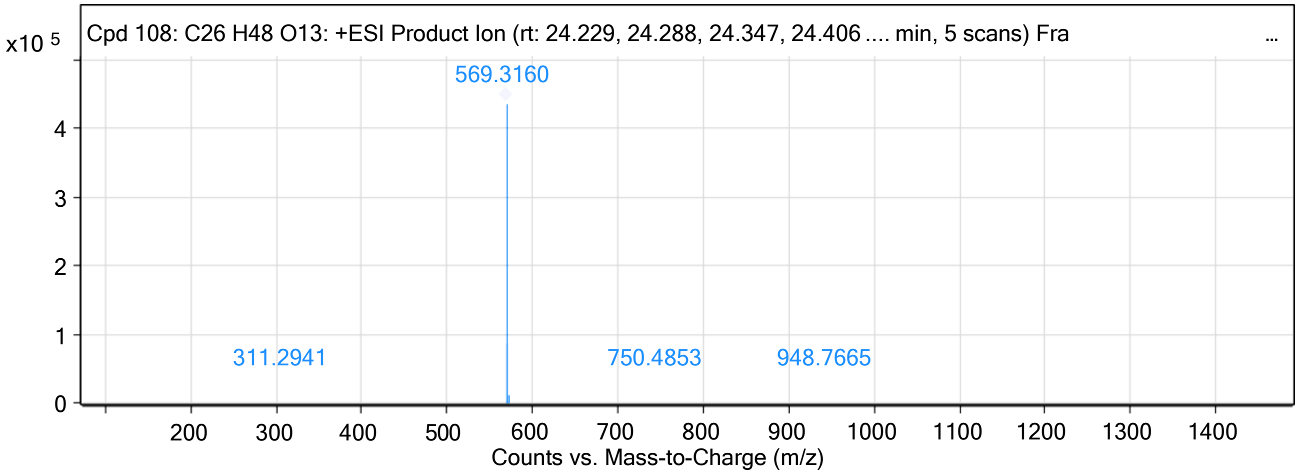

MS/MS Spectrum Peak List

m/z	*Calc m/z*	Diff(ppm)	z	Abund
133.0851	133.0859	6.06		23.64
309.278	309.2788	2.64		45.95
311.2941	311.2945	1.02		69.51
568.3623			2	686.67
568.6119			1	524.14
568.8621			2	474.63
569.316	569.3168	1.32	1	435245.06
570.3186			1	85659.48
571.3207			1	10216.75
571.3884			2	715.02

Compound Label	Name	*m/z*	RT	Algorithm	Mass
Cpd 109: 1-(O-alpha-D-glucopyranosyl)-(1,3R,25R)-hexacosanetriol	**1-(O-alpha-D-glucopyranosyl)-(1,3R,25R)-hexacosanetriol**	599.4517	24.39	Auto MS/MS	576.4624

Compound Chromatograms

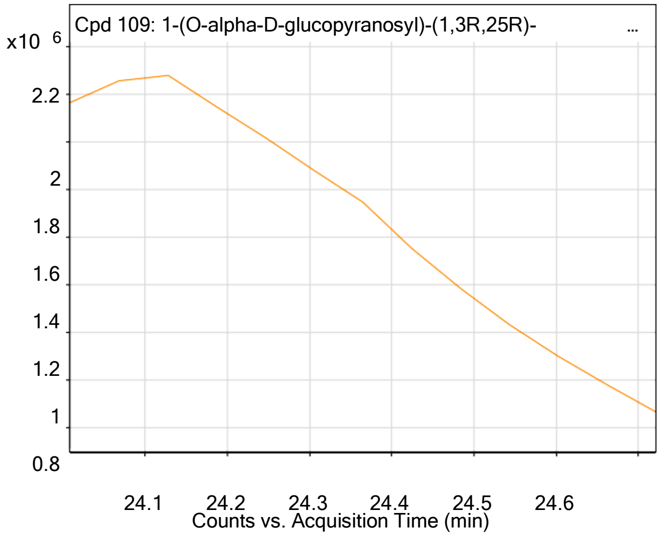

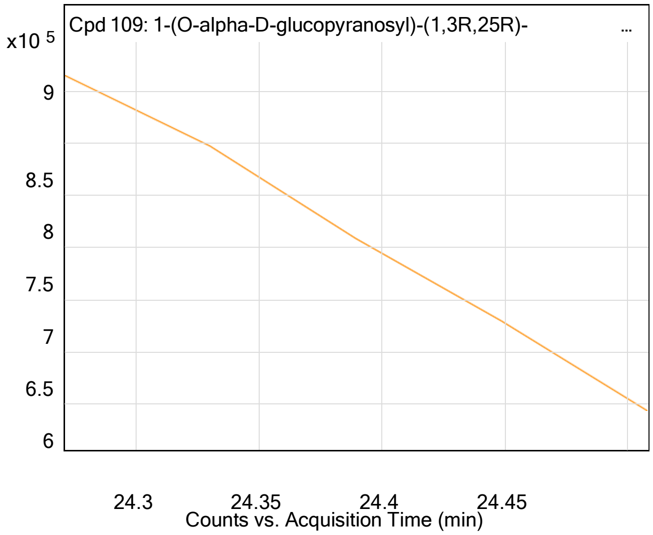

MS Spectrum

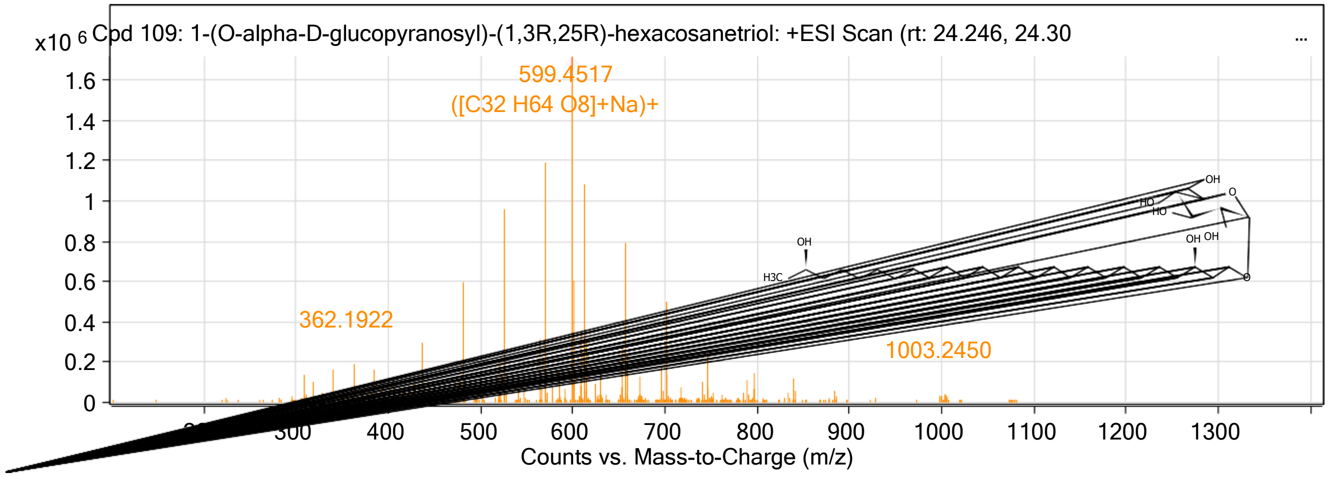

MS Zoomed Spectrum

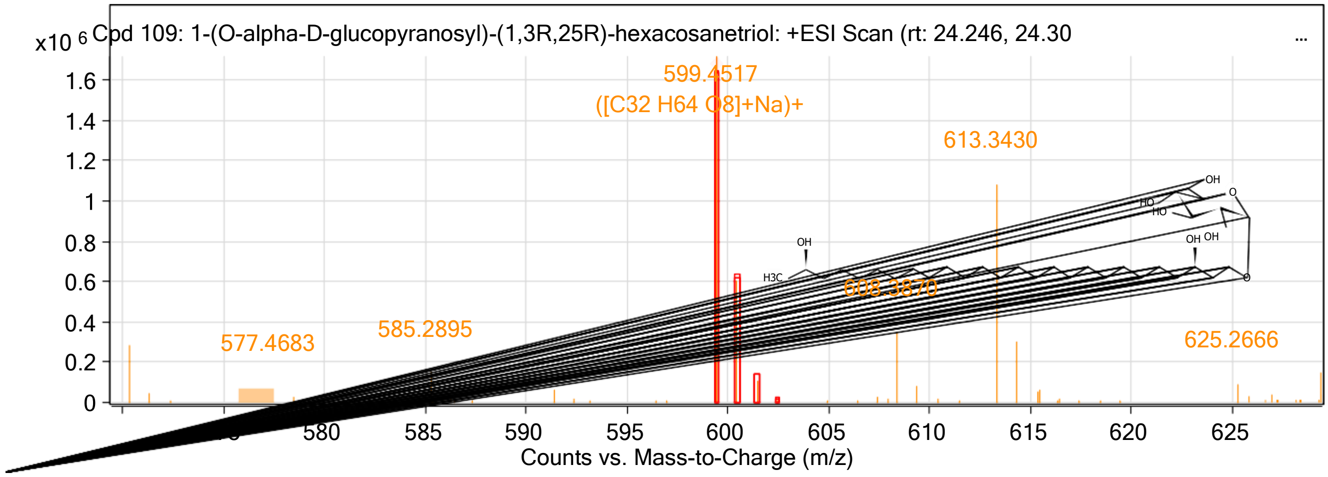

MS Spectrum Peak List

m/z	Calc *m/z*	Diff(ppm)	z	Abund	Formula	Ion
481.2634			1	593118.31		
525.2903			1	953829.88		
569.3169			1	1191094.75		
599.4517	599.4493	-3.95	1	1717793.75	C32 H64 O8	(M+Na)+
600.4549	600.4528	-3.56	1	601585.06	C32 H64 O8	(M+Na)+
601.457	601.4556	-2.21	1	107491.75	C32 H64 O8	(M+Na)+
602.4591	602.4584	-1.14	1	12970.83	C32 H64 O8	(M+Na)+
613.343			1	1080732.25		
657.3699			1	786895		

MSMS Spectrum

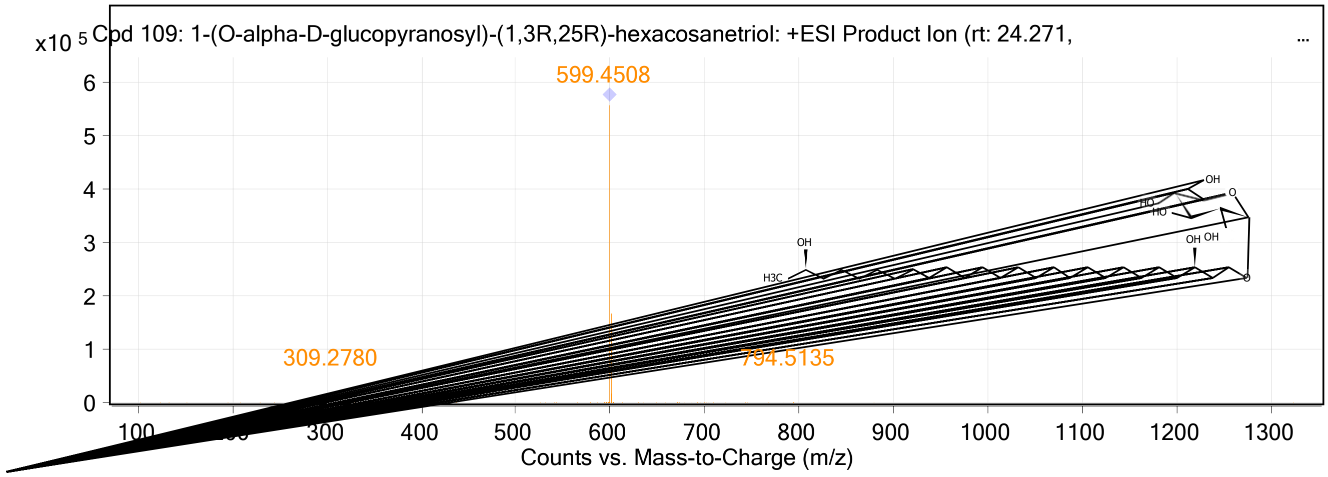

MS/MS Spectrum Peak List

m/z	*Calc m/z*	Diff(ppm)	z	Abund
133.0875	133.0859	-11.93		20.24
309.278	309.2788	2.72	1	73.65
398.3812	398.3754	-14.49		12.11
437.3945	437.3989	10.08		58.86
596.4279	596.4283	0.62		59.23
599.4508	599.4517	1.52	1	558242.88
600.4539			1	168435.06
601.3822			2	1368.93
601.4559			1	17590.48
601.6323			2	1152.45

Compound Structure

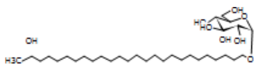

Compound Label	Name	*m/z*	RT	Algorithm	Mass
Cpd 110: 1-(O-alpha-D-glucopyranosyl)-(1,3R,25R)-hexacosanetriol	**1-(O-alpha-D-glucopyranosyl)-(1,3R,25R)-hexacosanetriol**	599.452	24.606	Auto MS/MS	576.4626

Compound Chromatograms

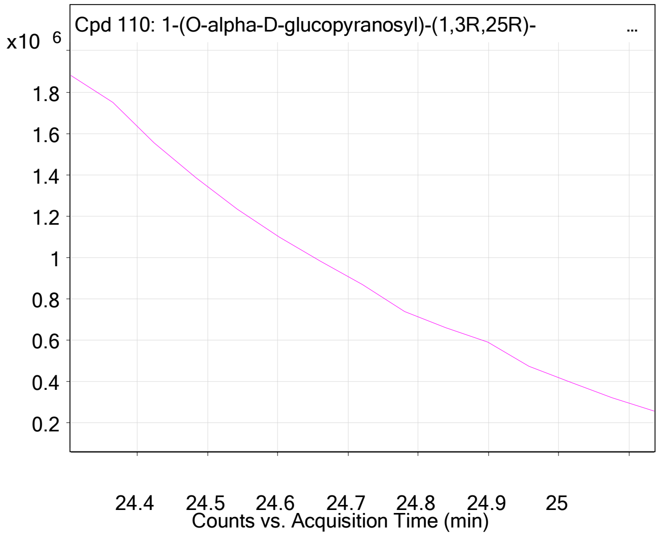

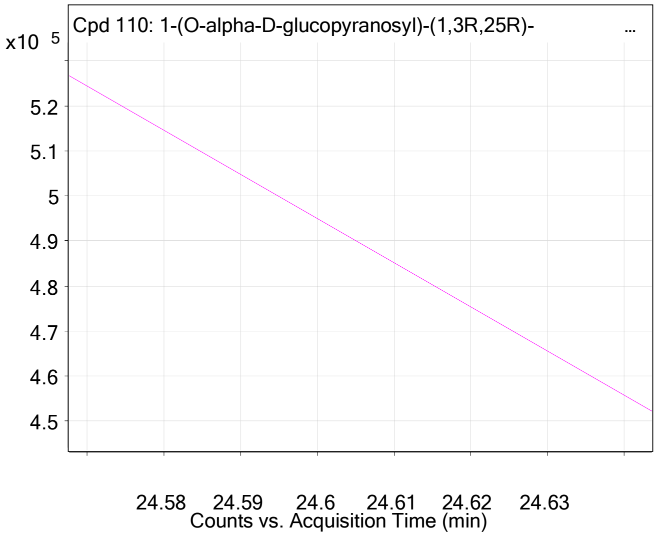

MS Spectrum

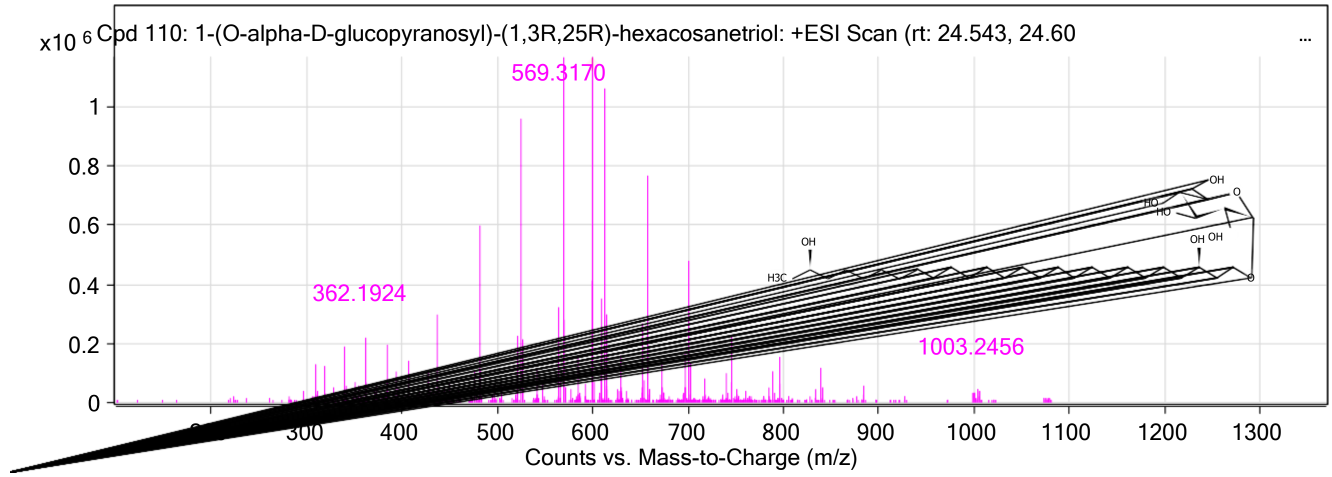

MS Zoomed Spectrum

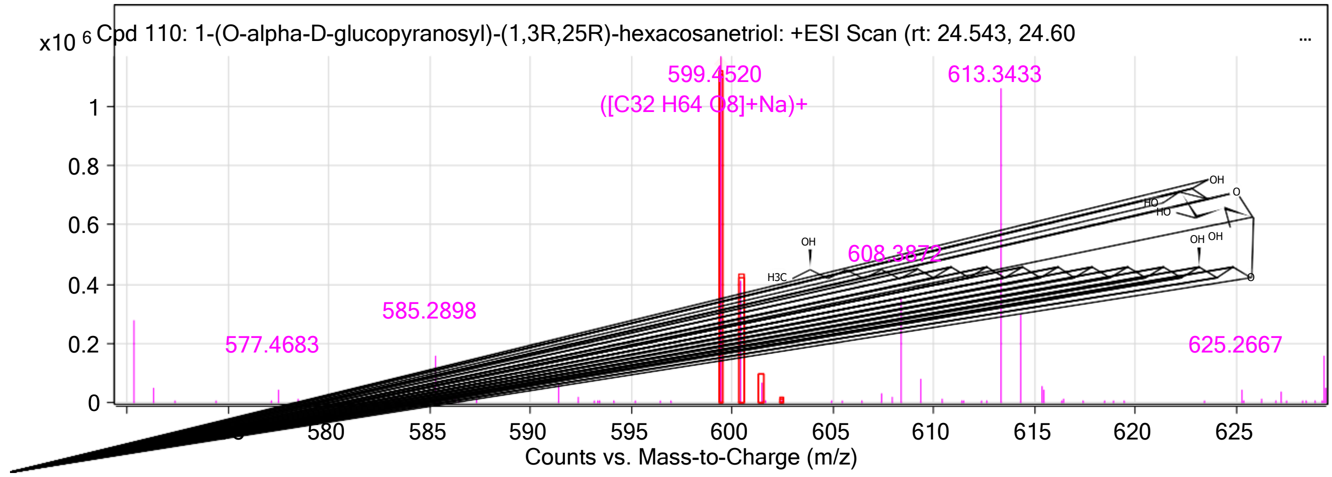

MS Spectrum Peak List

m/z	Calc *m/z*	Diff(ppm)	z	Abund	Formula	Ion
481.2636			1	597286.75		
525.2905			1	957289.13		
569.317			1	1185430.88		
599.452	599.4493	-4.42	1	1167293.75	C32 H64 O8	(M+Na)+
600.4549	600.4528	-3.59	1	407795.91	C32 H64 O8	(M+Na)+
601.457	601.4556	-2.19	1	68369.09	C32 H64 O8	(M+Na)+
602.4595	602.4584	-1.83	1	8401.43	C32 H64 O8	(M+Na)+
613.3433			1	1060815.88		
657.3699			1	766827.63		
701.3954			1	477852.44		

MSMS Spectrum

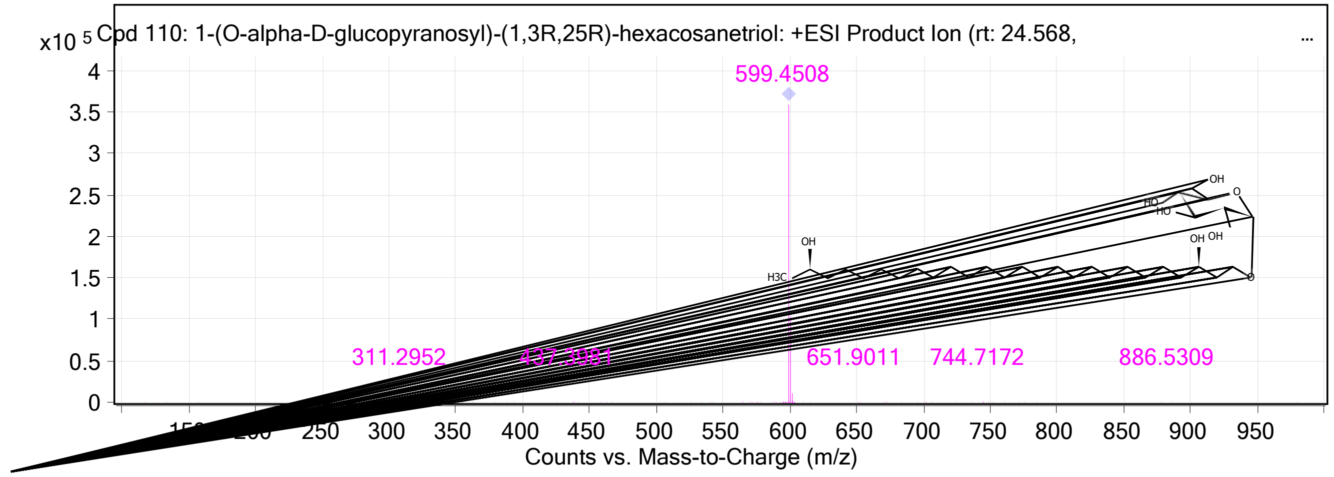

MS/MS Spectrum Peak List

m/z	*Calc m/z*	Diff(ppm)	z	Abund
117.0908	117.091	1.93		29.32
133.0872	133.0859	-9.68		38.12
157.1221	157.1223	1.11		19.93
311.2952	311.2945	-2.42		50.19
320.2257	320.2193	-19.96		21.84
425.2609	425.2534	-17.74		21.33
437.3981	437.3989	1.77	1	68.94
597.4315	597.4361	7.7		19.78
599.4508	599.4517	1.62	1	358881.88
600.4537			1	103390.81

Compound Structure

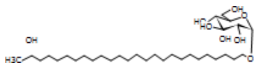

Compound Label	*m/z*	RT	Algorithm	Mass
Cpd 111: C26 H48 O13	569.317	24.635	Auto MS/MS	568.3095

Compound Chromatograms

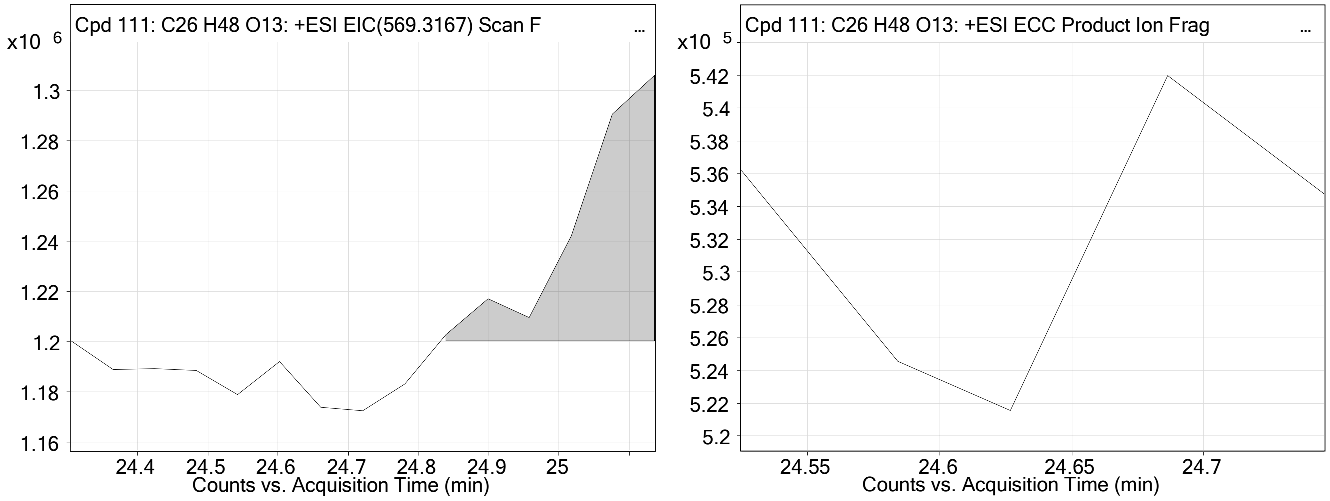

MS Spectrum

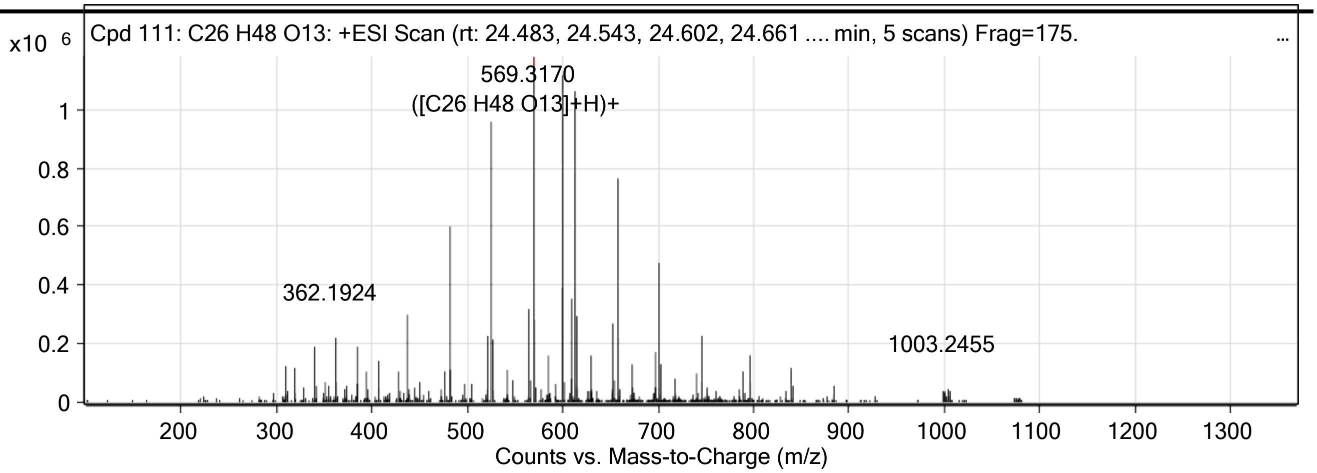

MS Zoomed Spectrum

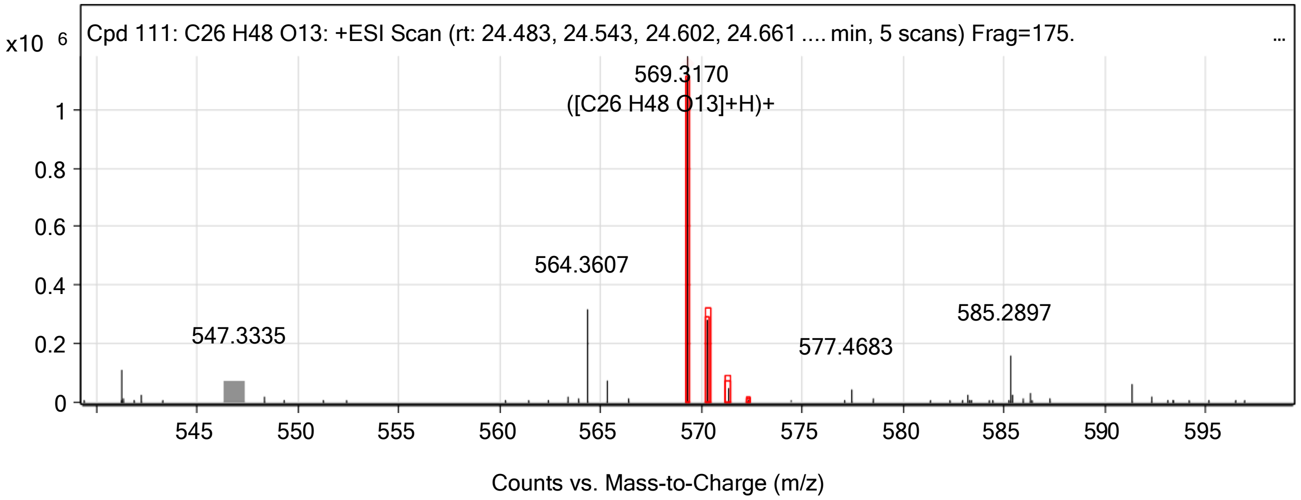

MS Spectrum Peak List

m/z	Calc *m/z*	Diff(ppm)	z	Abund	Formula	Ion
481.2635			1	596426.63		
525.2904			1	954790		
569.317	569.3168	-0.37	1	1181147.75	C26 H48 O13	(M+H)+
570.3194	570.3202	1.38	1	277598.38	C26 H48 O13	(M+H)+
571.3214	571.3226	2.13	1	47555.25	C26 H48 O13	(M+H)+
572.3245	572.3253	1.45	1	6526.93	C26 H48 O13	(M+H)+
599.452			1	1113898		
613.3432			1	1058206.63		
657.3698			1	763278.81		
701.3953			1	476090.5		

MSMS Spectrum

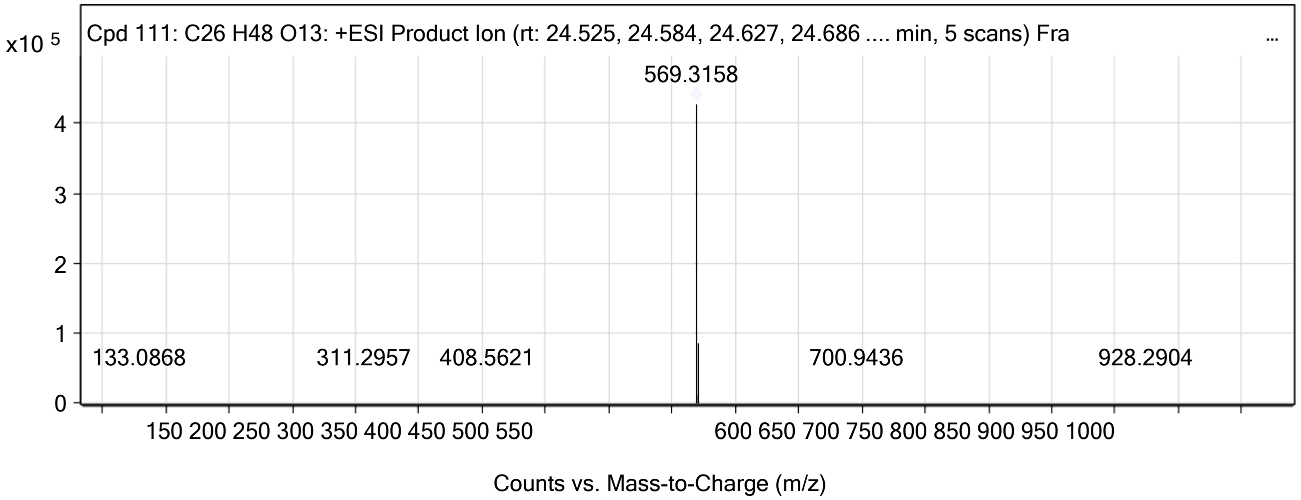

MS/MS Spectrum Peak List

m/z	*Calc m/z*	Diff(ppm)	z	Abund
133.0868	133.0859	-6.94		44.33
232.1318	232.1305	-5.6		15.01
283.2643	283.2632	-3.95		12.28
307.2114	307.2115	0.46		12.09
309.2803	309.2788	-4.98		25.96
311.2957	311.2945	-3.95		59.82
496.2799	496.2878	15.95		14.71
538.2985	538.2984	-0.17		26.12
539.3004	539.3062	10.75		27.35
569.3158	569.3168	1.63	1	427114.34

Compound Label	*m/z*	RT	Algorithm	Mass
Cpd 112: C28 H52 O14	613.3433	24.821	Auto MS/MS	612.3358

Compound Chromatograms

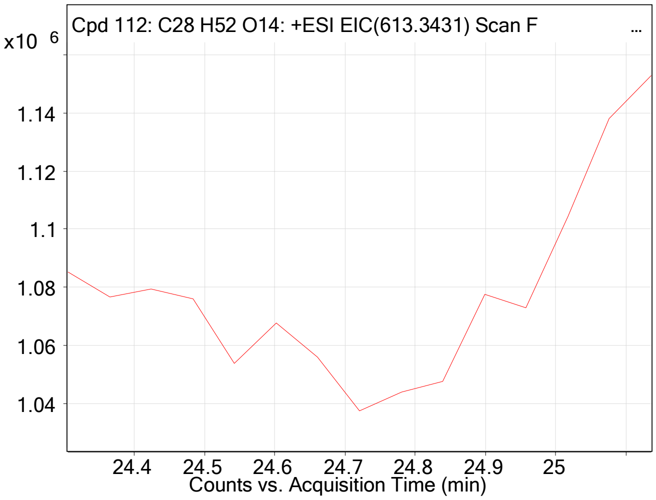

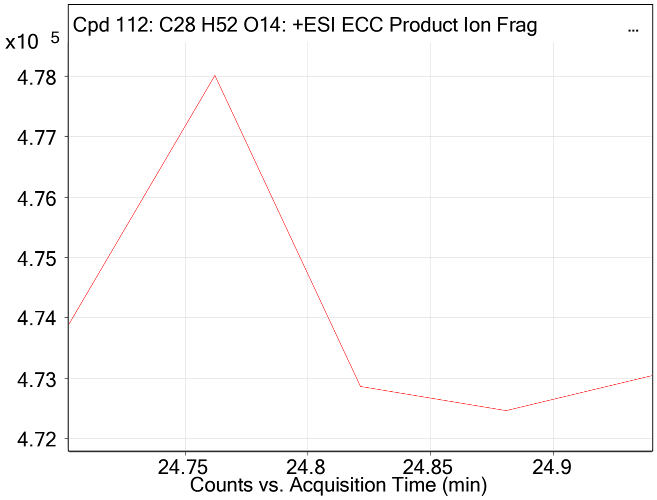

MS Spectrum

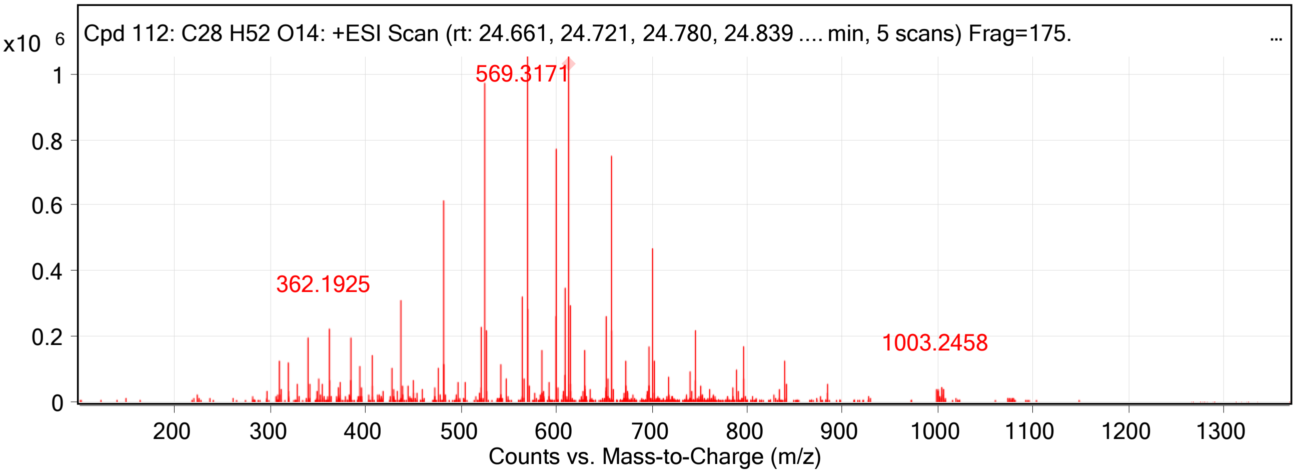

MS Zoomed Spectrum

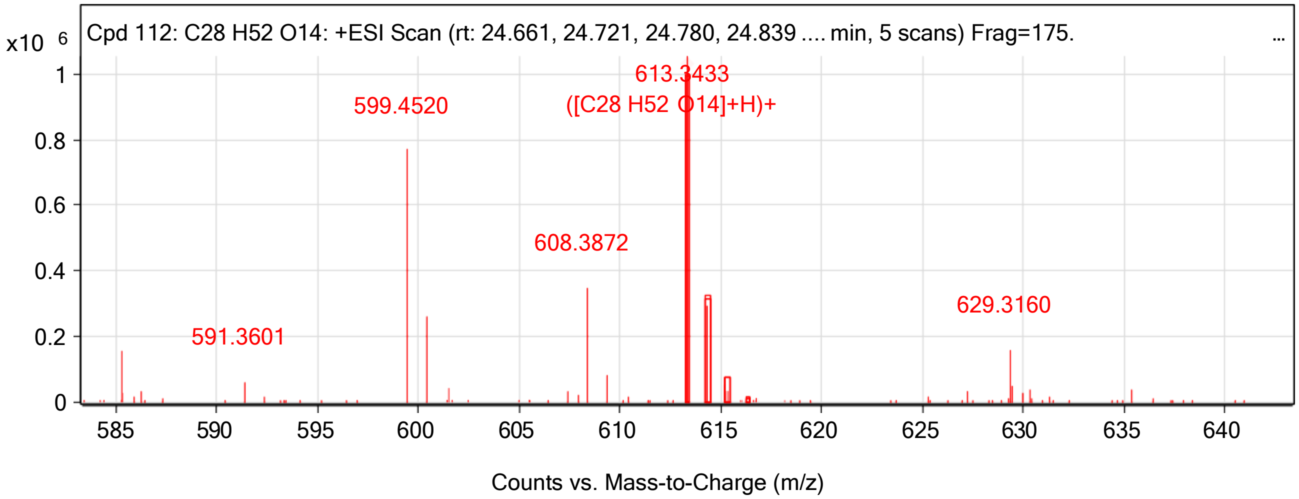

MS Spectrum Peak List

m/z	Calc *m/z*	Diff(ppm)	z	Abund	Formula	Ion
481.2636			1	611477.25		
525.2905			1	970788.38		
569.3171			1	1189866.38		
599.452			1	768206.31		
613.3433	613.343	-0.5	1	1052504.13	C28 H52 O14	(M+H)+
614.3459	614.3464	0.79	1	291871.13	C28 H52 O14	(M+H)+
615.3478	615.3489	1.78	1	51937.61	C28 H52 O14	(M+H)+
616.3519	616.3515	-0.51	1	7028.24	C28 H52 O14	(M+H)+
657.3698			1	748945.88		
701.3955			1	465562.25		

MSMS Spectrum

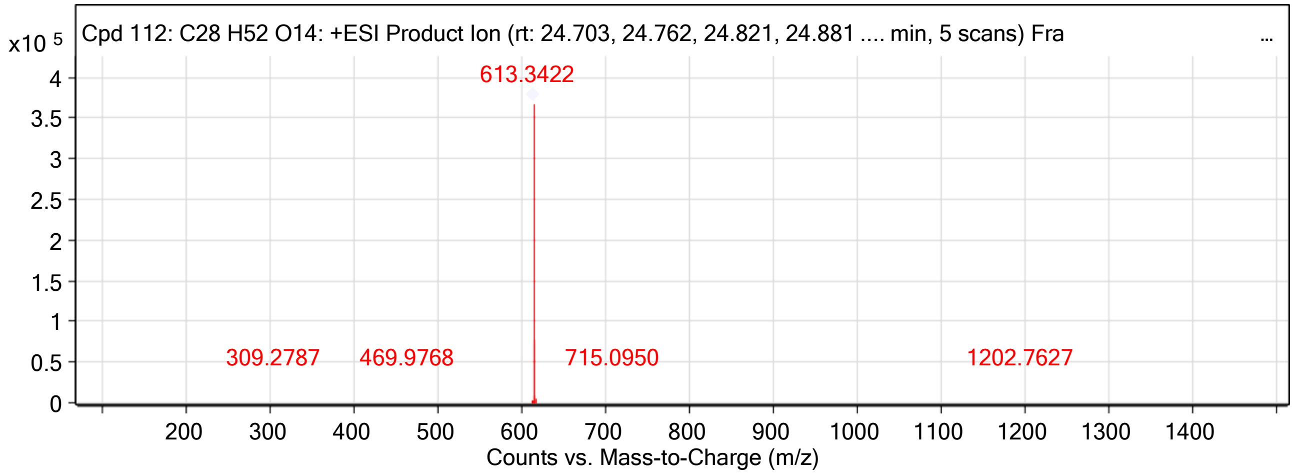

MS/MS Spectrum Peak List

m/z	Calc *m/z*	Diff(ppm)	z	Abund
133.0872	133.0859	-9.57		39.59
175.1312	175.1329	9.54		16.09
309.2787	309.2788	0.38	1	48.15
310.2848			1	19.66
311.2953	311.2945	-2.68		36.1
312.2972	312.3023	16.16		15.84
589.3356	589.343	12.61		18.64
613.3422	613.343	1.36	1	366642.06
614.3448			1	77447.09
615.3469			1	9009.92

Compound Label	*m/z*	RT	Algorithm	Mass
Cpd 113: C26 H48 O13	569.3169	24.923	Auto MS/MS	568.3095

Compound Chromatograms

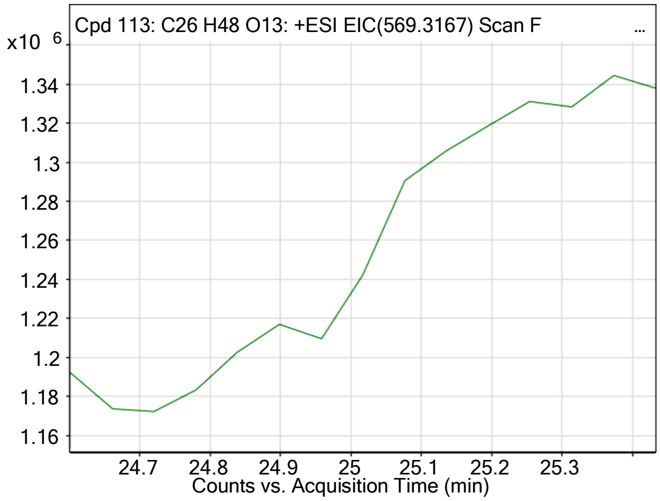

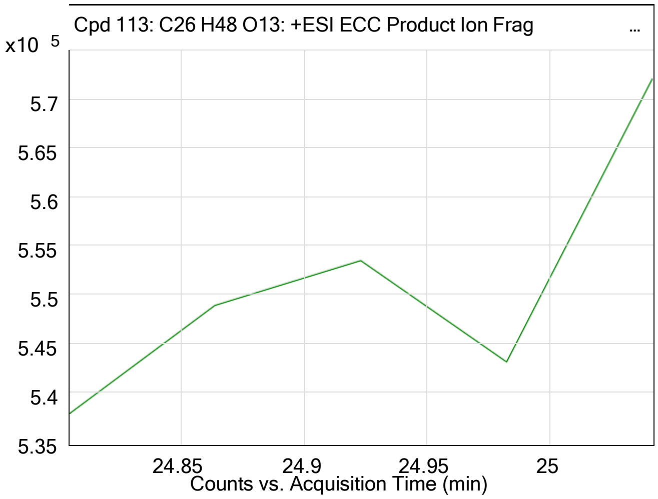

MS Spectrum

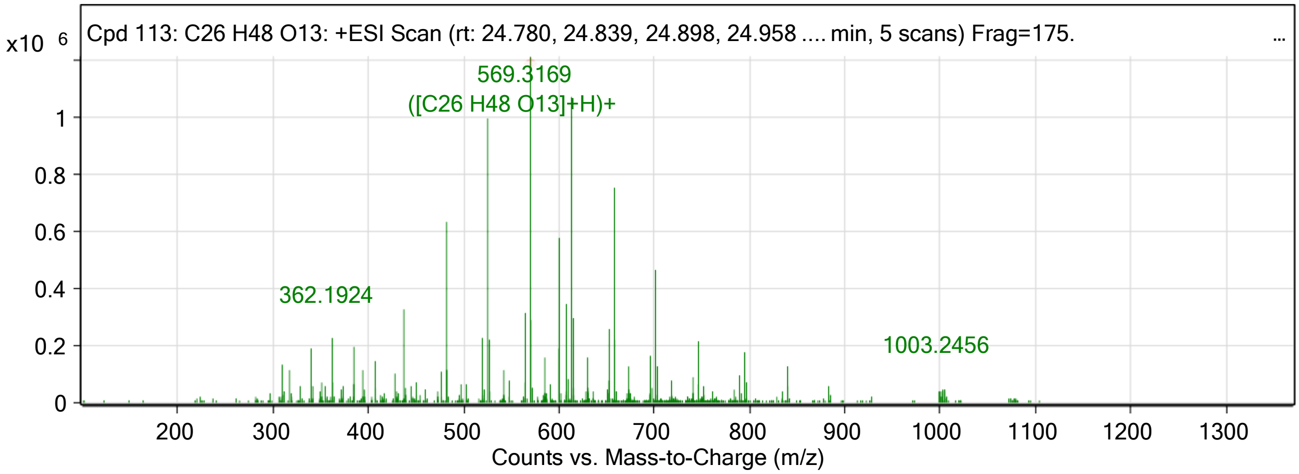

MS Zoomed Spectrum

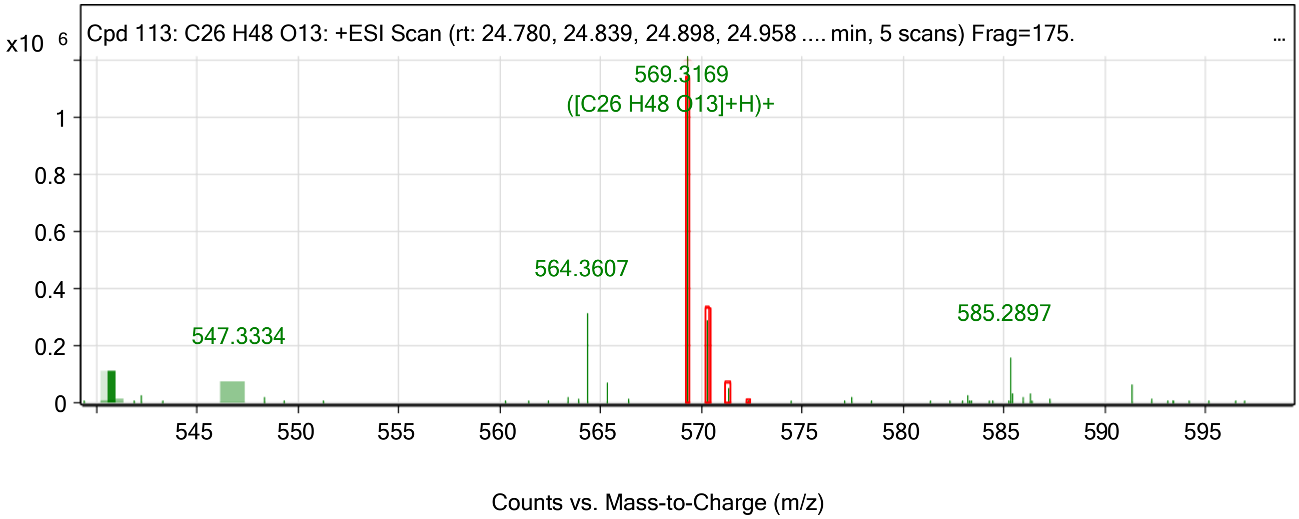

MS Spectrum Peak List

m/z	Calc *m/z*	Diff(ppm)	z	Abund	Formula	Ion
481.2635			1	630330.38		
525.2904			1	995894.19		
569.3169	569.3168	-0.31	1	1210979.63	C26 H48 O13	(M+H)+
570.3195	570.3202	1.29	1	288643.03	C26 H48 O13	(M+H)+
571.3215	571.3226	1.97	1	49471.29	C26 H48 O13	(M+H)+
572.3239	572.3253	2.41	1	6738.06	C26 H48 O13	(M+H)+
599.4515			1	572476.81		
613.3431			1	1069325.5		
657.3695			1	750678.13		

MSMS Spectrum

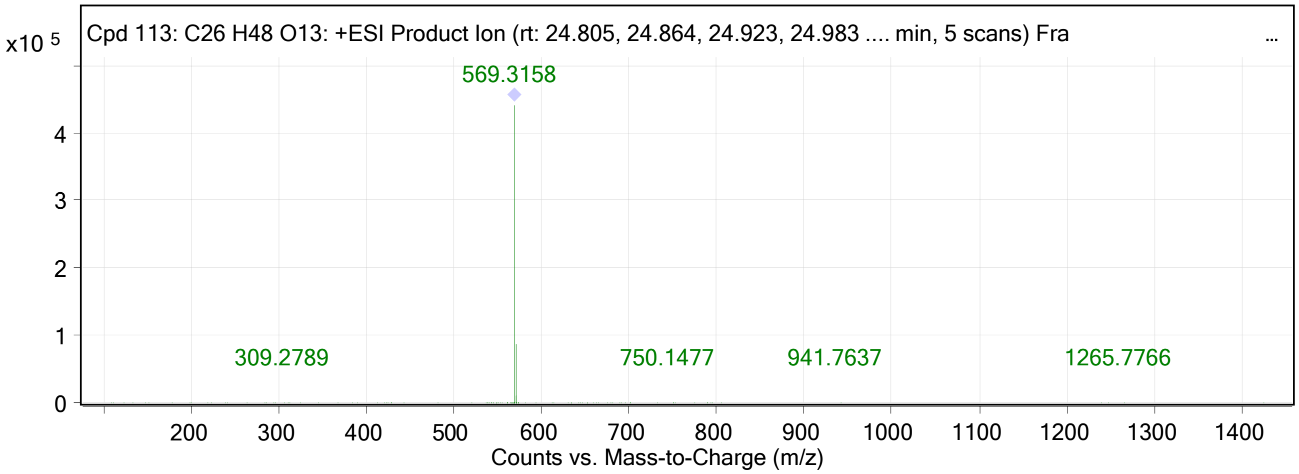

MS/MS Spectrum Peak List

m/z	*Calc m/z*	Diff(ppm)	z	Abund
133.0857	133.0859	1.44		54.25
309.2789	309.2788	-0.17		31.51
538.2932	538.2984	9.54		28.58
539.311	539.3062	-8.94		21.25
566.2876	283.1464	-499995.49	2	22.09
567.2958			2	14.79
569.3158	569.3168	1.66	1	441444.69
570.3184			1	88479.85
571.3205			1	10463.34
571.3877			2	809.09

Compound Label	*m/z*	RT	Algorithm	Mass
Cpd 114: C28 H52 O14	613.343	25.118	Auto MS/MS	612.3355

Compound Chromatograms

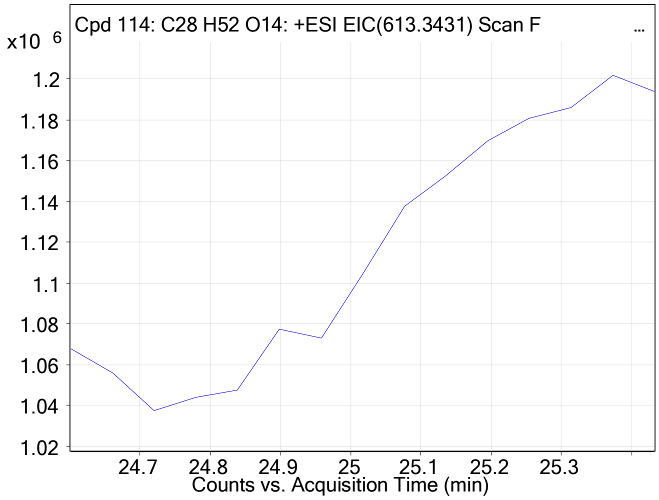

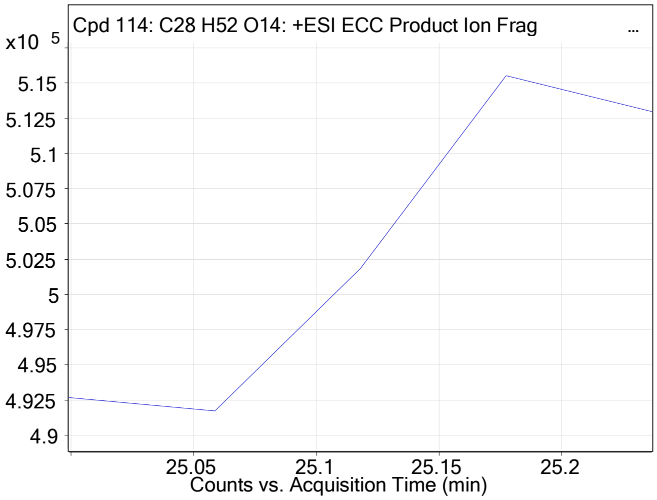

MS Spectrum

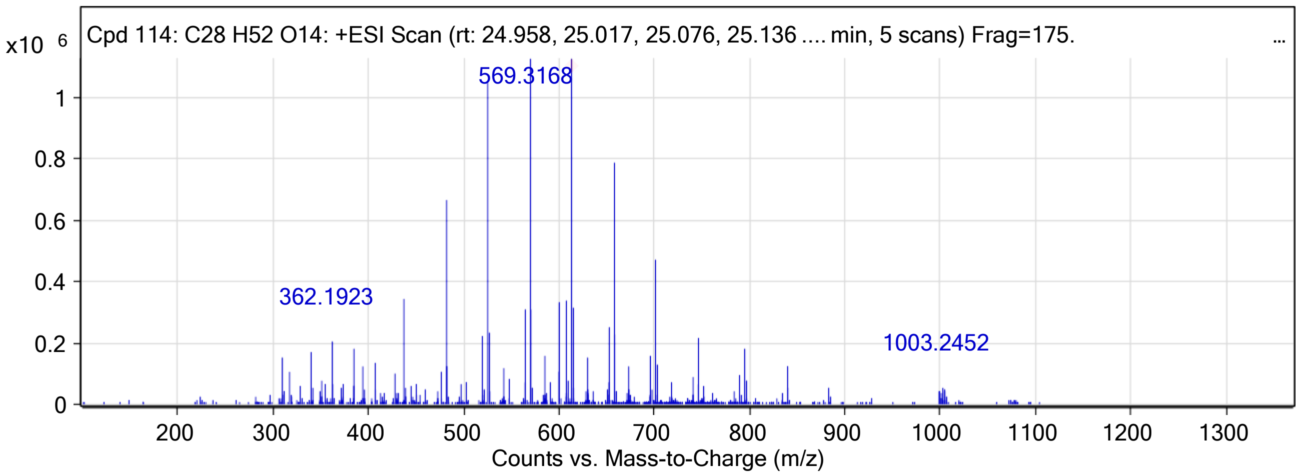

MS Zoomed Spectrum

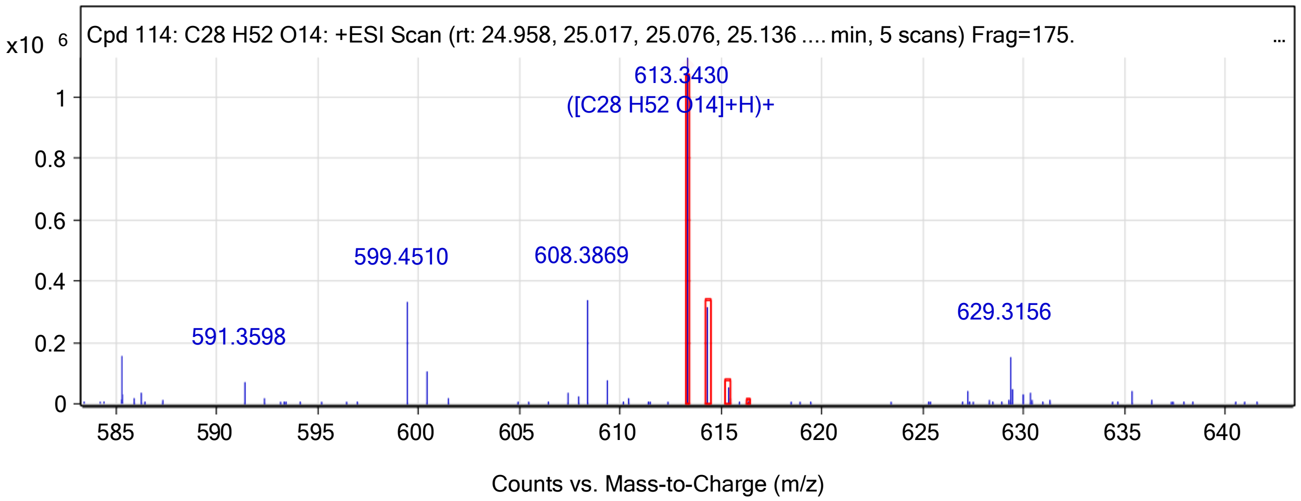

MS Spectrum Peak List

m/z	Calc *m/z*	Diff(ppm)	z	Abund	Formula	Ion
437.2369			1	342799.28		
481.2634			1	661932.38		
525.2902			1	1048355.69		
569.3168			1	1273408.63		
613.343	613.343	0.04	1	1127644.75	C28 H52 O14	(M+H)+
614.3456	614.3464	1.34	1	312974.06	C28 H52 O14	(M+H)+
615.3475	615.3489	2.19	1	55245.3	C28 H52 O14	(M+H)+
616.3511	616.3515	0.75	1	7672.24	C28 H52 O14	(M+H)+
657.3692			1	787814.63		
701.395			1	474058.44		

MSMS Spectrum

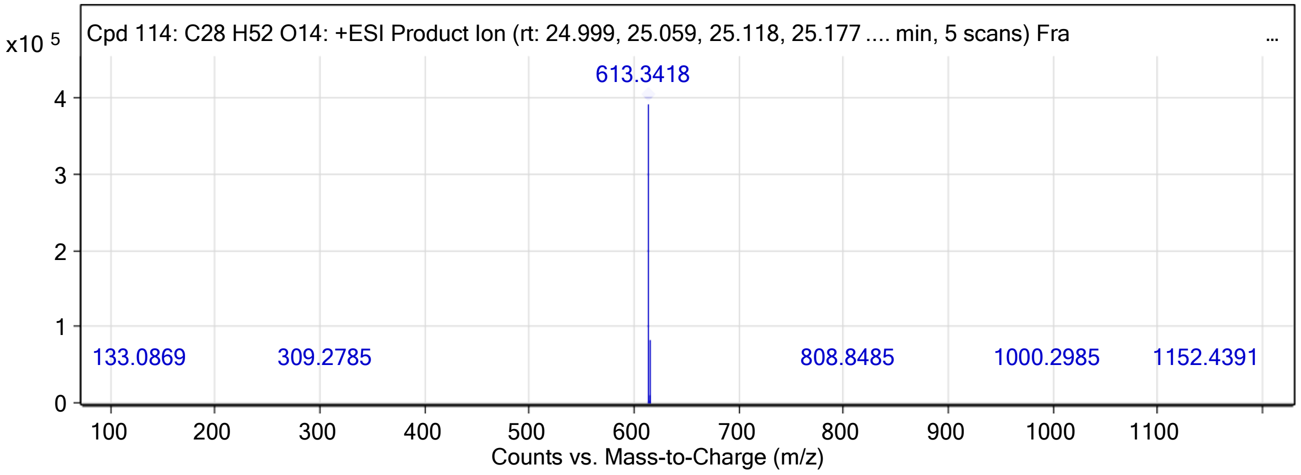

MS/MS Spectrum Peak List

m/z	*Calc m/z*	Diff(ppm)	z	Abund
133.0869	133.0859	-7.63		20.03
232.1264	232.1305	17.96		19.2
283.2631	283.2632	0.35		18.52
309.2785	309.2788	1.03		63.32
311.2943	311.2945	0.43	1	50.83
612.6388				1256.58
613.3418	613.343	1.88	1	391798.19
614.3445			1	83303.79
615.3467			1	9926.25
615.4205			2	2716.38

Compound Label	*m/z*	RT	Algorithm	Mass
Cpd 115: C26 H48 O13	569.317	25.22	Auto MS/MS	568.3095

Compound Chromatograms

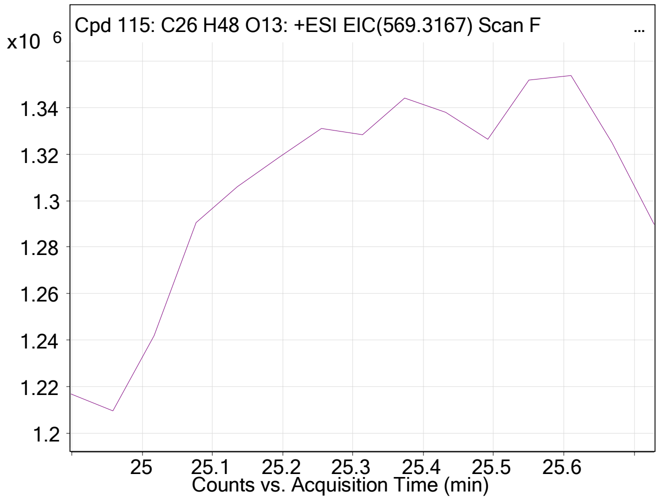

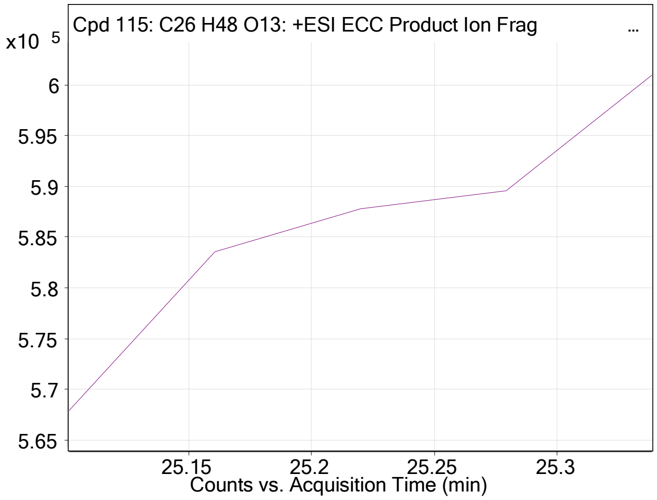

MS Spectrum

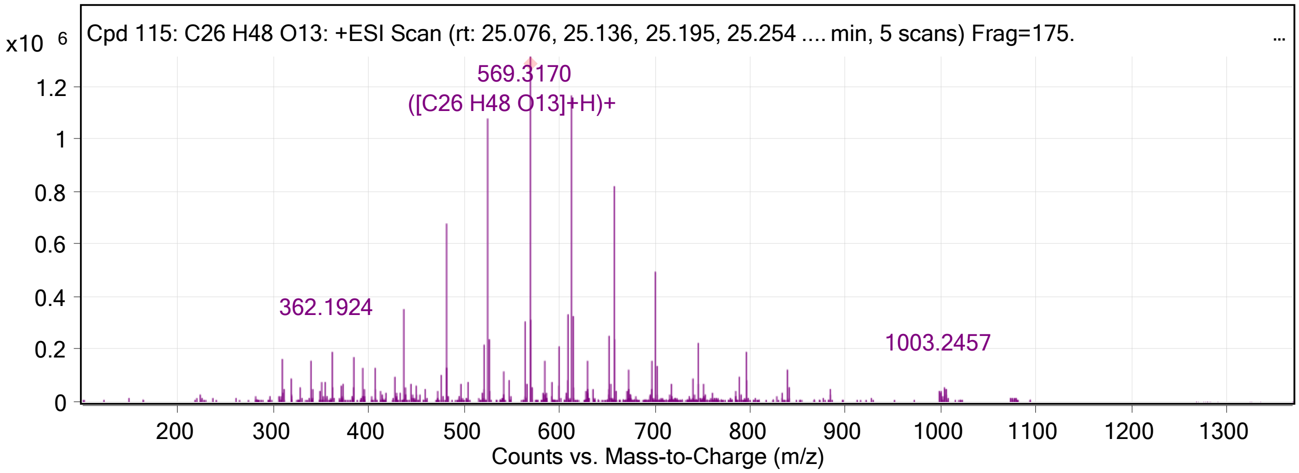

MS Zoomed Spectrum

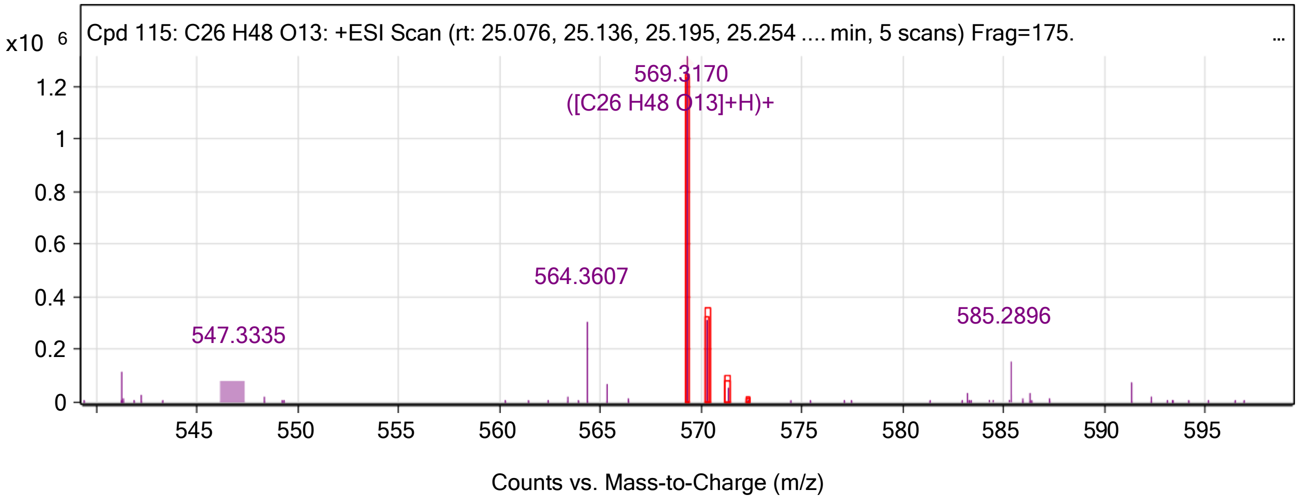

MS Spectrum Peak List

m/z	Calc *m/z*	Diff(ppm)	z	Abund	Formula	Ion
437.2371			1	352291.19		
481.2635			1	675749.13		
525.2905			1	1076950.88		
569.317	569.3168	-0.37	1	1314946	C26 H48 O13	(M+H)+
570.3194	570.3202	1.31	1	314517.03	C26 H48 O13	(M+H)+
571.3215	571.3226	2	1	54123.52	C26 H48 O13	(M+H)+
572.3239	572.3253	2.38	1	7230.46	C26 H48 O13	(M+H)+
613.3432			1	1165578.25		
657.3694			1	817903.75		
701.3953			1	493260.09		

MSMS Spectrum

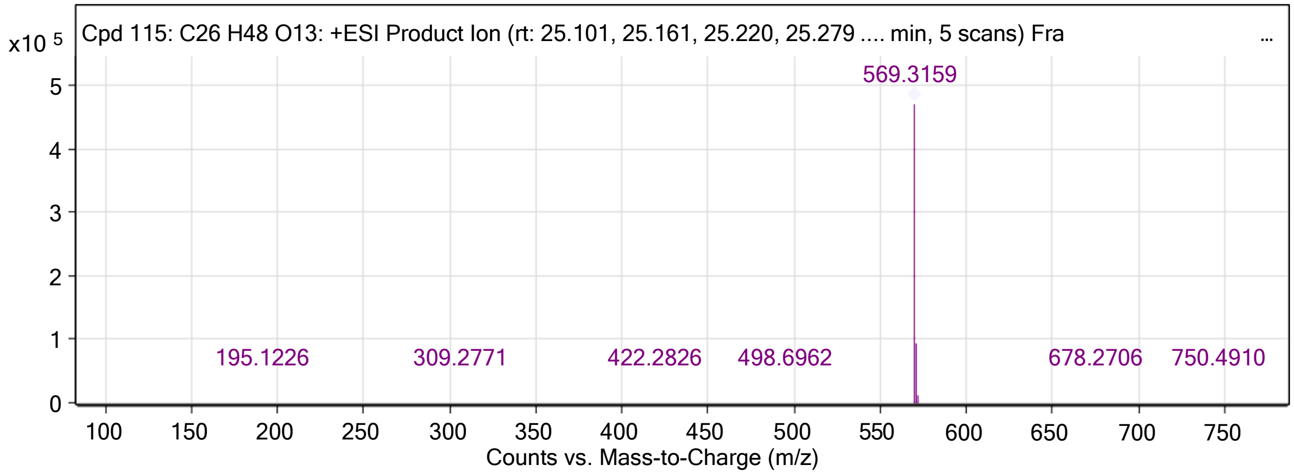

MS/MS Spectrum Peak List

m/z	Calc *m/z*	Diff(ppm)	z	Abund
101.0577	101.0597	20.33		15.38
133.0868	133.0859	-6.49		29.66
177.1113	88.5558	-499999.24	2	28.33
243.1557	243.1591	13.77		25.41
256.1668	256.1669	0.55		15.32
283.2656	283.2632	-8.48		32.32
296.1478	296.1466	-4.27		14.9
309.2771	309.2788	5.6		45.67
311.2947	311.2945	-0.73		17.82
569.3159	569.3168	1.56	1	470105.5

Compound Label	*m/z*	RT	Algorithm	Mass
Cpd 116: C28 H52 O14	613.3437	25.414	Auto MS/MS	612.3362

Compound Chromatograms

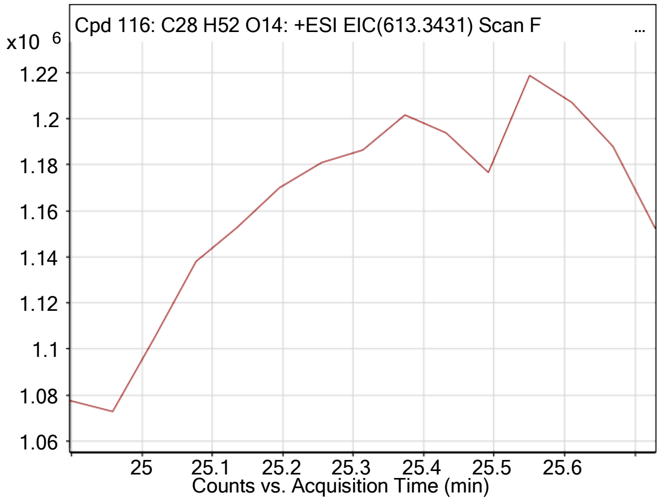

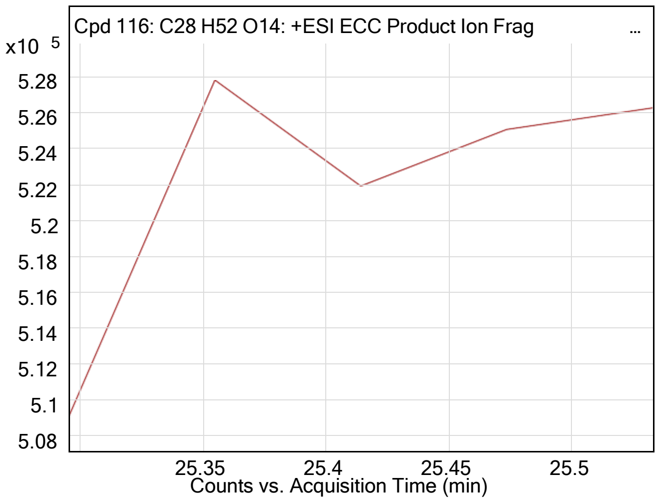

MS Spectrum

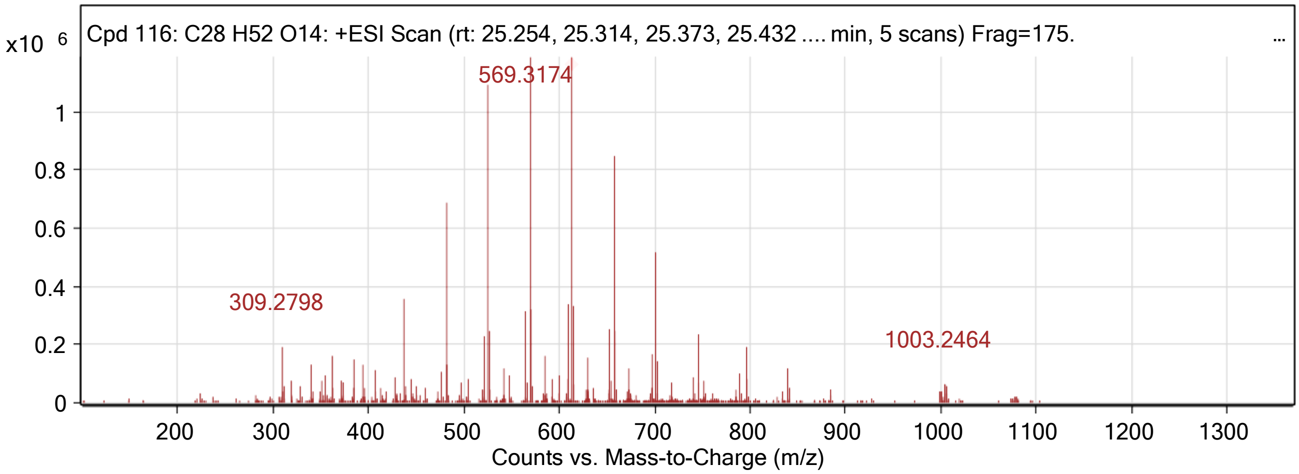

MS Zoomed Spectrum

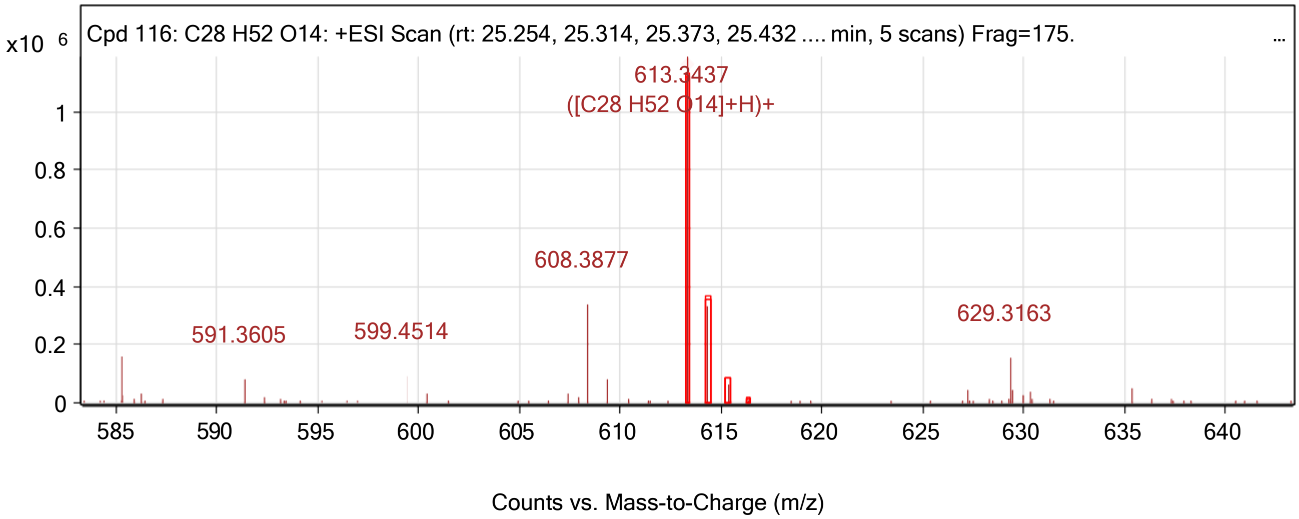

MS Spectrum Peak List

m/z	Calc *m/z*	Diff(ppm)	z	Abund	Formula	Ion
437.2374			1	354569.78		
481.264			1	683443.13		
525.2909			1	1088961.38		
569.3174			1	1333679.75		
613.3437	613.343	-1.22	1	1187919.75	C28 H52 O14	(M+H)+
614.3464	614.3464	0.08	1	332113.06	C28 H52 O14	(M+H)+
615.3482	615.3489	1.01	1	59097.24	C28 H52 O14	(M+H)+
616.3513	616.3515	0.41	1	7974.69	C28 H52 O14	(M+H)+
657.3699			1	846055.13		

MSMS Spectrum

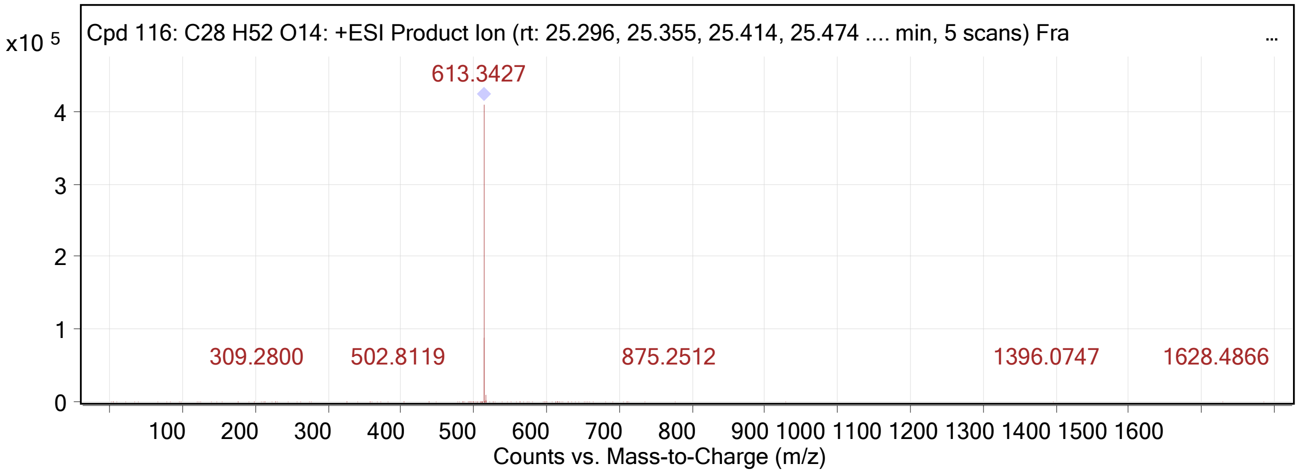

MS/MS Spectrum Peak List

m/z	*Calc m/z*	Diff(ppm)	z	Abund
102.0681	102.0675	-5.65		13.67
103.0437	103.039	-45.88		11.01
133.0867	133.0859	-6.01		26.57
166.1291	166.1352	37.06		11.69
177.1111	177.1121	5.79		15.81
222.9528	222.9568	17.92		13.02
309.28	309.2788	-3.88		113.76
311.2947	311.2945	-0.82	1	76.29
609.3041	304.6556	-499994.22	2	102.48
613.3427	613.343	0.52	1	409500.5

Compound Label	*m/z*	RT	Algorithm	Mass
Cpd 117: C26 H48 O13	569.3174	25.516	Auto MS/MS	568.3099

Compound Chromatograms

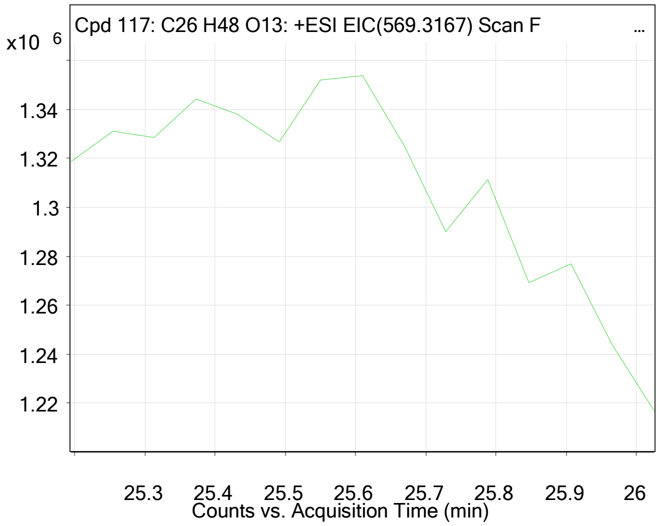

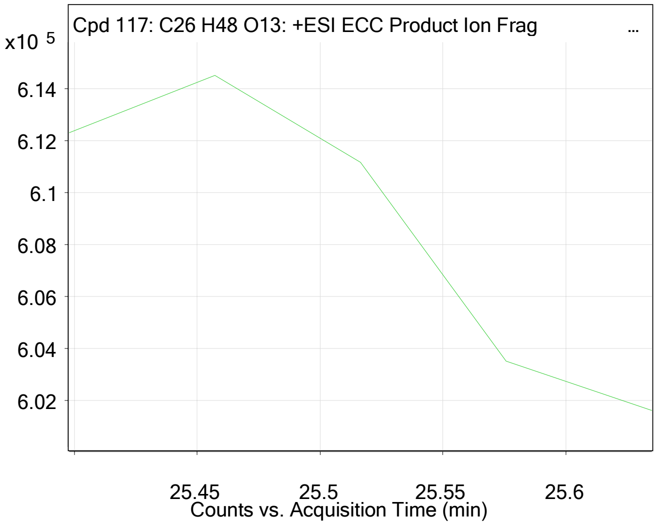

MS Spectrum

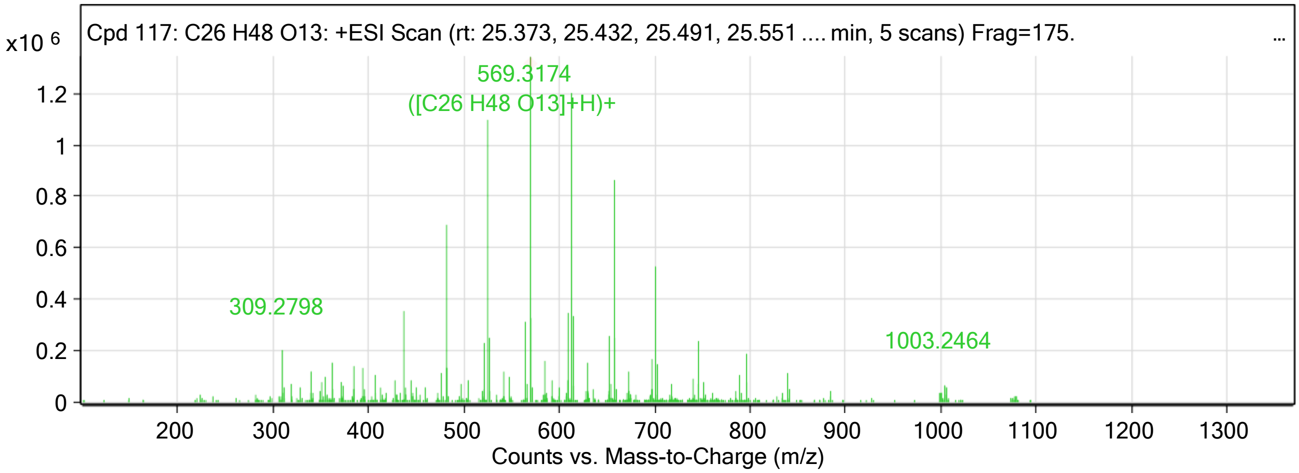

MS Zoomed Spectrum

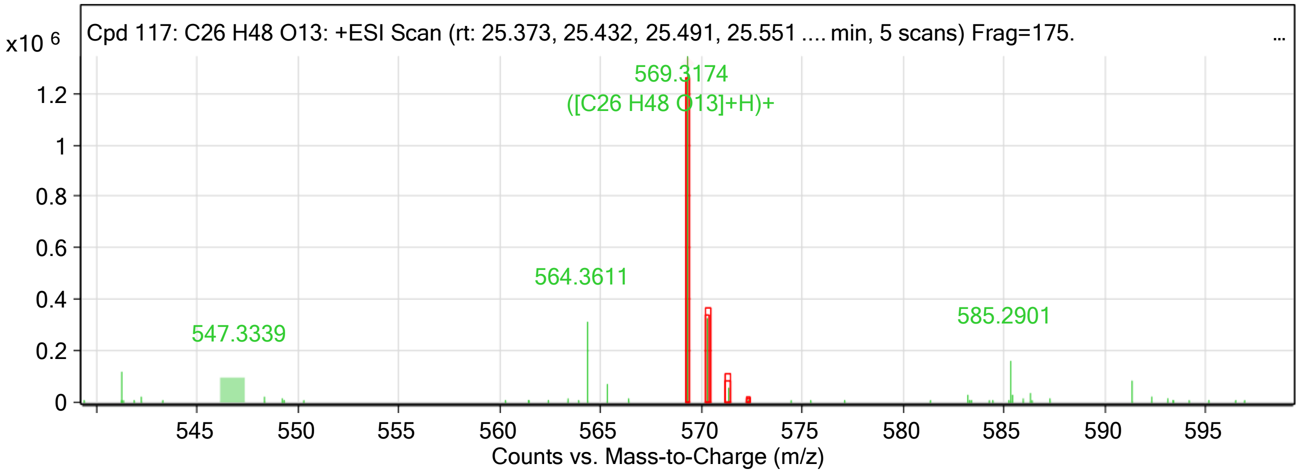

MS Spectrum Peak List

m/z	Calc *m/z*	Diff(ppm)	z	Abund	Formula	Ion
437.2374			1	354562.94		
481.264			1	683631.38		
525.2909			1	1095636.88		
569.3174	569.3168	-1.1	1	1342884.13	C26 H48 O13	(M+H)+
570.3199	570.3202	0.46	1	322300.56	C26 H48 O13	(M+H)+
571.322	571.3226	1.04	1	55922.95	C26 H48 O13	(M+H)+
572.3243	572.3253	1.7	1	7129.65	C26 H48 O13	(M+H)+
613.3436			1	1199636.75		
657.3699			1	857598.31		
701.3959			1	523610.19		

MSMS Spectrum

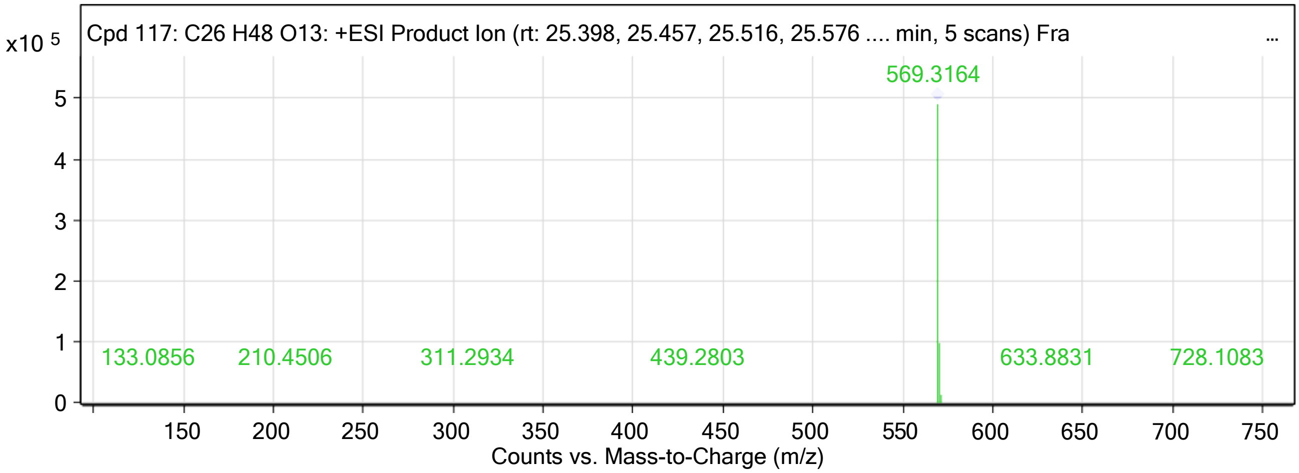

MS/MS Spectrum Peak List

m/z	*Calc m/z*	Diff(ppm)	z	Abund
133.0856	133.0859	2.67		76.77
144.0774	144.0781	5.14		19.74
283.2622	283.2632	3.52		35.56
307.218	307.2115	-21.04		20.57
309.2784	309.2788	1.18		26.51
311.2934	311.2945	3.51		52.51
338.2245	338.2299	16.05		11.42
494.279	494.2722	-13.93		11.16
545.323	545.3168	-11.39		12.05
569.3164	569.3168	0.72	1	490164.84

Compound Label	*m/z*	RT	Algorithm	Mass
Cpd 118: C28 H52 O14	613.3433	25.711	Auto MS/MS	612.3359

Compound Chromatograms

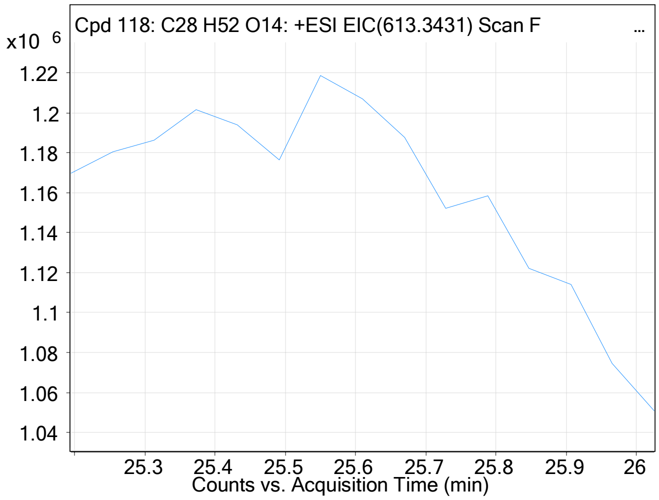

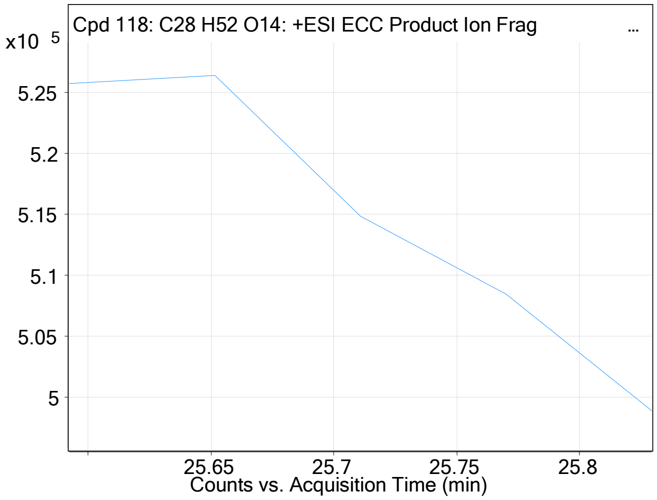

MS Spectrum

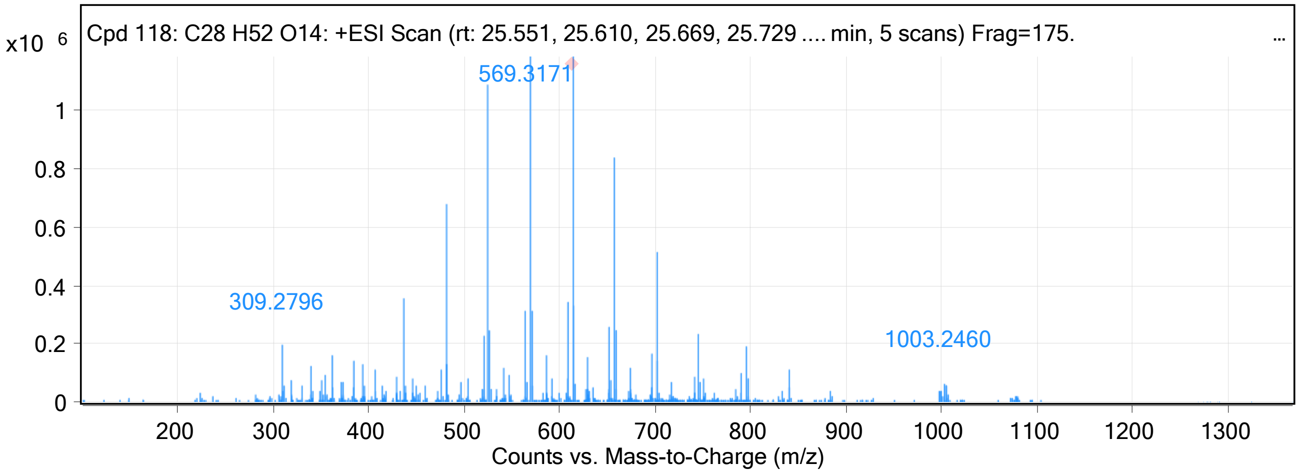

MS Zoomed Spectrum

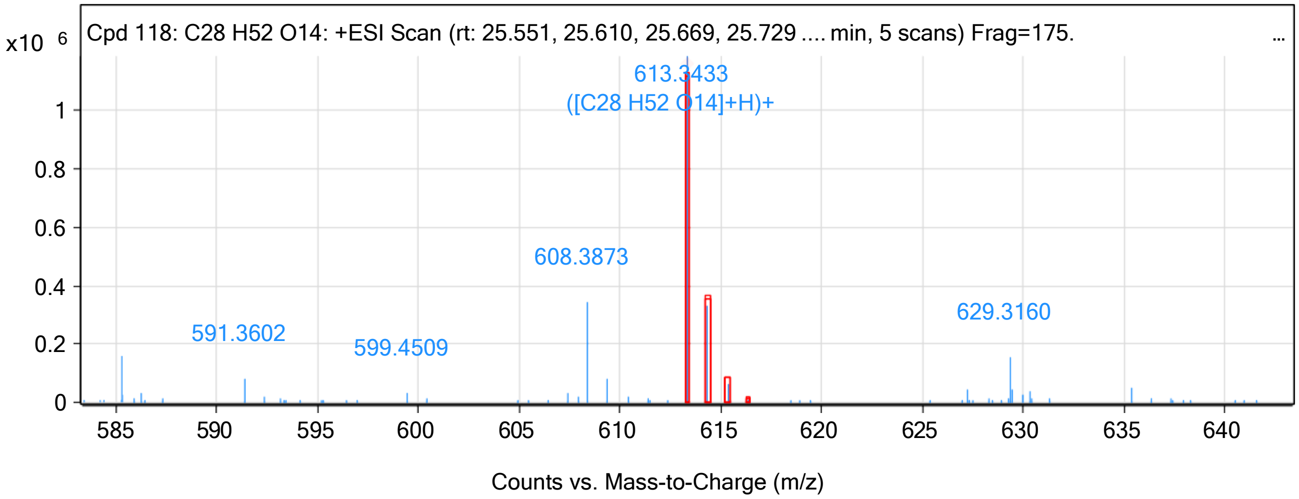

MS Spectrum Peak List

m/z	Calc *m/z*	Diff(ppm)	z	Abund	Formula	Ion
437.2373			1	352945.34		
481.2638			1	675664.25		
525.2906			1	1087135.38		
569.3171			1	1326340.38		
613.3433	613.343	-0.59	1	1184993	C28 H52 O14	(M+H)+
614.3461	614.3464	0.56	1	329789.66	C28 H52 O14	(M+H)+
615.348	615.3489	1.37	1	60031.39	C28 H52 O14	(M+H)+
616.3508	616.3515	1.18	1	8159.17	C28 H52 O14	(M+H)+
657.3696			1	839727.19		
701.3955			1	513441.06		

MSMS Spectrum

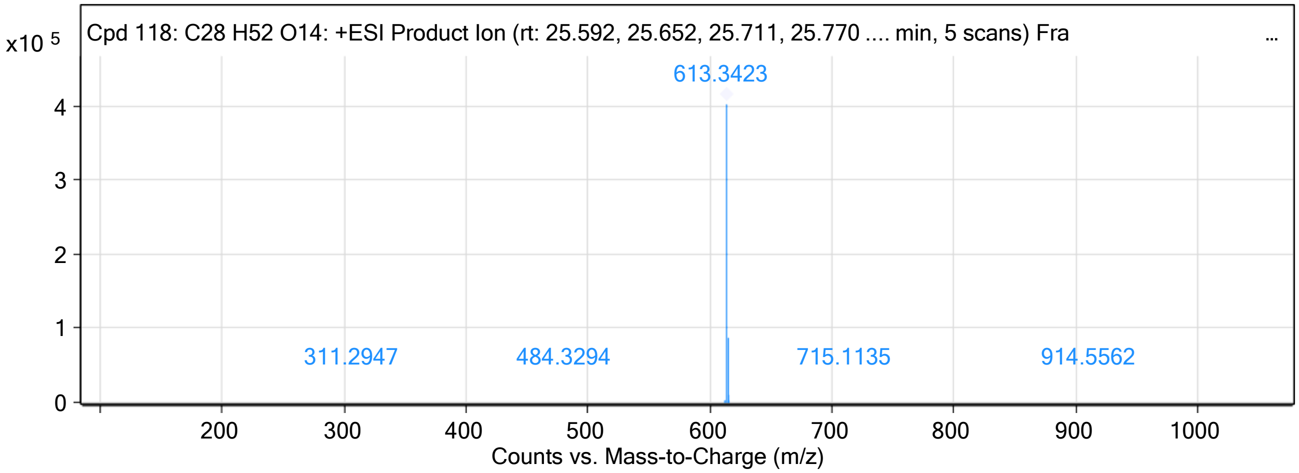

MS/MS Spectrum Peak List

m/z	Calc *m/z*	Diff(ppm)	z	Abund
133.0863	133.0859	-2.69		65.09
146.0941	146.0937	-2.42		12.58
152.0993	152.1043	33.12		12.91
263.1877	263.1853	-9.24		12.77
265.17	265.1646	-20.52		13.16
309.2771	309.2788	5.38		48.87
311.2947	311.2945	-0.72	1	109.95
357.0632	357.0664	8.93		15.24
560.3487	560.3402	-15.15		13.22
613.3423	613.343	1.14	1	402697.84

Compound Label	*m/z*	RT	Algorithm	Mass
Cpd 119: C26 H48 O13	569.3171	25.813	Auto MS/MS	568.3096

Compound Chromatograms

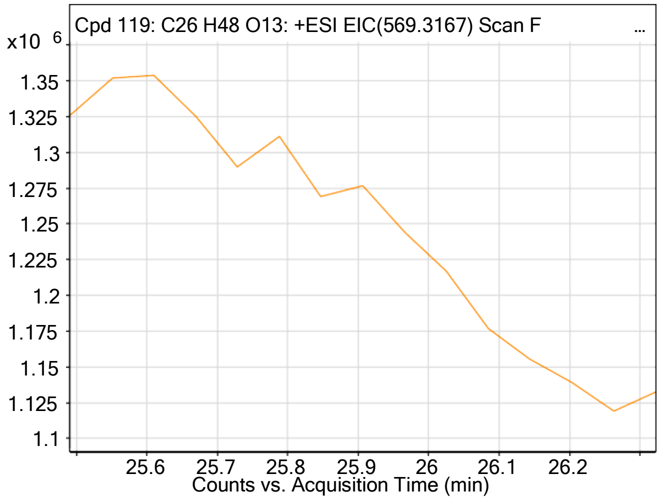

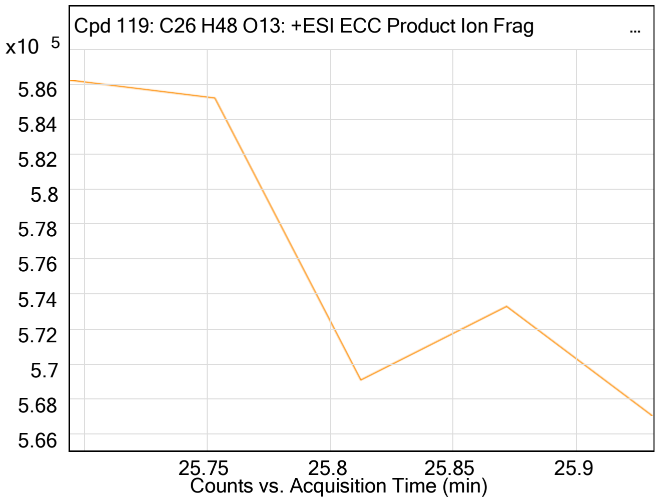

MS Spectrum

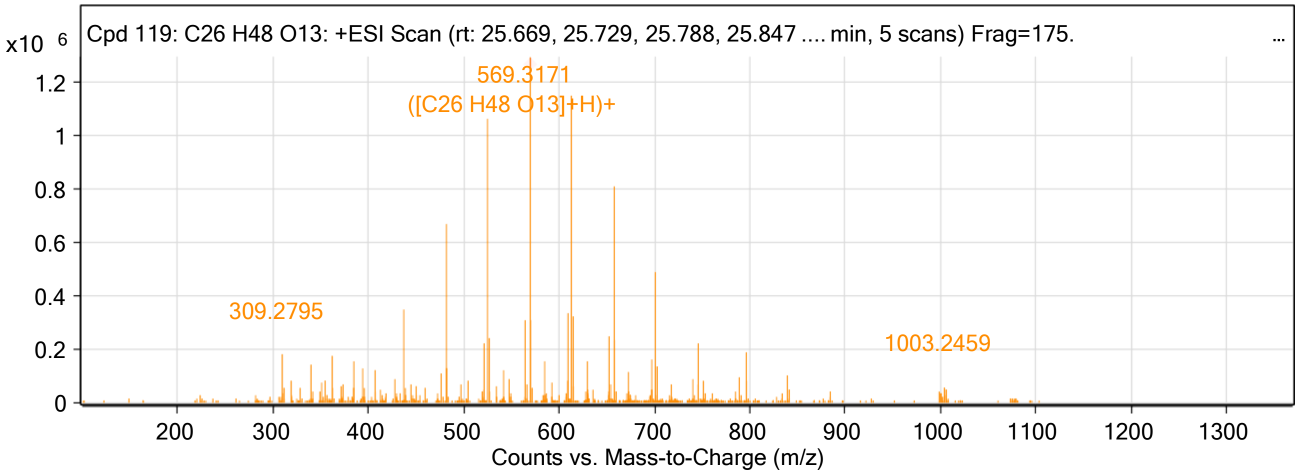

MS Zoomed Spectrum

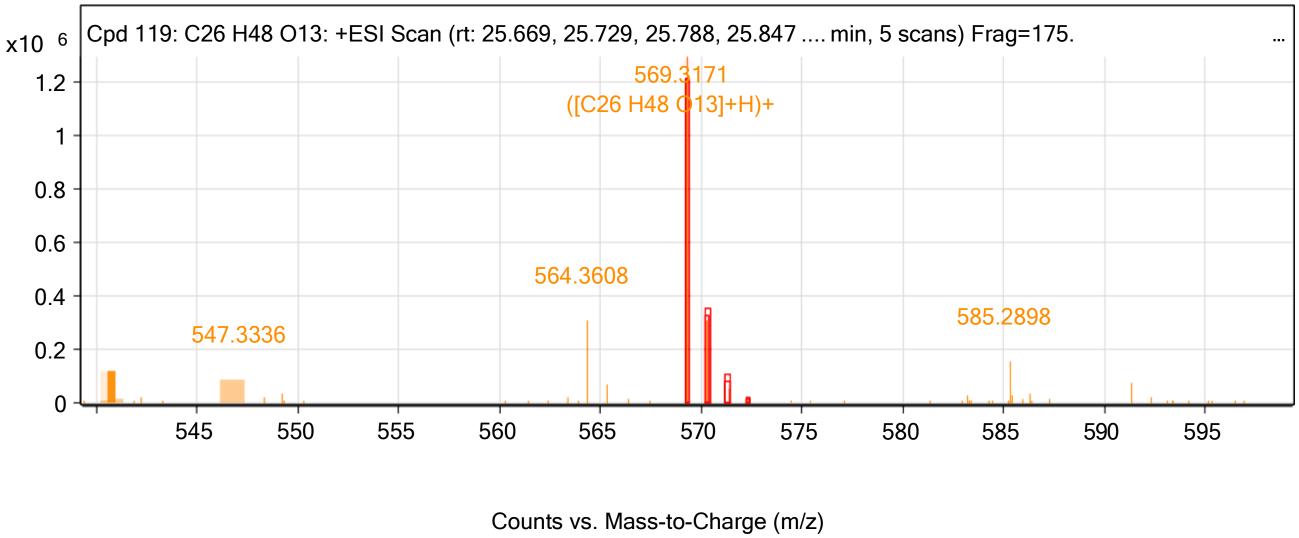

MS Spectrum Peak List

m/z	Calc *m/z*	Diff(ppm)	z	Abund	Formula	Ion
437.2372			1	350042.69		
481.2637			1	666751.69		
525.2906			1	1063187.63		
569.3171	569.3168	-0.6	1	1294442.63	C26 H48 O13	(M+H)+
570.3196	570.3202	1.02	1	306411.5	C26 H48 O13	(M+H)+
571.3216	571.3226	1.66	1	54450.14	C26 H48 O13	(M+H)+
572.3242	572.3253	1.84	1	7115.66	C26 H48 O13	(M+H)+
613.3434			1	1147111.25		
657.3695			1	810197.13		

MSMS Spectrum

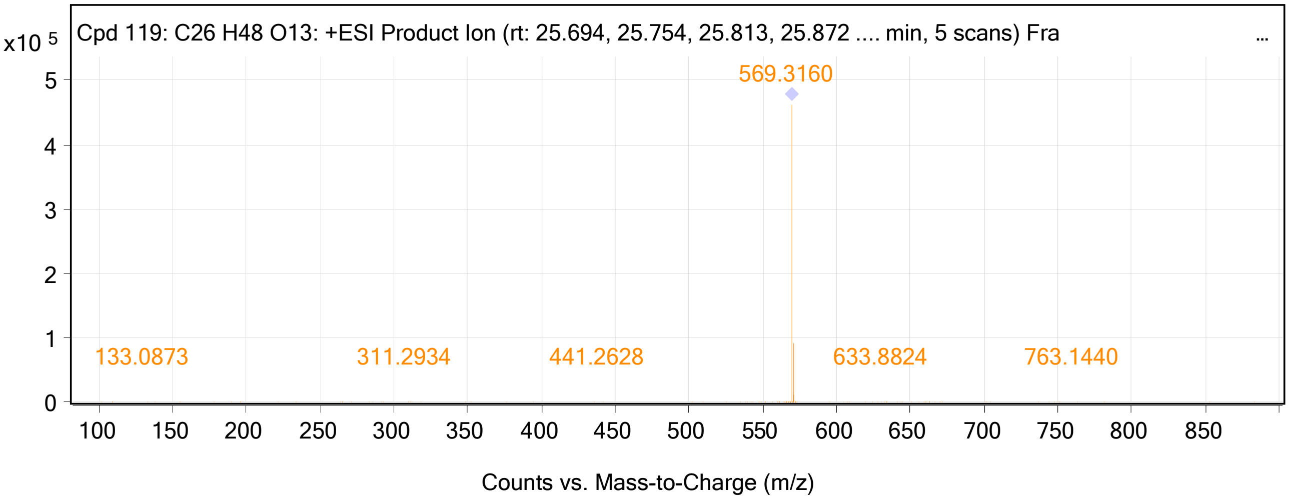

MS/MS Spectrum Peak List

m/z	*Calc m/z*	Diff(ppm)	z	Abund
133.0873	133.0859	-10.3		31.91
177.1117	177.1121	2.49		21.71
190.1223	190.12	-12.36		11.46
233.173	233.1747	7.25		16.35
285.2101	285.206	-14.27		12.18
291.2124	291.2166	14.28		13.8
309.2802	309.2788	-4.37		33.06
311.2934	311.2945	3.25		45.59
441.2628	441.2694	14.99		12.61
569.316	569.3168	1.4	1	462977.81

Compound Label	*m/z*	RT	Algorithm	Mass
Cpd 120: C28 H52 O14	613.3432	26.007	Auto MS/MS	612.3357

Compound Chromatograms

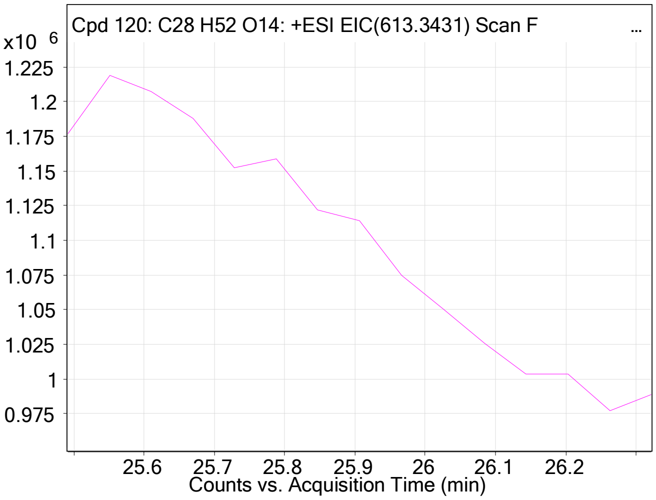

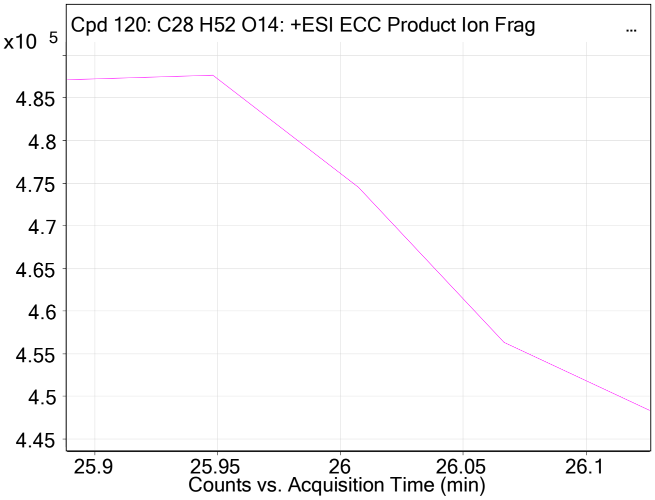

MS Spectrum

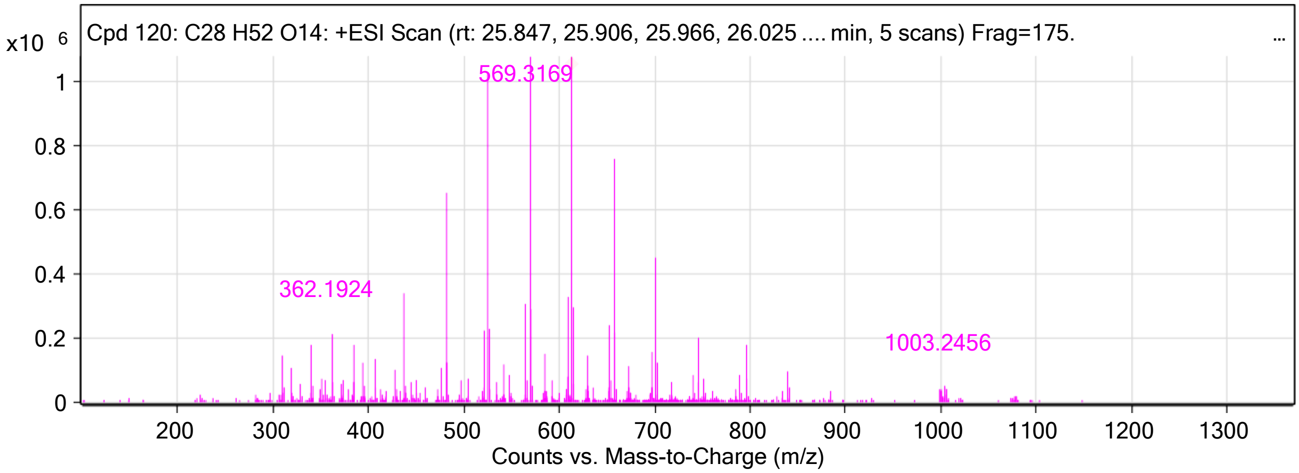

MS Zoomed Spectrum

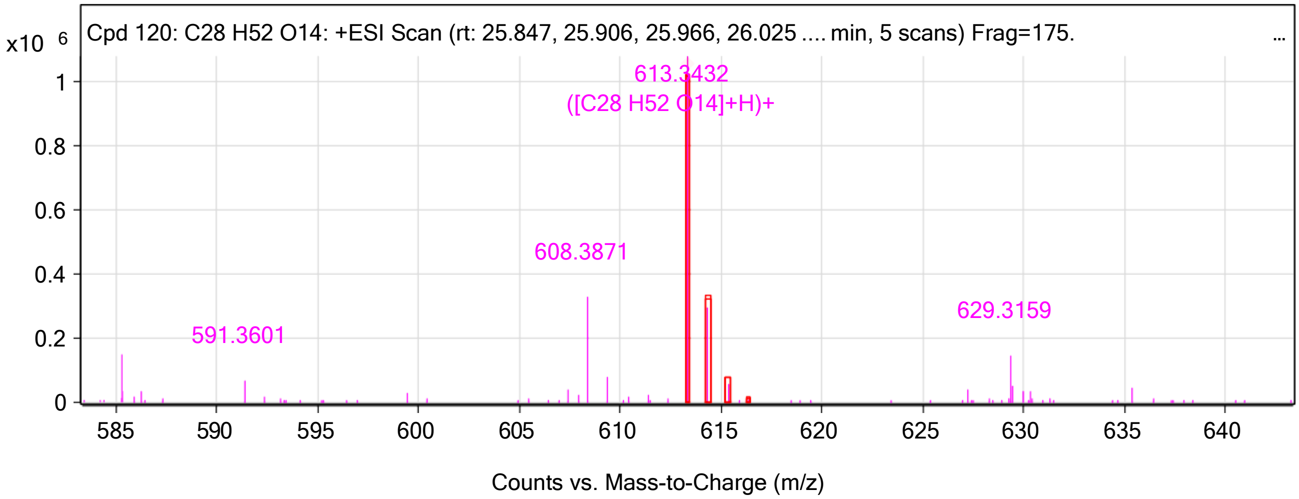

MS Spectrum Peak List

m/z	Calc *m/z*	Diff(ppm)	z	Abund	Formula	Ion
437.2371			1	340249.97		
481.2636			1	650982.38		
525.2905			1	1021762		
569.3169			1	1236800.25		
613.3432	613.343	-0.34	1	1077428.63	C28 H52 O14	(M+H)+
614.3458	614.3464	0.99	1	297382.75	C28 H52 O14	(M+H)+
615.3477	615.3489	1.83	1	54056.3	C28 H52 O14	(M+H)+
616.351	616.3515	0.81	1	7180.11	C28 H52 O14	(M+H)+
657.3693			1	754913.56		
701.3953			1	449836		

MSMS Spectrum

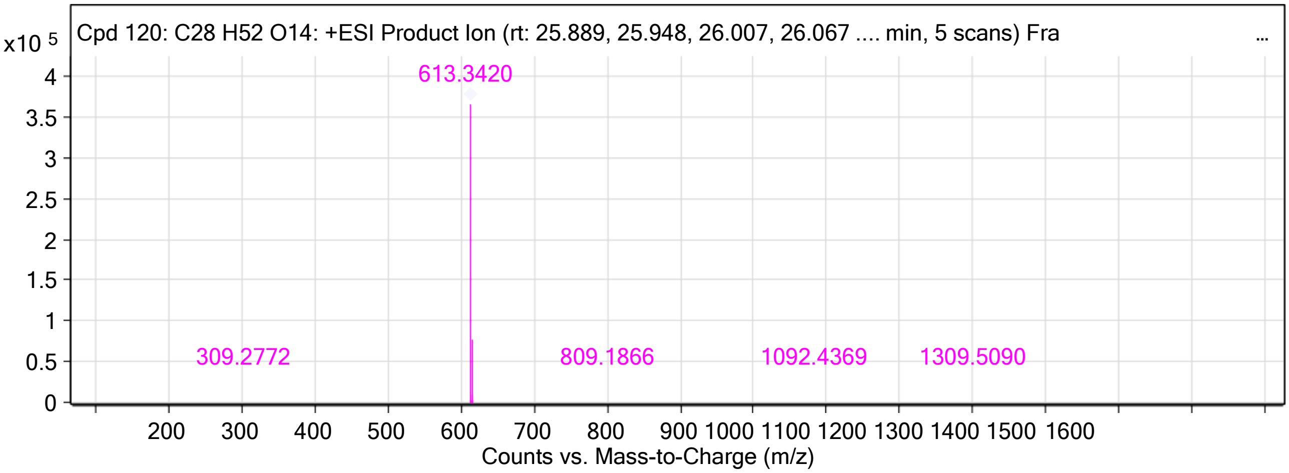

MS/MS Spectrum Peak List

m/z	Calc m/z	Diff(ppm)	z	Abund
133.0862	133.0859	-2.42		25.74
198.1983	198.1978	-2.39		21.44
274.2134	274.2139	1.52		15.43
309.2772	309.2788	5.27	1	69.37
357.0665	357.0664	-0.41		35.3
612.3938			2	2640.78
613.342	613.343	1.66	1	365777.06
614.3446			1	76742.71
615.3467			1	9115.77
615.4169			2	1815.31

Compound Label	m/z	RT	Algorithm	Mass
Cpd 121: C26 H48 O13	569.317	26.109	Auto MS/MS	568.3095

Compound Chromatograms

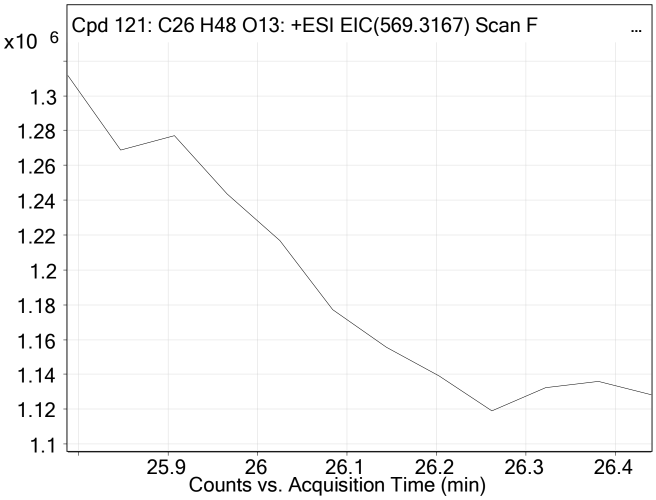

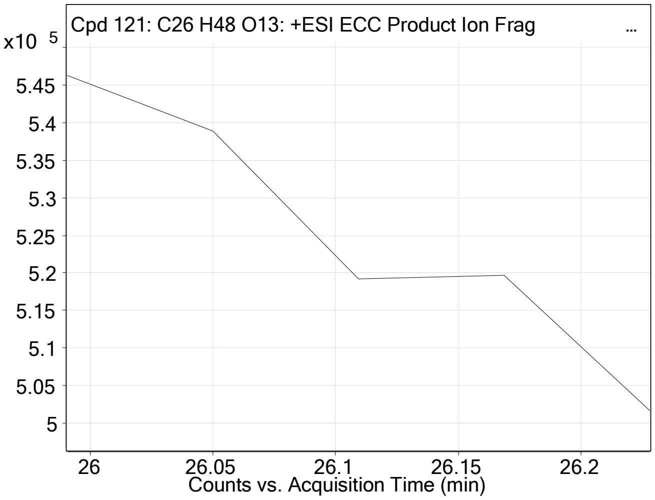

MS Spectrum

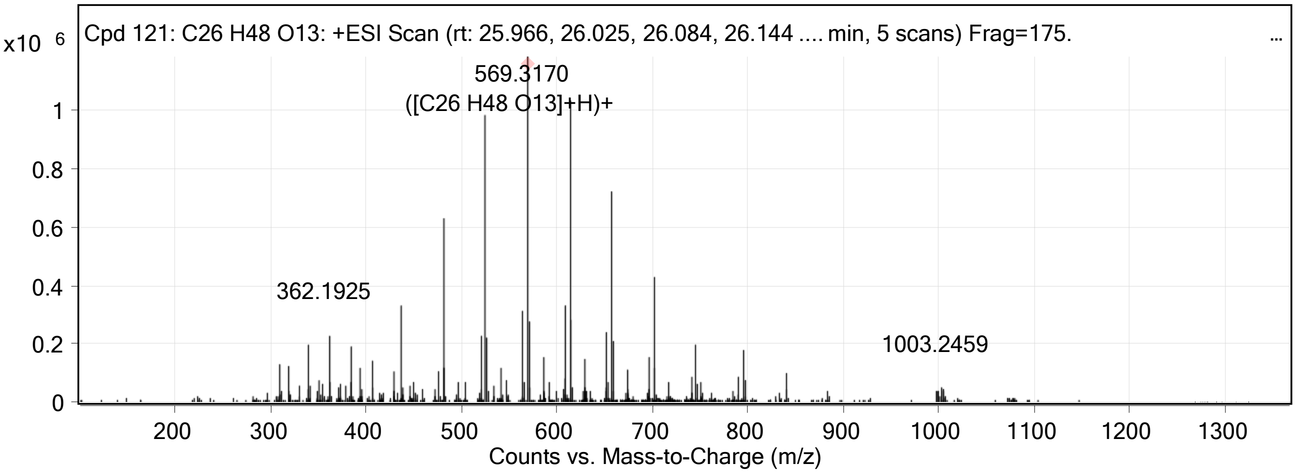

MS Zoomed Spectrum

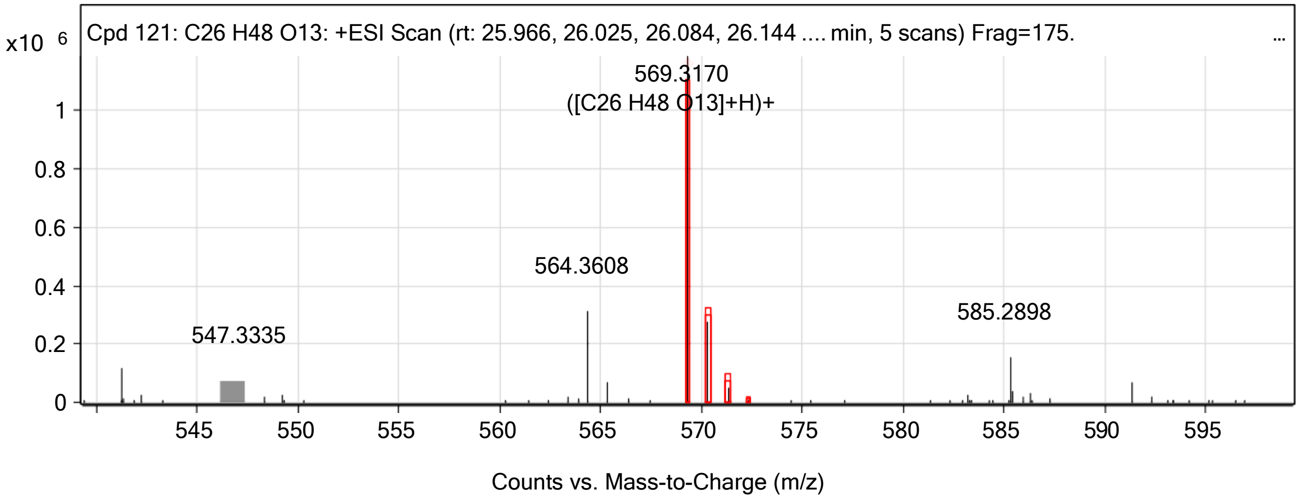

MS Spectrum Peak List

m/z	Calc *m/z*	Diff(ppm)	z	Abund	Formula	Ion
481.2637			1	633336.31		
525.2906			1	983876.88		
569.317	569.3168	-0.4	1	1186524.5	C26 H48 O13	(M+H)+
570.3195	570.3202	1.13	1	278502.31	C26 H48 O13	(M+H)+
571.3215	571.3226	1.83	1	48579.35	C26 H48 O13	(M+H)+
572.3243	572.3253	1.8	1	6730.81	C26 H48 O13	(M+H)+
608.3873			1	331857.44		
613.3433			1	1031696.5		
657.3695			1	719597.56		
701.3954			1	430183.59		

MSMS Spectrum

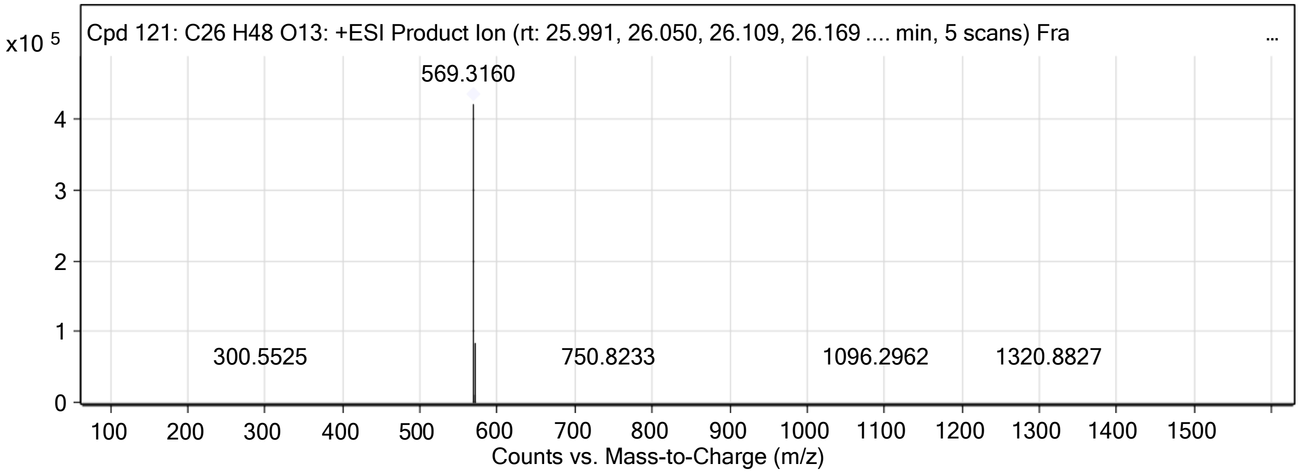

MS/MS Spectrum Peak List

m/z	Calc *m/z*	Diff(ppm)	z	Abund
117.0908	117.091	1.45		15.02
133.085	133.0859	7.14		39.47
265.2491	265.2526	13.31		19
309.2766	309.2788	7.28	1	26.5
438.2743	438.2823	18.41		14.51
566.2885	566.2933	8.38		48.8
569.316	569.3168	1.34	1	421506.81
570.3187			1	82221.21
571.3207			1	9931.07
571.3907			2	890.46

Compound Label	*m/z*	RT	Algorithm	Mass
Cpd 122: C28 H52 O14	613.3437	26.215	Auto MS/MS	612.3362

Compound Chromatograms

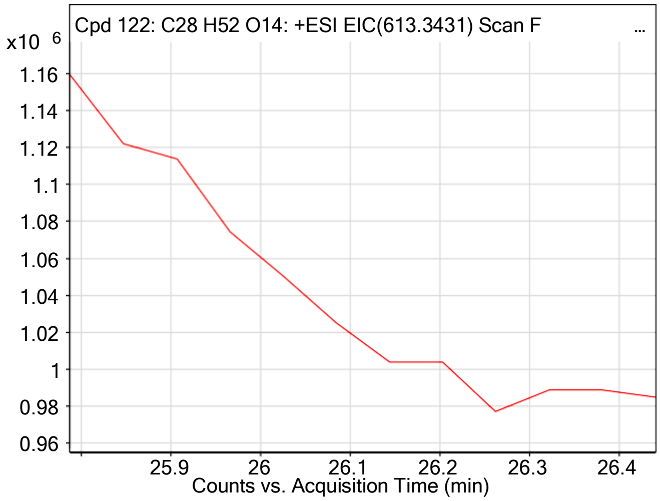

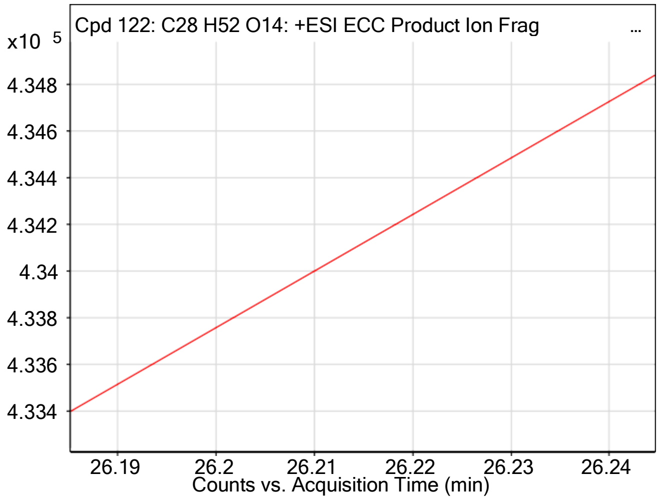

MS Spectrum

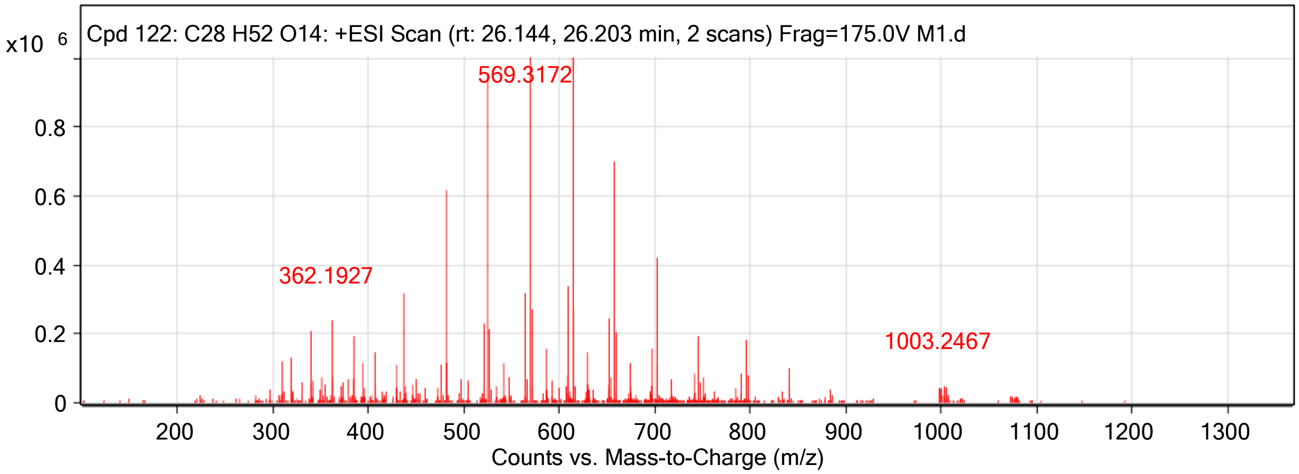

MS Zoomed Spectrum

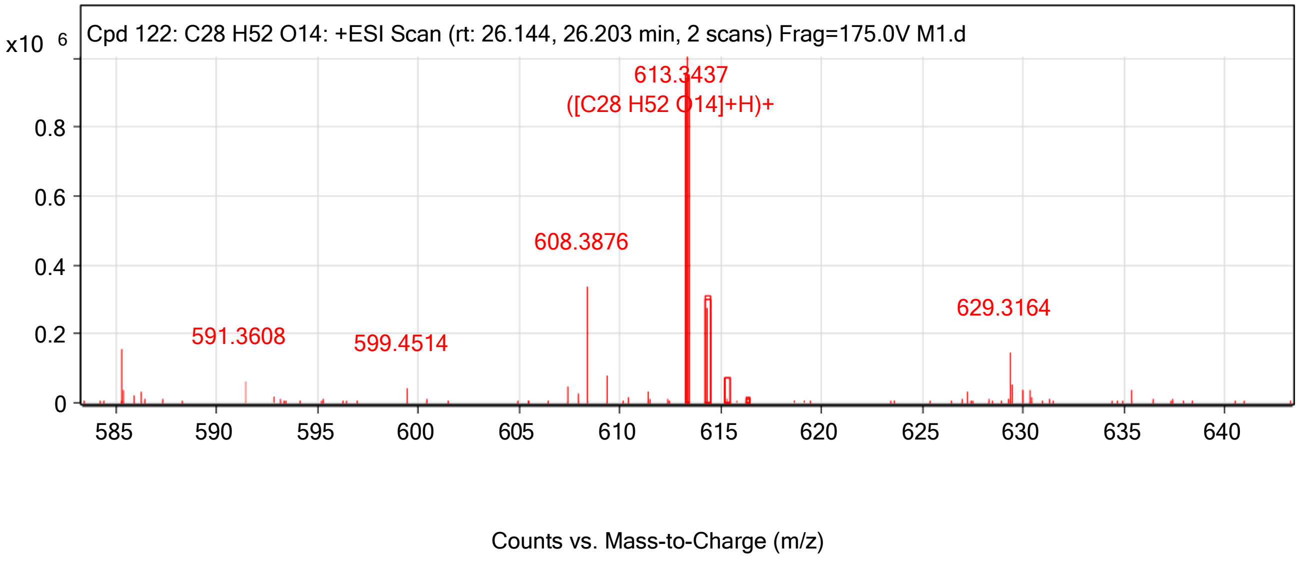

MS Spectrum Peak List

m/z	Calc *m/z*	Diff(ppm)	z	Abund	Formula	Ion
481.264			1	613487.44		
525.2909			1	950788.25		
569.3172			1	1147327.25		
608.3876			1	336996.56		
613.3437	613.343	-1.24	1	1003813.13	C28 H52 O14	(M+H)+
614.3463	614.3464	0.15	1	275381.16	C28 H52 O14	(M+H)+
615.3482	615.3489	1.06	1	48801.38	C28 H52 O14	(M+H)+
616.3531	616.3515	-2.57	1	7017.49	C28 H52 O14	(M+H)+
657.3699			1	699093		

MSMS Spectrum

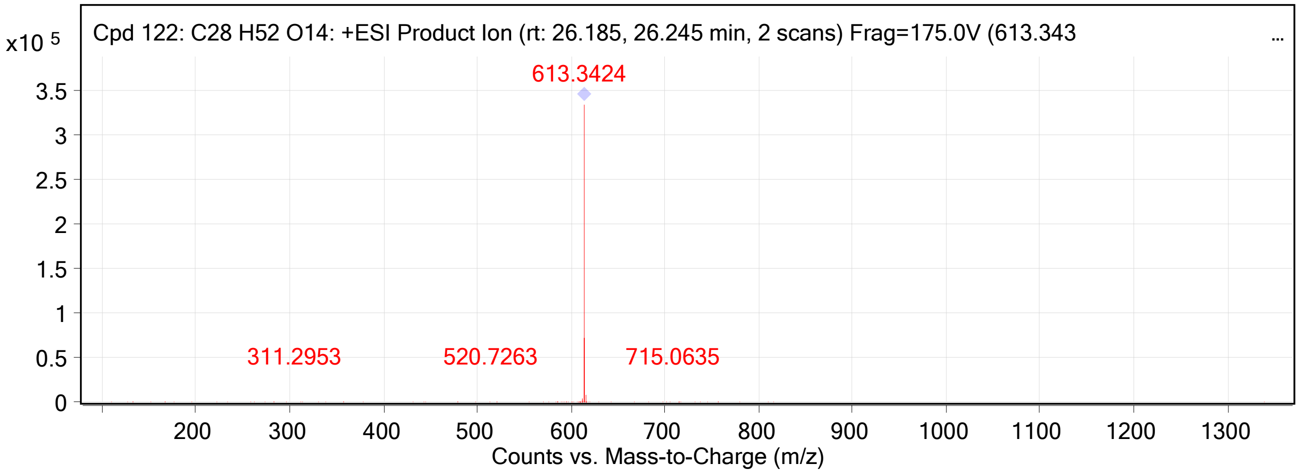

MS/MS Spectrum Peak List

m/z	*Calc m/z*	Diff(ppm)	z	Abund
133.0867	133.0859	-5.79		125.26
177.1137	177.1121	-9.01		24.09
283.2634	283.2632	-0.7		47.26
296.1509	296.1466	-14.57		30.75
311.2953	311.2945	-2.59		59.58
312.2993	312.3023	9.67		44.84
357.0705	357.0664	-11.49		32.03
576.3396	576.3352	-7.7		27.13
585.3526	585.3481	-7.73		34.3
613.3424	613.343	1.03	1	334551.34

Compound Label	*m/z*	RT	Algorithm	Mass
Cpd 123: C26 H48 O13	569.3169	26.406	Auto MS/MS	568.3095

Compound Chromatograms

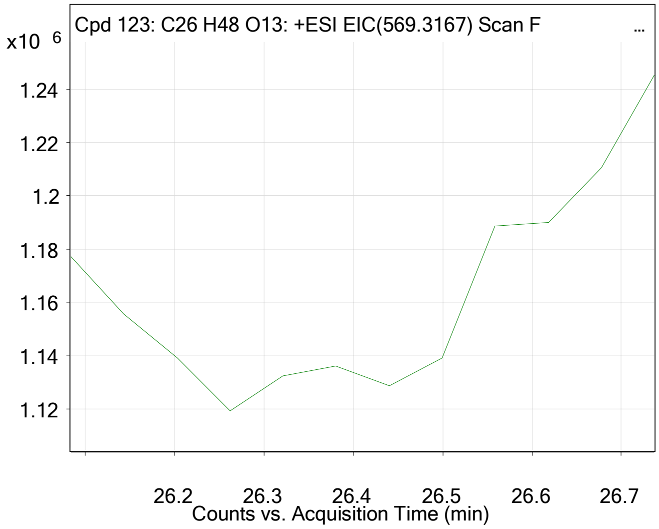

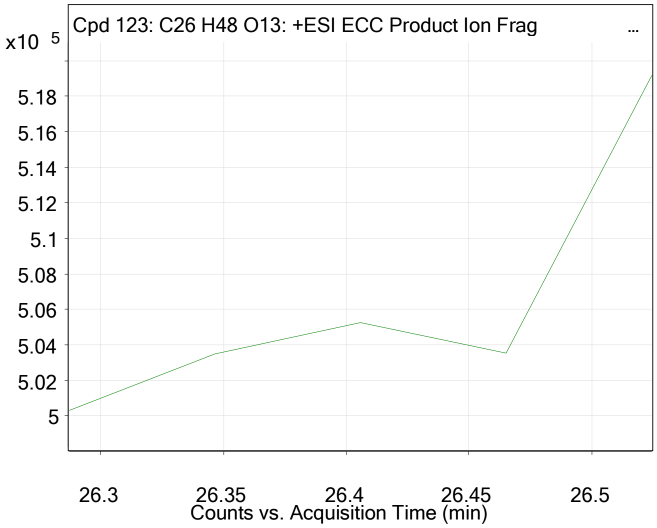

MS Spectrum

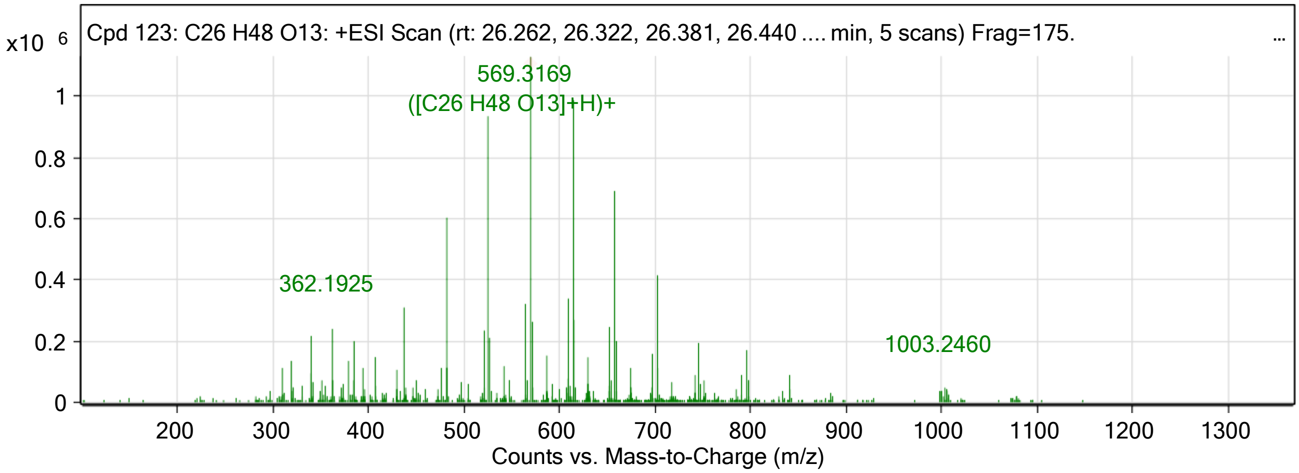

MS Zoomed Spectrum

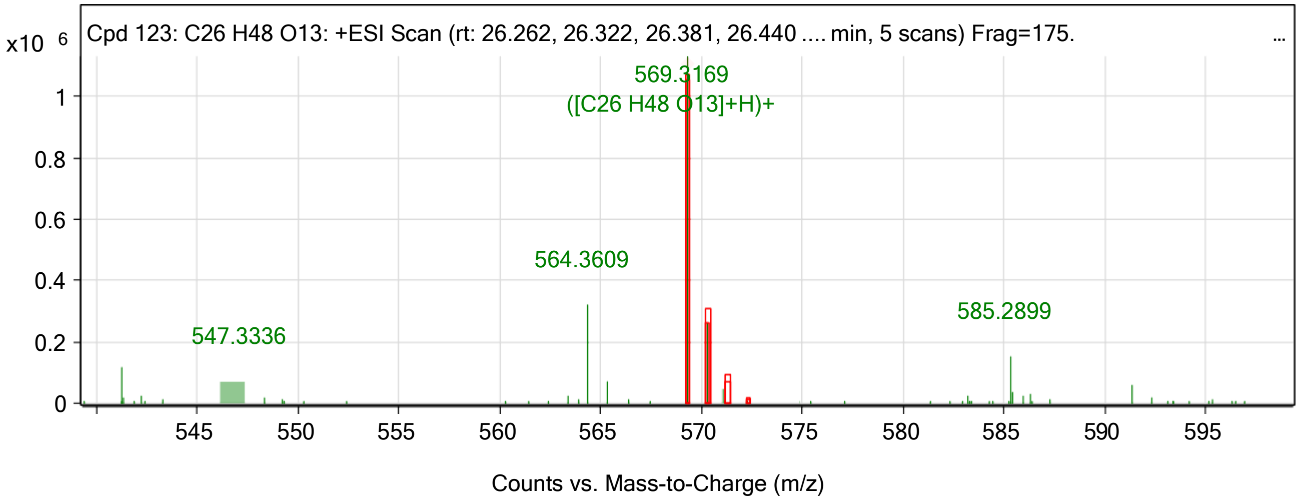

MS Spectrum Peak List

m/z	Calc *m/z*	Diff(ppm)	z	Abund	Formula	Ion
481.2637			1	597981.31		
525.2906			1	934187.5		
569.3169	569.3168	-0.26	1	1131102.75	C26 H48 O13	(M+H)+
570.3196	570.3202	1.03	1	265065.81	C26 H48 O13	(M+H)+
571.3216	571.3226	1.7	1	45618.41	C26 H48 O13	(M+H)+
572.3243	572.3253	1.65	1	5939.04	C26 H48 O13	(M+H)+
608.3874			1	339823.16		
613.3434			1	986910.19		
657.3696			1	686939.19		
701.3955			1	415200.47		

MSMS Spectrum

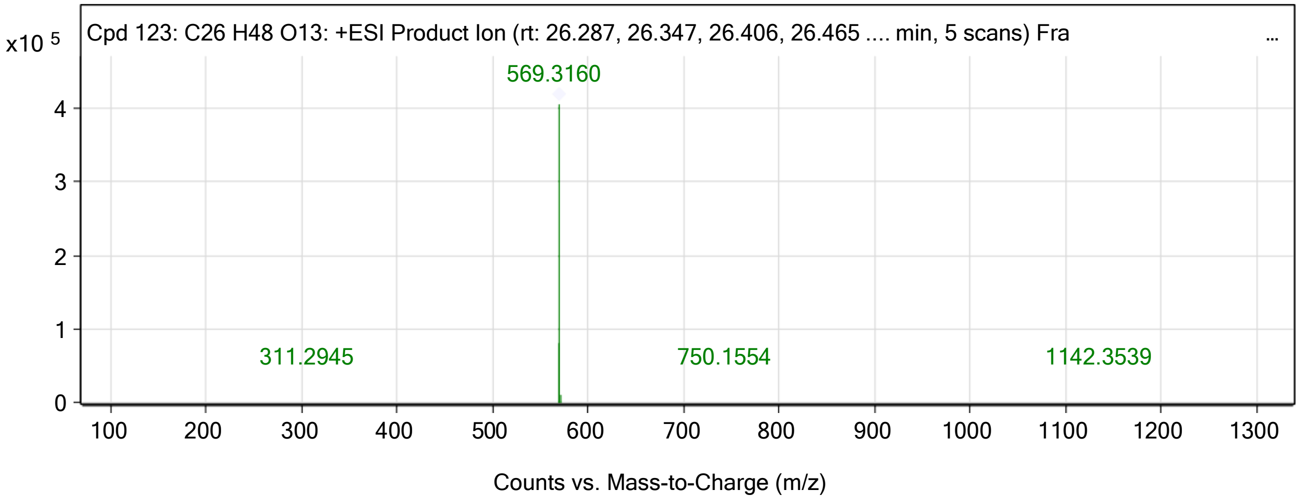

MS/MS Spectrum Peak List

m/z	*Calc m/z*	Diff(ppm)	z	Abund
133.0873	133.0859	-10.25		32.29
210.1145	210.1098	-22.2		26.96
311.2945	311.2945	-0.11		58.24
387.2345	387.2377	8.33		15.94
538.2935	538.2984	9.03		17.22
568.3599			2	701.61
569.316	569.3168	1.31	1	405325.31
570.3186			1	79567.23
571.321			1	9623.7
571.3904			2	949.4

Compound Label	*m/z*	RT	Algorithm	Mass
Cpd 124: C24 H44 O12	525.2906	26.422	Auto MS/MS	524.2832

Compound Chromatograms

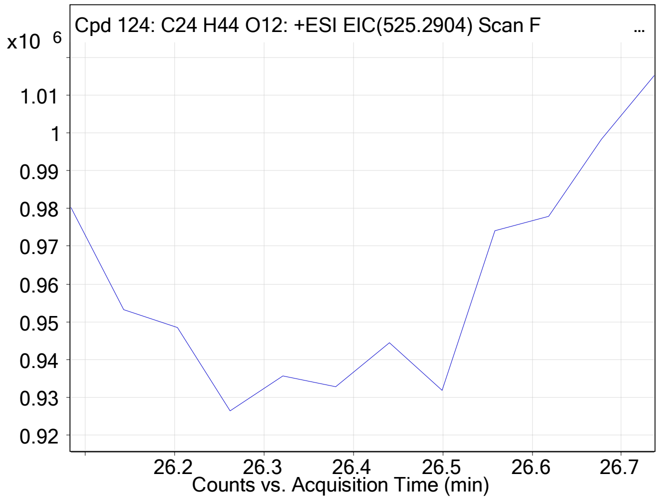

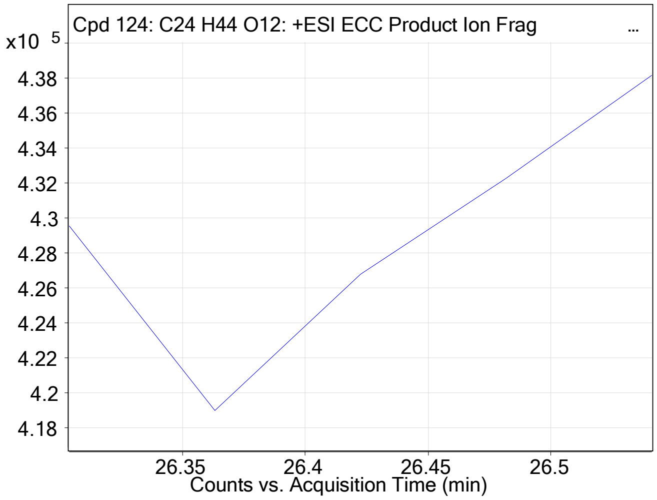

MS Spectrum

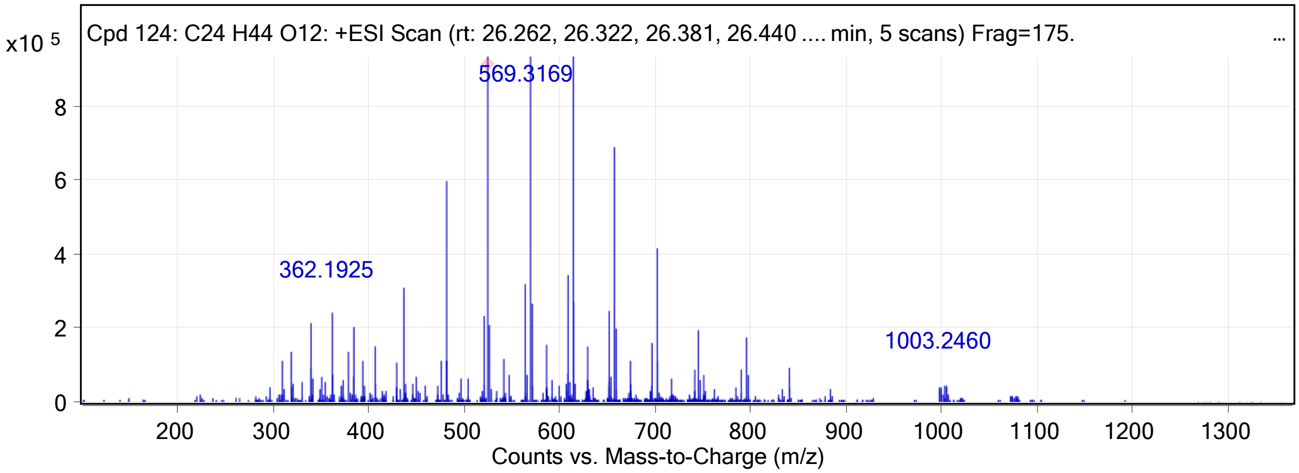

MS Zoomed Spectrum

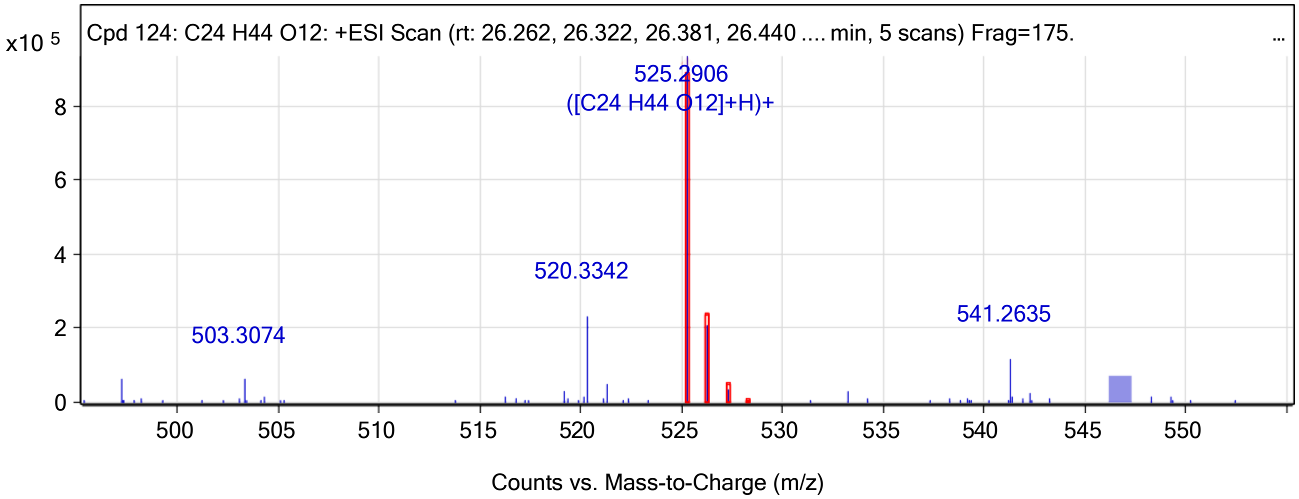

MS Spectrum Peak List

m/z	Calc *m/z*	Diff(ppm)	z	Abund	Formula	Ion
481.2637			1	597981.31		
525.2906	525.2906	-0.16	1	934187.5	C24 H44 O12	(M+H)+
526.2933	526.294	1.35	1	209124.75	C24 H44 O12	(M+H)+
527.2957	527.2963	1.2	1	34684	C24 H44 O12	(M+H)+
528.2985	528.299	0.9	1	4650.25	C24 H44 O12	(M+H)+
569.3169			1	1131102.75		
608.3874			1	339823.16		
613.3434			1	986910.19		
657.3696			1	686939.19		
701.3955			1	415200.47		

MSMS Spectrum

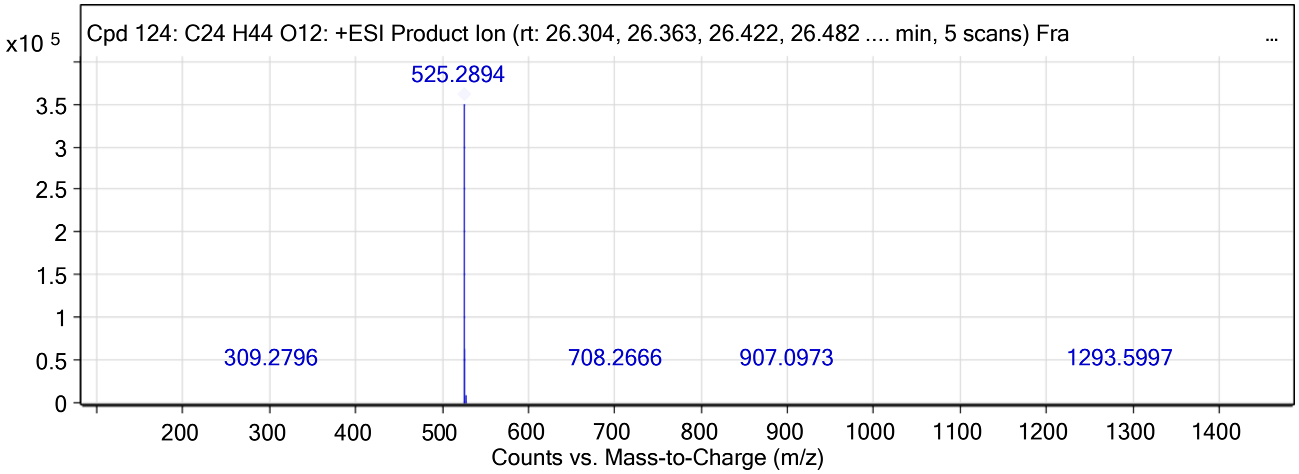

MS/MS Spectrum Peak List

m/z	Calc *m/z*	Diff(ppm)	z	Abund
117.0909	117.091	1.25		11.69
133.0864	133.0859	-3.84		36.05
166.0897	166.0836	-37.02		22.36
232.1326	232.1305	-8.84		10.95
292.2176	292.2244	23.46		8.65
309.2796	309.2788	-2.71		42.98
311.2947	311.2945	-0.89		8.62
372.2188	372.2143	-12.17		10
494.2736	494.2722	-2.93		23.87
525.2894	525.2906	2.15	1	349515.31

Compound Label	*m/z*	RT	Algorithm	Mass
Cpd 125: C26 H48 O13	569.317	26.702	Auto MS/MS	568.3096

Compound Chromatograms

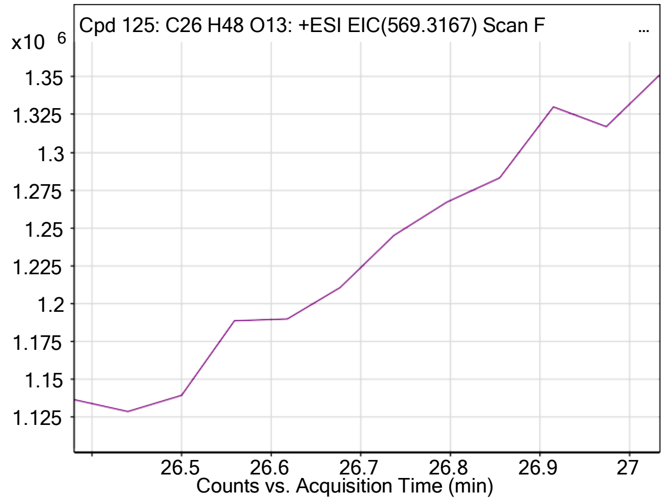

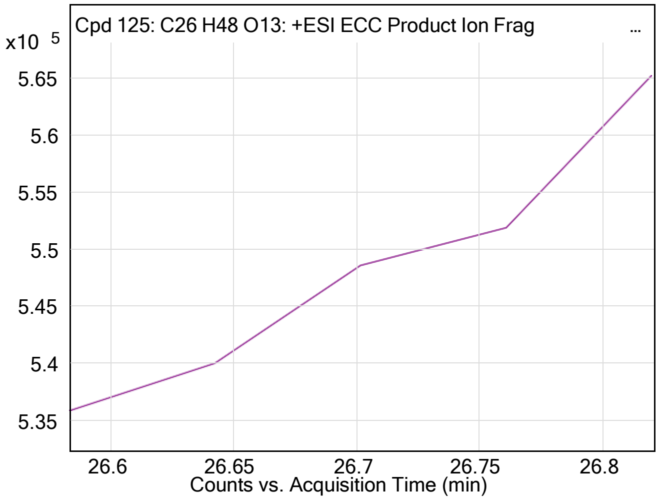

MS Spectrum

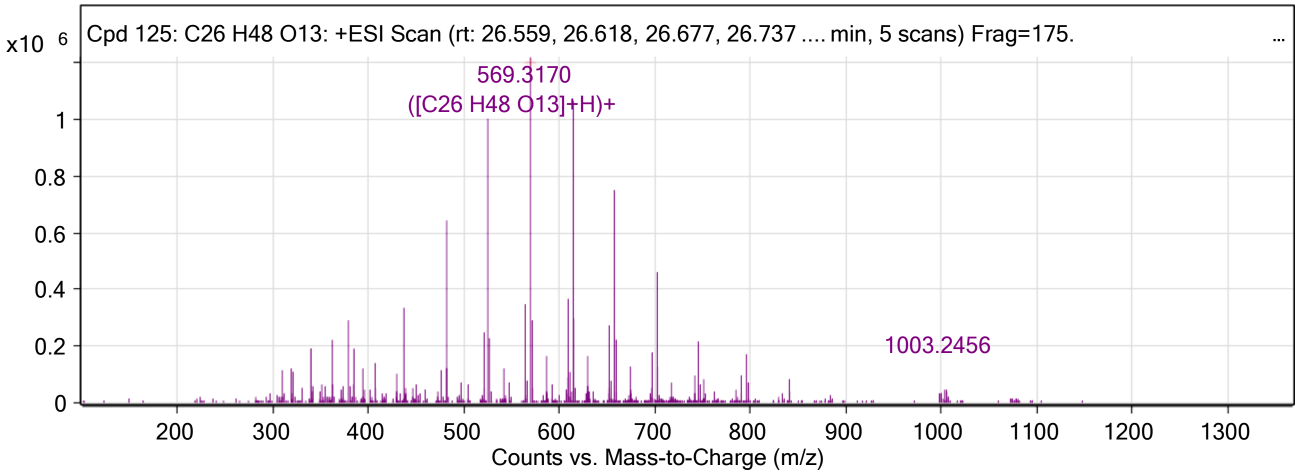

MS Zoomed Spectrum

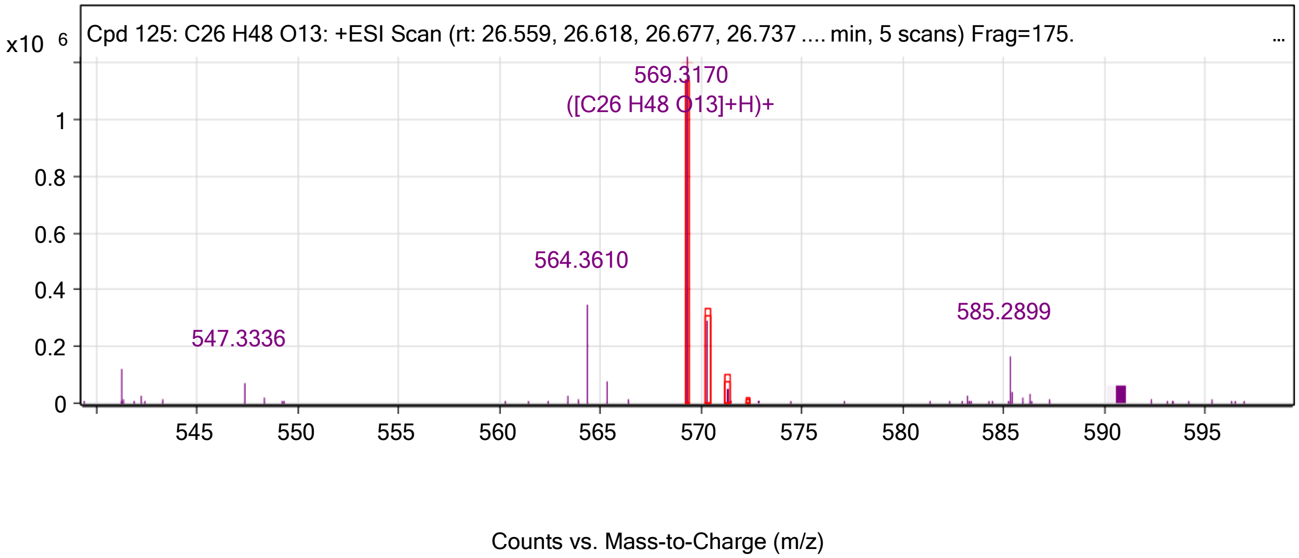

MS Spectrum Peak List

m/z	Calc *m/z*	Diff(ppm)	z	Abund	Formula	Ion
481.2637			1	643439.13		
525.2906			1	1002167.81		
569.317	569.3168	-0.49	1	1220355.13	C26 H48 O13	(M+H)+
570.3196	570.3202	1.02	1	287053.97	C26 H48 O13	(M+H)+
571.3216	571.3226	1.81	1	49491.14	C26 H48 O13	(M+H)+
572.3243	572.3253	1.64	1	6323.81	C26 H48 O13	(M+H)+
608.3873			1	367549.75		
613.3434			1	1062644.25		
657.3695			1	751714.63		

MSMS Spectrum

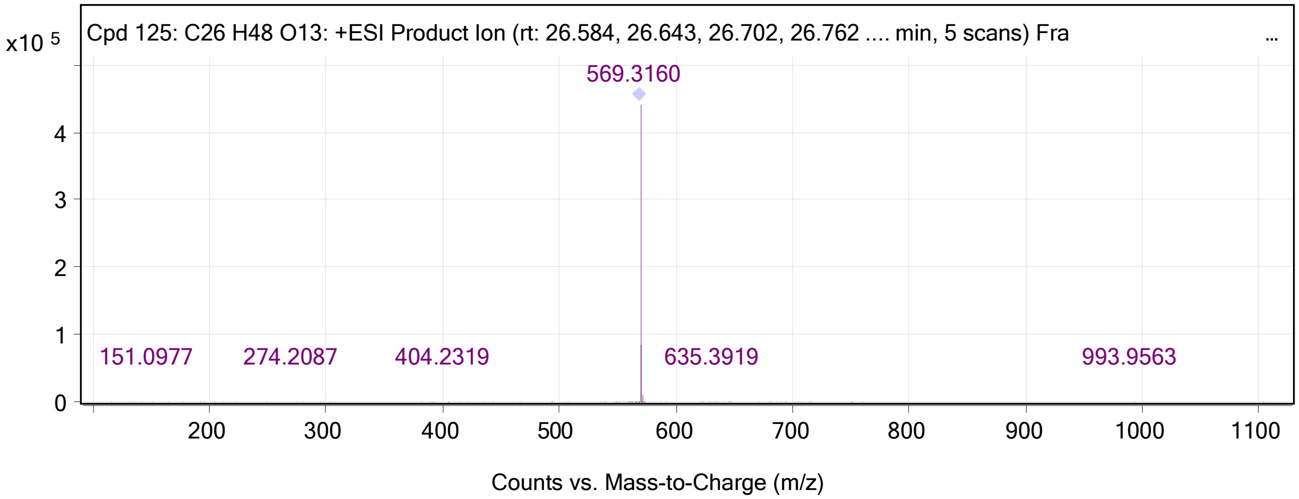

MS/MS Spectrum Peak List

m/z	*Calc m/z*	Diff(ppm)	z	Abund
133.0873	133.0859	-10.34		10.8
136.0925	136.0883	-31.1		12.56
165.0929	165.091	-11.25		14.85
177.1097	177.1121	13.76		12.43
274.2087	274.2139	18.93		15.66
295.1955	295.1904	-17.41		10.94
389.252	389.2534	3.52		9.22
538.3042	269.1489	-500005.89	2	10.09
569.316	569.3168	1.3	1	441824.41
570.3187			1	85973.86

Compound Label	*m/z*	RT	Algorithm	Mass
Cpd 126: C24 H44 O12	525.2906	26.719	Auto MS/MS	524.2832

Compound Chromatograms

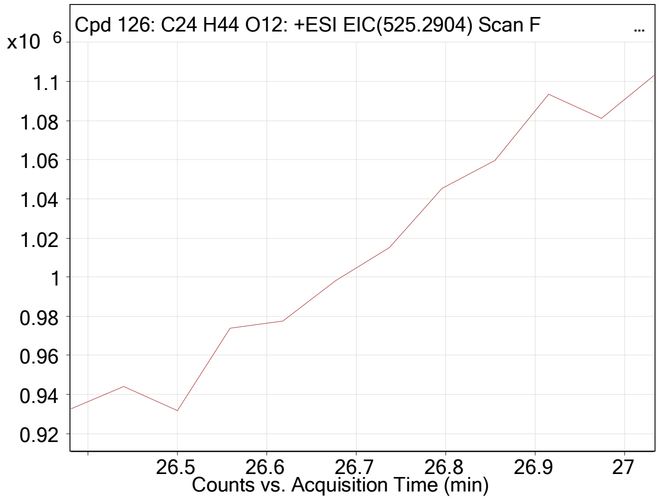

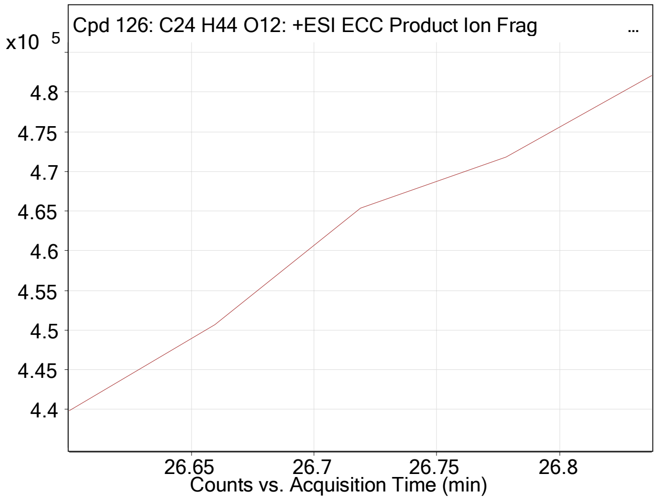

MS Spectrum

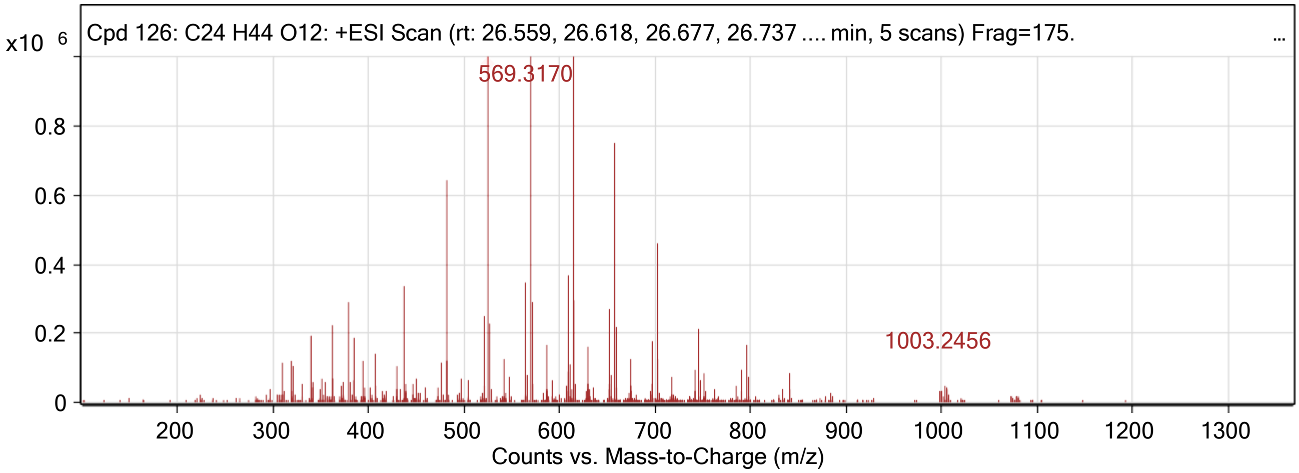

MS Zoomed Spectrum

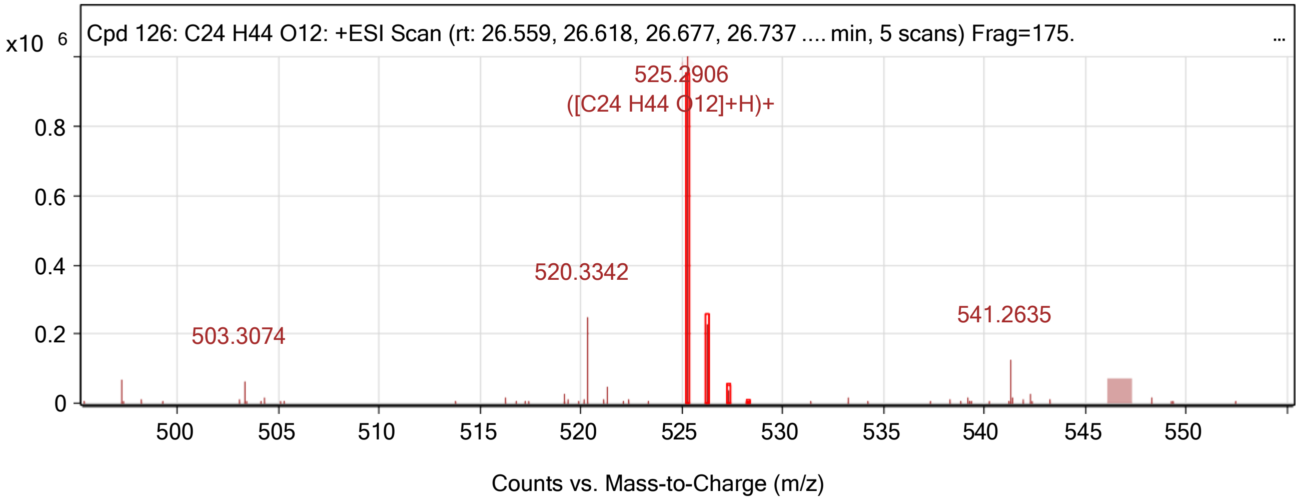

MS Spectrum Peak List

m/z	Calc *m/z*	Diff(ppm)	z	Abund	Formula	Ion
481.2637			1	643439.13		
525.2906	525.2906	-0.11	1	1002167.81	C24 H44 O12	(M+H)+
526.2933	526.294	1.32	1	226014.92	C24 H44 O12	(M+H)+
527.2956	527.2963	1.45	1	37874.53	C24 H44 O12	(M+H)+
528.298	528.299	1.84	1	5052.44	C24 H44 O12	(M+H)+
569.317			1	1220355.13		
608.3873			1	367549.75		
613.3434			1	1062644.25		
657.3695			1	751714.63		
701.3955			1	458282.09		

MSMS Spectrum

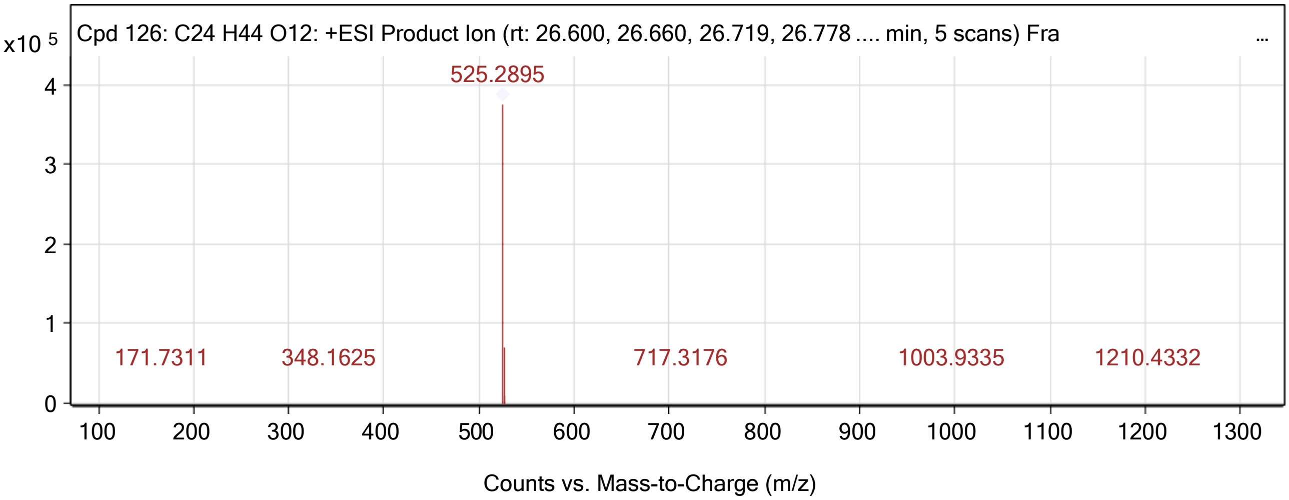

MS/MS Spectrum Peak List

m/z	*Calc m/z*	Diff(ppm)	z	Abund
111.0399	111.0441	37.38		10.95
253.1396	253.1434	15.29		13
263.1946	263.2006	22.8		10.27
277.2486	277.2526	14.44		7.93
296.185	296.183	-6.82		8.8
309.2764	309.2788	7.81		12.14
494.2705	247.1358	-499998.84	2	24.07
495.2715			2	27.03
522.2675	522.2671	-0.8	1	60.46
525.2895	525.2906	2.06	1	375469.09

Compound Label	*m/z*	RT	Algorithm	Mass
Cpd 127: C24 H46 N3 O12	569.3166	26.999	Auto MS/MS	568.3091

Compound Chromatograms

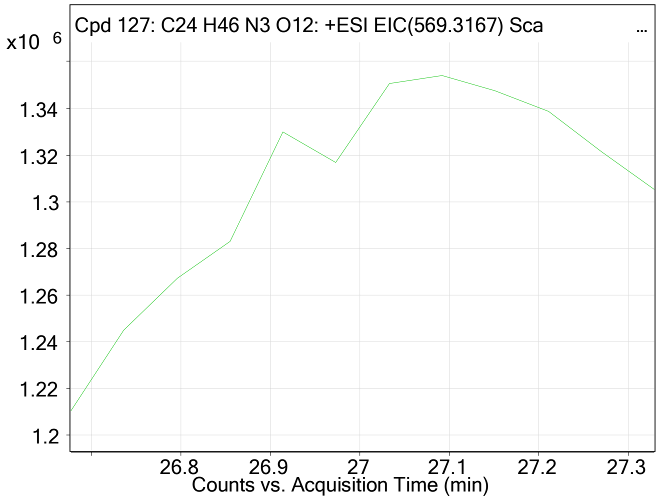

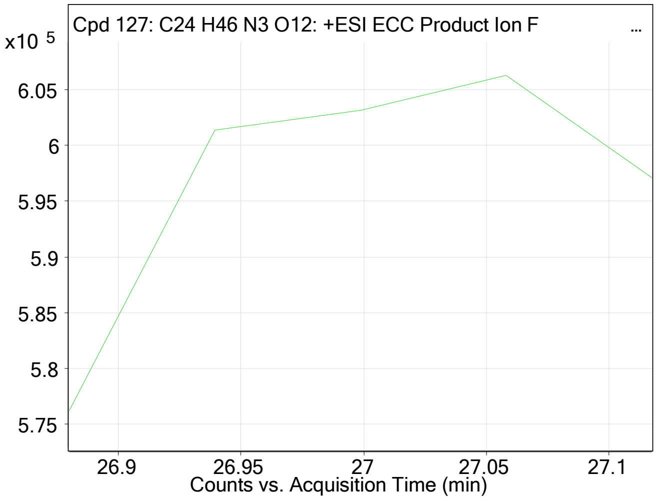

MS Spectrum

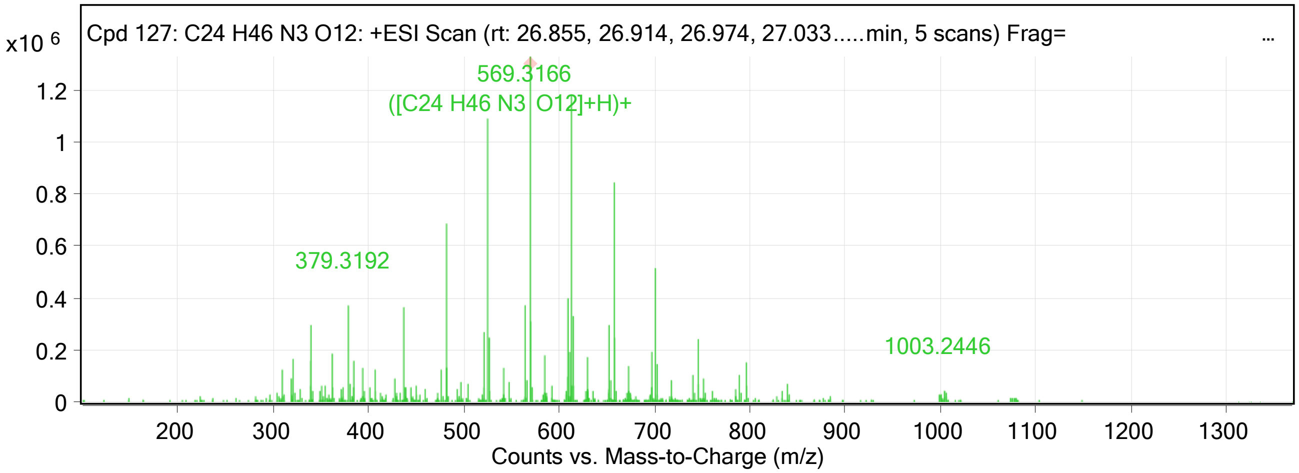

MS Zoomed Spectrum

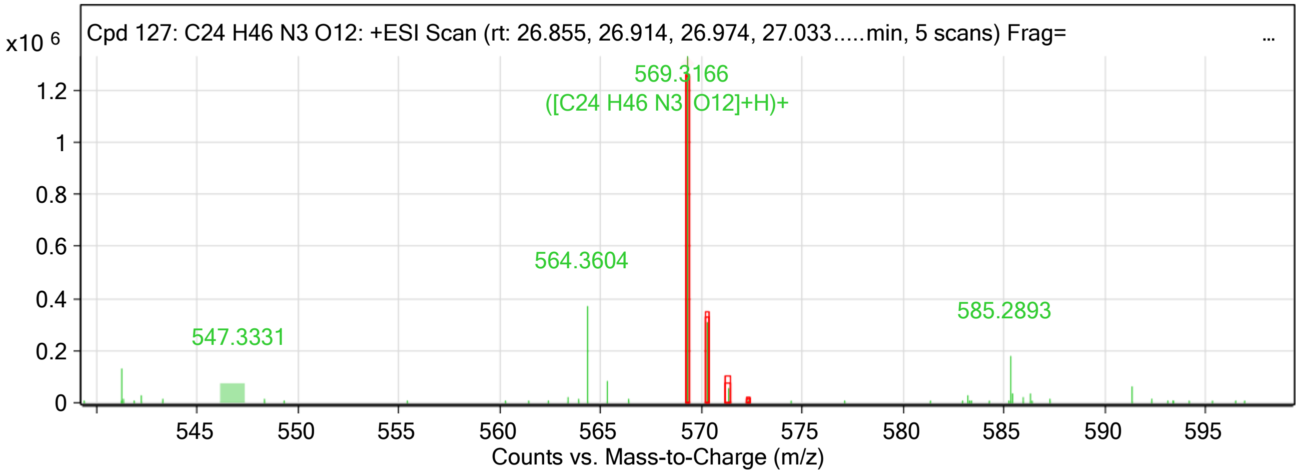

MS Spectrum Peak List

m/z	Calc *m/z*	Diff(ppm)	z	Abund	Formula	Ion
481.2632			1	687156.5		
525.2901			1	1090827.25		
569.3166	569.3154	-2.03	1	1326877.38	C24 H46 N3 O12	(M+H)+
570.3191	570.3186	-0.89	1	310344.09	C24 H46 N3 O12	(M+H)+
571.3211	571.3209	-0.19	1	54245.86	C24 H46 N3 O12	(M+H)+
572.3237	572.3235	-0.31	1	7299.28	C24 H46 N3 O12	(M+H)+
608.3868			1	400318.59		
613.3428			1	1176897.13		
657.3689			1	839534		
701.3948			1	514791		

MSMS Spectrum

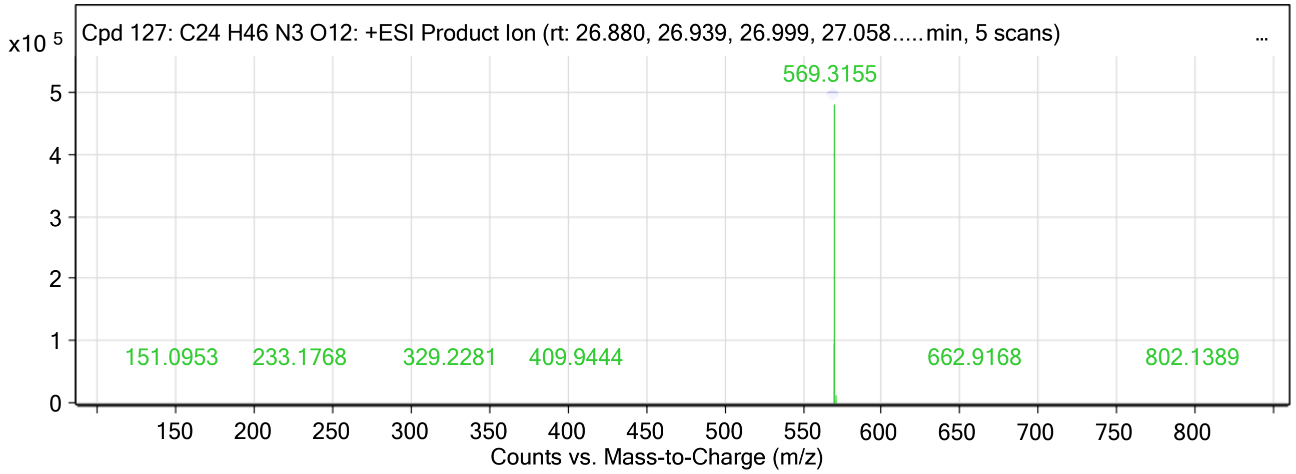

MS/MS Spectrum Peak List

m/z	*Calc m/z*	Diff(ppm)	z	Abund
133.0871	133.0859	-8.84		29.85
144.0756	144.0768	8.15		13.56
151.0953	151.0951	-0.84		46.77
195.1218	195.1214	-2.47		14.55
233.1768	233.1774	2.46		15.2
265.1635	265.1632	-1.13		10.64
309.277	309.2775	1.35		10.95
329.2281	329.2309	8.49		13.2
466.2988	466.3011	4.75		10.73
569.3155	569.3154	-0.13	1	480446.06

Compound Label	*m/z*	RT	Algorithm	Mass
Cpd 128: C22 H42 N3 O11	525.2901	27.015	Auto MS/MS	524.2827

Compound Chromatograms

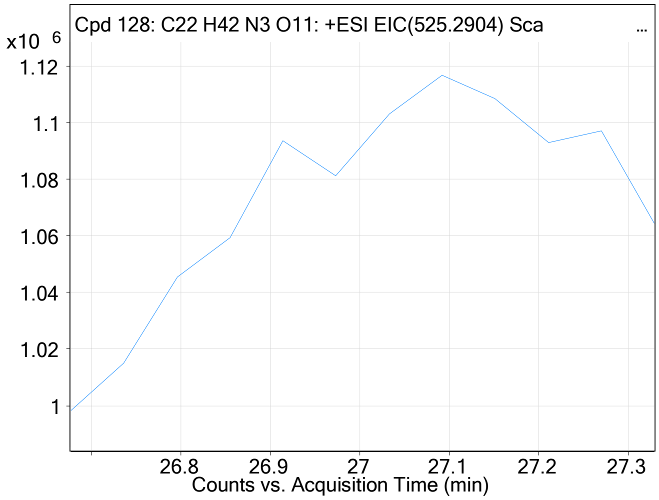

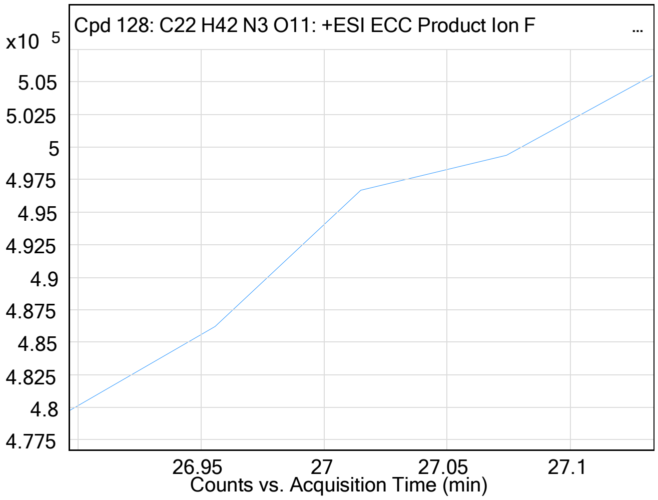

MS Spectrum

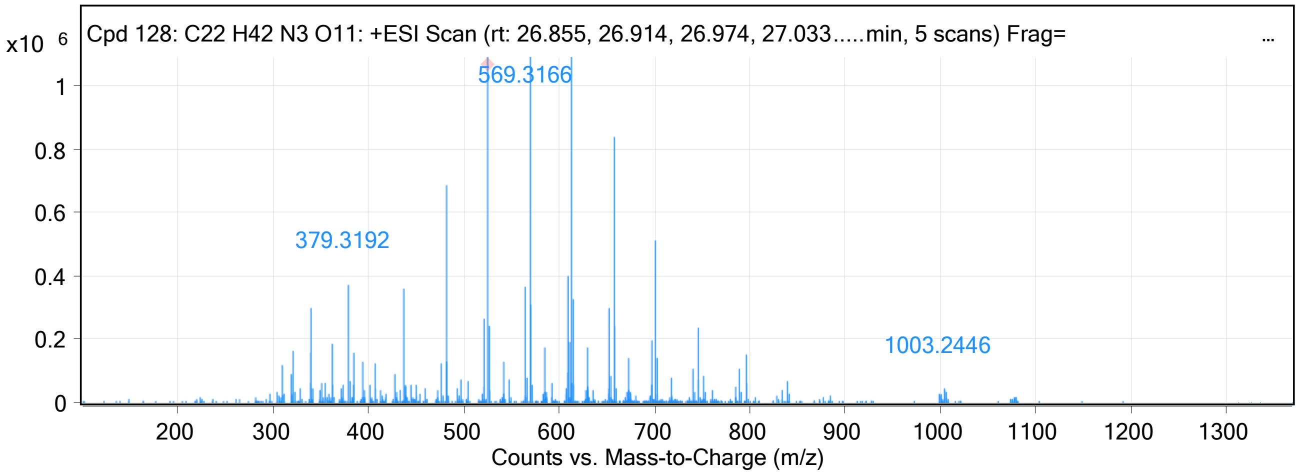

MS Zoomed Spectrum

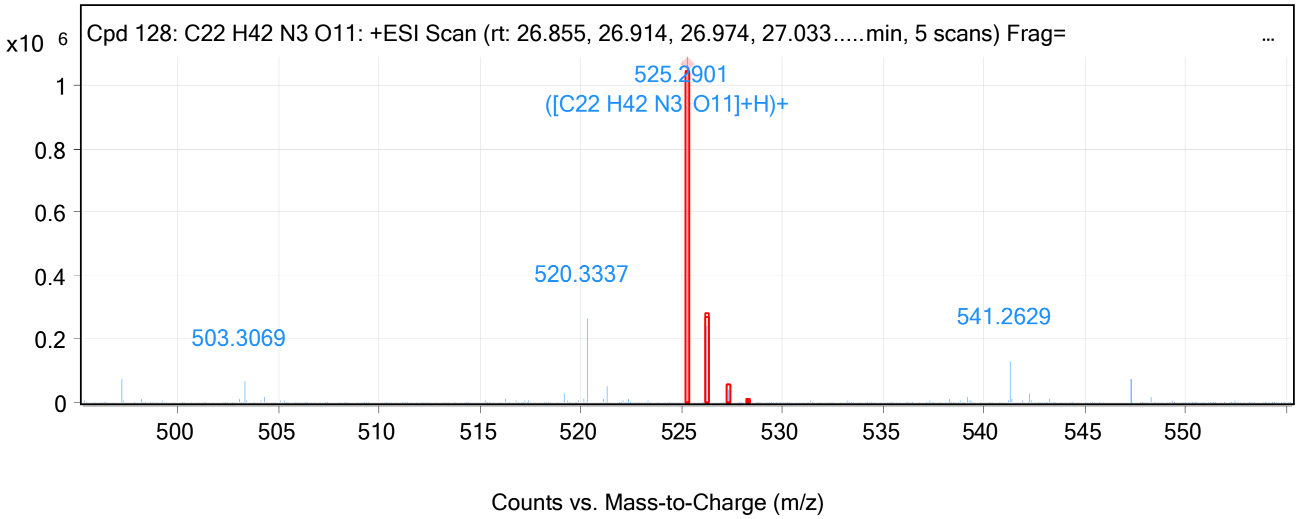

MS Spectrum Peak List

m/z	Calc *m/z*	Diff(ppm)	z	Abund	Formula	Ion
481.2632			1	687156.5		
525.2901	525.2892	-1.68	1	1090827.25	C22 H42 N3 O11	(M+H)+
526.2928	526.2924	-0.8	1	244549.83	C22 H42 N3 O11	(M+H)+
527.2951	527.2947	-0.76	1	40639.93	C22 H42 N3 O11	(M+H)+
528.2978	528.2972	-1.04	1	5166.33	C22 H42 N3 O11	(M+H)+
569.3166			1	1326877.38		
608.3868			1	400318.59		
613.3428			1	1176897.13		
657.3689			1	839534		

MSMS Spectrum

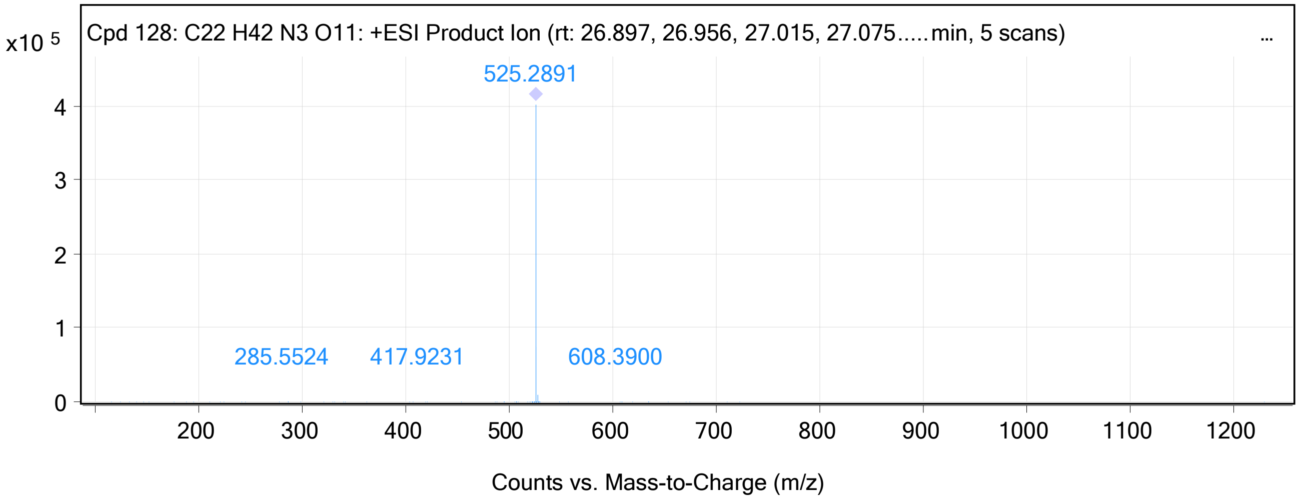

MS/MS Spectrum Peak List

m/z	*Calc m/z*	Diff(ppm)	z	Abund
124.0805	124.0757	-38.94		16.69
151.0973	151.0992	12.04		15.28
188.1035	188.103	-2.9		13.51
210.115	210.1125	-11.93		12.18
221.1368	221.137	0.87		10.74
285.2069	285.206	-3.01		11.87
328.2341	328.2357	4.89		10.43
453.2563	453.2568	1.24		10.08
494.2684	494.2708	4.95	1	26.72
525.2891	525.2892	0.31	1	402156.06

Compound Label	*m/z*	RT	Algorithm	Mass
Cpd 129: C26 H48 O13	569.3168	27.295	Auto MS/MS	568.3093

Compound Chromatograms

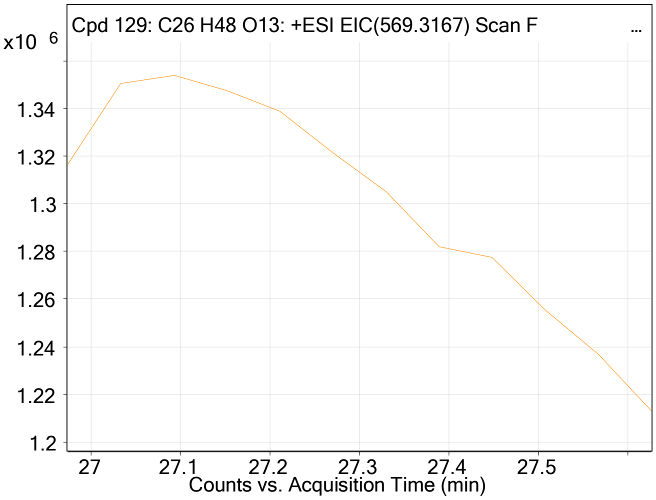

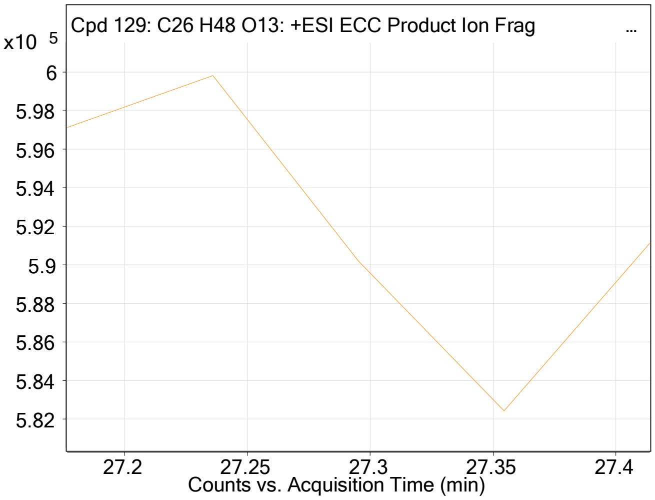

MS Spectrum

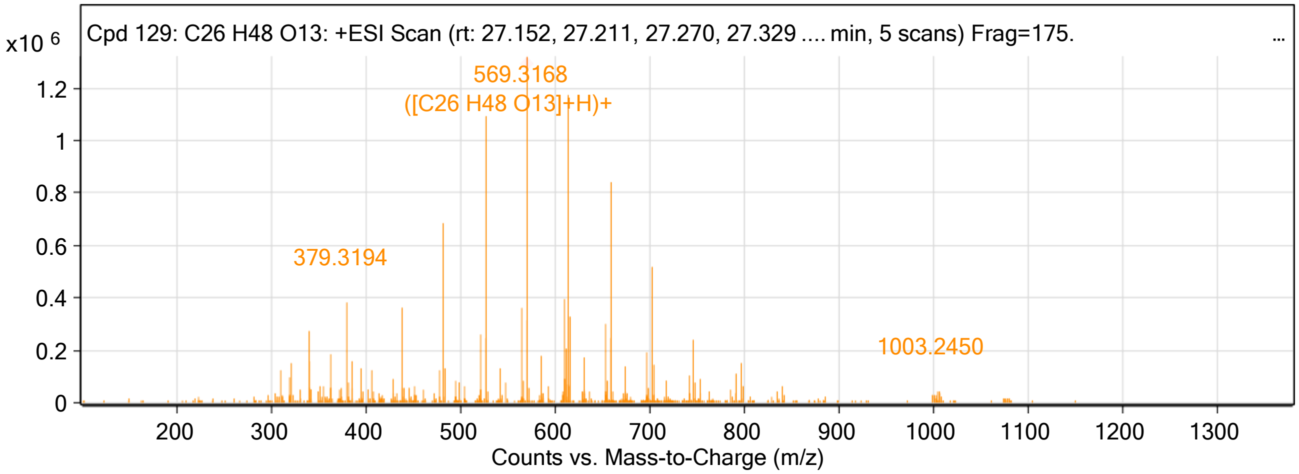

MS Zoomed Spectrum

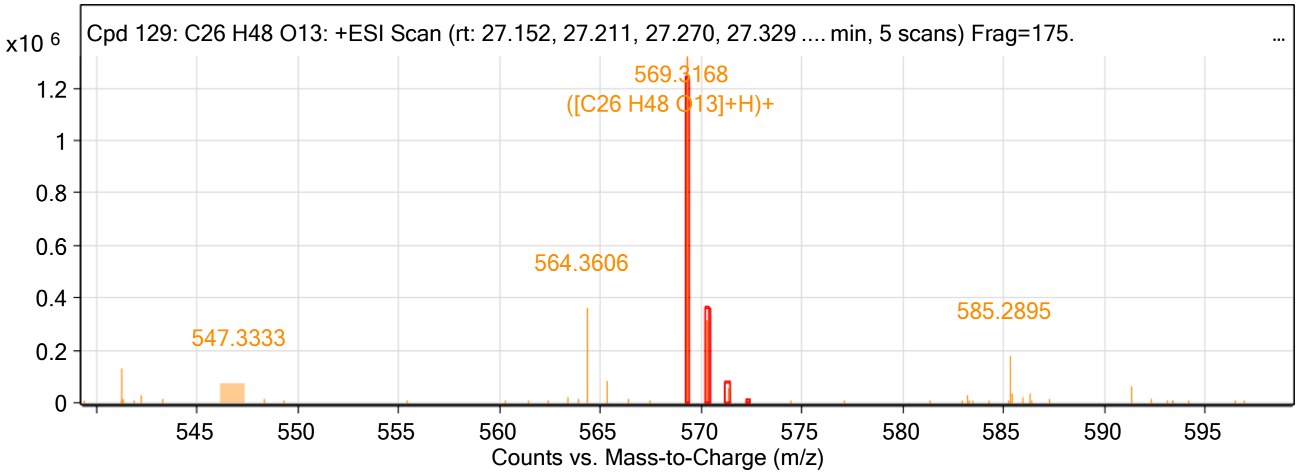

MS Spectrum Peak List

m/z	Calc *m/z*	Diff(ppm)	z	Abund	Formula	Ion
481.2636			1	678218.81		
525.2903			1	1085649.75		
569.3168	569.3168	-0.08	1	1319097.5	C26 H48 O13	(M+H)+
570.3193	570.3202	1.5	1	313481.5	C26 H48 O13	(M+H)+
571.3213	571.3226	2.2	1	54317.59	C26 H48 O13	(M+H)+
572.3238	572.3253	2.64	1	6884.66	C26 H48 O13	(M+H)+
608.387			1	395547.38		
613.3431			1	1168113.75		
657.3692			1	835473.88		
701.3951			1	515739.19		

MSMS Spectrum

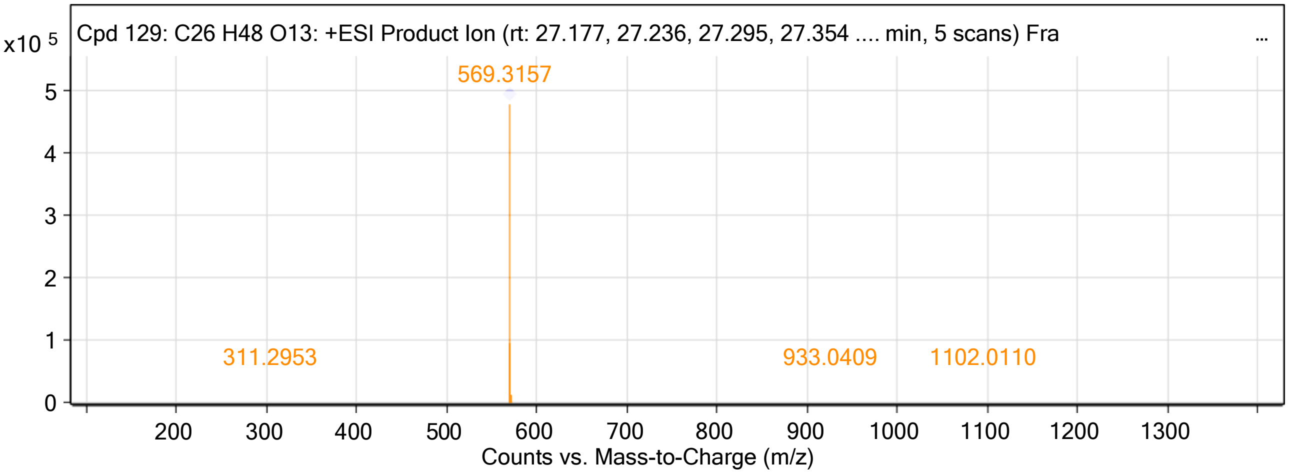

MS/MS Spectrum Peak List

m/z	*Calc m/z*	Diff(ppm)	z	Abund
133.0863	133.0859	-2.47		61.3
157.1222	157.1223	0.57		10.89
177.1123	177.1121	-0.69		11.16
241.1793	241.1798	2.25		14.81
307.2223	307.2268	14.65		11.42
309.2763	309.2788	8.24		18.4
311.2953	311.2945	-2.68		46.37
333.2215	333.2272	17.13		14.56
382.2566	382.2561	-1.34		14.08
569.3157	569.3168	1.83	1	476343.34

Compound Label	*m/z*	RT	Algorithm	Mass
Cpd 130: C22 H42 N3 O11	525.2903	27.312	Auto MS/MS	524.2829

Compound Chromatograms

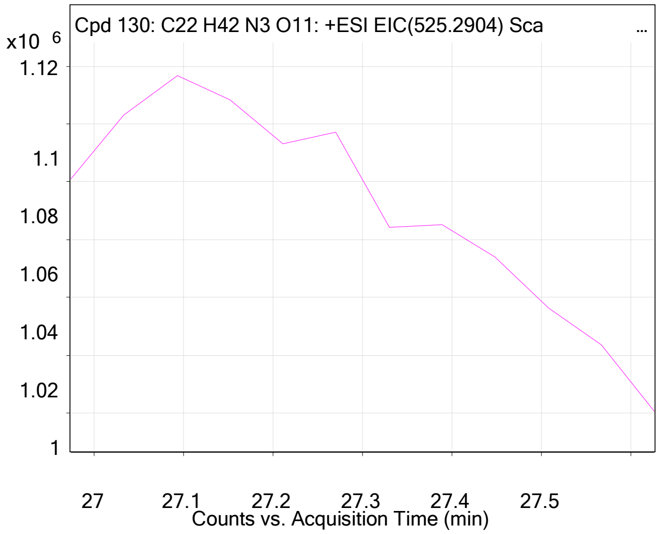

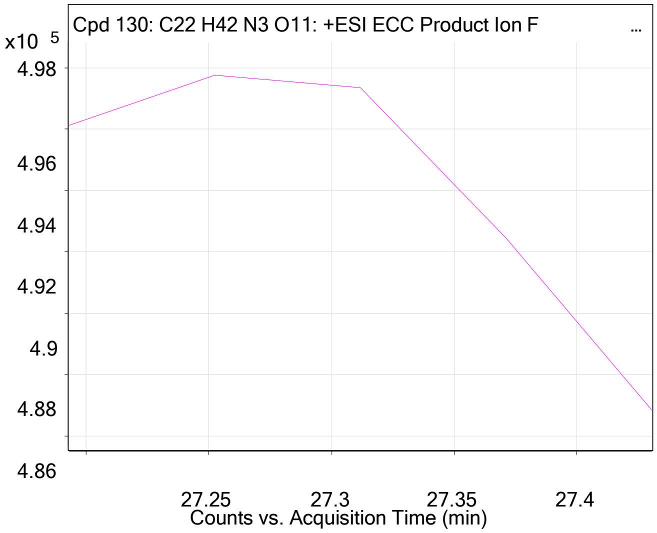

MS Spectrum

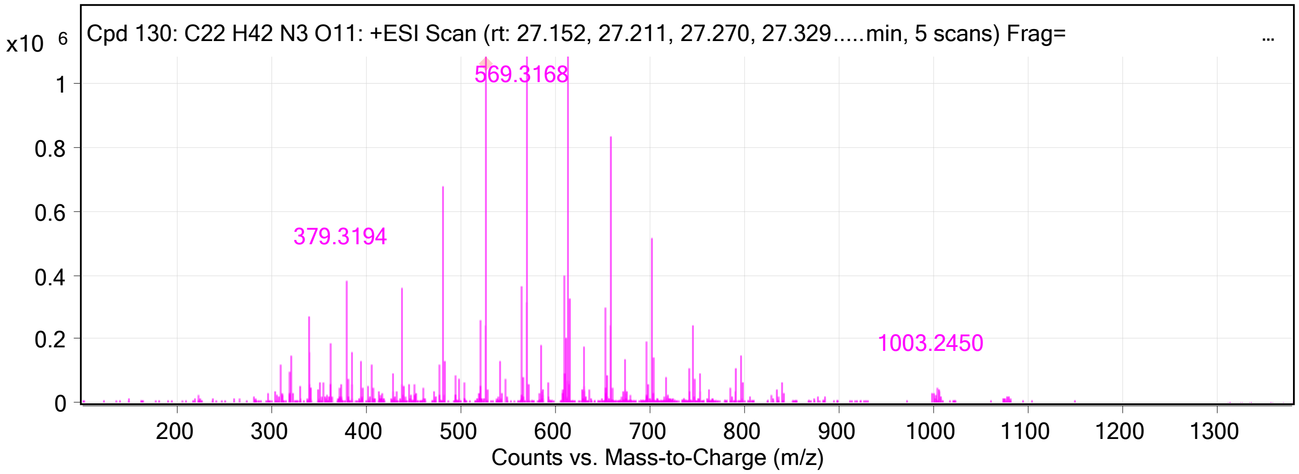

MS Zoomed Spectrum

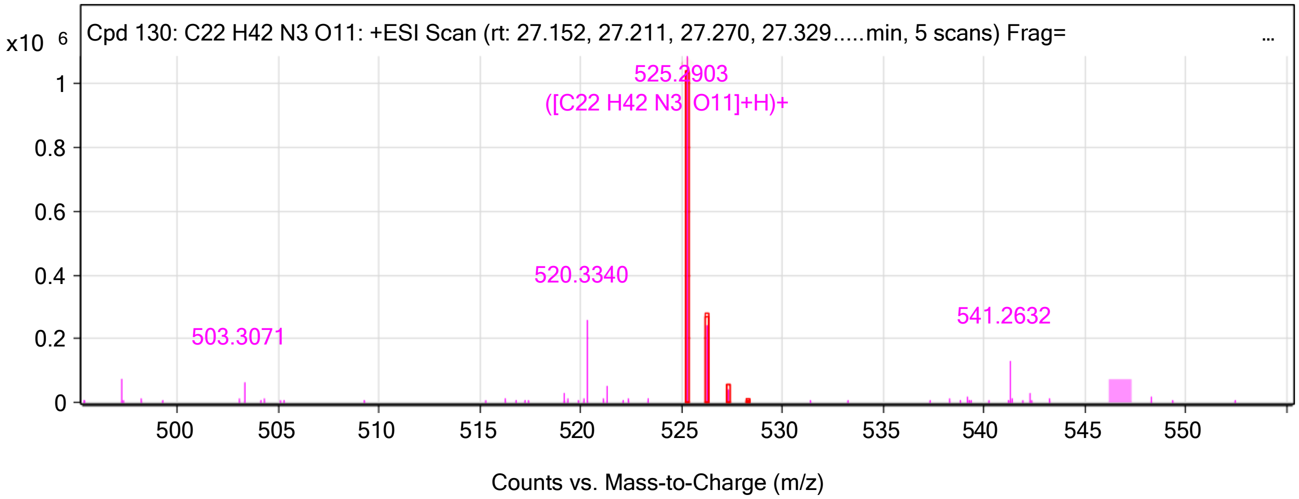

MS Spectrum Peak List

m/z	Calc *m/z*	Diff(ppm)	z	Abund	Formula	Ion
481.2636			1	678218.81		
525.2903	525.2892	-2.14	1	1085649.75	C22 H42 N3 O11	(M+H)+
526.293	526.2924	-1.15	1	243194.59	C22 H42 N3 O11	(M+H)+
527.2952	527.2947	-0.94	1	41871.57	C22 H42 N3 O11	(M+H)+
528.298	528.2972	-1.51	1	5494.75	C22 H42 N3 O11	(M+H)+
569.3168			1	1319097.5		
608.387			1	395547.38		
613.3431			1	1168113.75		
657.3692			1	835473.88		
701.3951			1	515739.19		

MSMS Spectrum

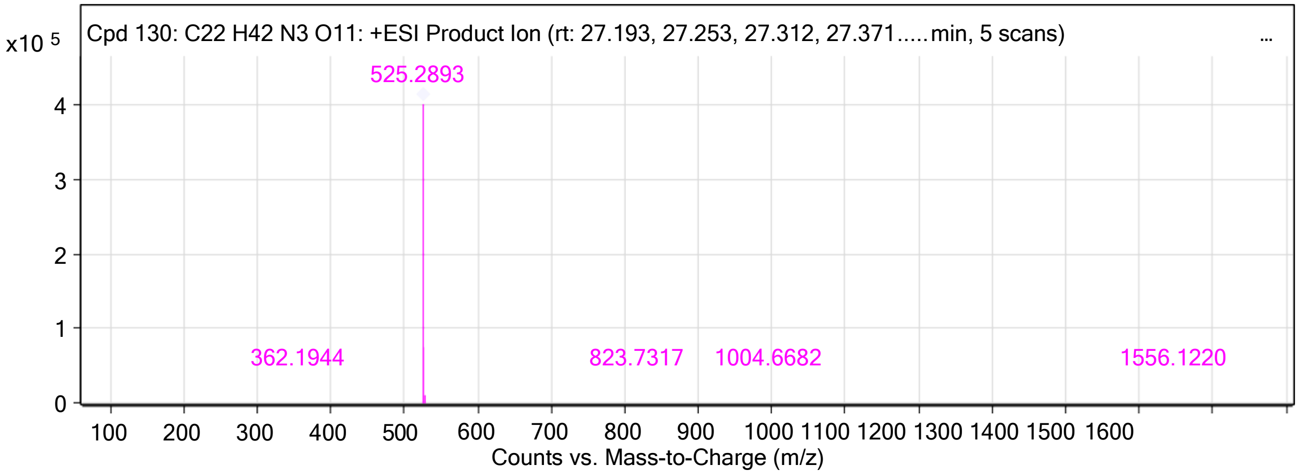

MS/MS Spectrum Peak List

m/z	Calc *m/z*	Diff(ppm)	z	Abund
117.0907	117.091	2.96		10.47
133.0851	133.0846	-4.24		32.97
209.0805	209.0768	-17.74		17.6
309.2824	309.2788	-11.56		11.6
318.2342	318.2387	14.3		10.24
334.245	334.2462	3.56		10.86
362.1944	362.1935	-2.51		19.26
478.2779	478.2759	-4.2		10.03
494.2742	247.1351	-500003.99	2	19.47
525.2893	525.2892	-0.13	1	400943.59

Compound Label	*m/z*	RT	Algorithm	Mass
Cpd 131: C26 H48 O13	569.3168	27.592	Auto MS/MS	568.3094

Compound Chromatograms

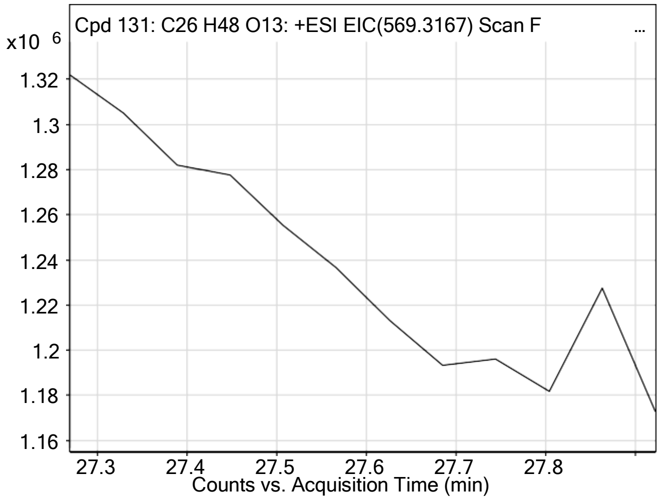

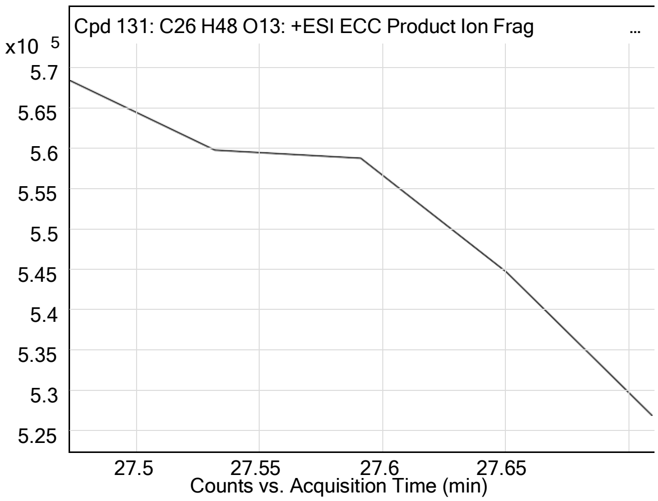

MS Spectrum

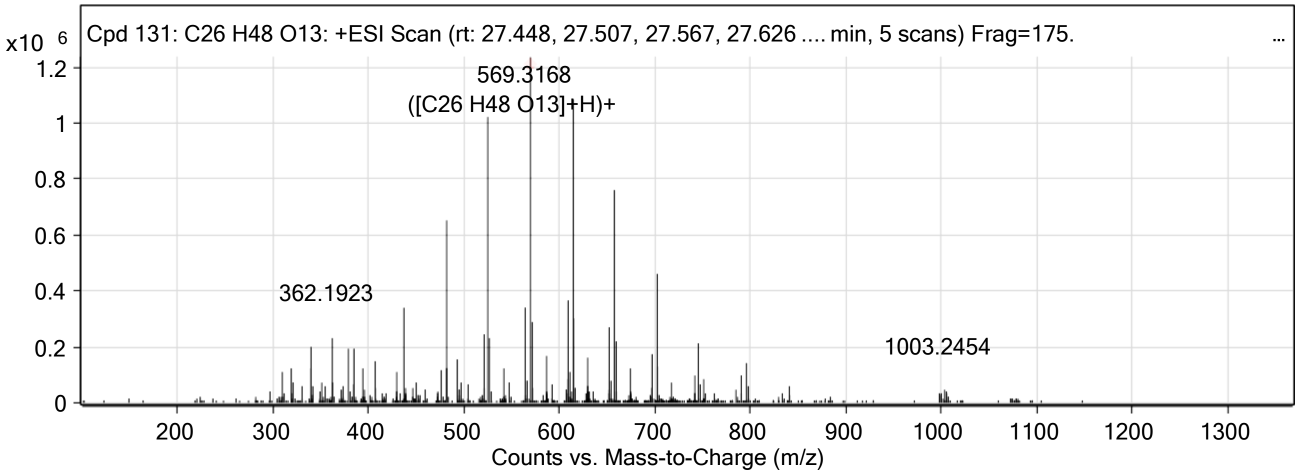

MS Zoomed Spectrum

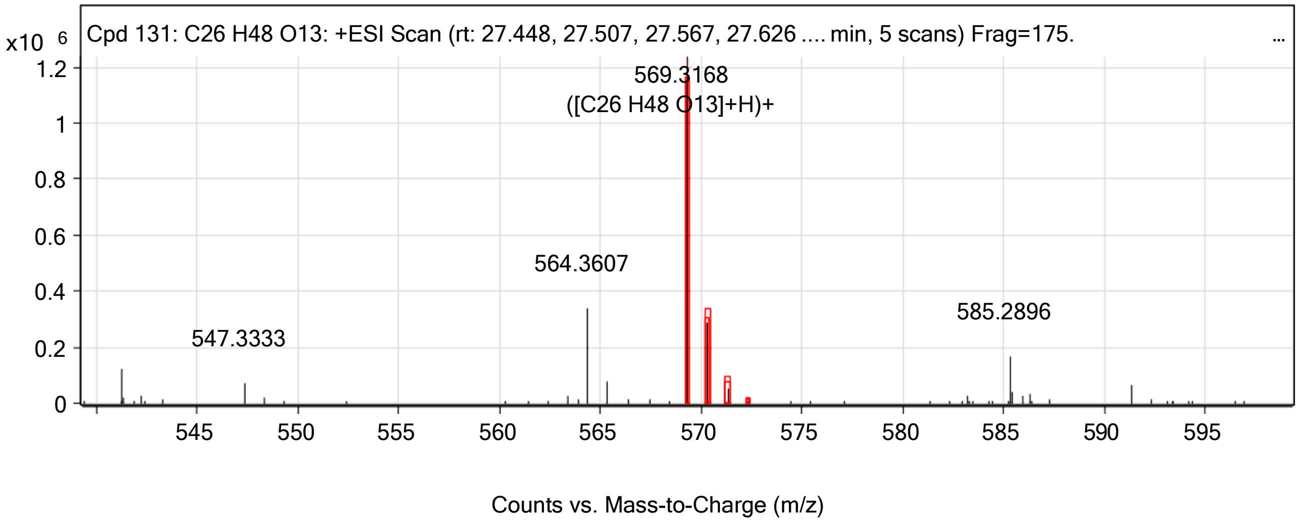

MS Spectrum Peak List

m/z	Calc *m/z*	Diff(ppm)	z	Abund	Formula	Ion
481.2635			1	650201.56		
525.2904			1	1018129.69		
569.3168	569.3168	-0.14	1	1235237.75	C26 H48 O13	(M+H)+
570.3194	570.3202	1.48	1	289762.81	C26 H48 O13	(M+H)+
571.3213	571.3226	2.21	1	50886.73	C26 H48 O13	(M+H)+
572.3241	572.3253	2.14	1	6892.16	C26 H48 O13	(M+H)+
608.3871			1	363693.88		
613.3431			1	1081946.75		
657.3692			1	760803.38		

MSMS Spectrum

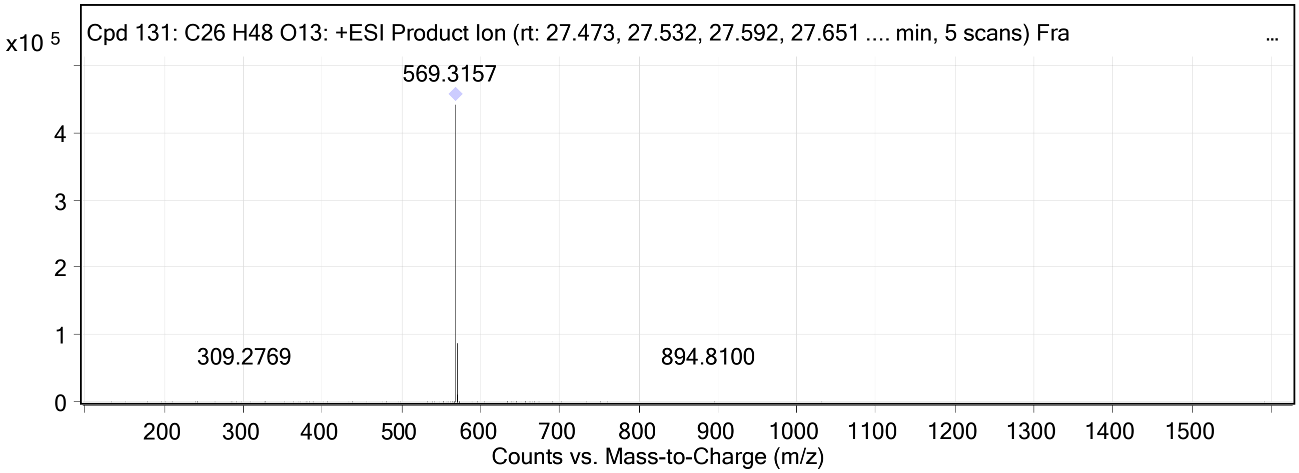

MS/MS Spectrum Peak List

m/z	*Calc m/z*	Diff(ppm)	z	Abund
133.0864	133.0859	-3.48		17.54
283.263	283.2632	0.49		15.63
285.2068	285.206	-2.6		13.42
291.2183	291.2166	-6		12.23
309.2769	309.2788	6.16		22.8
432.2722	432.2718	-1.06		13.04
569.3157	569.3168	1.83	1	442308.31
570.3183			1	88089.32
571.3206			1	10556.08
571.3915			2	984.8

Compound Label	*m/z*	RT	Algorithm	Mass
Cpd 132: C22 H42 N3 O11	525.2904	27.608	Auto MS/MS	524.283

Compound Chromatograms

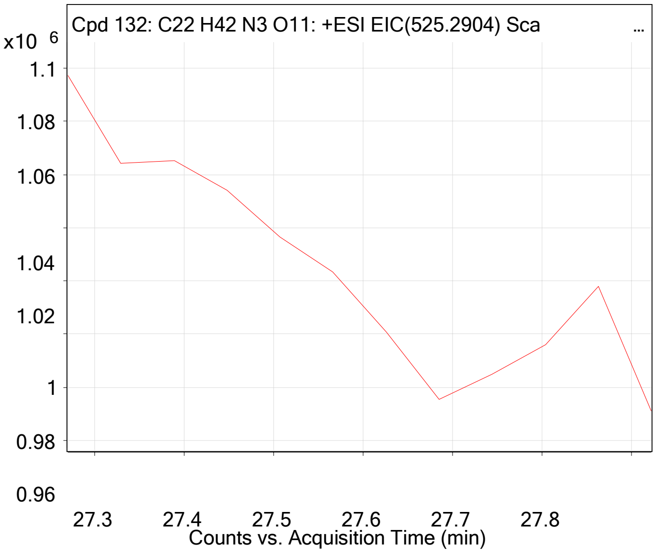

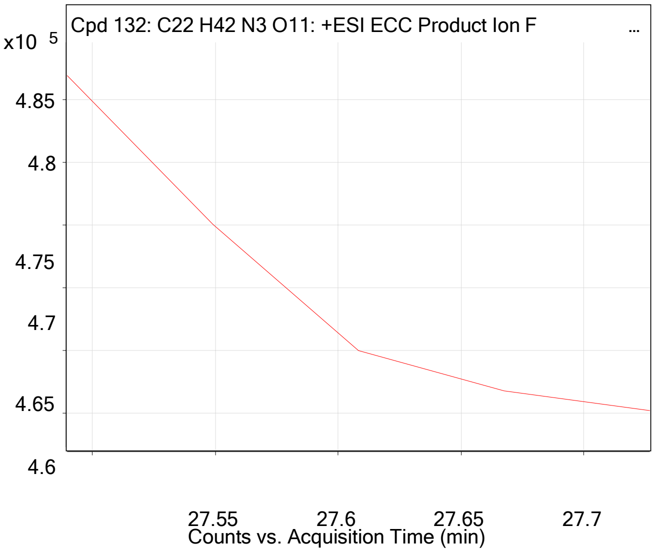

MS Spectrum

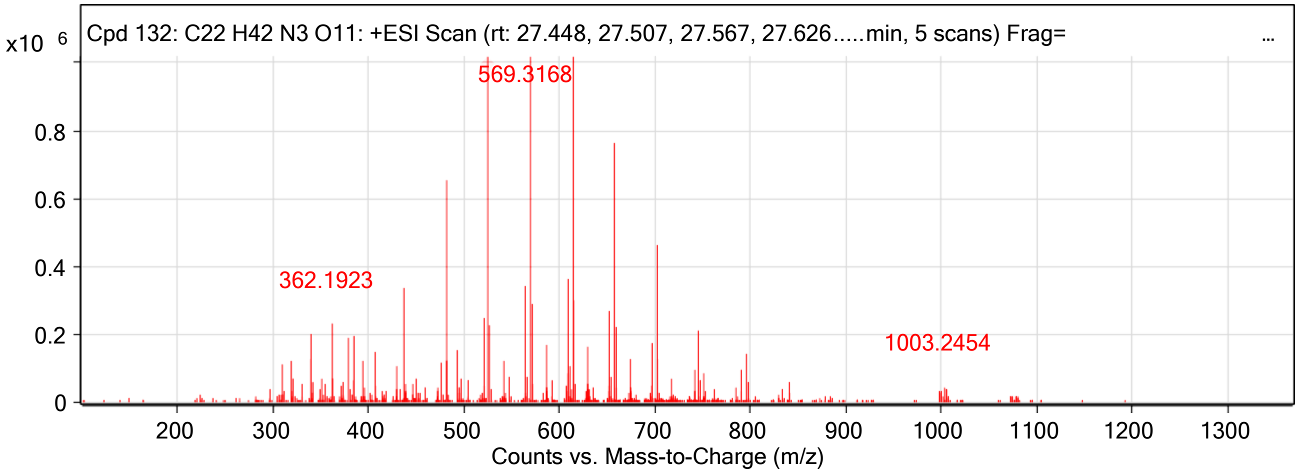

MS Zoomed Spectrum

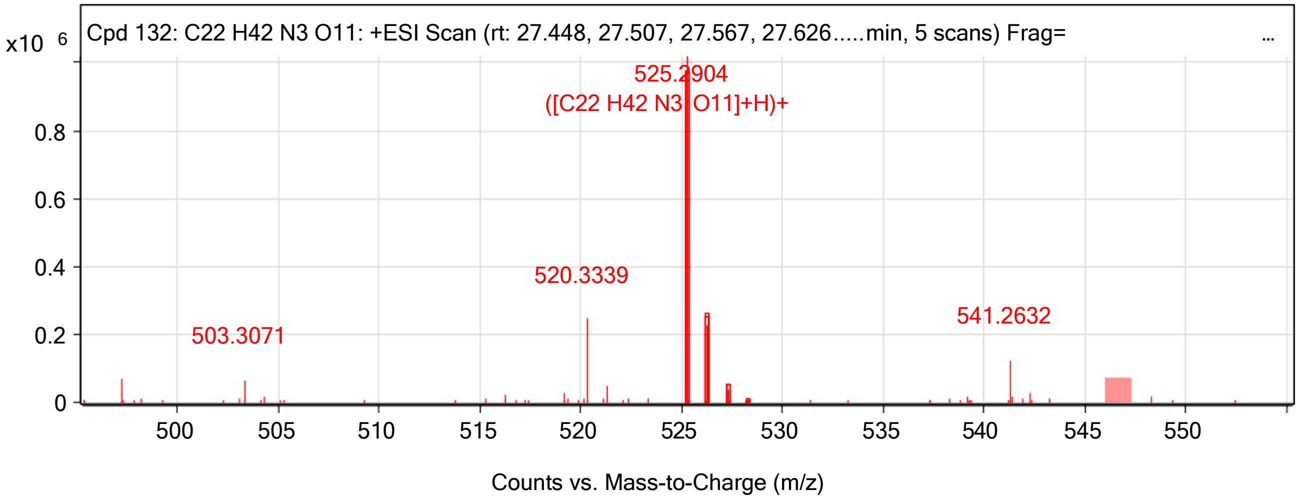

MS Spectrum Peak List

m/z	Calc *m/z*	Diff(ppm)	z	Abund	Formula	Ion
481.2635			1	650201.56		
525.2904	525.2892	-2.22	1	1018129.69	C22 H42 N3 O11	(M+H)+
526.293	526.2924	-1.29	1	227756.58	C22 H42 N3 O11	(M+H)+
527.2954	527.2947	-1.33	1	38335.95	C22 H42 N3 O11	(M+H)+
528.2979	528.2972	-1.26	1	5128.02	C22 H42 N3 O11	(M+H)+
569.3168			1	1235237.75		
608.3871			1	363693.88		
613.3431			1	1081946.75		
657.3692			1	760803.38		
701.3952			1	461156.81		

MSMS Spectrum

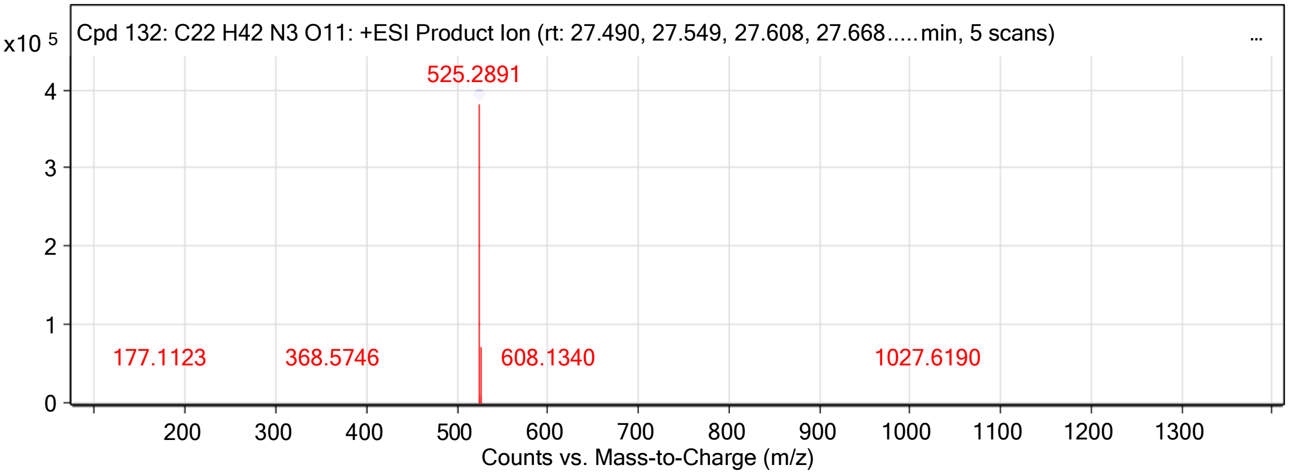

MS/MS Spectrum Peak List

m/z	*Calc m/z*	Diff(ppm)	z	Abund
133.0874	133.0886	8.76		15.82
177.1123	177.1121	-0.94		32.94
232.1294	232.1292	-1.11		24.43
271.1684	271.1652	-11.74		12.96
274.0642	274.0643	0.29		11.84
295.2002	295.1989	-4.42		10.06
495.2688	495.2674	-2.86		25.31
497.2673	497.2705	6.31		13.21
509.2953	509.2943	-1.94		39.68
525.2891	525.2892	0.16	1	381051.03

Compound Label	*m/z*	RT	Algorithm	Mass
Cpd 133: C22 H42 N3 O11	525.2899	27.845	Auto MS/MS	524.2825

Compound Chromatograms

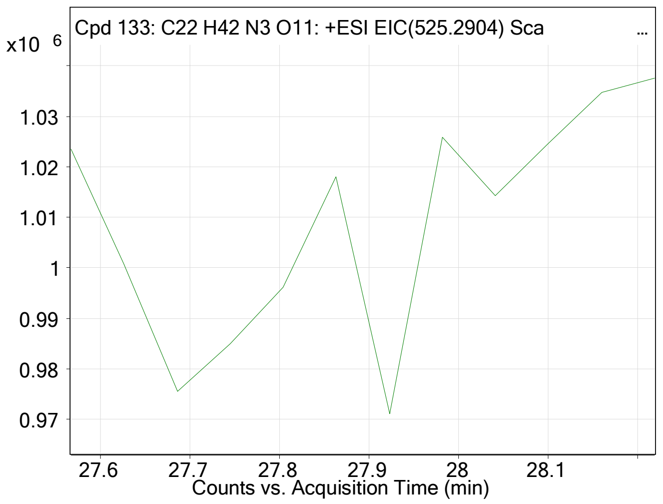

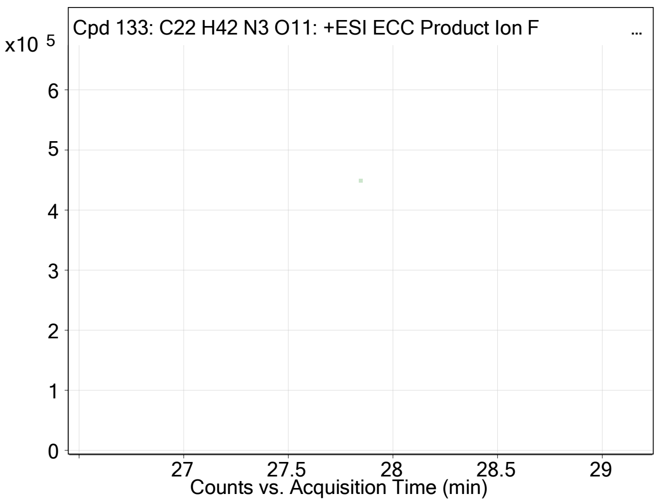

MS Spectrum

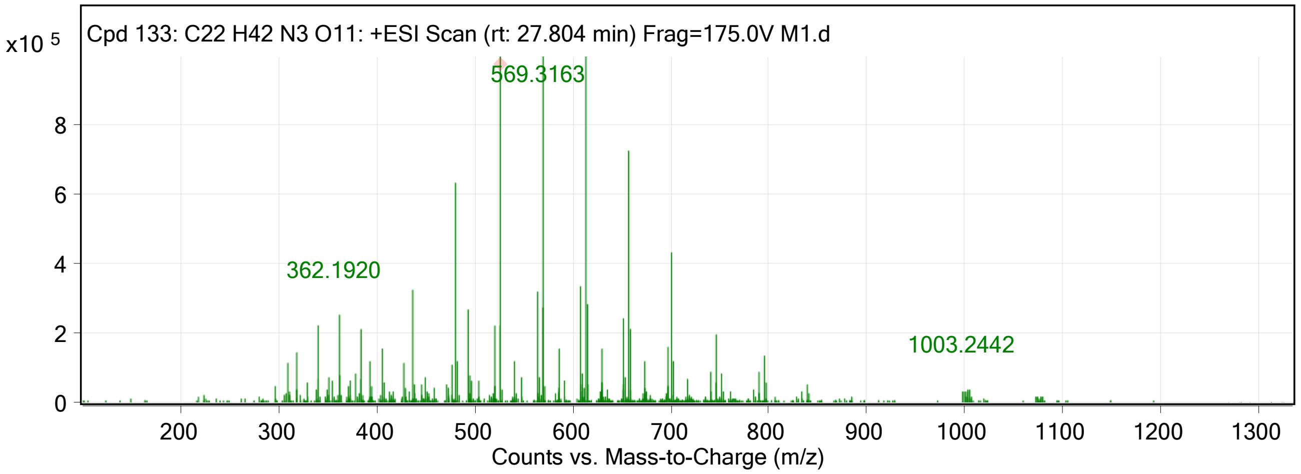

MS Zoomed Spectrum

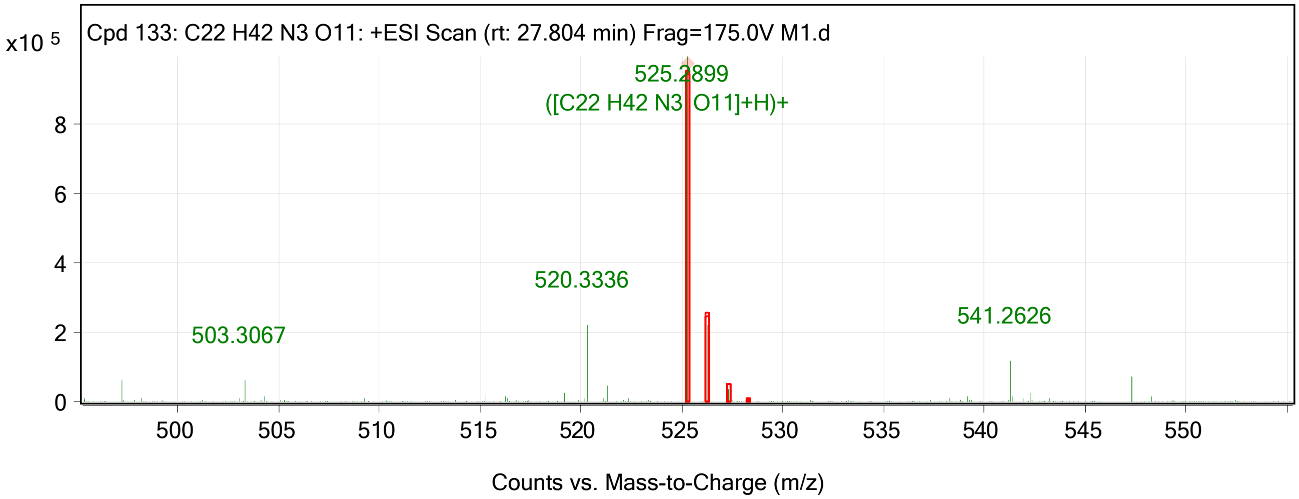

MS Spectrum Peak List

m/z	Calc *m/z*	Diff(ppm)	z	Abund	Formula	Ion
481.2631			1	631035.31		
525.2899	525.2892	-1.32	1	996111.56	C22 H42 N3 O11	(M+H)+
526.2924	526.2924	-0.04	1	220428.97	C22 H42 N3 O11	(M+H)+
527.295	527.2947	-0.68	1	37314.72	C22 H42 N3 O11	(M+H)+
528.2977	528.2972	-0.97	1	4870.87	C22 H42 N3 O11	(M+H)+
569.3163			1	1181843.88		
608.3865			1	334313.59		
613.3425			1	1033361.69		
657.3686			1	723454.56		
701.3946			1	432939.5		

MSMS Spectrum

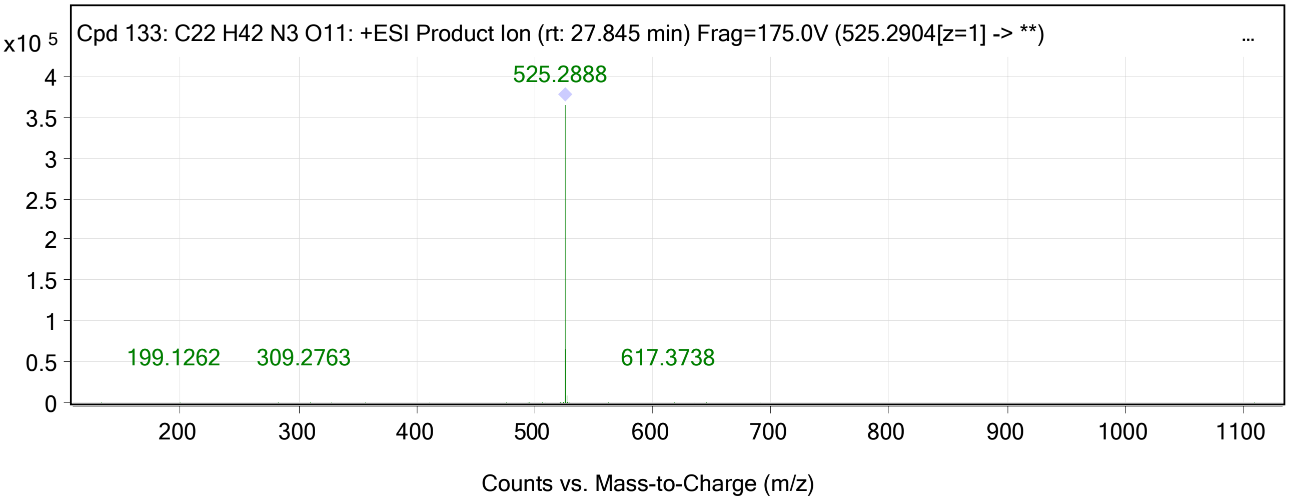

MS/MS Spectrum Peak List

m/z	Calc *m/z*	Diff(ppm)	z	Abund
133.0871	133.0859	-9.22		54
199.1262	199.1315	26.58		46.62
283.1759	283.1778	6.63		54.86
309.2763	309.2775	3.74		69.73
327.1944	327.1914	-9.03		39.45
494.2744	494.2708	-7.19	1	88.88
509.2927	509.2943	3.21		138.46
525.2888	525.2892	0.88	1	365067.47
526.2916			1	66137.36
527.2944			1	8798.19

Compound Label	*m/z*	RT	Algorithm	Mass
Cpd 134: C26 H48 O13	569.3169	27.888	Auto MS/MS	568.3094

Compound Chromatograms

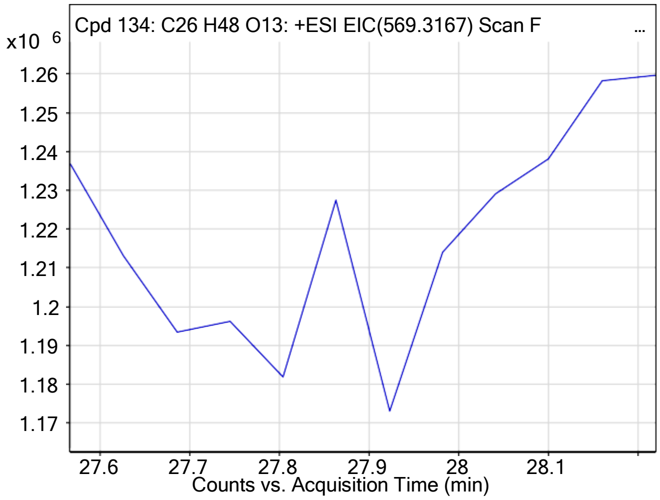

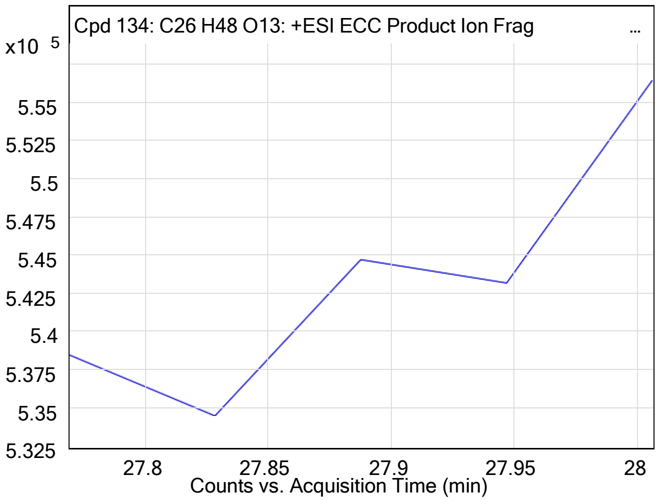

MS Spectrum

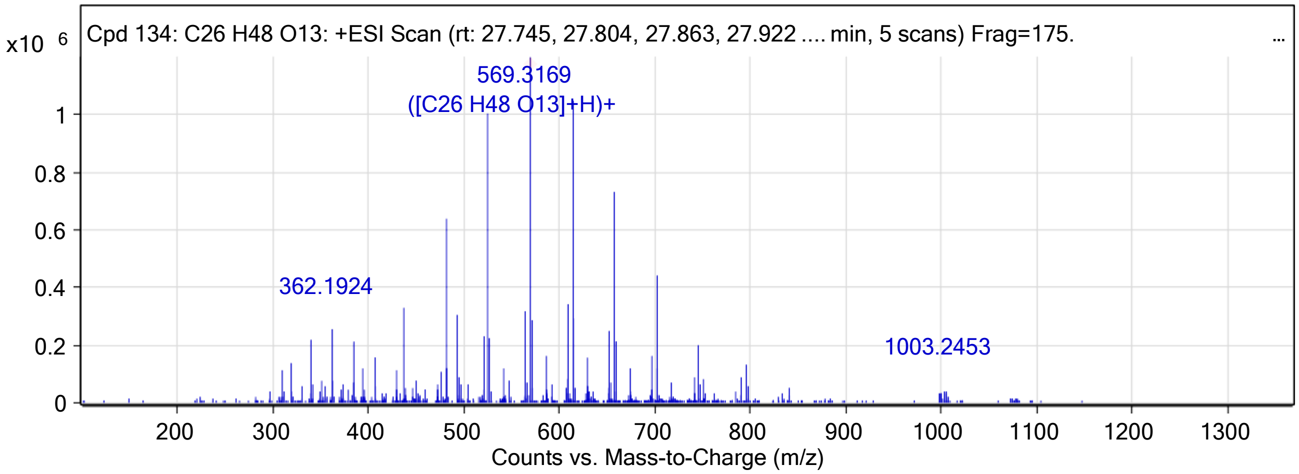

MS Zoomed Spectrum

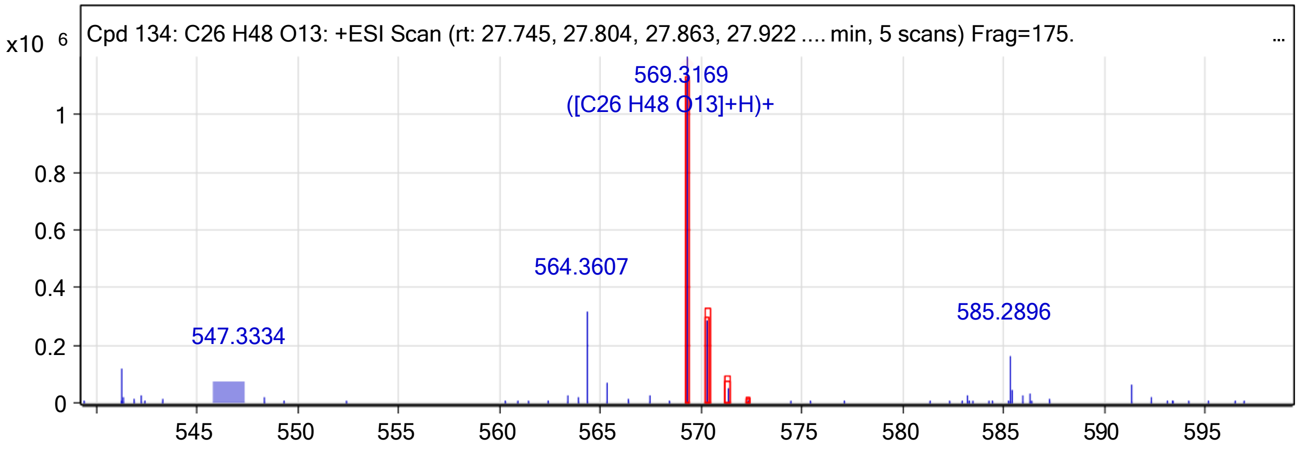

Counts vs. Mass-to-Charge (m/z)

MS Spectrum Peak List

m/z	Calc *m/z*	Diff(ppm)	z	Abund	Formula	Ion
481.2635			1	636826.44		
525.2904			1	999235.63		
569.3169	569.3168	-0.2	1	1198499.25	C26 H48 O13	(M+H)+
570.3194	570.3202	1.44	1	281516.69	C26 H48 O13	(M+H)+
571.3213	571.3226	2.33	1	48971.41	C26 H48 O13	(M+H)+
572.324	572.3253	2.32	1	6754.69	C26 H48 O13	(M+H)+
608.3871			1	341391.47		
613.3431			1	1047000.38		
657.3692			1	730526.63		

MSMS Spectrum

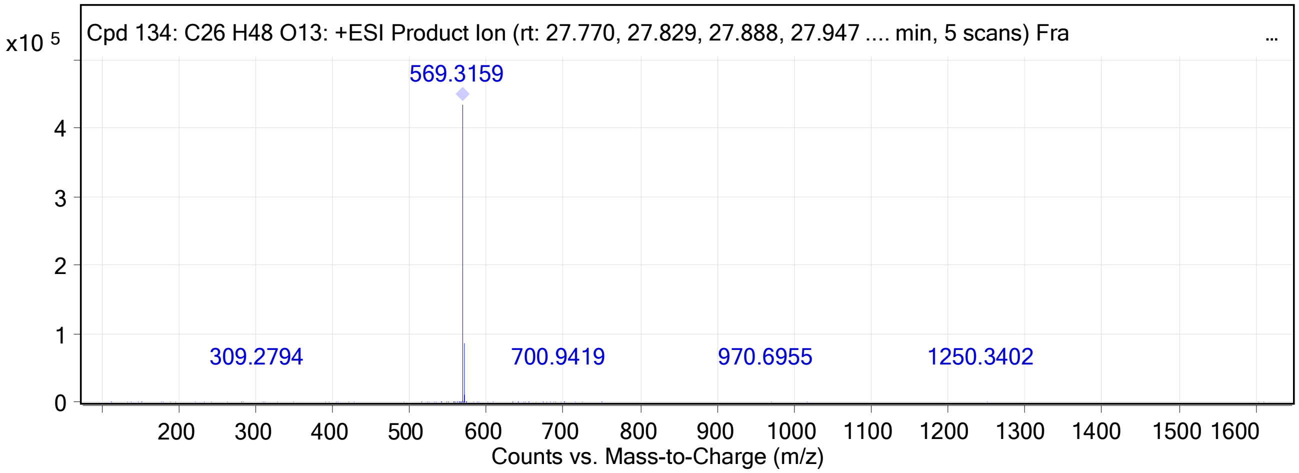

MS/MS Spectrum Peak List

m/z	*Calc m/z*	Diff(ppm)	z	Abund
113.0987	113.0961	-23.19		14.41
133.086	133.0859	-0.81		60.75
177.1112	177.1121	5.19		15.96
188.1026	188.1043	9.13		14.87
263.1908	263.1853	-20.95		25.96
283.2691	283.2632	-20.85		16.02
309.2794	309.2788	-2.04		40.3
311.2952	311.2945	-2.45		29.36
523.2823	523.2749	-14.11		13.45
569.3159	569.3168	1.58	1	434960.31

Compound Label	*m/z*	RT	Algorithm	Mass
Cpd 135: C28 H52 O14	613.3433	27.905	Auto MS/MS	612.3358

Compound Chromatograms

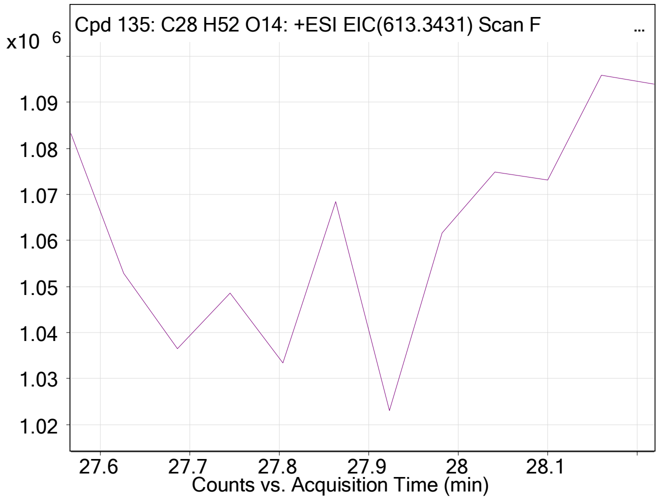

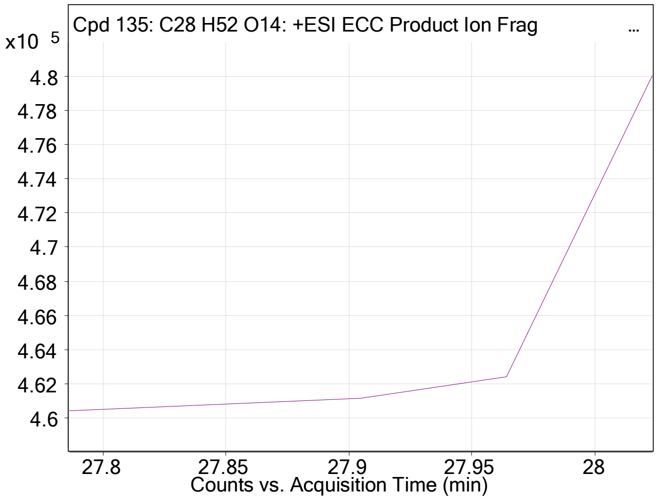

MS Spectrum

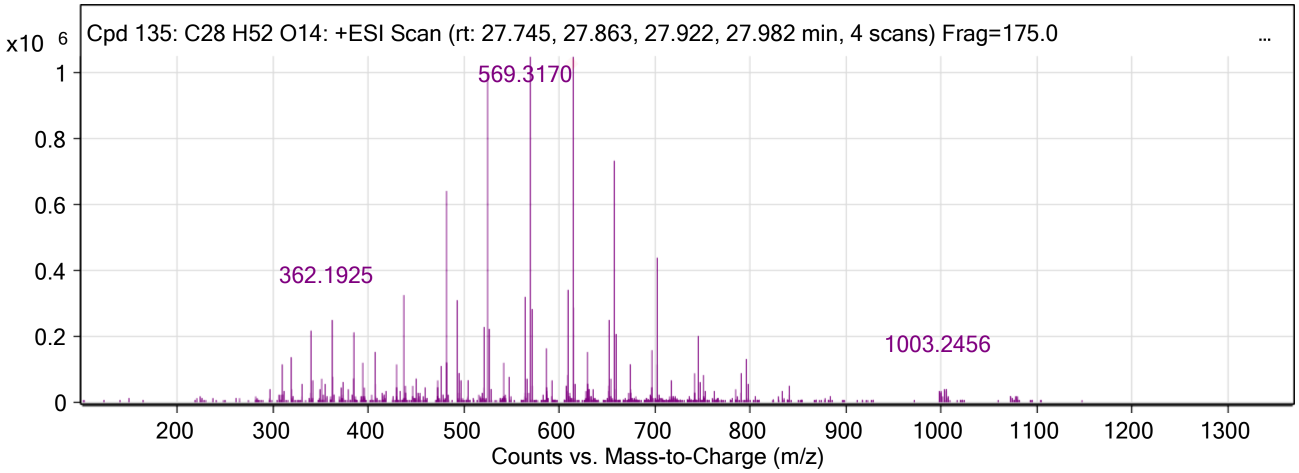

MS Zoomed Spectrum

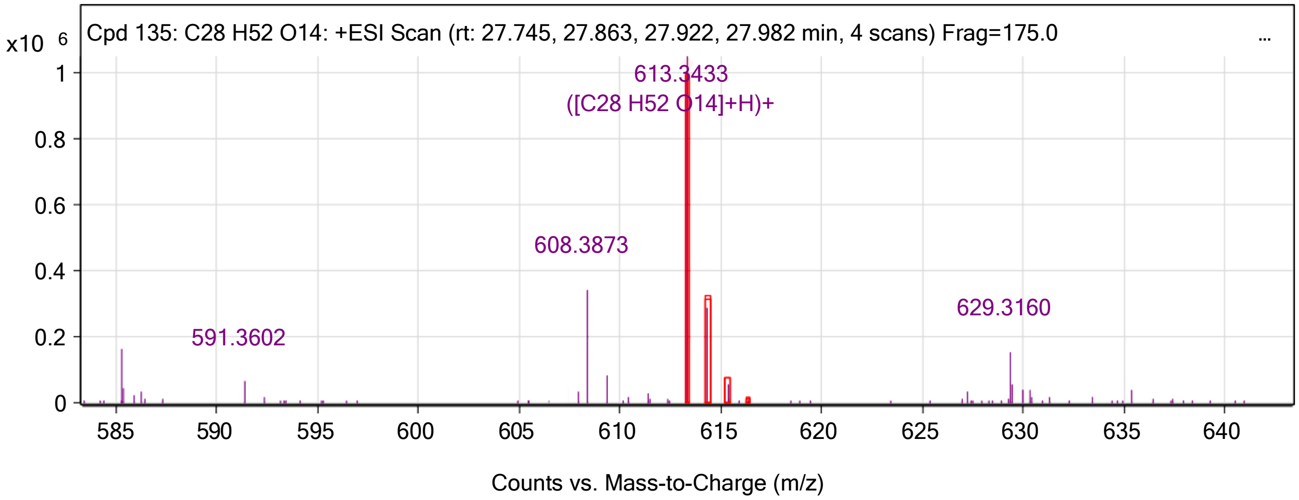

MS Spectrum Peak List

m/z	Calc *m/z*	Diff(ppm)	z	Abund	Formula	Ion
481.2636			1	638274.25		
525.2905			1	1000016.56		
569.317			1	1202663		
608.3873			1	343160.94		
613.3433	613.343	-0.49	1	1050410.13	C28 H52 O14	(M+H)+
614.3459	614.3464	0.83	1	289136.91	C28 H52 O14	(M+H)+
615.3478	615.3489	1.75	1	52068.78	C28 H52 O14	(M+H)+
616.3511	616.3515	0.65	1	7165.96	C28 H52 O14	(M+H)+
657.3694			1	732294.69		
701.3954			1	438203.56		

MSMS Spectrum

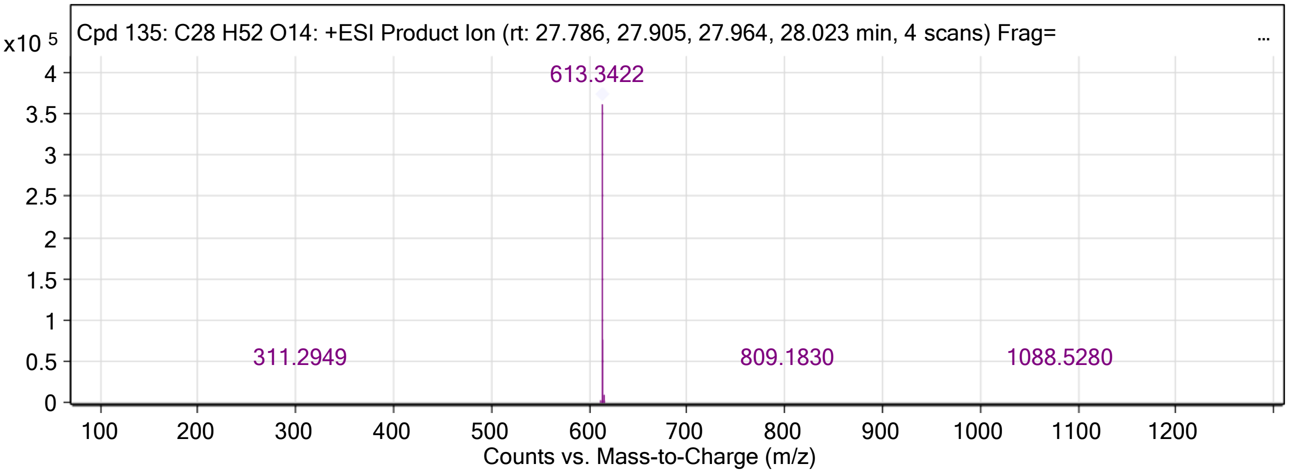

MS/MS Spectrum Peak List

m/z	*Calc m/z*	Diff(ppm)	z	Abund
133.0863	133.0859	-2.58		52.94
309.2768	309.2788	6.57		62.56
311.2949	311.2945	-1.56		66.78
391.2723	391.269	-8.42		15.97
582.322	582.3246	4.4		18.52
612.3936			2	2536.95
613.3422	613.343	1.2	1	361481.13
614.345			1	76010.47
615.3472			1	9005.79
615.4155			2	1973.06

Compound Label	*m/z*	RT	Algorithm	Mass
Cpd 136: C26 H48 O13	569.3171	28.185	Auto MS/MS	568.3096

Compound Chromatograms

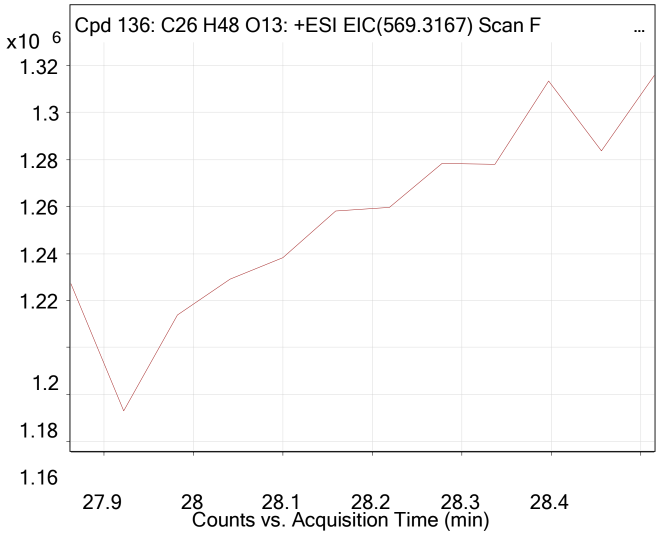

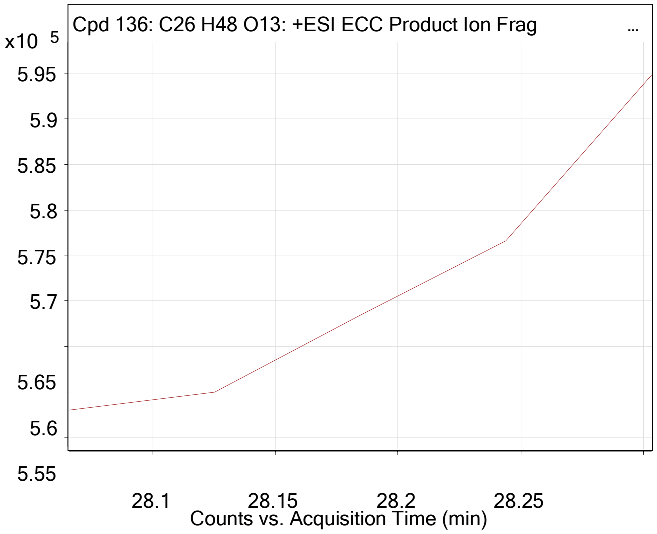

MS Spectrum

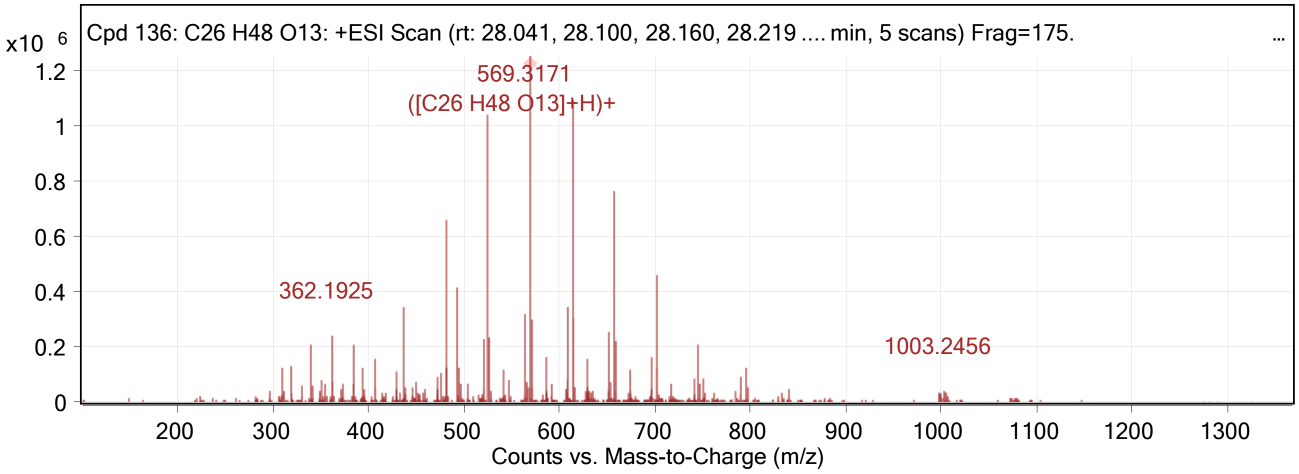

MS Zoomed Spectrum

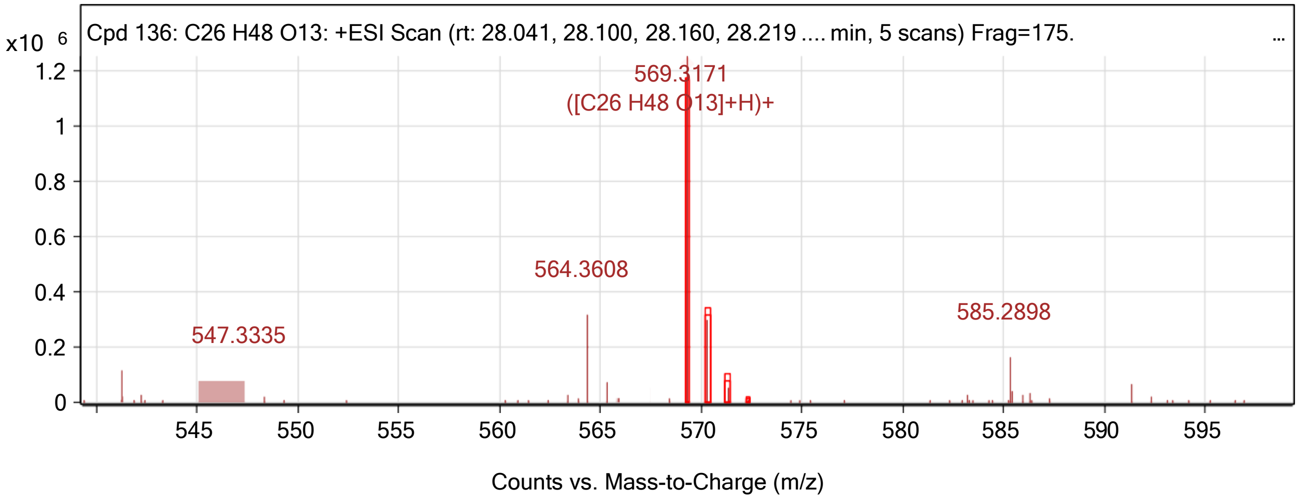

MS Spectrum Peak List

m/z	Calc *m/z*	Diff(ppm)	z	Abund	Formula	Ion
481.2637			1	661945.88		
493.3304			1	415486.88		
525.2905			1	1038701.5		
569.3171	569.3168	-0.52	1	1252734.75	C26 H48 O13	(M+H)+
570.3196	570.3202	1.09	1	296272.19	C26 H48 O13	(M+H)+
571.3215	571.3226	1.91	1	50838.63	C26 H48 O13	(M+H)+
572.3243	572.3253	1.65	1	7312.69	C26 H48 O13	(M+H)+
613.3433			1	1090813.75		
657.3694			1	759701.81		
701.3954			1	457605.75		

MSMS Spectrum

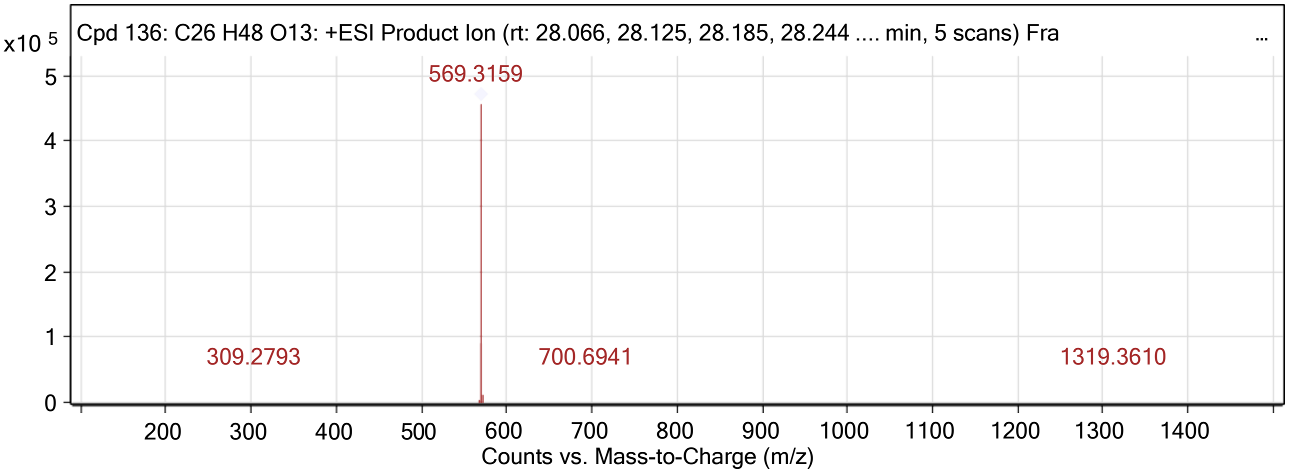

MS/MS Spectrum Peak List

m/z	Calc *m/z*	Diff(ppm)	z	Abund
133.0882	133.0859	-17.34		11.35
188.1034	188.1043	4.73		18.08
221.1398	221.1384	-6.64		14.19
231.1955	231.1955	-0.11		10.81
309.2793	309.2788	-1.54		39.13
311.2946	311.2945	-0.47		26.47
329.2309	329.2323	4.12		11.07
509.2902	509.2956	10.75		11.34
566.2953	283.1464	-500002.28	2	30.85
569.3159	569.3168	1.51	1	456134.41

Compound Label	*m/z*	RT	Algorithm	Mass
Cpd 137: C28 H52 O14	613.3433	28.201	Auto MS/MS	612.3358

Compound Chromatograms

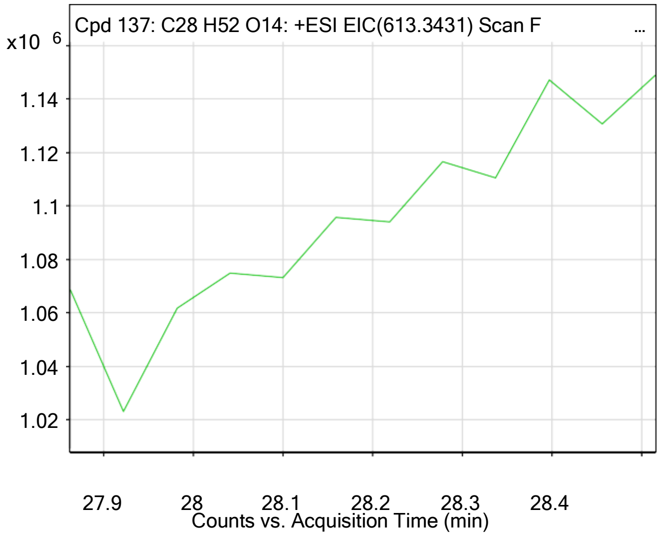

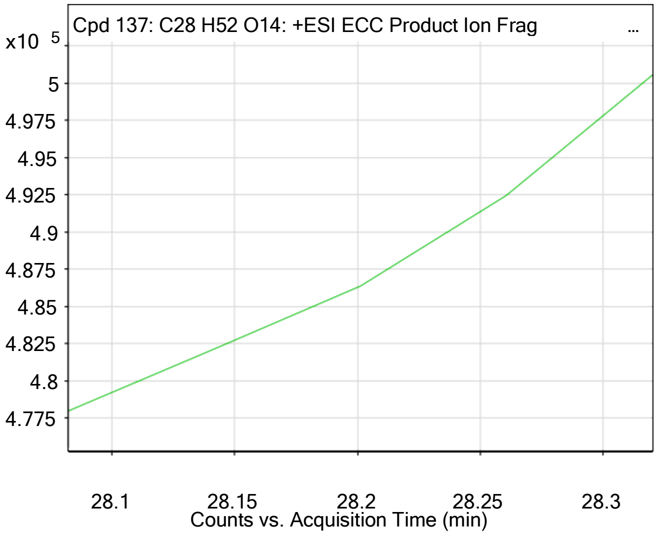

MS Spectrum

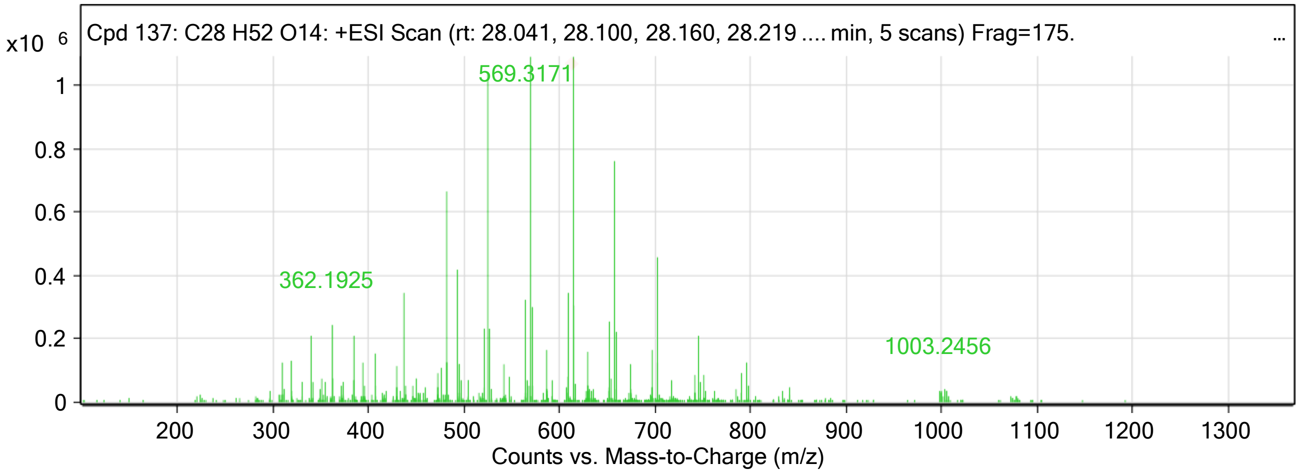

MS Zoomed Spectrum

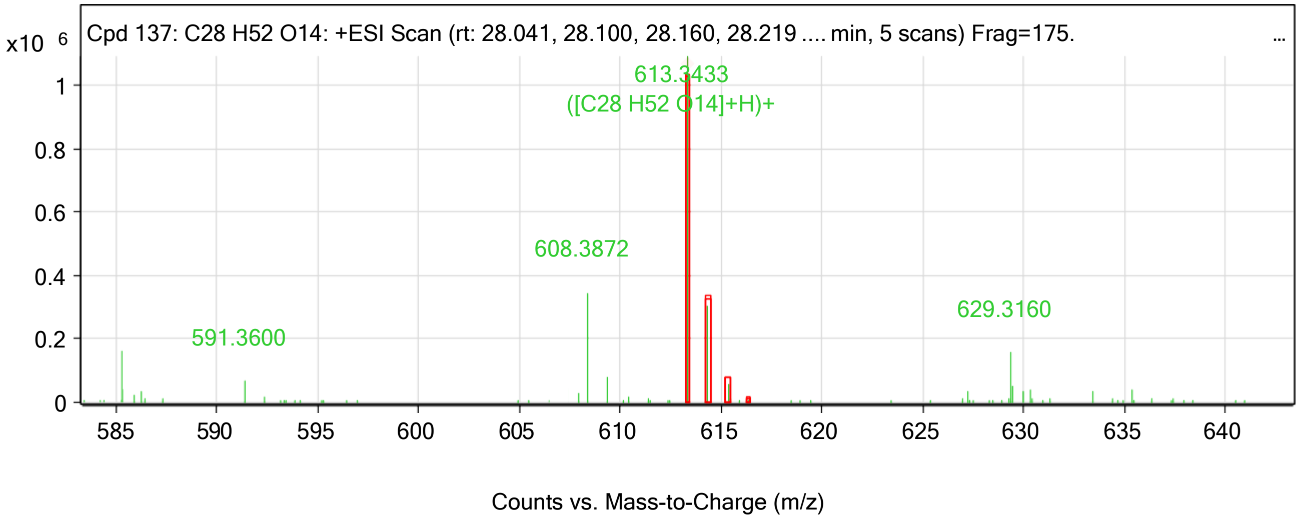

MS Spectrum Peak List

m/z	Calc *m/z*	Diff(ppm)	z	Abund	Formula	Ion
481.2637			1	661945.88		
525.2905			1	1038701.5		
569.3171			1	1252734.75		
613.3433	613.343	-0.55	1	1090813.75	C28 H52 O14	(M+H)+
614.3459	614.3464	0.76	1	302955.56	C28 H52 O14	(M+H)+
615.3478	615.3489	1.79	1	54592.06	C28 H52 O14	(M+H)+
616.3513	616.3515	0.38	1	7386.11	C28 H52 O14	(M+H)+
617.3635	617.354	-15.31	1	1396.1	C28 H52 O14	(M+H)+
657.3694			1	759701.81		

MSMS Spectrum

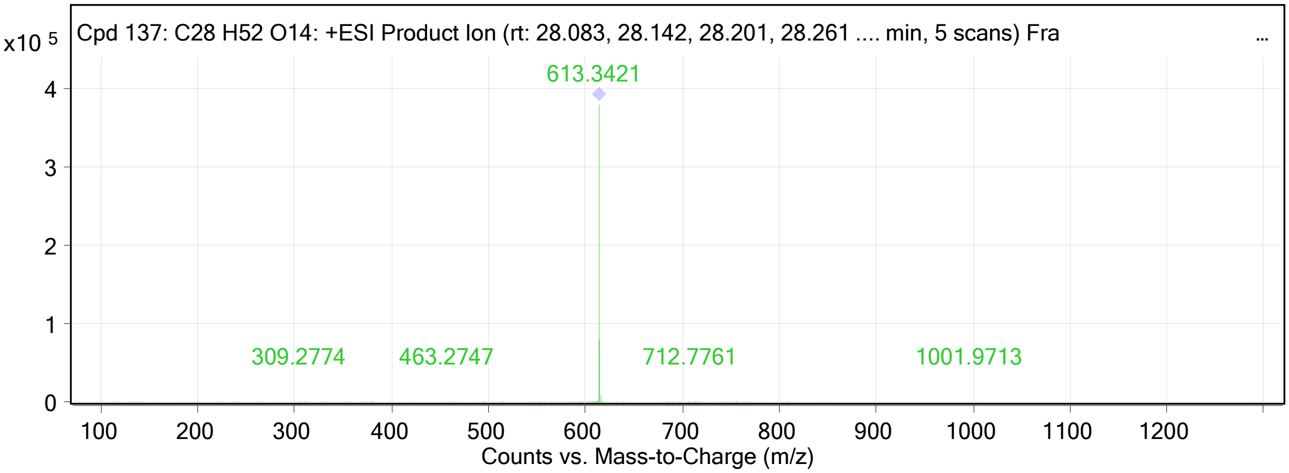

MS/MS Spectrum Peak List

m/z	*Calc m/z*	Diff(ppm)	z	Abund
115.0769	115.0754	-13.36		14.81
133.0856	133.0859	2.19		40.22
177.114	177.1121	-10.42		17.02
199.1311	199.1329	8.73		13.61
309.1916	309.1908	-2.64		15.08
309.2774	309.2788	4.44		97.68
311.295	311.2945	-1.63	1	44.14
597.3439	597.3481	7.05		14.01
610.3129	305.1595	-499995.06	2	58.24
613.3421	613.343	1.43	1	380161.84

Compound Label	*m/z*	RT	Algorithm	Mass
Cpd 138: C26 H48 O13	569.317	28.481	Auto MS/MS	568.3095

Compound Chromatograms

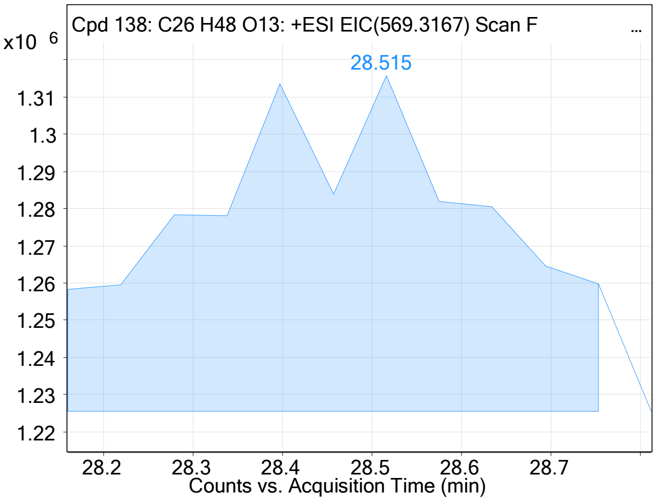

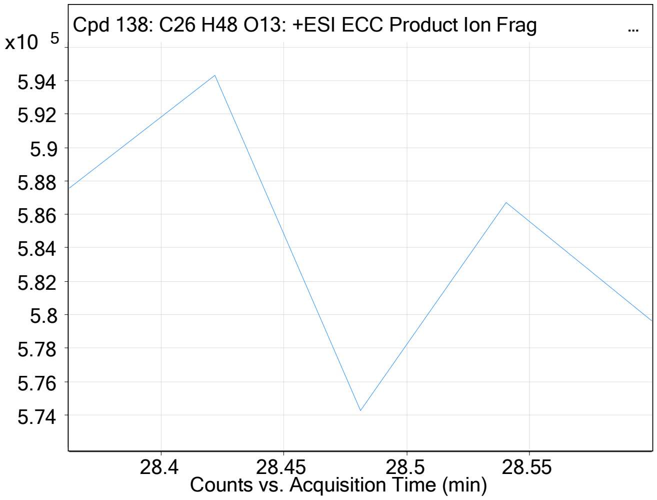

MS Spectrum

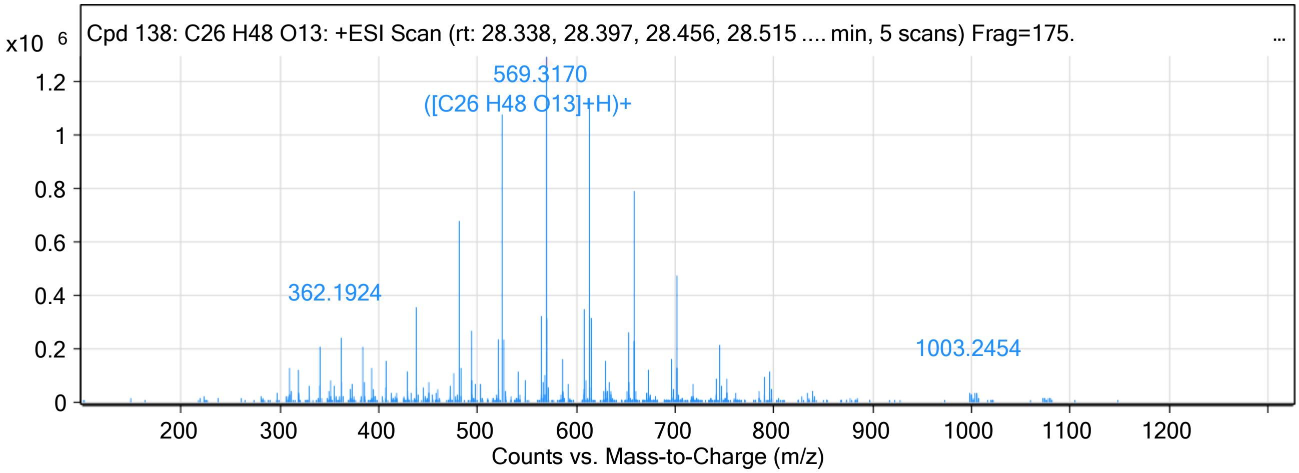

MS Zoomed Spectrum

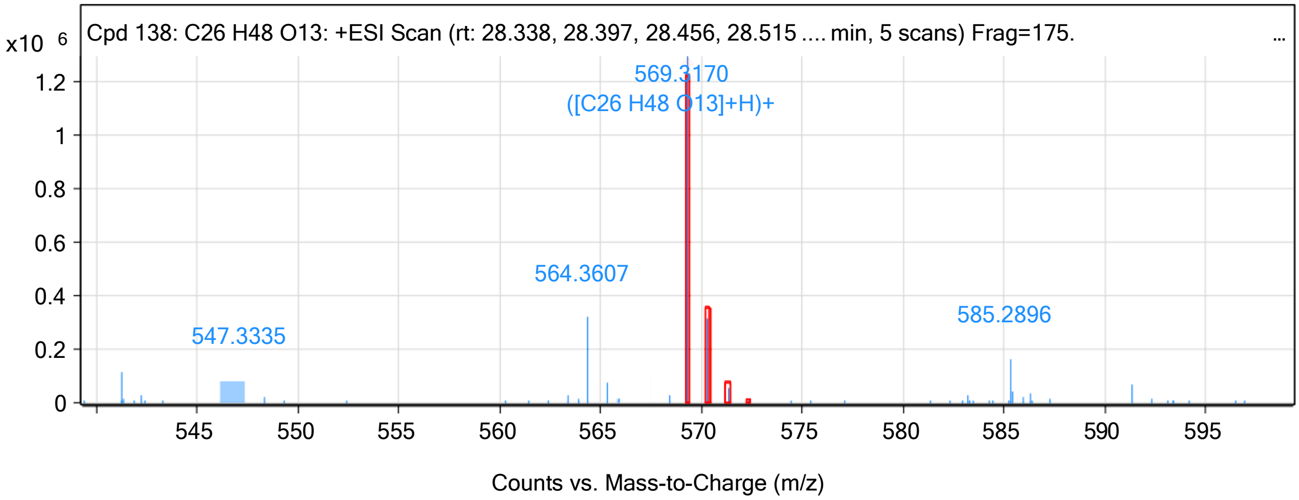

MS Spectrum Peak List

m/z	Calc *m/z*	Diff(ppm)	z	Abund	Formula	Ion
437.2371			1	351094.56		
481.2635			1	672225.38		
525.2904			1	1074061.88		
569.317	569.3168	-0.36	1	1294616.38	C26 H48 O13	(M+H)+
570.3195	570.3202	1.3	1	315245.41	C26 H48 O13	(M+H)+
571.3215	571.3226	1.9	1	53240.99	C26 H48 O13	(M+H)+
572.324	572.3253	2.32	1	7472.94	C26 H48 O13	(M+H)+
613.3432			1	1132630.5		
657.3693			1	787210.19		
701.3953			1	474025.66		

MSMS Spectrum

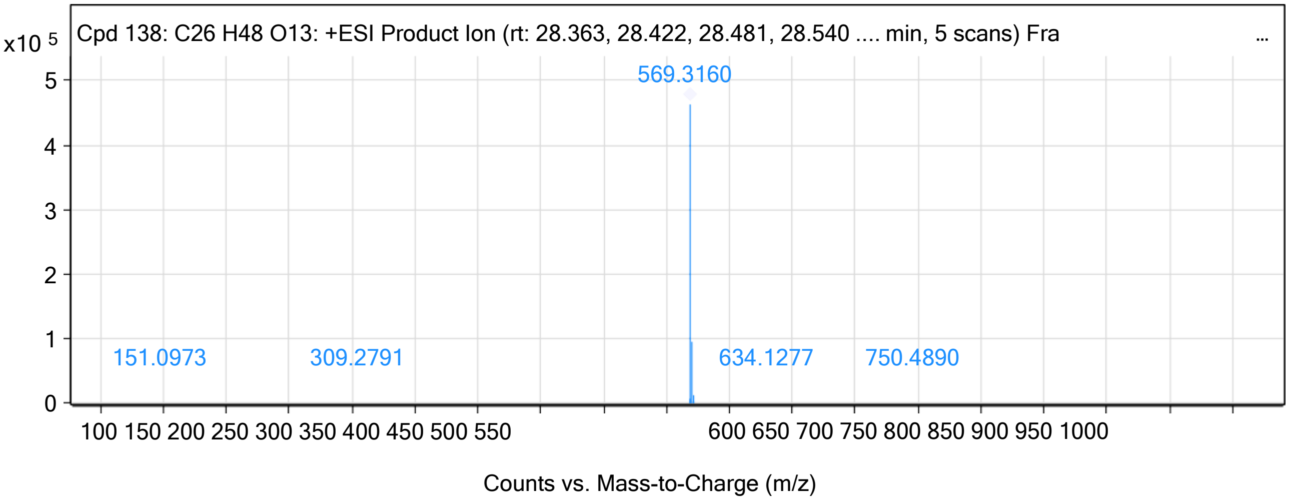

MS/MS Spectrum Peak List

m/z	*Calc m/z*	Diff(ppm)	z	Abund
133.0872	133.0859	-9.52		23.06
177.1113	177.1121	4.87		12.54
188.1023	188.1043	10.88		27.73
252.1655	252.172	25.89		13.63
309.2791	309.2788	-1		49.22
311.2954	311.2945	-2.92		40.97
329.2277	329.2323	13.84		12.59
497.268	497.2593	-17.54		22.54
539.3034	539.3062	5.15		13.97
569.316	569.3168	1.32	1	462977.09

Compound Label	*m/z*	RT	Algorithm	Mass
Cpd 139: C28 H52 O14	613.3432	28.498	Auto MS/MS	612.3357

Compound Chromatograms

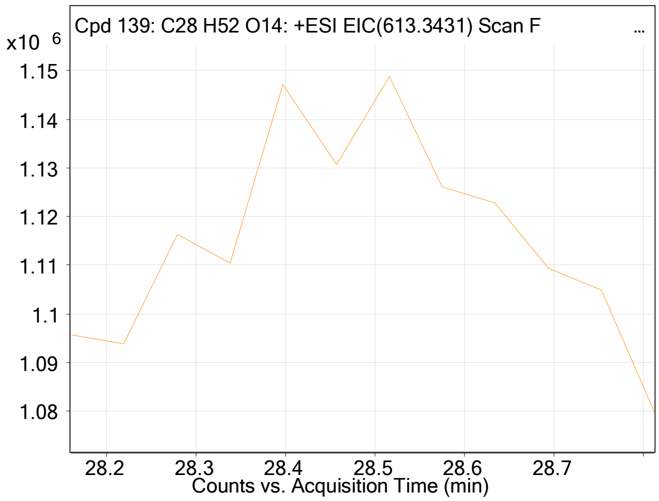

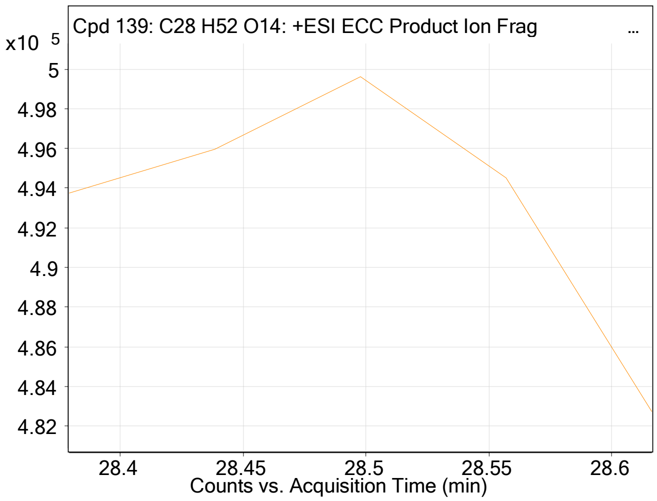

MS Spectrum

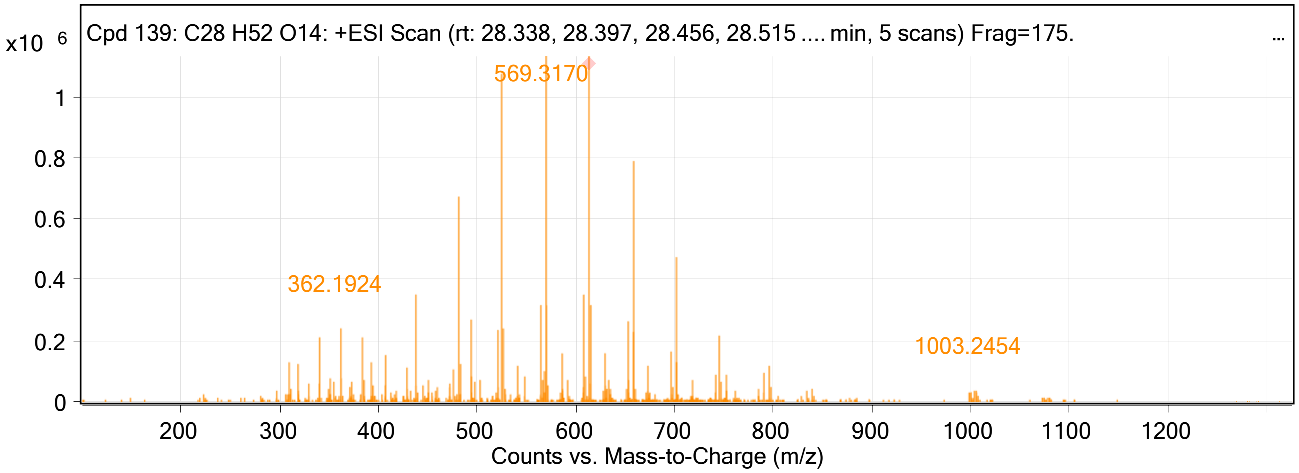

MS Zoomed Spectrum

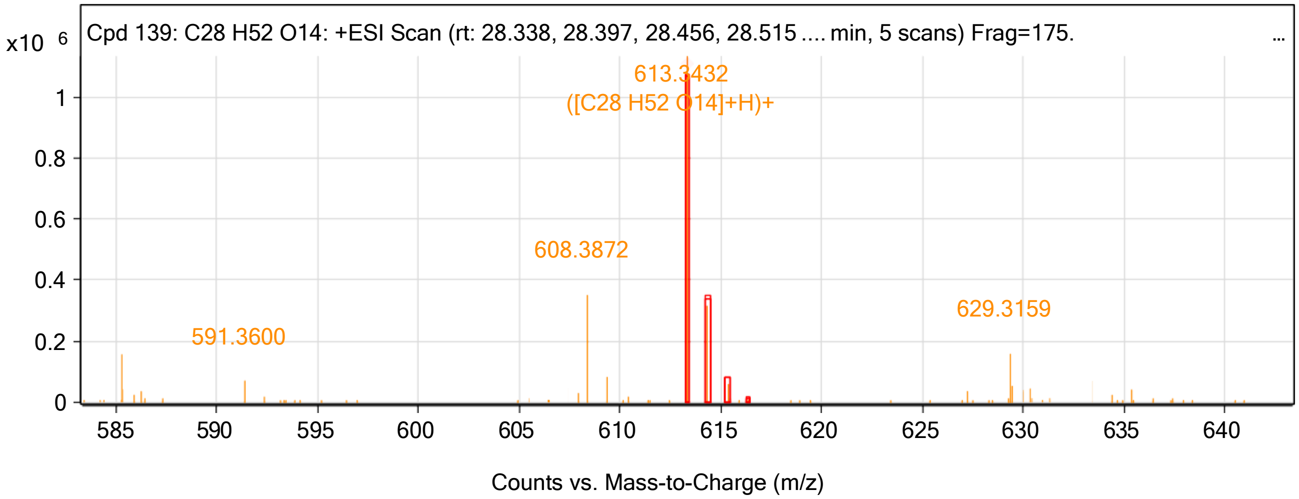

MS Spectrum Peak List

m/z	Calc *m/z*	Diff(ppm)	z	Abund	Formula	Ion
437.2371			1	351094.56		
481.2635			1	672225.38		
525.2904			1	1074061.88		
569.317			1	1294616.38		
613.3432	613.343	-0.32	1	1132630.5	C28 H52 O14	(M+H)+
614.3459	614.3464	0.9	1	312969.75	C28 H52 O14	(M+H)+
615.3477	615.3489	1.85	1	56123.29	C28 H52 O14	(M+H)+
616.3505	616.3515	1.66	1	7330.14	C28 H52 O14	(M+H)+
657.3693			1	787210.19		
701.3953			1	474025.66		

MSMS Spectrum

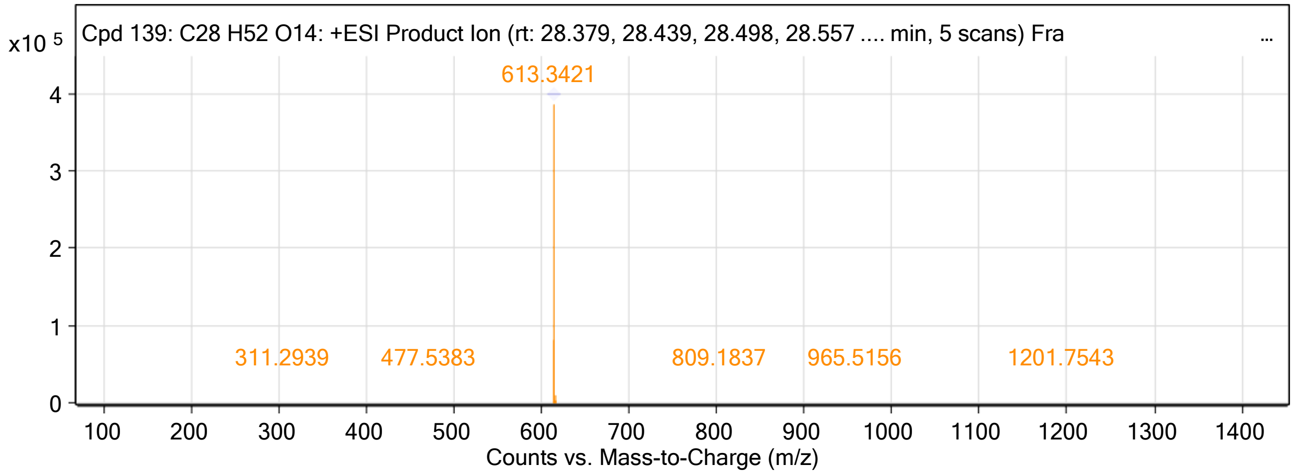

MS/MS Spectrum Peak List

m/z	*Calc m/z*	Diff(ppm)	z	Abund
103.0755	103.0754	-0.92		13.68
133.0849	133.0859	8	1	62.39
157.122	157.1223	2.03		18.53
188.1028	188.1043	8.03		13.39
265.2146	265.2162	5.97		13.8
287.182	287.1853	11.52		22.34
309.2773	309.2788	4.96	1	55.24
357.0661	357.0664	0.72		19.26
610.3194	610.3195	0.12	1	33.43
613.3421	613.343	1.44	1	385610.72

Compound Label	*m/z*	RT	Algorithm	Mass
Cpd 140: C26 H48 O13	569.3171	28.756	Auto MS/MS	568.3096

Compound Chromatograms

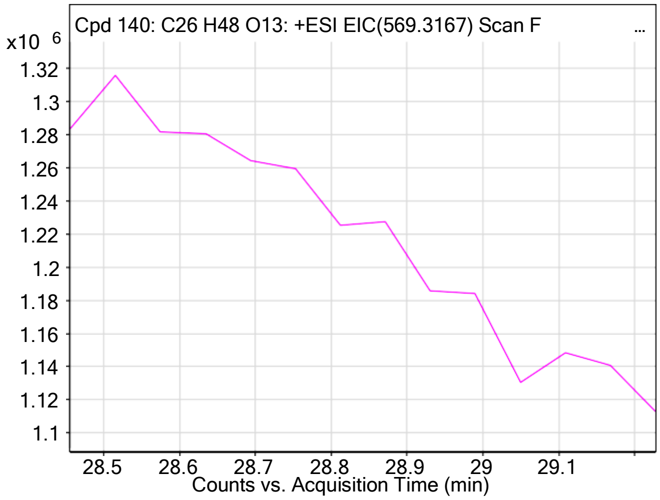

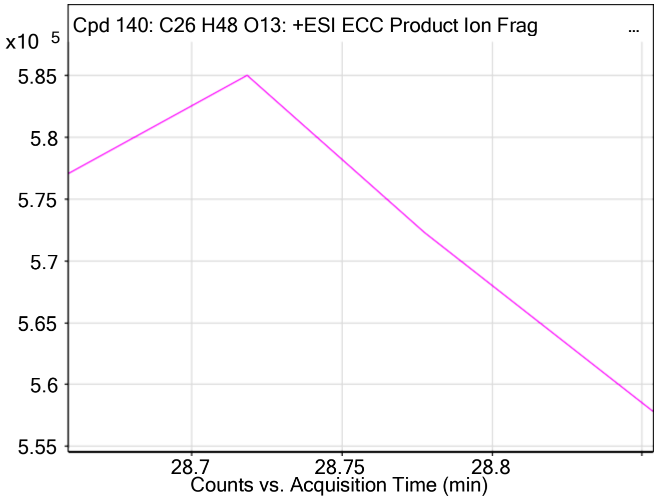

MS Spectrum

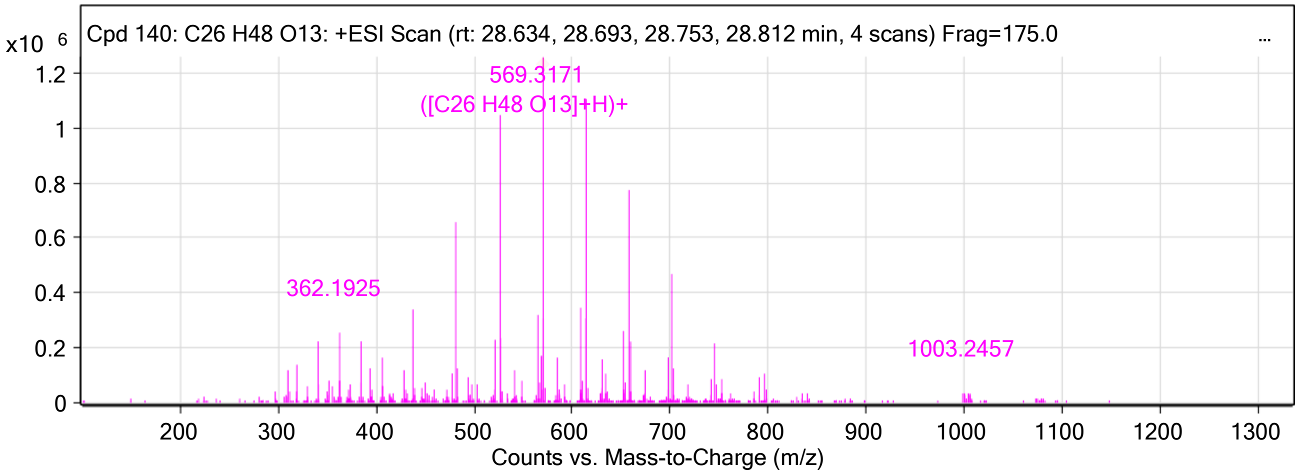

MS Zoomed Spectrum

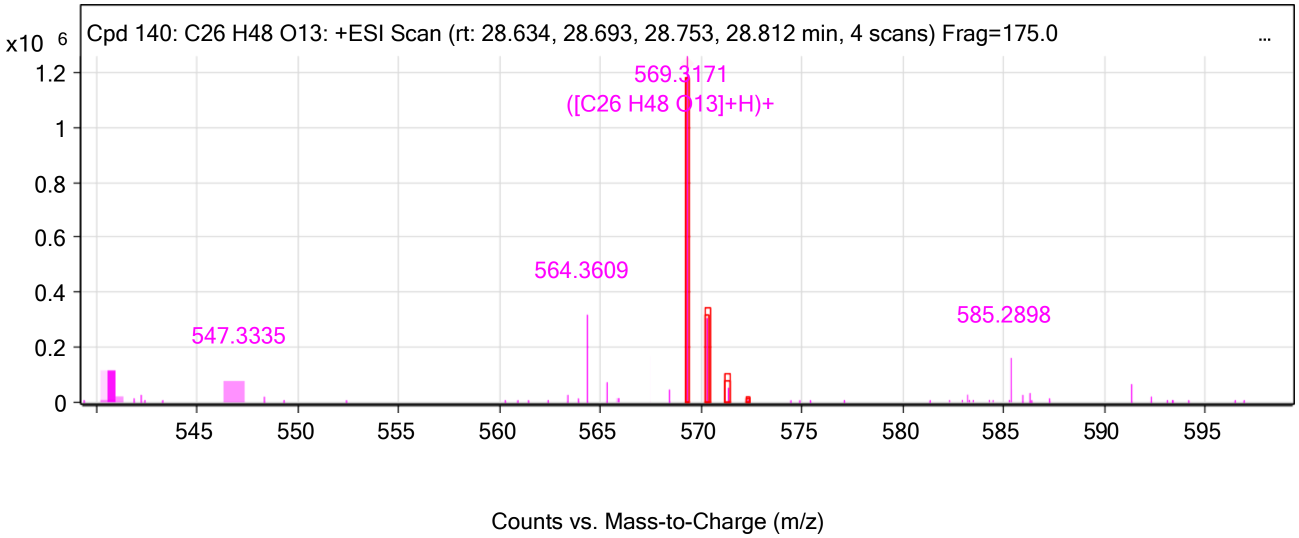

MS Spectrum Peak List

m/z	Calc *m/z*	Diff(ppm)	z	Abund	Formula	Ion
481.2637			1	657220.69		
525.2906			1	1043848.75		
569.3171	569.3168	-0.64	1	1257624.5	C26 H48 O13	(M+H)+
570.3197	570.3202	0.95	1	302069.47	C26 H48 O13	(M+H)+
571.3217	571.3226	1.62	1	52255.59	C26 H48 O13	(M+H)+
572.3248	572.3253	0.93	1	6459.85	C26 H48 O13	(M+H)+
608.3874			1	345995.5		
613.3433			1	1104274.5		
657.3695			1	770959.5		

MSMS Spectrum

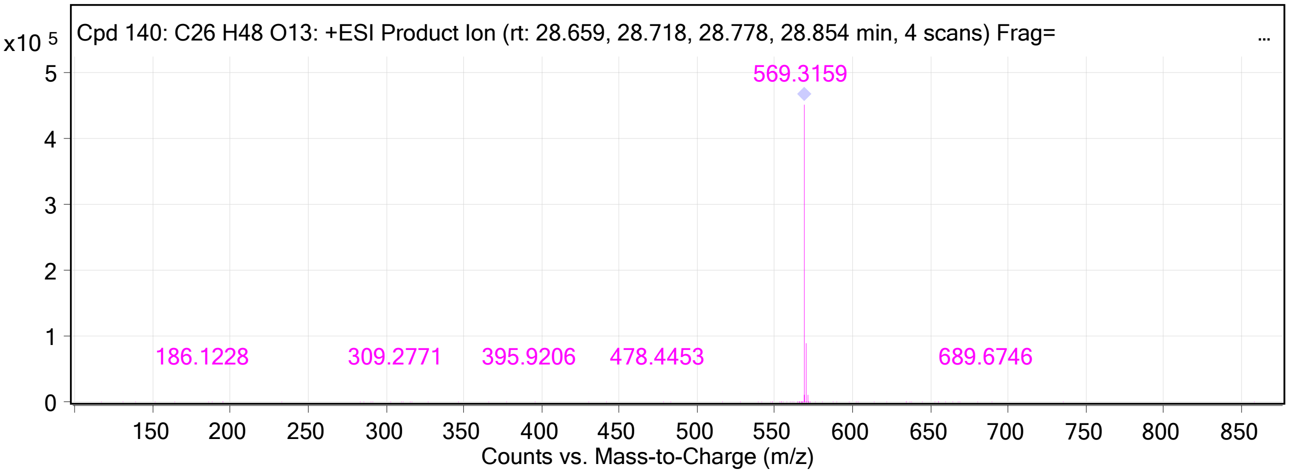

MS/MS Spectrum Peak List

m/z	*Calc m/z*	Diff(ppm)	z	Abund
117.0916	117.091	-4.98		33.72
131.0704	131.0703	-1.3		16.25
139.1134	139.1117	-11.7		15.11
186.1228	186.125	11.88		26.22
283.2634	283.2632	-1.03		38.78
285.2013	285.206	16.44		18.01
309.2771	309.2788	5.36		54.71
311.2931	311.2945	4.5		14.8
346.2744	346.2714	-8.61		17.04
569.3159	569.3168	1.5	1	451880.5

Compound Label	*m/z*	RT	Algorithm	Mass
Cpd 141: C28 H52 O14	613.3433	28.786	Auto MS/MS	612.3358

Compound Chromatograms

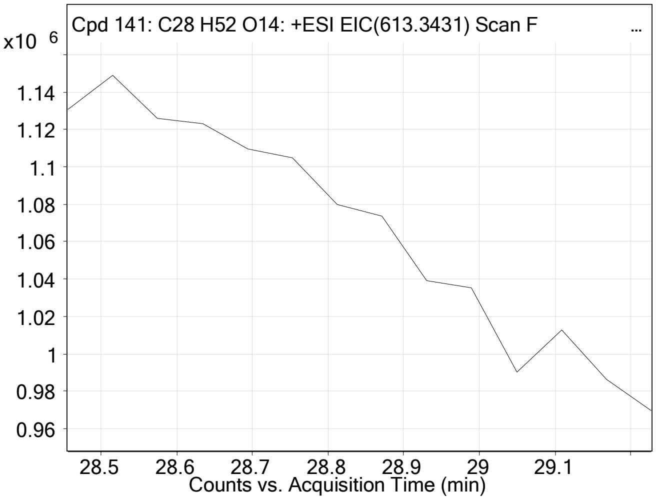

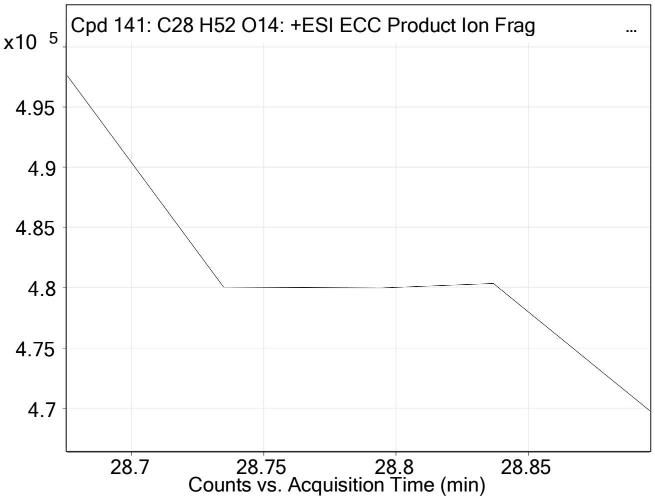

MS Spectrum

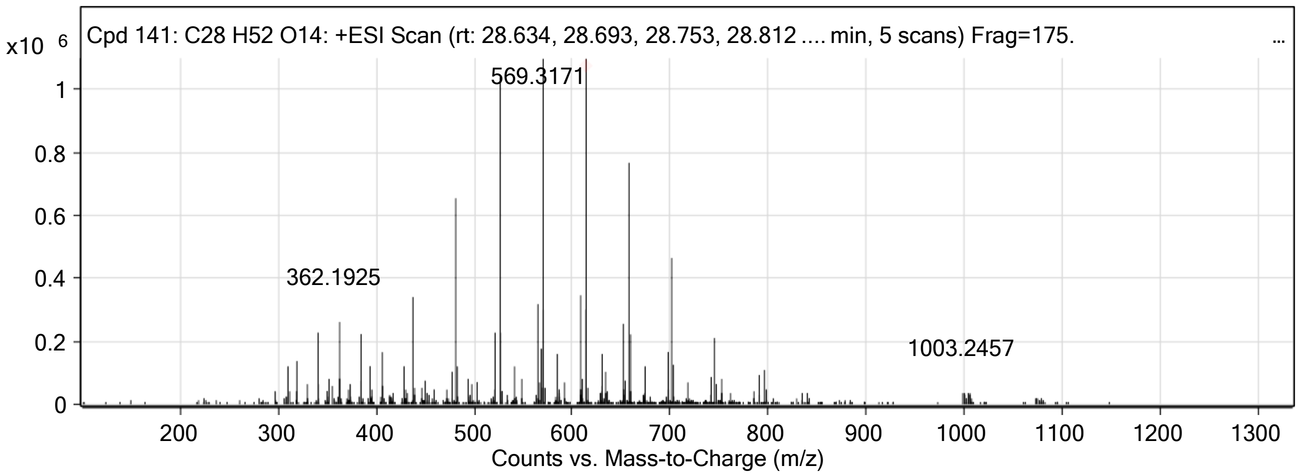

MS Zoomed Spectrum

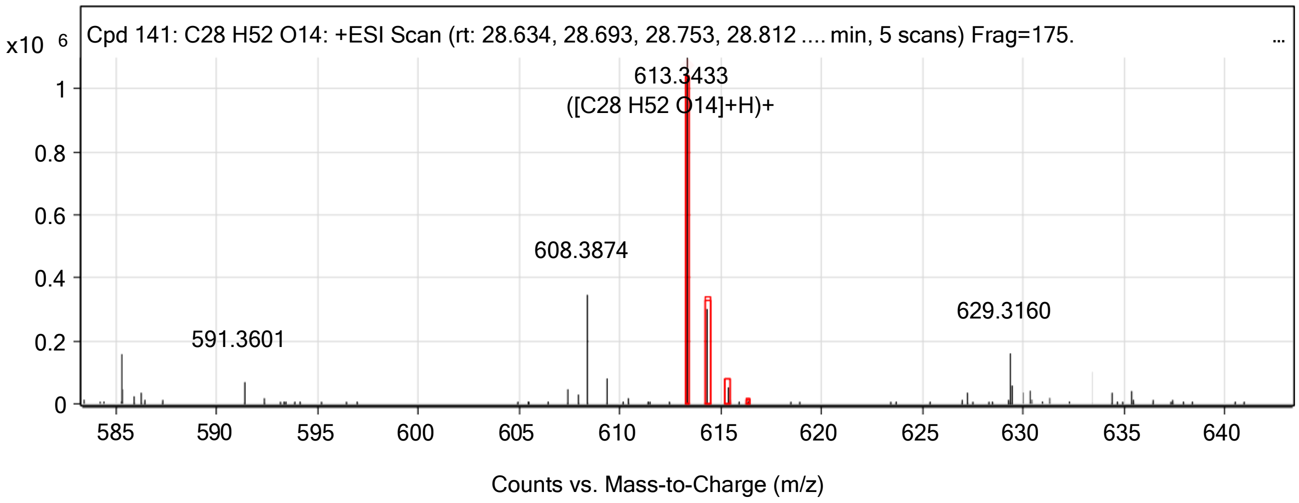

MS Spectrum Peak List

m/z	Calc *m/z*	Diff(ppm)	z	Abund	Formula	Ion
481.2637			1	652866.69		
525.2906			1	1038022.69		
569.3171			1	1251585.13		
608.3874			1	344043.16		
613.3433	613.343	-0.51	1	1098179.75	C28 H52 O14	(M+H)+
614.3459	614.3464	0.77	1	302504.31	C28 H52 O14	(M+H)+
615.3479	615.3489	1.55	1	53740.61	C28 H52 O14	(M+H)+
616.3514	616.3515	0.28	1	7319.02	C28 H52 O14	(M+H)+
657.3695			1	766742.75		
701.3955			1	462257.59		

MSMS Spectrum

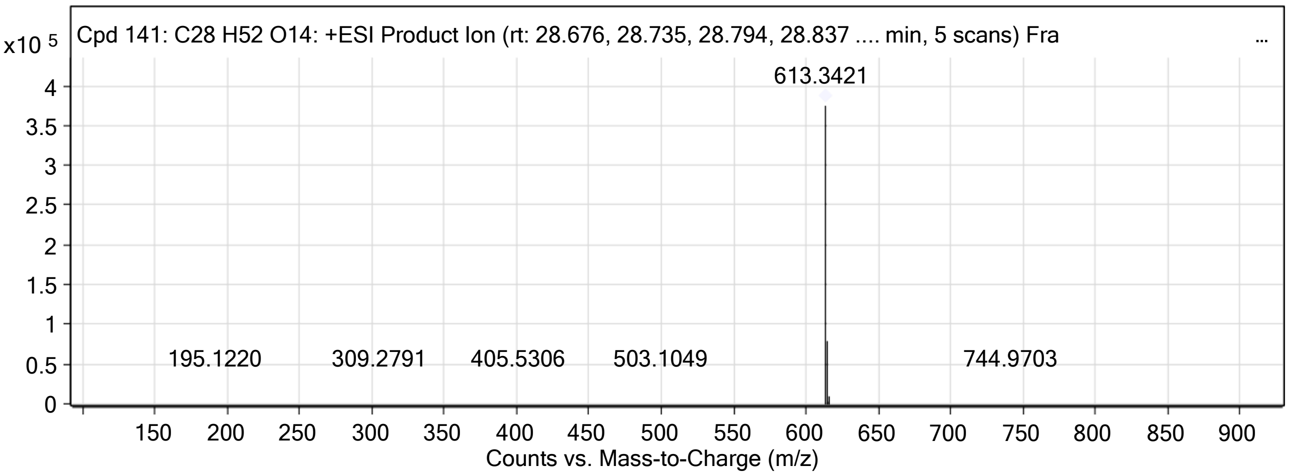

MS/MS Spectrum Peak List

m/z	*Calc m/z*	Diff(ppm)	z	Abund
133.0855	133.0859	3.52		13.35
177.1142	177.1121	-11.63		20.72
283.2635	283.2632	-1.33	1	26.6
296.1481	296.1466	-5.1		18.13
309.2791	309.2788	-1.04	1	61.36
357.0692	357.0664	-7.82		28.05
490.3438	490.35	12.67		11.71
504.3227	504.3293	13.08		10.61
609.3124	304.6556	-500001.06	2	67.23
613.3421	613.343	1.4	1	374706.16

Compound Label	*m/z*	RT	Algorithm	Mass
Cpd 142: C26 H48 O13	569.317	28.972	Auto MS/MS	568.3095

Compound Chromatograms

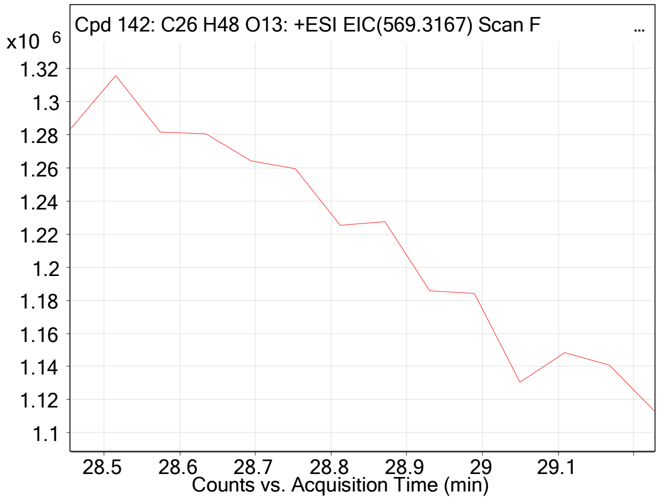

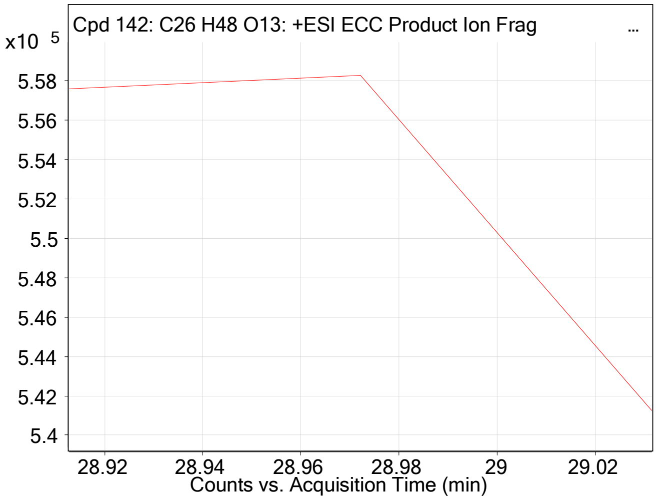

MS Spectrum

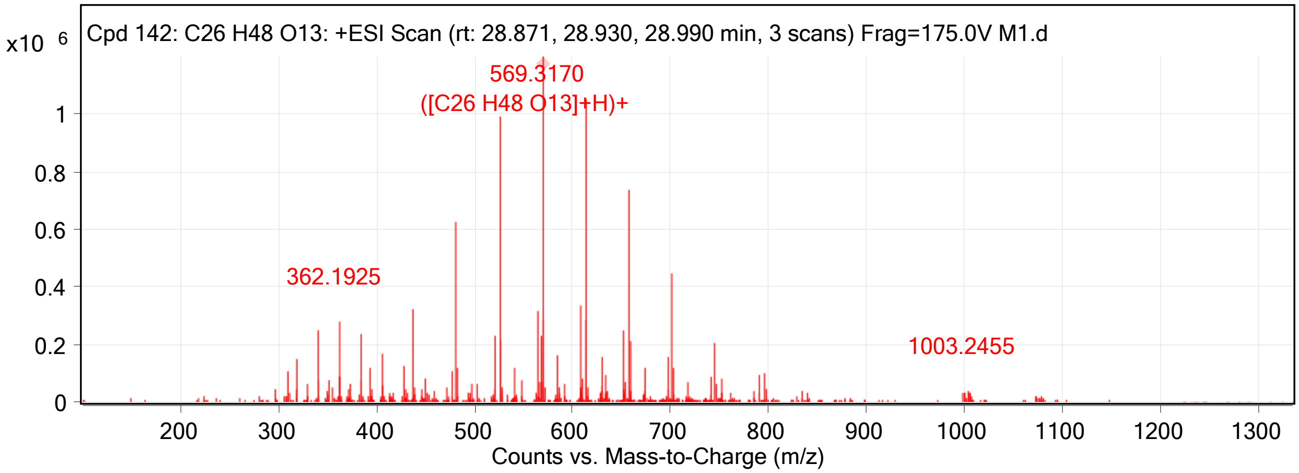

MS Zoomed Spectrum

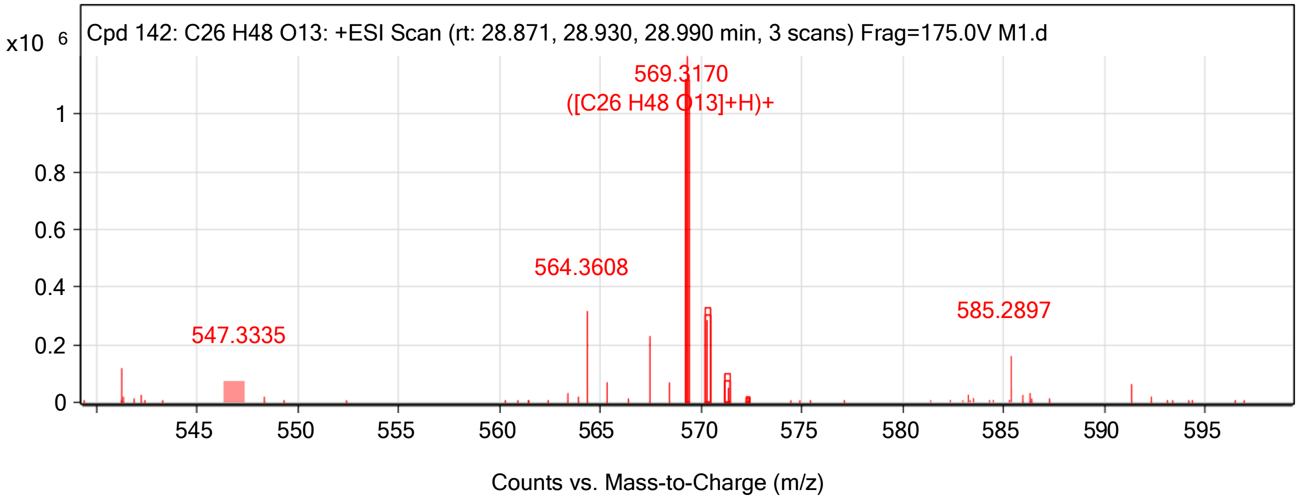

MS Spectrum Peak List

m/z	Calc *m/z*	Diff(ppm)	z	Abund	Formula	Ion
481.2636			1	624163.81		
525.2905			1	988199.5		
569.317	569.3168	-0.43	1	1199141.13	C26 H48 O13	(M+H)+
570.3195	570.3202	1.2	1	283641.09	C26 H48 O13	(M+H)+
571.3215	571.3226	1.9	1	48125.69	C26 H48 O13	(M+H)+
572.3243	572.3253	1.65	1	6077.48	C26 H48 O13	(M+H)+
608.3873			1	333581		
613.3433			1	1049477.5		
657.3694			1	734032.44		
701.3954			1	443529.34		

MSMS Spectrum

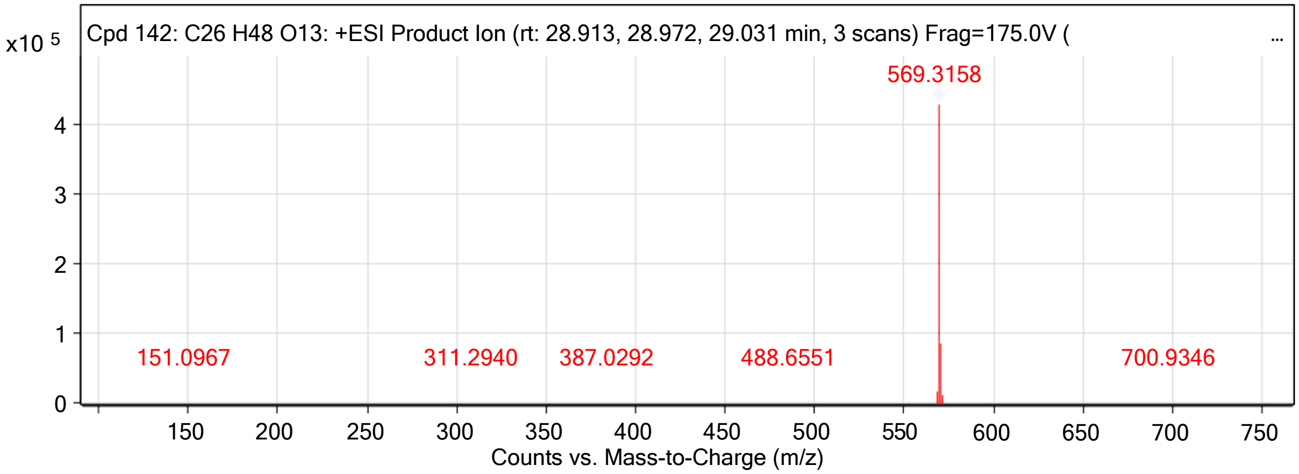

MS/MS Spectrum Peak List

m/z	Calc *m/z*	Diff(ppm)	z	Abund
133.0864	133.0859	-3.54		22.31
166.1299	166.1352	31.76		18.4
283.2646	283.2632	-5.13		20.51
309.2836	309.2788	-15.66		19.56
311.294	311.2945	1.33		44.58
318.2392	318.2401	2.69		20.52
453.3206	453.3211	1.1		21.09
538.2932	538.2984	9.66		30.17
566.3007	283.1464	-500006.99	2	17.51
569.3158	569.3168	1.62	1	429930.16

Compound Label	*m/z*	RT	Algorithm	Mass
Cpd 143: C28 H52 O14	613.3431	29.074	Auto MS/MS	612.3356

Compound Chromatograms

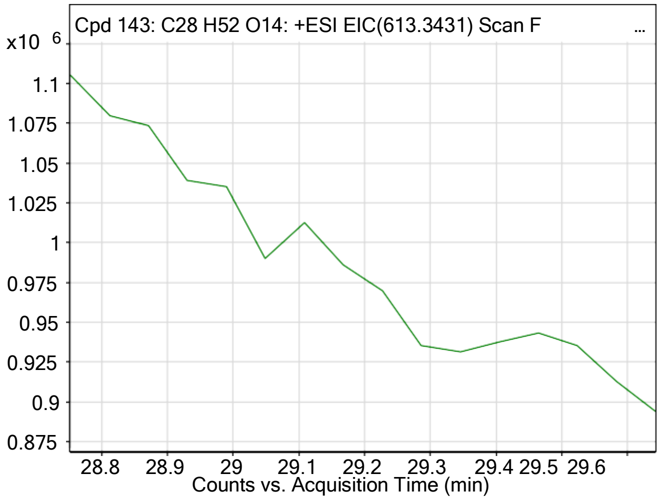

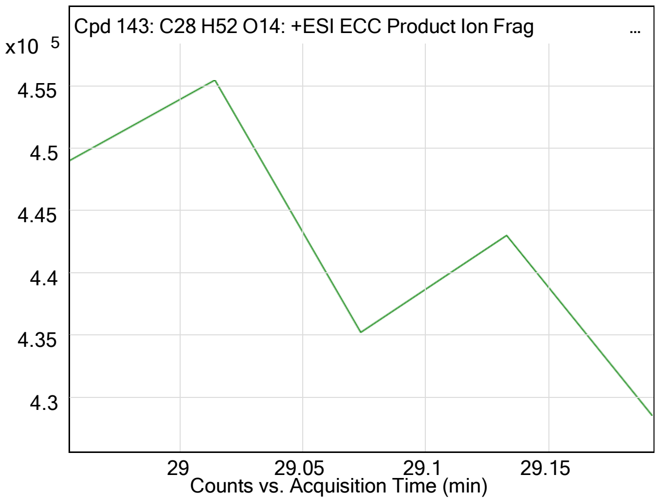

MS Spectrum

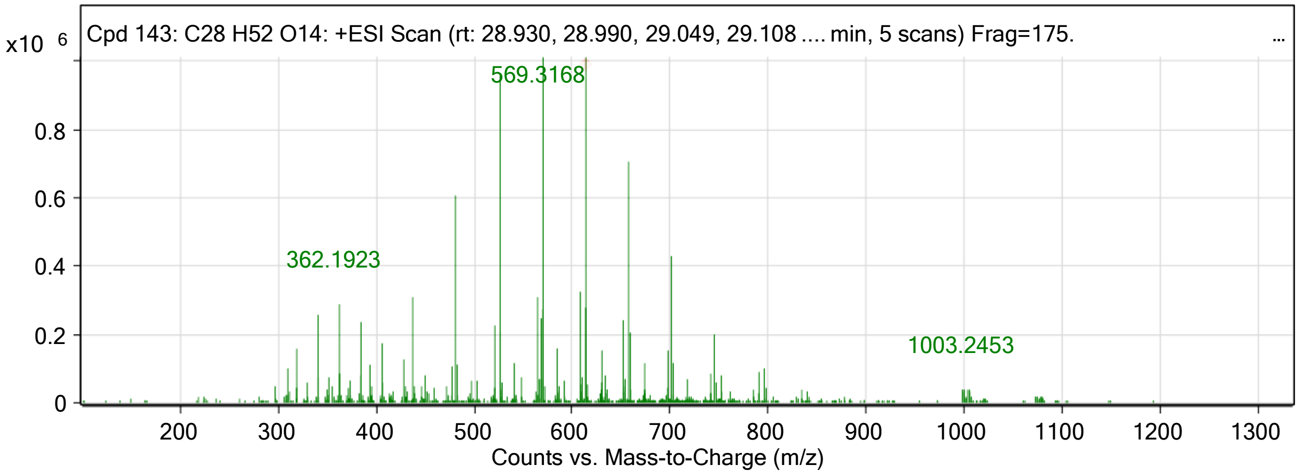

MS Zoomed Spectrum

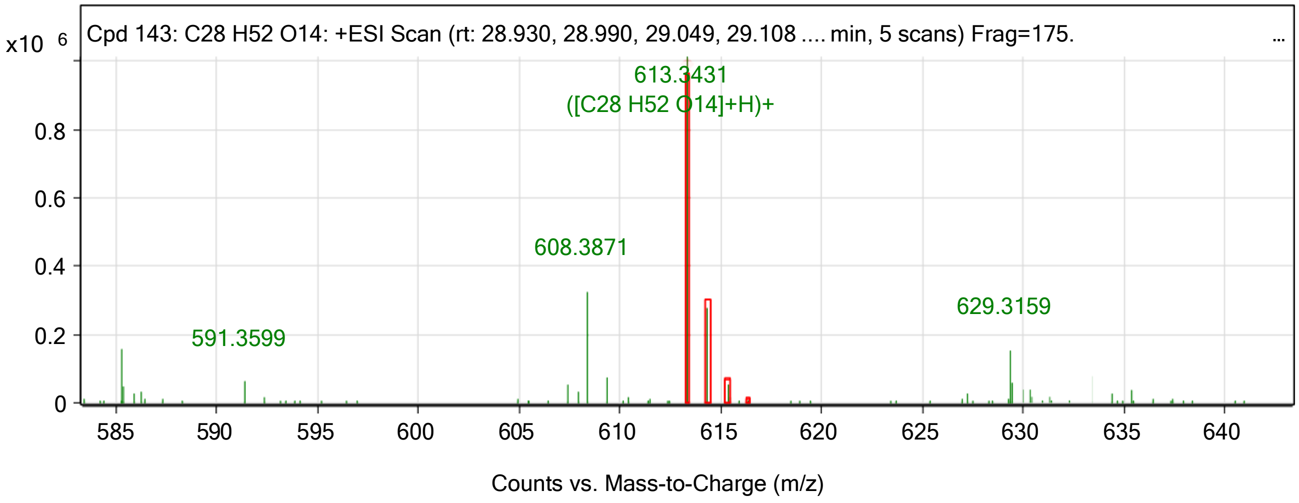

MS Spectrum Peak List

m/z	Calc *m/z*	Diff(ppm)	z	Abund	Formula	Ion
481.2635			1	604136.13		
525.2904			1	954885		
569.3168			1	1157939		
608.3871			1	324997.31		

613.3431	613.343	-0.16	1	1012804.38	C28 H52 O14	(M+H)+
614.3457	614.3464	1.21	1	276633.28	C28 H52 O14	(M+H)+
615.3476	615.3489	2.03	1	50003.73	C28 H52 O14	(M+H)+
616.3508	616.3515	1.24	1	6599.56	C28 H52 O14	(M+H)+
657.3692			1	703213.44		

MSMS Spectrum

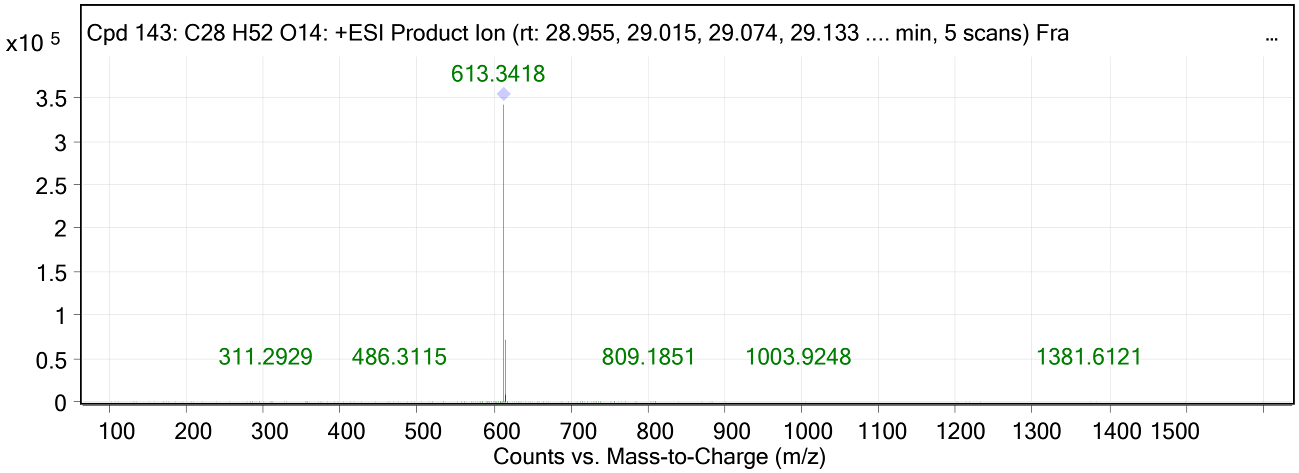

MS/MS Spectrum Peak List

m/z	*Calc m/z*	Diff(ppm)	z	Abund
103.0753	103.0754	0.19		15.42
177.1122	177.1121	-0.17		34.67
296.148	296.1466	-4.76		22.38
309.2774	309.2788	4.54		19.14
311.2929	311.2945	4.93	1	79.52
373.0995	373.0977	-4.98		21.58
486.3115	486.3187	14.81		20.23
514.2904	514.2984	15.58		15.65
610.3161	610.3195	5.62	1	54.41
613.3418	613.343	1.87	1	342543.19

Compound Label	*m/z*	RT	Algorithm	Mass
Cpd 144: C22 H42 N3 O11	525.2901	29.18	Auto MS/MS	524.2828

Compound Chromatograms

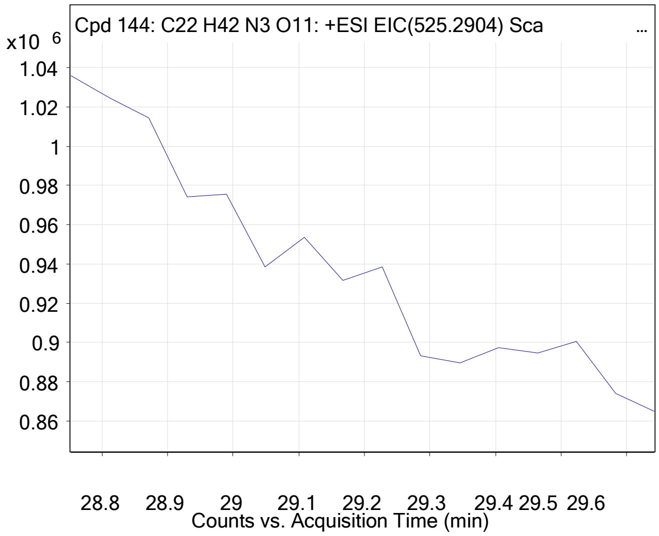

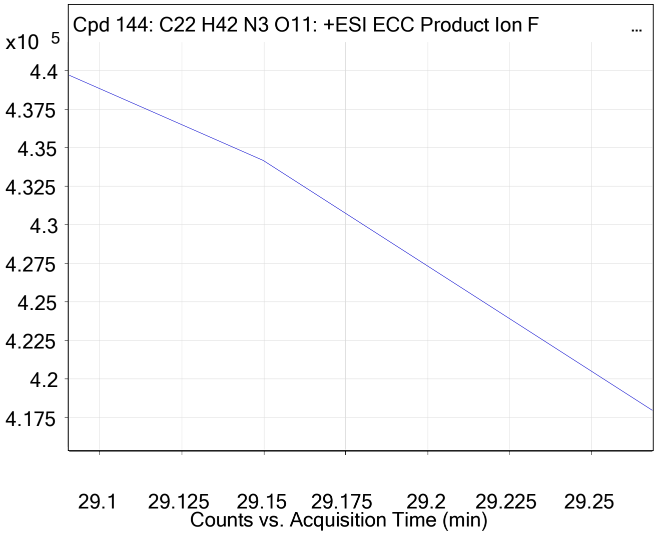

MS Spectrum

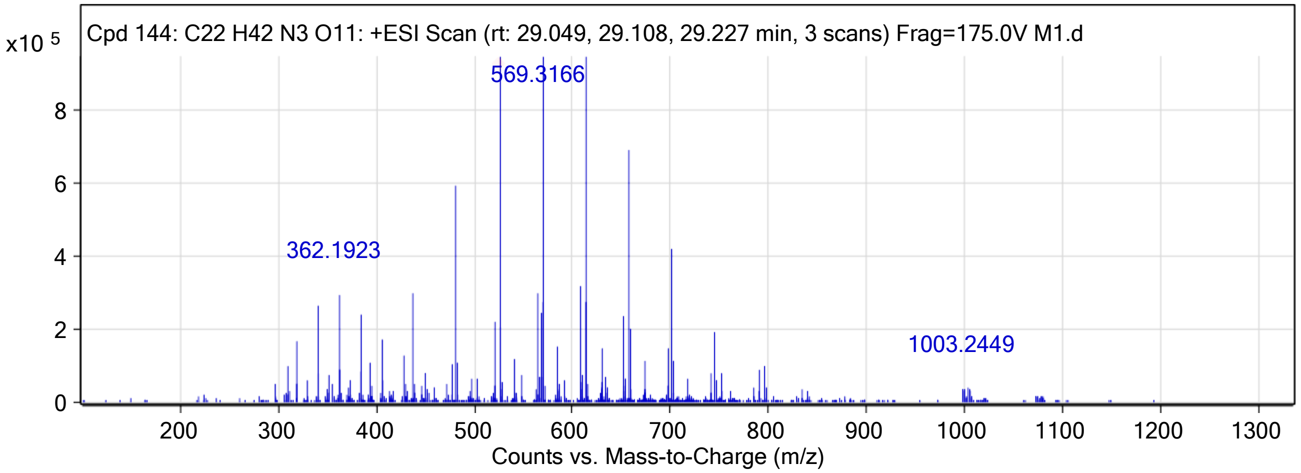

MS Zoomed Spectrum

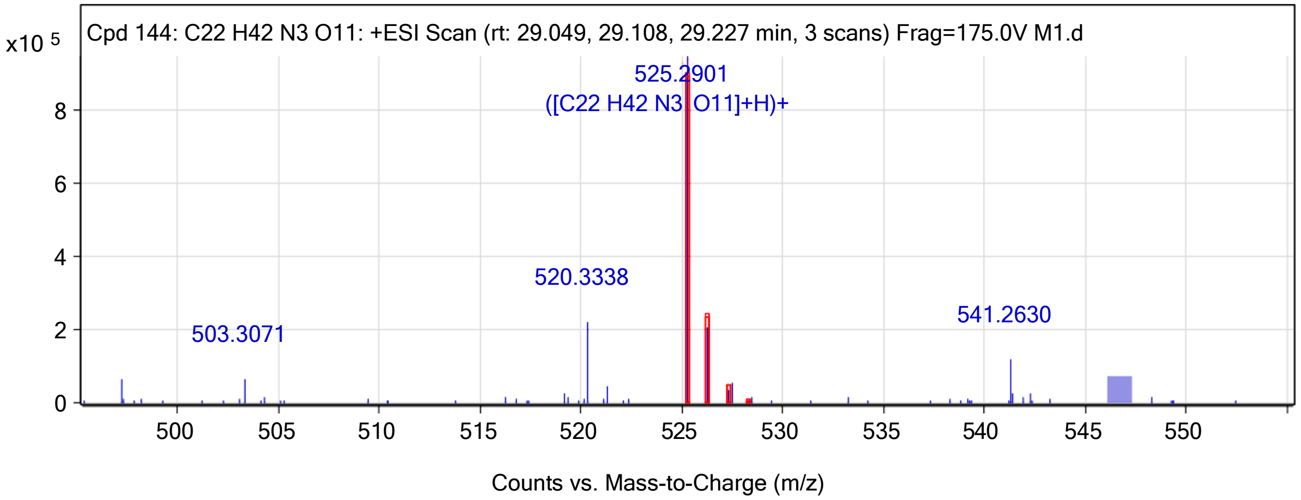

MS Spectrum Peak List

m/z	Calc *m/z*	Diff(ppm)	z	Abund	Formula	Ion
481.2633			1	589497.81		
525.2901	525.2892	-1.78	1	943682	C22 H42 N3 O11	(M+H)+
526.2928	526.2924	-0.82	1	204165.88	C22 H42 N3 O11	(M+H)+
527.2953	527.2947	-1.11	1	33600.35	C22 H42 N3 O11	(M+H)+
528.2983	528.2972	-2.01	1	4508.36	C22 H42 N3 O11	(M+H)+
569.3166			1	1130770.38		
608.3869			1	314310.03		
613.3428			1	990941		
657.3689			1	684617.75		
701.3949			1	419705.13		

MSMS Spectrum

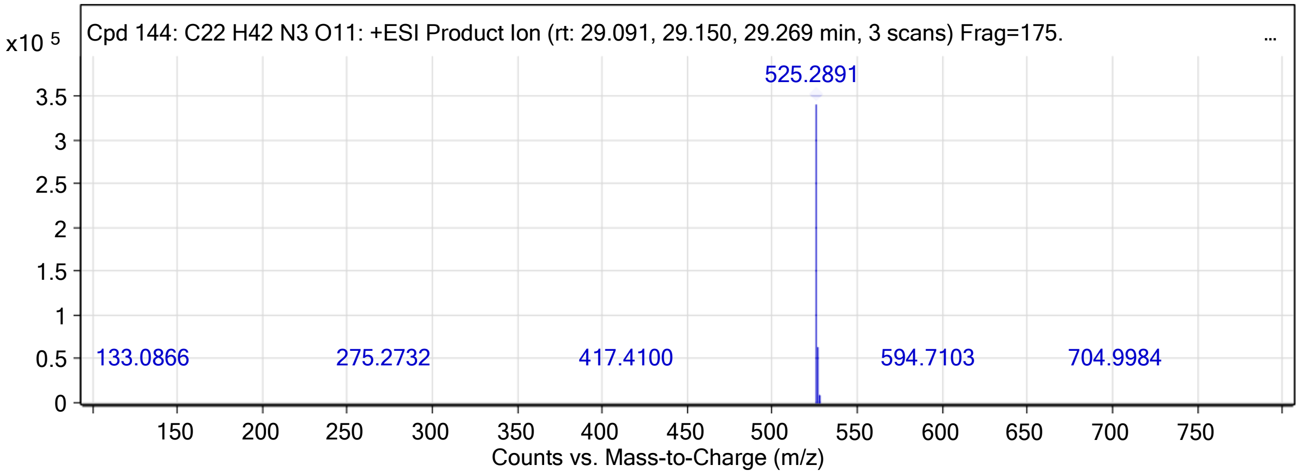

MS/MS Spectrum Peak List

m/z	*Calc m/z*	Diff(ppm)	z	Abund
133.0866	133.0859	-4.85		56.8
141.1282	141.1274	-5.93		18.34
177.1131	177.1121	-5.69		31.32
239.1455	239.1476	8.81		18.06
275.2732	275.2733	0.37		19.38
283.2633	283.2632	-0.4		17.8
311.2924	311.2931	2.4		17.9
322.2092	322.2098	1.84		18.43
495.2624	247.6334	-499995.52	2	18.34
525.2891	525.2892	0.22	1	341041.31

Compound Label	*m/z*	RT	Algorithm	Mass
Cpd 145: C26 H48 O13	569.3168	29.319	Auto MS/MS	568.3094

Compound Chromatograms

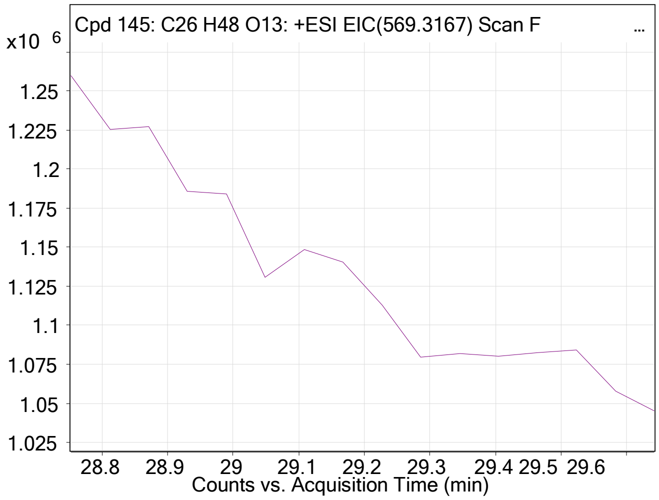

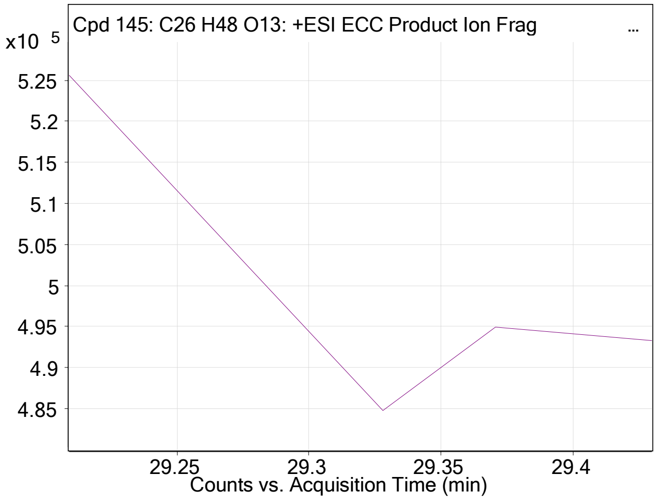

MS Spectrum

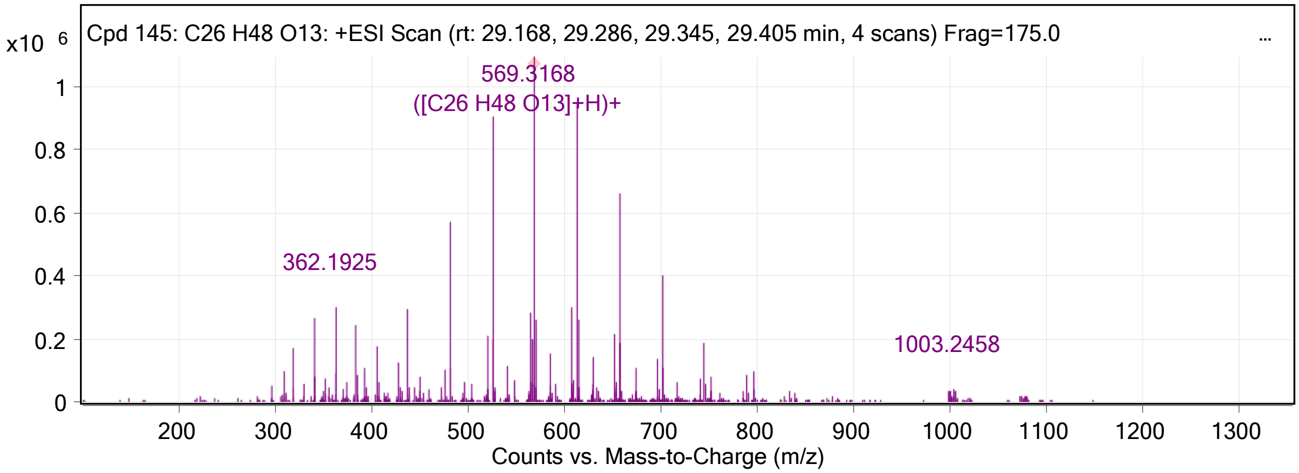

MS Zoomed Spectrum

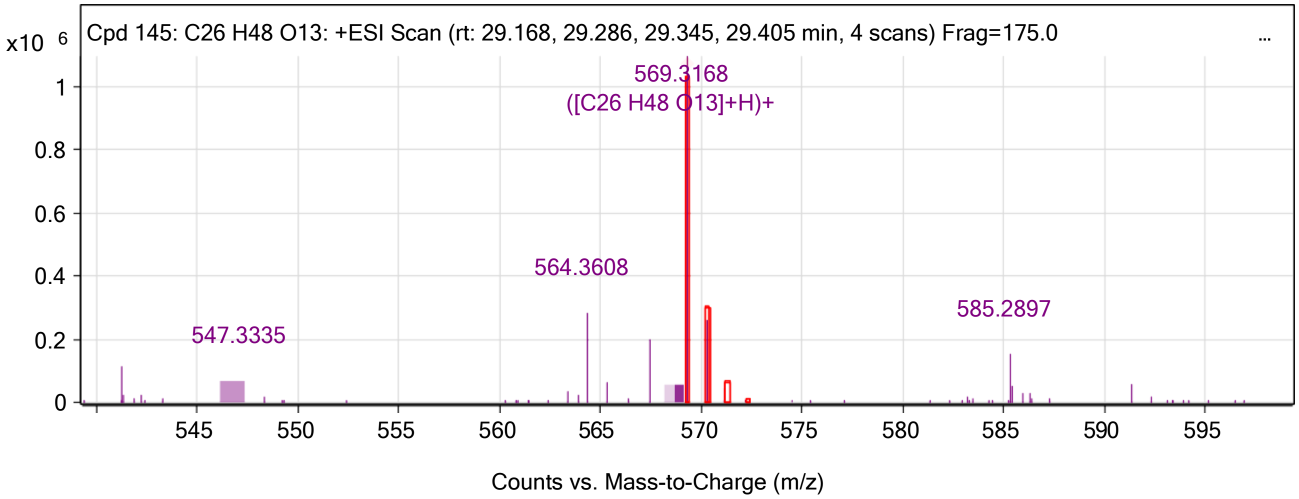

MS Spectrum Peak List

m/z	Calc m/z	Diff(ppm)	z	Abund	Formula	Ion
362.1925			2	299917.5		
481.2636			1	570391.63		
525.2905			1	903126.25		
569.3168	569.3168	-0.09	1	1095542	C26 H48 O13	(M+H)+
570.3195	570.3202	1.27	1	261198.56	C26 H48 O13	(M+H)+
571.3214	571.3226	2.04	1	44018.05	C26 H48 O13	(M+H)+
572.3244	572.3253	1.6	1	5649.51	C26 H48 O13	(M+H)+
613.3432			1	947812.88		
657.3694			1	660733.25		
701.3954			1	404065.31		

MSMS Spectrum

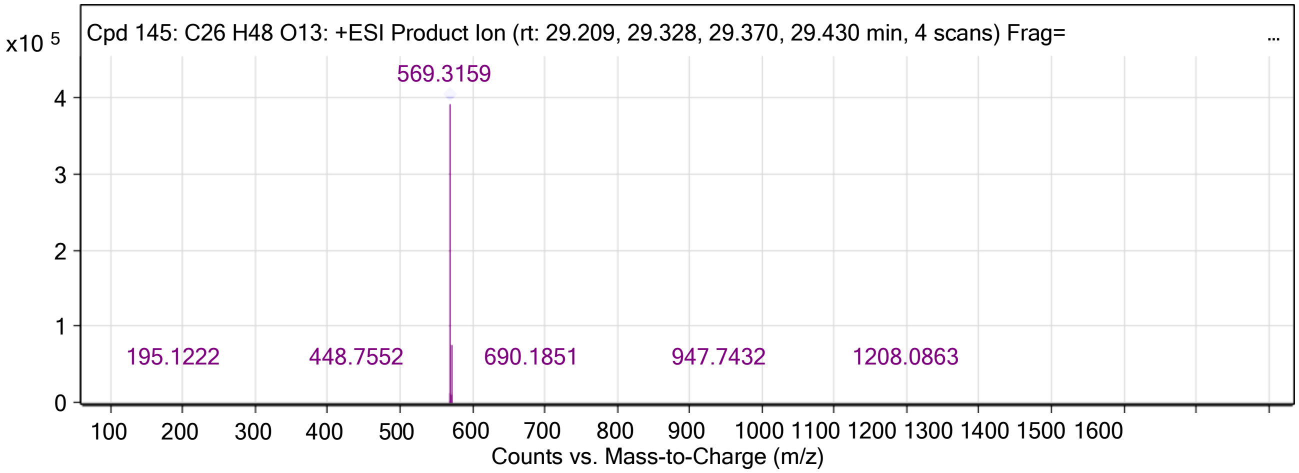

MS/MS Spectrum Peak List

m/z	Calc m/z	Diff(ppm)	z	Abund
233.1748	233.1747	-0.32		24.98
274.2138	274.2139	0.32		18.04
285.2067	285.206	-2.42		17.75
309.2786	309.2788	0.68		14.78
311.2924	311.2945	6.64	1	43.23
384.2005	384.199	-3.84		14.52
545.3238	545.3168	-12.81		21.73
566.301	566.2933	-13.53		44.44
569.3159	569.3168	1.54	1	391177.19
570.3184			1	76110.75

Compound Label	m/z	RT	Algorithm	Mass
Cpd 146: C28 H52 O14	613.3432	29.349	Auto MS/MS	612.3357

Compound Chromatograms

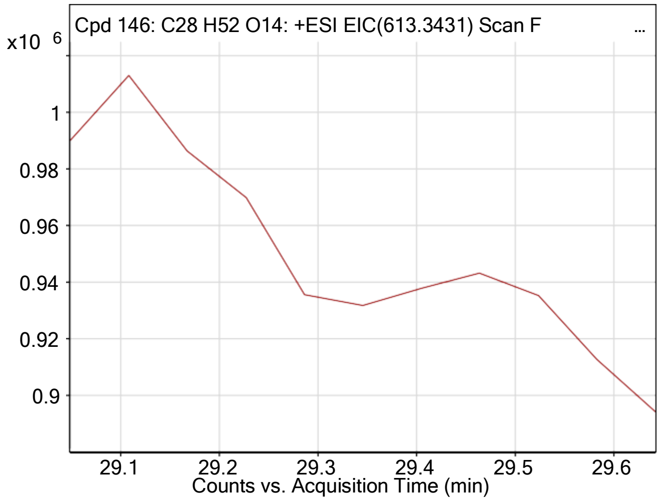

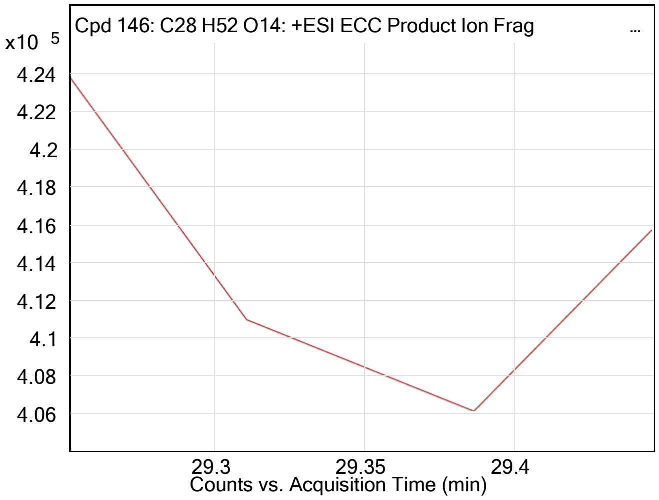

MS Spectrum

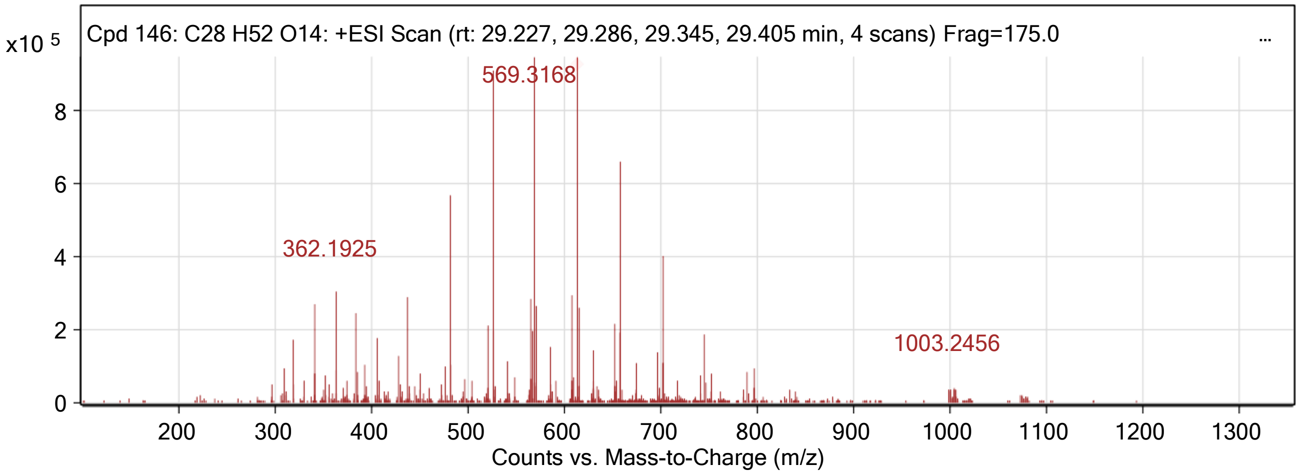

MS Zoomed Spectrum

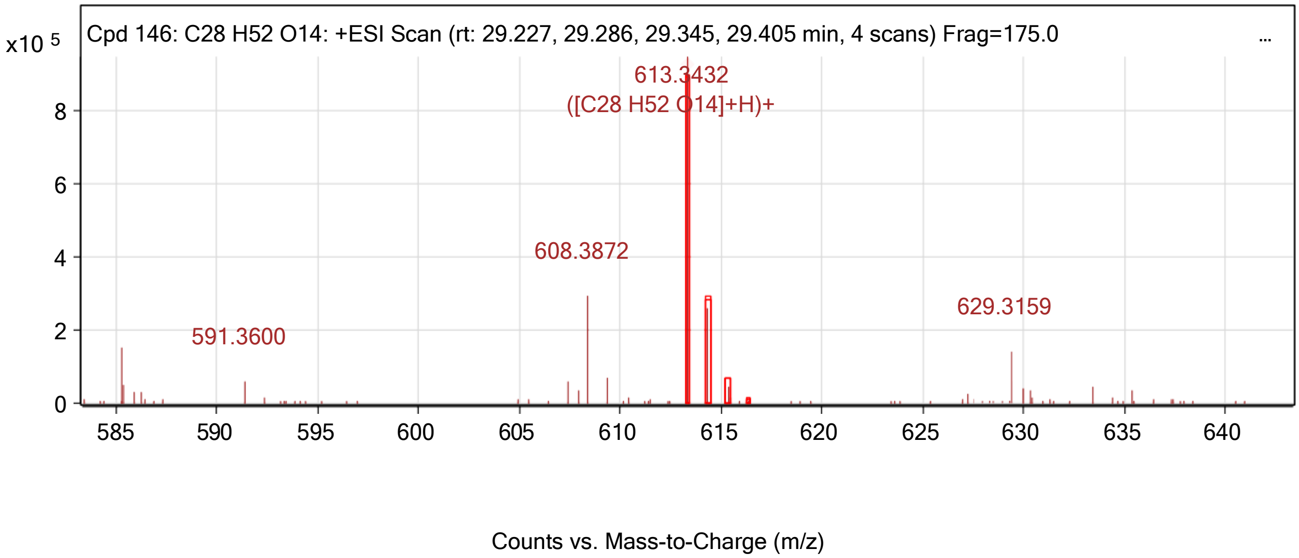

MS Spectrum Peak List

m/z	Calc *m/z*	Diff(ppm)	z	Abund	Formula	Ion
362.1925			2	300034.16		
481.2635			1	566605.13		
525.2904			1	904751.5		
569.3168			1	1088695.13		
613.3432	613.343	-0.29	1	943671	C28 H52 O14	(M+H)+
614.3458	614.3464	1.05	1	259902.72	C28 H52 O14	(M+H)+
615.3477	615.3489	1.91	1	46230.77	C28 H52 O14	(M+H)+
616.351	616.3515	0.95	1	6281.71	C28 H52 O14	(M+H)+
657.3693			1	658235.13		

MSMS Spectrum

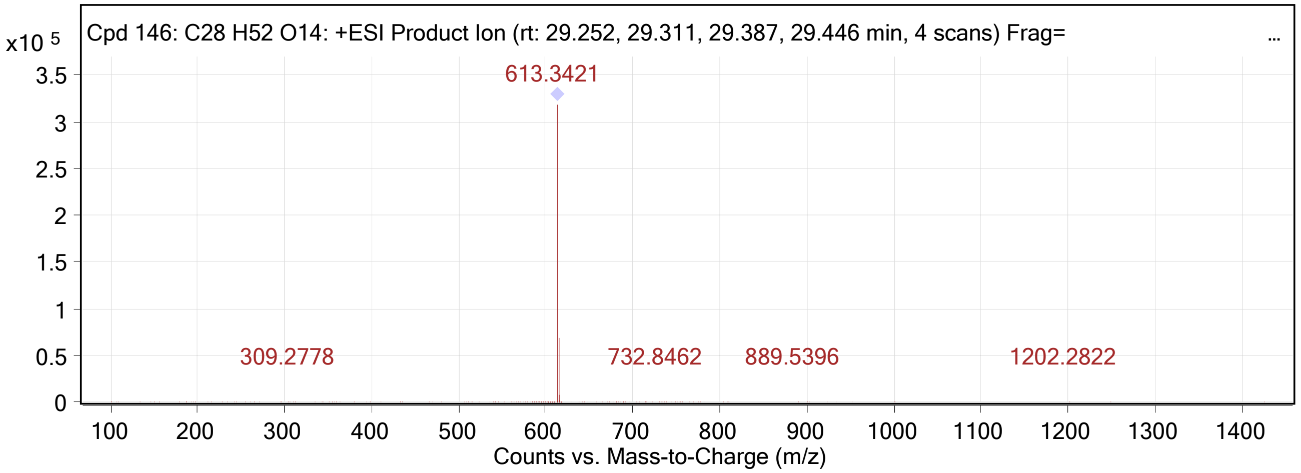

MS/MS Spectrum Peak List

m/z	*Calc m/z*	Diff(ppm)	z	Abund
133.0854	133.0859	3.58		27.13
309.2778	309.2788	3.2		79.54
311.2915	311.2945	9.43		51.03
357.0636	357.0664	7.67	1	33.61
363.2317	363.2377	16.57		18.33
393.2444	393.2483	9.98		18.74
610.3282	305.1595	-500007.57	2	37.13
613.3421	613.343	1.5	1	318847.59
614.3447			1	67826.17
615.347			1	8172.08

Compound Label	*m/z*	RT	Algorithm	Mass
Cpd 147: C24 H46 N3 O12	569.3166	29.608	Auto MS/MS	568.3092

Compound Chromatograms

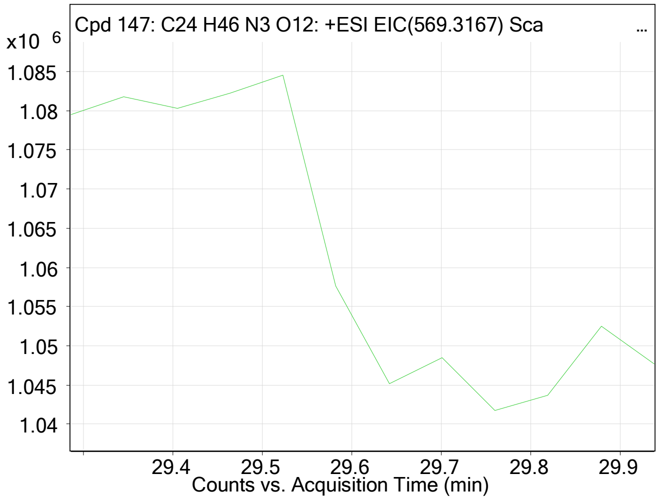

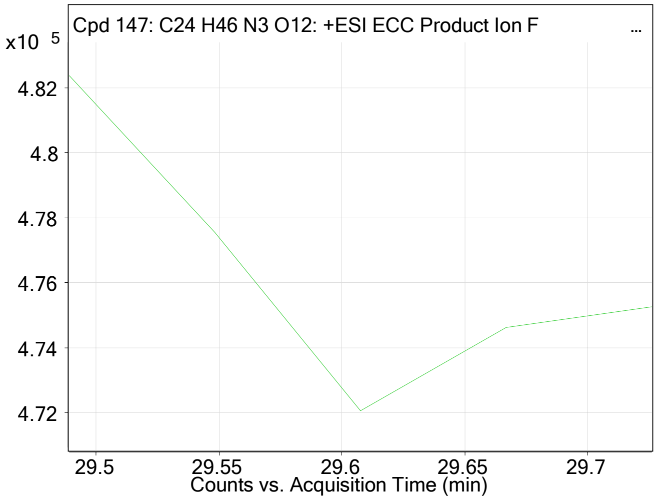

MS Spectrum

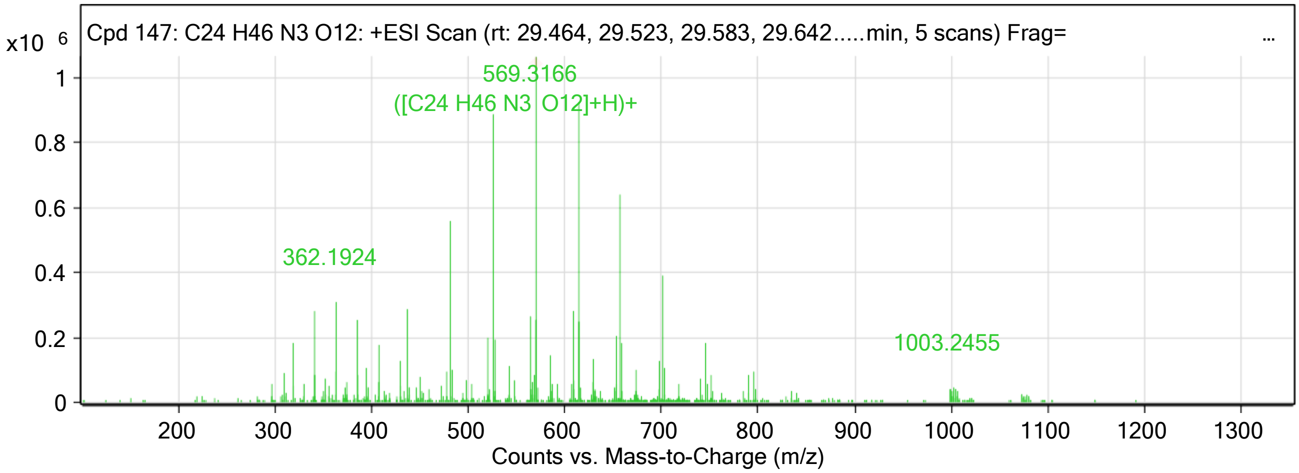

MS Zoomed Spectrum

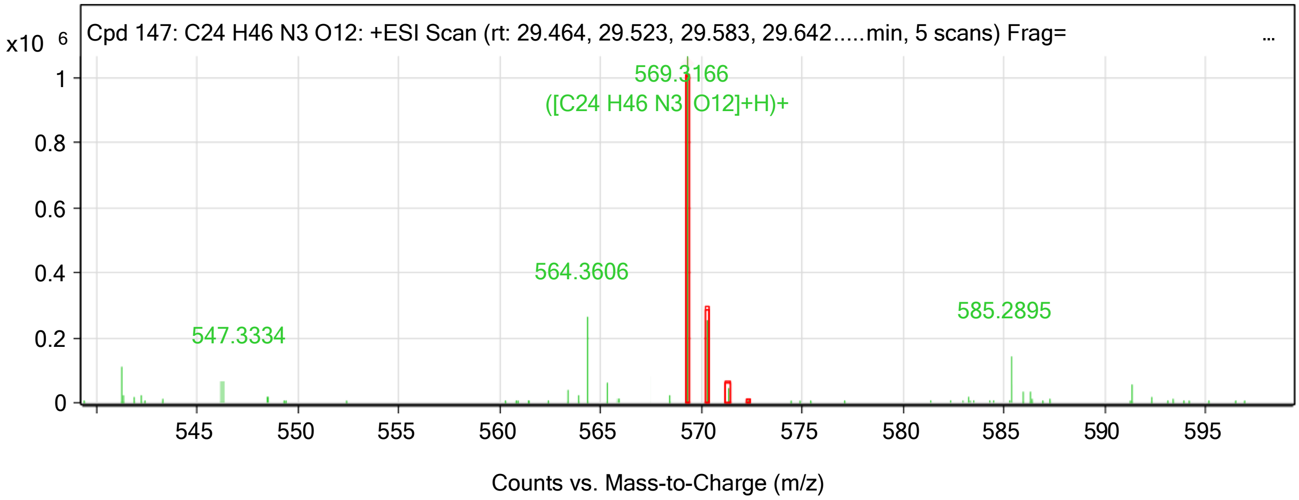

MS Spectrum Peak List

m/z	Calc *m/z*	Diff(ppm)	z	Abund	Formula	Ion
362.1924			2	309336.31		
481.2634			1	555476.44		
525.2903			1	882303.63		
569.3166	569.3154	-2.07	1	1063629.13	C24 H46 N3 O12	(M+H)+
570.3193	570.3186	-1.25	1	252301.5	C24 H46 N3 O12	(M+H)+
571.3214	571.3209	-0.8	1	42900.15	C24 H46 N3 O12	(M+H)+
572.3243	572.3235	-1.35	1	5619.08	C24 H46 N3 O12	(M+H)+
613.3431			1	919383.31		
657.3692			1	635559.13		
701.3952			1	389435.53		

MSMS Spectrum

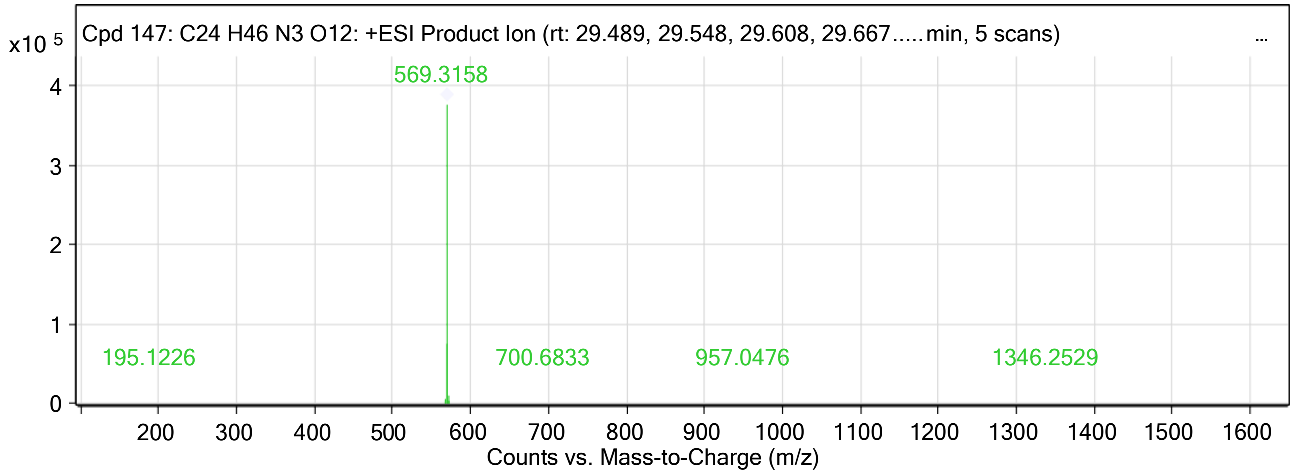

MS/MS Spectrum Peak List

m/z	*Calc m/z*	Diff(ppm)	z	Abund
133.0866	133.0859	-5.34		35.34
195.1226	195.1214	-6.56		38.89
263.1444	263.1476	11.89		12.98
309.2771	309.2775	1.02		38.16
311.2942	311.2945	0.84		31.57
538.2934	269.1482	-499997.11	2	22.63
566.2939	566.292	-3.46	1	30.54
567.2909			1	19.33
569.3158	569.3154	-0.63	1	375936.84
570.3184			1	73973.11

Compound Label	*m/z*	RT	Algorithm	Mass
Cpd 148: C28 H52 O14	613.3431	29.624	Auto MS/MS	612.3356

Compound Chromatograms

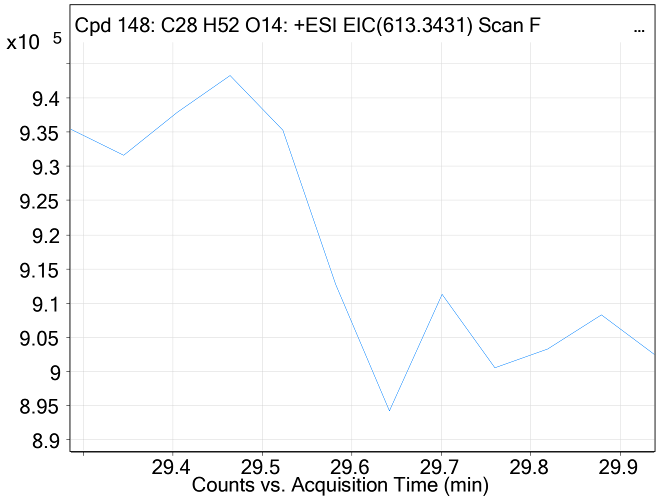

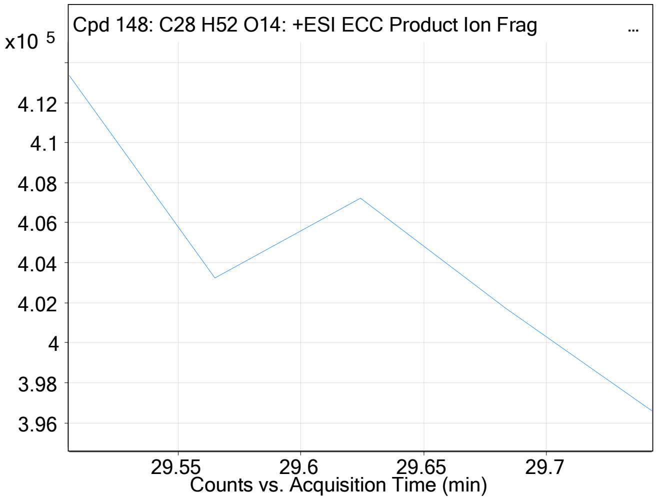

MS Spectrum

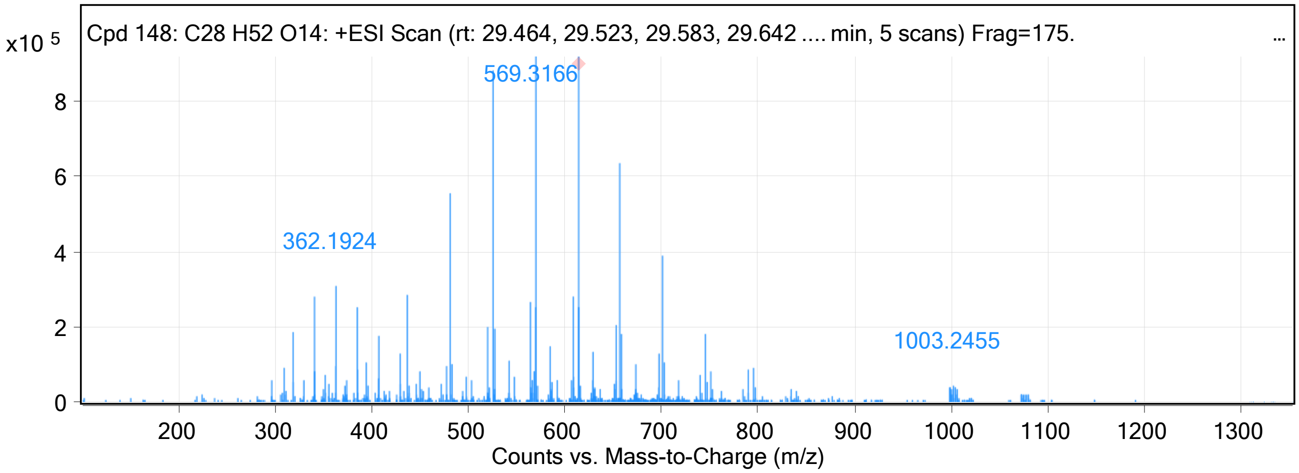

MS Zoomed Spectrum

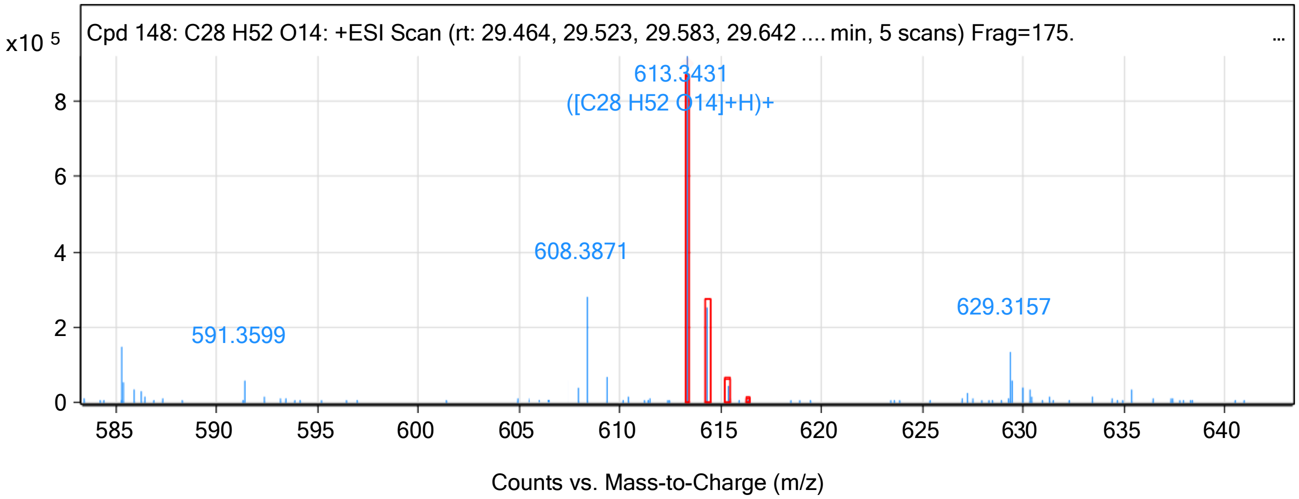

MS Spectrum Peak List

m/z	Calc *m/z*	Diff(ppm)	z	Abund	Formula	Ion
362.1924			2	309336.31		
481.2634			1	555476.44		
525.2903			1	882303.63		
569.3166			1	1063629.13		
613.3431	613.343	-0.11	1	919383.31	C28 H52 O14	(M+H)+
614.3457	614.3464	1.22	1	249711.97	C28 H52 O14	(M+H)+
615.3477	615.3489	1.85	1	45267.2	C28 H52 O14	(M+H)+
616.3516	616.3515	-0.09	1	6131.24	C28 H52 O14	(M+H)+
657.3692			1	635559.13		
701.3952			1	389435.53		

MSMS Spectrum

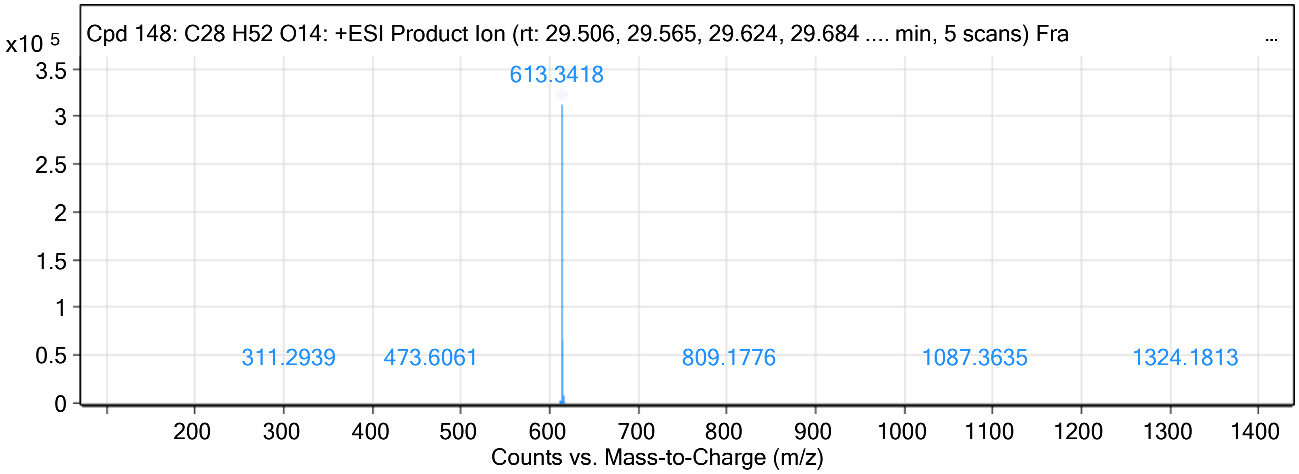

MS/MS Spectrum Peak List

m/z	*Calc m/z*	Diff(ppm)	z	Abund
133.0863	133.0859	-3.01		45.63
145.016	145.0131	-19.5		19
221.1373	221.1384	4.95		28.13
296.151	296.1466	-15.01		15.85
307.2203	307.2268	21.07		13.01
309.1973	309.1908	-21.16		13
309.2787	309.2788	0.43		38.67
311.2939	311.2945	1.89	1	66.63
357.0665	357.0664	-0.48	1	16.55
613.3418	613.343	1.93	1	312112.31

Compound Label	*m/z*	RT	Algorithm	Mass
Cpd 149: C26 H48 O13	569.3167	29.874	Auto MS/MS	568.3093

Compound Chromatograms

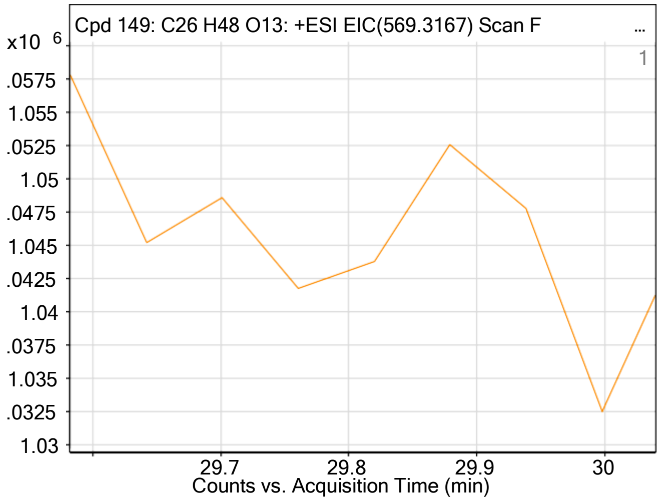

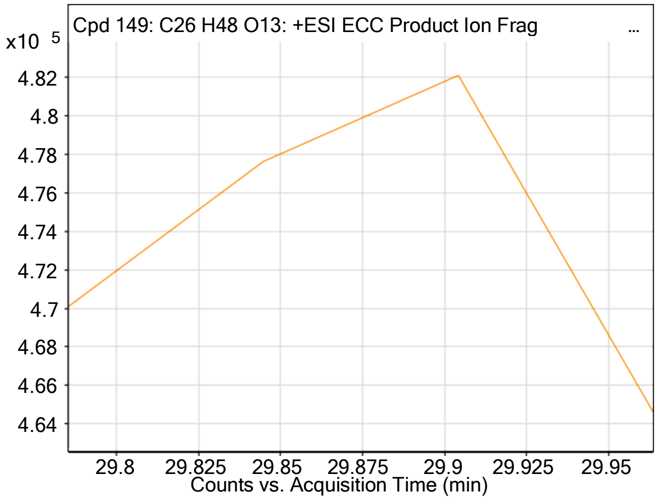

MS Spectrum

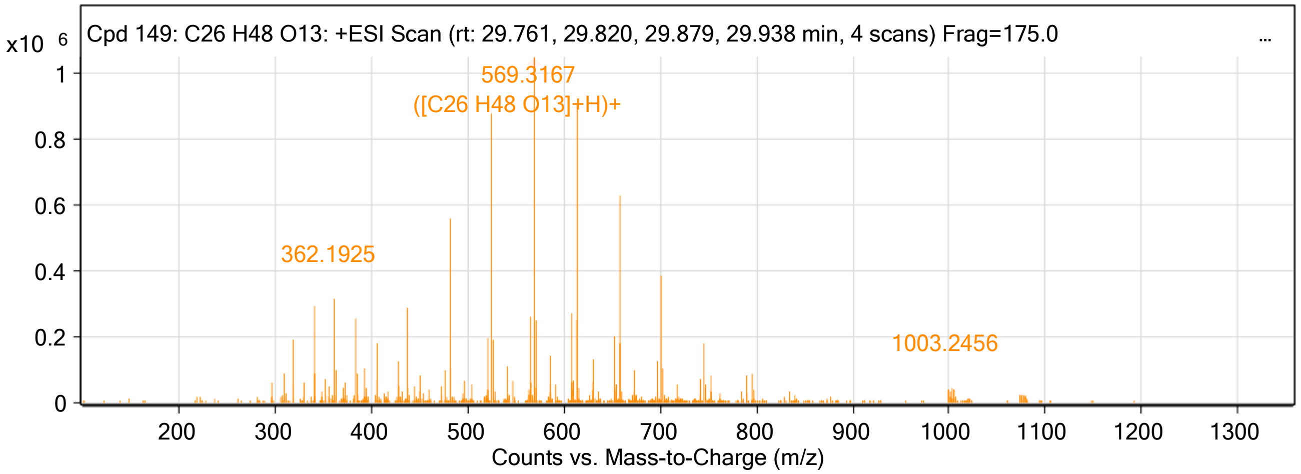

MS Zoomed Spectrum

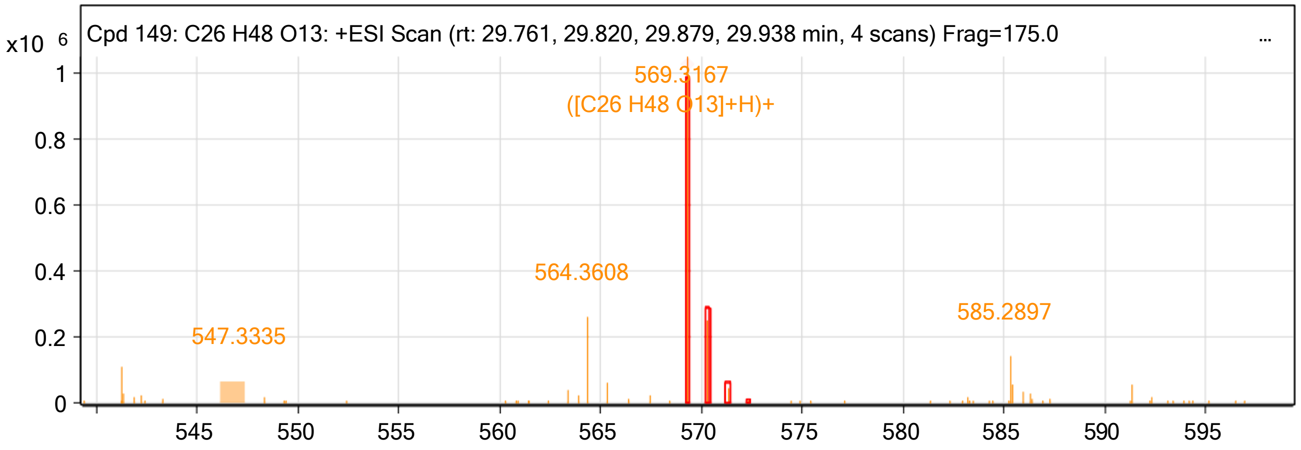

Counts vs. Mass-to-Charge (m/z)

MS Spectrum Peak List

m/z	Calc *m/z*	Diff(ppm)	z	Abund	Formula	Ion
362.1925			2	312462		
481.2636			1	557406.63		
525.2905			1	875276.31		
569.3167	569.3168	0.06	1	1046445.19	C26 H48 O13	(M+H)+
570.3195	570.3202	1.28	1	249569.02	C26 H48 O13	(M+H)+
571.3216	571.3226	1.82	1	42723.23	C26 H48 O13	(M+H)+
572.3243	572.3253	1.77	1	6011.05	C26 H48 O13	(M+H)+
613.3432			1	903692.19		
657.3693			1	628058.75		

MSMS Spectrum

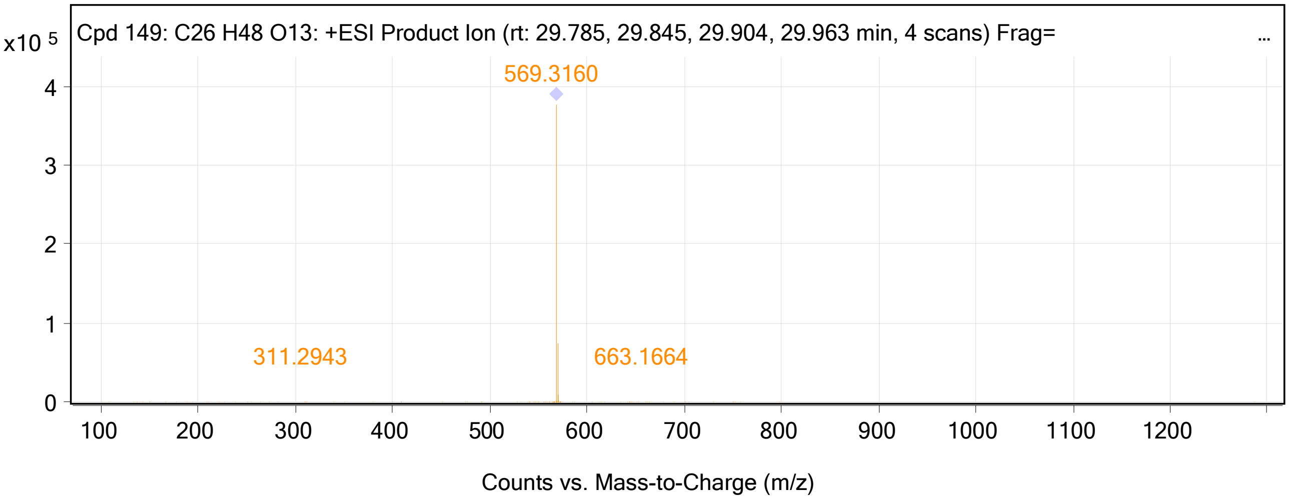

MS/MS Spectrum Peak List

m/z	*Calc m/z*	Diff(ppm)	z	Abund
142.0981	142.0988	5.34		15.06
177.1112	177.1121	5.47		13
211.0925	211.0965	18.74		14.03
227.1277	227.1278	0.51		13.26
263.1897	263.1853	-16.71		28.82
311.2943	311.2945	0.56		52.45
408.2317	408.2354	8.91		13.81
569.316	569.3168	1.36	1	376993.81
570.3186			1	74450.87
571.3208			1	8931.74

Compound Label	*m/z*	RT	Algorithm	Mass
Cpd 150: C28 H52 O14	613.3432	29.891	Auto MS/MS	612.3357

Compound Chromatograms

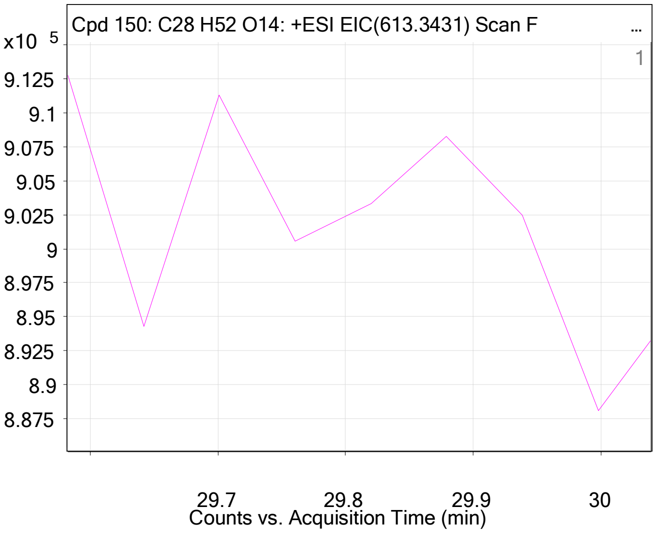

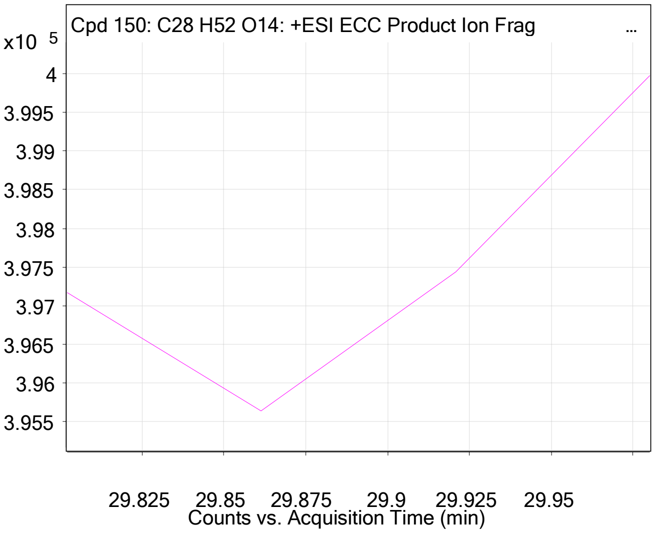

MS Spectrum

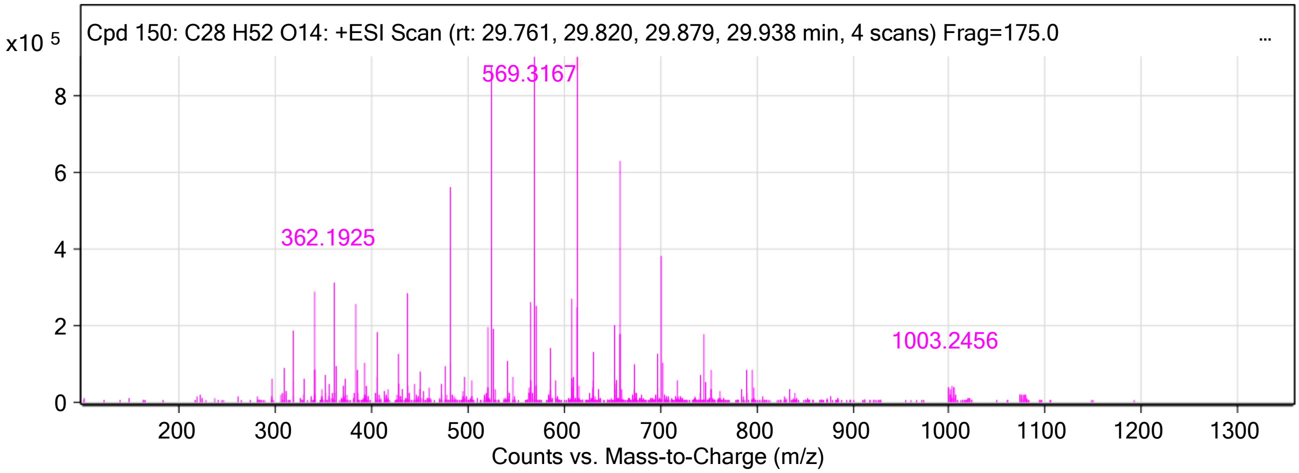

MS Zoomed Spectrum

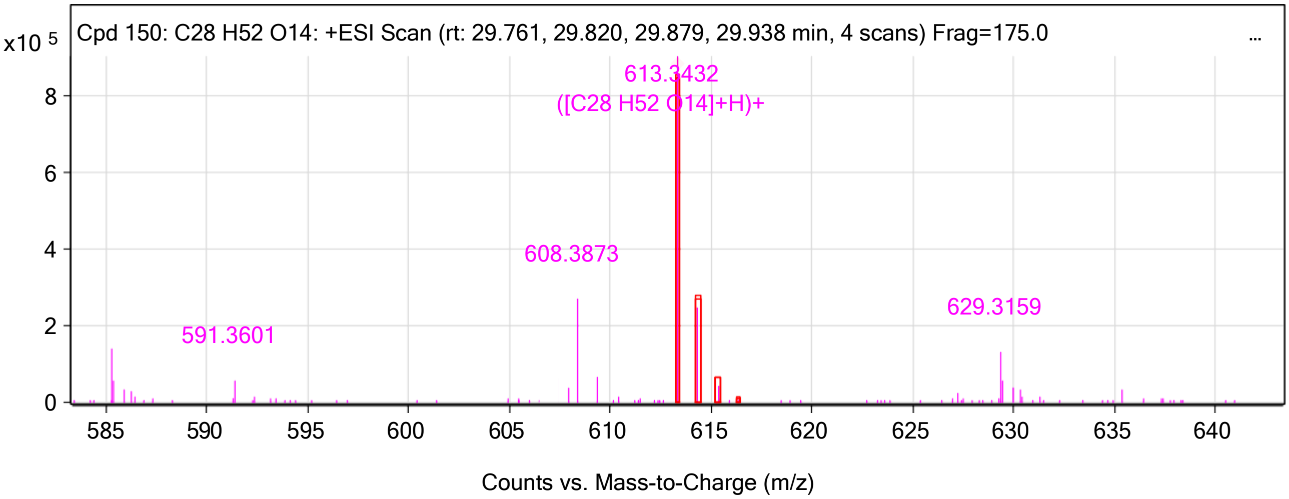

MS Spectrum Peak List

m/z	Calc *m/z*	Diff(ppm)	z	Abund	Formula	Ion
362.1925			2	312462		
481.2636			1	557406.63		
525.2905			1	875276.31		
569.3167			1	1046445.19		
613.3432	613.343	-0.39	1	903692.19	C28 H52 O14	(M+H)+
614.3458	614.3464	0.99	1	248462.2	C28 H52 O14	(M+H)+
615.3478	615.3489	1.78	1	44215.35	C28 H52 O14	(M+H)+
616.3514	616.3515	0.29	1	6187.29	C28 H52 O14	(M+H)+
657.3693			1	628058.75		
701.3954			1	384081.63		

MSMS Spectrum

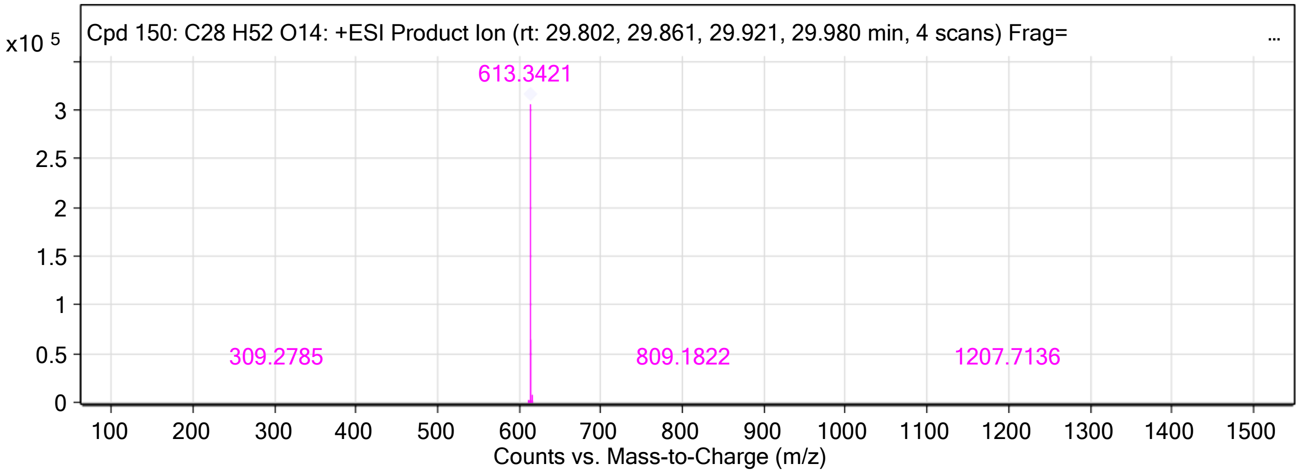

MS/MS Spectrum Peak List

m/z	*Calc m/z*	Diff(ppm)	z	Abund
175.1352	175.1329	-13.22		17.16
221.1371	221.1384	5.52		17.34
233.1771	233.1747	-10.06		18.27
252.2019	252.2084	25.78		17.39
309.2785	309.2788	1.03	1	84.71
329.2268	329.2323	16.53		15.49
357.0697	357.0664	-9.2		13
529.3314	529.3371	10.74		15.32
610.3121	305.1595	-499994.39	2	33.67
613.3421	613.343	1.43	1	305906.91

--- End Of Report ---
